# Assessing the non-inferiority of prosthesis constructs used in hip replacement using data from the National Joint Registry of England, Wales, Northern Ireland and the Isle of Man: a benchmarking study

**DOI:** 10.1136/bmjopen-2018-026685

**Published:** 2019-04-29

**Authors:** Kevin C Deere, Michael R Whitehouse, Martyn Porter, Ashley W Blom, Adrian Sayers

**Affiliations:** 1 Musculoskeletal Research Unit,Translational Health Sciences, Bristol Medical School, University of Bristol, Bristol, UK; 2 National Institute for Health Research,Bristol Biomedical Research Centre, University Hospitals Bristol NHS Foundation Trust and University of Bristol, Bristol, UK; 3 Centre for Hip Surgery, Wrightington Hospital, Wigan, UK; 4 Population Health Sciences, Bristol Medical School, University of Bristol, Bristol, UK

**Keywords:** hip arthroplasty, non-inferiority, national joint registry, benchmarking, medical devices

## Abstract

**Objectives:**

To investigate the relative performance of hip prosthesis constructs as compared with the best performing prosthesis constructs and illustrate the substantial variability in performance of currently used prostheses.

**Design:**

A non-inferiority study.

**Setting:**

The National Joint Registry for England, Wales, Northern Ireland and the Isle of Man (NJR).

**Participants:**

All patients with a primary total hip replacement registered in the NJR between 1 April 2003 and 31 December 2016.

**Main outcome measures:**

Kaplan-Meier failure function for hip prosthesis constructs. Failure difference between best performing construct and remaining constructs.

**Methods:**

Using a non-inferiority analysis, the performance of hip prosthesis constructs by brand were compared with the best performing contemporary construct. Construct failure was estimated using the 1-Kaplan-Meier survival function method, that is, an estimate of net failure. The difference in failure between the contemporary benchmark and all other constructs was tested.

**Results:**

Of the 4442 constructs used, only 134 had ≥500 procedures at risk at 3 years postprimary, 89 of which were not demonstrated to be inferior to the benchmark by at least 100% relative risk. By 10 years postprimary, there were 26 constructs with ≥500 at risk, 13 of which were not demonstrated to be inferior by at least 20% relative risk.

Even fewer constructs were not inferior to the benchmark when analysed by age and gender. At 5 years postprimary, there were 15 constructs in males and 11 in females, aged 55–75 years, not shown to be inferior.

**Conclusions:**

There is great variability in construct performance and the majority of constructs have not been demonstrated to be non-inferior to contemporary benchmarks. These results can help to inform patients, clinicians and commissioners when considering hip replacement surgery.

**Strengths and limitations of this study**

- Data collected from the largest joint registry in the world.
- For the first time, we have explicitly compared the performance of prosthesis constructs to a contemporary reference.
- Unambiguous presentation of data allows surgeons, patients and policy makers to directly compare commonly used prosthesis constructs to a reference construct.
- Residual and unmeasured confounding factors are likely to be present.
- The number of patients remaining at risk after extended follow-up is low, and therefore the power to detect non-inferiority after extended follow-up is also low.

## Introduction

When patients are considering a hip replacement, they would be forgiven for thinking that all hip prostheses function equally.^1^ However, all prostheses are not equal as evidenced by the failure of the 3M Capital hip implant and metal-on-metal bearings.^2^ The extent to which patients and clinicians are aware of this lack of equality is unclear.

The National Joint Registry for England, Wales, Northern Ireland and the Isle of Man (NJR) was established to monitor the effectiveness of different types of joint replacement surgery, improve clinical standards and to identify poorly performing implants. It has not focused on identifying exceptionally well-performing implants due to limitations inherent with routine data collection and interpreting data from a standpoint of cause and effect. The NJR publishes the unadjusted cumulative failure rates of the most commonly used stem and cup brand combinations used in hip replacement surgery.^3^

Therefore, the role of promoting perceived good practice has been filled by other organisations such as the Orthopaedic Device Evaluation Panel (ODEP) in the UK,^4^ the Netherlands Orthopaedic Association in the Netherlands^5^ and the Australian superior clinical performance programme.^6^ Benchmarking bodies typically attempt to provide some type of classification to describe whether an implant is functioning at an acceptable level or not.

In the absence of evidence from randomised control trials, benchmarking organisations and prostheses registries are currently the best sources of evidence for prosthesis performance. However, both registries and benchmarking bodies have limitations which make the interpretation of prosthesis, or prosthesis construct, performance difficult. The cumulative failure reported by the NJR gives an indication of implant construct performance in absolute terms, but head-to-head comparison of different constructs is difficult to estimate without more advanced statistical manipulation. The ODEP grading system is focused on individual implants rather than the constructs they form and is based on meeting an acceptable externally decided benchmark. This simple dichotomisation does not facilitate comparison between the many different prosthesis constructs being used today or illustrate the extensive variability in so-called well-performing prostheses.^7^

Sayers *et al* recently proposed a method of comparison for joint replacement prostheses using a non-inferiority design against an external benchmark.^8^ However, the primary limitation of this method is the arbitrary requirement for an externally specified benchmark.

In a non-inferiority clinical trial^9^ that has failure as an outcome, two treatments (comparator and reference) can be directly compared to ensure that the comparator treatment is within a clinically acceptable range (non-inferiority margin) of performance at a specified point in time.^10 11^ Therefore, standard methods for conducting non-inferiority trials could be applied in an orthopaedic benchmarking setting, assuming an appropriate comparator, non-inferiority margin and time of interest can be identified. This is a method we have applied in a medical device setting, namely knee replacements using NJR data, in which we assessed the non-inferiority of knee replacement constructs as compared with a benchmark construct.^12^

Choosing an appropriate contemporary reference is difficult. There is no evidence from randomised trials that suggests any prosthesis construct outperforms all others, therefore the choice of reference is more heuristic. Patients would like to receive the best available care and clinicians would like to provide the best possible care, or at least care that is non-inferior to the best. Therefore, the natural choice of reference against which all other prostheses should be compared is the construct with the lowest failure rate. However, in order to protect against chance, good fortune and a low observed failure rate, the construct should be used in large enough numbers to mitigate sampling variability.

As the failure rate of prostheses is known to be influenced by both age and gender, the choice of reference should reflect this specificity,^13^ whereas the selection of an appropriate time and non-inferiority margin to assess prosthesis performance is much more subjective, as is the reader’s specific interest. For example, a surgeon interested in an older patient with lower life expectancy may be interested in minimising short-term complications opposed to ensuring long-term implant survivorship.

The aim of this study is to investigate the relative performance of hip prosthesis constructs as compared with the best performing prosthesis constructs using a non-inferiority study design, and illustrate the substantial variability in performance of currently used prostheses. Stem, bearing and cup brand combinations (constructs) are examined against non-inferiority margins of 20% relative risk and 100% relative risk at 3, 5, 7 and 10 years following surgery.

## Methods

### Patients and data sources

We identified all patients with a primary total hip replacement (THR) registered in the NJR between 1 April 2003 and 31 December 2016. All patients were consented to be included in the NJR as part of the standard NJR process.

Procedures were included if the bearing surface was either metal-on-polyethylene (MoP), ceramic-on-polyethylene (CoP) or ceramic-on-ceramic (CoC). Procedures using any other bearing surfaces were excluded as were hemiarthroplasty procedures. Procedures were also excluded if the patient age and gender were missing, or the National Health Service number was untraceable and therefore mortality unknown. Metal-on-metal prosthesis constructs were excluded as their very high failure rates across all ages and both genders have already been demonstrated^2 14^ and their use no longer reflects contemporary practice.^7^

### Patient and public involvement

Patient representatives sit on the committee structure of the National Joint Registry. The research priorities of the National Joint Registry are identified by this committee structure and approved by the patient representatives. Patients were not involved in the setting of the research question or the outcome measures, nor were they involved in designing or implementing this work or interpretation of the results. We are unable to disseminate results of this study directly to study participants due to the anonymous nature of the data. We plan to disseminate our findings to the National Joint Registry, via their communications team, to relevant to the provision of joint replacement and to the general population through the local and national press.

### Primary exposure

The primary exposure used in this analysis is hip prosthesis construct. This is defined by the femoral stem, acetabular cup combination and bearing combination. Groupings were defined using data recorded by the NJR and based on the catalogue numbers of individual hip prosthesis.

### Statistical methods

Using a non-inferiority analysis, the performance of hip prosthesis constructs was compared with an internally identified reference group. Prosthesis construct failure was estimated using the 1-Kaplan-Meier method, that is, an estimate of net failure.

Failure is defined using the first linked surgical revision; patients were censored at death or administratively censored on 31 December 2016. In a recent national audit of NJR procedure recording compliance,^15^ the percentage capture rates were 95.7% and 90.3% for primary and revision procedures, respectively. The difference in stratum-specific failure probabilities compared with the reference were calculated at 3, 5, 7 and 10 years for all prosthesis (stem–cup) combinations, stratified by gender and stratified by gender and age group (<55, 55–75 and >75 years).

The difference and 95% CI of the difference between the comparator prosthesis construct and the reference prosthesis construct was estimated at the specified time points. The SE of the difference was constructed using a pooled estimate of the Greenwood SE,^16^

$\overset{\overset{⌢}{SE(Diff)}}{}=\sqrt{GSE_{xi}^{2}\;+\;GSE_{ref}^{2}}$ and a z-test comparing the difference between the reference and test prosthesis was then constructed,

$$Z=\left(\left(\overset{⌢}{F_{xi}}-\;\overset{⌢}{F_{ref}}\right)+\delta \right)/\overset{⌢}{SE\left(Diff\right)}.$$

The stratum-specific contemporaneous reference construct was selected as the stem–cup and bearing combination with the lowest failure rate with at least 1000 patients at risk at the time point of interest. The choice of 1000 procedures of the same construct was based on simulation work by Sayers *et al* which demonstrated that 1000 procedures at risk will give rise to a CI width of ~3% (±1.5%).^8^ We believe this represents an acceptable minimal level of accuracy to be considered a suitable reference standard.

Two non-inferiority margins were chosen to illustrate the sensitivity of the choice. The first margin was conservatively set at a 20% increase in relative risk of failure compared with the reference, in line with clinical trials using this methodology, although towards the upper end.^17^ The second was a 100% increase in relative risk, that is, a doubling in cumulative probability of failure, as this is an easily interpretable outcome.

If a construct had 500 or more patients still at risk, at each time point, we calculated the difference in failure between that construct and the reference construct. Results are graphically reported for all comparator prosthesis constructs meeting this criterion at each time point of interest. These figures show the failure difference for each construct against the reference and the number of constructs still at risk. The threshold for graphical presentation, 500 procedures, was chosen based on the previous work of Sayers *et al* 8 as this would give rise to an individual CI width of ~5% (±2.5%), and because it complements the number of procedures at risk used by ODEP when evaluating devices at 10 years. However, as this decision is somewhat arbitrary, we also present results in a tabular format for all comparator prosthesis constructs with at least 250 patients at risk at the beginning of the time point of interest (see online supplementary tables).

10.1136/bmjopen-2018-026685.supp1 Supplementary data

**Figure 1:**
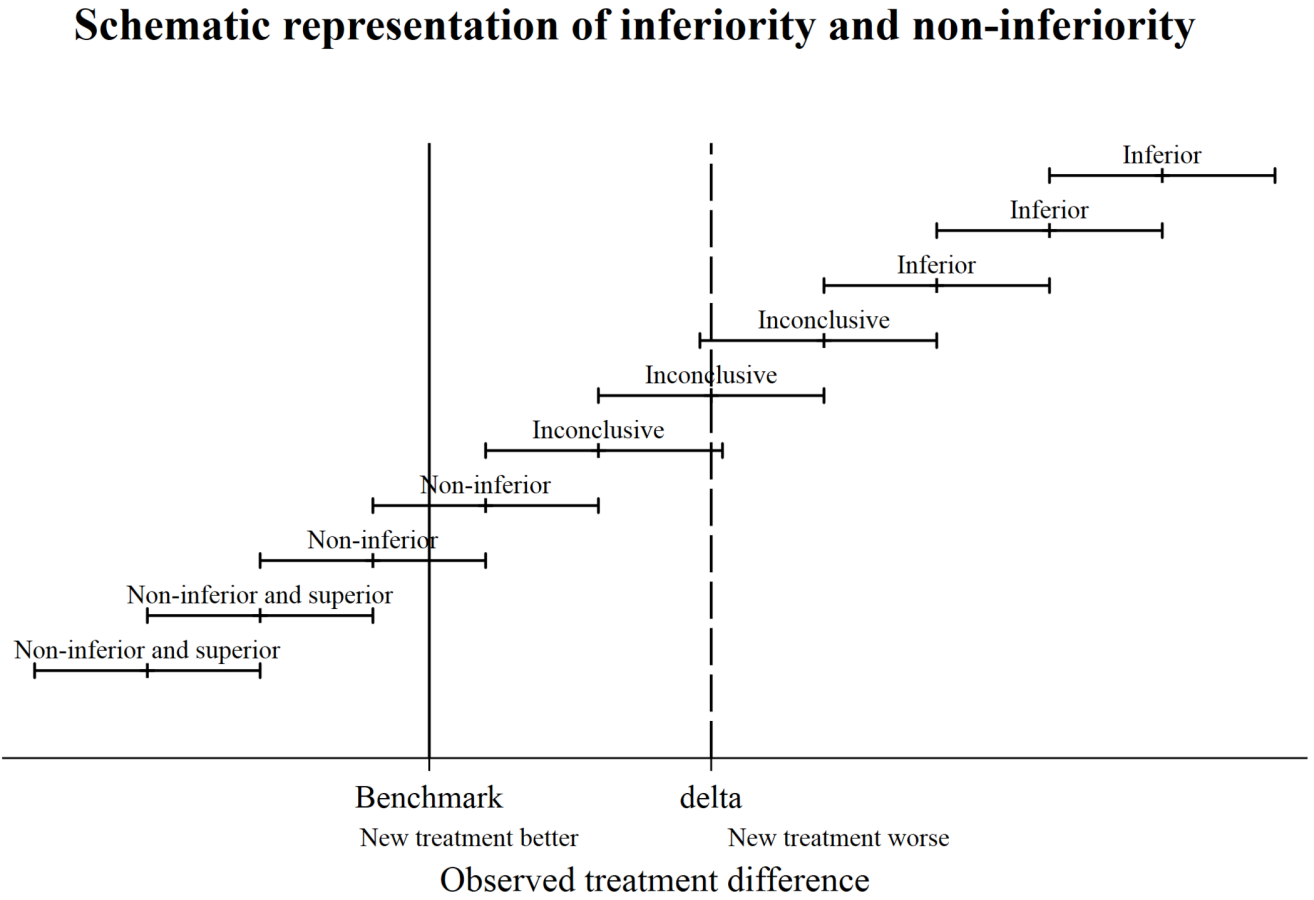
Schematic representation of inferiority and non-inferiority.

Prosthesis constructs were either classified as non-inferior, inconclusive or inferior. If the upper CI limit is less than or equal to the 20% non-inferiority margin, the prosthesis construct was non-inferior. If the lower CI of the difference was greater than the non-inferiority margin at either 20% or 100% the prosthesis construct was classed as inferior at 20% or 100%, respectively. If the lower confidence limit is less than the non-inferiority margin, and the upper confidence limit greater than non-inferiority margin the construct was described as inconclusive, see figure 1 for graphical representation of the classification.

### Sensitivity analysis

We repeated all analyses using a historic reference group, this specified the reference at 3, 5 and 7 years as the best performing stem–cup prosthesis construct at 10 years with at least 1000 still at risk in the stratum of interest.

All analyses were carried out using Stata V.14.2.

## Results

There were 890 681 primary hip replacements included in the NJR between 1 April 2003 and 31 December 2016. Following the application of the exclusion criteria defined above, 797 178 procedures were included in the final analysis, see online supplementary figure 1. In total, 4442 different prosthesis constructs were used at least once. A detailed description of non-inferiority across all procedures is provided. Due to the large number of clinically relevant subdivisions and sensitivity analyses, results will be described more broadly. Constructs are described using bearing and brand. Bearings are either ceramic (C), metal (M) or polyethylene (P). Brands are described listing the stem and cup combination (stem/cup).

Figures were produced for each stratification of gender, age group and time since primary. To view data at 3 years postprimary for all men, men aged <55 years, men aged 55–75 years and men aged >75 years, see online supplementary figures 2a–d, respectively. To view data at 5 years postprimary for all men, men aged <55 years, men aged 55–75 years and men aged >75 years, see online supplementary figures 3a–d, respectively. To view data at 7 years postprimary for all men, men aged 55–75 years and men aged >75 years, see online supplementary figures 4a–c, respectively. To view data at 10 years postprimary for all men and men aged 55–75 years, see online supplementary figures 5a and b, respectively. To view data at 3 years postprimary for all women, women aged <55 years, women aged 55–75 years and women aged >75 years, see online supplementary figures 6a–d, respectively. To view data at 5 years postprimary for all women, women aged <55 years, women aged 55–75 years and women aged >75 years, see online supplementary figures 7a–d, respectively. To view data at 7 years postprimary for all women, women aged <55 years, women aged 55–75 years and women aged >75 years, see online supplementary figures 8a–d, respectively. To view data at 10 years postprimary for all women and women aged 55–75 years, see online supplementary figures 9a and b, respectively

Estimates for the difference in failure between the reference and comparator prosthesis constructs with ≥250 procedure at risk at the time of interest for all, and for each stratification of gender and age group were tabulated. To view data for all at 3, 5, 7 and 10 years postprimary, see online supplementary tables 1a–d, respectively. To view data in all women at 3, 5, 7 and 10 years postprimary, see online supplementary tables 2a–d, respectively. To view data for women aged <55 years at 3, 5 and 7 years postprimary, see online supplementary tables 3a–c, respectively. To view data for women between 55 and 75 years at 3, 5, 7 and 10 years postprimary, see online supplementary tables 4a–d, respectively. To view data for women aged >75 years at 3, 5 and 7 years postprimary, see online supplementary tables 5a–c, respectively. To view data in all men at 3, 5, 7 and 10 years postprimary, see online supplementary tables 6a–d, respectively. To view data in men aged <55 years at 3 and 5 years postprimary, see online supplementary tables 7a and b, respectively. To view data in men between 55 and 75 years at 3, 5, 7 and 10 years postprimary, see online supplementary tables 8a–d, respectively. To view data in men aged >75 years at 3, 5 and 7 years postprimary, see online supplementary tables 9a–c, respectively. In this analysis, there were 415 608 implants at risk at 3 years (in constructs with at least 500 procedures) and 41 908 at 10 years. Of these, there were 3733 implant failures at 3 years and 1325 at 10 years. The total number of implants at risk and total implant failures for each subdivision and time point can be seen in online supplementary tables 10a and b, respectively.

### Non-inferiority: all procedures

**Figure 2:**
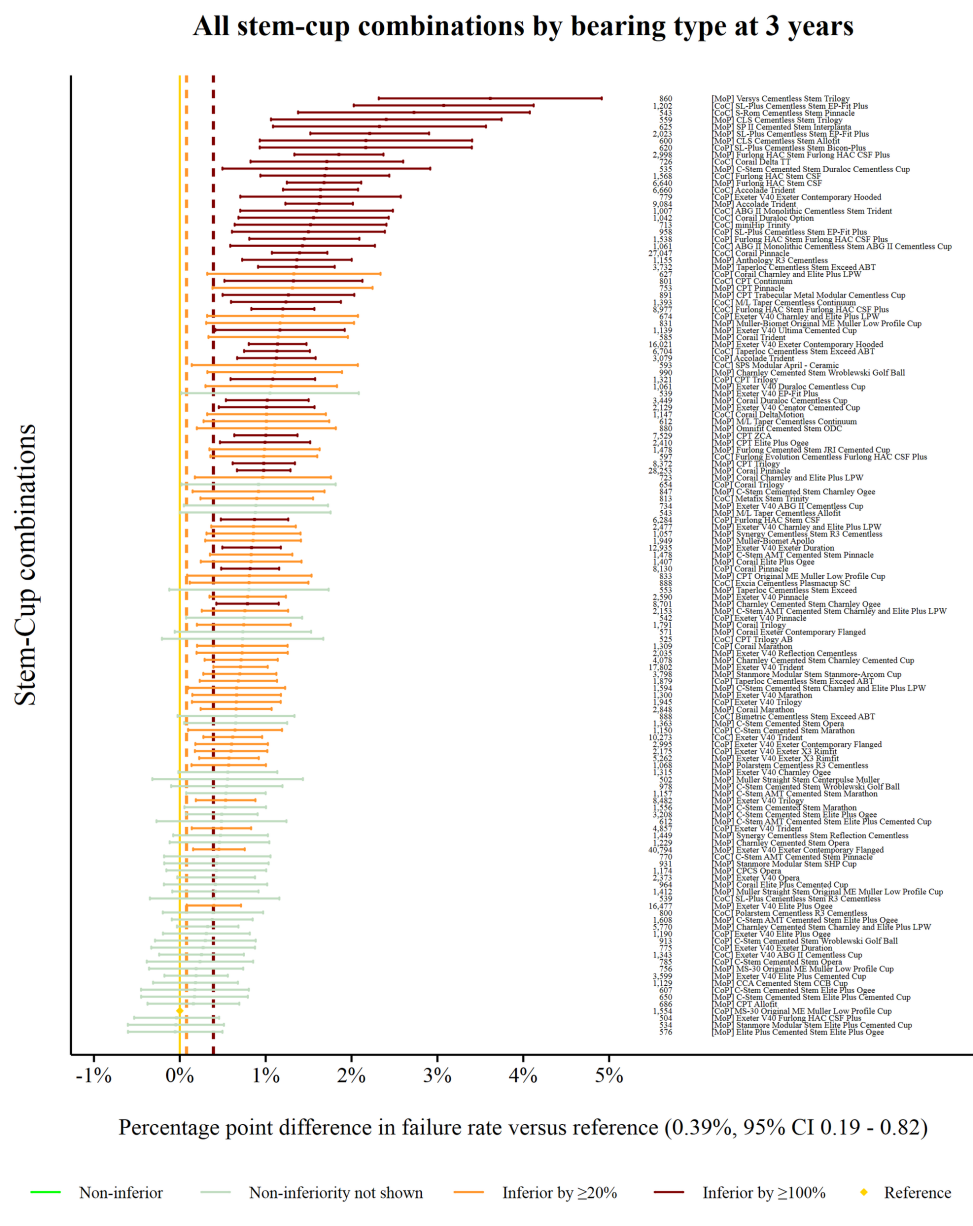
Difference in failure of implanted constructs compared with a contemporary reference at 3 years, using all stem–cup combinations with ≥500 procedures remaining at risk. CoC, ceramic-on-ceramic; CoP, ceramic-on-polyethylene; MoP, metal-on-polyethylene.

The reference prosthesis construct at 3 years was identified as the CoP MS-30/Low profile Muller. There were 1554 procedures remaining at risk and the failure rate was 0.39% (95% CI 0.19 to 0.82). There were 134 prosthesis combinations with ≥500 procedures at risk. Ninety combinations were classified as inferior to the reference by at least 20% relative risk of failure. Forty-four of the 90 were shown to be inferior by at least 100% relative risk (figure 2). No prosthesis constructs could be described as non-inferior.

**Figure 3:**
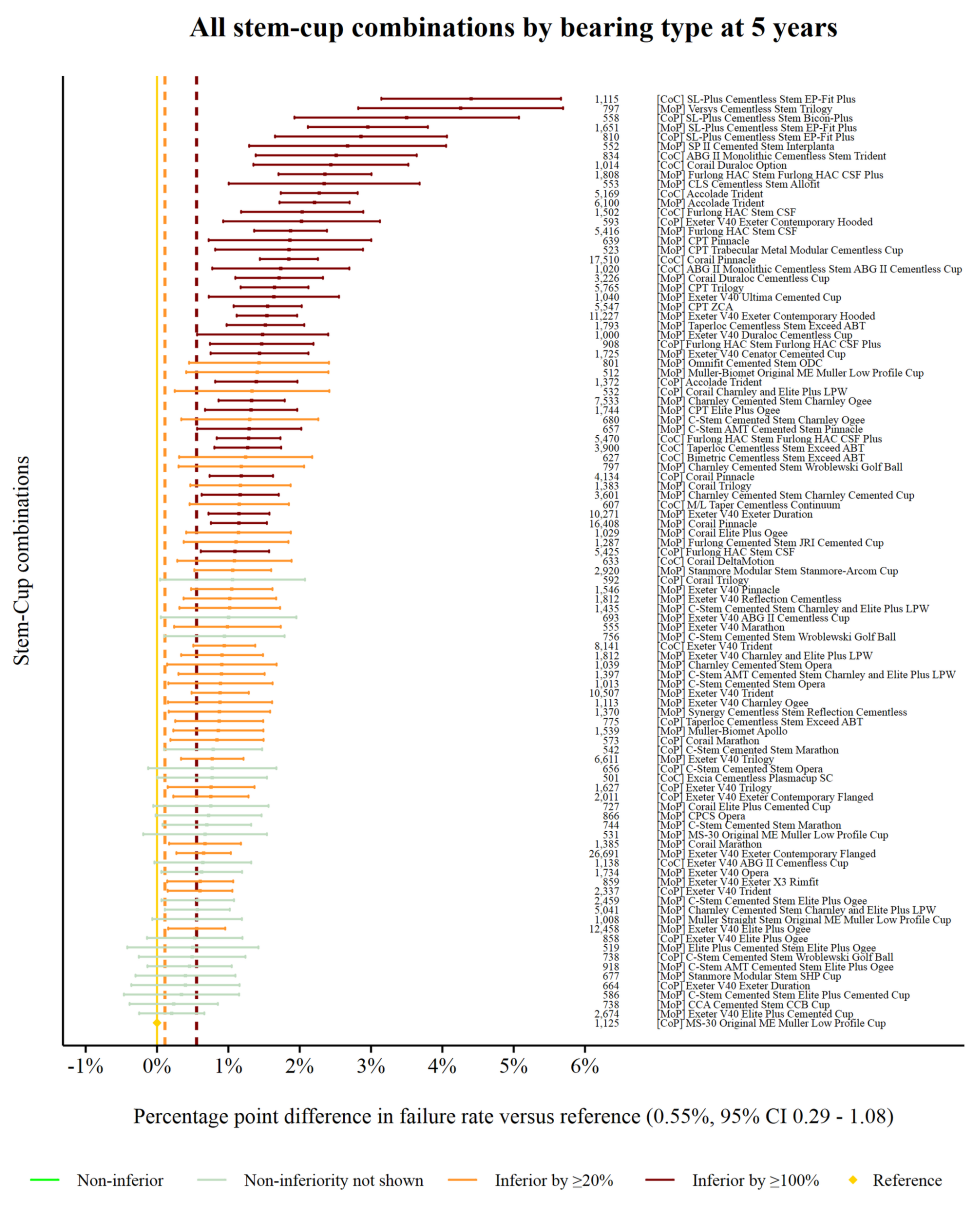
Difference in failure of implanted constructs compared with a contemporary reference at 5 years, using all stem–cup combinations with ≥500 procedures remaining at risk. CoC, ceramic-on-ceramic; CoP, ceramic-on-polyethylene; MoP, metal-on-polyethylene.

The reference prosthesis construct at 5 years was again identified as CoP MS-30/Low profile Muller. There were 1125 procedures remaining at risk and the failure rate was 0.55% (95% CI 0.29 to 1.08). There were 99 prosthesis constructs with ≥500 procedures at risk. Seventy-four prosthesis constructs were classified as inferior to the reference by at least 20% relative risk of failure. Thirty-nine of the 74 were shown to be inferior by at least 100% relative risk (figure 3). No prosthesis constructs could be described as non-inferior.

**Figure 4:**
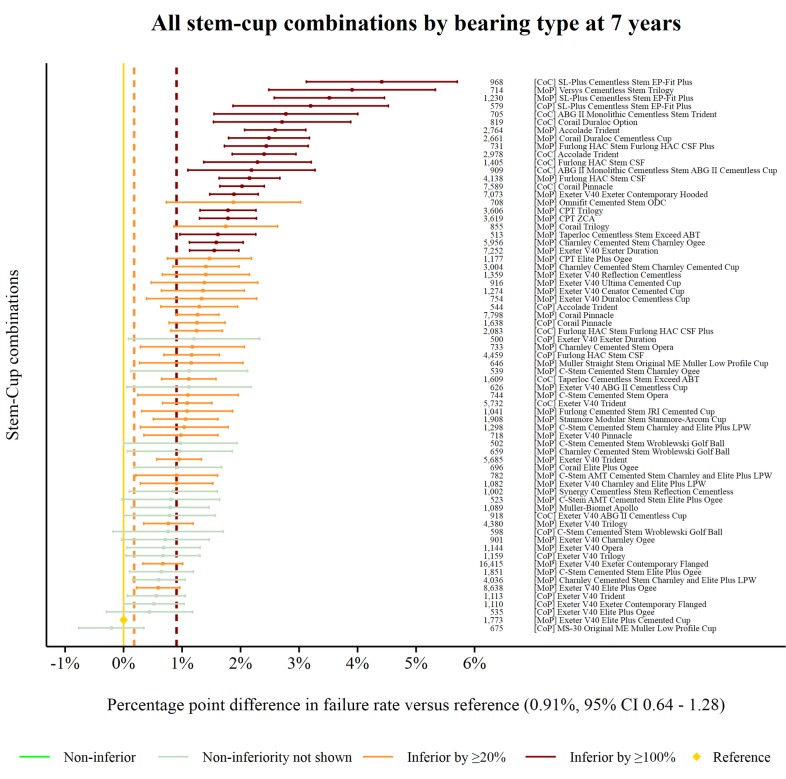
Difference in failure of implanted constructs compared with a contemporary reference at 7 years, using all stem–cup combinations with ≥500 procedures remaining at risk. CoC, ceramic-on-ceramic; CoP, ceramic-on-polyethylene; MoP, metal-on-polyethylene.

The reference prosthesis constructs at 7 years was identified as the MoP Exeter V40/Elite Plus Cemented Cup. There were 1173 procedures remaining at risk and the failure rate was 0.91% (95% CI 0.64 to 1.28). There were 69 prosthesis constructs with ≥500 procedures at risk. Forty-eight prosthesis constructs were classified as inferior to the reference by at least 20% relative risk of failure. Twenty of the 48 were shown to be inferior by at least 100% relative risk (figure 4). No prosthesis constructs could be described as non-inferior.

**Figure 5:**
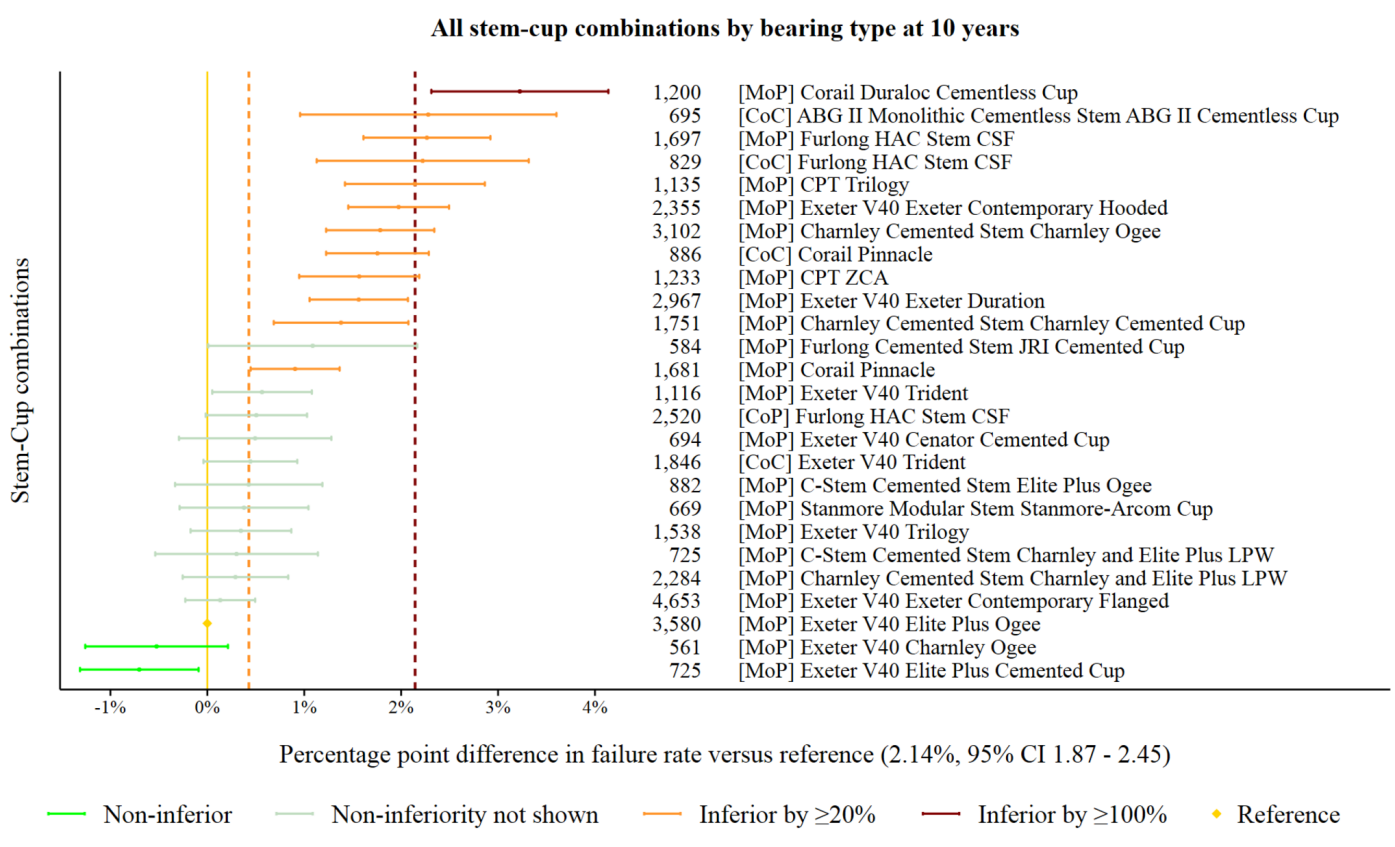
Difference in failure of implanted constructs compared with a contemporary reference at 10 years, using all stem–cup combinations with ≥500 procedures remaining at risk. CoC, ceramic-on-ceramic; CoP, ceramic-on-polyethylene; MoP, metal-on-polyethylene.

The reference prosthesis constructs at 10 years was identified as the MoP Exeter V40/Elite Plus Ogee. There were 3580 procedures remaining at risk and the failure rate was 2.14% (95% CI 1.87 to 2.45). There were 26 prosthesis constructs with ≥500 procedures at risk. Twelve prosthesis constructs were classified as inferior to the reference by at least 20% relative risk of failure. One of the 12 was shown to be inferior by at least 100% relative risk (figure 5). Two prosthesis constructs were identified as non-inferior.

### Non-inferiority: gender specific

Gender-specific non-inferiority analyses were also performed at 3, 5, 7 and 10 years after the primary operation.

At 3 years, only a small number of prosthesis constructs demonstrated non-inferiority in comparison to the reference. Most striking is the large variability of prosthesis constructs used in females compared with males (58 different prosthesis constructs were used >500 times in males vs 93 in females), and the gender-specific heterogeneity in performance. For example, the CoP Exeter V40/Exeter Contemporary Flanged is used as the reference at 3 years in males, yet is inferior by 20% compared with the reference in females at 3 years. A performance difference was also noted in the CoC SL-Plus cementless Stem/EP-Fit Plus between the genders. At 3 years, the failure rate for this prosthesis constructs in all females was 1.75% yet in males after the same period the failure was 5.11% (p<0.001).

At 5 years, the reference failure rate in females is less than half that in males. While there are only 3 prosthesis constructs marked as 100% worse than the reference prosthesis construct in males, there are 24 prosthesis constructs that are 100% worse than the reference in females. Some prosthesis constructs have been used in large numbers despite having relatively poor performance.

At 7 years, the reference failure rate in females remained less than half that of males. There were no prosthesis constructs, used in sufficient numbers, which could be described as non-inferior to the reference in both males and females. One prosthesis construct in males was at least 100% worse than the reference, while 14 prosthesis constructs were at least 100% worse in females.

At 10 years, no prosthesis constructs were described as non-inferior to the reference in both males and females and there were no implants inferior by 100% in either males or females.

### Non-inferiority: gender and age specific

Subdividing procedures by age and gender highlights the paucity of information available pertaining to either male or female patients <55 years undergoing THR. Similarly, the volume of longer-term outcomes on patients beyond 7 years is relatively low in comparison to the number of implanted prosthesis constructs. Most strikingly is the preference for hard-on-hard bearing surfaces (such as CoC) in younger male patients (<55 years). Five of the six prosthesis constructs with at least 500 procedures at 3 years were CoC, contrasted with the vast majority of prosthesis constructs used in older male patients (≥55 years) where either MoP or CoP bearing couples were used. In addition, changes to the distribution of failure rates of prostheses become increasingly apparent. For example, the reference prosthesis construct in men aged <55 years at 3 years has a cumulative failure of 1.26%, whereas the failure rate of the reference in men aged over 75 years at 3 years has a cumulative failure of 0.78%. This is a 60% increase in relative failure rate of the reference procedure for younger males compared with the reference procedure for older males.

The paucity of constructs in each age/gender group which have been utilised over 500 times and for which non-inferiority to the reference prosthesis construct is demonstrated is notable. For men aged <55 years, only four prosthesis constructs (including the reference construct) meet this requirement at 3 years, three prosthesis constructs at 5 years and none thereafter. For women of the same age, the numbers are 4 at 3 years, 3 at 5 years and 7 at 7 years, with none at 10 years. In the largest grouping, those aged between 55 and 75 years, for men, 16 prosthesis constructs meet this requirement at 5 years and only 7 at 10 years, while for women the numbers are 12 at 5 years and 9 at 10 years.

### Sensitivity analysis

We conducted a sensitivity analysis which used the reference prosthesis construct at 10 years as the reference at 3, 5 and 7 years. We assume that the failure trajectory of a non-inferior construct will have the same or lower failure rate compared with the reference construct at 3, 5, 7 and 10 years. This approach is conservative, as it preserves the status quo with respects implant performance. The reference construct in all procedures at 10 years is the Exeter V40/Elite Plus Ogee with a failure rate of 2.14% (95% CI 1.87 to 2.45). Only one prosthesis construct is non-inferior and statistically superior, that is, the Exeter V40/Elite Plus Cemented cup, but it does not have 1000 implants at risk at 10 years and therefore is not considered to be the reference construct. At 7, 5 and 3 years, the contemporary reference has a 0.59%, a 0.55% and 0.40% lower failure rate than the historical reference, respectively. While the good performance of many prosthesis constructs appear to track, some exhibit substantially variability in their relative performance at the times of interest. Specifically, the MoP Exeter V40/Charnley Ogee is non-inferior to the historical reference at 10 years, but is inferior by 20% at 5 years.

## Discussion

We have demonstrated in 797 178 primary THRs the relative performance of implanted prosthesis constructs in comparison to an internally selected contemporary reference. There is substantial variation in the performance of prosthesis constructs. A non-inferiority approach to benchmarking provides an immediate comparison of commonly used implanted prosthesis constructs compared with an internal contemporary reference and conveys distinct advantages opposed to standard Kaplan-Meier analyses as currently reported in the NJR annual reports or categorical grades provided by ODEP. The heterogeneity in implanted constructs in females compared with males is aptly illustrated, as is the paucity of information in clinically relevant substrata. The marked differences in outcomes between the different age/gender substrata confirm the importance of comparing prosthesis constructs within these strata.^13^ We present this study as a novel way of assessing hip prosthesis constructs and as such there is, in the authors opinion, no relevant evidence published to date.

What is most striking is that so few prosthesis constructs in each age/gender strata meet the criteria of 500 cases at each time point and are at least classified as ‘inferiority not shown’. Of the 4442 constructs used, only seven meet these criteria in men aged 55–75 years (online supplementary figure 5b) and nine in women aged 55–75 years at 10 years (online supplementary figure 9b). None meet the criteria in any other age/gender substrata at 10 years. Even at the relatively short follow-up of 5 years, only 16 constructs in men (online supplementary figure 3c) and 12 in women aged 55–75 years (online supplementary figure 7c) meet these criteria. Patients would have a reasonable expectation that the implants they receive have a proven track record and have not been demonstrated as having a 20% or more increased revision rate for patients of the same age and gender. It is important to note that some prosthesis constructs have a higher early relative failure rate and a lower relative failure rate in later years and thus are inferior at 3, 5 and 7 years, but inferiority is not shown at 10 years. Examples of this in men aged 55–75 years are the MoP Corail/Pinnacle and MoP Exeter/Contemporary hooded, we believe this effect is principally driven by the lower failure of the reference at earlier timepoints, that is, a reduction in revision rate with a later cohort. Late failure is preferential to early failure from the patient, societal and health economic perspectives, particularly as early revision is unfortunately associated with a high rate of rerevision.^18^

One of the most obvious trends across all stratifications is the outstanding performance of the Exeter V40 stem as part of various prosthesis constructs. However, the heterogeneity in acetabular prostheses paired with the Exeter V40 stem is substantial, as is the subsequent variation in performance. This aptly illustrates the need to benchmark constructs opposed to individual implants which make up prosthesis constructs, which has the potential to provide false reassurance in terms of efficacy as the individual elements of a construct are not independent. Patient-specific construct selection is another strong feature of the data, with the majority of younger patient receiving CoC bearing surfaces, whereas the majority of older patients receive MoP bearing surfaces.

This analysis does show that certain constructs are either the reference or are non-inferior to the reference prosthesis construct across almost all age and gender strata. This strongly suggests that they could appropriately be used as default options for the majority of patients. This is particularly relevant for inexperienced surgical teams, as they can focus training on, and become expert with, a single prosthesis construct. This has the potential to reduce the risk of technical error, to be cost saving through bulk purchasing arrangements and via a reduction in failure rates. The absolute level of failure of commonly used constructs is relatively low, and <5% in many instances. This apparently excellent (ODEP 10A*) performance is exhibited by nearly all prosthesis constructs with sufficient data at 10 years (≥500 patients at risk) and raises questions about whether an externally placed benchmark is the optimal way to ensure best care.

Encouragingly, the sensitivity analysis demonstrates the prognosis for patients undergoing hip replacement is continually improving, and the currently best performing implant at 10 years is unlikely to be as good as the contemporary references at 3, 5 and 7 years when these reach 10 years of follow-up. While the refinement of clinical practice and development of prosthesis constructs appears to be raising the bar in performance, it is clear that these improvements are not universal. This raises questions about how implants are introduced into a market safely; ensuring enough prosthesis constructs are implanted to ascertain their relative performance, but no more than the necessary number of prosthesis constructs are implanted to minimise the exposure of patients to poor performing implants, and finally to ensure there is sufficient incentive for implant manufacturers to develop new prostheses that benefit patients.

This analysis has a number of important strengths. We explicitly compare prosthesis constructs to a contemporary reference, and a historical defined reference with known performance using a non-inferiority study design. The unambiguous presentation of data allows surgeons, patients and policy makers to directly compare commonly used prosthesis constructs to a reference construct. We illustrate the paucity of information in clinically relevant substrata and the need to compare implant constructs opposed to implant elements. The analysis has a number of limitations; case-mix adjustment by stratification is difficult to assimilate despite the restricted set of confounding factors. Residual and unmeasured confounding factors are likely to be present, and the ability to interpret analyses from a causal perspective is limited. It is also known that revision rate is influenced by factors such as the primary indication. In this study, there is a breadth of indications for the primary procedure; however, 89.1% (709 902) of procedures had osteoarthritis listed as the only reason for primary whereas fractured neck of femur was the reason for primary in 23 336 (2.9%) cases. The NJR annual report illustrates that neck of femur fracture is associated with a small increase in risk of revision in the first 6 months, but then revision rate is approximately equal to other patients.^3^ The number of patients remaining at risk after extended follow-up is low, and therefore the power to detect non-inferiority assuming there is truly no difference in prosthesis constructs compared with the reference is also low. This phenomenon is further compounded when we compare stratum-specific performance of constructs at nearly all time points. Whilst reducing the number of procedures required to be at risk may be appealing, to increase the number of comparisons, the precision of those comparisons would be low, and therefore not informative. It is also possible that the constructs listed in this analysis may use a mixture of liner materials (ie, cross-linked polyethylene [XLPE] and non-XLPE) which may lead to variation of revision rates within constructs. Data entry is mandated and data capture is extremely high (over 95%),^15^ thus the findings in this study are highly likely to be generalisable. Furthermore, the reasons for failing to record those remaining primaries or revisions in the NJR is unlikely to related to the choice of implant. Therefore, these data would be classified as missing completely at random and would be unlikely to bias our results.^19^

### Conclusions, policy and future research implications

The use of product benchmarking has the potential to be highly informative for patients, change the practice of surgeons and influence policy makers if presented clearly and unambiguously. Clinical implications of this research are far reaching. We are unable to definitively state which construct is the best choice for all patients, due to the presence of selection effects and residual confounding. However, we believe that the information presented here illustrates the variability, frequency and performance of different constructs currently used in clinical practice. This in turn, should be used to further inform the consenting process between the patient and the surgeon, and facilitate implant selection. We believe commissioners and policy makers should consider the variability and performance of different implants before commissioning healthcare providers. Furthermore, qualitative research is required to understand why surgeons select new implants, with limited understanding of long-term performance, in favour of constructs with demonstrably low failure rates, for example, the (MoP) Exeter V40 Elite plus Ogee, (MoP) Exeter V40 Charnley Ogee, (MoP) Exeter V40 Elite plus cemented cup which represented only ~1% of the 91 698 constructs implanted in the NJR in 2017.^3^

## Supplementary Material

Reviewer comments

Author's manuscript
