# Supplementary material for: Assessing the non-inferiority of prosthesis constructs used in hip replacement using data from the National Joint Registry of England, Wales, Northern Ireland and the Isle of Man: a benchmarking study
Source: BMJ Open. 2019 Apr 29;9(4):e026685. doi: 10.1136/bmjopen-2018-026685 (PMC6502009; doi:10.1136/bmjopen-2018-026685)
Supplement: Supplementary data [file bmjopen-2018-026685supp001.pdf]

Supplementary Figure 1: Flow diagram of showing derivation of procedures used in analyses

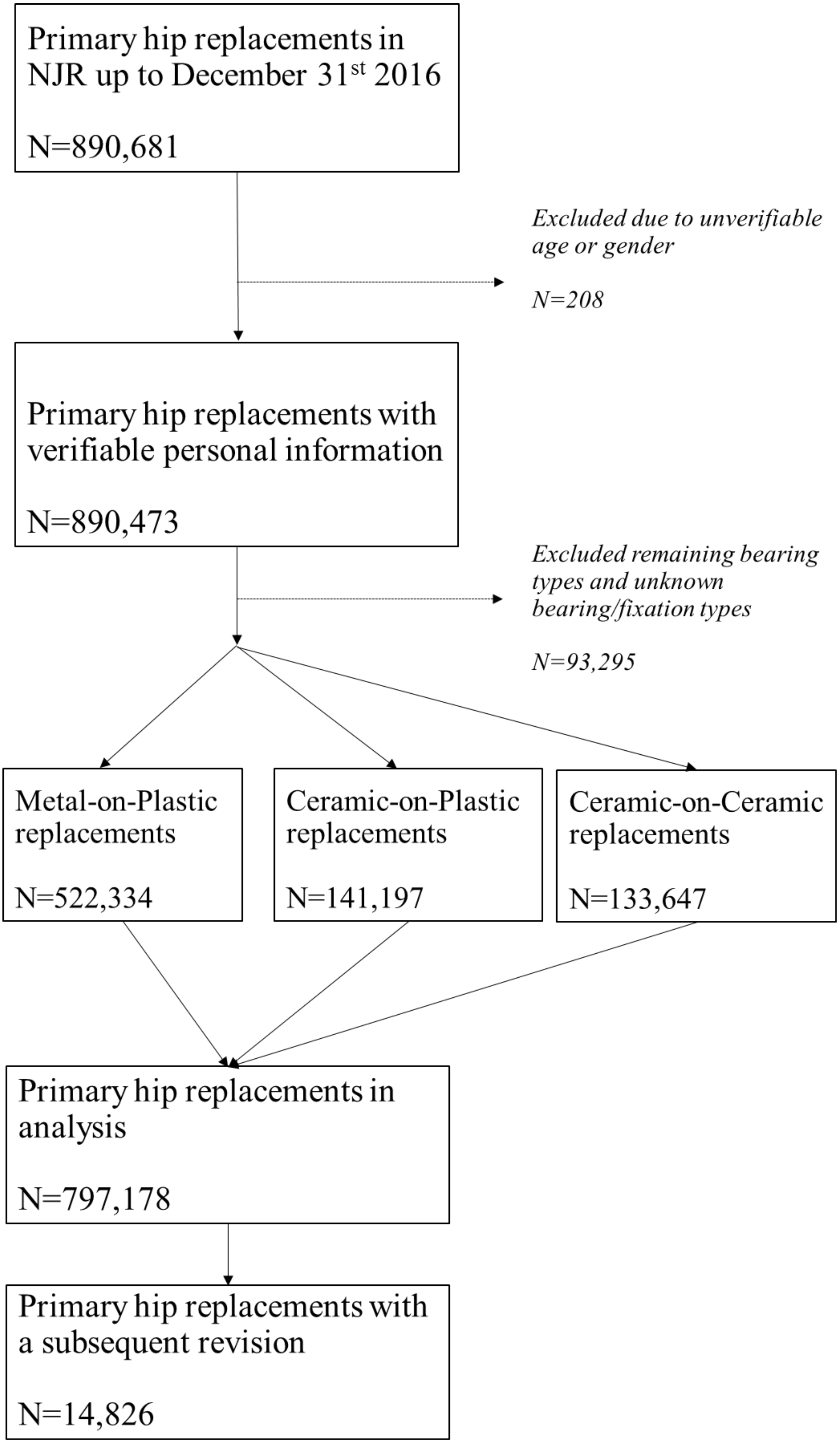

Supplementary Figure 2a: Difference in failure of implanted constructs compared to a contemporary reference at 3 years in men, using all stem-cup combinations with ≥500 procedures remaining at risk

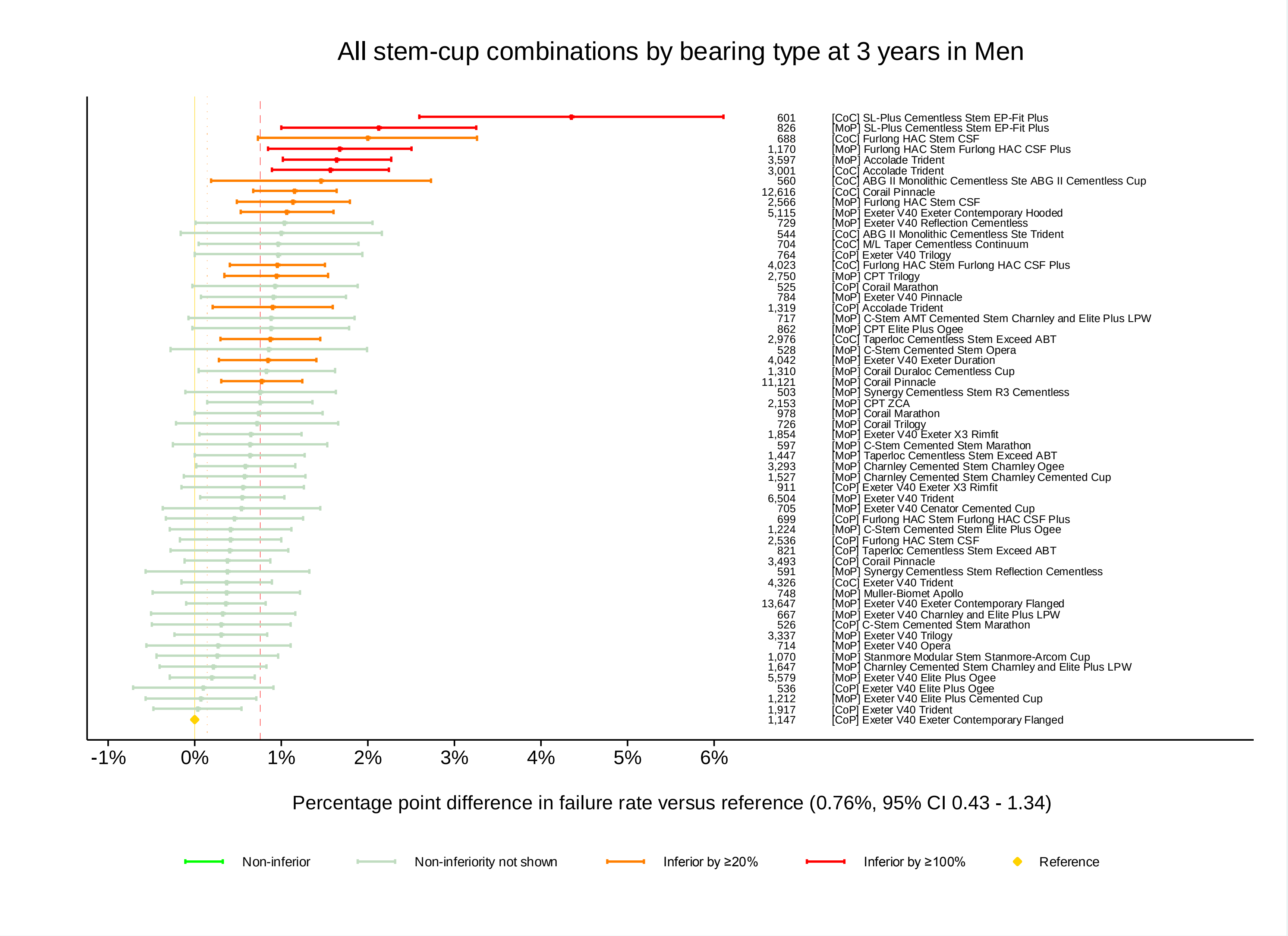

Supplementary Figure 2b: Difference in failure of implanted constructs compared to a contemporary reference at 3 years in men less than 55 years, using all stem-cup combinations with ≥500 procedures remaining at risk

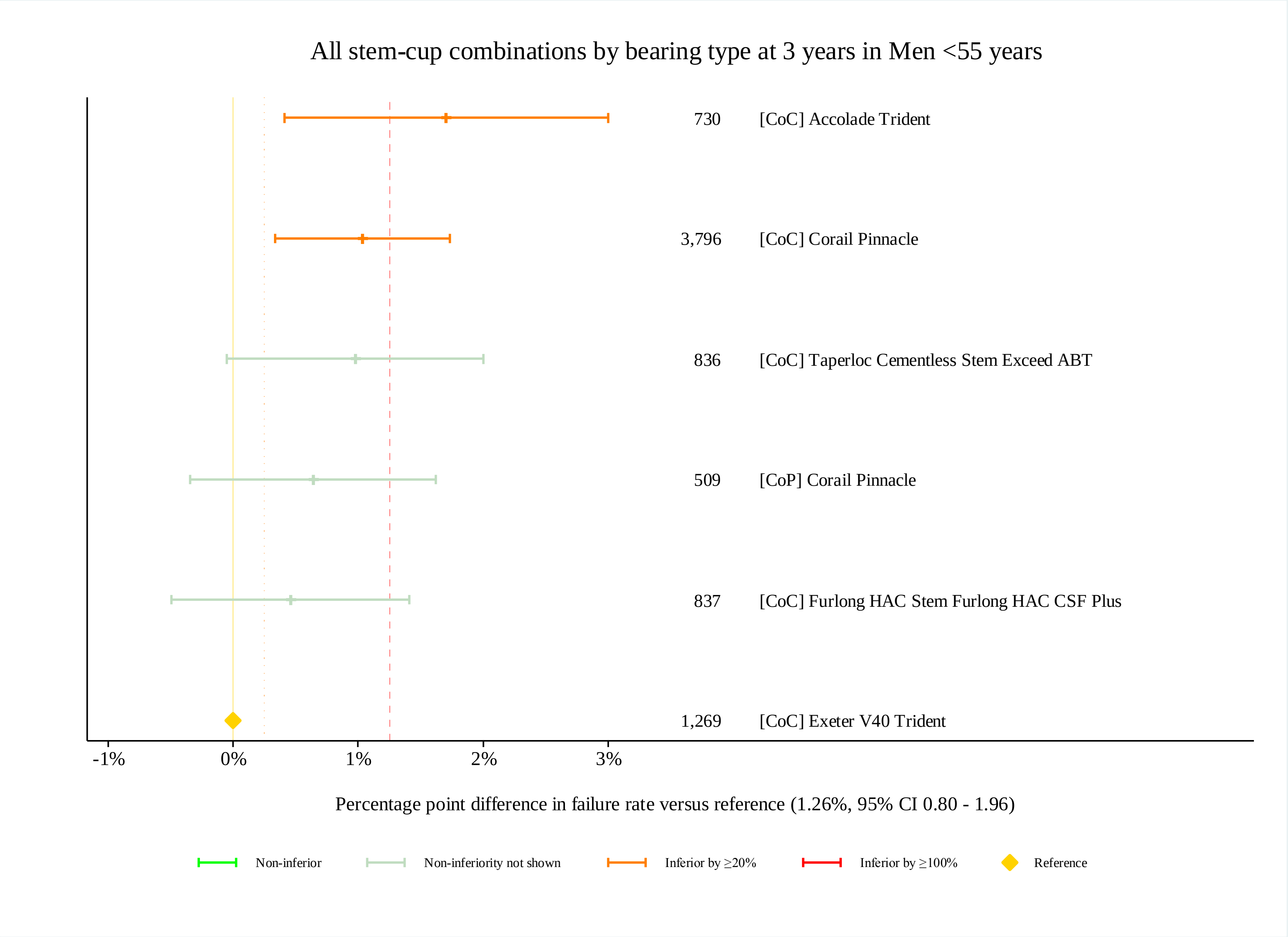

Supplementary Figure 2c: Difference in failure of implanted constructs compared to a contemporary reference at 3 years in men between 55 and 75 years, using all stem-cup combinations with ≥500 procedures remaining at risk

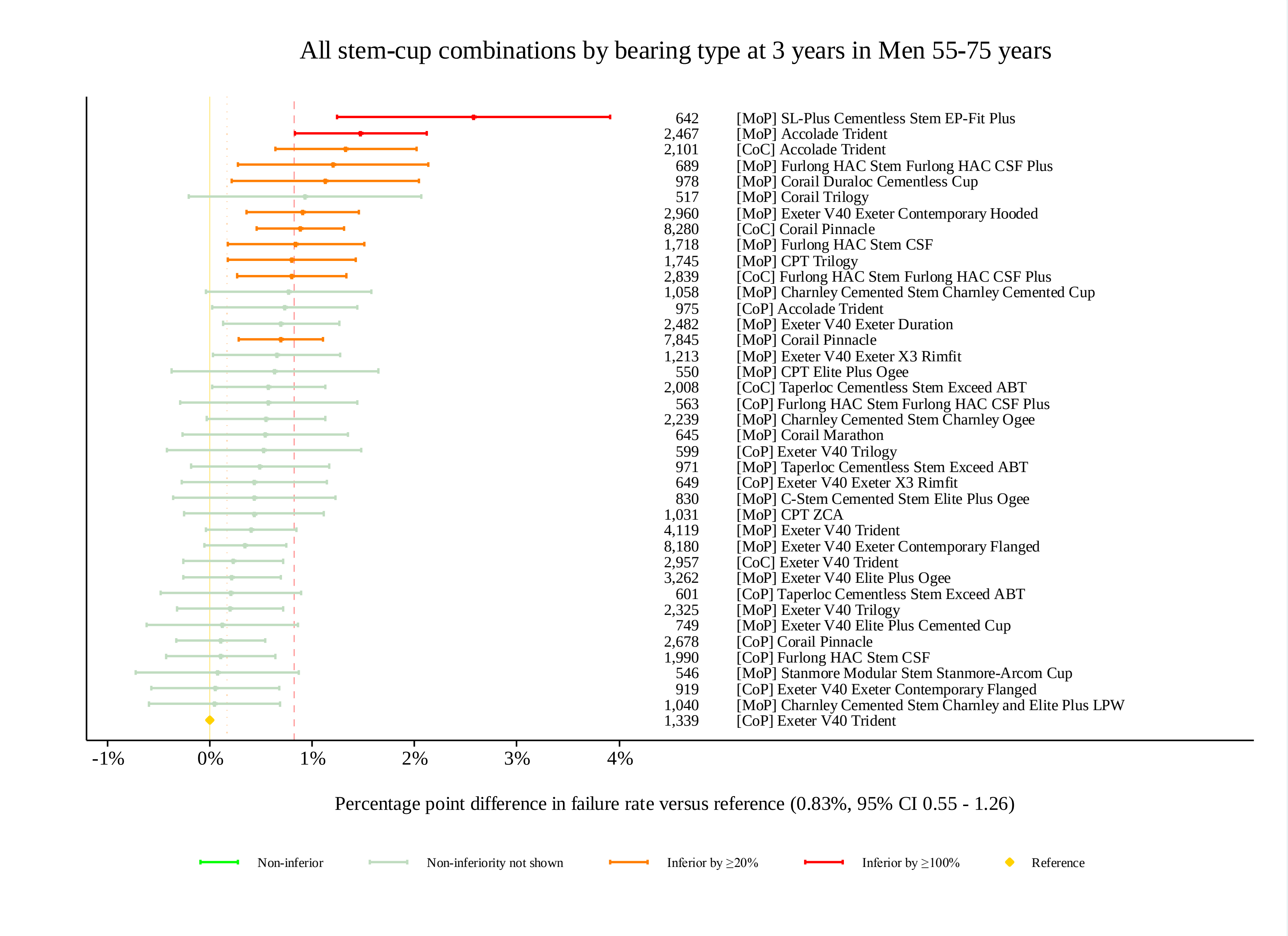

Supplementary Figure 2d: Difference in failure of implanted constructs compared to a contemporary reference at 3 years in men greater than 75 years, using all stem-cup combinations with ≥500 procedures remaining at risk

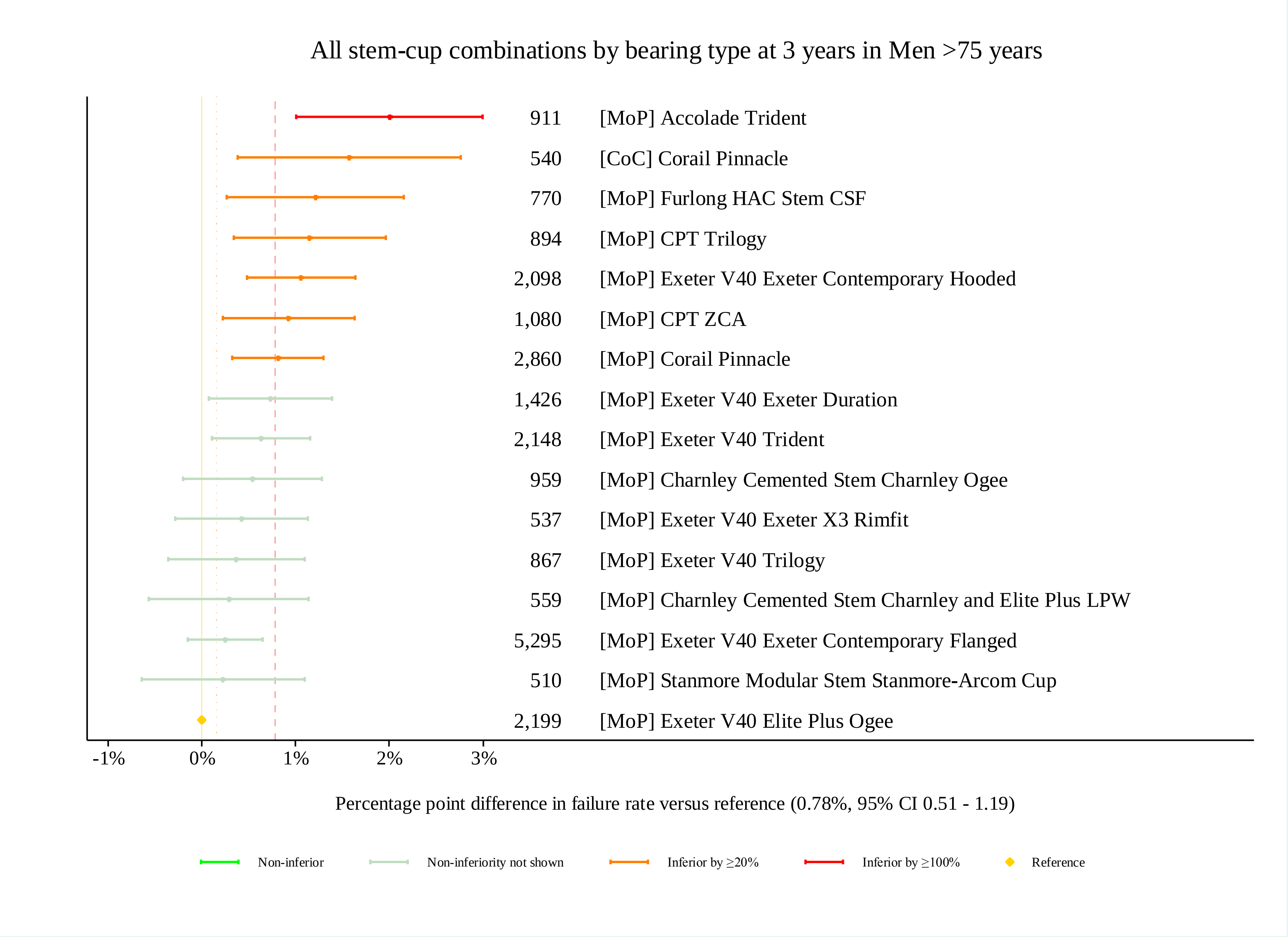

Supplementary Figure 3a: Difference in failure of implanted constructs compared to a contemporary reference at 5 years in men, using all stem-cup combinations with ≥500 procedures remaining at risk

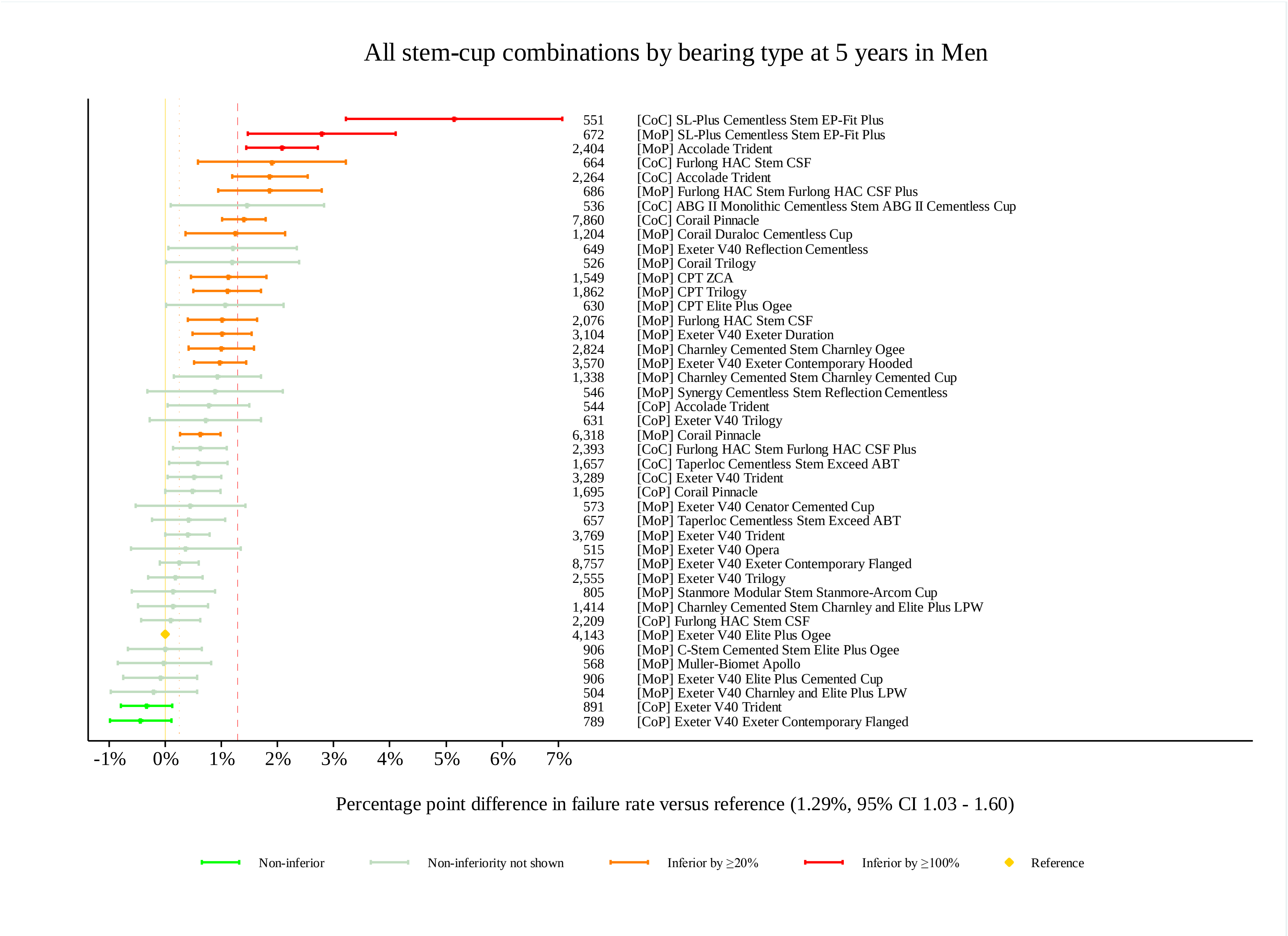

Supplementary Figure 3b: Difference in failure of implanted constructs compared to a contemporary reference at 5 years in men less than 55 years, using all stem-cup combinations with ≥500 procedures remaining at risk

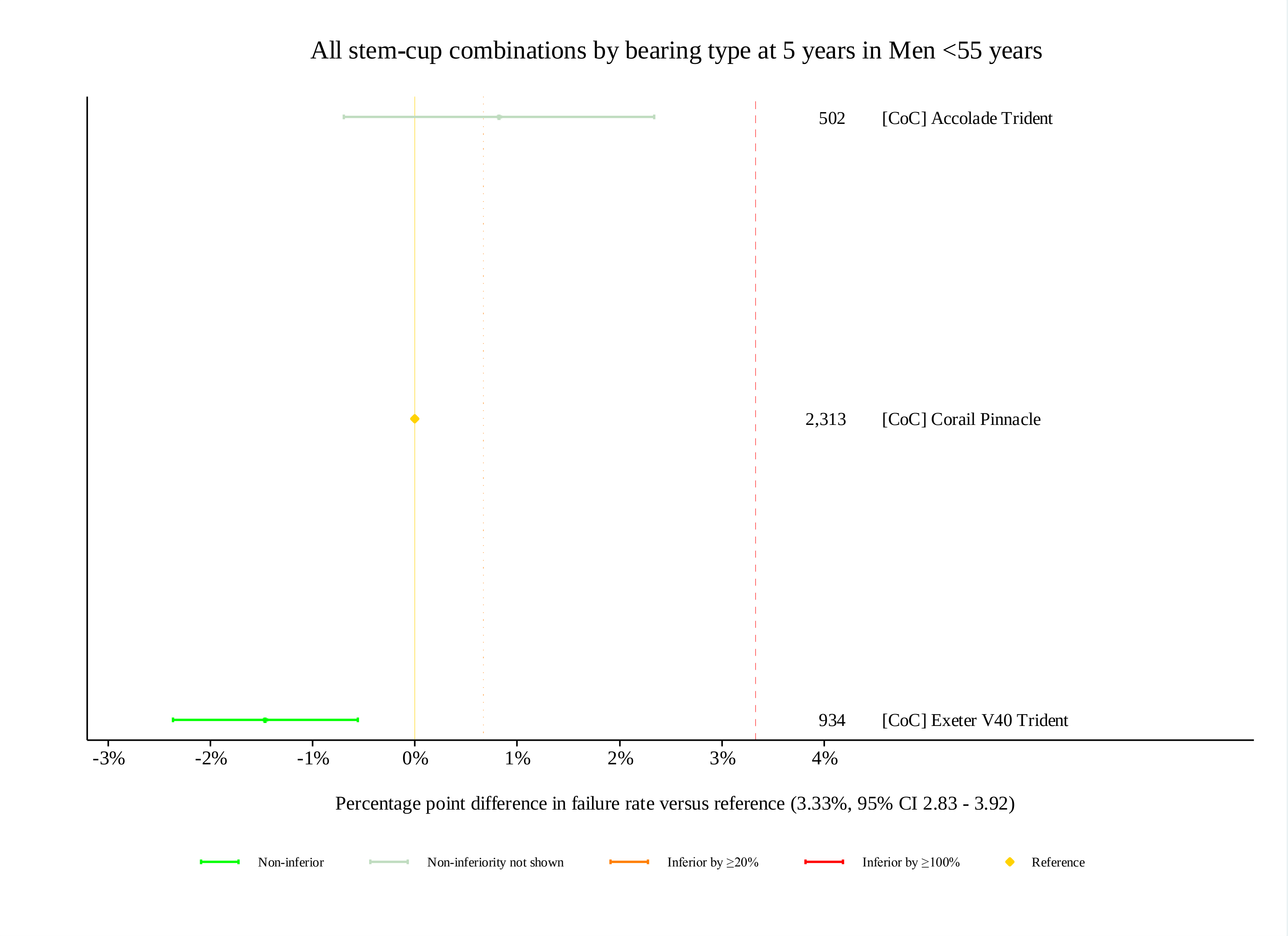

Supplementary Figure 3c: Difference in failure of implanted constructs compared to a contemporary reference at 5 years in men between 55 and 75 years, using all stem-cup combinations with ≥500 procedures remaining at risk

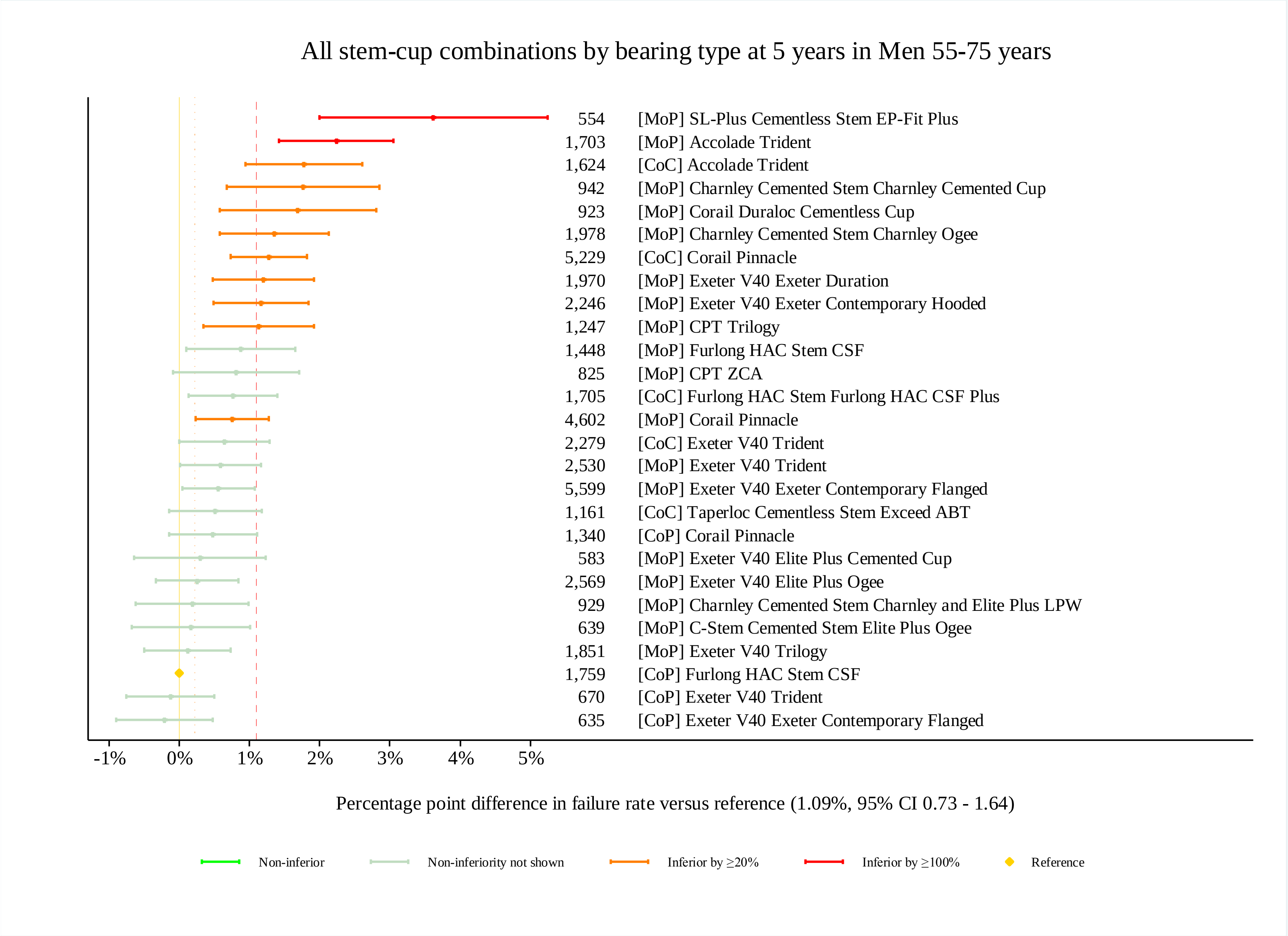

Supplementary Figure 3d: Difference in failure of implanted constructs compared to a contemporary reference at 5 years in men greater than 75 years, using all stem-cup combinations with ≥500 procedures remaining at risk

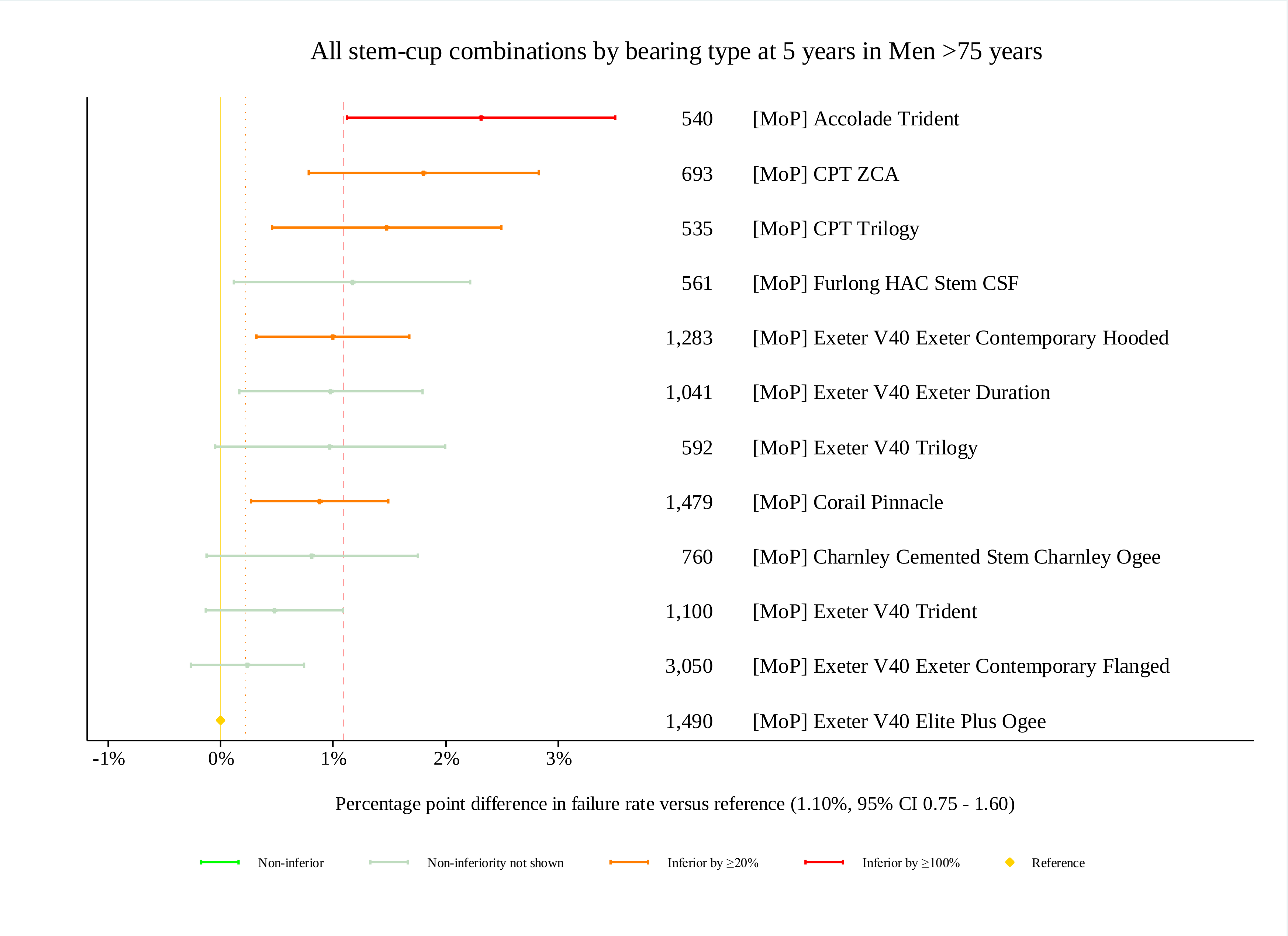

Supplementary Figure 4a: Difference in failure of implanted constructs compared to a contemporary reference at 7 years in men, using all stem-cup combinations with ≥500 procedures remaining at risk

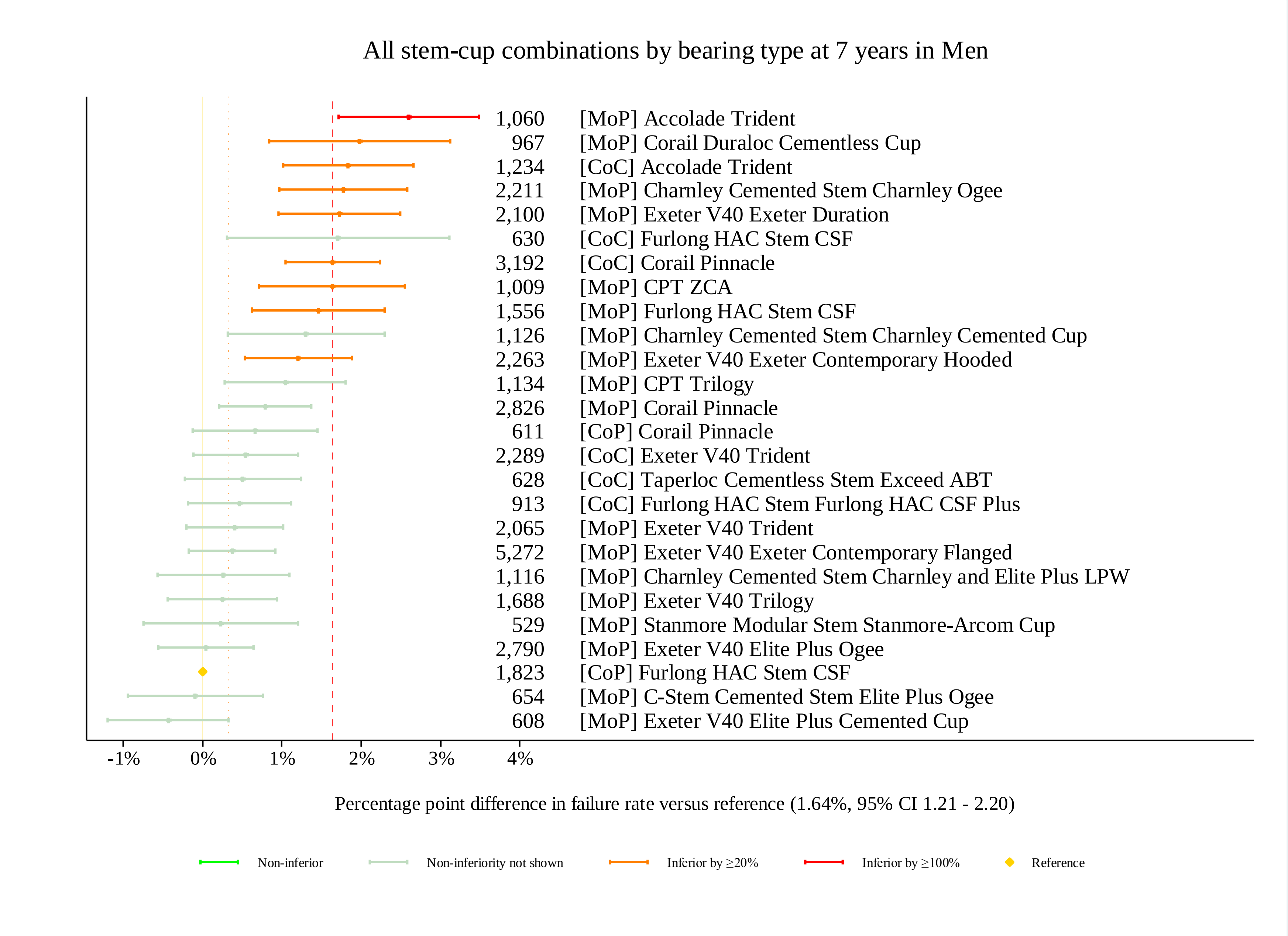

Supplementary Figure 4b: Difference in failure of implanted constructs compared to a contemporary reference at 7 years in men between 55 and 75 years, using all stem-cup combinations with ≥500 procedures remaining at risk

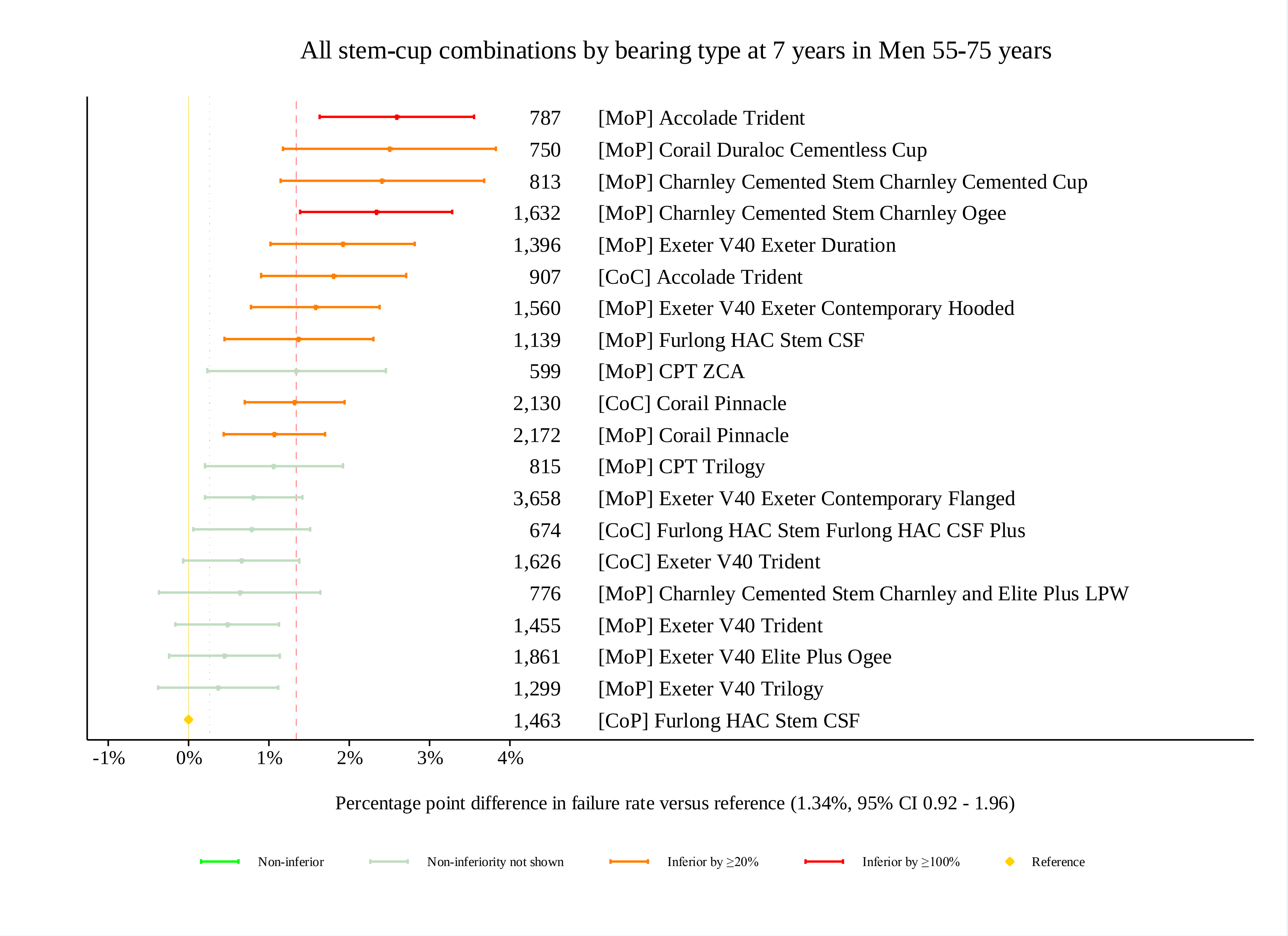

Supplementary Figure 4c: Difference in failure of implanted constructs compared to a contemporary reference at 7 years in men greater than 75 years, using all stem-cup combinations with ≥500 procedures remaining at risk

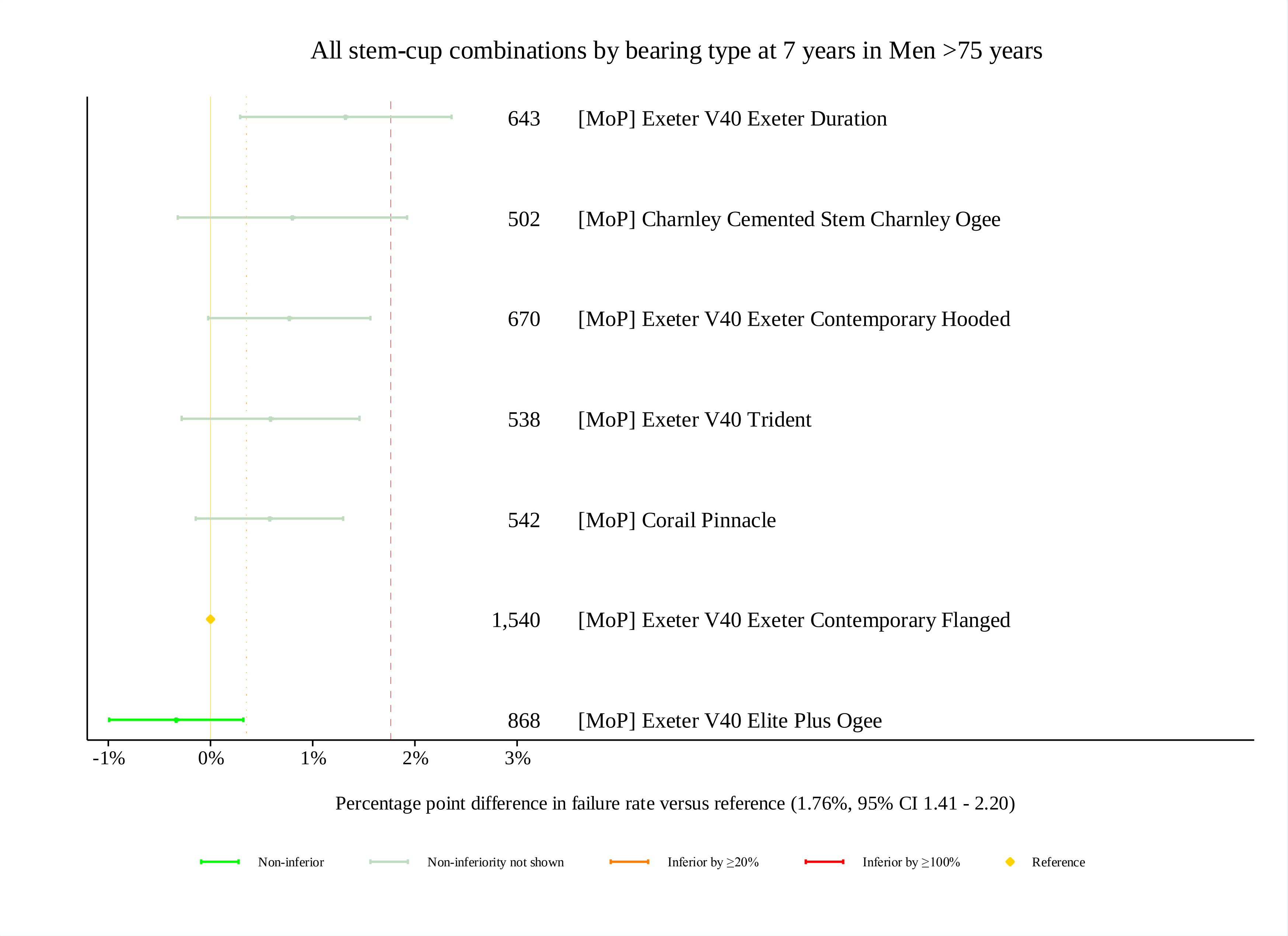

Supplementary Figure 5a: Difference in failure of implanted constructs compared to a contemporary reference at 10 years, using all stem-cup combinations with ≥500 procedures remaining at risk

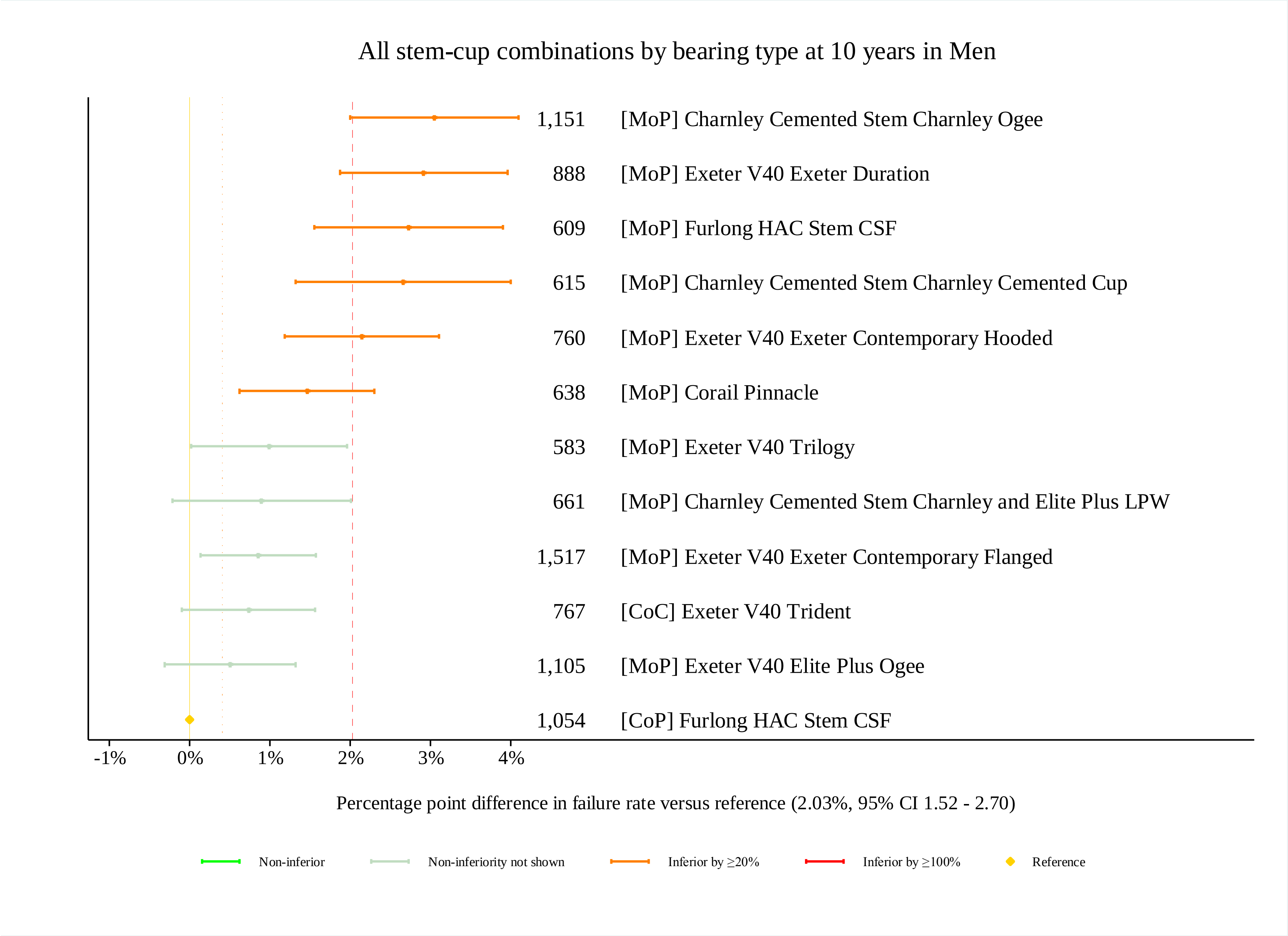

Supplementary Figure 5b: Difference in failure of implanted constructs compared to a contemporary reference at 10 years in men between 55 and 75 years, using all stem-cup combinations with ≥500 procedures remaining at risk

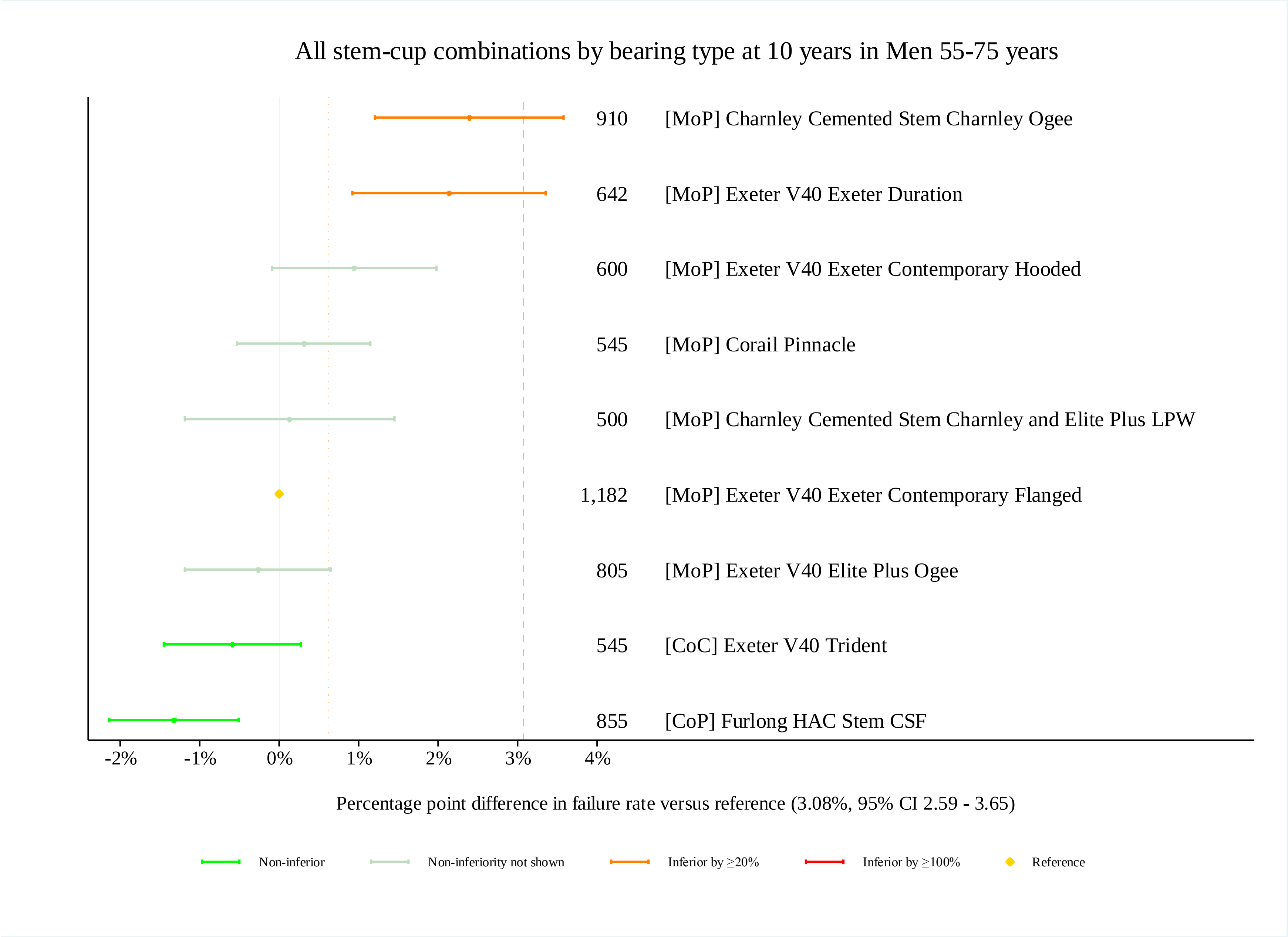

Supplementary Figure 6a: Difference in failure of implanted constructs compared to a contemporary reference at 3 years in women, using all stem-cup combinations with ≥500 procedures remaining at risk

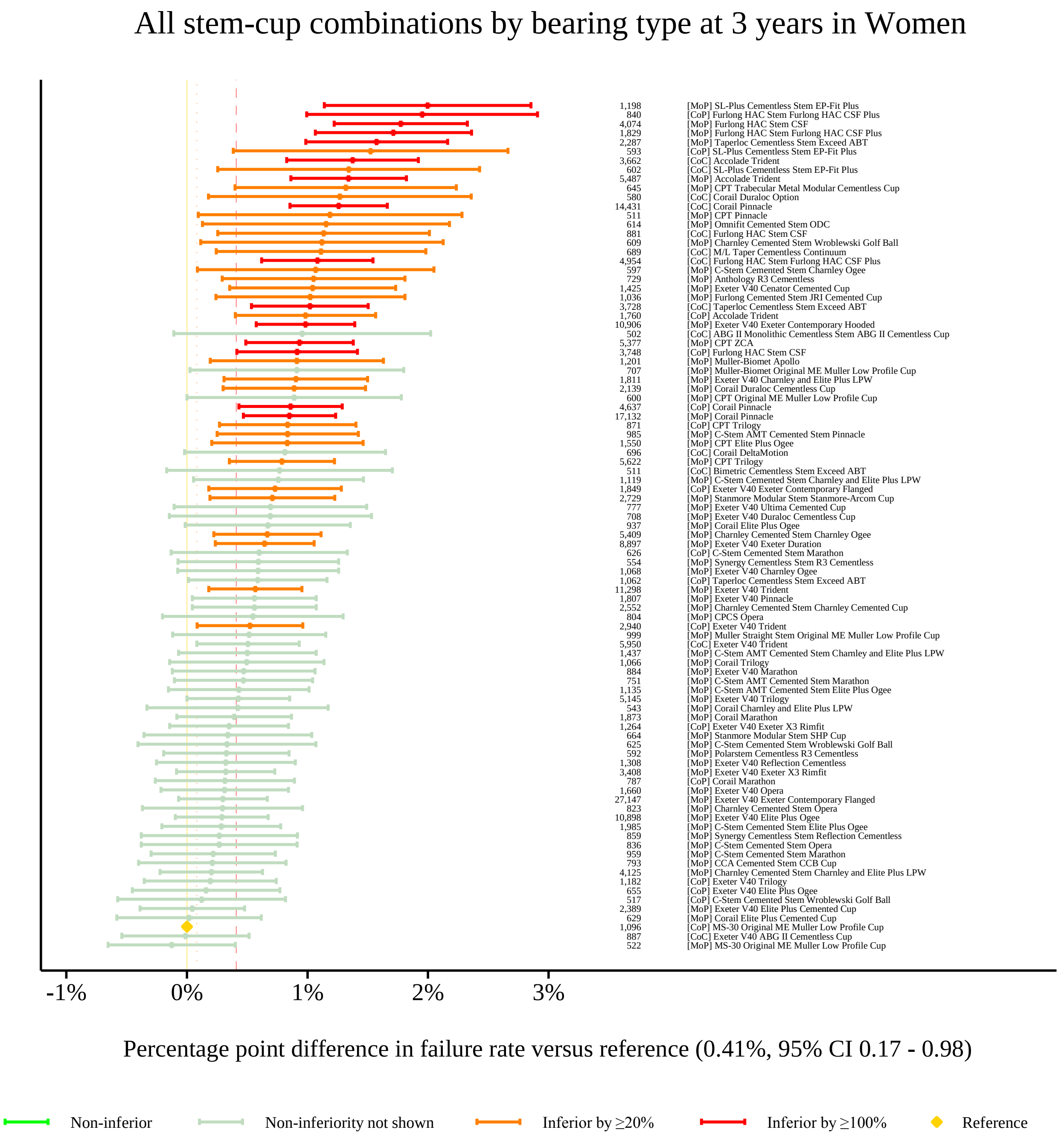

Supplementary Figure 6b: Difference in failure of implanted constructs compared to a contemporary reference at 3 years in women less than 55 years, using all stem-cup combinations with ≥500 procedures remaining at risk

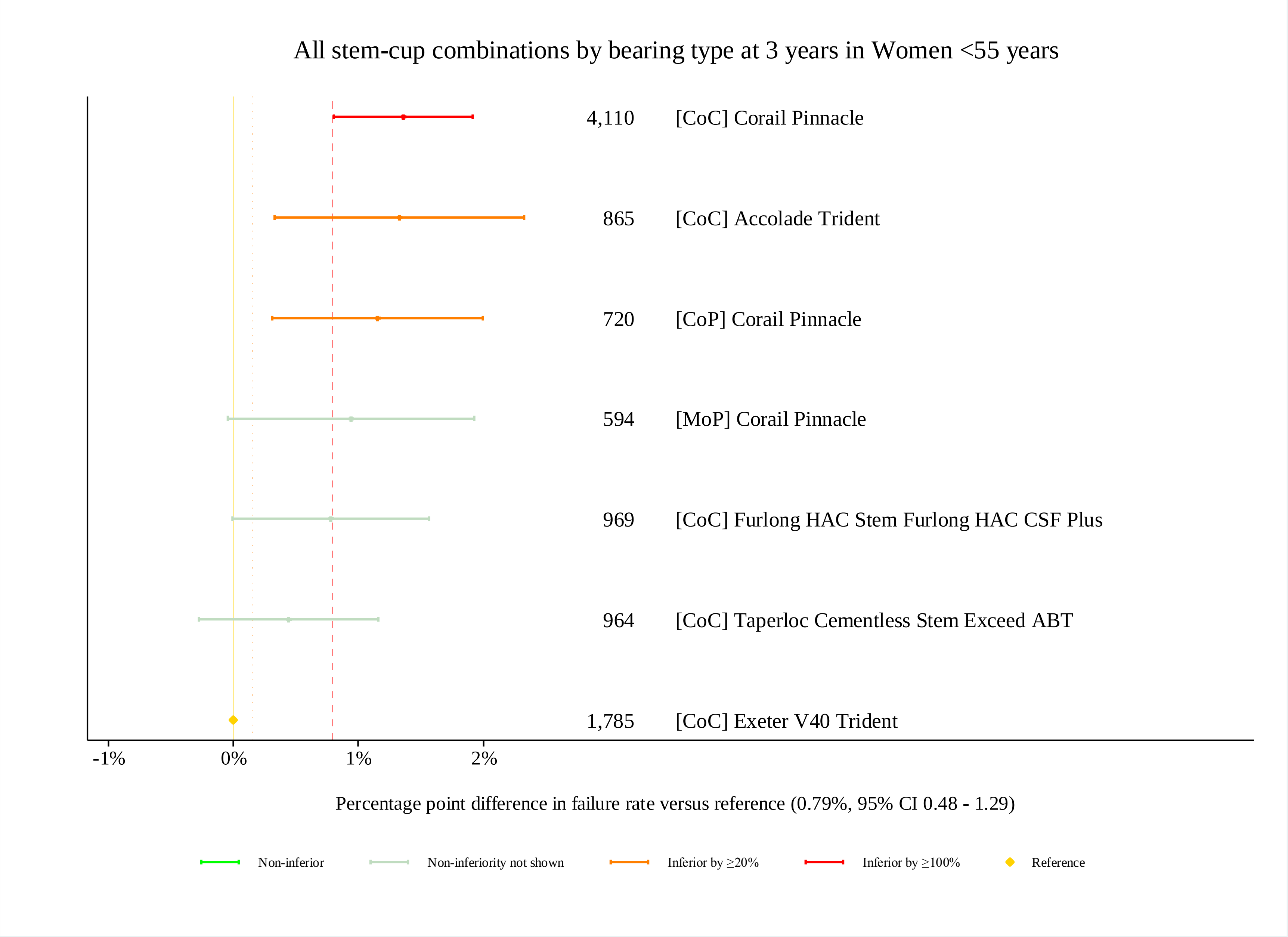

Supplementary Figure 6c: Difference in failure of implanted constructs compared to a contemporary reference at 3 years in women between 55 and 75 years, using all stem-cup combinations with ≥500 procedures remaining at risk

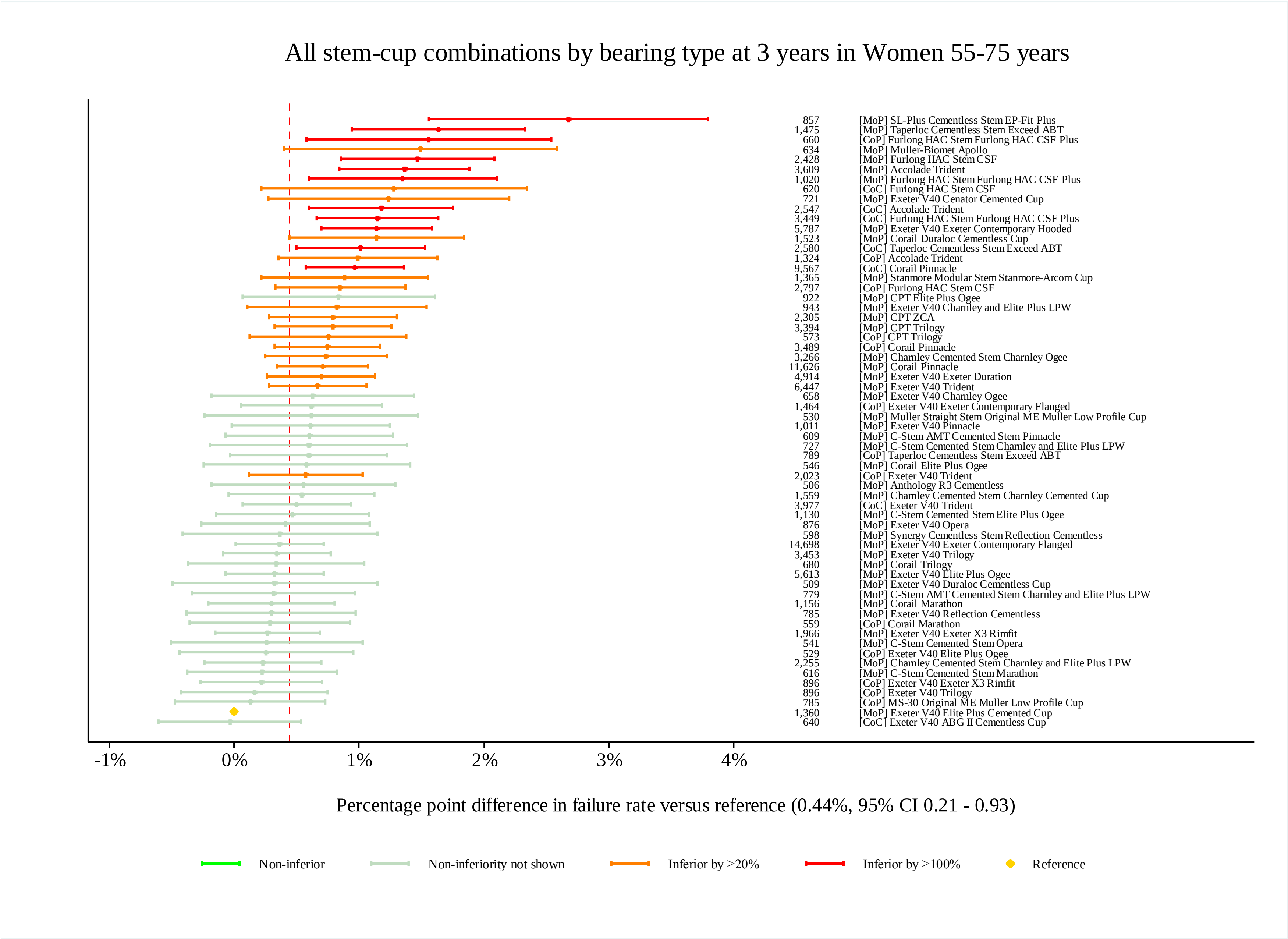

Supplementary Figure 6d: Difference in failure of implanted constructs compared to a contemporary reference at 3 years in women greater than 75 years, using all stem-cup combinations with ≥500 procedures remaining at risk

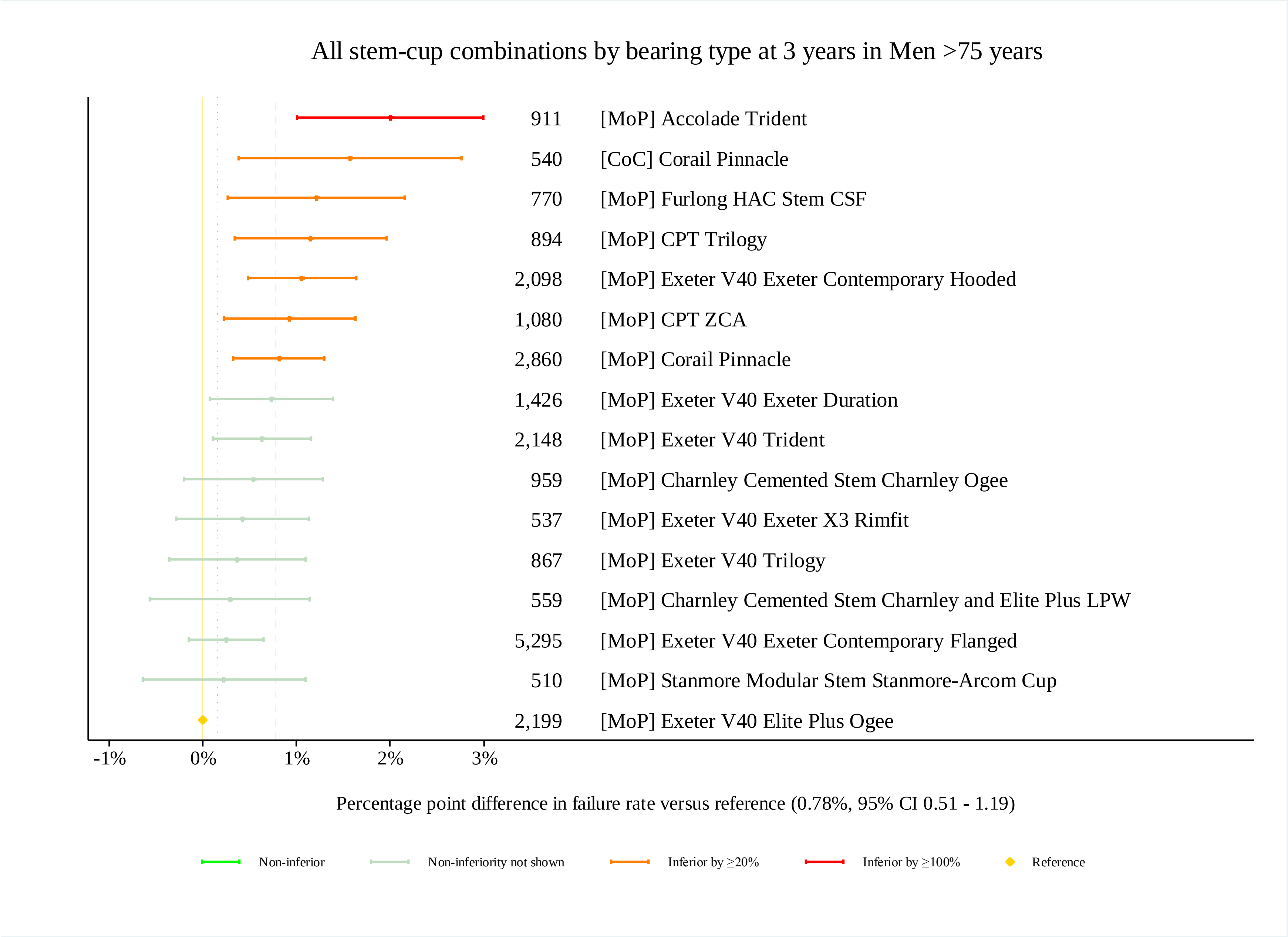

Supplementary Figure 7a: Difference in failure of implanted constructs compared to a contemporary reference at 5 years in women, using all stem-cup combinations with ≥500 procedures remaining at risk

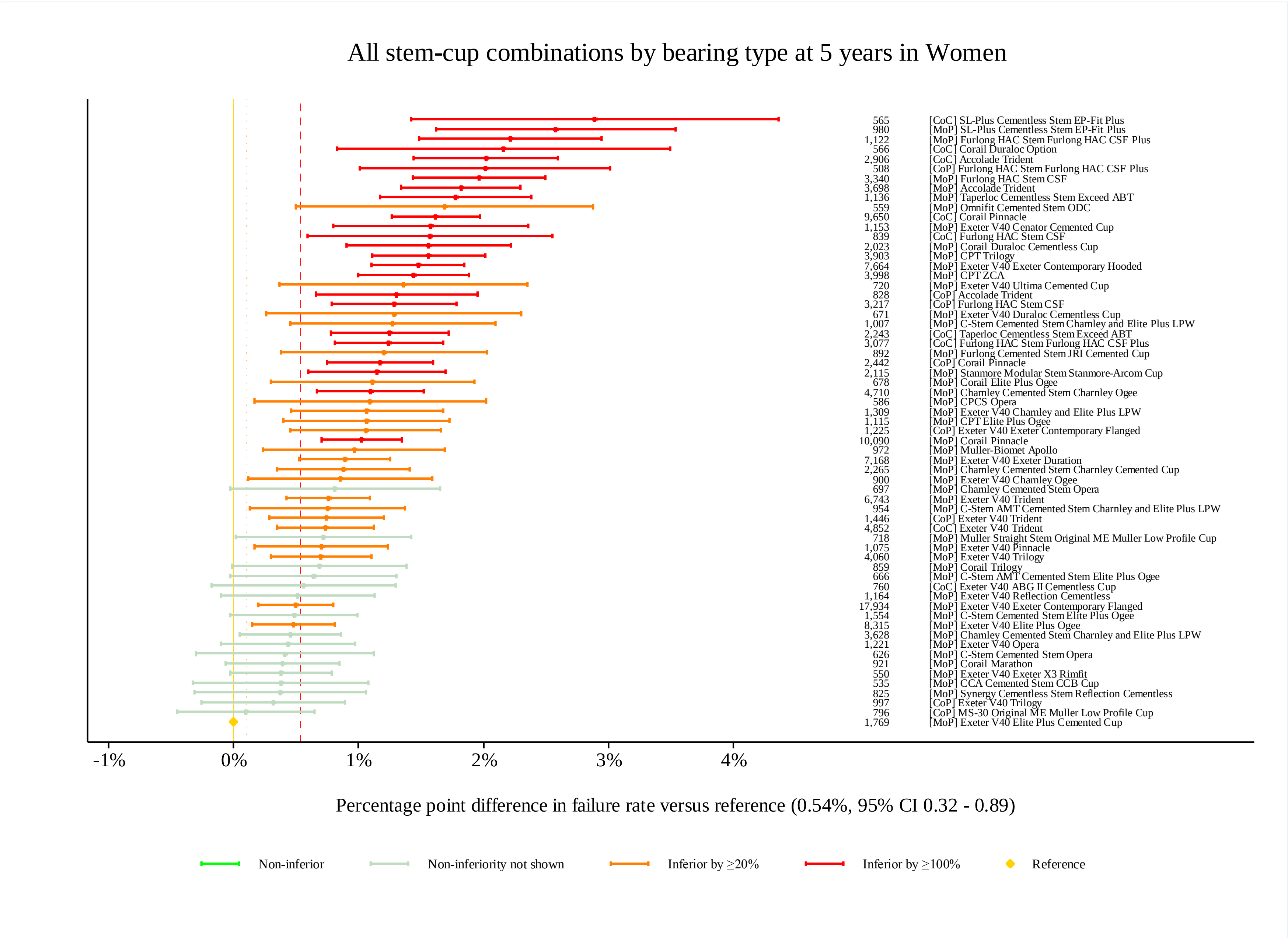

Supplementary Figure 7b: Difference in failure of implanted constructs compared to a contemporary reference at 5 years in women less than 55 years, using all stem-cup combinations with ≥500 procedures remaining at risk

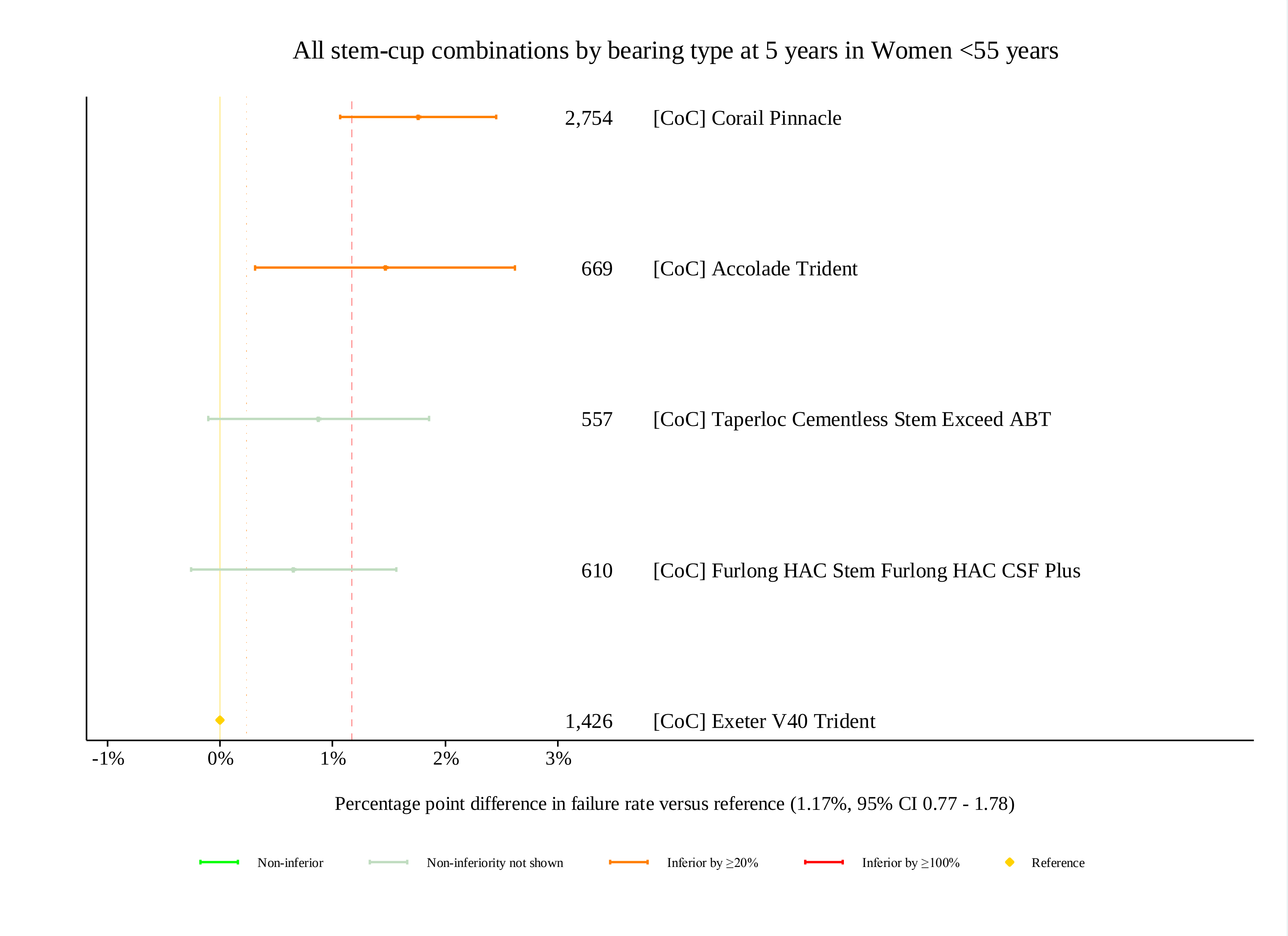

Supplementary Figure 7c: Difference in failure of implanted constructs compared to a contemporary reference at 5 years in women between 55 and 75 years, using all stem-cup combinations with ≥500 procedures remaining at risk

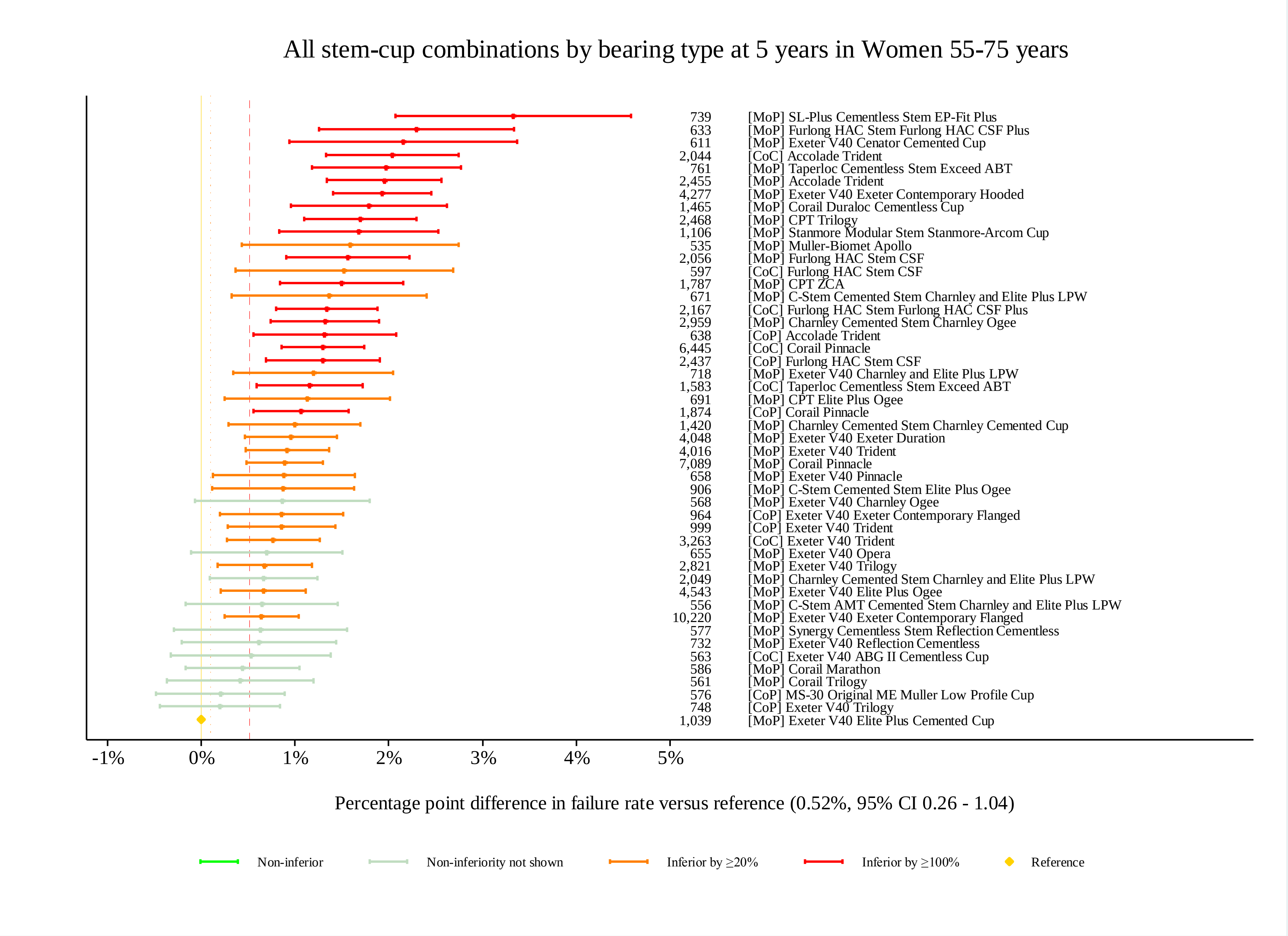

Supplementary Figure 7d: Difference in failure of implanted constructs compared to a contemporary reference at 5 years in women greater than 75 years, using all stem-cup combinations with ≥500 procedures remaining at risk

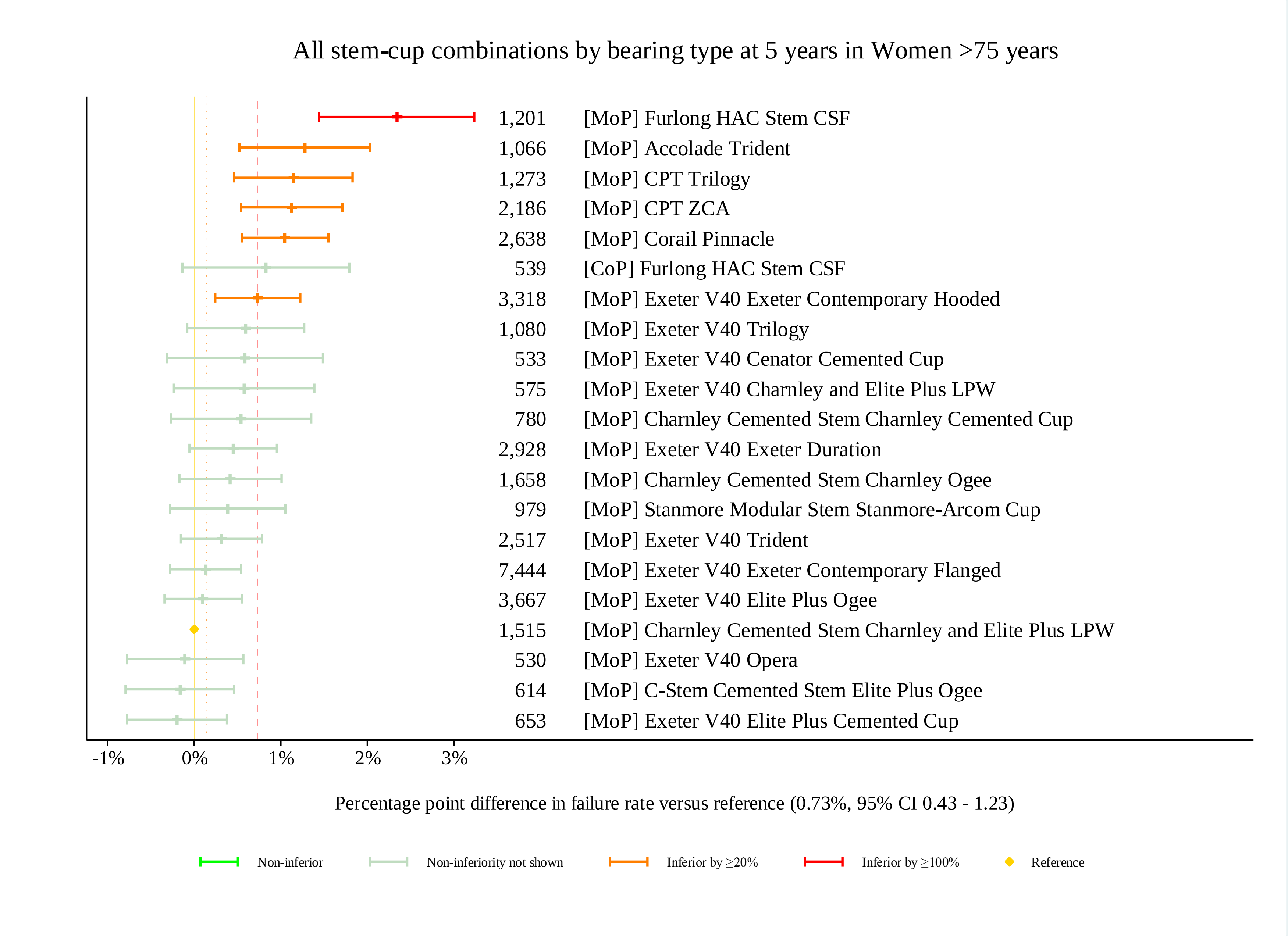

Supplementary Figure 8a: Difference in failure of implanted constructs compared to a contemporary reference at 7 years in women, using all stem-cup combinations with ≥500 procedures remaining at risk

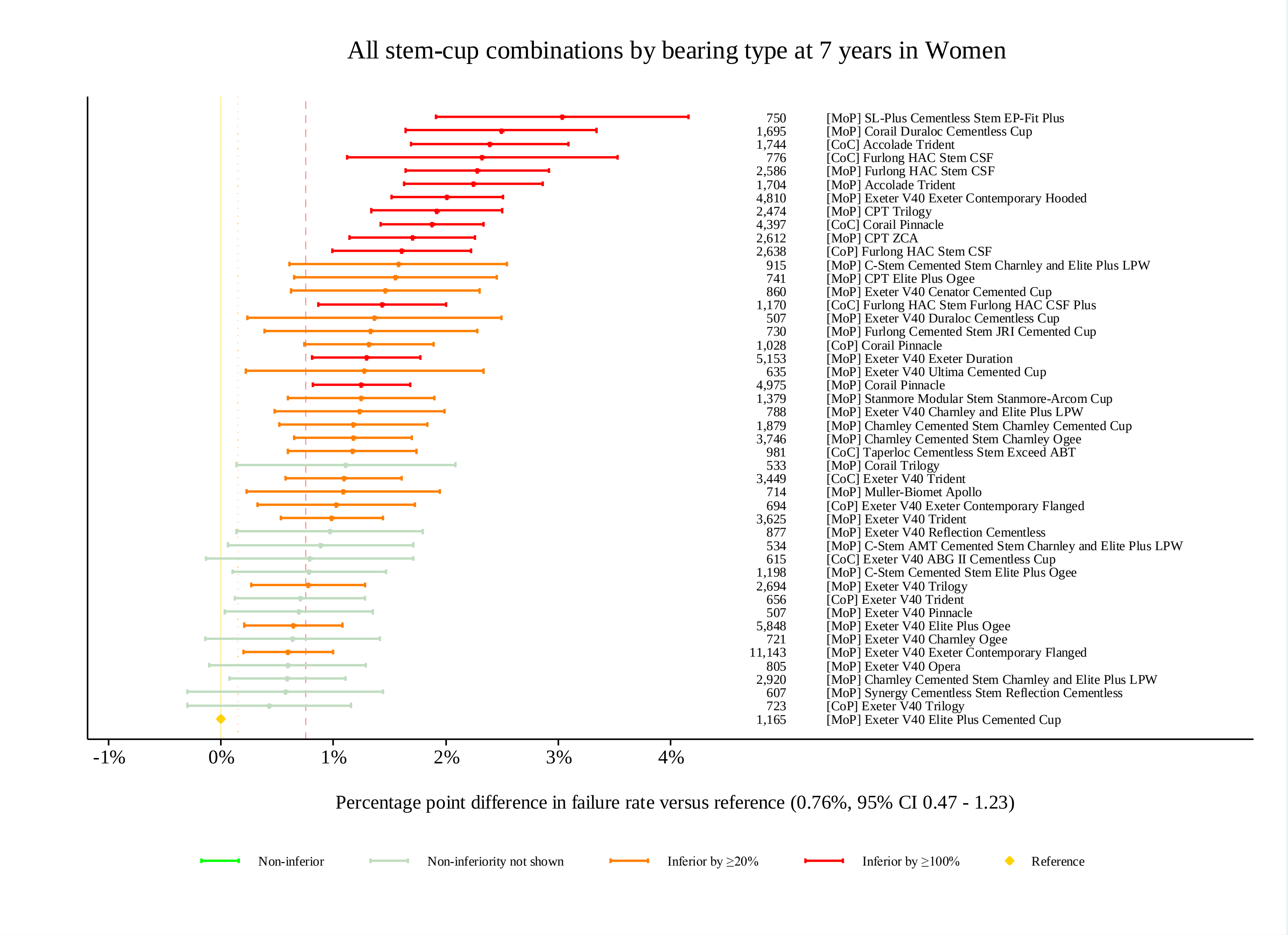

Supplementary Figure 8b: Difference in failure of implanted constructs compared to a contemporary reference at 7 years in women less than 55 years, using all stem-cup combinations with ≥500 procedures remaining at risk

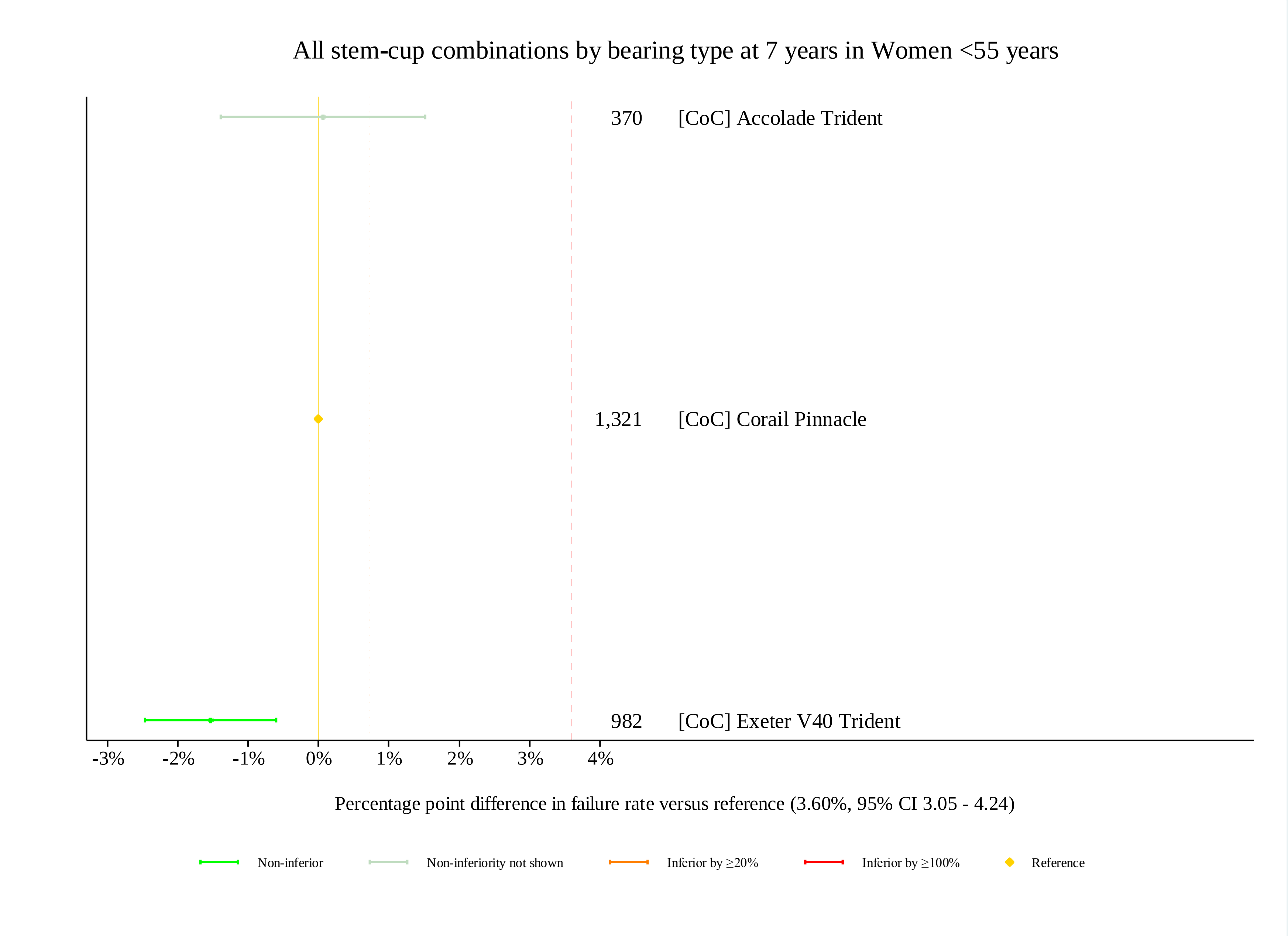

Supplementary Figure 8c: Difference in failure of implanted constructs compared to a contemporary reference at 7 years in women between 55 and 75 years, using all stem-cup combinations with ≥500 procedures remaining at risk

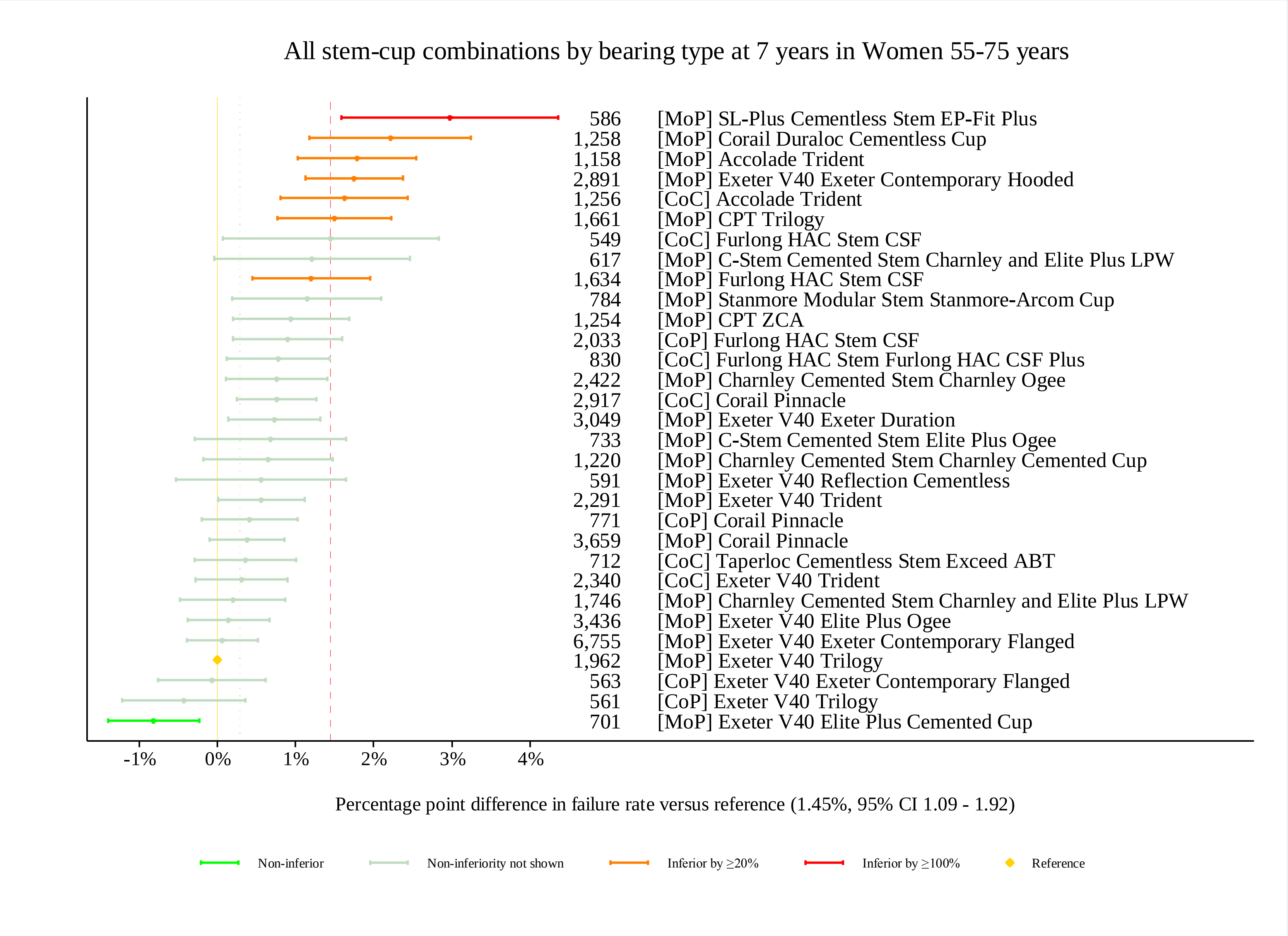

Supplementary Figure 8d: Difference in failure of implanted constructs compared to a contemporary reference at 7 years in women greater than 75 years, using all stem-cup combinations with ≥500 procedures remaining at risk

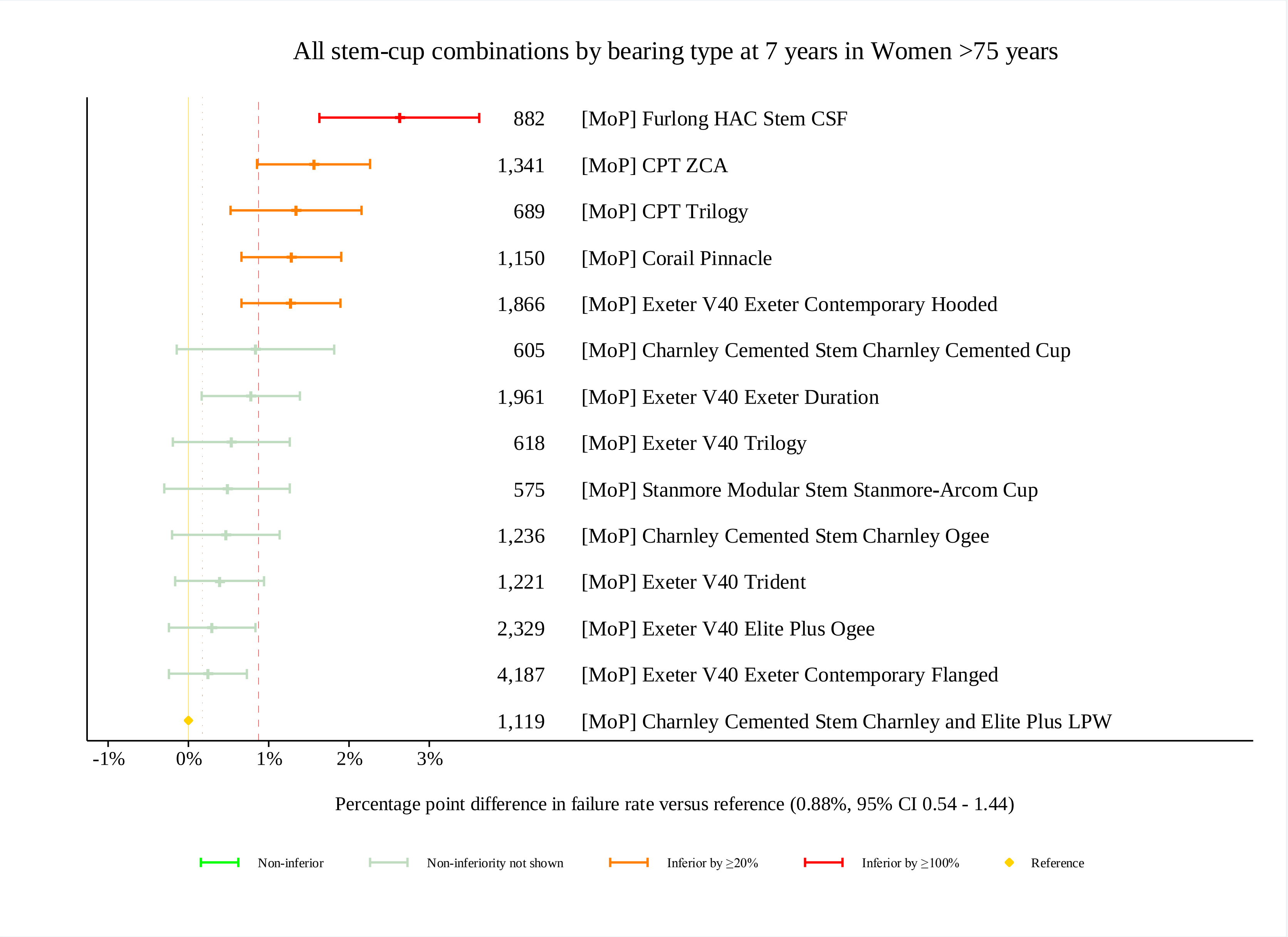

Supplementary Figure 9a: Difference in failure of implanted constructs compared to a contemporary reference at 10 years in women, using all stem-cup combinations with ≥500 procedures remaining at risk

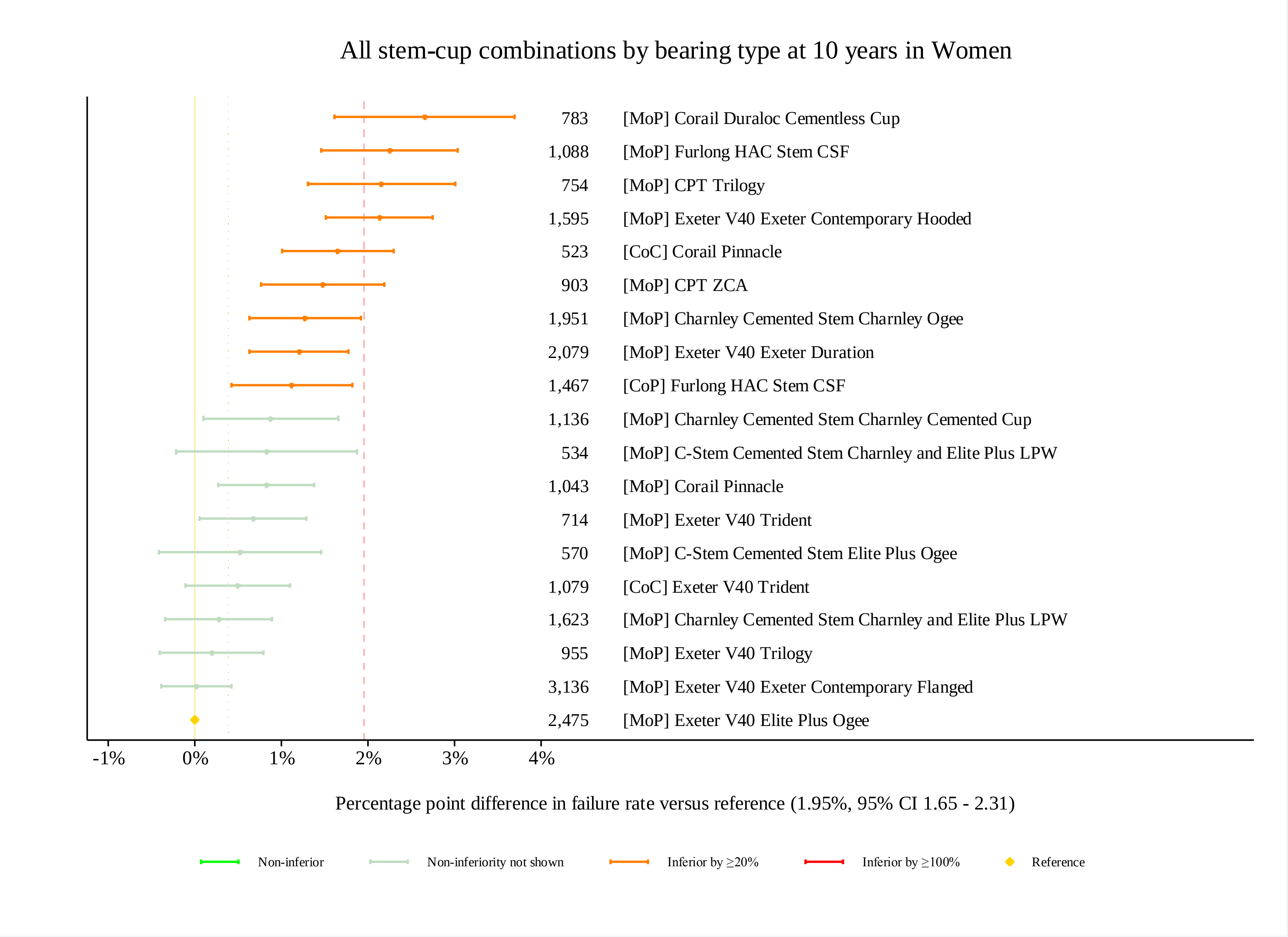

Supplementary Figure 9b: Difference in failure of implanted constructs compared to a contemporary reference at 10 years in women between 55 and 75 years, using all stem-cup combinations with ≥500 procedures remaining at risk

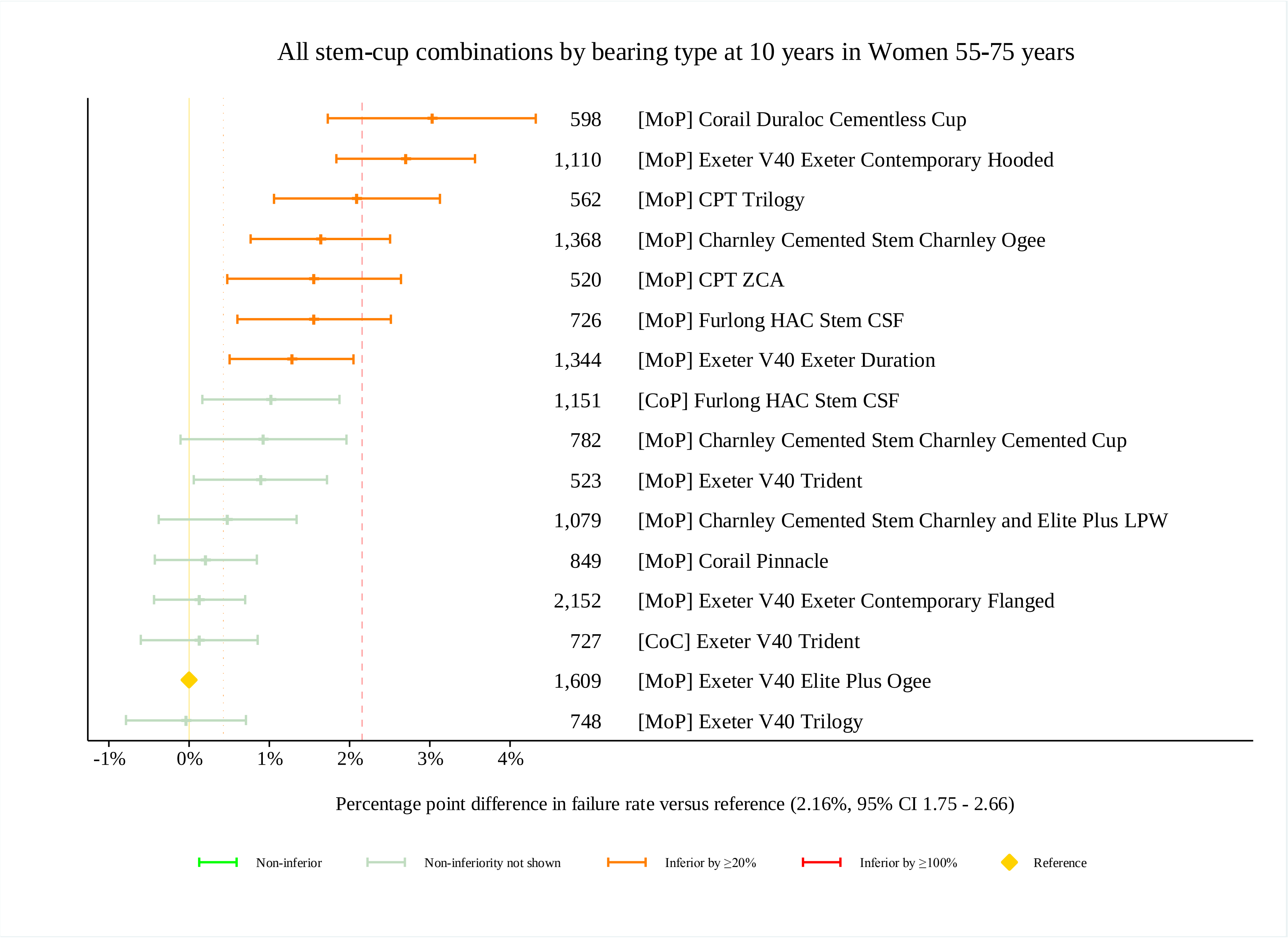

Supplementary table 1a: Difference in Kaplan-Meier failure estimate between a contemporary reference and implants with at least 250 at risk at 3 years since primary

Stem/cup brand	Number at risk	Cumulative failure (%)	Difference in failure (%)	95% CI	Equivalence status	p-value
[CoP] MS-30 Original ME Muller Low Profile Cup	1,554	0.39	[REFERENCE]			
[CoC] ABG II Monolithic Cementless Stem ABG II Cementless Cup	1,061	1.82	1.43	[0.59 , 2.27]	Inferior by ≥100%	0.001
[CoC] ABG II Monolithic Cementless Stem Trident	1,007	1.98	1.59	[0.70 , 2.48]	Inferior by ≥100%	<0.001
[CoC] AMIStem-H Versafit CC Trio	331	2.00	1.61	[0.44 , 2.77]	Inferior by ≥100%	0.007
[CoC] Accolade Trident	6,660	2.03	1.64	[1.20 , 2.08]	Inferior by ≥100%	<0.001
[CoC] Anthology R3 Cementless	327	1.13	0.74	[-0.24 , 1.72]	Non-inferiority not shown	0.140
[CoC] Bicontact Cementless Stem Plasmacup SC	346	1.01	0.62	[-0.41 , 1.65]	Non-inferiority not shown	0.237
[CoC] Bimetric Cementless Stem Exceed ABT	888	1.05	0.65	[-0.03 , 1.34]	Non-inferiority not shown	0.059
[CoC] C-Stem AMT Cemented Stem Pinnacle	770	0.83	0.44	[-0.18 , 1.06]	Non-inferiority not shown	0.168
[CoC] C-Stem Cemented Stem Pinnacle	342	0.54	0.15	[-0.65 , 0.95]	Non-inferiority not shown	0.715
[CoC] CPT Continuum	801	1.71	1.32	[0.52 , 2.13]	Inferior by ≥100%	0.001
[CoC] CPT Trilogy AB	525	1.12	0.73	[-0.21 , 1.67]	Non-inferiority not shown	0.127
[CoC] CPT Trilogy IT	314	1.14	0.74	[-0.13 , 1.62]	Non-inferiority not shown	0.095
[CoC] Charnley Modular Trilogy AB	252	0.39	0.00	[-0.82 , 0.81]	Non-inferiority not shown	0.996
[CoC] Corail Delta TT	726	2.10	1.71	[0.82 , 2.60]	Inferior by ≥100%	<0.001
[CoC] Corail DeltaMotion	1,147	1.40	1.01	[0.32 , 1.70]	Inferior by ≥20%	0.004
[CoC] Corail Duraloc Option	1,042	1.95	1.56	[0.68 , 2.43]	Inferior by ≥100%	<0.001
[CoC] Corail Pinnacle	27,047	1.79	1.40	[1.07 , 1.72]	Inferior by ≥100%	<0.001
[CoC] Corail Pinnacle Gription	452	2.41	2.02	[0.97 , 3.07]	Inferior by ≥100%	<0.001
[CoC] Corail Trinity	431	0.65	0.26	[-0.38 , 0.89]	Non-inferiority not shown	0.430
[CoC] Excia Cementless Plasmacup SC	888	1.20	0.81	[0.12 , 1.50]	Inferior by ≥20%	0.022
[CoC] Exeter V40 ABG II Cementless Cup	1,343	0.64	0.25	[-0.24 , 0.75]	Non-inferiority not shown	0.315
[CoC] Exeter V40 Trident	10,273	1.01	0.62	[0.27 , 0.96]	Inferior by ≥20%	<0.001
[CoC] Furlong Evolution Cementless Furlong HAC CSF Plus	597	1.37	0.98	[0.36 , 1.60]	Inferior by ≥20%	0.002
[CoC] Furlong HAC Stem CSF	1,568	2.08	1.69	[0.94 , 2.44]	Inferior by ≥100%	<0.001
[CoC] Furlong HAC Stem Furlong HAC CSF Plus	8,977	1.59	1.20	[0.83 , 1.57]	Inferior by ≥100%	<0.001
[CoC] Furlong HAC Stem Furlong Threaded	323	2.10	1.71	[0.14 , 3.28]	Inferior by ≥20%	0.033
[CoC] M/L Taper Cementless Continuum	1,393	1.63	1.24	[0.60 , 1.88]	Inferior by ≥100%	<0.001
[CoC] M/L Taper Cementless Trilogy IT	257	2.94	2.55	[0.93 , 4.18]	Inferior by ≥100%	0.002
[CoC] M/L Taper Kinectiv Cementless Continuum	258	3.39	3.00	[0.91 , 5.08]	Inferior by ≥100%	0.005
[CoC] Metafix Stem Trinity	813	1.29	0.90	[0.24 , 1.55]	Inferior by ≥20%	0.007
[CoC] Omnifit Cementless Stem Trident	486	1.99	1.60	[0.34 , 2.85]	Inferior by ≥20%	0.013
[CoC] Polarstem Cementless R3 Cementless	800	0.78	0.39	[-0.20 , 0.97]	Non-inferiority not shown	0.195
[CoC] S-Rom Cementless Stem Pinnacle	543	3.12	2.73	[1.38 , 4.08]	Inferior by ≥100%	<0.001
[CoC] SL-Plus Cementless Stem EP-Fit Plus	1,202	3.46	3.07	[2.03 , 4.12]	Inferior by ≥100%	<0.001
[CoC] SL-Plus Cementless Stem R3 Cementless	539	0.80	0.41	[-0.35 , 1.16]	Non-inferiority not shown	0.292
[CoC] SPS Modular April - Ceramic	593	1.50	1.11	[0.14 , 2.07]	Inferior by ≥20%	0.025
[CoC] Summit Cementless Stem Pinnacle	482	1.33	0.94	[0.02 , 1.87]	Non-inferiority not shown	0.046
[CoC] Synergy Cementless Stem R3 Cementless	280	0.96	0.57	[-0.41 , 1.54]	Non-inferiority not shown	0.257

[CoC] Taperloc Cementless Stem Exceed ABT	6,704	1.52	1.13	[0.75 , 1.51]	Inferior by ≥100%	<0.001
[CoC] Trilock BPS Pinnacle	341	2.06	1.67	[0.36 , 2.98]	Inferior by ≥20%	0.012
[CoC] miniHip Trinity	713	1.91	1.52	[0.64 , 2.41]	Inferior by ≥100%	0.001
[CoP] Accolade Trident	3,079	1.52	1.12	[0.67 , 1.58]	Inferior by ≥100%	<0.001
[CoP] C-Stem AMT Cemented Stem Marathon	321	0.80	0.41	[-0.34 , 1.15]	Non-inferiority not shown	0.285
[CoP] C-Stem AMT Cemented Stem Pinnacle	346	1.63	1.24	[0.36 , 2.12]	Inferior by ≥20%	0.006
[CoP] C-Stem Cemented Stem Elite Plus Ogee	607	0.57	0.18	[-0.45 , 0.80]	Non-inferiority not shown	0.583
[CoP] C-Stem Cemented Stem Marathon	1,150	1.03	0.64	[0.10 , 1.19]	Inferior by ≥20%	0.021
[CoP] C-Stem Cemented Stem Opera	785	0.63	0.23	[-0.38 , 0.85]	Non-inferiority not shown	0.457
[CoP] C-Stem Cemented Stem Wroblewski Golf Ball	913	0.69	0.30	[-0.29 , 0.88]	Non-inferiority not shown	0.320
[CoP] CLS Cementless Stem Trilogy	283	0.59	0.20	[-0.67 , 1.06]	Non-inferiority not shown	0.656
[CoP] CPT Trilogy	1,321	1.47	1.08	[0.59 , 1.58]	Inferior by ≥100%	<0.001
[CoP] CPT ZCA	349	0.39	-0.01	[-0.62 , 0.61]	Non-inferiority not shown	0.987
[CoP] Corail Charnley and Elite Plus LPW	627	1.72	1.33	[0.32 , 2.34]	Inferior by ≥20%	0.010
[CoP] Corail Duraloc Cementless Cup	301	4.40	4.01	[1.74 , 6.28]	Inferior by ≥100%	0.001
[CoP] Corail Elite Plus Cemented Cup	409	0.95	0.56	[-0.32 , 1.43]	Non-inferiority not shown	0.214
[CoP] Corail Elite Plus Ogee	464	1.70	1.31	[0.17 , 2.46]	Inferior by ≥20%	0.024
[CoP] Corail Marathon	1,309	1.12	0.73	[0.20 , 1.26]	Inferior by ≥20%	0.007
[CoP] Corail Pinnacle	8,130	1.21	0.82	[0.48 , 1.16]	Inferior by ≥100%	<0.001
[CoP] Corail Trabecular Metal Modular Cementless Cup	335	2.76	2.36	[0.85 , 3.88]	Inferior by ≥100%	0.002
[CoP] Corail Trident	300	0.26	-0.13	[-0.72 , 0.45]	Non-inferiority not shown	0.660
[CoP] Corail Trilogy	654	1.31	0.92	[0.02 , 1.81]	Non-inferiority not shown	0.045
[CoP] Excia Cementless Plasmacup SC	301	0.96	0.57	[-0.32 , 1.47]	Non-inferiority not shown	0.210
[CoP] Exeter V40 Charnley and Elite Plus LPW	674	1.59	1.20	[0.32 , 2.07]	Inferior by ≥20%	0.008
[CoP] Exeter V40 Elite Plus Ogee	1,190	0.70	0.31	[-0.20 , 0.81]	Non-inferiority not shown	0.231
[CoP] Exeter V40 Exeter Contemporary Flanged	2,995	0.99	0.60	[0.18 , 1.03]	Inferior by ≥20%	0.005
[CoP] Exeter V40 Exeter Contemporary Hooded	779	2.03	1.64	[0.70 , 2.57]	Inferior by ≥100%	0.001
[CoP] Exeter V40 Exeter Duration	775	0.66	0.27	[-0.33 , 0.88]	Non-inferiority not shown	0.378
[CoP] Exeter V40 Exeter X3 Rimfit	2,175	0.99	0.60	[0.18 , 1.02]	Inferior by ≥20%	0.005
[CoP] Exeter V40 Marathon	485	0.70	0.31	[-0.34 , 0.96]	Non-inferiority not shown	0.349
[CoP] Exeter V40 Pinnacle	542	1.14	0.75	[0.07 , 1.42]	Non-inferiority not shown	0.030
[CoP] Exeter V40 Trident	4,857	0.88	0.48	[0.14 , 0.83]	Inferior by ≥20%	0.006
[CoP] Exeter V40 Trilogy	1,945	1.05	0.66	[0.14 , 1.17]	Inferior by ≥20%	0.013
[CoP] Exeter V40 Tritanium	437	1.91	1.52	[0.60 , 2.45]	Inferior by ≥100%	0.001
[CoP] Furlong HAC Stem CSF	6,284	1.26	0.87	[0.48 , 1.26]	Inferior by ≥100%	<0.001
[CoP] Furlong HAC Stem Furlong HAC CSF Plus	1,538	1.84	1.45	[0.81 , 2.09]	Inferior by ≥100%	<0.001
[CoP] Furlong HAC Stem Furlong Threaded	388	0.50	0.11	[-0.64 , 0.86]	Non-inferiority not shown	0.778
[CoP] M/L Taper Cementless Continuum	391	2.79	2.40	[1.26 , 3.53]	Inferior by ≥100%	<0.001
[CoP] Muller Straight Stem Original ME Muller Low Profile Cup	364	1.22	0.83	[-0.19 , 1.85]	Non-inferiority not shown	0.110
[CoP] SL-Plus Cementless Stem Bicon-Plus	620	2.56	2.17	[0.93 , 3.40]	Inferior by ≥100%	0.001
[CoP] SL-Plus Cementless Stem EP-Fit Plus	958	1.89	1.50	[0.61 , 2.39]	Inferior by ≥100%	0.001
[CoP] SP II Cemented Stem Interplanta	310	0.00	- -	[-.-- , -.--]	No failures to date	
[CoP] Stanmore Modular Stem Stanmore-Arcom Cup	355	1.19	0.80	[-0.28 , 1.88]	Non-inferiority not shown	0.145
[CoP] Taperloc Cementless Stem Exceed ABT	1,879	1.07	0.68	[0.23 , 1.13]	Inferior by ≥20%	0.003
[CoP] Versys Cementless Stem Trilogy	363	1.07	0.68	[-0.40 , 1.76]	Non-inferiority not shown	0.220
[MoP] ABG II Monolithic Cementless Stem ABG II Cementless Cup	442	1.70	1.31	[0.10 , 2.51]	Inferior by ≥20%	0.033

[MoP] ABG II Monolithic Cementless Stem Trident	440	3.19	2.80	[1.18 , 4.42]	Inferior by ≥100%	0.001
[MoP] Accolade Trident	9,084	2.01	1.62	[1.23 , 2.02]	Inferior by ≥100%	<0.001
[MoP] Anthology R3 Cementless	1,155	1.76	1.36	[0.73 , 2.00]	Inferior by ≥100%	<0.001
[MoP] Bimetric Cementless Stem Mallory-Head Cementless Cup	359	2.05	1.66	[0.42 , 2.90]	Inferior by ≥100%	0.009
[MoP] C-Stem AMT Cemented Stem Charnley and Elite Plus LPW	2,153	1.15	0.76	[0.26 , 1.26]	Inferior by ≥20%	0.003
[MoP] C-Stem AMT Cemented Stem Elite Plus Cemented Cup	612	0.88	0.48	[-0.27 , 1.24]	Non-inferiority not shown	0.209
[MoP] C-Stem AMT Cemented Stem Elite Plus Ogee	1,608	0.77	0.38	[-0.09 , 0.85]	Non-inferiority not shown	0.116
[MoP] C-Stem AMT Cemented Stem Marathon	1,157	0.93	0.54	[0.07 , 1.00]	Non-inferiority not shown	0.023
[MoP] C-Stem AMT Cemented Stem Pinnacle	1,478	1.22	0.83	[0.35 , 1.31]	Inferior by ≥20%	0.001
[MoP] C-Stem Cemented Stem Charnley Ogee	847	1.31	0.92	[0.15 , 1.68]	Inferior by ≥20%	0.019
[MoP] C-Stem Cemented Stem Charnley and Elite Plus LPW	1,594	1.05	0.66	[0.10 , 1.23]	Inferior by ≥20%	0.022
[MoP] C-Stem Cemented Stem Duraloc Cementless Cup	535	2.10	1.71	[0.50 , 2.92]	Inferior by ≥100%	0.006
[MoP] C-Stem Cemented Stem Elite Plus Cemented Cup	650	0.56	0.17	[-0.45 , 0.79]	Non-inferiority not shown	0.590
[MoP] C-Stem Cemented Stem Elite Plus Ogee	3,208	0.88	0.49	[0.07 , 0.91]	Non-inferiority not shown	0.022
[MoP] C-Stem Cemented Stem Marathon	1,556	0.92	0.53	[0.05 , 1.00]	Non-inferiority not shown	0.029
[MoP] C-Stem Cemented Stem Opera	1,363	1.04	0.65	[0.05 , 1.25]	Non-inferiority not shown	0.033
[MoP] C-Stem Cemented Stem Pinnacle	417	1.56	1.17	[0.06 , 2.28]	Non-inferiority not shown	0.039
[MoP] C-Stem Cemented Stem Polarcup Cementless	268	0.71	0.32	[-0.70 , 1.35]	Non-inferiority not shown	0.539
[MoP] C-Stem Cemented Stem Trilogy	364	0.53	0.14	[-0.65 , 0.93]	Non-inferiority not shown	0.730
[MoP] C-Stem Cemented Stem Wroblewski Golf Ball	978	0.94	0.55	[-0.10 , 1.20]	Non-inferiority not shown	0.098
[MoP] CCA Cemented Stem CCB Cup	1,129	0.57	0.18	[-0.31 , 0.68]	Non-inferiority not shown	0.469
[MoP] CLS Cementless Stem Allofit	600	2.56	2.17	[0.93 , 3.40]	Inferior by ≥100%	0.001
[MoP] CLS Cementless Stem Trilogy	559	2.80	2.40	[1.06 , 3.75]	Inferior by ≥100%	<0.001
[MoP] CMK Modular Cemented Stem CMK Cemented Cup	462	0.41	0.02	[-0.62 , 0.67]	Non-inferiority not shown	0.943
[MoP] CPCS Opera	1,174	0.82	0.42	[-0.16 , 1.01]	Non-inferiority not shown	0.152
[MoP] CPCS Polarcup Cementless	400	0.49	0.09	[-0.64 , 0.83]	Non-inferiority not shown	0.801
[MoP] CPCS Reflection Cemented	382	1.02	0.63	[-0.14 , 1.40]	Non-inferiority not shown	0.107
[MoP] CPS Plus Cenator Cemented Cup	381	0.26	-0.13	[-0.72 , 0.46]	Non-inferiority not shown	0.662
[MoP] CPS Plus EP-Fit Plus	301	1.58	1.19	[-0.21 , 2.60]	Non-inferiority not shown	0.097
[MoP] CPS Plus Opera	490	1.55	1.16	[0.05 , 2.27]	Non-inferiority not shown	0.040
[MoP] CPT Allofit	686	0.55	0.16	[-0.38 , 0.69]	Non-inferiority not shown	0.561
[MoP] CPT Continuum	480	2.60	2.20	[1.33 , 3.08]	Inferior by ≥100%	<0.001
[MoP] CPT Elite Plus Ogee	2,410	1.38	0.99	[0.47 , 1.52]	Inferior by ≥100%	<0.001
[MoP] CPT Exceed	284	3.02	2.63	[0.66 , 4.59]	Inferior by ≥100%	0.009
[MoP] CPT Exeter Contemporary Flanged	386	2.10	1.71	[0.50 , 2.92]	Inferior by ≥100%	0.006
[MoP] CPT Opera	406	1.40	1.01	[-0.14 , 2.16]	Non-inferiority not shown	0.086
[MoP] CPT Original ME Muller Low Profile Cup	833	1.20	0.81	[0.09 , 1.53]	Inferior by ≥20%	0.028
[MoP] CPT Pinnacle	753	1.70	1.31	[0.38 , 2.24]	Inferior by ≥20%	0.006
[MoP] CPT Trabecular Metal Modular Cementless Cup	891	1.66	1.27	[0.50 , 2.03]	Inferior by ≥100%	0.001
[MoP] CPT Trilogy	8,372	1.37	0.98	[0.62 , 1.34]	Inferior by ≥100%	<0.001
[MoP] CPT Trilogy IT	450	2.89	2.50	[1.61 , 3.40]	Inferior by ≥100%	<0.001
[MoP] CPT ZCA	7,529	1.39	1.00	[0.63 , 1.37]	Inferior by ≥100%	<0.001
[MoP] Centrament Chirulen	378	0.24	-0.15	[-0.71 , 0.41]	Non-inferiority not shown	0.608
[MoP] Charnley Cemented Stem Charnley Cemented Cup	4,078	1.11	0.71	[0.29 , 1.14]	Inferior by ≥20%	0.001
[MoP] Charnley Cemented Stem Charnley Ogee	8,701	1.18	0.79	[0.43 , 1.15]	Inferior by ≥100%	<0.001
[MoP] Charnley Cemented Stem Charnley and Elite Plus LPW	5,770	0.72	0.32	[-0.03 , 0.68]	Non-inferiority not shown	0.075

[MoP] Charnley Cemented Stem Opera	1,229	0.85	0.46	[-0.12 , 1.04]	Non-inferiority not shown	0.119
[MoP] Charnley Cemented Stem Wroblewski Golf Ball	990	1.50	1.10	[0.32 , 1.89]	Inferior by ≥20%	0.006
[MoP] Charnley Modular Charnley and Elite Plus LPW	363	0.27	-0.12	[-0.73 , 0.49]	Non-inferiority not shown	0.702
[MoP] Corail Charnley and Elite Plus LPW	723	1.36	0.97	[0.18 , 1.76]	Inferior by ≥20%	0.017
[MoP] Corail Duraloc Cementless Cup	3,449	1.41	1.02	[0.54 , 1.50]	Inferior by ≥100%	<0.001
[MoP] Corail Elite Plus Cemented Cup	964	0.81	0.42	[-0.19 , 1.02]	Non-inferiority not shown	0.175
[MoP] Corail Elite Plus Ogee	1,407	1.22	0.83	[0.24 , 1.42]	Inferior by ≥20%	0.005
[MoP] Corail Exeter Contemporary Flanged	571	1.13	0.73	[-0.06 , 1.53]	Non-inferiority not shown	0.070
[MoP] Corail Marathon	2,848	1.05	0.66	[0.24 , 1.07]	Inferior by ≥20%	0.002
[MoP] Corail Pinnacle	28,253	1.37	0.98	[0.67 , 1.29]	Inferior by ≥100%	<0.001
[MoP] Corail Pinnacle Gription	382	1.54	1.15	[0.34 , 1.95]	Inferior by ≥20%	0.005
[MoP] Corail Trident	585	1.54	1.15	[0.33 , 1.96]	Inferior by ≥20%	0.006
[MoP] Corail Trilogy	1,791	1.14	0.74	[0.20 , 1.29]	Inferior by ≥20%	0.007
[MoP] Elite Plus Cemented Stem Charnley and Elite Plus LPW	331	1.66	1.27	[-0.08 , 2.62]	Non-inferiority not shown	0.065
[MoP] Elite Plus Cemented Stem Elite Plus Ogee	576	0.34	-0.05	[-0.60 , 0.50]	Non-inferiority not shown	0.848
[MoP] Excia Cementless Plasmacup SC	316	3.20	2.81	[1.00 , 4.62]	Inferior by ≥100%	0.002
[MoP] Exeter Elite Plus Ogee	282	0.33	-0.06	[-0.77 , 0.65]	Non-inferiority not shown	0.871
[MoP] Exeter V40 ABG II Cementless Cup	734	1.28	0.89	[0.05 , 1.73]	Non-inferiority not shown	0.038
[MoP] Exeter V40 Cenator Cemented Cup	2,129	1.40	1.01	[0.45 , 1.57]	Inferior by ≥100%	<0.001
[MoP] Exeter V40 Charnley Cemented Cup	314	0.87	0.48	[-0.55 , 1.51]	Non-inferiority not shown	0.358
[MoP] Exeter V40 Charnley Ogee	1,315	0.95	0.56	[-0.02 , 1.14]	Non-inferiority not shown	0.057
[MoP] Exeter V40 Charnley and Elite Plus LPW	2,477	1.25	0.86	[0.37 , 1.35]	Inferior by ≥20%	0.001
[MoP] Exeter V40 Duraloc Cementless Cup	1,061	1.46	1.06	[0.30 , 1.83]	Inferior by ≥20%	0.006
[MoP] Exeter V40 EP-Fit Plus	539	1.44	1.05	[0.02 , 2.08]	Non-inferiority not shown	0.046
[MoP] Exeter V40 Elite Plus Cemented Cup	3,599	0.58	0.19	[-0.18 , 0.56]	Non-inferiority not shown	0.314
[MoP] Exeter V40 Elite Plus Ogee	16,477	0.79	0.40	[0.08 , 0.71]	Inferior by ≥20%	0.014
[MoP] Exeter V40 Exceed	494	0.94	0.55	[-0.32 , 1.42]	Non-inferiority not shown	0.215
[MoP] Exeter V40 Exceed ABT	456	0.60	0.21	[-0.45 , 0.87]	Non-inferiority not shown	0.528
[MoP] Exeter V40 Exeter Contemporary Flanged	40,794	0.85	0.46	[0.15 , 0.76]	Inferior by ≥20%	0.003
[MoP] Exeter V40 Exeter Contemporary Hooded	16,021	1.53	1.14	[0.80 , 1.47]	Inferior by ≥100%	<0.001
[MoP] Exeter V40 Exeter Duration	12,935	1.23	0.84	[0.50 , 1.18]	Inferior by ≥100%	<0.001
[MoP] Exeter V40 Exeter X3 Rimfit	5,262	0.96	0.57	[0.23 , 0.92]	Inferior by ≥20%	0.001
[MoP] Exeter V40 Furlong HAC CSF Plus	504	0.35	-0.04	[-0.53 , 0.46]	Non-inferiority not shown	0.882
[MoP] Exeter V40 Marathon	1,300	1.05	0.66	[0.15 , 1.18]	Inferior by ≥20%	0.012
[MoP] Exeter V40 Opera	2,373	0.81	0.42	[-0.03 , 0.88]	Non-inferiority not shown	0.067
[MoP] Exeter V40 Pinnacle	2,590	1.18	0.79	[0.35 , 1.24]	Inferior by ≥20%	<0.001
[MoP] Exeter V40 R3 Cementless	461	1.23	0.84	[0.10 , 1.57]	Inferior by ≥20%	0.025
[MoP] Exeter V40 Reflection Cementless	2,035	1.12	0.73	[0.19 , 1.26]	Inferior by ≥20%	0.007
[MoP] Exeter V40 Trabecular Metal Modular Cementless Cup	411	1.22	0.83	[-0.12 , 1.77]	Non-inferiority not shown	0.086
[MoP] Exeter V40 Trabecular Metal Natural Cup	318	3.43	3.04	[1.11 , 4.97]	Inferior by ≥100%	0.002
[MoP] Exeter V40 Trident	17,802	1.10	0.71	[0.39 , 1.02]	Inferior by ≥20%	<0.001
[MoP] Exeter V40 Trilogy	8,482	0.92	0.53	[0.19 , 0.88]	Inferior by ≥20%	0.003
[MoP] Exeter V40 Tritanium	475	2.06	1.67	[0.71 , 2.63]	Inferior by ≥100%	0.001
[MoP] Exeter V40 Ultima Cemented Cup	1,139	1.56	1.17	[0.41 , 1.92]	Inferior by ≥100%	0.002
[MoP] Exeter V40 ZCA	274	0.36	-0.03	[-0.79 , 0.73]	Non-inferiority not shown	0.933
[MoP] Furlong Cemented Stem JRI Cemented Cup	1,478	1.38	0.99	[0.34 , 1.63]	Inferior by ≥20%	0.003

[MoP] Furlong HAC Stem CSF	6,640	2.07	1.68	[1.25 , 2.11]	Inferior by ≥100%	<0.001
[MoP] Furlong HAC Stem Furlong HAC CSF Plus	2,998	2.24	1.85	[1.33 , 2.37]	Inferior by ≥100%	<0.001
[MoP] Furlong HAC Stem Furlong Threaded	479	2.13	1.74	[0.46 , 3.02]	Inferior by ≥100%	0.008
[MoP] Furlong HAC Stem Trilogy	341	1.90	1.51	[0.09 , 2.94]	Inferior by ≥20%	0.038
[MoP] M/L Taper Cementless Allofit	543	1.27	0.88	[0.00 , 1.76]	Non-inferiority not shown	0.049
[MoP] M/L Taper Cementless Continuum	612	1.40	1.01	[0.28 , 1.74]	Inferior by ≥20%	0.007
[MoP] M/L Taper Cementless Trilogy	475	2.08	1.69	[0.63 , 2.74]	Inferior by ≥100%	0.002
[MoP] M/L Taper Cementless Trilogy IT	270	1.86	1.47	[0.46 , 2.47]	Inferior by ≥100%	0.004
[MoP] MS-30 Allofit	314	0.57	0.17	[-0.66 , 1.01]	Non-inferiority not shown	0.683
[MoP] MS-30 Original ME Muller Low Profile Cup	756	0.58	0.19	[-0.36 , 0.74]	Non-inferiority not shown	0.497
[MoP] Mem Original ME Muller Low Profile Cup	337	0.56	0.17	[-0.66 , 1.00]	Non-inferiority not shown	0.688
[MoP] Muller Straight Stem Centerpulse Muller	502	0.95	0.56	[-0.32 , 1.43]	Non-inferiority not shown	0.213
[MoP] Muller Straight Stem Original ME Muller Low Profile Cup	1,412	0.81	0.41	[-0.09 , 0.92]	Non-inferiority not shown	0.106
[MoP] Muller-Biomet Apollo	1,949	1.24	0.85	[0.30 , 1.41]	Inferior by ≥20%	0.003
[MoP] Muller-Biomet Original ME Muller Low Profile Cup	831	1.56	1.17	[0.31 , 2.03]	Inferior by ≥20%	0.008
[MoP] Omnifit Cemented Stem ODC	880	1.40	1.01	[0.20 , 1.82]	Inferior by ≥20%	0.015
[MoP] Omnifit Cemented Stem Trident	270	0.36	-0.03	[-0.80 , 0.74]	Non-inferiority not shown	0.947
[MoP] Omnifit Cementless Stem Secure Fit Cementless Cup	283	0.69	0.30	[-0.70 , 1.30]	Non-inferiority not shown	0.554
[MoP] Omnifit Cementless Stem Trident	426	3.34	2.95	[1.31 , 4.58]	Inferior by ≥100%	<0.001
[MoP] P10 Muller Original ME Muller Low Profile Cup	415	0.96	0.57	[-0.33 , 1.47]	Non-inferiority not shown	0.213
[MoP] Polarstem Cementless R3 Cementless	1,068	0.96	0.57	[0.14 , 1.00]	Inferior by ≥20%	0.010
[MoP] SL-Plus Cementless Stem EP-Fit Plus	2,023	2.60	2.21	[1.52 , 2.90]	Inferior by ≥100%	<0.001
[MoP] SP II Cemented Stem Interplanta	625	2.72	2.33	[1.09 , 3.57]	Inferior by ≥100%	<0.001
[MoP] SP II Cemented Stem Link Flange Cup	274	0.69	0.29	[-0.70 , 1.29]	Non-inferiority not shown	0.560
[MoP] Spectron Reflection Cementless	298	1.24	0.84	[-0.39 , 2.08]	Non-inferiority not shown	0.182
[MoP] Stanmore Modular Stem Elite Plus Cemented Cup	534	0.35	-0.05	[-0.61 , 0.51]	Non-inferiority not shown	0.872
[MoP] Stanmore Modular Stem SHP Cup	931	0.82	0.43	[-0.18 , 1.03]	Non-inferiority not shown	0.169
[MoP] Stanmore Modular Stem Stanmore-Arcom Cup	3,798	1.09	0.70	[0.27 , 1.12]	Inferior by ≥20%	0.001
[MoP] Synergy Cementless Stem R3 Cementless	1,057	1.25	0.86	[0.31 , 1.41]	Inferior by ≥20%	0.002
[MoP] Synergy Cementless Stem Reflection Cementless	1,449	0.87	0.47	[-0.08 , 1.03]	Non-inferiority not shown	0.092
[MoP] Taperfit Cemented Stem Atlas IIIp	444	0.84	0.45	[-0.34 , 1.24]	Non-inferiority not shown	0.268
[MoP] Taperloc Cemented Stem Exceed ABT	280	0.97	0.58	[-0.21 , 1.38]	Non-inferiority not shown	0.152
[MoP] Taperloc Cementless Stem Exceed	553	1.20	0.81	[-0.12 , 1.73]	Non-inferiority not shown	0.089
[MoP] Taperloc Cementless Stem Exceed ABT	3,732	1.75	1.36	[0.91 , 1.80]	Inferior by ≥100%	<0.001
[MoP] Trilock BPS Pinnacle	264	1.65	1.26	[0.01 , 2.51]	Non-inferiority not shown	0.048
[MoP] Trilock BPS Pinnacle Gription	275	1.37	0.98	[-0.19 , 2.15]	Non-inferiority not shown	0.100
[MoP] VerSys Cemented Stem Trilogy	263	1.11	0.71	[-0.56 , 1.99]	Non-inferiority not shown	0.273
[MoP] Versys Cementless Stem Trilogy	860	4.01	3.62	[2.32 , 4.91]	Inferior by ≥100%	<0.001

Supplementary table 1b: Difference in Kaplan-Meier failure estimate between a contemporary reference and implants with at least 250 at risk at 5 years since primary

Stem/cup brand	Number at risk	Cumulative failure (%)	Difference in failure (%)	95% CI	Equivalence status	p-value
[CoP] MS-30 Original ME Muller Low Profile Cup	1,125	0.55	[REFERENCE]			
[CoC] ABG II Monolithic Cementless Stem ABG II Cementless Cup	1,020	2.29	1.73	[0.77 , 2.70]	Inferior by ≥100%	<0.001
[CoC] ABG II Monolithic Cementless Stem Trident	834	3.07	2.51	[1.38 , 3.64]	Inferior by ≥100%	<0.001
[CoC] Accolade Trident	5,169	2.83	2.27	[1.74 , 2.81]	Inferior by ≥100%	<0.001
[CoC] Bicontact Cementless Stem Plasmacup SC	289	1.01	0.46	[-0.60 , 1.51]	Non-inferiority not shown	0.395
[CoC] Bimetric Cementless Stem Exceed ABT	627	1.80	1.24	[0.31 , 2.17]	Inferior by ≥20%	0.009
[CoC] C-Stem AMT Cemented Stem Pinnacle	427	1.30	0.74	[-0.10 , 1.59]	Non-inferiority not shown	0.085
[CoC] C-Stem Cemented Stem Pinnacle	310	1.44	0.88	[-0.42 , 2.19]	Non-inferiority not shown	0.185
[CoC] CPT Continuum	379	2.40	1.85	[0.77 , 2.92]	Inferior by ≥100%	0.001
[CoC] CPT Trilogy AB	468	1.70	1.15	[-0.02 , 2.31]	Non-inferiority not shown	0.053
[CoC] Corail DeltaMotion	633	1.64	1.08	[0.28 , 1.89]	Inferior by ≥20%	0.008
[CoC] Corail Duraloc Option	1,014	2.99	2.44	[1.35 , 3.52]	Inferior by ≥100%	<0.001
[CoC] Corail Pinnacle	17,510	2.40	1.85	[1.44 , 2.26]	Inferior by ≥100%	<0.001
[CoC] Excia Cementless Plasmacup SC	501	1.33	0.77	[0.00 , 1.54]	Non-inferiority not shown	0.049
[CoC] Exeter V40 ABG II Cementless Cup	1,138	1.20	0.64	[-0.04 , 1.32]	Non-inferiority not shown	0.063
[CoC] Exeter V40 Trident	8,141	1.50	0.94	[0.51 , 1.38]	Inferior by ≥20%	<0.001
[CoC] Furlong HAC Stem CSF	1,502	2.59	2.03	[1.18 , 2.89]	Inferior by ≥100%	<0.001
[CoC] Furlong HAC Stem Furlong HAC CSF Plus	5,470	1.84	1.28	[0.84 , 1.73]	Inferior by ≥100%	<0.001
[CoC] Furlong HAC Stem Furlong Threaded	318	3.01	2.46	[0.58 , 4.34]	Inferior by ≥100%	0.010
[CoC] M/L Taper Cementless Continuum	607	1.71	1.15	[0.46 , 1.85]	Inferior by ≥20%	0.001
[CoC] Metafix Stem Trinity	280	1.52	0.96	[0.14 , 1.79]	Inferior by ≥20%	0.022
[CoC] Omnifit Cementless Stem Trident	387	3.10	2.54	[0.95 , 4.13]	Inferior by ≥100%	0.002
[CoC] Polarstem Cementless R3 Cementless	300	0.78	0.22	[-0.40 , 0.85]	Non-inferiority not shown	0.487
[CoC] S-Rom Cementless Stem Pinnacle	407	4.35	3.79	[2.12 , 5.46]	Inferior by ≥100%	<0.001
[CoC] SL-Plus Cementless Stem EP-Fit Plus	1,115	4.96	4.40	[3.14 , 5.66]	Inferior by ≥100%	<0.001
[CoC] SPS Modular April - Ceramic	321	2.99	2.43	[0.95 , 3.92]	Inferior by ≥100%	0.001
[CoC] Summit Cementless Stem Pinnacle	385	1.33	0.78	[-0.18 , 1.73]	Non-inferiority not shown	0.110
[CoC] Taperloc Cementless Stem Exceed ABT	3,900	1.83	1.27	[0.80 , 1.74]	Inferior by ≥100%	<0.001
[CoP] Accolade Trident	1,372	1.95	1.39	[0.82 , 1.97]	Inferior by ≥100%	<0.001
[CoP] C-Stem Cemented Stem Elite Plus Ogee	474	0.75	0.20	[-0.56 , 0.96]	Non-inferiority not shown	0.609
[CoP] C-Stem Cemented Stem Marathon	542	1.34	0.79	[0.10 , 1.47]	Non-inferiority not shown	0.024
[CoP] C-Stem Cemented Stem Opera	656	1.33	0.77	[-0.12 , 1.67]	Non-inferiority not shown	0.091
[CoP] C-Stem Cemented Stem Wroblewski Golf Ball	738	1.05	0.49	[-0.25 , 1.24]	Non-inferiority not shown	0.196
[CoP] CPT ZCA	261	0.39	-0.17	[-0.82 , 0.48]	Non-inferiority not shown	0.611
[CoP] Corail Charnley and Elite Plus LPW	532	1.89	1.33	[0.25 , 2.42]	Inferior by ≥20%	0.016
[CoP] Corail Duraloc Cementless Cup	289	4.72	4.17	[1.81 , 6.53]	Inferior by ≥100%	0.001
[CoP] Corail Elite Plus Cemented Cup	328	1.50	0.95	[-0.24 , 2.13]	Non-inferiority not shown	0.117
[CoP] Corail Elite Plus Ogee	340	2.64	2.08	[0.61 , 3.55]	Inferior by ≥100%	0.006
[CoP] Corail Marathon	573	1.40	0.84	[0.19 , 1.49]	Inferior by ≥20%	0.012
[CoP] Corail Pinnacle	4,134	1.74	1.18	[0.74 , 1.63]	Inferior by ≥100%	<0.001

[CoP] Corail Trilogy	592	1.61	1.06	[0.04 , 2.07]	Non-inferiority not shown	0.041
[CoP] Exeter V40 Charnley and Elite Plus LPW	433	2.02	1.47	[0.38 , 2.55]	Inferior by ≥20%	0.008
[CoP] Exeter V40 Elite Plus Ogee	858	1.08	0.53	[-0.14 , 1.20]	Non-inferiority not shown	0.122
[CoP] Exeter V40 Exeter Contemporary Flanged	2,011	1.31	0.76	[0.23 , 1.28]	Inferior by ≥20%	0.005
[CoP] Exeter V40 Exeter Contemporary Hooded	593	2.58	2.03	[0.93 , 3.12]	Inferior by ≥100%	<0.001
[CoP] Exeter V40 Exeter Duration	664	0.95	0.40	[-0.36 , 1.16]	Non-inferiority not shown	0.304
[CoP] Exeter V40 Exeter X3 Rimfit	363	1.16	0.60	[0.09 , 1.12]	Non-inferiority not shown	0.022
[CoP] Exeter V40 Marathon	258	0.94	0.38	[-0.44 , 1.21]	Non-inferiority not shown	0.365
[CoP] Exeter V40 Pinnacle	297	1.53	0.98	[0.08 , 1.87]	Non-inferiority not shown	0.032
[CoP] Exeter V40 Trident	2,337	1.16	0.60	[0.15 , 1.06]	Inferior by ≥20%	0.009
[CoP] Exeter V40 Trilogy	1,627	1.31	0.76	[0.15 , 1.37]	Inferior by ≥20%	0.015
[CoP] Furlong HAC Stem CSF	5,425	1.65	1.09	[0.61 , 1.57]	Inferior by ≥100%	<0.001
[CoP] Furlong HAC Stem Furlong HAC CSF Plus	908	2.02	1.47	[0.74 , 2.19]	Inferior by ≥100%	<0.001
[CoP] Furlong HAC Stem Furlong Threaded	362	1.29	0.73	[-0.45 , 1.91]	Non-inferiority not shown	0.225
[CoP] Muller Straight Stem Original ME Muller Low Profile Cup	298	1.22	0.67	[-0.38 , 1.71]	Non-inferiority not shown	0.210
[CoP] SL-Plus Cementless Stem Bicon-Plus	558	4.05	3.50	[1.93 , 5.07]	Inferior by ≥100%	<0.001
[CoP] SL-Plus Cementless Stem EP-Fit Plus	810	3.41	2.86	[1.65 , 4.06]	Inferior by ≥100%	<0.001
[CoP] Stanmore Modular Stem Stanmore-Arcom Cup	257	1.19	0.64	[-0.46 , 1.74]	Non-inferiority not shown	0.257
[CoP] Taperloc Cementless Stem Exceed ABT	775	1.43	0.87	[0.26 , 1.49]	Inferior by ≥20%	0.006
[CoP] Versys Cementless Stem Trilogy	342	1.07	0.51	[-0.59 , 1.61]	Non-inferiority not shown	0.363
[MoP] ABG II Monolithic Cementless Stem ABG II Cementless Cup	419	1.92	1.37	[0.07 , 2.66]	Non-inferiority not shown	0.039
[MoP] ABG II Monolithic Cementless Stem Trident	402	4.11	3.56	[1.71 , 5.41]	Inferior by ≥100%	<0.001
[MoP] Accolade Trident	6,100	2.76	2.21	[1.72 , 2.70]	Inferior by ≥100%	<0.001
[MoP] Anthology R3 Cementless	353	2.14	1.59	[0.69 , 2.48]	Inferior by ≥100%	<0.001
[MoP] Bimetric Cementless Stem Mallory-Head Cementless Cup	271	2.05	1.50	[0.23 , 2.76]	Inferior by ≥20%	0.020
[MoP] C-Stem AMT Cemented Stem Charnley and Elite Plus LPW	1,397	1.46	0.90	[0.30 , 1.51]	Inferior by ≥20%	0.003
[MoP] C-Stem AMT Cemented Stem Elite Plus Cemented Cup	435	1.22	0.66	[-0.26 , 1.58]	Non-inferiority not shown	0.157
[MoP] C-Stem AMT Cemented Stem Elite Plus Ogee	918	1.01	0.46	[-0.13 , 1.05]	Non-inferiority not shown	0.130
[MoP] C-Stem AMT Cemented Stem Marathon	356	1.46	0.90	[0.10 , 1.71]	Non-inferiority not shown	0.028
[MoP] C-Stem AMT Cemented Stem Pinnacle	657	1.85	1.29	[0.56 , 2.02]	Inferior by ≥100%	0.001
[MoP] C-Stem Cemented Stem Charnley Ogee	680	1.86	1.30	[0.34 , 2.26]	Inferior by ≥20%	0.008
[MoP] C-Stem Cemented Stem Charnley and Elite Plus LPW	1,435	1.57	1.02	[0.31 , 1.72]	Inferior by ≥20%	0.005
[MoP] C-Stem Cemented Stem Duraloc Cementless Cup	496	2.67	2.12	[0.73 , 3.50]	Inferior by ≥100%	0.003
[MoP] C-Stem Cemented Stem Elite Plus Cemented Cup	586	0.90	0.34	[-0.46 , 1.15]	Non-inferiority not shown	0.405
[MoP] C-Stem Cemented Stem Elite Plus Ogee	2,459	1.13	0.57	[0.06 , 1.08]	Non-inferiority not shown	0.028
[MoP] C-Stem Cemented Stem Marathon	744	1.25	0.70	[0.08 , 1.32]	Non-inferiority not shown	0.028
[MoP] C-Stem Cemented Stem Opera	1,013	1.44	0.89	[0.16 , 1.62]	Inferior by ≥20%	0.017
[MoP] C-Stem Cemented Stem Pinnacle	331	1.56	1.01	[-0.13 , 2.14]	Non-inferiority not shown	0.083
[MoP] C-Stem Cemented Stem Polarcup Cementless	255	0.71	0.16	[-0.89 , 1.21]	Non-inferiority not shown	0.768
[MoP] C-Stem Cemented Stem Trilogy	338	0.82	0.26	[-0.73 , 1.25]	Non-inferiority not shown	0.606
[MoP] C-Stem Cemented Stem Wroblewski Golf Ball	756	1.50	0.94	[0.10 , 1.79]	Non-inferiority not shown	0.028
[MoP] CCA Cemented Stem CCB Cup	738	0.79	0.23	[-0.38 , 0.85]	Non-inferiority not shown	0.459
[MoP] CLS Cementless Stem Allofit	553	2.90	2.34	[1.01 , 3.68]	Inferior by ≥100%	0.001
[MoP] CLS Cementless Stem Trilogy	419	3.18	2.63	[1.17 , 4.09]	Inferior by ≥100%	<0.001
[MoP] CMK Modular Cemented Stem CMK Cemented Cup	418	1.08	0.52	[-0.49 , 1.53]	Non-inferiority not shown	0.310
[MoP] CPCS Opera	866	1.28	0.72	[-0.02 , 1.46]	Non-inferiority not shown	0.056

[MoP] CPCS Polarcup Cementless	368	0.99	0.43	[-0.60 , 1.46]	Non-inferiority not shown	0.410
[MoP] CPS Plus Cenator Cemented Cup	339	0.80	0.25	[-0.73 , 1.23]	Non-inferiority not shown	0.618
[MoP] CPS Plus EP-Fit Plus	282	3.23	2.68	[0.67 , 4.69]	Inferior by ≥100%	0.009
[MoP] CPS Plus Opera	464	2.16	1.61	[0.29 , 2.92]	Inferior by ≥20%	0.017
[MoP] CPT Allofit	430	0.94	0.39	[-0.41 , 1.18]	Non-inferiority not shown	0.339
[MoP] CPT Elite Plus Ogee	1,744	1.87	1.32	[0.67 , 1.97]	Inferior by ≥100%	<0.001
[MoP] CPT Exceed	263	4.10	3.55	[1.24 , 5.85]	Inferior by ≥100%	0.003
[MoP] CPT Opera	378	2.17	1.62	[0.16 , 3.07]	Inferior by ≥20%	0.029
[MoP] CPT Original ME Muller Low Profile Cup	422	2.20	1.64	[0.54 , 2.75]	Inferior by ≥20%	0.004
[MoP] CPT Pinnacle	639	2.42	1.86	[0.72 , 3.00]	Inferior by ≥100%	0.001
[MoP] CPT Trabecular Metal Modular Cementless Cup	523	2.41	1.85	[0.82 , 2.89]	Inferior by ≥100%	<0.001
[MoP] CPT Trilogy	5,765	2.20	1.65	[1.17 , 2.12]	Inferior by ≥100%	<0.001
[MoP] CPT ZCA	5,547	2.11	1.55	[1.07 , 2.03]	Inferior by ≥100%	<0.001
[MoP] Centrament Chirulen	310	1.12	0.57	[-0.59 , 1.72]	Non-inferiority not shown	0.337
[MoP] Charnley Cemented Stem Charnley Cemented Cup	3,601	1.72	1.17	[0.63 , 1.71]	Inferior by ≥100%	<0.001
[MoP] Charnley Cemented Stem Charnley Ogee	7,533	1.88	1.33	[0.86 , 1.79]	Inferior by ≥100%	<0.001
[MoP] Charnley Cemented Stem Charnley and Elite Plus LPW	5,041	1.12	0.56	[0.11 , 1.02]	Non-inferiority not shown	0.015
[MoP] Charnley Cemented Stem Opera	1,039	1.46	0.91	[0.14 , 1.67]	Inferior by ≥20%	0.020
[MoP] Charnley Cemented Stem Wroblewski Golf Ball	797	1.74	1.18	[0.30 , 2.06]	Inferior by ≥20%	0.008
[MoP] Charnley Modular Charnley and Elite Plus LPW	277	0.27	-0.28	[-0.93 , 0.37]	Non-inferiority not shown	0.393
[MoP] Corail Charnley and Elite Plus LPW	448	1.85	1.30	[0.30 , 2.29]	Inferior by ≥20%	0.010
[MoP] Corail Duraloc Cementless Cup	3,226	2.27	1.71	[1.10 , 2.33]	Inferior by ≥100%	<0.001
[MoP] Corail Elite Plus Cemented Cup	727	1.31	0.75	[-0.05 , 1.56]	Non-inferiority not shown	0.067
[MoP] Corail Elite Plus Ogee	1,029	1.70	1.14	[0.41 , 1.88]	Inferior by ≥20%	0.002
[MoP] Corail Exeter Contemporary Flanged	328	1.35	0.79	[-0.14 , 1.72]	Non-inferiority not shown	0.095
[MoP] Corail Marathon	1,385	1.23	0.67	[0.17 , 1.18]	Inferior by ≥20%	0.009
[MoP] Corail Pinnacle	16,408	1.70	1.15	[0.76 , 1.54]	Inferior by ≥100%	<0.001
[MoP] Corail Trilogy	1,383	1.72	1.17	[0.47 , 1.87]	Inferior by ≥20%	0.001
[MoP] Elite Plus Cemented Stem Charnley and Elite Plus LPW	308	1.66	1.11	[-0.26 , 2.47]	Non-inferiority not shown	0.113
[MoP] Elite Plus Cemented Stem Elite Plus Ogee	519	1.06	0.50	[-0.42 , 1.42]	Non-inferiority not shown	0.284
[MoP] Exeter Elite Plus Ogee	259	0.71	0.15	[-0.89 , 1.20]	Non-inferiority not shown	0.775
[MoP] Exeter V40 ABG II Cementless Cup	693	1.56	1.00	[0.05 , 1.95]	Non-inferiority not shown	0.038
[MoP] Exeter V40 Cenator Cemented Cup	1,725	1.99	1.44	[0.75 , 2.12]	Inferior by ≥100%	<0.001
[MoP] Exeter V40 Charnley Cemented Cup	286	0.87	0.32	[-0.73 , 1.37]	Non-inferiority not shown	0.553
[MoP] Exeter V40 Charnley Ogee	1,113	1.44	0.88	[0.15 , 1.61]	Inferior by ≥20%	0.018
[MoP] Exeter V40 Charnley and Elite Plus LPW	1,812	1.47	0.91	[0.34 , 1.48]	Inferior by ≥20%	0.002
[MoP] Exeter V40 Duraloc Cementless Cup	1,000	2.04	1.48	[0.56 , 2.40]	Inferior by ≥100%	0.002
[MoP] Exeter V40 EP-Fit Plus	496	2.56	2.01	[0.63 , 3.38]	Inferior by ≥100%	0.004
[MoP] Exeter V40 Elite Plus Cemented Cup	2,674	0.76	0.21	[-0.25 , 0.66]	Non-inferiority not shown	0.376
[MoP] Exeter V40 Elite Plus Ogee	12,458	1.11	0.56	[0.16 , 0.96]	Inferior by ≥20%	0.006
[MoP] Exeter V40 Exceed	375	0.94	0.39	[-0.51 , 1.29]	Non-inferiority not shown	0.399
[MoP] Exeter V40 Exceed ABT	305	1.17	0.62	[-0.43 , 1.67]	Non-inferiority not shown	0.251
[MoP] Exeter V40 Exeter Contemporary Flanged	26,691	1.21	0.65	[0.27 , 1.03]	Inferior by ≥20%	0.001
[MoP] Exeter V40 Exeter Contemporary Hooded	11,227	2.10	1.54	[1.12 , 1.96]	Inferior by ≥100%	<0.001
[MoP] Exeter V40 Exeter Duration	10,271	1.70	1.15	[0.72 , 1.57]	Inferior by ≥100%	<0.001
[MoP] Exeter V40 Exeter X3 Rimfit	859	1.16	0.61	[0.14 , 1.07]	Inferior by ≥20%	0.010

[MoP] Exeter V40 Marathon	555	1.54	0.99	[0.24 , 1.73]	Inferior by ≥20%	0.010
[MoP] Exeter V40 Opera	1,734	1.18	0.63	[0.06 , 1.19]	Non-inferiority not shown	0.030
[MoP] Exeter V40 Pinnacle	1,546	1.60	1.05	[0.48 , 1.62]	Inferior by ≥20%	<0.001
[MoP] Exeter V40 Reflection Cementless	1,812	1.58	1.02	[0.37 , 1.67]	Inferior by ≥20%	0.002
[MoP] Exeter V40 Trabecular Metal Modular Cementless Cup	276	2.23	1.68	[0.19 , 3.17]	Inferior by ≥20%	0.028
[MoP] Exeter V40 Trabecular Metal Natural Cup	284	4.06	3.51	[1.39 , 5.63]	Inferior by ≥100%	0.001
[MoP] Exeter V40 Trident	10,507	1.44	0.88	[0.48 , 1.29]	Inferior by ≥20%	<0.001
[MoP] Exeter V40 Trilogy	6,611	1.33	0.77	[0.34 , 1.21]	Inferior by ≥20%	0.001
[MoP] Exeter V40 Ultima Cemented Cup	1,040	2.19	1.64	[0.73 , 2.55]	Inferior by ≥100%	<0.001
[MoP] Furlong Cemented Stem JRI Cemented Cup	1,287	1.66	1.11	[0.37 , 1.84]	Inferior by ≥20%	0.003
[MoP] Furlong HAC Stem CSF	5,416	2.43	1.87	[1.36 , 2.38]	Inferior by ≥100%	<0.001
[MoP] Furlong HAC Stem Furlong HAC CSF Plus	1,808	2.91	2.35	[1.70 , 3.01]	Inferior by ≥100%	<0.001
[MoP] Furlong HAC Stem Furlong Threaded	416	3.01	2.46	[0.91 , 4.00]	Inferior by ≥100%	0.002
[MoP] Furlong HAC Stem Trilogy	264	1.90	1.35	[-0.10 , 2.80]	Non-inferiority not shown	0.067
[MoP] M/L Taper Cementless Allofit	295	2.04	1.48	[0.22 , 2.74]	Inferior by ≥20%	0.021
[MoP] M/L Taper Cementless Trilogy	328	2.54	1.98	[0.73 , 3.23]	Inferior by ≥100%	0.002
[MoP] MS-30 Allofit	269	0.57	0.01	[-0.85 , 0.87]	Non-inferiority not shown	0.981
[MoP] MS-30 Original ME Muller Low Profile Cup	531	1.23	0.67	[-0.19 , 1.54]	Non-inferiority not shown	0.128
[MoP] Mem Original ME Muller Low Profile Cup	313	0.86	0.30	[-0.73 , 1.34]	Non-inferiority not shown	0.567
[MoP] Muller Straight Stem Centerpulse Muller	442	1.79	1.24	[0.02 , 2.46]	Non-inferiority not shown	0.047
[MoP] Muller Straight Stem Original ME Muller Low Profile Cup	1,008	1.12	0.56	[-0.07 , 1.19]	Non-inferiority not shown	0.080
[MoP] Muller-Biomet Apollo	1,539	1.41	0.86	[0.23 , 1.49]	Inferior by ≥20%	0.008
[MoP] Muller-Biomet Original ME Muller Low Profile Cup	512	1.96	1.41	[0.41 , 2.40]	Inferior by ≥20%	0.006
[MoP] Omnifit Cemented Stem ODC	801	1.98	1.43	[0.45 , 2.41]	Inferior by ≥20%	0.004
[MoP] Omnifit Cemented Stem Trident	250	1.89	1.34	[-0.35 , 3.02]	Non-inferiority not shown	0.120
[MoP] Omnifit Cementless Stem Secure Fit Cementless Cup	275	0.69	0.14	[-0.89 , 1.16]	Non-inferiority not shown	0.791
[MoP] Omnifit Cementless Stem Trident	385	5.46	4.90	[2.78 , 7.02]	Inferior by ≥100%	<0.001
[MoP] P10 Muller Original ME Muller Low Profile Cup	293	0.96	0.41	[-0.52 , 1.34]	Non-inferiority not shown	0.388
[MoP] SL-Plus Cementless Stem EP-Fit Plus	1,651	3.51	2.96	[2.12 , 3.80]	Inferior by ≥100%	<0.001
[MoP] SP II Cemented Stem Interplanta	552	3.23	2.67	[1.29 , 4.05]	Inferior by ≥100%	<0.001
[MoP] Spectron Reflection Cementless	267	1.24	0.68	[-0.58 , 1.94]	Non-inferiority not shown	0.289
[MoP] Stanmore Modular Stem Elite Plus Cemented Cup	406	0.98	0.42	[-0.51 , 1.36]	Non-inferiority not shown	0.375
[MoP] Stanmore Modular Stem SHP Cup	677	0.95	0.40	[-0.30 , 1.10]	Non-inferiority not shown	0.265
[MoP] Stanmore Modular Stem Stanmore-Arcom Cup	2,920	1.62	1.06	[0.52 , 1.60]	Inferior by ≥20%	<0.001
[MoP] Synergy Cementless Stem Reflection Cementless	1,370	1.43	0.87	[0.16 , 1.59]	Inferior by ≥20%	0.016
[MoP] Taperfit Cemented Stem Atlas IIIp	250	2.18	1.63	[0.20 , 3.06]	Inferior by ≥20%	0.026
[MoP] Taperloc Cementless Stem Exceed	470	1.57	1.01	[-0.07 , 2.10]	Non-inferiority not shown	0.066
[MoP] Taperloc Cementless Stem Exceed ABT	1,793	2.07	1.52	[0.98 , 2.06]	Inferior by ≥100%	<0.001
[MoP] Versys Cementless Stem Trilogy	797	4.81	4.26	[2.82 , 5.69]	Inferior by ≥100%	<0.001

Supplementary table 1c: Difference in Kaplan-Meier failure estimate between a contemporary reference and implants with at least 250 at risk at 7 years since primary

Stem/cup brand	Number at risk	Cumulative failure (%)	Difference in failure (%)	95% CI	Equivalence status	p-value
[MoP] Exeter V40 Elite Plus Cemented Cup	1,773	0.91	[REFERENCE]			
[CoC] ABG II Monolithic Cementless Stem ABG II Cementless Cup	909	3.09	2.19	[1.10 , 3.27]	Inferior by ≥100%	<0.001
[CoC] ABG II Monolithic Cementless Stem Trident	705	3.68	2.77	[1.55 , 4.00]	Inferior by ≥100%	<0.001
[CoC] Accolade Trident	2,978	3.31	2.40	[1.86 , 2.95]	Inferior by ≥100%	<0.001
[CoC] C-Stem Cemented Stem Pinnacle	286	1.44	0.53	[-0.76 , 1.82]	Non-inferiority not shown	0.420
[CoC] CPT Trilogy AB	265	1.93	1.03	[-0.20 , 2.26]	Non-inferiority not shown	0.101
[CoC] Corail Duraloc Option	819	3.61	2.71	[1.54 , 3.88]	Inferior by ≥100%	<0.001
[CoC] Corail Pinnacle	7,589	2.93	2.03	[1.65 , 2.40]	Inferior by ≥100%	<0.001
[CoC] Exeter V40 ABG II Cementless Cup	918	1.69	0.79	[0.01 , 1.57]	Non-inferiority not shown	0.048
[CoC] Exeter V40 Trident	5,732	1.99	1.09	[0.67 , 1.51]	Inferior by ≥20%	<0.001
[CoC] Furlong HAC Stem CSF	1,405	3.19	2.29	[1.37 , 3.21]	Inferior by ≥100%	<0.001
[CoC] Furlong HAC Stem Furlong HAC CSF Plus	2,083	2.15	1.25	[0.81 , 1.69]	Inferior by ≥20%	<0.001
[CoC] Furlong HAC Stem Furlong Threaded	308	4.24	3.34	[1.14 , 5.53]	Inferior by ≥100%	0.003
[CoC] Omnifit Cementless Stem Trident	313	4.21	3.31	[1.41 , 5.21]	Inferior by ≥100%	0.001
[CoC] SL-Plus Cementless Stem EP-Fit Plus	968	5.32	4.41	[3.12 , 5.70]	Inferior by ≥100%	<0.001
[CoC] Summit Cementless Stem Pinnacle	301	1.60	0.69	[-0.37 , 1.76]	Non-inferiority not shown	0.203
[CoC] Taperloc Cementless Stem Exceed ABT	1,609	2.02	1.12	[0.65 , 1.58]	Inferior by ≥20%	<0.001
[CoP] Accolade Trident	544	2.20	1.29	[0.64 , 1.95]	Inferior by ≥20%	<0.001
[CoP] C-Stem Cemented Stem Elite Plus Ogee	342	0.75	-0.15	[-0.89 , 0.58]	Non-inferiority not shown	0.684
[CoP] C-Stem Cemented Stem Opera	478	1.69	0.79	[-0.22 , 1.80]	Non-inferiority not shown	0.127
[CoP] C-Stem Cemented Stem Wroblewski Golf Ball	598	1.67	0.76	[-0.18 , 1.70]	Non-inferiority not shown	0.112
[CoP] Corail Charnley and Elite Plus LPW	335	2.49	1.59	[0.32 , 2.85]	Inferior by ≥20%	0.014
[CoP] Corail Duraloc Cementless Cup	266	5.06	4.15	[1.72 , 6.59]	Inferior by ≥100%	0.001
[CoP] Corail Pinnacle	1,638	2.16	1.25	[0.78 , 1.73]	Inferior by ≥20%	<0.001
[CoP] Corail Trilogy	477	1.61	0.71	[-0.29 , 1.71]	Non-inferiority not shown	0.164
[CoP] Exeter V40 Charnley and Elite Plus LPW	250	2.02	1.12	[0.05 , 2.19]	Non-inferiority not shown	0.040
[CoP] Exeter V40 Elite Plus Ogee	535	1.35	0.44	[-0.29 , 1.18]	Non-inferiority not shown	0.240
[CoP] Exeter V40 Exeter Contemporary Flanged	1,110	1.42	0.52	[0.00 , 1.03]	Non-inferiority not shown	0.048
[CoP] Exeter V40 Exeter Contemporary Hooded	410	3.14	2.23	[0.98 , 3.48]	Inferior by ≥100%	<0.001
[CoP] Exeter V40 Exeter Duration	500	2.11	1.21	[0.08 , 2.33]	Non-inferiority not shown	0.035
[CoP] Exeter V40 Trident	1,113	1.46	0.56	[0.07 , 1.05]	Non-inferiority not shown	0.026
[CoP] Exeter V40 Trilogy	1,159	1.58	0.67	[0.04 , 1.30]	Non-inferiority not shown	0.037
[CoP] Furlong HAC Stem CSF	4,459	2.07	1.16	[0.69 , 1.64]	Inferior by ≥20%	<0.001
[CoP] Furlong HAC Stem Furlong HAC CSF Plus	345	2.46	1.55	[0.69 , 2.41]	Inferior by ≥20%	<0.001
[CoP] Furlong HAC Stem Furlong Threaded	327	1.84	0.94	[-0.45 , 2.33]	Non-inferiority not shown	0.187
[CoP] MS-30 Original ME Muller Low Profile Cup	675	0.70	-0.21	[-0.77 , 0.35]	Non-inferiority not shown	0.460
[CoP] SL-Plus Cementless Stem Bicon-Plus	406	4.66	3.75	[2.06 , 5.45]	Inferior by ≥100%	<0.001
[CoP] SL-Plus Cementless Stem EP-Fit Plus	579	4.10	3.20	[1.87 , 4.53]	Inferior by ≥100%	<0.001
[CoP] Versys Cementless Stem Trilogy	303	1.36	0.46	[-0.77 , 1.69]	Non-inferiority not shown	0.465
[MoP] ABG II Monolithic Cementless Stem ABG II Cementless Cup	376	2.40	1.50	[0.06 , 2.94]	Non-inferiority not shown	0.041

[MoP] ABG II Monolithic Cementless Stem Trident	301	5.72	4.81	[2.60 , 7.02]	Inferior by ≥100%	<0.001
[MoP] Accolade Trident	2,764	3.50	2.59	[2.07 , 3.11]	Inferior by ≥100%	<0.001
[MoP] C-Stem AMT Cemented Stem Charnley and Elite Plus LPW	782	1.81	0.91	[0.21 , 1.61]	Inferior by ≥20%	0.011
[MoP] C-Stem AMT Cemented Stem Elite Plus Ogee	523	1.71	0.81	[-0.02 , 1.64]	Non-inferiority not shown	0.057
[MoP] C-Stem AMT Cemented Stem Pinnacle	298	2.08	1.18	[0.34 , 2.02]	Inferior by ≥20%	0.006
[MoP] C-Stem Cemented Stem Charnley Ogee	539	2.03	1.12	[0.12 , 2.12]	Non-inferiority not shown	0.028
[MoP] C-Stem Cemented Stem Charnley and Elite Plus LPW	1,298	1.94	1.04	[0.29 , 1.79]	Inferior by ≥20%	0.007
[MoP] C-Stem Cemented Stem Duraloc Cementless Cup	391	3.73	2.83	[1.18 , 4.47]	Inferior by ≥100%	0.001
[MoP] C-Stem Cemented Stem Elite Plus Cemented Cup	472	1.62	0.71	[-0.34 , 1.76]	Non-inferiority not shown	0.184
[MoP] C-Stem Cemented Stem Elite Plus Ogee	1,851	1.55	0.65	[0.10 , 1.19]	Non-inferiority not shown	0.020
[MoP] C-Stem Cemented Stem Opera	744	2.01	1.10	[0.24 , 1.96]	Inferior by ≥20%	0.012
[MoP] C-Stem Cemented Stem Pinnacle	258	1.94	1.04	[-0.30 , 2.38]	Non-inferiority not shown	0.130
[MoP] C-Stem Cemented Stem Trilogy	278	1.11	0.21	[-0.92 , 1.34]	Non-inferiority not shown	0.719
[MoP] C-Stem Cemented Stem Wroblewski Golf Ball	502	1.88	0.97	[0.00 , 1.94]	Non-inferiority not shown	0.050
[MoP] CCA Cemented Stem CCB Cup	328	1.69	0.78	[-0.20 , 1.77]	Non-inferiority not shown	0.119
[MoP] CLS Cementless Stem Allofit	350	3.77	2.87	[1.30 , 4.44]	Inferior by ≥100%	<0.001
[MoP] CMK Modular Cemented Stem CMK Cemented Cup	329	1.08	0.17	[-0.82 , 1.16]	Non-inferiority not shown	0.734
[MoP] CPCS Opera	324	2.86	1.95	[0.78 , 3.12]	Inferior by ≥20%	0.001
[MoP] CPS Plus Cenator Cemented Cup	250	0.80	-0.10	[-1.06 , 0.86]	Non-inferiority not shown	0.836
[MoP] CPS Plus Opera	421	3.49	2.58	[0.92 , 4.25]	Inferior by ≥100%	0.002
[MoP] CPT Elite Plus Ogee	1,177	2.37	1.47	[0.75 , 2.19]	Inferior by ≥20%	<0.001
[MoP] CPT Opera	276	3.01	2.11	[0.39 , 3.82]	Inferior by ≥20%	0.016
[MoP] CPT Trabecular Metal Modular Cementless Cup	270	3.00	2.09	[0.78 , 3.40]	Inferior by ≥20%	0.002
[MoP] CPT Trilogy	3,606	2.69	1.78	[1.31 , 2.26]	Inferior by ≥100%	<0.001
[MoP] CPT ZCA	3,619	2.69	1.78	[1.30 , 2.27]	Inferior by ≥100%	<0.001
[MoP] Centrament Chirulen	257	1.12	0.22	[-0.93 , 1.36]	Non-inferiority not shown	0.710
[MoP] Charnley Cemented Stem Charnley Cemented Cup	3,004	2.31	1.41	[0.84 , 1.97]	Inferior by ≥20%	<0.001
[MoP] Charnley Cemented Stem Charnley Ogee	5,956	2.49	1.58	[1.13 , 2.04]	Inferior by ≥100%	<0.001
[MoP] Charnley Cemented Stem Charnley and Elite Plus LPW	4,036	1.51	0.60	[0.15 , 1.05]	Non-inferiority not shown	0.009
[MoP] Charnley Cemented Stem Opera	733	2.08	1.18	[0.29 , 2.07]	Inferior by ≥20%	0.009
[MoP] Charnley Cemented Stem Wroblewski Golf Ball	659	1.87	0.96	[0.07 , 1.86]	Non-inferiority not shown	0.035
[MoP] Corail Duraloc Cementless Cup	2,661	3.39	2.48	[1.79 , 3.18]	Inferior by ≥100%	<0.001
[MoP] Corail Elite Plus Cemented Cup	467	1.82	0.91	[-0.06 , 1.88]	Non-inferiority not shown	0.065
[MoP] Corail Elite Plus Ogee	696	1.83	0.92	[0.17 , 1.68]	Non-inferiority not shown	0.016
[MoP] Corail Marathon	354	1.49	0.59	[0.03 , 1.15]	Non-inferiority not shown	0.038
[MoP] Corail Pinnacle	7,798	2.17	1.26	[0.90 , 1.63]	Inferior by ≥20%	<0.001
[MoP] Corail Trilogy	855	2.65	1.75	[0.86 , 2.63]	Inferior by ≥20%	<0.001
[MoP] Elite Plus Cemented Stem Charnley and Elite Plus LPW	281	2.00	1.10	[-0.41 , 2.60]	Non-inferiority not shown	0.154
[MoP] Elite Plus Cemented Stem Elite Plus Ogee	465	1.26	0.35	[-0.63 , 1.33]	Non-inferiority not shown	0.480
[MoP] Exeter V40 ABG II Cementless Cup	626	2.02	1.11	[0.05 , 2.18]	Non-inferiority not shown	0.040
[MoP] Exeter V40 Cenator Cemented Cup	1,274	2.26	1.36	[0.65 , 2.07]	Inferior by ≥20%	<0.001
[MoP] Exeter V40 Charnley Ogee	901	1.62	0.71	[-0.03 , 1.46]	Non-inferiority not shown	0.061
[MoP] Exeter V40 Charnley and Elite Plus LPW	1,082	1.81	0.91	[0.29 , 1.53]	Inferior by ≥20%	0.004
[MoP] Exeter V40 Duraloc Cementless Cup	754	2.24	1.33	[0.39 , 2.27]	Inferior by ≥20%	0.005
[MoP] Exeter V40 EP-Fit Plus	424	3.64	2.74	[1.09 , 4.38]	Inferior by ≥100%	0.001
[MoP] Exeter V40 Elite Plus Ogee	8,638	1.50	0.59	[0.22 , 0.96]	Inferior by ≥20%	0.002

[MoP] Exeter V40 Exceed	265	0.94	0.04	[-0.84 , 0.92]	Non-inferiority not shown	0.934
[MoP] Exeter V40 Exeter Contemporary Flanged	16,415	1.58	0.67	[0.33 , 1.01]	Inferior by ≥20%	<0.001
[MoP] Exeter V40 Exeter Contemporary Hooded	7,073	2.79	1.89	[1.47 , 2.30]	Inferior by ≥100%	<0.001
[MoP] Exeter V40 Exeter Duration	7,252	2.45	1.55	[1.13 , 1.97]	Inferior by ≥100%	<0.001
[MoP] Exeter V40 Opera	1,144	1.59	0.68	[0.06 , 1.31]	Non-inferiority not shown	0.032
[MoP] Exeter V40 Pinnacle	718	1.89	0.98	[0.35 , 1.61]	Inferior by ≥20%	0.002
[MoP] Exeter V40 Reflection Cementless	1,359	2.31	1.41	[0.66 , 2.15]	Inferior by ≥20%	<0.001
[MoP] Exeter V40 Trident	5,685	1.85	0.95	[0.57 , 1.33]	Inferior by ≥20%	<0.001
[MoP] Exeter V40 Trilogy	4,380	1.67	0.77	[0.34 , 1.19]	Inferior by ≥20%	<0.001
[MoP] Exeter V40 Ultima Cemented Cup	916	2.29	1.38	[0.47 , 2.29]	Inferior by ≥20%	0.003
[MoP] Furlong Cemented Stem JRI Cemented Cup	1,041	1.99	1.09	[0.31 , 1.86]	Inferior by ≥20%	0.006
[MoP] Furlong HAC Stem CSF	4,138	3.06	2.15	[1.63 , 2.67]	Inferior by ≥100%	<0.001
[MoP] Furlong HAC Stem Furlong HAC CSF Plus	731	3.34	2.44	[1.72 , 3.16]	Inferior by ≥100%	<0.001
[MoP] Furlong HAC Stem Furlong Threaded	348	3.27	2.37	[0.75 , 3.98]	Inferior by ≥20%	0.004
[MoP] MS-30 Original ME Muller Low Profile Cup	334	1.74	0.83	[-0.27 , 1.93]	Non-inferiority not shown	0.139
[MoP] Mem Original ME Muller Low Profile Cup	286	0.86	-0.05	[-1.06 , 0.97]	Non-inferiority not shown	0.926
[MoP] Muller Straight Stem Centerpulse Muller	316	3.15	2.24	[0.56 , 3.92]	Inferior by ≥20%	0.009
[MoP] Muller Straight Stem Original ME Muller Low Profile Cup	646	2.06	1.16	[0.27 , 2.04]	Inferior by ≥20%	0.010
[MoP] Muller-Biomet Apollo	1,089	1.70	0.80	[0.13 , 1.46]	Non-inferiority not shown	0.019
[MoP] Muller-Biomet Original ME Muller Low Profile Cup	267	2.22	1.32	[0.21 , 2.42]	Inferior by ≥20%	0.019
[MoP] Omnifit Cemented Stem ODC	708	2.78	1.88	[0.73 , 3.03]	Inferior by ≥20%	0.001
[MoP] Omnifit Cementless Stem Secure Fit Cementless Cup	255	1.42	0.52	[-0.90 , 1.93]	Non-inferiority not shown	0.476
[MoP] Omnifit Cementless Stem Trident	321	6.51	5.61	[3.28 , 7.94]	Inferior by ≥100%	<0.001
[MoP] SL-Plus Cementless Stem EP-Fit Plus	1,230	4.42	3.52	[2.58 , 4.46]	Inferior by ≥100%	<0.001
[MoP] SP II Cemented Stem Interplanta	455	4.00	3.09	[1.54 , 4.65]	Inferior by ≥100%	<0.001
[MoP] Stanmore Modular Stem SHP Cup	480	1.46	0.55	[-0.33 , 1.44]	Non-inferiority not shown	0.222
[MoP] Stanmore Modular Stem Stanmore-Arcom Cup	1,908	1.97	1.06	[0.51 , 1.61]	Inferior by ≥20%	<0.001
[MoP] Synergy Cementless Stem Reflection Cementless	1,002	1.75	0.85	[0.10 , 1.60]	Non-inferiority not shown	0.027
[MoP] Taperloc Cementless Stem Exceed	370	2.27	1.37	[0.04 , 2.69]	Non-inferiority not shown	0.043
[MoP] Taperloc Cementless Stem Exceed ABT	513	2.52	1.61	[0.96 , 2.26]	Inferior by ≥100%	<0.001
[MoP] Versys Cementless Stem Trilogy	714	4.81	3.91	[2.48 , 5.33]	Inferior by ≥100%	<0.001

Supplementary table 1d: Difference in Kaplan-Meier failure estimate between a contemporary reference and implants with at least 250 at risk at 10 years since primary

Stem/cup brand	Number at risk	Cumulative failure (%)	Difference in failure (%)	95% CI	Equivalence status	p-value
[MoP] Exeter V40 Elite Plus Ogee	3,580	2.14	[REFERENCE]			
[CoC] ABG II Monolithic Cementless Stem ABG II Cementless Cup	695	4.42	2.28	[0.96 , 3.60]	Inferior by ≥20%	0.001
[CoC] ABG II Monolithic Cementless Stem Trident	412	4.15	2.01	[0.68 , 3.34]	Inferior by ≥20%	0.003
[CoC] Accolade Trident	454	4.38	2.24	[1.48 , 3.00]	Inferior by ≥20%	<0.001
[CoC] Corail Duraloc Option	383	4.45	2.31	[0.93 , 3.68]	Inferior by ≥20%	0.001
[CoC] Corail Pinnacle	886	3.90	1.76	[1.23 , 2.29]	Inferior by ≥20%	<0.001
[CoC] Exeter V40 ABG II Cementless Cup	440	2.66	0.52	[-0.54 , 1.58]	Non-inferiority not shown	0.334
[CoC] Exeter V40 Trident	1,846	2.59	0.44	[-0.04 , 0.93]	Non-inferiority not shown	0.072
[CoC] Furlong HAC Stem CSF	829	4.37	2.22	[1.13 , 3.32]	Inferior by ≥20%	<0.001
[CoC] SL-Plus Cementless Stem EP-Fit Plus	288	6.94	4.80	[3.25 , 6.35]	Inferior by ≥100%	<0.001
[CoP] C-Stem Cemented Stem Wroblewski Golf Ball	317	2.51	0.36	[-0.88 , 1.61]	Non-inferiority not shown	0.567
[CoP] Exeter V40 Exeter Contemporary Flanged	269	2.10	-0.05	[-0.90 , 0.80]	Non-inferiority not shown	0.913
[CoP] Exeter V40 Trilogy	494	1.91	-0.23	[-0.96 , 0.50]	Non-inferiority not shown	0.537
[CoP] Furlong HAC Stem CSF	2,520	2.65	0.51	[-0.02 , 1.03]	Non-inferiority not shown	0.058
[MoP] ABG II Monolithic Cementless Stem ABG II Cementless Cup	278	4.37	2.22	[0.21 , 4.24]	Non-inferiority not shown	0.031
[MoP] C-Stem Cemented Stem Charnley Ogee	293	2.25	0.11	[-0.97 , 1.19]	Non-inferiority not shown	0.846
[MoP] C-Stem Cemented Stem Charnley and Elite Plus LPW	725	2.45	0.30	[-0.54 , 1.14]	Non-inferiority not shown	0.481
[MoP] C-Stem Cemented Stem Elite Plus Cemented Cup	267	2.49	0.35	[-1.08 , 1.79]	Non-inferiority not shown	0.632
[MoP] C-Stem Cemented Stem Elite Plus Ogee	882	2.57	0.43	[-0.33 , 1.19]	Non-inferiority not shown	0.271
[MoP] C-Stem Cemented Stem Opera	356	3.79	1.64	[0.21 , 3.08]	Non-inferiority not shown	0.025
[MoP] C-Stem Cemented Stem Wroblewski Golf Ball	259	2.47	0.33	[-0.94 , 1.59]	Non-inferiority not shown	0.614
[MoP] CPT Elite Plus Ogee	408	3.16	1.01	[0.04 , 1.98]	Non-inferiority not shown	0.041
[MoP] CPT Trilogy	1,135	4.29	2.14	[1.42 , 2.86]	Inferior by ≥20%	<0.001
[MoP] CPT ZCA	1,233	3.71	1.57	[0.95 , 2.19]	Inferior by ≥20%	<0.001
[MoP] Charnley Cemented Stem Charnley Cemented Cup	1,751	3.52	1.38	[0.69 , 2.07]	Inferior by ≥20%	<0.001
[MoP] Charnley Cemented Stem Charnley Ogee	3,102	3.93	1.78	[1.23 , 2.34]	Inferior by ≥20%	<0.001
[MoP] Charnley Cemented Stem Charnley and Elite Plus LPW	2,284	2.43	0.29	[-0.25 , 0.83]	Non-inferiority not shown	0.297
[MoP] Charnley Cemented Stem Opera	254	3.99	1.85	[0.19 , 3.51]	Non-inferiority not shown	0.029
[MoP] Charnley Cemented Stem Wroblewski Golf Ball	419	2.42	0.28	[-0.81 , 1.36]	Non-inferiority not shown	0.619
[MoP] Corail Duraloc Cementless Cup	1,200	5.37	3.22	[2.31 , 4.14]	Inferior by ≥100%	<0.001
[MoP] Corail Pinnacle	1,681	3.05	0.91	[0.45 , 1.36]	Inferior by ≥20%	<0.001
[MoP] Elite Plus Cemented Stem Elite Plus Ogee	351	2.03	-0.11	[-1.41 , 1.19]	Non-inferiority not shown	0.865
[MoP] Exeter V40 ABG II Cementless Cup	429	2.62	0.48	[-0.77 , 1.73]	Non-inferiority not shown	0.456
[MoP] Exeter V40 Cenator Cemented Cup	694	2.64	0.49	[-0.29 , 1.28]	Non-inferiority not shown	0.219
[MoP] Exeter V40 Charnley Ogee	561	1.62	-0.52	[-1.26 , 0.21]	Non-inferior	0.163
[MoP] Exeter V40 Charnley and Elite Plus LPW	404	2.44	0.30	[-0.53 , 1.12]	Non-inferiority not shown	0.484
[MoP] Exeter V40 Duraloc Cementless Cup	451	4.19	2.05	[0.62 , 3.48]	Inferior by ≥20%	0.005
[MoP] Exeter V40 Elite Plus Cemented Cup	725	1.44	-0.70	[-1.31 , -0.09]	Non-inferior	0.024
[MoP] Exeter V40 Exeter Contemporary Flanged	4,653	2.28	0.13	[-0.23 , 0.49]	Non-inferiority not shown	0.469

[MoP] Exeter V40 Exeter Contemporary Hooded	2,355	4.12	1.97	[1.45 , 2.49]	Inferior by ≥20%	<0.001
[MoP] Exeter V40 Exeter Duration	2,967	3.71	1.56	[1.05 , 2.07]	Inferior by ≥20%	<0.001
[MoP] Exeter V40 Opera	443	3.23	1.09	[-0.03 , 2.21]	Non-inferiority not shown	0.057
[MoP] Exeter V40 Reflection Cementless	393	4.18	2.03	[0.76 , 3.30]	Inferior by ≥20%	0.002
[MoP] Exeter V40 Trident	1,116	2.71	0.56	[0.05 , 1.08]	Non-inferiority not shown	0.031
[MoP] Exeter V40 Trilogy	1,538	2.49	0.35	[-0.17 , 0.87]	Non-inferiority not shown	0.191
[MoP] Exeter V40 Ultima Cemented Cup	498	3.27	1.12	[-0.03 , 2.27]	Non-inferiority not shown	0.056
[MoP] Furlong Cemented Stem JRI Cemented Cup	584	3.23	1.09	[0.01 , 2.17]	Non-inferiority not shown	0.048
[MoP] Furlong HAC Stem CSF	1,697	4.41	2.27	[1.61 , 2.92]	Inferior by ≥20%	<0.001
[MoP] Muller-Biomet Apollo	371	2.67	0.53	[-0.56 , 1.62]	Non-inferiority not shown	0.345
[MoP] Omnifit Cemented Stem ODC	458	3.89	1.74	[0.35 , 3.14]	Non-inferiority not shown	0.014
[MoP] SP II Cemented Stem Interplanta	258	5.45	3.31	[1.32 , 5.30]	Inferior by ≥20%	0.001
[MoP] Stanmore Modular Stem Stanmore-Arcom Cup	669	2.52	0.38	[-0.29 , 1.04]	Non-inferiority not shown	0.264
[MoP] Synergy Cementless Stem Reflection Cementless	322	2.38	0.24	[-0.80 , 1.28]	Non-inferiority not shown	0.651
[MoP] Versys Cementless Stem Trilogy	464	5.47	3.32	[1.77 , 4.87]	Inferior by ≥20%	<0.001

Supplemental table 2a: Difference in Kaplan-Meier failure estimate between a contemporary reference and implants with at least 250 at risk at 3 years since primary in females

Stem/cup brand	Number at risk	Cumulative failure (%)	Difference in failure (%)	95% CI	Equivalence status	p-value
[CoP] MS-30 Original ME Muller Low Profile Cup	1,096	0.41	[REFERENCE]			
[CoC] ABG II Monolithic Cementless Stem ABG II Cementless Cup	502	1.36	0.96	[-0.11 , 2.02]	Non-inferiority not shown	0.079
[CoC] ABG II Monolithic Cementless Stem Trident	465	2.24	1.84	[0.48 , 3.19]	Inferior by ≥100%	0.008
[CoC] Accolade Trident	3,662	1.78	1.37	[0.83 , 1.92]	Inferior by ≥100%	<0.001
[CoC] Bimetric Cementless Stem Exceed ABT	511	1.18	0.77	[-0.17 , 1.70]	Non-inferiority not shown	0.108
[CoC] C-Stem AMT Cemented Stem Pinnacle	487	1.06	0.65	[-0.22 , 1.52]	Non-inferiority not shown	0.143
[CoC] CPT Continuum	481	1.17	0.76	[-0.13 , 1.64]	Non-inferiority not shown	0.093
[CoC] CPT Trilogy AB	305	0.97	0.56	[-0.59 , 1.71]	Non-inferiority not shown	0.339
[CoC] Corail Delta TT	453	1.90	1.49	[0.44 , 2.54]	Inferior by ≥100%	0.005
[CoC] Corail DeltaMotion	696	1.22	0.81	[-0.02 , 1.65]	Non-inferiority not shown	0.056
[CoC] Corail Duraloc Option	580	1.68	1.27	[0.18 , 2.36]	Inferior by ≥20%	0.023
[CoC] Corail Pinnacle	14,431	1.67	1.26	[0.85 , 1.66]	Inferior by ≥100%	<0.001
[CoC] Corail Pinnacle Gription	292	2.28	1.87	[0.57 , 3.17]	Inferior by ≥100%	0.005
[CoC] Corail Trinity	256	0.21	-0.20	[-0.74 , 0.35]	Non-inferiority not shown	0.474
[CoC] Excia Cementless Plasmacup SC	471	1.52	1.11	[0.06 , 2.17]	Non-inferiority not shown	0.038
[CoC] Exeter V40 ABG II Cementless Cup	887	0.40	-0.01	[-0.54 , 0.52]	Non-inferiority not shown	0.964
[CoC] Exeter V40 Trident	5,950	0.91	0.51	[0.08 , 0.93]	Non-inferiority not shown	0.020
[CoC] Furlong Evolution Cementless Furlong HAC CSF Plus	323	0.90	0.49	[-0.17 , 1.16]	Non-inferiority not shown	0.147
[CoC] Furlong HAC Stem CSF	881	1.54	1.13	[0.26 , 2.01]	Inferior by ≥20%	0.011
[CoC] Furlong HAC Stem Furlong HAC CSF Plus	4,954	1.49	1.08	[0.62 , 1.54]	Inferior by ≥100%	<0.001
[CoC] M/L Taper Cementless Continuum	689	1.52	1.11	[0.24 , 1.98]	Inferior by ≥20%	0.012
[CoC] Metafix Stem Trinity	469	1.43	1.03	[0.13 , 1.93]	Inferior by ≥20%	0.025
[CoC] Omnifit Cementless Stem Trident	267	2.20	1.79	[0.01 , 3.57]	Non-inferiority not shown	0.048
[CoC] Polarstem Cementless R3 Cementless	460	1.10	0.70	[-0.19 , 1.59]	Non-inferiority not shown	0.125
[CoC] S-Rom Cementless Stem Pinnacle	327	2.46	2.06	[0.50 , 3.61]	Inferior by ≥100%	0.010
[CoC] SL-Plus Cementless Stem EP-Fit Plus	602	1.75	1.34	[0.26 , 2.43]	Inferior by ≥20%	0.015
[CoC] SPS Modular April - Ceramic	302	2.39	1.98	[0.31 , 3.66]	Inferior by ≥20%	0.020
[CoC] Summit Cementless Stem Pinnacle	269	1.14	0.73	[-0.44 , 1.90]	Non-inferiority not shown	0.223
[CoC] Taperloc Cementless Stem Exceed ABT	3,728	1.43	1.02	[0.54 , 1.50]	Inferior by ≥100%	<0.001
[CoC] miniHip Trinity	441	1.11	0.70	[-0.19 , 1.60]	Non-inferiority not shown	0.125
[CoP] Accolade Trident	1,760	1.39	0.98	[0.40 , 1.56]	Inferior by ≥20%	0.001
[CoP] C-Stem Cemented Stem Elite Plus Ogee	342	0.28	-0.12	[-0.79 , 0.54]	Non-inferiority not shown	0.716
[CoP] C-Stem Cemented Stem Marathon	626	1.01	0.60	[-0.13 , 1.33]	Non-inferiority not shown	0.108
[CoP] C-Stem Cemented Stem Opera	445	0.22	-0.19	[-0.75 , 0.38]	Non-inferiority not shown	0.515
[CoP] C-Stem Cemented Stem Wroblewski Golf Ball	517	0.53	0.12	[-0.57 , 0.82]	Non-inferiority not shown	0.732
[CoP] CPT Trilogy	871	1.24	0.84	[0.27 , 1.40]	Inferior by ≥20%	0.004
[CoP] Corail Charnley and Elite Plus LPW	376	2.14	1.73	[0.30 , 3.16]	Inferior by ≥20%	0.018
[CoP] Corail Elite Plus Cemented Cup	267	0.91	0.50	[-0.58 , 1.58]	Non-inferiority not shown	0.367
[CoP] Corail Elite Plus Ogee	268	1.67	1.26	[-0.23 , 2.76]	Non-inferiority not shown	0.098
[CoP] Corail Marathon	787	0.72	0.31	[-0.26 , 0.89]	Non-inferiority not shown	0.285

[CoP] Corail Pinnacle	4,637	1.27	0.86	[0.43 , 1.29]	Inferior by ≥100%	<0.001
[CoP] Corail Trilogy	437	0.87	0.46	[-0.46 , 1.39]	Non-inferiority not shown	0.324
[CoP] Exeter V40 Charnley and Elite Plus LPW	374	1.99	1.58	[0.30 , 2.86]	Inferior by ≥20%	0.015
[CoP] Exeter V40 Elite Plus Ogee	655	0.57	0.16	[-0.45 , 0.77]	Non-inferiority not shown	0.611
[CoP] Exeter V40 Exeter Contemporary Flanged	1,849	1.14	0.73	[0.18 , 1.28]	Inferior by ≥20%	0.009
[CoP] Exeter V40 Exeter Contemporary Hooded	482	1.83	1.42	[0.29 , 2.56]	Inferior by ≥20%	0.014
[CoP] Exeter V40 Exeter Duration	476	0.93	0.52	[-0.37 , 1.40]	Non-inferiority not shown	0.251
[CoP] Exeter V40 Exeter X3 Rimfit	1,264	0.76	0.35	[-0.14 , 0.84]	Non-inferiority not shown	0.164
[CoP] Exeter V40 Marathon	284	0.92	0.51	[-0.48 , 1.49]	Non-inferiority not shown	0.312
[CoP] Exeter V40 Pinnacle	292	1.12	0.71	[-0.20 , 1.62]	Non-inferiority not shown	0.127
[CoP] Exeter V40 Trident	2,940	0.93	0.52	[0.08 , 0.96]	Inferior by ≥20%	0.020
[CoP] Exeter V40 Trilogy	1,182	0.60	0.19	[-0.36 , 0.74]	Non-inferiority not shown	0.491
[CoP] Exeter V40 Tritanium	250	1.74	1.33	[0.12 , 2.55]	Inferior by ≥20%	0.032
[CoP] Furlong HAC Stem CSF	3,748	1.32	0.91	[0.41 , 1.41]	Inferior by ≥100%	<0.001
[CoP] Furlong HAC Stem Furlong HAC CSF Plus	840	2.36	1.95	[0.99 , 2.91]	Inferior by ≥100%	<0.001
[CoP] SL-Plus Cementless Stem Bicon-Plus	342	1.66	1.25	[-0.11 , 2.61]	Non-inferiority not shown	0.073
[CoP] SL-Plus Cementless Stem EP-Fit Plus	593	1.93	1.52	[0.38 , 2.66]	Inferior by ≥20%	0.009
[CoP] Stanmore Modular Stem Stanmore-Arcom Cup	262	1.58	1.17	[-0.25 , 2.59]	Non-inferiority not shown	0.106
[CoP] Taperloc Cementless Stem Exceed ABT	1,062	0.99	0.59	[0.01 , 1.16]	Non-inferiority not shown	0.046
[MoP] Accolade Trident	5,487	1.75	1.34	[0.86 , 1.82]	Inferior by ≥100%	<0.001
[MoP] Anthology R3 Cementless	729	1.46	1.05	[0.29 , 1.81]	Inferior by ≥20%	0.007
[MoP] C-Stem AMT Cemented Stem Charnley and Elite Plus LPW	1,437	0.91	0.50	[-0.07 , 1.07]	Non-inferiority not shown	0.085
[MoP] C-Stem AMT Cemented Stem Elite Plus Cemented Cup	438	0.61	0.20	[-0.57 , 0.97]	Non-inferiority not shown	0.614
[MoP] C-Stem AMT Cemented Stem Elite Plus Ogee	1,135	0.84	0.43	[-0.15 , 1.01]	Non-inferiority not shown	0.149
[MoP] C-Stem AMT Cemented Stem Marathon	751	0.88	0.47	[-0.10 , 1.04]	Non-inferiority not shown	0.108
[MoP] C-Stem AMT Cemented Stem Pinnacle	985	1.24	0.84	[0.25 , 1.42]	Inferior by ≥20%	0.005
[MoP] C-Stem Cemented Stem Charnley Ogee	597	1.48	1.07	[0.09 , 2.05]	Inferior by ≥20%	0.033
[MoP] C-Stem Cemented Stem Charnley and Elite Plus LPW	1,119	1.17	0.76	[0.05 , 1.46]	Non-inferiority not shown	0.035
[MoP] C-Stem Cemented Stem Duraloc Cementless Cup	420	1.59	1.18	[-0.04 , 2.41]	Non-inferiority not shown	0.058
[MoP] C-Stem Cemented Stem Elite Plus Cemented Cup	390	0.47	0.06	[-0.68 , 0.80]	Non-inferiority not shown	0.870
[MoP] C-Stem Cemented Stem Elite Plus Ogee	1,985	0.69	0.28	[-0.21 , 0.78]	Non-inferiority not shown	0.257
[MoP] C-Stem Cemented Stem Marathon	959	0.63	0.22	[-0.30 , 0.73]	Non-inferiority not shown	0.407
[MoP] C-Stem Cemented Stem Opera	836	0.68	0.27	[-0.38 , 0.91]	Non-inferiority not shown	0.417
[MoP] C-Stem Cemented Stem Pinnacle	301	1.59	1.19	[-0.13 , 2.50]	Non-inferiority not shown	0.077
[MoP] C-Stem Cemented Stem Wroblewski Golf Ball	625	0.74	0.33	[-0.41 , 1.07]	Non-inferiority not shown	0.379
[MoP] CCA Cemented Stem CCB Cup	793	0.62	0.21	[-0.40 , 0.82]	Non-inferiority not shown	0.501
[MoP] CLS Cementless Stem Allofit	336	1.93	1.52	[0.06 , 2.99]	Non-inferiority not shown	0.041
[MoP] CLS Cementless Stem Trilogy	321	3.16	2.75	[0.88 , 4.62]	Inferior by ≥100%	0.004
[MoP] CMK Modular Cemented Stem CMK Cemented Cup	319	0.30	-0.11	[-0.79 , 0.58]	Non-inferiority not shown	0.755
[MoP] CPCS Opera	804	0.95	0.55	[-0.20 , 1.30]	Non-inferiority not shown	0.153
[MoP] CPCS Polarcup Cementless	259	0.76	0.35	[-0.76 , 1.46]	Non-inferiority not shown	0.535
[MoP] CPCS Reflection Cemented	264	1.10	0.69	[-0.26 , 1.64]	Non-inferiority not shown	0.156
[MoP] CPS Plus Cenator Cemented Cup	268	0.37	-0.04	[-0.85 , 0.77]	Non-inferiority not shown	0.931
[MoP] CPS Plus Opera	327	1.17	0.76	[-0.43 , 1.96]	Non-inferiority not shown	0.211
[MoP] CPT Allofit	485	0.32	-0.09	[-0.66 , 0.48]	Non-inferiority not shown	0.752
[MoP] CPT Continuum	315	2.66	2.25	[1.17 , 3.33]	Inferior by ≥100%	<0.001

[MoP] CPT Elite Plus Ogee	1,550	1.24	0.83	[0.21 , 1.46]	Inferior by ≥20%	0.009
[MoP] CPT Exeter Contemporary Flanged	283	2.17	1.76	[0.31 , 3.21]	Inferior by ≥20%	0.017
[MoP] CPT Opera	268	1.06	0.66	[-0.59 , 1.91]	Non-inferiority not shown	0.303
[MoP] CPT Original ME Muller Low Profile Cup	600	1.30	0.89	[0.00 , 1.78]	Non-inferiority not shown	0.050
[MoP] CPT Pinnacle	511	1.59	1.19	[0.09 , 2.28]	Inferior by ≥20%	0.033
[MoP] CPT Trabecular Metal Modular Cementless Cup	645	1.72	1.32	[0.40 , 2.23]	Inferior by ≥20%	0.005
[MoP] CPT Trilogy	5,622	1.20	0.79	[0.35 , 1.22]	Inferior by ≥20%	<0.001
[MoP] CPT Trilogy IT	304	2.93	2.52	[1.48 , 3.57]	Inferior by ≥100%	<0.001
[MoP] CPT ZCA	5,377	1.34	0.93	[0.49 , 1.38]	Inferior by ≥100%	<0.001
[MoP] Centrament Chirulen	294	0.00	- -	[-.-- , -.--]	No failures to date	
[MoP] Charnley Cemented Stem Charnley Cemented Cup	2,552	0.97	0.56	[0.04 , 1.07]	Non-inferiority not shown	0.033
[MoP] Charnley Cemented Stem Charnley Ogee	5,409	1.08	0.67	[0.22 , 1.11]	Inferior by ≥20%	0.003
[MoP] Charnley Cemented Stem Charnley and Elite Plus LPW	4,125	0.61	0.20	[-0.22 , 0.63]	Non-inferiority not shown	0.352
[MoP] Charnley Cemented Stem Opera	823	0.70	0.29	[-0.37 , 0.96]	Non-inferiority not shown	0.385
[MoP] Charnley Cemented Stem Wroblewski Golf Ball	609	1.53	1.12	[0.11 , 2.12]	Inferior by ≥20%	0.029
[MoP] Charnley Modular Charnley and Elite Plus LPW	275	0.36	-0.05	[-0.84 , 0.75]	Non-inferiority not shown	0.908
[MoP] Corail Charnley and Elite Plus LPW	543	0.83	0.42	[-0.33 , 1.17]	Non-inferiority not shown	0.274
[MoP] Corail Duraloc Cementless Cup	2,139	1.30	0.89	[0.30 , 1.48]	Inferior by ≥20%	0.003
[MoP] Corail Elite Plus Cemented Cup	629	0.42	0.02	[-0.58 , 0.62]	Non-inferiority not shown	0.955
[MoP] Corail Elite Plus Ogee	937	1.08	0.67	[-0.01 , 1.36]	Non-inferiority not shown	0.055
[MoP] Corail Exeter Contemporary Flanged	376	0.95	0.54	[-0.37 , 1.45]	Non-inferiority not shown	0.246
[MoP] Corail Marathon	1,873	0.80	0.39	[-0.08 , 0.87]	Non-inferiority not shown	0.107
[MoP] Corail Pinnacle	17,132	1.26	0.85	[0.47 , 1.23]	Inferior by ≥100%	<0.001
[MoP] Corail Trident	385	1.01	0.60	[-0.23 , 1.43]	Non-inferiority not shown	0.154
[MoP] Corail Trilogy	1,066	0.90	0.50	[-0.15 , 1.14]	Non-inferiority not shown	0.129
[MoP] Elite Plus Cemented Stem Elite Plus Ogee	402	0.49	0.08	[-0.68 , 0.84]	Non-inferiority not shown	0.842
[MoP] Exeter V40 ABG II Cementless Cup	477	0.80	0.39	[-0.47 , 1.25]	Non-inferiority not shown	0.373
[MoP] Exeter V40 Cenator Cemented Cup	1,425	1.45	1.04	[0.35 , 1.73]	Inferior by ≥20%	0.003
[MoP] Exeter V40 Charnley Ogee	1,068	1.00	0.59	[-0.08 , 1.26]	Non-inferiority not shown	0.083
[MoP] Exeter V40 Charnley and Elite Plus LPW	1,811	1.31	0.90	[0.31 , 1.50]	Inferior by ≥20%	0.003
[MoP] Exeter V40 Duraloc Cementless Cup	708	1.10	0.69	[-0.15 , 1.53]	Non-inferiority not shown	0.106
[MoP] Exeter V40 EP-Fit Plus	333	1.17	0.77	[-0.43 , 1.96]	Non-inferiority not shown	0.210
[MoP] Exeter V40 Elite Plus Cemented Cup	2,389	0.45	0.04	[-0.39 , 0.48]	Non-inferiority not shown	0.842
[MoP] Exeter V40 Elite Plus Ogee	10,898	0.70	0.29	[-0.10 , 0.67]	Non-inferiority not shown	0.142
[MoP] Exeter V40 Exceed	333	0.85	0.44	[-0.58 , 1.46]	Non-inferiority not shown	0.397
[MoP] Exeter V40 Exceed ABT	284	0.94	0.53	[-0.45 , 1.52]	Non-inferiority not shown	0.290
[MoP] Exeter V40 Exeter Contemporary Flanged	27,147	0.71	0.30	[-0.07 , 0.67]	Non-inferiority not shown	0.113
[MoP] Exeter V40 Exeter Contemporary Hooded	10,906	1.39	0.98	[0.58 , 1.39]	Inferior by ≥100%	<0.001
[MoP] Exeter V40 Exeter Duration	8,897	1.05	0.64	[0.23 , 1.05]	Inferior by ≥20%	0.002
[MoP] Exeter V40 Exeter X3 Rimfit	3,408	0.73	0.32	[-0.09 , 0.73]	Non-inferiority not shown	0.123
[MoP] Exeter V40 Furlong HAC CSF Plus	340	0.17	-0.24	[-0.73 , 0.25]	Non-inferiority not shown	0.333
[MoP] Exeter V40 Marathon	884	0.88	0.47	[-0.12 , 1.06]	Non-inferiority not shown	0.119
[MoP] Exeter V40 Opera	1,660	0.72	0.31	[-0.22 , 0.84]	Non-inferiority not shown	0.248
[MoP] Exeter V40 Pinnacle	1,807	0.97	0.56	[0.05 , 1.07]	Non-inferiority not shown	0.033
[MoP] Exeter V40 R3 Cementless	363	0.90	0.49	[-0.27 , 1.26]	Non-inferiority not shown	0.205
[MoP] Exeter V40 Reflection Cementless	1,308	0.73	0.32	[-0.25 , 0.90]	Non-inferiority not shown	0.273

[MoP] Exeter V40 Trabecular Metal Modular Cementless Cup	286	1.55	1.14	[-0.14 , 2.42]	Non-inferiority not shown	0.081
[MoP] Exeter V40 Trident	11,298	0.97	0.57	[0.18 , 0.95]	Inferior by ≥20%	0.004
[MoP] Exeter V40 Trilogy	5,145	0.83	0.43	[0.00 , 0.85]	Non-inferiority not shown	0.050
[MoP] Exeter V40 Tritanium	282	1.60	1.19	[0.12 , 2.25]	Inferior by ≥20%	0.029
[MoP] Exeter V40 Ultima Cemented Cup	777	1.10	0.69	[-0.11 , 1.49]	Non-inferiority not shown	0.090
[MoP] Furlong Cemented Stem JRI Cemented Cup	1,036	1.43	1.02	[0.24 , 1.81]	Inferior by ≥20%	0.010
[MoP] Furlong HAC Stem CSF	4,074	2.18	1.77	[1.22 , 2.33]	Inferior by ≥100%	<0.001
[MoP] Furlong HAC Stem Furlong HAC CSF Plus	1,829	2.12	1.71	[1.07 , 2.36]	Inferior by ≥100%	<0.001
[MoP] Furlong HAC Stem Furlong Threaded	311	1.83	1.42	[-0.07 , 2.92]	Non-inferiority not shown	0.062
[MoP] M/L Taper Cementless Continuum	381	1.72	1.31	[0.24 , 2.37]	Inferior by ≥20%	0.016
[MoP] M/L Taper Cementless Trilogy	281	2.08	1.68	[0.34 , 3.01]	Inferior by ≥20%	0.014
[MoP] MS-30 Original ME Muller Low Profile Cup	522	0.28	-0.13	[-0.66 , 0.40]	Non-inferiority not shown	0.637
[MoP] Muller Straight Stem Centerpulse Muller	328	0.88	0.47	[-0.58 , 1.52]	Non-inferiority not shown	0.382
[MoP] Muller Straight Stem Original ME Muller Low Profile Cup	999	0.92	0.52	[-0.12 , 1.15]	Non-inferiority not shown	0.112
[MoP] Muller-Biomet Apollo	1,201	1.32	0.91	[0.19 , 1.63]	Inferior by ≥20%	0.013
[MoP] Muller-Biomet Original ME Muller Low Profile Cup	707	1.32	0.91	[0.02 , 1.80]	Non-inferiority not shown	0.044
[MoP] Omnifit Cemented Stem ODC	614	1.56	1.15	[0.13 , 2.18]	Inferior by ≥20%	0.027
[MoP] Omnifit Cementless Stem Trident	251	2.95	2.55	[0.50 , 4.60]	Inferior by ≥100%	0.015
[MoP] P10 Muller Original ME Muller Low Profile Cup	268	1.03	0.62	[-0.59 , 1.83]	Non-inferiority not shown	0.317
[MoP] Polarstem Cementless R3 Cementless	592	0.73	0.33	[-0.19 , 0.85]	Non-inferiority not shown	0.217
[MoP] SL-Plus Cementless Stem EP-Fit Plus	1,198	2.40	2.00	[1.14 , 2.85]	Inferior by ≥100%	<0.001
[MoP] SP II Cemented Stem Interplanta	444	1.84	1.43	[0.19 , 2.68]	Inferior by ≥20%	0.024
[MoP] Stanmore Modular Stem Elite Plus Cemented Cup	367	0.26	-0.15	[-0.77 , 0.48]	Non-inferiority not shown	0.647
[MoP] Stanmore Modular Stem SHP Cup	664	0.75	0.34	[-0.36 , 1.04]	Non-inferiority not shown	0.339
[MoP] Stanmore Modular Stem Stanmore-Arcom Cup	2,729	1.12	0.71	[0.19 , 1.23]	Inferior by ≥20%	0.007
[MoP] Synergy Cementless Stem R3 Cementless	554	1.00	0.59	[-0.08 , 1.26]	Non-inferiority not shown	0.082
[MoP] Synergy Cementless Stem Reflection Cementless	859	0.68	0.27	[-0.38 , 0.92]	Non-inferiority not shown	0.416
[MoP] Taperfit Cemented Stem Atlas IIIp	327	1.14	0.73	[-0.33 , 1.79]	Non-inferiority not shown	0.177
[MoP] Taperloc Cementless Stem Exceed	318	1.20	0.79	[-0.43 , 2.01]	Non-inferiority not shown	0.204
[MoP] Taperloc Cementless Stem Exceed ABT	2,287	1.98	1.57	[0.98 , 2.16]	Inferior by ≥100%	<0.001
[MoP] Versys Cementless Stem Trilogy	492	3.79	3.38	[1.71 , 5.05]	Inferior by ≥100%	<0.001

Supplemental table 2b: Difference in Kaplan-Meier failure estimate between a contemporary reference and implants with at least 250 at risk at 5 years since primary in females

Stem/cup brand	Number at risk	Cumulative failure (%)	Difference in failure (%)	95% CI	Equivalence status	p-value
[MoP] Exeter V40 Elite Plus Cemented Cup	1,769	0.54	[REFERENCE]			
[CoC] ABG II Monolithic Cementless Stem ABG II Cementless Cup	485	1.76	1.22	[0.05 , 2.40]	Non-inferiority not shown	0.041
[CoC] ABG II Monolithic Cementless Stem Trident	372	2.95	2.41	[0.86 , 3.96]	Inferior by ≥100%	0.002
[CoC] Accolade Trident	2,906	2.56	2.02	[1.44 , 2.59]	Inferior by ≥100%	<0.001
[CoC] Bimetric Cementless Stem Exceed ABT	369	1.84	1.30	[0.13 , 2.48]	Inferior by ≥20%	0.030
[CoC] C-Stem AMT Cemented Stem Pinnacle	268	1.80	1.26	[0.08 , 2.44]	Non-inferiority not shown	0.037
[CoC] CPT Trilogy AB	278	1.64	1.10	[-0.35 , 2.55]	Non-inferiority not shown	0.138
[CoC] Corail DeltaMotion	373	1.47	0.93	[-0.01 , 1.86]	Non-inferiority not shown	0.051
[CoC] Corail Duraloc Option	566	2.70	2.16	[0.83 , 3.49]	Inferior by ≥100%	0.001
[CoC] Corail Pinnacle	9,650	2.16	1.62	[1.26 , 1.97]	Inferior by ≥100%	<0.001
[CoC] Excia Cementless Plasmacup SC	266	1.52	0.98	[-0.04 , 2.01]	Non-inferiority not shown	0.061
[CoC] Exeter V40 ABG II Cementless Cup	760	1.10	0.56	[-0.17 , 1.30]	Non-inferiority not shown	0.133
[CoC] Exeter V40 Trident	4,852	1.27	0.74	[0.35 , 1.13]	Inferior by ≥20%	<0.001
[CoC] Furlong HAC Stem CSF	839	2.11	1.57	[0.59 , 2.55]	Inferior by ≥100%	0.002
[CoC] Furlong HAC Stem Furlong HAC CSF Plus	3,077	1.78	1.24	[0.81 , 1.68]	Inferior by ≥100%	<0.001
[CoC] M/L Taper Cementless Continuum	304	1.68	1.14	[0.25 , 2.04]	Inferior by ≥20%	0.012
[CoC] S-Rom Cementless Stem Pinnacle	251	3.21	2.67	[0.83 , 4.52]	Inferior by ≥100%	0.004
[CoC] SL-Plus Cementless Stem EP-Fit Plus	565	3.43	2.89	[1.42 , 4.36]	Inferior by ≥100%	<0.001
[CoC] Taperloc Cementless Stem Exceed ABT	2,243	1.79	1.25	[0.78 , 1.72]	Inferior by ≥100%	<0.001
[CoP] Accolade Trident	828	1.84	1.31	[0.66 , 1.95]	Inferior by ≥100%	<0.001
[CoP] C-Stem Cemented Stem Elite Plus Ogee	262	0.62	0.08	[-0.82 , 0.98]	Non-inferiority not shown	0.860
[CoP] C-Stem Cemented Stem Marathon	297	1.21	0.67	[-0.13 , 1.48]	Non-inferiority not shown	0.099
[CoP] C-Stem Cemented Stem Opera	374	1.20	0.66	[-0.42 , 1.74]	Non-inferiority not shown	0.232
[CoP] C-Stem Cemented Stem Wroblewski Golf Ball	417	0.73	0.19	[-0.57 , 0.95]	Non-inferiority not shown	0.628
[CoP] Corail Charnley and Elite Plus LPW	321	2.42	1.88	[0.37 , 3.39]	Inferior by ≥20%	0.015
[CoP] Corail Marathon	333	1.04	0.50	[-0.19 , 1.19]	Non-inferiority not shown	0.153
[CoP] Corail Pinnacle	2,442	1.71	1.17	[0.75 , 1.59]	Inferior by ≥100%	<0.001
[CoP] Corail Trilogy	397	1.10	0.56	[-0.44 , 1.56]	Non-inferiority not shown	0.269
[CoP] Exeter V40 Elite Plus Ogee	478	1.06	0.52	[-0.27 , 1.31]	Non-inferiority not shown	0.198
[CoP] Exeter V40 Exeter Contemporary Flanged	1,225	1.60	1.06	[0.45 , 1.66]	Inferior by ≥20%	0.001
[CoP] Exeter V40 Exeter Contemporary Hooded	358	2.31	1.77	[0.48 , 3.06]	Inferior by ≥20%	0.007
[CoP] Exeter V40 Exeter Duration	405	0.93	0.39	[-0.47 , 1.24]	Non-inferiority not shown	0.374
[CoP] Exeter V40 Trident	1,446	1.28	0.75	[0.29 , 1.20]	Inferior by ≥20%	0.001
[CoP] Exeter V40 Trilogy	997	0.86	0.32	[-0.26 , 0.89]	Non-inferiority not shown	0.278
[CoP] Furlong HAC Stem CSF	3,217	1.83	1.29	[0.79 , 1.79]	Inferior by ≥100%	<0.001
[CoP] Furlong HAC Stem Furlong HAC CSF Plus	508	2.55	2.01	[1.01 , 3.01]	Inferior by ≥100%	<0.001
[CoP] MS-30 Original ME Muller Low Profile Cup	796	0.64	0.10	[-0.45 , 0.65]	Non-inferiority not shown	0.725
[CoP] SL-Plus Cementless Stem Bicon-Plus	306	3.49	2.95	[0.99 , 4.91]	Inferior by ≥100%	0.003
[CoP] SL-Plus Cementless Stem EP-Fit Plus	499	3.49	2.95	[1.46 , 4.45]	Inferior by ≥100%	<0.001
[CoP] Taperloc Cementless Stem Exceed ABT	466	1.23	0.69	[0.07 , 1.31]	Non-inferiority not shown	0.029

[MoP] Accolade Trident	3,698	2.36	1.82	[1.34 , 2.29]	Inferior by ≥100%	<0.001
[MoP] C-Stem AMT Cemented Stem Charnley and Elite Plus LPW	954	1.29	0.75	[0.13 , 1.37]	Inferior by ≥20%	0.017
[MoP] C-Stem AMT Cemented Stem Elite Plus Cemented Cup	313	0.85	0.31	[-0.57 , 1.18]	Non-inferiority not shown	0.488
[MoP] C-Stem AMT Cemented Stem Elite Plus Ogee	666	1.18	0.64	[-0.02 , 1.30]	Non-inferiority not shown	0.058
[MoP] C-Stem AMT Cemented Stem Pinnacle	426	1.66	1.12	[0.40 , 1.83]	Inferior by ≥20%	0.002
[MoP] C-Stem Cemented Stem Charnley Ogee	480	2.25	1.71	[0.50 , 2.92]	Inferior by ≥20%	0.006
[MoP] C-Stem Cemented Stem Charnley and Elite Plus LPW	1,007	1.81	1.28	[0.46 , 2.09]	Inferior by ≥20%	0.002
[MoP] C-Stem Cemented Stem Duraloc Cementless Cup	388	2.33	1.79	[0.34 , 3.24]	Inferior by ≥20%	0.016
[MoP] C-Stem Cemented Stem Elite Plus Cemented Cup	357	0.47	-0.07	[-0.77 , 0.64]	Non-inferiority not shown	0.848
[MoP] C-Stem Cemented Stem Elite Plus Ogee	1,554	1.02	0.49	[-0.02 , 0.99]	Non-inferiority not shown	0.062
[MoP] C-Stem Cemented Stem Marathon	473	0.91	0.37	[-0.24 , 0.98]	Non-inferiority not shown	0.235
[MoP] C-Stem Cemented Stem Opera	626	0.95	0.41	[-0.30 , 1.13]	Non-inferiority not shown	0.259
[MoP] C-Stem Cemented Stem Wroblewski Golf Ball	488	1.61	1.07	[0.04 , 2.11]	Non-inferiority not shown	0.042
[MoP] CCA Cemented Stem CCB Cup	535	0.92	0.38	[-0.32 , 1.08]	Non-inferiority not shown	0.289
[MoP] CLS Cementless Stem Allofit	316	2.53	1.99	[0.33 , 3.64]	Inferior by ≥20%	0.019
[MoP] CMK Modular Cemented Stem CMK Cemented Cup	291	1.25	0.72	[-0.54 , 1.97]	Non-inferiority not shown	0.263
[MoP] CPCS Opera	586	1.63	1.09	[0.17 , 2.02]	Inferior by ≥20%	0.020
[MoP] CPS Plus Opera	309	1.78	1.25	[-0.20 , 2.69]	Non-inferiority not shown	0.090
[MoP] CPT Allofit	308	0.58	0.04	[-0.69 , 0.77]	Non-inferiority not shown	0.921
[MoP] CPT Elite Plus Ogee	1,115	1.60	1.06	[0.40 , 1.73]	Inferior by ≥20%	0.002
[MoP] CPT Opera	252	1.84	1.30	[-0.32 , 2.92]	Non-inferiority not shown	0.116
[MoP] CPT Original ME Muller Low Profile Cup	322	1.98	1.44	[0.28 , 2.60]	Inferior by ≥20%	0.015
[MoP] CPT Pinnacle	433	2.03	1.49	[0.27 , 2.72]	Inferior by ≥20%	0.017
[MoP] CPT Trabecular Metal Modular Cementless Cup	399	2.48	1.95	[0.79 , 3.10]	Inferior by ≥100%	0.001
[MoP] CPT Trilogy	3,903	2.10	1.56	[1.11 , 2.01]	Inferior by ≥100%	<0.001
[MoP] CPT ZCA	3,998	1.98	1.44	[1.00 , 1.88]	Inferior by ≥100%	<0.001
[MoP] Charnley Cemented Stem Charnley Cemented Cup	2,265	1.42	0.88	[0.35 , 1.41]	Inferior by ≥20%	0.001
[MoP] Charnley Cemented Stem Charnley Ogee	4,710	1.63	1.10	[0.67 , 1.53]	Inferior by ≥100%	<0.001
[MoP] Charnley Cemented Stem Charnley and Elite Plus LPW	3,628	1.00	0.46	[0.05 , 0.86]	Non-inferiority not shown	0.027
[MoP] Charnley Cemented Stem Opera	697	1.35	0.81	[-0.03 , 1.65]	Non-inferiority not shown	0.058
[MoP] Charnley Cemented Stem Wroblewski Golf Ball	487	1.73	1.19	[0.14 , 2.24]	Inferior by ≥20%	0.027
[MoP] Corail Charnley and Elite Plus LPW	332	1.29	0.75	[-0.21 , 1.71]	Non-inferiority not shown	0.126
[MoP] Corail Duraloc Cementless Cup	2,023	2.10	1.56	[0.90 , 2.22]	Inferior by ≥100%	<0.001
[MoP] Corail Elite Plus Cemented Cup	481	0.99	0.45	[-0.39 , 1.29]	Non-inferiority not shown	0.295
[MoP] Corail Elite Plus Ogee	678	1.65	1.11	[0.30 , 1.93]	Inferior by ≥20%	0.007
[MoP] Corail Marathon	921	0.93	0.40	[-0.06 , 0.85]	Non-inferiority not shown	0.089
[MoP] Corail Pinnacle	10,090	1.56	1.03	[0.71 , 1.35]	Inferior by ≥100%	<0.001
[MoP] Corail Trilogy	859	1.22	0.69	[-0.01 , 1.38]	Non-inferiority not shown	0.054
[MoP] Elite Plus Cemented Stem Elite Plus Ogee	362	1.26	0.72	[-0.41 , 1.86]	Non-inferiority not shown	0.211
[MoP] Exeter V40 ABG II Cementless Cup	458	1.01	0.47	[-0.45 , 1.40]	Non-inferiority not shown	0.315
[MoP] Exeter V40 Cenator Cemented Cup	1,153	2.12	1.58	[0.80 , 2.36]	Inferior by ≥100%	<0.001
[MoP] Exeter V40 Charnley Ogee	900	1.39	0.85	[0.12 , 1.59]	Inferior by ≥20%	0.023
[MoP] Exeter V40 Charnley and Elite Plus LPW	1,309	1.61	1.07	[0.46 , 1.68]	Inferior by ≥20%	0.001
[MoP] Exeter V40 Duraloc Cementless Cup	671	1.82	1.28	[0.26 , 2.30]	Inferior by ≥20%	0.014
[MoP] Exeter V40 EP-Fit Plus	312	2.98	2.44	[0.60 , 4.29]	Inferior by ≥100%	0.009
[MoP] Exeter V40 Elite Plus Ogee	8,315	1.02	0.48	[0.15 , 0.81]	Inferior by ≥20%	0.004

[MoP] Exeter V40 Exceed	250	0.85	0.31	[-0.68 , 1.30]	Non-inferiority not shown	0.541
[MoP] Exeter V40 Exeter Contemporary Flanged	17,934	1.04	0.50	[0.20 , 0.80]	Inferior by ≥20%	0.001
[MoP] Exeter V40 Exeter Contemporary Hooded	7,664	2.02	1.48	[1.10 , 1.85]	Inferior by ≥100%	<0.001
[MoP] Exeter V40 Exeter Duration	7,168	1.43	0.89	[0.53 , 1.25]	Inferior by ≥20%	<0.001
[MoP] Exeter V40 Exeter X3 Rimfit	550	0.92	0.38	[-0.02 , 0.79]	Non-inferiority not shown	0.066
[MoP] Exeter V40 Marathon	364	1.25	0.71	[-0.04 , 1.46]	Non-inferiority not shown	0.064
[MoP] Exeter V40 Opera	1,221	0.98	0.44	[-0.10 , 0.98]	Non-inferiority not shown	0.110
[MoP] Exeter V40 Pinnacle	1,075	1.24	0.70	[0.17 , 1.24]	Inferior by ≥20%	0.010
[MoP] Exeter V40 Reflection Cementless	1,164	1.05	0.51	[-0.10 , 1.13]	Non-inferiority not shown	0.100
[MoP] Exeter V40 Trident	6,743	1.30	0.76	[0.43 , 1.09]	Inferior by ≥20%	<0.001
[MoP] Exeter V40 Trilogy	4,060	1.24	0.70	[0.30 , 1.10]	Inferior by ≥20%	0.001
[MoP] Exeter V40 Ultima Cemented Cup	720	1.90	1.36	[0.37 , 2.35]	Inferior by ≥20%	0.007
[MoP] Furlong Cemented Stem JRI Cemented Cup	892	1.74	1.20	[0.38 , 2.03]	Inferior by ≥20%	0.004
[MoP] Furlong HAC Stem CSF	3,340	2.50	1.96	[1.43 , 2.50]	Inferior by ≥100%	<0.001
[MoP] Furlong HAC Stem Furlong HAC CSF Plus	1,122	2.75	2.21	[1.48 , 2.94]	Inferior by ≥100%	<0.001
[MoP] Furlong HAC Stem Furlong Threaded	269	2.84	2.30	[0.45 , 4.15]	Inferior by ≥20%	0.015
[MoP] MS-30 Original ME Muller Low Profile Cup	366	0.98	0.44	[-0.48 , 1.36]	Non-inferiority not shown	0.351
[MoP] Muller Straight Stem Centerpulse Muller	291	1.85	1.31	[-0.18 , 2.81]	Non-inferiority not shown	0.085
[MoP] Muller Straight Stem Original ME Muller Low Profile Cup	718	1.26	0.72	[0.02 , 1.42]	Non-inferiority not shown	0.044
[MoP] Muller-Biomet Apollo	972	1.50	0.97	[0.24 , 1.69]	Inferior by ≥20%	0.009
[MoP] Muller-Biomet Original ME Muller Low Profile Cup	442	1.79	1.25	[0.25 , 2.26]	Inferior by ≥20%	0.015
[MoP] Omnifit Cemented Stem ODC	559	2.23	1.69	[0.50 , 2.87]	Inferior by ≥20%	0.005
[MoP] SL-Plus Cementless Stem EP-Fit Plus	980	3.12	2.58	[1.62 , 3.54]	Inferior by ≥100%	<0.001
[MoP] SP II Cemented Stem Interplanta	392	2.33	1.79	[0.40 , 3.18]	Inferior by ≥20%	0.012
[MoP] Stanmore Modular Stem Elite Plus Cemented Cup	273	1.19	0.65	[-0.55 , 1.85]	Non-inferiority not shown	0.287
[MoP] Stanmore Modular Stem SHP Cup	483	0.75	0.21	[-0.45 , 0.87]	Non-inferiority not shown	0.534
[MoP] Stanmore Modular Stem Stanmore-Arcom Cup	2,115	1.69	1.15	[0.60 , 1.70]	Inferior by ≥100%	<0.001
[MoP] Synergy Cementless Stem Reflection Cementless	825	0.91	0.38	[-0.31 , 1.06]	Non-inferiority not shown	0.284
[MoP] Taperloc Cementless Stem Exceed	277	1.84	1.30	[-0.18 , 2.79]	Non-inferiority not shown	0.086
[MoP] Taperloc Cementless Stem Exceed ABT	1,136	2.32	1.78	[1.17 , 2.38]	Inferior by ≥100%	<0.001
[MoP] Versys Cementless Stem Trilogy	462	4.99	4.45	[2.56 , 6.34]	Inferior by ≥100%	<0.001

Supplemental table 2c: Difference in Kaplan-Meier failure estimate between a contemporary reference and implants with at least 250 at risk at 7 years since primary in females

Stem/cup brand	Number at risk	Cumulative failure (%)	Difference in failure (%)	95% CI	Equivalence status	p-value
[MoP] Exeter V40 Elite Plus Cemented Cup	1,165	0.76	[REFERENCE]			
[CoC] ABG II Monolithic Cementless Stem ABG II Cementless Cup	441	2.60	1.84	[0.40 , 3.29]	Inferior by ≥20%	0.012
[CoC] ABG II Monolithic Cementless Stem Trident	341	3.21	2.45	[0.80 , 4.10]	Inferior by ≥100%	0.004
[CoC] Accolade Trident	1,744	3.15	2.39	[1.69 , 3.09]	Inferior by ≥100%	<0.001
[CoC] Corail Duraloc Option	466	3.09	2.33	[0.88 , 3.79]	Inferior by ≥100%	0.002
[CoC] Corail Pinnacle	4,397	2.64	1.88	[1.42 , 2.34]	Inferior by ≥100%	<0.001
[CoC] Exeter V40 ABG II Cementless Cup	615	1.55	0.79	[-0.13 , 1.71]	Non-inferiority not shown	0.094
[CoC] Exeter V40 Trident	3,449	1.85	1.09	[0.58 , 1.61]	Inferior by ≥20%	<0.001
[CoC] Furlong HAC Stem CSF	776	3.08	2.32	[1.12 , 3.53]	Inferior by ≥100%	<0.001
[CoC] Furlong HAC Stem Furlong HAC CSF Plus	1,170	2.19	1.43	[0.87 , 2.00]	Inferior by ≥100%	<0.001
[CoC] SL-Plus Cementless Stem EP-Fit Plus	486	3.61	2.85	[1.32 , 4.38]	Inferior by ≥100%	<0.001
[CoC] Taperloc Cementless Stem Exceed ABT	981	1.93	1.17	[0.60 , 1.74]	Inferior by ≥20%	<0.001
[CoP] Accolade Trident	345	1.98	1.22	[0.48 , 1.96]	Inferior by ≥20%	0.001
[CoP] C-Stem Cemented Stem Opera	274	1.84	1.08	[-0.34 , 2.49]	Non-inferiority not shown	0.135
[CoP] C-Stem Cemented Stem Wroblewski Golf Ball	347	1.80	1.05	[-0.27 , 2.37]	Non-inferiority not shown	0.120
[CoP] Corail Pinnacle	1,028	2.07	1.32	[0.74 , 1.89]	Inferior by ≥20%	<0.001
[CoP] Corail Trilogy	310	1.10	0.34	[-0.69 , 1.37]	Non-inferiority not shown	0.513
[CoP] Exeter V40 Elite Plus Ogee	304	1.29	0.54	[-0.41 , 1.49]	Non-inferiority not shown	0.268
[CoP] Exeter V40 Exeter Contemporary Flanged	694	1.78	1.02	[0.32 , 1.73]	Inferior by ≥20%	0.004
[CoP] Exeter V40 Exeter Duration	309	2.02	1.26	[-0.12 , 2.65]	Non-inferiority not shown	0.074
[CoP] Exeter V40 Trident	656	1.46	0.71	[0.13 , 1.28]	Non-inferiority not shown	0.017
[CoP] Exeter V40 Trilogy	723	1.19	0.43	[-0.30 , 1.16]	Non-inferiority not shown	0.249
[CoP] Furlong HAC Stem CSF	2,638	2.37	1.61	[0.99 , 2.22]	Inferior by ≥100%	<0.001
[CoP] MS-30 Original ME Muller Low Profile Cup	489	0.64	-0.12	[-0.72 , 0.48]	Non-inferiority not shown	0.696
[CoP] SL-Plus Cementless Stem EP-Fit Plus	364	4.38	3.63	[1.89 , 5.37]	Inferior by ≥100%	<0.001
[MoP] Accolade Trident	1,704	3.00	2.25	[1.63 , 2.86]	Inferior by ≥100%	<0.001
[MoP] C-Stem AMT Cemented Stem Charnley and Elite Plus LPW	534	1.65	0.89	[0.06 , 1.71]	Non-inferiority not shown	0.035
[MoP] C-Stem AMT Cemented Stem Elite Plus Ogee	378	1.98	1.22	[0.17 , 2.28]	Inferior by ≥20%	0.023
[MoP] C-Stem Cemented Stem Charnley Ogee	384	2.49	1.73	[0.41 , 3.05]	Inferior by ≥20%	0.010
[MoP] C-Stem Cemented Stem Charnley and Elite Plus LPW	915	2.34	1.58	[0.61 , 2.54]	Inferior by ≥20%	0.001
[MoP] C-Stem Cemented Stem Duraloc Cementless Cup	300	3.44	2.68	[0.86 , 4.49]	Inferior by ≥100%	0.004
[MoP] C-Stem Cemented Stem Elite Plus Cemented Cup	295	1.05	0.30	[-0.80 , 1.39]	Non-inferiority not shown	0.598
[MoP] C-Stem Cemented Stem Elite Plus Ogee	1,198	1.54	0.79	[0.10 , 1.47]	Non-inferiority not shown	0.024
[MoP] C-Stem Cemented Stem Opera	463	1.66	0.90	[-0.12 , 1.93]	Non-inferiority not shown	0.084
[MoP] C-Stem Cemented Stem Wroblewski Golf Ball	333	2.18	1.42	[0.10 , 2.73]	Non-inferiority not shown	0.034
[MoP] CPS Plus Opera	279	2.13	1.37	[-0.24 , 2.97]	Non-inferiority not shown	0.095
[MoP] CPT Elite Plus Ogee	741	2.31	1.55	[0.65 , 2.45]	Inferior by ≥20%	0.001
[MoP] CPT Trilogy	2,474	2.68	1.92	[1.34 , 2.50]	Inferior by ≥100%	<0.001
[MoP] CPT ZCA	2,612	2.46	1.70	[1.15 , 2.26]	Inferior by ≥100%	<0.001
[MoP] Charnley Cemented Stem Charnley Cemented Cup	1,879	1.93	1.18	[0.52 , 1.83]	Inferior by ≥20%	<0.001

[MoP] Charnley Cemented Stem Charnley Ogee	3,746	1.93	1.18	[0.65 , 1.70]	Inferior by ≥20%	<0.001
[MoP] Charnley Cemented Stem Charnley and Elite Plus LPW	2,920	1.35	0.59	[0.08 , 1.11]	Non-inferiority not shown	0.024
[MoP] Charnley Cemented Stem Opera	496	2.12	1.37	[0.26 , 2.47]	Inferior by ≥20%	0.015
[MoP] Charnley Cemented Stem Wroblewski Golf Ball	404	1.73	0.97	[-0.11 , 2.05]	Non-inferiority not shown	0.079
[MoP] Corail Duraloc Cementless Cup	1,695	3.25	2.49	[1.65 , 3.34]	Inferior by ≥100%	<0.001
[MoP] Corail Elite Plus Cemented Cup	322	1.46	0.70	[-0.39 , 1.79]	Non-inferiority not shown	0.208
[MoP] Corail Elite Plus Ogee	456	1.85	1.09	[0.16 , 2.03]	Inferior by ≥20%	0.022
[MoP] Corail Pinnacle	4,975	2.01	1.25	[0.82 , 1.68]	Inferior by ≥100%	<0.001
[MoP] Corail Trilogy	533	1.87	1.11	[0.14 , 2.08]	Non-inferiority not shown	0.025
[MoP] Elite Plus Cemented Stem Elite Plus Ogee	329	1.26	0.50	[-0.66 , 1.67]	Non-inferiority not shown	0.394
[MoP] Exeter V40 ABG II Cementless Cup	416	1.25	0.49	[-0.57 , 1.55]	Non-inferiority not shown	0.365
[MoP] Exeter V40 Cenator Cemented Cup	860	2.22	1.46	[0.62 , 2.30]	Inferior by ≥20%	0.001
[MoP] Exeter V40 Charnley Ogee	721	1.39	0.64	[-0.14 , 1.41]	Non-inferiority not shown	0.108
[MoP] Exeter V40 Charnley and Elite Plus LPW	788	1.99	1.23	[0.48 , 1.99]	Inferior by ≥20%	0.001
[MoP] Exeter V40 Duraloc Cementless Cup	507	2.12	1.37	[0.24 , 2.49]	Inferior by ≥20%	0.017
[MoP] Exeter V40 EP-Fit Plus	268	3.64	2.88	[0.82 , 4.94]	Inferior by ≥100%	0.006
[MoP] Exeter V40 Elite Plus Ogee	5,848	1.40	0.65	[0.21 , 1.08]	Inferior by ≥20%	0.004
[MoP] Exeter V40 Exeter Contemporary Flanged	11,143	1.36	0.60	[0.20 , 1.00]	Inferior by ≥20%	0.003
[MoP] Exeter V40 Exeter Contemporary Hooded	4,810	2.77	2.01	[1.52 , 2.51]	Inferior by ≥100%	<0.001
[MoP] Exeter V40 Exeter Duration	5,153	2.05	1.29	[0.81 , 1.77]	Inferior by ≥100%	<0.001
[MoP] Exeter V40 Opera	805	1.35	0.59	[-0.10 , 1.29]	Non-inferiority not shown	0.095
[MoP] Exeter V40 Pinnacle	507	1.45	0.69	[0.04 , 1.35]	Non-inferiority not shown	0.038
[MoP] Exeter V40 Reflection Cementless	877	1.73	0.97	[0.14 , 1.80]	Non-inferiority not shown	0.022
[MoP] Exeter V40 Trident	3,625	1.74	0.99	[0.53 , 1.44]	Inferior by ≥20%	<0.001
[MoP] Exeter V40 Trilogy	2,694	1.53	0.77	[0.27 , 1.28]	Inferior by ≥20%	0.003
[MoP] Exeter V40 Ultima Cemented Cup	635	2.04	1.28	[0.22 , 2.33]	Inferior by ≥20%	0.018
[MoP] Furlong Cemented Stem JRI Cemented Cup	730	2.09	1.33	[0.39 , 2.28]	Inferior by ≥20%	0.006
[MoP] Furlong HAC Stem CSF	2,586	3.04	2.28	[1.64 , 2.92]	Inferior by ≥100%	<0.001
[MoP] Furlong HAC Stem Furlong HAC CSF Plus	469	3.16	2.40	[1.53 , 3.27]	Inferior by ≥100%	<0.001
[MoP] Muller Straight Stem Original ME Muller Low Profile Cup	474	2.09	1.33	[0.29 , 2.37]	Inferior by ≥20%	0.012
[MoP] Muller-Biomet Apollo	714	1.85	1.09	[0.23 , 1.95]	Inferior by ≥20%	0.013
[MoP] Omnifit Cemented Stem ODC	495	3.36	2.60	[1.10 , 4.10]	Inferior by ≥100%	0.001
[MoP] SL-Plus Cementless Stem EP-Fit Plus	750	3.80	3.04	[1.92 , 4.16]	Inferior by ≥100%	<0.001
[MoP] SP II Cemented Stem Interplanta	326	2.88	2.12	[0.52 , 3.72]	Inferior by ≥20%	0.009
[MoP] Stanmore Modular Stem SHP Cup	352	1.24	0.48	[-0.50 , 1.46]	Non-inferiority not shown	0.338
[MoP] Stanmore Modular Stem Stanmore-Arcom Cup	1,379	2.01	1.25	[0.59 , 1.90]	Inferior by ≥20%	<0.001
[MoP] Synergy Cementless Stem Reflection Cementless	607	1.33	0.57	[-0.30 , 1.44]	Non-inferiority not shown	0.196
[MoP] Taperloc Cementless Stem Exceed ABT	346	2.99	2.24	[1.34 , 3.13]	Inferior by ≥100%	<0.001
[MoP] Versys Cementless Stem Trilogy	418	4.99	4.23	[2.32 , 6.14]	Inferior by ≥100%	<0.001

Supplemental table 2d: Difference in Kaplan-Meier failure estimate between a contemporary reference and implants with at least 250 at risk at 10 years since primary in females

Stem/cup brand	Number at risk	Cumulative failure (%)	Difference in failure (%)	95% CI	Equivalence status	p-value
[MoP] Exeter V40 Elite Plus Ogee	2,475	1.95	[REFERENCE]			
[CoC] ABG II Monolithic Cementless Stem ABG II Cementless Cup	347	3.34	1.38	[-0.27 , 3.03]	Non-inferiority not shown	0.100
[CoC] Accolade Trident	255	4.07	2.11	[1.17 , 3.05]	Inferior by ≥20%	<0.001
[CoC] Corail Pinnacle	523	3.61	1.65	[1.01 , 2.30]	Inferior by ≥20%	<0.001
[CoC] Exeter V40 ABG II Cementless Cup	302	2.63	0.67	[-0.64 , 1.98]	Non-inferiority not shown	0.317
[CoC] Exeter V40 Trident	1,079	2.45	0.50	[-0.11 , 1.11]	Non-inferiority not shown	0.106
[CoC] Furlong HAC Stem CSF	470	4.44	2.49	[1.01 , 3.97]	Inferior by ≥20%	0.001
[CoP] Exeter V40 Trilogy	300	1.58	-0.37	[-1.28 , 0.53]	Non-inferiority not shown	0.417
[CoP] Furlong HAC Stem CSF	1,467	3.08	1.12	[0.42 , 1.82]	Inferior by ≥20%	0.002
[MoP] C-Stem Cemented Stem Charnley and Elite Plus LPW	534	2.79	0.84	[-0.21 , 1.88]	Non-inferiority not shown	0.117
[MoP] C-Stem Cemented Stem Elite Plus Ogee	570	2.48	0.53	[-0.41 , 1.46]	Non-inferiority not shown	0.271
[MoP] CPT Elite Plus Ogee	256	2.80	0.85	[-0.31 , 2.01]	Non-inferiority not shown	0.151
[MoP] CPT Trilogy	754	4.11	2.16	[1.31 , 3.01]	Inferior by ≥20%	<0.001
[MoP] CPT ZCA	903	3.43	1.48	[0.76 , 2.19]	Inferior by ≥20%	<0.001
[MoP] Charnley Cemented Stem Charnley Cemented Cup	1,136	2.83	0.88	[0.10 , 1.66]	Non-inferiority not shown	0.027
[MoP] Charnley Cemented Stem Charnley Ogee	1,951	3.23	1.28	[0.63 , 1.92]	Inferior by ≥20%	<0.001
[MoP] Charnley Cemented Stem Charnley and Elite Plus LPW	1,623	2.23	0.28	[-0.34 , 0.90]	Non-inferiority not shown	0.376
[MoP] Charnley Cemented Stem Wroblewski Golf Ball	252	2.62	0.67	[-0.81 , 2.14]	Non-inferiority not shown	0.375
[MoP] Corail Duraloc Cementless Cup	783	4.61	2.66	[1.62 , 3.70]	Inferior by ≥20%	<0.001
[MoP] Corail Pinnacle	1,043	2.78	0.83	[0.28 , 1.38]	Non-inferiority not shown	0.003
[MoP] Exeter V40 ABG II Cementless Cup	279	1.55	-0.40	[-1.60 , 0.80]	Non-inferiority not shown	0.515
[MoP] Exeter V40 Cenator Cemented Cup	468	2.50	0.55	[-0.36 , 1.45]	Non-inferiority not shown	0.239
[MoP] Exeter V40 Charnley Ogee	434	1.39	-0.56	[-1.32 , 0.20]	Non-inferior	0.146
[MoP] Exeter V40 Charnley and Elite Plus LPW	292	2.60	0.65	[-0.30 , 1.60]	Non-inferiority not shown	0.180
[MoP] Exeter V40 Duraloc Cementless Cup	301	3.88	1.92	[0.23 , 3.62]	Non-inferiority not shown	0.026
[MoP] Exeter V40 Elite Plus Cemented Cup	480	1.04	-0.91	[-1.55 , -0.28]	Non-inferior	0.005
[MoP] Exeter V40 Exeter Contemporary Flanged	3,136	1.98	0.02	[-0.39 , 0.43]	Non-inferiority not shown	0.919
[MoP] Exeter V40 Exeter Contemporary Hooded	1,595	4.09	2.14	[1.52 , 2.75]	Inferior by ≥20%	<0.001
[MoP] Exeter V40 Exeter Duration	2,079	3.16	1.21	[0.64 , 1.78]	Inferior by ≥20%	<0.001
[MoP] Exeter V40 Opera	315	2.79	0.83	[-0.44 , 2.11]	Non-inferiority not shown	0.200
[MoP] Exeter V40 Trident	714	2.63	0.68	[0.06 , 1.29]	Non-inferiority not shown	0.031
[MoP] Exeter V40 Trilogy	955	2.15	0.20	[-0.40 , 0.80]	Non-inferiority not shown	0.518
[MoP] Exeter V40 Ultima Cemented Cup	334	2.65	0.70	[-0.55 , 1.94]	Non-inferiority not shown	0.275
[MoP] Furlong Cemented Stem JRI Cemented Cup	413	3.49	1.53	[0.20 , 2.87]	Non-inferiority not shown	0.024
[MoP] Furlong HAC Stem CSF	1,088	4.21	2.25	[1.46 , 3.04]	Inferior by ≥20%	<0.001
[MoP] Muller-Biomet Apollo	256	2.18	0.23	[-0.84 , 1.30]	Non-inferiority not shown	0.673
[MoP] Omnifit Cemented Stem ODC	321	4.49	2.54	[0.76 , 4.32]	Inferior by ≥20%	0.005
[MoP] Stanmore Modular Stem Stanmore-Arcom Cup	489	2.78	0.83	[-0.01 , 1.66]	Non-inferiority not shown	0.052
[MoP] Versys Cementless Stem Trilogy	263	5.52	3.57	[1.54 , 5.60]	Inferior by ≥20%	0.001

Supplemental table 3a: Difference in Kaplan-Meier failure estimate between a contemporary reference and implants with at least 250 at risk at 3 years since primary in females <55 years

Stem/cup brand	Number at risk	Cumulative failure (%)	Difference in failure (%)	95% CI	Equivalence status	p-value
[CoC] Exeter V40 Trident	1,785	0.79	[REFERENCE]			
[CoC] Accolade Trident	865	2.12	1.33	[0.33 , 2.33]	Inferior by ≥20%	0.009
[CoC] Corail DeltaMotion	342	1.53	0.74	[-0.54 , 2.02]	Non-inferiority not shown	0.256
[CoC] Corail Pinnacle	4,110	2.15	1.36	[0.81 , 1.92]	Inferior by ≥100%	<0.001
[CoC] Furlong HAC Stem Furlong HAC CSF Plus	969	1.57	0.78	[-0.01 , 1.57]	Non-inferiority not shown	0.052
[CoC] M/L Taper Cementless Continuum	324	1.22	0.43	[-0.70 , 1.57]	Non-inferiority not shown	0.456
[CoC] Taperloc Cementless Stem Exceed ABT	964	1.23	0.44	[-0.27 , 1.16]	Non-inferiority not shown	0.225
[CoP] Accolade Trident	313	1.01	0.22	[-0.67 , 1.12]	Non-inferiority not shown	0.629
[CoP] C-Stem Cemented Stem Marathon	251	1.23	0.44	[-0.71 , 1.58]	Non-inferiority not shown	0.457
[CoP] Corail Pinnacle	720	1.95	1.16	[0.31 , 2.00]	Inferior by ≥20%	0.007
[CoP] Exeter V40 Exeter X3 Rimfit	309	1.29	0.50	[-0.55 , 1.56]	Non-inferiority not shown	0.351
[CoP] Exeter V40 Trident	493	1.06	0.27	[-0.51 , 1.05]	Non-inferiority not shown	0.501
[CoP] Furlong HAC Stem CSF	259	1.81	1.02	[-0.60 , 2.64]	Non-inferiority not shown	0.217
[MoP] Accolade Trident	256	1.87	1.08	[-0.45 , 2.61]	Non-inferiority not shown	0.168
[MoP] Corail Pinnacle	594	1.73	0.94	[-0.05 , 1.93]	Non-inferiority not shown	0.062
[MoP] Exeter V40 Exeter Contemporary Flanged	366	0.56	-0.23	[-0.97 , 0.50]	Non-inferiority not shown	0.535
[MoP] Exeter V40 Trident	344	1.05	0.26	[-0.74 , 1.27]	Non-inferiority not shown	0.610

Supplemental table 3b: Difference in Kaplan-Meier failure estimate between a contemporary reference and implants with at least 250 at risk at 5 years since primary in females <55 years

Stem/cup brand	Number at risk	Cumulative failure (%)	Difference in failure (%)	95% CI	Equivalence status	p-value
[CoC] Exeter V40 Trident	1,426	1.17	[REFERENCE]			
[CoC] Accolade Trident	669	2.64	1.47	[0.31 , 2.62]	Inferior by ≥20%	0.013
[CoC] Corail Pinnacle	2,754	2.93	1.76	[1.07 , 2.45]	Inferior by ≥20%	<0.001
[CoC] Furlong HAC Stem Furlong HAC CSF Plus	610	1.82	0.65	[-0.26 , 1.57]	Non-inferiority not shown	0.161
[CoC] Taperloc Cementless Stem Exceed ABT	557	2.05	0.88	[-0.11 , 1.86]	Non-inferiority not shown	0.080
[CoP] Corail Pinnacle	369	2.59	1.42	[0.33 , 2.52]	Inferior by ≥20%	0.011
[MoP] Corail Pinnacle	365	2.65	1.48	[0.18 , 2.78]	Non-inferiority not shown	0.026
[MoP] Exeter V40 Exeter Contemporary Flanged	279	1.81	0.64	[-0.82 , 2.10]	Non-inferiority not shown	0.391

Supplemental table 3c: Difference in Kaplan-Meier failure estimate between a contemporary reference and implants with at least 250 at risk at 7 years since primary in females <55 years

Stem/cup brand	Number at risk	Cumulative failure (%)	Difference in failure (%)	95% CI	Equivalence status	p-value
[CoC] Corail Pinnacle	1,321	3.60	[REFERENCE]			
[CoC] Accolade Trident	370	3.66	0.06	[-1.38 , 1.51]	Non-inferiority not shown	0.930
[CoC] Exeter V40 Trident	982	2.07	-1.53	[-2.47 , -0.60]	Non-inferior	0.001

Supplemental table 4a: Difference in Kaplan-Meier failure estimate between a contemporary reference and implants with at least 250 at risk at 3 years since primary in females between 55 and 75 years

Stem/cup brand	Number at risk	Cumulative failure (%)	Difference in failure (%)	95% CI	Equivalence status	p-value
[MoP] Exeter V40 Elite Plus Cemented Cup	1,360	0.44	[REFERENCE]			
[CoC] ABG II Monolithic Cementless Stem ABG II Cementless Cup	375	1.57	1.13	[-0.16 , 2.42]	Non-inferiority not shown	0.087
[CoC] ABG II Monolithic Cementless Stem Trident	325	2.05	1.61	[0.07 , 3.14]	Non-inferiority not shown	0.041
[CoC] Accolade Trident	2,547	1.62	1.18	[0.60 , 1.75]	Inferior by ≥100%	<0.001
[CoC] Bimetric Cementless Stem Exceed ABT	407	1.48	1.04	[-0.10 , 2.17]	Non-inferiority not shown	0.074
[CoC] C-Stem AMT Cemented Stem Pinnacle	368	1.00	0.56	[-0.38 , 1.50]	Non-inferiority not shown	0.245
[CoC] CPT Continuum	275	0.90	0.45	[-0.61 , 1.52]	Non-inferiority not shown	0.405
[CoC] Corail Delta TT	293	2.02	1.58	[0.29 , 2.86]	Inferior by ≥20%	0.016
[CoC] Corail DeltaMotion	347	0.95	0.51	[-0.48 , 1.50]	Non-inferiority not shown	0.312
[CoC] Corail Duraloc Option	382	1.52	1.08	[-0.17 , 2.33]	Non-inferiority not shown	0.092
[CoC] Corail Pinnacle	9,567	1.41	0.97	[0.57 , 1.36]	Inferior by ≥100%	<0.001
[CoC] Excia Cementless Plasmacup SC	325	1.03	0.58	[-0.48 , 1.64]	Non-inferiority not shown	0.281
[CoC] Exeter V40 ABG II Cementless Cup	640	0.41	-0.03	[-0.60 , 0.54]	Non-inferiority not shown	0.909
[CoC] Exeter V40 Trident	3,977	0.95	0.50	[0.07 , 0.94]	Non-inferiority not shown	0.024
[CoC] Furlong HAC Stem CSF	620	1.73	1.28	[0.22 , 2.34]	Inferior by ≥20%	0.018
[CoC] Furlong HAC Stem Furlong HAC CSF Plus	3,449	1.59	1.15	[0.66 , 1.63]	Inferior by ≥100%	<0.001
[CoC] M/L Taper Cementless Continuum	363	1.61	1.16	[0.01 , 2.32]	Non-inferiority not shown	0.048
[CoC] Metafix Stem Trinity	290	1.39	0.94	[-0.16 , 2.04]	Non-inferiority not shown	0.093
[CoC] Polarstem Cementless R3 Cementless	370	0.84	0.40	[-0.49 , 1.29]	Non-inferiority not shown	0.377
[CoC] SL-Plus Cementless Stem EP-Fit Plus	376	1.53	1.09	[-0.17 , 2.34]	Non-inferiority not shown	0.091
[CoC] Taperloc Cementless Stem Exceed ABT	2,580	1.46	1.01	[0.50 , 1.53]	Inferior by ≥100%	<0.001
[CoP] Accolade Trident	1,324	1.44	0.99	[0.36 , 1.63]	Inferior by ≥20%	0.002
[CoP] C-Stem Cemented Stem Elite Plus Ogee	279	0.35	-0.09	[-0.85 , 0.66]	Non-inferiority not shown	0.807
[CoP] C-Stem Cemented Stem Marathon	364	0.59	0.15	[-0.62 , 0.91]	Non-inferiority not shown	0.706
[CoP] C-Stem Cemented Stem Opera	295	0.00	- -	[-.-- , -.--]	No failures to date	
[CoP] C-Stem Cemented Stem Wroblewski Golf Ball	346	0.53	0.08	[-0.72 , 0.89]	Non-inferiority not shown	0.836
[CoP] CPT Trilogy	573	1.20	0.75	[0.13 , 1.38]	Inferior by ≥20%	0.018
[CoP] Corail Charnley and Elite Plus LPW	296	2.41	1.97	[0.28 , 3.65]	Inferior by ≥20%	0.022
[CoP] Corail Marathon	559	0.73	0.29	[-0.35 , 0.93]	Non-inferiority not shown	0.378
[CoP] Corail Pinnacle	3,489	1.19	0.75	[0.33 , 1.17]	Inferior by ≥20%	<0.001
[CoP] Corail Trilogy	355	0.81	0.37	[-0.60 , 1.34]	Non-inferiority not shown	0.460
[CoP] Exeter V40 Charnley and Elite Plus LPW	355	1.69	1.24	[0.03 , 2.45]	Non-inferiority not shown	0.044
[CoP] Exeter V40 Elite Plus Ogee	529	0.70	0.26	[-0.44 , 0.95]	Non-inferiority not shown	0.468
[CoP] Exeter V40 Exeter Contemporary Flanged	1,464	1.06	0.62	[0.05 , 1.18]	Non-inferiority not shown	0.032
[CoP] Exeter V40 Exeter Contemporary Hooded	376	1.55	1.11	[-0.08 , 2.30]	Non-inferiority not shown	0.068
[CoP] Exeter V40 Exeter Duration	369	0.98	0.54	[-0.48 , 1.55]	Non-inferiority not shown	0.299
[CoP] Exeter V40 Exeter X3 Rimfit	896	0.66	0.22	[-0.27 , 0.70]	Non-inferiority not shown	0.385
[CoP] Exeter V40 Trident	2,023	1.02	0.57	[0.12 , 1.03]	Inferior by ≥20%	0.014
[CoP] Exeter V40 Trilogy	896	0.61	0.16	[-0.42 , 0.75]	Non-inferiority not shown	0.587
[CoP] Furlong HAC Stem CSF	2,797	1.29	0.85	[0.33 , 1.37]	Inferior by ≥20%	0.001

[CoP] Furlong HAC Stem Furlong HAC CSF Plus	660	2.00	1.56	[0.58 , 2.54]	Inferior by ≥100%	0.002
[CoP] MS-30 Original ME Muller Low Profile Cup	785	0.57	0.13	[-0.47 , 0.73]	Non-inferiority not shown	0.674
[CoP] SL-Plus Cementless Stem EP-Fit Plus	368	1.82	1.38	[-0.00 , 2.75]	Non-inferiority not shown	0.050
[CoP] Taperloc Cementless Stem Exceed ABT	789	1.04	0.60	[-0.03 , 1.22]	Non-inferiority not shown	0.062
[MoP] Accolade Trident	3,609	1.81	1.36	[0.84 , 1.89]	Inferior by ≥100%	<0.001
[MoP] Anthology R3 Cementless	506	1.00	0.56	[-0.18 , 1.29]	Non-inferiority not shown	0.138
[MoP] C-Stem AMT Cemented Stem Charnley and Elite Plus LPW	779	0.76	0.32	[-0.34 , 0.97]	Non-inferiority not shown	0.341
[MoP] C-Stem AMT Cemented Stem Elite Plus Ogee	482	0.79	0.35	[-0.43 , 1.12]	Non-inferiority not shown	0.380
[MoP] C-Stem AMT Cemented Stem Marathon	406	1.09	0.65	[-0.12 , 1.42]	Non-inferiority not shown	0.099
[MoP] C-Stem AMT Cemented Stem Pinnacle	609	1.05	0.60	[-0.07 , 1.27]	Non-inferiority not shown	0.077
[MoP] C-Stem Cemented Stem Charnley Ogee	400	1.80	1.35	[0.07 , 2.64]	Non-inferiority not shown	0.038
[MoP] C-Stem Cemented Stem Charnley and Elite Plus LPW	727	1.04	0.60	[-0.19 , 1.39]	Non-inferiority not shown	0.138
[MoP] C-Stem Cemented Stem Duraloc Cementless Cup	298	1.64	1.20	[-0.27 , 2.67]	Non-inferiority not shown	0.109
[MoP] C-Stem Cemented Stem Elite Plus Ogee	1,130	0.91	0.47	[-0.14 , 1.08]	Non-inferiority not shown	0.132
[MoP] C-Stem Cemented Stem Marathon	616	0.67	0.22	[-0.37 , 0.82]	Non-inferiority not shown	0.461
[MoP] C-Stem Cemented Stem Opera	541	0.71	0.26	[-0.50 , 1.03]	Non-inferiority not shown	0.500
[MoP] C-Stem Cemented Stem Wroblewski Golf Ball	397	0.48	0.04	[-0.71 , 0.78]	Non-inferiority not shown	0.925
[MoP] CCA Cemented Stem CCB Cup	457	0.34	-0.10	[-0.68 , 0.47]	Non-inferiority not shown	0.728
[MoP] CLS Cementless Stem Allofit	259	1.45	1.01	[-0.44 , 2.46]	Non-inferiority not shown	0.174
[MoP] CPCS Opera	488	1.19	0.75	[-0.26 , 1.75]	Non-inferiority not shown	0.145
[MoP] CPT Allofit	386	0.16	-0.28	[-0.74 , 0.17]	Non-inferiority not shown	0.220
[MoP] CPT Elite Plus Ogee	922	1.28	0.84	[0.07 , 1.61]	Non-inferiority not shown	0.032
[MoP] CPT Original ME Muller Low Profile Cup	324	0.92	0.48	[-0.48 , 1.44]	Non-inferiority not shown	0.329
[MoP] CPT Pinnacle	290	0.64	0.20	[-0.75 , 1.15]	Non-inferiority not shown	0.680
[MoP] CPT Trabecular Metal Modular Cementless Cup	317	2.73	2.29	[0.72 , 3.86]	Inferior by ≥100%	0.004
[MoP] CPT Trilogy	3,394	1.23	0.79	[0.32 , 1.26]	Inferior by ≥20%	0.001
[MoP] CPT ZCA	2,305	1.24	0.79	[0.28 , 1.31]	Inferior by ≥20%	0.003
[MoP] Charnley Cemented Stem Charnley Cemented Cup	1,559	0.98	0.54	[-0.04 , 1.12]	Non-inferiority not shown	0.069
[MoP] Charnley Cemented Stem Charnley Ogee	3,266	1.18	0.73	[0.25 , 1.22]	Inferior by ≥20%	0.003
[MoP] Charnley Cemented Stem Charnley and Elite Plus LPW	2,255	0.68	0.23	[-0.24 , 0.70]	Non-inferiority not shown	0.331
[MoP] Charnley Cemented Stem Opera	498	0.39	-0.05	[-0.69 , 0.58]	Non-inferiority not shown	0.868
[MoP] Charnley Cemented Stem Wroblewski Golf Ball	333	1.43	0.98	[-0.30 , 2.27]	Non-inferiority not shown	0.134
[MoP] Corail Charnley and Elite Plus LPW	334	0.23	-0.21	[-0.77 , 0.35]	Non-inferiority not shown	0.454
[MoP] Corail Duraloc Cementless Cup	1,523	1.59	1.14	[0.44 , 1.84]	Inferior by ≥20%	0.001
[MoP] Corail Elite Plus Cemented Cup	354	0.25	-0.20	[-0.78 , 0.39]	Non-inferiority not shown	0.510
[MoP] Corail Elite Plus Ogee	546	1.03	0.58	[-0.24 , 1.41]	Non-inferiority not shown	0.167
[MoP] Corail Marathon	1,156	0.74	0.30	[-0.21 , 0.80]	Non-inferiority not shown	0.246
[MoP] Corail Pinnacle	11,626	1.15	0.71	[0.34 , 1.08]	Inferior by ≥20%	<0.001
[MoP] Corail Trilogy	680	0.78	0.34	[-0.37 , 1.04]	Non-inferiority not shown	0.350
[MoP] Exeter V40 ABG II Cementless Cup	288	1.01	0.56	[-0.62 , 1.74]	Non-inferiority not shown	0.351
[MoP] Exeter V40 Cenator Cemented Cup	721	1.68	1.24	[0.27 , 2.20]	Inferior by ≥20%	0.012
[MoP] Exeter V40 Charnley Ogee	658	1.07	0.63	[-0.18 , 1.44]	Non-inferiority not shown	0.129
[MoP] Exeter V40 Charnley and Elite Plus LPW	943	1.27	0.82	[0.11 , 1.54]	Inferior by ≥20%	0.025
[MoP] Exeter V40 Duraloc Cementless Cup	509	0.77	0.33	[-0.49 , 1.15]	Non-inferiority not shown	0.437
[MoP] Exeter V40 EP-Fit Plus	265	1.11	0.67	[-0.63 , 1.96]	Non-inferiority not shown	0.312
[MoP] Exeter V40 Elite Plus Ogee	5,613	0.77	0.33	[-0.07 , 0.72]	Non-inferiority not shown	0.103

[MoP] Exeter V40 Exeter Contemporary Flanged	14,698	0.81	0.36	[0.01 , 0.72]	Non-inferiority not shown	0.043
[MoP] Exeter V40 Exeter Contemporary Hooded	5,787	1.59	1.14	[0.70 , 1.58]	Inferior by ≥100%	<0.001
[MoP] Exeter V40 Exeter Duration	4,914	1.14	0.70	[0.26 , 1.13]	Inferior by ≥20%	0.002
[MoP] Exeter V40 Exeter X3 Rimfit	1,966	0.71	0.27	[-0.15 , 0.68]	Non-inferiority not shown	0.210
[MoP] Exeter V40 Marathon	486	0.87	0.42	[-0.31 , 1.16]	Non-inferiority not shown	0.261
[MoP] Exeter V40 Opera	876	0.86	0.41	[-0.26 , 1.09]	Non-inferiority not shown	0.232
[MoP] Exeter V40 Pinnacle	1,011	1.06	0.61	[-0.02 , 1.25]	Non-inferiority not shown	0.058
[MoP] Exeter V40 Reflection Cementless	785	0.74	0.30	[-0.38 , 0.97]	Non-inferiority not shown	0.389
[MoP] Exeter V40 Trident	6,447	1.11	0.67	[0.28 , 1.06]	Inferior by ≥20%	0.001
[MoP] Exeter V40 Trilogy	3,453	0.79	0.34	[-0.09 , 0.77]	Non-inferiority not shown	0.119
[MoP] Exeter V40 Ultima Cemented Cup	425	1.38	0.93	[-0.21 , 2.08]	Non-inferiority not shown	0.109
[MoP] Furlong Cemented Stem JRI Cemented Cup	495	1.90	1.46	[0.24 , 2.67]	Inferior by ≥20%	0.019
[MoP] Furlong HAC Stem CSF	2,428	1.91	1.47	[0.85 , 2.08]	Inferior by ≥100%	<0.001
[MoP] Furlong HAC Stem Furlong HAC CSF Plus	1,020	1.79	1.35	[0.60 , 2.10]	Inferior by ≥100%	<0.001
[MoP] M/L Taper Cementless Continuum	253	1.51	1.07	[-0.14 , 2.28]	Non-inferiority not shown	0.084
[MoP] Muller Straight Stem Original ME Muller Low Profile Cup	530	1.06	0.62	[-0.24 , 1.47]	Non-inferiority not shown	0.157
[MoP] Muller-Biomet Apollo	634	1.93	1.49	[0.40 , 2.58]	Inferior by ≥20%	0.008
[MoP] Muller-Biomet Original ME Muller Low Profile Cup	362	1.57	1.12	[-0.16 , 2.41]	Non-inferiority not shown	0.087
[MoP] Omnifit Cemented Stem ODC	358	1.63	1.19	[-0.15 , 2.53]	Non-inferiority not shown	0.081
[MoP] Polarstem Cementless R3 Cementless	355	0.64	0.20	[-0.35 , 0.75]	Non-inferiority not shown	0.479
[MoP] SL-Plus Cementless Stem EP-Fit Plus	857	3.12	2.68	[1.56 , 3.79]	Inferior by ≥100%	<0.001
[MoP] SP II Cemented Stem Interplanta	258	2.11	1.67	[-0.04 , 3.37]	Non-inferiority not shown	0.055
[MoP] Stanmore Modular Stem SHP Cup	298	0.89	0.44	[-0.61 , 1.49]	Non-inferiority not shown	0.411
[MoP] Stanmore Modular Stem Stanmore-Arcom Cup	1,365	1.33	0.89	[0.22 , 1.56]	Inferior by ≥20%	0.009
[MoP] Synergy Cementless Stem R3 Cementless	363	0.63	0.19	[-0.46 , 0.83]	Non-inferiority not shown	0.571
[MoP] Synergy Cementless Stem Reflection Cementless	598	0.81	0.37	[-0.41 , 1.15]	Non-inferiority not shown	0.357
[MoP] Taperloc Cementless Stem Exceed ABT	1,475	2.08	1.63	[0.94 , 2.33]	Inferior by ≥100%	<0.001
[MoP] Versys Cementless Stem Trilogy	363	3.38	2.94	[1.10 , 4.78]	Inferior by ≥100%	0.002

Supplemental table 4b: Difference in Kaplan-Meier failure estimate between a contemporary reference and implants with at least 250 at risk at 5 years since primary in females between 55 and 75 years

Stem/cup brand	Number at risk	Cumulative failure (%)	Difference in failure (%)	95% CI	Equivalence status	p-value
[MoP] Exeter V40 Elite Plus Cemented Cup	1,039	0.52	[REFERENCE]			
[CoC] ABG II Monolithic Cementless Stem ABG II Cementless Cup	363	1.84	1.32	[-0.08 , 2.71]	Non-inferiority not shown	0.064
[CoC] ABG II Monolithic Cementless Stem Trident	250	3.09	2.57	[0.64 , 4.49]	Inferior by ≥100%	0.009
[CoC] Accolade Trident	2,044	2.56	2.04	[1.33 , 2.75]	Inferior by ≥100%	<0.001
[CoC] Bimetric Cementless Stem Exceed ABT	289	2.32	1.80	[0.32 , 3.28]	Inferior by ≥20%	0.017
[CoC] Corail Duraloc Option	370	2.82	2.30	[0.62 , 3.99]	Inferior by ≥100%	0.007
[CoC] Corail Pinnacle	6,445	1.82	1.30	[0.86 , 1.74]	Inferior by ≥100%	<0.001

[CoC] Exeter V40 ABG II Cementless Cup	563	1.05	0.53	[-0.33 , 1.38]	Non-inferiority not shown	0.225
[CoC] Exeter V40 Trident	3,263	1.29	0.77	[0.27 , 1.26]	Inferior by ≥20%	0.002
[CoC] Furlong HAC Stem CSF	597	2.05	1.53	[0.37 , 2.68]	Inferior by ≥20%	0.010
[CoC] Furlong HAC Stem Furlong HAC CSF Plus	2,167	1.86	1.34	[0.80 , 1.88]	Inferior by ≥100%	<0.001
[CoC] SL-Plus Cementless Stem EP-Fit Plus	352	3.15	2.63	[0.84 , 4.43]	Inferior by ≥100%	0.004
[CoC] Taperloc Cementless Stem Exceed ABT	1,583	1.68	1.16	[0.59 , 1.72]	Inferior by ≥100%	<0.001
[CoP] Accolade Trident	638	1.84	1.32	[0.56 , 2.08]	Inferior by ≥100%	0.001
[CoP] C-Stem Cemented Stem Wroblewski Golf Ball	283	0.82	0.30	[-0.69 , 1.30]	Non-inferiority not shown	0.549
[CoP] Corail Pinnacle	1,874	1.58	1.06	[0.56 , 1.57]	Inferior by ≥100%	<0.001
[CoP] Corail Trilogy	327	1.09	0.57	[-0.55 , 1.70]	Non-inferiority not shown	0.317
[CoP] Exeter V40 Elite Plus Ogee	397	1.31	0.79	[-0.20 , 1.77]	Non-inferiority not shown	0.117
[CoP] Exeter V40 Exeter Contemporary Flanged	964	1.38	0.86	[0.20 , 1.52]	Inferior by ≥20%	0.011
[CoP] Exeter V40 Exeter Contemporary Hooded	290	1.82	1.30	[-0.01 , 2.60]	Non-inferiority not shown	0.051
[CoP] Exeter V40 Exeter Duration	325	0.98	0.46	[-0.56 , 1.49]	Non-inferiority not shown	0.377
[CoP] Exeter V40 Trident	999	1.37	0.86	[0.28 , 1.43]	Inferior by ≥20%	0.003
[CoP] Exeter V40 Trilogy	748	0.72	0.20	[-0.44 , 0.84]	Non-inferiority not shown	0.540
[CoP] Furlong HAC Stem CSF	2,437	1.82	1.30	[0.69 , 1.90]	Inferior by ≥100%	<0.001
[CoP] Furlong HAC Stem Furlong HAC CSF Plus	397	2.25	1.73	[0.63 , 2.83]	Inferior by ≥100%	0.002
[CoP] MS-30 Original ME Muller Low Profile Cup	576	0.72	0.21	[-0.48 , 0.89]	Non-inferiority not shown	0.557
[CoP] SL-Plus Cementless Stem EP-Fit Plus	317	3.48	2.96	[1.07 , 4.86]	Inferior by ≥100%	0.002
[CoP] Taperloc Cementless Stem Exceed ABT	355	1.36	0.84	[0.06 , 1.61]	Non-inferiority not shown	0.035
[MoP] Accolade Trident	2,455	2.47	1.95	[1.34 , 2.56]	Inferior by ≥100%	<0.001
[MoP] C-Stem AMT Cemented Stem Charnley and Elite Plus LPW	556	1.17	0.65	[-0.16 , 1.46]	Non-inferiority not shown	0.117
[MoP] C-Stem AMT Cemented Stem Elite Plus Ogee	319	1.54	1.02	[-0.14 , 2.18]	Non-inferiority not shown	0.084
[MoP] C-Stem AMT Cemented Stem Pinnacle	308	1.68	1.16	[0.17 , 2.14]	Inferior by ≥20%	0.021
[MoP] C-Stem Cemented Stem Charnley Ogee	335	2.64	2.12	[0.53 , 3.71]	Inferior by ≥100%	0.009
[MoP] C-Stem Cemented Stem Charnley and Elite Plus LPW	671	1.88	1.36	[0.32 , 2.41]	Inferior by ≥20%	0.010
[MoP] C-Stem Cemented Stem Duraloc Cementless Cup	285	2.32	1.80	[0.06 , 3.54]	Non-inferiority not shown	0.042
[MoP] C-Stem Cemented Stem Elite Plus Ogee	906	1.40	0.88	[0.12 , 1.63]	Inferior by ≥20%	0.023
[MoP] C-Stem Cemented Stem Marathon	330	0.67	0.15	[-0.46 , 0.76]	Non-inferiority not shown	0.633
[MoP] C-Stem Cemented Stem Opera	407	0.90	0.38	[-0.48 , 1.24]	Non-inferiority not shown	0.390
[MoP] C-Stem Cemented Stem Wroblewski Golf Ball	324	1.57	1.05	[-0.25 , 2.36]	Non-inferiority not shown	0.113
[MoP] CCA Cemented Stem CCB Cup	327	0.61	0.09	[-0.70 , 0.88]	Non-inferiority not shown	0.827
[MoP] CPCS Opera	367	2.09	1.57	[0.24 , 2.91]	Inferior by ≥20%	0.021
[MoP] CPT Elite Plus Ogee	691	1.65	1.13	[0.25 , 2.01]	Inferior by ≥20%	0.012
[MoP] CPT Pinnacle	254	1.40	0.88	[-0.53 , 2.29]	Non-inferiority not shown	0.223
[MoP] CPT Trilogy	2,468	2.22	1.70	[1.10 , 2.30]	Inferior by ≥100%	<0.001
[MoP] CPT ZCA	1,787	2.02	1.50	[0.84 , 2.15]	Inferior by ≥100%	<0.001
[MoP] Charnley Cemented Stem Charnley Cemented Cup	1,420	1.52	1.00	[0.30 , 1.70]	Inferior by ≥20%	0.005
[MoP] Charnley Cemented Stem Charnley Ogee	2,959	1.84	1.32	[0.74 , 1.90]	Inferior by ≥100%	<0.001
[MoP] Charnley Cemented Stem Charnley and Elite Plus LPW	2,049	1.18	0.67	[0.09 , 1.24]	Non-inferiority not shown	0.023
[MoP] Charnley Cemented Stem Opera	454	1.22	0.70	[-0.34 , 1.73]	Non-inferiority not shown	0.185
[MoP] Charnley Cemented Stem Wroblewski Golf Ball	277	1.77	1.26	[-0.20 , 2.71]	Non-inferiority not shown	0.091
[MoP] Corail Duraloc Cementless Cup	1,465	2.31	1.79	[0.96 , 2.62]	Inferior by ≥100%	<0.001
[MoP] Corail Elite Plus Cemented Cup	277	0.55	0.03	[-0.81 , 0.87]	Non-inferiority not shown	0.947
[MoP] Corail Elite Plus Ogee	418	1.62	1.10	[0.03 , 2.17]	Non-inferiority not shown	0.044

[MoP] Corail Marathon	586	0.96	0.44	[-0.16 , 1.05]	Non-inferiority not shown	0.153
[MoP] Corail Pinnacle	7,089	1.41	0.89	[0.49 , 1.30]	Inferior by ≥20%	<0.001
[MoP] Corail Trilogy	561	0.94	0.42	[-0.36 , 1.20]	Non-inferiority not shown	0.293
[MoP] Exeter V40 ABG II Cementless Cup	283	1.01	0.49	[-0.70 , 1.67]	Non-inferiority not shown	0.422
[MoP] Exeter V40 Cenator Cemented Cup	611	2.67	2.15	[0.94 , 3.37]	Inferior by ≥100%	<0.001
[MoP] Exeter V40 Charnley Ogee	568	1.39	0.87	[-0.06 , 1.79]	Non-inferiority not shown	0.068
[MoP] Exeter V40 Charnley and Elite Plus LPW	718	1.72	1.20	[0.34 , 2.05]	Inferior by ≥20%	0.006
[MoP] Exeter V40 Duraloc Cementless Cup	488	1.37	0.85	[-0.22 , 1.93]	Non-inferiority not shown	0.119
[MoP] Exeter V40 EP-Fit Plus	250	3.01	2.49	[0.40 , 4.57]	Inferior by ≥20%	0.019
[MoP] Exeter V40 Elite Plus Ogee	4,543	1.18	0.66	[0.21 , 1.12]	Inferior by ≥20%	0.004
[MoP] Exeter V40 Exeter Contemporary Flanged	10,220	1.16	0.64	[0.25 , 1.04]	Inferior by ≥20%	0.001
[MoP] Exeter V40 Exeter Contemporary Hooded	4,277	2.45	1.93	[1.40 , 2.46]	Inferior by ≥100%	<0.001
[MoP] Exeter V40 Exeter Duration	4,048	1.48	0.96	[0.47 , 1.44]	Inferior by ≥20%	<0.001
[MoP] Exeter V40 Exeter X3 Rimfit	319	0.71	0.19	[-0.25 , 0.63]	Non-inferiority not shown	0.395
[MoP] Exeter V40 Opera	655	1.22	0.70	[-0.10 , 1.50]	Non-inferiority not shown	0.088
[MoP] Exeter V40 Pinnacle	658	1.40	0.88	[0.12 , 1.64]	Inferior by ≥20%	0.023
[MoP] Exeter V40 Reflection Cementless	732	1.14	0.62	[-0.20 , 1.44]	Non-inferiority not shown	0.141
[MoP] Exeter V40 Trident	4,016	1.44	0.92	[0.47 , 1.36]	Inferior by ≥20%	<0.001
[MoP] Exeter V40 Trilogy	2,821	1.20	0.68	[0.17 , 1.18]	Inferior by ≥20%	0.009
[MoP] Exeter V40 Ultima Cemented Cup	408	2.56	2.04	[0.51 , 3.58]	Inferior by ≥20%	0.009
[MoP] Furlong Cemented Stem JRI Cemented Cup	458	2.52	2.00	[0.60 , 3.41]	Inferior by ≥100%	0.005
[MoP] Furlong HAC Stem CSF	2,056	2.08	1.56	[0.91 , 2.22]	Inferior by ≥100%	<0.001
[MoP] Furlong HAC Stem Furlong HAC CSF Plus	633	2.82	2.30	[1.26 , 3.34]	Inferior by ≥100%	<0.001
[MoP] Muller Straight Stem Original ME Muller Low Profile Cup	395	1.47	0.95	[-0.08 , 1.98]	Non-inferiority not shown	0.071
[MoP] Muller-Biomet Apollo	535	2.11	1.59	[0.44 , 2.74]	Inferior by ≥20%	0.007
[MoP] Omnifit Cemented Stem ODC	340	2.48	1.96	[0.32 , 3.60]	Inferior by ≥20%	0.019
[MoP] SL-Plus Cementless Stem EP-Fit Plus	739	3.85	3.33	[2.07 , 4.59]	Inferior by ≥100%	<0.001
[MoP] Stanmore Modular Stem Stanmore-Arcom Cup	1,106	2.20	1.68	[0.83 , 2.53]	Inferior by ≥100%	<0.001
[MoP] Synergy Cementless Stem Reflection Cementless	577	1.15	0.63	[-0.29 , 1.55]	Non-inferiority not shown	0.179
[MoP] Taperloc Cementless Stem Exceed ABT	761	2.49	1.97	[1.18 , 2.77]	Inferior by ≥100%	<0.001
[MoP] Versys Cementless Stem Trilogy	342	4.74	4.22	[2.05 , 6.38]	Inferior by ≥100%	<0.001

Supplemental table 4c: Difference in Kaplan-Meier failure estimate between a contemporary reference and implants with at least 250 at risk at 7 years since primary in females between 55 and 75 years

Stem/cup brand	Number at risk	Cumulative failure (%)	Difference in failure (%)	95% CI	Equivalence status	p-value
[MoP] Exeter V40 Trilogy	1,962	1.45	[REFERENCE]			
[CoC] ABG II Monolithic Cementless Stem ABG II Cementless Cup	335	2.11	0.66	[-0.84 , 2.16]	Non-inferiority not shown	0.389
[CoC] Accolade Trident	1,256	3.07	1.62	[0.81 , 2.44]	Inferior by ≥20%	<0.001
[CoC] Corail Duraloc Option	301	3.15	1.70	[-0.11 , 3.50]	Non-inferiority not shown	0.065
[CoC] Corail Pinnacle	2,917	2.20	0.76	[0.25 , 1.26]	Non-inferiority not shown	0.004
[CoC] Exeter V40 ABG II Cementless Cup	454	1.65	0.20	[-0.90 , 1.31]	Non-inferiority not shown	0.721
[CoC] Exeter V40 Trident	2,340	1.76	0.31	[-0.28 , 0.90]	Non-inferiority not shown	0.300
[CoC] Furlong HAC Stem CSF	549	2.90	1.45	[0.07 , 2.83]	Non-inferiority not shown	0.040
[CoC] Furlong HAC Stem Furlong HAC CSF Plus	830	2.23	0.78	[0.12 , 1.44]	Non-inferiority not shown	0.021
[CoC] SL-Plus Cementless Stem EP-Fit Plus	295	3.46	2.01	[0.11 , 3.90]	Non-inferiority not shown	0.038
[CoC] Taperloc Cementless Stem Exceed ABT	712	1.81	0.36	[-0.29 , 1.01]	Non-inferiority not shown	0.279
[CoP] Accolade Trident	257	2.02	0.57	[-0.29 , 1.43]	Non-inferiority not shown	0.195
[CoP] Corail Pinnacle	771	1.86	0.41	[-0.20 , 1.02]	Non-inferiority not shown	0.183
[CoP] Corail Trilogy	258	1.09	-0.35	[-1.50 , 0.79]	Non-inferiority not shown	0.543
[CoP] Exeter V40 Elite Plus Ogee	251	1.59	0.15	[-1.00 , 1.29]	Non-inferiority not shown	0.804
[CoP] Exeter V40 Exeter Contemporary Flanged	563	1.38	-0.07	[-0.76 , 0.61]	Non-inferiority not shown	0.839
[CoP] Exeter V40 Exeter Duration	255	1.30	-0.14	[-1.36 , 1.07]	Non-inferiority not shown	0.818
[CoP] Exeter V40 Trident	463	1.63	0.19	[-0.52 , 0.89]	Non-inferiority not shown	0.606
[CoP] Exeter V40 Trilogy	561	1.02	-0.43	[-1.22 , 0.35]	Non-inferiority not shown	0.282
[CoP] Furlong HAC Stem CSF	2,033	2.34	0.90	[0.20 , 1.60]	Non-inferiority not shown	0.012
[CoP] MS-30 Original ME Muller Low Profile Cup	374	0.72	-0.72	[-1.43 , -0.01]	Non-inferior	0.046
[MoP] Accolade Trident	1,158	3.23	1.79	[1.03 , 2.54]	Inferior by ≥20%	<0.001
[MoP] C-Stem AMT Cemented Stem Charnley and Elite Plus LPW	344	1.72	0.28	[-0.85 , 1.41]	Non-inferiority not shown	0.632
[MoP] C-Stem Cemented Stem Charnley Ogee	284	2.97	1.52	[-0.20 , 3.24]	Non-inferiority not shown	0.084
[MoP] C-Stem Cemented Stem Charnley and Elite Plus LPW	617	2.66	1.21	[-0.04 , 2.46]	Non-inferiority not shown	0.058
[MoP] C-Stem Cemented Stem Elite Plus Ogee	733	2.13	0.68	[-0.29 , 1.65]	Non-inferiority not shown	0.170
[MoP] C-Stem Cemented Stem Opera	311	1.69	0.24	[-1.01 , 1.49]	Non-inferiority not shown	0.709
[MoP] CPT Elite Plus Ogee	470	2.58	1.13	[-0.08 , 2.34]	Non-inferiority not shown	0.067
[MoP] CPT Trilogy	1,661	2.95	1.50	[0.77 , 2.23]	Inferior by ≥20%	<0.001
[MoP] CPT ZCA	1,254	2.39	0.94	[0.20 , 1.68]	Non-inferiority not shown	0.013
[MoP] Charnley Cemented Stem Charnley Cemented Cup	1,220	2.10	0.65	[-0.18 , 1.48]	Non-inferiority not shown	0.125
[MoP] Charnley Cemented Stem Charnley Ogee	2,422	2.20	0.76	[0.11 , 1.40]	Non-inferiority not shown	0.022
[MoP] Charnley Cemented Stem Charnley and Elite Plus LPW	1,746	1.64	0.20	[-0.48 , 0.87]	Non-inferiority not shown	0.569
[MoP] Charnley Cemented Stem Opera	345	2.16	0.71	[-0.68 , 2.10]	Non-inferiority not shown	0.316
[MoP] Corail Duraloc Cementless Cup	1,258	3.66	2.21	[1.18 , 3.25]	Inferior by ≥20%	<0.001
[MoP] Corail Elite Plus Ogee	292	1.93	0.48	[-0.76 , 1.72]	Non-inferiority not shown	0.449
[MoP] Corail Pinnacle	3,659	1.82	0.38	[-0.10 , 0.86]	Non-inferiority not shown	0.123
[MoP] Corail Trilogy	369	1.65	0.20	[-0.93 , 1.34]	Non-inferiority not shown	0.725
[MoP] Exeter V40 ABG II Cementless Cup	272	1.01	-0.44	[-1.65 , 0.76]	Non-inferiority not shown	0.472

[MoP] Exeter V40 Cenator Cemented Cup	478	2.86	1.41	[0.13 , 2.69]	Non-inferiority not shown	0.030
[MoP] Exeter V40 Charnley Ogee	467	1.39	-0.06	[-1.01 , 0.89]	Non-inferiority not shown	0.897
[MoP] Exeter V40 Charnley and Elite Plus LPW	470	2.39	0.94	[-0.15 , 2.03]	Non-inferiority not shown	0.091
[MoP] Exeter V40 Duraloc Cementless Cup	392	1.79	0.34	[-0.89 , 1.56]	Non-inferiority not shown	0.589
[MoP] Exeter V40 Elite Plus Cemented Cup	701	0.63	-0.82	[-1.40 , -0.23]	Non-inferior	0.006
[MoP] Exeter V40 Elite Plus Ogee	3,436	1.59	0.14	[-0.38 , 0.67]	Non-inferiority not shown	0.593
[MoP] Exeter V40 Exeter Contemporary Flanged	6,755	1.51	0.06	[-0.39 , 0.52]	Non-inferiority not shown	0.788
[MoP] Exeter V40 Exeter Contemporary Hooded	2,891	3.20	1.75	[1.12 , 2.38]	Inferior by ≥20%	<0.001
[MoP] Exeter V40 Exeter Duration	3,049	2.17	0.72	[0.14 , 1.31]	Non-inferiority not shown	0.016
[MoP] Exeter V40 Opera	462	1.54	0.09	[-0.84 , 1.02]	Non-inferiority not shown	0.850
[MoP] Exeter V40 Pinnacle	346	1.56	0.11	[-0.73 , 0.95]	Non-inferiority not shown	0.797
[MoP] Exeter V40 Reflection Cementless	591	2.01	0.56	[-0.53 , 1.65]	Non-inferiority not shown	0.312
[MoP] Exeter V40 Trident	2,291	2.01	0.56	[0.01 , 1.11]	Non-inferiority not shown	0.046
[MoP] Exeter V40 Ultima Cemented Cup	379	2.80	1.36	[-0.26 , 2.97]	Non-inferiority not shown	0.100
[MoP] Furlong Cemented Stem JRI Cemented Cup	416	2.97	1.52	[-0.02 , 3.05]	Non-inferiority not shown	0.053
[MoP] Furlong HAC Stem CSF	1,634	2.65	1.20	[0.44 , 1.96]	Inferior by ≥20%	0.002
[MoP] Furlong HAC Stem Furlong HAC CSF Plus	270	3.32	1.87	[0.67 , 3.06]	Inferior by ≥20%	0.002
[MoP] Muller Straight Stem Original ME Muller Low Profile Cup	264	2.38	0.93	[-0.53 , 2.40]	Non-inferiority not shown	0.211
[MoP] Muller-Biomet Apollo	414	2.72	1.27	[-0.08 , 2.62]	Non-inferiority not shown	0.065
[MoP] Omnifit Cemented Stem ODC	309	3.99	2.54	[0.45 , 4.63]	Inferior by ≥20%	0.017
[MoP] SL-Plus Cementless Stem EP-Fit Plus	586	4.42	2.97	[1.59 , 4.36]	Inferior by ≥100%	<0.001
[MoP] Stanmore Modular Stem Stanmore-Arcom Cup	784	2.59	1.14	[0.19 , 2.09]	Non-inferiority not shown	0.018
[MoP] Synergy Cementless Stem Reflection Cementless	432	1.74	0.29	[-0.86 , 1.44]	Non-inferiority not shown	0.617
[MoP] Versys Cementless Stem Trilogy	318	4.74	3.29	[1.11 , 5.46]	Inferior by ≥20%	0.003

Supplemental table 4d: Difference in Kaplan-Meier failure estimate between a contemporary reference and implants with at least 250 at risk at 10 years since primary in females between 55 and 75 years

Stem/cup brand	Number at risk	Cumulative failure (%)	Difference in failure (%)	95% CI	Equivalence status	p-value
[MoP] Exeter V40 Elite Plus Ogee	1,609	2.16	[REFERENCE]			
[CoC] ABG II Monolithic Cementless Stem ABG II Cementless Cup	259	2.44	0.28	[-1.36 , 1.92]	Non-inferiority not shown	0.739
[CoC] Corail Pinnacle	342	2.97	0.81	[0.10 , 1.52]	Non-inferiority not shown	0.025
[CoC] Exeter V40 Trident	727	2.29	0.13	[-0.60 , 0.85]	Non-inferiority not shown	0.731
[CoC] Furlong HAC Stem CSF	322	3.61	1.45	[-0.16 , 3.06]	Non-inferiority not shown	0.077
[CoP] Exeter V40 Trilogy	250	1.52	-0.64	[-1.72 , 0.43]	Non-inferiority not shown	0.243
[CoP] Furlong HAC Stem CSF	1,151	3.18	1.02	[0.17 , 1.88]	Non-inferiority not shown	0.019
[MoP] C-Stem Cemented Stem Charnley and Elite Plus LPW	376	3.32	1.16	[-0.26 , 2.57]	Non-inferiority not shown	0.108
[MoP] C-Stem Cemented Stem Elite Plus Ogee	373	2.97	0.81	[-0.42 , 2.04]	Non-inferiority not shown	0.198
[MoP] CPT Trilogy	562	4.25	2.09	[1.06 , 3.12]	Inferior by ≥20%	<0.001
[MoP] CPT ZCA	520	3.72	1.56	[0.48 , 2.64]	Inferior by ≥20%	0.005
[MoP] Charnley Cemented Stem Charnley Cemented Cup	782	3.09	0.93	[-0.10 , 1.96]	Non-inferiority not shown	0.076
[MoP] Charnley Cemented Stem Charnley Ogee	1,368	3.80	1.64	[0.77 , 2.51]	Inferior by ≥20%	<0.001
[MoP] Charnley Cemented Stem Charnley and Elite Plus LPW	1,079	2.64	0.48	[-0.38 , 1.34]	Non-inferiority not shown	0.275
[MoP] Corail Duraloc Cementless Cup	598	5.19	3.03	[1.73 , 4.32]	Inferior by ≥20%	<0.001
[MoP] Corail Pinnacle	849	2.37	0.21	[-0.43 , 0.84]	Non-inferiority not shown	0.521
[MoP] Exeter V40 Cenator Cemented Cup	312	3.32	1.16	[-0.28 , 2.59]	Non-inferiority not shown	0.114
[MoP] Exeter V40 Charnley Ogee	312	1.39	-0.77	[-1.74 , 0.19]	Non-inferior	0.116
[MoP] Exeter V40 Duraloc Cementless Cup	257	3.96	1.80	[-0.21 , 3.82]	Non-inferiority not shown	0.079
[MoP] Exeter V40 Elite Plus Cemented Cup	305	0.89	-1.27	[-2.07 , -0.48]	Non-inferior	0.002
[MoP] Exeter V40 Exeter Contemporary Flanged	2,152	2.29	0.13	[-0.43 , 0.70]	Non-inferiority not shown	0.644
[MoP] Exeter V40 Exeter Contemporary Hooded	1,110	4.86	2.70	[1.83 , 3.56]	Inferior by ≥20%	<0.001
[MoP] Exeter V40 Exeter Duration	1,344	3.44	1.28	[0.51 , 2.05]	Inferior by ≥20%	0.001
[MoP] Exeter V40 Trident	523	3.05	0.89	[0.06 , 1.72]	Non-inferiority not shown	0.035
[MoP] Exeter V40 Trilogy	748	2.13	-0.03	[-0.78 , 0.72]	Non-inferiority not shown	0.931
[MoP] Furlong Cemented Stem JRI Cemented Cup	268	4.14	1.98	[0.07 , 3.89]	Non-inferiority not shown	0.043
[MoP] Furlong HAC Stem CSF	726	3.72	1.56	[0.60 , 2.51]	Inferior by ≥20%	0.001
[MoP] Stanmore Modular Stem Stanmore-Arcom Cup	329	3.31	1.15	[-0.00 , 2.30]	Non-inferiority not shown	0.050

Supplemental table 5a: Difference in Kaplan-Meier failure estimate between a contemporary reference and implants with at least 250 at risk at 3 years since primary in females >75 years

Stem/cup brand	Number at risk	Cumulative failure (%)	Difference in failure (%)	95% CI	Equivalence status	p-value
[MoP] Charnley Cemented Stem Charnley and Elite Plus LPW	1,794	0.55	[REFERENCE]			
[CoC] Accolade Trident	251	2.21	1.66	[-0.12 , 3.44]	Non-inferiority not shown	0.067
[CoC] Corail Pinnacle	757	2.22	1.67	[0.67 , 2.66]	Inferior by ≥100%	0.001
[CoC] Furlong HAC Stem Furlong HAC CSF Plus	540	0.68	0.13	[-0.55 , 0.81]	Non-inferiority not shown	0.700
[CoP] Corail Pinnacle	431	0.69	0.14	[-0.48 , 0.76]	Non-inferiority not shown	0.653
[CoP] Exeter V40 Trident	424	0.34	-0.21	[-0.72 , 0.30]	Non-inferiority not shown	0.417
[CoP] Furlong HAC Stem CSF	693	1.24	0.69	[-0.14 , 1.52]	Non-inferiority not shown	0.103
[CoP] MS-30 Original ME Muller Low Profile Cup	300	0.00	- -	[-.-- , -.--]	No failures to date	
[MoP] Accolade Trident	1,623	1.59	1.04	[0.40 , 1.69]	Inferior by ≥20%	0.002
[MoP] C-Stem AMT Cemented Stem Charnley and Elite Plus LPW	646	1.09	0.54	[-0.24 , 1.32]	Non-inferiority not shown	0.176
[MoP] C-Stem AMT Cemented Stem Elite Plus Ogee	646	0.88	0.33	[-0.37 , 1.03]	Non-inferiority not shown	0.354
[MoP] C-Stem AMT Cemented Stem Marathon	343	0.68	0.13	[-0.52 , 0.78]	Non-inferiority not shown	0.691
[MoP] C-Stem AMT Cemented Stem Pinnacle	369	1.48	0.93	[0.12 , 1.74]	Inferior by ≥20%	0.024
[MoP] C-Stem Cemented Stem Charnley and Elite Plus LPW	384	1.43	0.88	[-0.30 , 2.06]	Non-inferiority not shown	0.145
[MoP] C-Stem Cemented Stem Elite Plus Ogee	808	0.43	-0.12	[-0.65 , 0.42]	Non-inferiority not shown	0.672
[MoP] C-Stem Cemented Stem Marathon	333	0.58	0.03	[-0.63 , 0.69]	Non-inferiority not shown	0.928
[MoP] C-Stem Cemented Stem Opera	270	0.34	-0.21	[-0.95 , 0.52]	Non-inferiority not shown	0.568
[MoP] CCA Cemented Stem CCB Cup	329	1.00	0.45	[-0.59 , 1.49]	Non-inferiority not shown	0.394
[MoP] CPCS Opera	309	0.61	0.06	[-0.84 , 0.95]	Non-inferiority not shown	0.904
[MoP] CPT Elite Plus Ogee	585	1.11	0.56	[-0.27 , 1.40]	Non-inferiority not shown	0.186
[MoP] CPT Original ME Muller Low Profile Cup	272	1.78	1.23	[-0.25 , 2.70]	Non-inferiority not shown	0.104
[MoP] CPT Trabecular Metal Modular Cementless Cup	306	0.92	0.37	[-0.60 , 1.34]	Non-inferiority not shown	0.451
[MoP] CPT Trilogy	2,024	1.12	0.57	[0.07 , 1.08]	Non-inferiority not shown	0.027
[MoP] CPT ZCA	3,039	1.37	0.82	[0.34 , 1.30]	Inferior by ≥20%	0.001
[MoP] Charnley Cemented Stem Charnley Cemented Cup	926	0.92	0.37	[-0.31 , 1.05]	Non-inferiority not shown	0.289
[MoP] Charnley Cemented Stem Charnley Ogee	2,041	0.79	0.24	[-0.25 , 0.72]	Non-inferiority not shown	0.344
[MoP] Charnley Cemented Stem Opera	306	0.93	0.38	[-0.72 , 1.49]	Non-inferiority not shown	0.494
[MoP] Charnley Cemented Stem Wroblewski Golf Ball	275	1.66	1.11	[-0.37 , 2.59]	Non-inferiority not shown	0.142
[MoP] Corail Duraloc Cementless Cup	535	0.68	0.13	[-0.61 , 0.87]	Non-inferiority not shown	0.727
[MoP] Corail Elite Plus Cemented Cup	272	0.65	0.10	[-0.86 , 1.06]	Non-inferiority not shown	0.841
[MoP] Corail Elite Plus Ogee	376	1.19	0.64	[-0.36 , 1.64]	Non-inferiority not shown	0.211
[MoP] Corail Marathon	690	0.92	0.37	[-0.28 , 1.01]	Non-inferiority not shown	0.264
[MoP] Corail Pinnacle	4,912	1.43	0.88	[0.46 , 1.30]	Inferior by ≥20%	<0.001
[MoP] Corail Trilogy	336	0.77	0.22	[-0.71 , 1.15]	Non-inferiority not shown	0.642
[MoP] Exeter V40 Cenator Cemented Cup	694	0.99	0.44	[-0.32 , 1.20]	Non-inferiority not shown	0.256
[MoP] Exeter V40 Charnley Ogee	321	0.59	0.04	[-0.84 , 0.92]	Non-inferiority not shown	0.930
[MoP] Exeter V40 Charnley and Elite Plus LPW	847	1.31	0.76	[-0.02 , 1.54]	Non-inferiority not shown	0.057
[MoP] Exeter V40 Elite Plus Cemented Cup	906	0.42	-0.14	[-0.62 , 0.35]	Non-inferiority not shown	0.587
[MoP] Exeter V40 Elite Plus Ogee	5,144	0.61	0.06	[-0.32 , 0.43]	Non-inferiority not shown	0.772

[MoP] Exeter V40 Exeter Contemporary Flanged	12,084	0.59	0.04	[-0.31 , 0.39]	Non-inferiority not shown	0.821
[MoP] Exeter V40 Exeter Contemporary Hooded	5,022	1.11	0.56	[0.15 , 0.97]	Inferior by ≥20%	0.007
[MoP] Exeter V40 Exeter Duration	3,745	0.79	0.24	[-0.18 , 0.65]	Non-inferiority not shown	0.265
[MoP] Exeter V40 Exeter X3 Rimfit	1,286	0.68	0.13	[-0.31 , 0.57]	Non-inferiority not shown	0.567
[MoP] Exeter V40 Marathon	365	0.95	0.40	[-0.38 , 1.18]	Non-inferiority not shown	0.319
[MoP] Exeter V40 Opera	732	0.48	-0.07	[-0.64 , 0.50]	Non-inferiority not shown	0.815
[MoP] Exeter V40 Pinnacle	736	0.83	0.28	[-0.31 , 0.87]	Non-inferiority not shown	0.358
[MoP] Exeter V40 Reflection Cementless	494	0.75	0.20	[-0.60 , 1.00]	Non-inferiority not shown	0.623
[MoP] Exeter V40 Trident	4,508	0.77	0.22	[-0.16 , 0.60]	Non-inferiority not shown	0.261
[MoP] Exeter V40 Trilogy	1,511	0.87	0.32	[-0.21 , 0.86]	Non-inferiority not shown	0.240
[MoP] Exeter V40 Ultima Cemented Cup	337	0.82	0.27	[-0.71 , 1.25]	Non-inferiority not shown	0.591
[MoP] Furlong Cemented Stem JRI Cemented Cup	531	0.88	0.33	[-0.51 , 1.16]	Non-inferiority not shown	0.442
[MoP] Furlong HAC Stem CSF	1,553	2.48	1.93	[1.15 , 2.70]	Inferior by ≥100%	<0.001
[MoP] Furlong HAC Stem Furlong HAC CSF Plus	792	2.58	2.03	[1.08 , 2.97]	Inferior by ≥100%	<0.001
[MoP] MS-30 Original ME Muller Low Profile Cup	401	0.36	-0.20	[-0.78 , 0.39]	Non-inferiority not shown	0.516
[MoP] Muller Straight Stem Original ME Muller Low Profile Cup	461	0.78	0.23	[-0.53 , 0.99]	Non-inferiority not shown	0.550
[MoP] Muller-Biomet Apollo	551	0.66	0.11	[-0.61 , 0.83]	Non-inferiority not shown	0.763
[MoP] Muller-Biomet Original ME Muller Low Profile Cup	342	1.08	0.53	[-0.57 , 1.63]	Non-inferiority not shown	0.346
[MoP] Omnifit Cemented Stem ODC	253	1.47	0.92	[-0.55 , 2.39]	Non-inferiority not shown	0.218
[MoP] SL-Plus Cementless Stem EP-Fit Plus	290	0.71	0.16	[-0.70 , 1.02]	Non-inferiority not shown	0.720
[MoP] Stanmore Modular Stem SHP Cup	367	0.64	0.09	[-0.70 , 0.89]	Non-inferiority not shown	0.819
[MoP] Stanmore Modular Stem Stanmore-Arcom Cup	1,325	0.86	0.31	[-0.26 , 0.87]	Non-inferiority not shown	0.289
[MoP] Taperloc Cementless Stem Exceed ABT	733	1.73	1.18	[0.37 , 1.99]	Inferior by ≥20%	0.004

Supplemental table 5b: Difference in Kaplan-Meier failure estimate between a contemporary reference and implants with at least 250 at risk at 5 years since primary in females >75 years

Stem/cup brand	Number at risk	Cumulative failure (%)	Difference in failure (%)	95% CI	Equivalence status	p-value
[MoP] Charnley Cemented Stem Charnley and Elite Plus LPW	1,515	0.73	[REFERENCE]			
[CoC] Corail Pinnacle	454	2.22	1.49	[0.47 , 2.50]	Inferior by ≥20%	0.004
[CoC] Furlong HAC Stem Furlong HAC CSF Plus	302	1.22	0.49	[-0.55 , 1.53]	Non-inferiority not shown	0.352
[CoP] Furlong HAC Stem CSF	539	1.56	0.83	[-0.13 , 1.80]	Non-inferiority not shown	0.089
[MoP] Accolade Trident	1,066	2.01	1.28	[0.53 , 2.03]	Inferior by ≥20%	0.001
[MoP] C-Stem AMT Cemented Stem Charnley and Elite Plus LPW	388	1.25	0.52	[-0.34 , 1.39]	Non-inferiority not shown	0.236
[MoP] C-Stem AMT Cemented Stem Elite Plus Ogee	345	0.88	0.15	[-0.57 , 0.88]	Non-inferiority not shown	0.682
[MoP] C-Stem Cemented Stem Charnley and Elite Plus LPW	328	1.73	1.00	[-0.33 , 2.33]	Non-inferiority not shown	0.141
[MoP] C-Stem Cemented Stem Elite Plus Ogee	614	0.57	-0.16	[-0.79 , 0.47]	Non-inferiority not shown	0.615
[MoP] CPT Elite Plus Ogee	388	1.49	0.76	[-0.24 , 1.77]	Non-inferiority not shown	0.136
[MoP] CPT Trilogy	1,273	1.88	1.15	[0.47 , 1.83]	Inferior by ≥20%	0.001
[MoP] CPT ZCA	2,186	1.86	1.13	[0.55 , 1.71]	Inferior by ≥20%	<0.001
[MoP] Charnley Cemented Stem Charnley Cemented Cup	780	1.27	0.54	[-0.27 , 1.36]	Non-inferiority not shown	0.190
[MoP] Charnley Cemented Stem Charnley Ogee	1,658	1.15	0.42	[-0.17 , 1.01]	Non-inferiority not shown	0.162
[MoP] Corail Duraloc Cementless Cup	479	1.66	0.93	[-0.21 , 2.07]	Non-inferiority not shown	0.111
[MoP] Corail Marathon	325	0.92	0.19	[-0.49 , 0.86]	Non-inferiority not shown	0.585
[MoP] Corail Pinnacle	2,638	1.78	1.05	[0.55 , 1.55]	Inferior by ≥20%	<0.001
[MoP] Corail Trilogy	261	1.11	0.39	[-0.78 , 1.55]	Non-inferiority not shown	0.515
[MoP] Exeter V40 Cenator Cemented Cup	533	1.32	0.59	[-0.31 , 1.49]	Non-inferiority not shown	0.201
[MoP] Exeter V40 Charnley Ogee	264	1.27	0.54	[-0.76 , 1.85]	Non-inferiority not shown	0.414
[MoP] Exeter V40 Charnley and Elite Plus LPW	575	1.31	0.58	[-0.23 , 1.39]	Non-inferiority not shown	0.159
[MoP] Exeter V40 Elite Plus Cemented Cup	653	0.53	-0.20	[-0.77 , 0.38]	Non-inferiority not shown	0.502
[MoP] Exeter V40 Elite Plus Ogee	3,667	0.84	0.11	[-0.34 , 0.56]	Non-inferiority not shown	0.644
[MoP] Exeter V40 Exeter Contemporary Flanged	7,444	0.86	0.13	[-0.28 , 0.55]	Non-inferiority not shown	0.524
[MoP] Exeter V40 Exeter Contemporary Hooded	3,318	1.47	0.74	[0.24 , 1.23]	Inferior by ≥20%	0.003
[MoP] Exeter V40 Exeter Duration	2,928	1.18	0.46	[-0.05 , 0.96]	Non-inferiority not shown	0.078
[MoP] Exeter V40 Opera	530	0.63	-0.10	[-0.77 , 0.57]	Non-inferiority not shown	0.768
[MoP] Exeter V40 Pinnacle	386	1.03	0.30	[-0.44 , 1.04]	Non-inferiority not shown	0.422
[MoP] Exeter V40 Reflection Cementless	402	0.96	0.23	[-0.69 , 1.16]	Non-inferiority not shown	0.620
[MoP] Exeter V40 Trident	2,517	1.05	0.32	[-0.15 , 0.79]	Non-inferiority not shown	0.179
[MoP] Exeter V40 Trilogy	1,080	1.32	0.59	[-0.08 , 1.27]	Non-inferiority not shown	0.085
[MoP] Exeter V40 Ultima Cemented Cup	300	1.15	0.42	[-0.77 , 1.61]	Non-inferiority not shown	0.488
[MoP] Furlong Cemented Stem JRI Cemented Cup	426	0.88	0.15	[-0.71 , 1.00]	Non-inferiority not shown	0.734
[MoP] Furlong HAC Stem CSF	1,201	3.07	2.34	[1.44 , 3.24]	Inferior by ≥100%	<0.001
[MoP] Furlong HAC Stem Furlong HAC CSF Plus	481	2.71	1.98	[0.98 , 2.98]	Inferior by ≥100%	<0.001
[MoP] MS-30 Original ME Muller Low Profile Cup	259	1.30	0.57	[-0.67 , 1.81]	Non-inferiority not shown	0.366
[MoP] Muller Straight Stem Original ME Muller Low Profile Cup	319	1.04	0.31	[-0.62 , 1.25]	Non-inferiority not shown	0.514
[MoP] Muller-Biomet Apollo	422	0.86	0.13	[-0.71 , 0.98]	Non-inferiority not shown	0.759
[MoP] Stanmore Modular Stem Stanmore-Arcom Cup	979	1.12	0.39	[-0.28 , 1.06]	Non-inferiority not shown	0.253

[MoP] Taperloc Cementless Stem Exceed ABT	337	1.95	1.22	[0.29 , 2.15]	Inferior by ≥20%	0.010

Supplemental table 5c: Difference in Kaplan-Meier failure estimate between a contemporary reference and implants with at least 250 at risk at 7 years since primary in females >75 years

Stem/cup brand	Number at risk	Cumulative failure (%)	Difference in failure (%)	95% CI	Equivalence status	p-value
[MoP] Charnley Cemented Stem Charnley and Elite Plus LPW	1,119	0.88	[REFERENCE]			
[CoP] Furlong HAC Stem CSF	391	2.04	1.16	[-0.02 , 2.34]	Non-inferiority not shown	0.055
[MoP] Accolade Trident	460	2.40	1.52	[0.61 , 2.42]	Inferior by ≥20%	0.001
[MoP] C-Stem Cemented Stem Charnley and Elite Plus LPW	292	1.73	0.85	[-0.50 , 2.19]	Non-inferiority not shown	0.218
[MoP] C-Stem Cemented Stem Elite Plus Ogee	444	0.57	-0.31	[-0.98 , 0.35]	Non-inferiority not shown	0.354
[MoP] CPT Trilogy	689	2.22	1.34	[0.53 , 2.16]	Inferior by ≥20%	0.001
[MoP] CPT ZCA	1,341	2.44	1.56	[0.85 , 2.27]	Inferior by ≥20%	<0.001
[MoP] Charnley Cemented Stem Charnley Cemented Cup	605	1.72	0.84	[-0.14 , 1.81]	Non-inferiority not shown	0.093
[MoP] Charnley Cemented Stem Charnley Ogee	1,236	1.35	0.47	[-0.20 , 1.13]	Non-inferiority not shown	0.167
[MoP] Corail Duraloc Cementless Cup	363	2.36	1.48	[0.08 , 2.88]	Non-inferiority not shown	0.039
[MoP] Corail Pinnacle	1,150	2.17	1.28	[0.67 , 1.90]	Inferior by ≥20%	<0.001
[MoP] Exeter V40 Cenator Cemented Cup	373	1.32	0.44	[-0.49 , 1.37]	Non-inferiority not shown	0.355
[MoP] Exeter V40 Charnley and Elite Plus LPW	309	1.31	0.43	[-0.41 , 1.26]	Non-inferiority not shown	0.313
[MoP] Exeter V40 Elite Plus Cemented Cup	429	0.95	0.07	[-0.77 , 0.90]	Non-inferiority not shown	0.879
[MoP] Exeter V40 Elite Plus Ogee	2,329	1.18	0.30	[-0.24 , 0.83]	Non-inferiority not shown	0.283
[MoP] Exeter V40 Exeter Contemporary Flanged	4,187	1.13	0.24	[-0.24 , 0.73]	Non-inferiority not shown	0.320
[MoP] Exeter V40 Exeter Contemporary Hooded	1,866	2.16	1.28	[0.66 , 1.90]	Inferior by ≥20%	<0.001
[MoP] Exeter V40 Exeter Duration	1,961	1.66	0.78	[0.17 , 1.39]	Non-inferiority not shown	0.012
[MoP] Exeter V40 Opera	317	0.85	-0.03	[-0.86 , 0.80]	Non-inferiority not shown	0.950
[MoP] Exeter V40 Reflection Cementless	258	1.34	0.46	[-0.74 , 1.66]	Non-inferiority not shown	0.452
[MoP] Exeter V40 Trident	1,221	1.27	0.39	[-0.17 , 0.95]	Non-inferiority not shown	0.168
[MoP] Exeter V40 Trilogy	618	1.41	0.53	[-0.20 , 1.26]	Non-inferiority not shown	0.151
[MoP] Furlong Cemented Stem JRI Cemented Cup	307	1.11	0.23	[-0.76 , 1.22]	Non-inferiority not shown	0.649
[MoP] Furlong HAC Stem CSF	882	3.51	2.63	[1.63 , 3.63]	Inferior by ≥100%	<0.001
[MoP] Muller-Biomet Apollo	288	0.86	-0.02	[-0.89 , 0.85]	Non-inferiority not shown	0.965
[MoP] Stanmore Modular Stem Stanmore-Arcom Cup	575	1.36	0.48	[-0.29 , 1.26]	Non-inferiority not shown	0.223

Supplemental table 6a: Difference in Kaplan-Meier failure estimate between a contemporary reference and implants with at least 250 at risk at 3 years since primary in males

Stem/cup brand	Number at risk	Cumulative failure (%)	Difference in failure (%)	95% CI	Equivalence status	p-value
[CoP] Exeter V40 Exeter Contemporary Flanged	1,147	0.76	[REFERENCE]			
[CoC] ABG II Monolithic Cementless Stem ABG II Cementless Cup	560	2.22	1.46	[0.19 , 2.73]	Inferior by ≥20%	0.024
[CoC] ABG II Monolithic Cementless Stem Trident	544	1.76	1.00	[-0.16 , 2.17]	Non-inferiority not shown	0.091
[CoC] Accolade Trident	3,001	2.33	1.57	[0.90 , 2.25]	Inferior by ≥100%	<0.001
[CoC] Bimetric Cementless Stem Exceed ABT	377	0.89	0.14	[-0.84 , 1.11]	Non-inferiority not shown	0.784
[CoC] C-Stem AMT Cemented Stem Pinnacle	284	0.43	-0.33	[-1.06 , 0.40]	Non-inferiority not shown	0.380
[CoC] CPT Continuum	321	2.54	1.78	[0.30 , 3.27]	Inferior by ≥20%	0.019
[CoC] Corail Delta TT	274	2.45	1.69	[0.11 , 3.27]	Non-inferiority not shown	0.036
[CoC] Corail DeltaMotion	452	1.67	0.91	[-0.25 , 2.08]	Non-inferiority not shown	0.125
[CoC] Corail Duraloc Option	463	2.29	1.53	[0.13 , 2.94]	Non-inferiority not shown	0.033
[CoC] Corail Pinnacle	12,616	1.92	1.16	[0.68 , 1.64]	Inferior by ≥20%	<0.001
[CoC] Excia Cementless Plasmacup SC	419	0.85	0.09	[-0.77 , 0.95]	Non-inferiority not shown	0.832
[CoC] Exeter V40 ABG II Cementless Cup	457	1.13	0.38	[-0.62 , 1.38]	Non-inferiority not shown	0.460
[CoC] Exeter V40 Trident	4,326	1.13	0.37	[-0.15 , 0.89]	Non-inferiority not shown	0.158
[CoC] Furlong Evolution Cementless Furlong HAC CSF Plus	275	2.00	1.24	[0.12 , 2.36]	Non-inferiority not shown	0.030
[CoC] Furlong HAC Stem CSF	688	2.76	2.00	[0.73 , 3.27]	Inferior by ≥20%	0.002
[CoC] Furlong HAC Stem Furlong HAC CSF Plus	4,023	1.72	0.96	[0.41 , 1.51]	Inferior by ≥20%	0.001
[CoC] M/L Taper Cementless Continuum	704	1.73	0.97	[0.05 , 1.90]	Non-inferiority not shown	0.040
[CoC] Metafix Stem Trinity	344	1.09	0.33	[-0.59 , 1.26]	Non-inferiority not shown	0.479
[CoC] Polarstem Cementless R3 Cementless	341	0.37	-0.39	[-1.06 , 0.27]	Non-inferiority not shown	0.248
[CoC] SL-Plus Cementless Stem EP-Fit Plus	601	5.11	4.36	[2.60 , 6.11]	Inferior by ≥100%	<0.001
[CoC] SL-Plus Cementless Stem R3 Cementless	298	1.48	0.72	[-0.64 , 2.08]	Non-inferiority not shown	0.298
[CoC] SPS Modular April - Ceramic	291	0.59	-0.17	[-1.09 , 0.75]	Non-inferiority not shown	0.722
[CoC] Taperloc Cementless Stem Exceed ABT	2,976	1.64	0.88	[0.30 , 1.46]	Inferior by ≥20%	0.003
[CoC] miniHip Trinity	273	3.21	2.46	[0.68 , 4.24]	Inferior by ≥20%	0.007
[CoP] Accolade Trident	1,319	1.66	0.91	[0.21 , 1.60]	Inferior by ≥20%	0.011
[CoP] C-Stem Cemented Stem Elite Plus Ogee	265	0.93	0.17	[-0.96 , 1.30]	Non-inferiority not shown	0.771
[CoP] C-Stem Cemented Stem Marathon	526	1.07	0.31	[-0.49 , 1.11]	Non-inferiority not shown	0.447
[CoP] C-Stem Cemented Stem Opera	341	1.15	0.39	[-0.81 , 1.60]	Non-inferiority not shown	0.520
[CoP] C-Stem Cemented Stem Wroblewski Golf Ball	396	0.90	0.14	[-0.84 , 1.12]	Non-inferiority not shown	0.779
[CoP] CPT Trilogy	450	1.91	1.15	[0.24 , 2.05]	Inferior by ≥20%	0.013
[CoP] Corail Charnley and Elite Plus LPW	252	1.10	0.35	[-0.97 , 1.66]	Non-inferiority not shown	0.606
[CoP] Corail Marathon	525	1.69	0.93	[-0.02 , 1.89]	Non-inferiority not shown	0.055
[CoP] Corail Pinnacle	3,493	1.14	0.38	[-0.11 , 0.88]	Non-inferiority not shown	0.131
[CoP] Exeter V40 Charnley and Elite Plus LPW	300	1.07	0.31	[-0.82 , 1.44]	Non-inferiority not shown	0.593
[CoP] Exeter V40 Elite Plus Ogee	536	0.86	0.10	[-0.71 , 0.92]	Non-inferiority not shown	0.803
[CoP] Exeter V40 Exeter Contemporary Hooded	298	2.35	1.59	[-0.00 , 3.18]	Non-inferiority not shown	0.050
[CoP] Exeter V40 Exeter Duration	300	0.27	-0.49	[-1.17 , 0.18]	Non-inferiority not shown	0.152
[CoP] Exeter V40 Exeter X3 Rimfit	911	1.32	0.56	[-0.15 , 1.27]	Non-inferiority not shown	0.120
[CoP] Exeter V40 Pinnacle	251	1.17	0.41	[-0.58 , 1.40]	Non-inferiority not shown	0.418

[CoP] Exeter V40 Trident	1,917	0.79	0.04	[-0.47 , 0.55]	Non-inferiority not shown	0.887
[CoP] Exeter V40 Trilogy	764	1.73	0.97	[0.00 , 1.94]	Non-inferiority not shown	0.050
[CoP] Furlong HAC Stem CSF	2,536	1.18	0.42	[-0.17 , 1.01]	Non-inferiority not shown	0.162
[CoP] Furlong HAC Stem Furlong HAC CSF Plus	699	1.22	0.46	[-0.33 , 1.26]	Non-inferiority not shown	0.250
[CoP] MS-30 Original ME Muller Low Profile Cup	459	0.35	-0.41	[-1.06 , 0.24]	Non-inferiority not shown	0.217
[CoP] SL-Plus Cementless Stem Bicon-Plus	279	3.64	2.88	[0.73 , 5.04]	Inferior by ≥20%	0.009
[CoP] SL-Plus Cementless Stem EP-Fit Plus	366	1.82	1.06	[-0.34 , 2.47]	Non-inferiority not shown	0.138
[CoP] Taperloc Cementless Stem Exceed ABT	821	1.17	0.41	[-0.27 , 1.09]	Non-inferiority not shown	0.240
[MoP] Accolade Trident	3,597	2.40	1.65	[1.02 , 2.27]	Inferior by ≥100%	<0.001
[MoP] Anthology R3 Cementless	427	2.22	1.46	[0.36 , 2.56]	Inferior by ≥20%	0.009
[MoP] C-Stem AMT Cemented Stem Charnley and Elite Plus LPW	717	1.65	0.89	[-0.07 , 1.85]	Non-inferiority not shown	0.070
[MoP] C-Stem AMT Cemented Stem Elite Plus Ogee	473	0.61	-0.15	[-0.89 , 0.59]	Non-inferiority not shown	0.690
[MoP] C-Stem AMT Cemented Stem Marathon	406	1.04	0.28	[-0.47 , 1.03]	Non-inferiority not shown	0.461
[MoP] C-Stem AMT Cemented Stem Pinnacle	495	1.18	0.42	[-0.37 , 1.22]	Non-inferiority not shown	0.296
[MoP] C-Stem Cemented Stem Charnley Ogee	251	0.91	0.15	[-0.96 , 1.26]	Non-inferiority not shown	0.794
[MoP] C-Stem Cemented Stem Charnley and Elite Plus LPW	476	0.79	0.03	[-0.85 , 0.92]	Non-inferiority not shown	0.942
[MoP] C-Stem Cemented Stem Elite Plus Cemented Cup	261	0.70	-0.06	[-1.12 , 1.00]	Non-inferiority not shown	0.918
[MoP] C-Stem Cemented Stem Elite Plus Ogee	1,224	1.18	0.42	[-0.29 , 1.12]	Non-inferiority not shown	0.243
[MoP] C-Stem Cemented Stem Marathon	597	1.40	0.65	[-0.25 , 1.54]	Non-inferiority not shown	0.155
[MoP] C-Stem Cemented Stem Opera	528	1.62	0.86	[-0.27 , 2.00]	Non-inferiority not shown	0.137
[MoP] C-Stem Cemented Stem Wroblewski Golf Ball	354	1.29	0.53	[-0.67 , 1.73]	Non-inferiority not shown	0.388
[MoP] CCA Cemented Stem CCB Cup	337	0.47	-0.28	[-1.07 , 0.50]	Non-inferiority not shown	0.480
[MoP] CLS Cementless Stem Allofit	265	3.32	2.56	[0.49 , 4.63]	Inferior by ≥20%	0.015
[MoP] CPCS Opera	371	0.51	-0.24	[-1.07 , 0.59]	Non-inferiority not shown	0.567
[MoP] CPT Elite Plus Ogee	862	1.64	0.88	[-0.02 , 1.79]	Non-inferiority not shown	0.056
[MoP] CPT Trilogy	2,750	1.70	0.95	[0.35 , 1.54]	Inferior by ≥20%	0.002
[MoP] CPT ZCA	2,153	1.52	0.76	[0.15 , 1.37]	Non-inferiority not shown	0.015
[MoP] Charnley Cemented Stem Charnley Cemented Cup	1,527	1.34	0.58	[-0.12 , 1.28]	Non-inferiority not shown	0.107
[MoP] Charnley Cemented Stem Charnley Ogee	3,293	1.35	0.59	[0.02 , 1.16]	Non-inferiority not shown	0.043
[MoP] Charnley Cemented Stem Charnley and Elite Plus LPW	1,647	0.97	0.22	[-0.41 , 0.84]	Non-inferiority not shown	0.496
[MoP] Charnley Cemented Stem Opera	407	1.15	0.39	[-0.70 , 1.49]	Non-inferiority not shown	0.479
[MoP] Charnley Cemented Stem Wroblewski Golf Ball	382	1.45	0.69	[-0.54 , 1.92]	Non-inferiority not shown	0.270
[MoP] Corail Duraloc Cementless Cup	1,310	1.59	0.83	[0.05 , 1.62]	Non-inferiority not shown	0.038
[MoP] Corail Elite Plus Cemented Cup	337	1.52	0.76	[-0.52 , 2.04]	Non-inferiority not shown	0.245
[MoP] Corail Elite Plus Ogee	471	1.51	0.75	[-0.32 , 1.82]	Non-inferiority not shown	0.171
[MoP] Corail Marathon	978	1.50	0.74	[-0.00 , 1.48]	Non-inferiority not shown	0.050
[MoP] Corail Pinnacle	11,121	1.53	0.78	[0.31 , 1.25]	Inferior by ≥20%	0.001
[MoP] Corail Trilogy	726	1.48	0.72	[-0.21 , 1.66]	Non-inferiority not shown	0.131
[MoP] Exeter V40 ABG II Cementless Cup	258	2.15	1.39	[-0.36 , 3.15]	Non-inferiority not shown	0.120
[MoP] Exeter V40 Cenator Cemented Cup	705	1.30	0.55	[-0.37 , 1.46]	Non-inferiority not shown	0.240
[MoP] Exeter V40 Charnley and Elite Plus LPW	667	1.09	0.33	[-0.50 , 1.16]	Non-inferiority not shown	0.437
[MoP] Exeter V40 Duraloc Cementless Cup	355	2.15	1.40	[-0.14 , 2.93]	Non-inferiority not shown	0.075
[MoP] Exeter V40 Elite Plus Cemented Cup	1,212	0.84	0.08	[-0.56 , 0.72]	Non-inferiority not shown	0.809
[MoP] Exeter V40 Elite Plus Ogee	5,579	0.96	0.21	[-0.29 , 0.70]	Non-inferiority not shown	0.413
[MoP] Exeter V40 Exeter Contemporary Flanged	13,647	1.12	0.36	[-0.09 , 0.82]	Non-inferiority not shown	0.118
[MoP] Exeter V40 Exeter Contemporary Hooded	5,115	1.83	1.07	[0.53 , 1.60]	Inferior by ≥20%	<0.001

[MoP] Exeter V40 Exeter Duration	4,042	1.60	0.85	[0.29 , 1.41]	Inferior by ≥20%	0.003
[MoP] Exeter V40 Exeter X3 Rimfit	1,854	1.41	0.65	[0.06 , 1.24]	Non-inferiority not shown	0.031
[MoP] Exeter V40 Marathon	418	1.41	0.65	[-0.32 , 1.61]	Non-inferiority not shown	0.188
[MoP] Exeter V40 Opera	714	1.04	0.28	[-0.56 , 1.11]	Non-inferiority not shown	0.514
[MoP] Exeter V40 Pinnacle	784	1.67	0.91	[0.08 , 1.75]	Non-inferiority not shown	0.033
[MoP] Exeter V40 Reflection Cementless	729	1.79	1.04	[0.01 , 2.06]	Non-inferiority not shown	0.048
[MoP] Exeter V40 Trident	6,504	1.31	0.55	[0.07 , 1.04]	Non-inferiority not shown	0.025
[MoP] Exeter V40 Trilogy	3,337	1.06	0.31	[-0.23 , 0.85]	Non-inferiority not shown	0.265
[MoP] Exeter V40 Ultima Cemented Cup	363	2.49	1.73	[0.15 , 3.32]	Non-inferiority not shown	0.032
[MoP] Furlong Cemented Stem JRI Cemented Cup	443	1.25	0.50	[-0.59 , 1.58]	Non-inferiority not shown	0.370
[MoP] Furlong HAC Stem CSF	2,566	1.90	1.14	[0.49 , 1.79]	Inferior by ≥20%	0.001
[MoP] Furlong HAC Stem Furlong HAC CSF Plus	1,170	2.43	1.68	[0.85 , 2.50]	Inferior by ≥100%	<0.001
[MoP] M/L Taper Cementless Allofit	339	1.83	1.08	[-0.26 , 2.41]	Non-inferiority not shown	0.113
[MoP] Muller Straight Stem Original ME Muller Low Profile Cup	414	0.52	-0.24	[-0.97 , 0.49]	Non-inferiority not shown	0.522
[MoP] Muller-Biomet Apollo	748	1.13	0.37	[-0.48 , 1.22]	Non-inferiority not shown	0.395
[MoP] Omnifit Cemented Stem ODC	267	1.04	0.28	[-0.96 , 1.52]	Non-inferiority not shown	0.661
[MoP] Polarstem Cementless R3 Cementless	476	1.24	0.48	[-0.21 , 1.18]	Non-inferiority not shown	0.174
[MoP] SL-Plus Cementless Stem EP-Fit Plus	826	2.89	2.13	[1.01 , 3.26]	Inferior by ≥100%	<0.001
[MoP] Stanmore Modular Stem SHP Cup	267	0.99	0.23	[-0.97 , 1.44]	Non-inferiority not shown	0.702
[MoP] Stanmore Modular Stem Stanmore-Arcom Cup	1,070	1.02	0.26	[-0.44 , 0.97]	Non-inferiority not shown	0.461
[MoP] Synergy Cementless Stem R3 Cementless	503	1.52	0.76	[-0.10 , 1.63]	Non-inferiority not shown	0.083
[MoP] Synergy Cementless Stem Reflection Cementless	591	1.14	0.38	[-0.56 , 1.32]	Non-inferiority not shown	0.429
[MoP] Taperloc Cementless Stem Exceed ABT	1,447	1.40	0.64	[0.00 , 1.28]	Non-inferiority not shown	0.049
[MoP] Versys Cementless Stem Trilogy	369	4.30	3.54	[1.49 , 5.58]	Inferior by ≥100%	0.001

Supplemental table 6b: Difference in Kaplan-Meier failure estimate between a contemporary reference and implants with at least 250 at risk at 5 years since primary in males

Stem/cup brand	Number at risk	Cumulative failure (%)	Difference in failure (%)	95% CI	Equivalence status	p-value
[MoP] Exeter V40 Elite Plus Ogee	4,143	1.29	[REFERENCE]			
[CoC] ABG II Monolithic Cementless Stem ABG II Cementless Cup	536	2.75	1.47	[0.11 , 2.82]	Non-inferiority not shown	0.035
[CoC] ABG II Monolithic Cementless Stem Trident	463	3.15	1.86	[0.36 , 3.37]	Inferior by ≥20%	0.015
[CoC] Accolade Trident	2,264	3.15	1.87	[1.19 , 2.54]	Inferior by ≥20%	<0.001
[CoC] Bimetric Cementless Stem Exceed ABT	259	1.75	0.46	[-0.86 , 1.79]	Non-inferiority not shown	0.494
[CoC] Corail DeltaMotion	261	1.91	0.62	[-0.59 , 1.83]	Non-inferiority not shown	0.315
[CoC] Corail Duraloc Option	449	3.36	2.07	[0.43 , 3.72]	Inferior by ≥20%	0.013
[CoC] Corail Pinnacle	7,860	2.68	1.40	[1.01 , 1.79]	Inferior by ≥20%	<0.001
[CoC] Exeter V40 ABG II Cementless Cup	379	1.39	0.10	[-0.97 , 1.17]	Non-inferiority not shown	0.852
[CoC] Exeter V40 Trident	3,289	1.81	0.52	[0.04 , 1.00]	Non-inferiority not shown	0.034
[CoC] Furlong HAC Stem CSF	664	3.19	1.91	[0.59 , 3.22]	Inferior by ≥20%	0.005
[CoC] Furlong HAC Stem Furlong HAC CSF Plus	2,393	1.91	0.62	[0.15 , 1.10]	Non-inferiority not shown	0.010
[CoC] M/L Taper Cementless Continuum	304	1.73	0.44	[-0.42 , 1.31]	Non-inferiority not shown	0.316
[CoC] SL-Plus Cementless Stem EP-Fit Plus	551	6.43	5.14	[3.22 , 7.07]	Inferior by ≥100%	<0.001

[CoC] Taperloc Cementless Stem Exceed ABT	1,657	1.87	0.59	[0.07 , 1.11]	Non-inferiority not shown	0.027
[CoP] Accolade Trident	544	2.06	0.77	[0.05 , 1.50]	Non-inferiority not shown	0.037
[CoP] C-Stem Cemented Stem Opera	283	1.49	0.21	[-1.13 , 1.54]	Non-inferiority not shown	0.759
[CoP] C-Stem Cemented Stem Wroblewski Golf Ball	321	1.47	0.18	[-1.03 , 1.39]	Non-inferiority not shown	0.769
[CoP] Corail Pinnacle	1,695	1.78	0.49	[0.01 , 0.98]	Non-inferiority not shown	0.047
[CoP] Exeter V40 Elite Plus Ogee	381	1.11	-0.18	[-1.06 , 0.71]	Non-inferiority not shown	0.697
[CoP] Exeter V40 Exeter Contemporary Flanged	789	0.85	-0.44	[-0.98 , 0.11]	Non-inferior	0.116
[CoP] Exeter V40 Exeter Duration	261	1.01	-0.28	[-1.46 , 0.90]	Non-inferiority not shown	0.641
[CoP] Exeter V40 Trident	891	0.96	-0.33	[-0.79 , 0.13]	Non-inferior	0.162
[CoP] Exeter V40 Trilogy	631	2.00	0.72	[-0.27 , 1.71]	Non-inferiority not shown	0.154
[CoP] Furlong HAC Stem CSF	2,209	1.39	0.10	[-0.42 , 0.62]	Non-inferiority not shown	0.703
[CoP] Furlong HAC Stem Furlong HAC CSF Plus	400	1.38	0.10	[-0.69 , 0.88]	Non-inferiority not shown	0.809
[CoP] MS-30 Original ME Muller Low Profile Cup	330	0.35	-0.94	[-1.50 , -0.38]	Non-inferior	0.001
[CoP] SL-Plus Cementless Stem Bicon-Plus	253	4.74	3.46	[1.01 , 5.90]	Inferior by ≥20%	0.006
[CoP] SL-Plus Cementless Stem EP-Fit Plus	312	3.28	1.99	[0.14 , 3.85]	Non-inferiority not shown	0.035
[CoP] Taperloc Cementless Stem Exceed ABT	309	1.70	0.42	[-0.54 , 1.37]	Non-inferiority not shown	0.391
[MoP] Accolade Trident	2,404	3.37	2.08	[1.45 , 2.71]	Inferior by ≥100%	<0.001
[MoP] C-Stem AMT Cemented Stem Charnley and Elite Plus LPW	444	1.81	0.52	[-0.44 , 1.47]	Non-inferiority not shown	0.286
[MoP] C-Stem AMT Cemented Stem Elite Plus Ogee	253	0.61	-0.68	[-1.34 , -0.01]	Non-inferior	0.045
[MoP] C-Stem Cemented Stem Charnley and Elite Plus LPW	429	1.01	-0.27	[-1.20 , 0.66]	Non-inferiority not shown	0.564
[MoP] C-Stem Cemented Stem Elite Plus Ogee	906	1.28	0.00	[-0.66 , 0.66]	Non-inferiority not shown	0.997
[MoP] C-Stem Cemented Stem Marathon	274	1.82	0.54	[-0.48 , 1.55]	Non-inferiority not shown	0.300
[MoP] C-Stem Cemented Stem Opera	389	2.23	0.94	[-0.34 , 2.23]	Non-inferiority not shown	0.149
[MoP] C-Stem Cemented Stem Wroblewski Golf Ball	269	1.29	0.00	[-1.16 , 1.16]	Non-inferiority not shown	0.999
[MoP] CPCS Opera	281	0.51	-0.77	[-1.54 , -0.01]	Non-inferior	0.048
[MoP] CPT Elite Plus Ogee	630	2.35	1.07	[0.02 , 2.11]	Non-inferiority not shown	0.046
[MoP] CPT Trilogy	1,862	2.39	1.11	[0.50 , 1.71]	Inferior by ≥20%	<0.001
[MoP] CPT ZCA	1,549	2.42	1.13	[0.46 , 1.80]	Inferior by ≥20%	0.001
[MoP] Charnley Cemented Stem Charnley Cemented Cup	1,338	2.22	0.93	[0.15 , 1.71]	Non-inferiority not shown	0.019
[MoP] Charnley Cemented Stem Charnley Ogee	2,824	2.29	1.00	[0.42 , 1.58]	Inferior by ≥20%	0.001
[MoP] Charnley Cemented Stem Charnley and Elite Plus LPW	1,414	1.42	0.14	[-0.49 , 0.76]	Non-inferiority not shown	0.667
[MoP] Charnley Cemented Stem Opera	343	1.68	0.40	[-0.88 , 1.67]	Non-inferiority not shown	0.540
[MoP] Charnley Cemented Stem Wroblewski Golf Ball	311	1.75	0.47	[-0.86 , 1.79]	Non-inferiority not shown	0.489
[MoP] Corail Duraloc Cementless Cup	1,204	2.54	1.25	[0.36 , 2.14]	Inferior by ≥20%	0.006
[MoP] Corail Elite Plus Ogee	352	1.79	0.50	[-0.66 , 1.66]	Non-inferiority not shown	0.397
[MoP] Corail Marathon	465	1.77	0.48	[-0.28 , 1.24]	Non-inferiority not shown	0.216
[MoP] Corail Pinnacle	6,318	1.91	0.63	[0.26 , 0.99]	Inferior by ≥20%	0.001
[MoP] Corail Trilogy	526	2.48	1.20	[0.01 , 2.38]	Non-inferiority not shown	0.047
[MoP] Exeter V40 Cenator Cemented Cup	573	1.74	0.45	[-0.53 , 1.43]	Non-inferiority not shown	0.366
[MoP] Exeter V40 Charnley and Elite Plus LPW	504	1.09	-0.20	[-0.97 , 0.57]	Non-inferiority not shown	0.612
[MoP] Exeter V40 Duraloc Cementless Cup	330	2.45	1.16	[-0.44 , 2.77]	Non-inferiority not shown	0.156
[MoP] Exeter V40 Elite Plus Cemented Cup	906	1.20	-0.09	[-0.74 , 0.57]	Non-inferiority not shown	0.797
[MoP] Exeter V40 Exeter Contemporary Flanged	8,757	1.54	0.26	[-0.09 , 0.60]	Non-inferiority not shown	0.145
[MoP] Exeter V40 Exeter Contemporary Hooded	3,570	2.27	0.98	[0.51 , 1.45]	Inferior by ≥20%	<0.001
[MoP] Exeter V40 Exeter Duration	3,104	2.30	1.02	[0.49 , 1.54]	Inferior by ≥20%	<0.001
[MoP] Exeter V40 Exeter X3 Rimfit	309	1.61	0.33	[-0.31 , 0.96]	Non-inferiority not shown	0.314

[MoP] Exeter V40 Opera	515	1.66	0.37	[-0.61 , 1.35]	Non-inferiority not shown	0.457
[MoP] Exeter V40 Pinnacle	471	2.43	1.14	[0.13 , 2.15]	Non-inferiority not shown	0.027
[MoP] Exeter V40 Reflection Cementless	649	2.49	1.21	[0.06 , 2.35]	Non-inferiority not shown	0.039
[MoP] Exeter V40 Trident	3,769	1.68	0.40	[-0.00 , 0.80]	Non-inferiority not shown	0.051
[MoP] Exeter V40 Trilogy	2,555	1.46	0.18	[-0.31 , 0.66]	Non-inferiority not shown	0.473
[MoP] Exeter V40 Ultima Cemented Cup	321	2.77	1.48	[-0.16 , 3.12]	Non-inferiority not shown	0.077
[MoP] Furlong Cemented Stem JRI Cemented Cup	396	1.48	0.19	[-0.93 , 1.32]	Non-inferiority not shown	0.735
[MoP] Furlong HAC Stem CSF	2,076	2.31	1.02	[0.40 , 1.64]	Inferior by ≥20%	0.001
[MoP] Furlong HAC Stem Furlong HAC CSF Plus	686	3.15	1.86	[0.94 , 2.79]	Inferior by ≥20%	<0.001
[MoP] Muller Straight Stem Original ME Muller Low Profile Cup	291	0.78	-0.51	[-1.33 , 0.31]	Non-inferiority not shown	0.224
[MoP] Muller-Biomet Apollo	568	1.27	-0.02	[-0.85 , 0.81]	Non-inferiority not shown	0.967
[MoP] SL-Plus Cementless Stem EP-Fit Plus	672	4.08	2.79	[1.48 , 4.11]	Inferior by ≥100%	<0.001
[MoP] Stanmore Modular Stem Stanmore-Arcom Cup	805	1.43	0.15	[-0.59 , 0.89]	Non-inferiority not shown	0.697
[MoP] Synergy Cementless Stem Reflection Cementless	546	2.18	0.90	[-0.31 , 2.10]	Non-inferiority not shown	0.146
[MoP] Taperloc Cementless Stem Exceed ABT	657	1.71	0.42	[-0.23 , 1.08]	Non-inferiority not shown	0.208
[MoP] Versys Cementless Stem Trilogy	336	4.56	3.28	[1.20 , 5.36]	Inferior by ≥20%	0.002

Supplemental table 6c: Difference in Kaplan-Meier failure estimate between a contemporary reference and implants with at least 250 at risk at 7 years since primary in males

Stem/cup brand	Number at risk	Cumulative failure (%)	Difference in failure (%)	95% CI	Equivalence status	p-value
[CoP] Furlong HAC Stem CSF	1,823	1.64	[REFERENCE]			
[CoC] ABG II Monolithic Cementless Stem ABG II Cementless Cup	469	3.52	1.89	[0.29 , 3.48]	Non-inferiority not shown	0.020
[CoC] ABG II Monolithic Cementless Stem Trident	365	4.06	2.42	[0.64 , 4.20]	Inferior by ≥20%	0.008
[CoC] Accolade Trident	1,234	3.47	1.84	[1.01 , 2.66]	Inferior by ≥20%	<0.001
[CoC] Corail Duraloc Option	354	4.27	2.64	[0.74 , 4.54]	Inferior by ≥20%	0.006
[CoC] Corail Pinnacle	3,192	3.28	1.64	[1.05 , 2.23]	Inferior by ≥20%	<0.001
[CoC] Exeter V40 ABG II Cementless Cup	304	1.98	0.35	[-1.05 , 1.75]	Non-inferiority not shown	0.627
[CoC] Exeter V40 Trident	2,289	2.18	0.54	[-0.12 , 1.20]	Non-inferiority not shown	0.108
[CoC] Furlong HAC Stem CSF	630	3.34	1.71	[0.30 , 3.11]	Non-inferiority not shown	0.017
[CoC] Furlong HAC Stem Furlong HAC CSF Plus	913	2.10	0.47	[-0.18 , 1.11]	Non-inferiority not shown	0.158
[CoC] SL-Plus Cementless Stem EP-Fit Plus	483	6.96	5.33	[3.28 , 7.38]	Inferior by ≥100%	<0.001
[CoC] Taperloc Cementless Stem Exceed ABT	628	2.14	0.51	[-0.23 , 1.24]	Non-inferiority not shown	0.175
[CoP] C-Stem Cemented Stem Wroblewski Golf Ball	252	1.47	-0.17	[-1.44 , 1.11]	Non-inferiority not shown	0.796
[CoP] Corail Pinnacle	611	2.30	0.66	[-0.12 , 1.45]	Non-inferiority not shown	0.098
[CoP] Exeter V40 Exeter Contemporary Flanged	417	0.85	-0.79	[-1.46 , -0.11]	Non-inferior	0.022
[CoP] Exeter V40 Trident	459	1.46	-0.18	[-1.01 , 0.65]	Non-inferiority not shown	0.676
[CoP] Exeter V40 Trilogy	438	2.16	0.53	[-0.58 , 1.64]	Non-inferiority not shown	0.351
[MoP] Accolade Trident	1,060	4.24	2.60	[1.71 , 3.49]	Inferior by ≥100%	<0.001
[MoP] C-Stem Cemented Stem Charnley and Elite Plus LPW	384	1.01	-0.62	[-1.63 , 0.39]	Non-inferiority not shown	0.227
[MoP] C-Stem Cemented Stem Elite Plus Ogee	654	1.54	-0.09	[-0.94 , 0.76]	Non-inferiority not shown	0.830
[MoP] C-Stem Cemented Stem Opera	282	2.55	0.92	[-0.56 , 2.40]	Non-inferiority not shown	0.225
[MoP] CPT Elite Plus Ogee	436	2.51	0.87	[-0.29 , 2.03]	Non-inferiority not shown	0.140
[MoP] CPT Trilogy	1,134	2.68	1.04	[0.28 , 1.81]	Non-inferiority not shown	0.008
[MoP] CPT ZCA	1,009	3.27	1.63	[0.71 , 2.56]	Inferior by ≥20%	0.001
[MoP] Charnley Cemented Stem Charnley Cemented Cup	1,126	2.94	1.31	[0.31 , 2.30]	Non-inferiority not shown	0.010
[MoP] Charnley Cemented Stem Charnley Ogee	2,211	3.41	1.77	[0.96 , 2.58]	Inferior by ≥20%	<0.001
[MoP] Charnley Cemented Stem Charnley and Elite Plus LPW	1,116	1.90	0.26	[-0.57 , 1.09]	Non-inferiority not shown	0.539
[MoP] Charnley Cemented Stem Wroblewski Golf Ball	256	2.09	0.46	[-1.07 , 1.98]	Non-inferiority not shown	0.559
[MoP] Corail Duraloc Cementless Cup	967	3.62	1.98	[0.84 , 3.13]	Inferior by ≥20%	0.001
[MoP] Corail Pinnacle	2,826	2.42	0.79	[0.21 , 1.37]	Non-inferiority not shown	0.008
[MoP] Corail Trilogy	323	3.87	2.23	[0.58 , 3.89]	Inferior by ≥20%	0.008
[MoP] Exeter V40 Cenator Cemented Cup	415	2.35	0.71	[-0.55 , 1.97]	Non-inferiority not shown	0.267
[MoP] Exeter V40 Charnley and Elite Plus LPW	294	1.33	-0.30	[-1.29 , 0.68]	Non-inferiority not shown	0.547
[MoP] Exeter V40 Elite Plus Cemented Cup	608	1.20	-0.44	[-1.20 , 0.33]	Non-inferiority not shown	0.266
[MoP] Exeter V40 Elite Plus Ogee	2,790	1.68	0.04	[-0.56 , 0.64]	Non-inferiority not shown	0.888
[MoP] Exeter V40 Exeter Contemporary Flanged	5,272	2.01	0.37	[-0.18 , 0.92]	Non-inferiority not shown	0.183
[MoP] Exeter V40 Exeter Contemporary Hooded	2,263	2.84	1.20	[0.53 , 1.88]	Inferior by ≥20%	<0.001
[MoP] Exeter V40 Exeter Duration	2,100	3.36	1.72	[0.96 , 2.49]	Inferior by ≥20%	<0.001
[MoP] Exeter V40 Opera	340	2.14	0.50	[-0.75 , 1.75]	Non-inferiority not shown	0.432
[MoP] Exeter V40 Reflection Cementless	483	3.34	1.70	[0.29 , 3.12]	Non-inferiority not shown	0.018

[MoP] Exeter V40 Trident	2,065	2.04	0.41	[-0.20 , 1.01]	Non-inferiority not shown	0.191
[MoP] Exeter V40 Trilogy	1,688	1.88	0.25	[-0.44 , 0.93]	Non-inferiority not shown	0.477
[MoP] Exeter V40 Ultima Cemented Cup	282	2.77	1.13	[-0.55 , 2.82]	Non-inferiority not shown	0.188
[MoP] Furlong Cemented Stem JRI Cemented Cup	312	1.77	0.13	[-1.18 , 1.45]	Non-inferiority not shown	0.843
[MoP] Furlong HAC Stem CSF	1,556	3.09	1.46	[0.62 , 2.29]	Inferior by ≥20%	0.001
[MoP] Furlong HAC Stem Furlong HAC CSF Plus	265	3.66	2.02	[0.80 , 3.25]	Inferior by ≥20%	0.001
[MoP] Muller-Biomet Apollo	376	1.46	-0.18	[-1.17 , 0.81]	Non-inferiority not shown	0.722
[MoP] SL-Plus Cementless Stem EP-Fit Plus	483	5.32	3.69	[2.08 , 5.29]	Inferior by ≥100%	<0.001
[MoP] Stanmore Modular Stem Stanmore-Arcom Cup	529	1.86	0.23	[-0.74 , 1.20]	Non-inferiority not shown	0.644
[MoP] Synergy Cementless Stem Reflection Cementless	396	2.37	0.73	[-0.59 , 2.05]	Non-inferiority not shown	0.277
[MoP] Versys Cementless Stem Trilogy	297	4.56	2.93	[0.81 , 5.05]	Inferior by ≥20%	0.007

Supplemental table 6d: Difference in Kaplan-Meier failure estimate between a contemporary reference and implants with at least 250 at risk at 10 years since primary in males

Stem/cup brand	Number at risk	Cumulative failure (%)	Difference in failure (%)	95% CI	Equivalence status	p-value
[CoP] Furlong HAC Stem CSF	1,054	2.03	[REFERENCE]			
[CoC] ABG II Monolithic Cementless Stem ABG II Cementless Cup	348	5.43	3.40	[1.33 , 5.47]	Inferior by ≥20%	0.001
[CoC] Corail Pinnacle	363	4.24	2.21	[1.28 , 3.15]	Inferior by ≥20%	<0.001
[CoC] Exeter V40 Trident	767	2.76	0.74	[-0.10 , 1.57]	Non-inferiority not shown	0.083
[CoC] Furlong HAC Stem CSF	360	4.28	2.25	[0.60 , 3.90]	Inferior by ≥20%	0.008
[MoP] C-Stem Cemented Stem Elite Plus Ogee	313	2.71	0.68	[-0.63 , 1.98]	Non-inferiority not shown	0.308
[MoP] CPT Trilogy	382	4.61	2.58	[1.24 , 3.92]	Inferior by ≥20%	<0.001
[MoP] CPT ZCA	332	4.41	2.38	[1.15 , 3.61]	Inferior by ≥20%	<0.001
[MoP] Charnley Cemented Stem Charnley Cemented Cup	615	4.69	2.66	[1.32 , 4.00]	Inferior by ≥20%	<0.001
[MoP] Charnley Cemented Stem Charnley Ogee	1,151	5.08	3.05	[2.00 , 4.10]	Inferior by ≥20%	<0.001
[MoP] Charnley Cemented Stem Charnley and Elite Plus LPW	661	2.93	0.90	[-0.21 , 2.01]	Non-inferiority not shown	0.114
[MoP] Corail Duraloc Cementless Cup	418	6.69	4.66	[2.92 , 6.41]	Inferior by ≥100%	<0.001
[MoP] Corail Pinnacle	638	3.49	1.47	[0.63 , 2.30]	Inferior by ≥20%	0.001
[MoP] Exeter V40 Elite Plus Ogee	1,105	2.53	0.51	[-0.31 , 1.32]	Non-inferiority not shown	0.226
[MoP] Exeter V40 Exeter Contemporary Flanged	1,517	2.88	0.85	[0.13 , 1.57]	Non-inferiority not shown	0.020
[MoP] Exeter V40 Exeter Contemporary Hooded	760	4.17	2.15	[1.19 , 3.11]	Inferior by ≥20%	<0.001
[MoP] Exeter V40 Exeter Duration	888	4.95	2.92	[1.88 , 3.96]	Inferior by ≥20%	<0.001
[MoP] Exeter V40 Trident	402	2.84	0.81	[-0.12 , 1.74]	Non-inferiority not shown	0.089
[MoP] Exeter V40 Trilogy	583	3.02	0.99	[0.02 , 1.96]	Non-inferiority not shown	0.045
[MoP] Furlong HAC Stem CSF	609	4.76	2.73	[1.56 , 3.90]	Inferior by ≥20%	<0.001

Supplemental table 7a: Difference in Kaplan-Meier failure estimate between a contemporary reference and implants with at least 250 at risk at 3 years since primary in males <55 years

Stem/cup brand	Number at risk	Cumulative failure (%)	Difference in failure (%)	95% CI	Equivalence status	p-value
[CoC] Exeter V40 Trident	1,269	1.26	[REFERENCE]			
[CoC] Accolade Trident	730	2.96	1.70	[0.41 , 3.00]	Inferior by ≥20%	0.010
[CoC] Corail Pinnacle	3,796	2.29	1.03	[0.33 , 1.74]	Inferior by ≥20%	0.004
[CoC] Furlong HAC Stem Furlong HAC CSF Plus	837	1.71	0.46	[-0.49 , 1.41]	Non-inferiority not shown	0.345
[CoC] M/L Taper Cementless Continuum	296	2.21	0.95	[-0.59 , 2.49]	Non-inferiority not shown	0.226
[CoC] Taperloc Cementless Stem Exceed ABT	836	2.23	0.98	[-0.05 , 2.00]	Non-inferiority not shown	0.062
[CoP] Accolade Trident	251	2.12	0.87	[-0.64 , 2.37]	Non-inferiority not shown	0.259
[CoP] Corail Pinnacle	509	1.90	0.64	[-0.34 , 1.62]	Non-inferiority not shown	0.201
[CoP] Exeter V40 Trident	369	0.81	-0.44	[-1.25 , 0.37]	Non-inferiority not shown	0.286
[MoP] Corail Pinnacle	417	1.20	-0.06	[-1.12 , 1.00]	Non-inferiority not shown	0.915

Supplemental table 7b: Difference in Kaplan-Meier failure estimate between a contemporary reference and implants with at least 250 at risk at 5 years since primary in males <55 years

Stem/cup brand	Number at risk	Cumulative failure (%)	Difference in failure (%)	95% CI	Equivalence status	p-value
[CoC] Corail Pinnacle	2,313	3.33	[REFERENCE]			
[CoC] Accolade Trident	502	4.15	0.82	[-0.70 , 2.34]	Non-inferiority not shown	0.290
[CoC] Exeter V40 Trident	934	1.87	-1.46	[-2.36 , -0.56]	Non-inferior	0.001
[CoC] Furlong HAC Stem Furlong HAC CSF Plus	494	1.85	-1.48	[-2.45 , -0.50]	Non-inferior	0.003
[CoC] Taperloc Cementless Stem Exceed ABT	430	2.57	-0.76	[-1.88 , 0.35]	Non-inferior	0.179

Supplemental table 8a: Difference in Kaplan-Meier failure estimate between a contemporary reference and implants with at least 250 at risk at 3 years since primary in males between 55 and 75 years

Stem/cup brand	Number at risk	Cumulative failure (%)	Difference in failure (%)	95% CI	Equivalence status	p-value
[CoP] Exeter V40 Trident	1,339	0.83	[REFERENCE]			
[CoC] ABG II Monolithic Cementless Stem ABG II Cementless Cup	408	2.58	1.75	[0.21 , 3.30]	Inferior by ≥20%	0.026
[CoC] ABG II Monolithic Cementless Stem Trident	368	1.82	0.99	[-0.39 , 2.37]	Non-inferiority not shown	0.159
[CoC] Accolade Trident	2,101	2.16	1.33	[0.64 , 2.02]	Inferior by ≥20%	<0.001
[CoC] Bimetric Cementless Stem Exceed ABT	279	0.60	-0.22	[-1.13 , 0.68]	Non-inferiority not shown	0.628
[CoC] Corail Duraloc Option	311	1.86	1.04	[-0.48 , 2.55]	Non-inferiority not shown	0.181
[CoC] Corail Pinnacle	8,280	1.71	0.89	[0.46 , 1.31]	Inferior by ≥20%	<0.001
[CoC] Excia Cementless Plasmacup SC	284	1.25	0.42	[-0.72 , 1.57]	Non-inferiority not shown	0.468
[CoC] Exeter V40 ABG II Cementless Cup	324	0.80	-0.03	[-0.99 , 0.93]	Non-inferiority not shown	0.949
[CoC] Exeter V40 Trident	2,957	1.06	0.23	[-0.26 , 0.72]	Non-inferiority not shown	0.350
[CoC] Furlong HAC Stem CSF	452	3.36	2.53	[0.88 , 4.19]	Inferior by ≥100%	0.003
[CoC] Furlong HAC Stem Furlong HAC CSF Plus	2,839	1.63	0.80	[0.27 , 1.33]	Inferior by ≥20%	0.003
[CoC] M/L Taper Cementless Continuum	405	1.41	0.58	[-0.45 , 1.61]	Non-inferiority not shown	0.269
[CoC] Polarstem Cementless R3 Cementless	256	0.54	-0.29	[-1.11 , 0.54]	Non-inferiority not shown	0.496
[CoC] SL-Plus Cementless Stem EP-Fit Plus	398	4.90	4.07	[2.00 , 6.14]	Inferior by ≥100%	<0.001
[CoC] Taperloc Cementless Stem Exceed ABT	2,008	1.40	0.58	[0.02 , 1.13]	Non-inferiority not shown	0.041
[CoP] Accolade Trident	975	1.56	0.73	[0.03 , 1.44]	Non-inferiority not shown	0.042
[CoP] C-Stem Cemented Stem Marathon	344	0.69	-0.14	[-0.92 , 0.64]	Non-inferiority not shown	0.726
[CoP] C-Stem Cemented Stem Wroblewski Golf Ball	273	0.31	-0.52	[-1.21 , 0.17]	Non-inferiority not shown	0.142
[CoP] CPT Trilogy	274	1.73	0.90	[-0.14 , 1.94]	Non-inferiority not shown	0.089
[CoP] Corail Marathon	368	1.84	1.02	[-0.12 , 2.15]	Non-inferiority not shown	0.079
[CoP] Corail Pinnacle	2,678	0.94	0.11	[-0.33 , 0.54]	Non-inferiority not shown	0.630
[CoP] Exeter V40 Charnley and Elite Plus LPW	275	1.16	0.33	[-0.85 , 1.52]	Non-inferiority not shown	0.585
[CoP] Exeter V40 Elite Plus Ogee	420	0.56	-0.27	[-0.99 , 0.45]	Non-inferiority not shown	0.467
[CoP] Exeter V40 Exeter Contemporary Flanged	919	0.88	0.06	[-0.57 , 0.68]	Non-inferiority not shown	0.862
[CoP] Exeter V40 Exeter X3 Rimfit	649	1.27	0.44	[-0.27 , 1.15]	Non-inferiority not shown	0.225
[CoP] Exeter V40 Trilogy	599	1.36	0.53	[-0.42 , 1.48]	Non-inferiority not shown	0.274
[CoP] Furlong HAC Stem CSF	1,990	0.93	0.11	[-0.43 , 0.64]	Non-inferiority not shown	0.699
[CoP] Furlong HAC Stem Furlong HAC CSF Plus	563	1.40	0.58	[-0.29 , 1.44]	Non-inferiority not shown	0.193
[CoP] MS-30 Original ME Muller Low Profile Cup	375	0.21	-0.62	[-1.16 , -0.07]	Non-inferior	0.026
[CoP] SL-Plus Cementless Stem EP-Fit Plus	268	1.45	0.62	[-0.83 , 2.07]	Non-inferiority not shown	0.404
[CoP] Taperloc Cementless Stem Exceed ABT	601	1.04	0.21	[-0.48 , 0.89]	Non-inferiority not shown	0.552
[MoP] Accolade Trident	2,467	2.30	1.47	[0.83 , 2.12]	Inferior by ≥100%	<0.001
[MoP] Anthology R3 Cementless	292	2.08	1.25	[0.01 , 2.49]	Non-inferiority not shown	0.048
[MoP] C-Stem AMT Cemented Stem Charnley and Elite Plus LPW	419	1.68	0.85	[-0.36 , 2.06]	Non-inferiority not shown	0.167
[MoP] C-Stem AMT Cemented Stem Marathon	256	1.18	0.35	[-0.62 , 1.32]	Non-inferiority not shown	0.475
[MoP] C-Stem AMT Cemented Stem Pinnacle	322	1.15	0.32	[-0.55 , 1.19]	Non-inferiority not shown	0.474
[MoP] C-Stem Cemented Stem Charnley and Elite Plus LPW	322	1.16	0.34	[-0.85 , 1.52]	Non-inferiority not shown	0.579
[MoP] C-Stem Cemented Stem Elite Plus Ogee	830	1.26	0.43	[-0.36 , 1.23]	Non-inferiority not shown	0.283

[MoP] C-Stem Cemented Stem Marathon	418	1.27	0.44	[-0.53 , 1.41]	Non-inferiority not shown	0.372
[MoP] C-Stem Cemented Stem Opera	391	1.47	0.64	[-0.58 , 1.86]	Non-inferiority not shown	0.301
[MoP] CPT Elite Plus Ogee	550	1.47	0.64	[-0.37 , 1.65]	Non-inferiority not shown	0.217
[MoP] CPT Trilogy	1,745	1.63	0.80	[0.18 , 1.42]	Inferior by ≥20%	0.012
[MoP] CPT ZCA	1,031	1.26	0.43	[-0.25 , 1.12]	Non-inferiority not shown	0.213
[MoP] Charnley Cemented Stem Charnley Cemented Cup	1,058	1.60	0.77	[-0.04 , 1.58]	Non-inferiority not shown	0.063
[MoP] Charnley Cemented Stem Charnley Ogee	2,239	1.38	0.55	[-0.03 , 1.13]	Non-inferiority not shown	0.064
[MoP] Charnley Cemented Stem Charnley and Elite Plus LPW	1,040	0.88	0.05	[-0.59 , 0.69]	Non-inferiority not shown	0.886
[MoP] Charnley Cemented Stem Opera	280	1.36	0.53	[-0.84 , 1.90]	Non-inferiority not shown	0.446
[MoP] Corail Duraloc Cementless Cup	978	1.96	1.13	[0.21 , 2.05]	Inferior by ≥20%	0.016
[MoP] Corail Elite Plus Ogee	308	1.86	1.03	[-0.38 , 2.44]	Non-inferiority not shown	0.153
[MoP] Corail Marathon	645	1.37	0.54	[-0.27 , 1.35]	Non-inferiority not shown	0.188
[MoP] Corail Pinnacle	7,845	1.52	0.69	[0.28 , 1.11]	Inferior by ≥20%	0.001
[MoP] Corail Trilogy	517	1.76	0.93	[-0.20 , 2.07]	Non-inferiority not shown	0.108
[MoP] Exeter V40 Cenator Cemented Cup	395	1.46	0.63	[-0.58 , 1.84]	Non-inferiority not shown	0.307
[MoP] Exeter V40 Charnley and Elite Plus LPW	397	1.70	0.87	[-0.35 , 2.09]	Non-inferiority not shown	0.164
[MoP] Exeter V40 Duraloc Cementless Cup	265	2.54	1.71	[-0.18 , 3.60]	Non-inferiority not shown	0.076
[MoP] Exeter V40 Elite Plus Cemented Cup	749	0.95	0.12	[-0.62 , 0.86]	Non-inferiority not shown	0.748
[MoP] Exeter V40 Elite Plus Ogee	3,262	1.05	0.22	[-0.26 , 0.69]	Non-inferiority not shown	0.369
[MoP] Exeter V40 Exeter Contemporary Flanged	8,180	1.18	0.35	[-0.05 , 0.75]	Non-inferiority not shown	0.090
[MoP] Exeter V40 Exeter Contemporary Hooded	2,960	1.74	0.91	[0.36 , 1.45]	Inferior by ≥20%	0.001
[MoP] Exeter V40 Exeter Duration	2,482	1.53	0.70	[0.13 , 1.27]	Non-inferiority not shown	0.016
[MoP] Exeter V40 Exeter X3 Rimfit	1,213	1.48	0.65	[0.03 , 1.28]	Non-inferiority not shown	0.039
[MoP] Exeter V40 Marathon	261	0.86	0.03	[-0.80 , 0.85]	Non-inferiority not shown	0.946
[MoP] Exeter V40 Opera	414	0.71	-0.12	[-0.99 , 0.75]	Non-inferiority not shown	0.785
[MoP] Exeter V40 Pinnacle	462	1.75	0.92	[-0.12 , 1.97]	Non-inferiority not shown	0.084
[MoP] Exeter V40 Reflection Cementless	467	1.44	0.61	[-0.50 , 1.72]	Non-inferiority not shown	0.284
[MoP] Exeter V40 Trident	4,119	1.23	0.40	[-0.04 , 0.85]	Non-inferiority not shown	0.074
[MoP] Exeter V40 Trilogy	2,325	1.03	0.20	[-0.32 , 0.71]	Non-inferiority not shown	0.455
[MoP] Furlong Cemented Stem JRI Cemented Cup	250	1.52	0.69	[-0.83 , 2.21]	Non-inferiority not shown	0.371
[MoP] Furlong HAC Stem CSF	1,718	1.67	0.84	[0.17 , 1.51]	Inferior by ≥20%	0.014
[MoP] Furlong HAC Stem Furlong HAC CSF Plus	689	2.04	1.21	[0.27 , 2.14]	Inferior by ≥20%	0.011
[MoP] M/L Taper Cementless Allofit	280	1.41	0.58	[-0.69 , 1.85]	Non-inferiority not shown	0.373
[MoP] Muller-Biomet Apollo	460	1.24	0.41	[-0.63 , 1.46]	Non-inferiority not shown	0.438
[MoP] Polarstem Cementless R3 Cementless	330	1.22	0.39	[-0.35 , 1.13]	Non-inferiority not shown	0.302
[MoP] SL-Plus Cementless Stem EP-Fit Plus	642	3.41	2.58	[1.24 , 3.91]	Inferior by ≥100%	<0.001
[MoP] Stanmore Modular Stem Stanmore-Arcom Cup	546	0.90	0.07	[-0.72 , 0.87]	Non-inferiority not shown	0.856
[MoP] Synergy Cementless Stem R3 Cementless	318	1.21	0.38	[-0.54 , 1.29]	Non-inferiority not shown	0.416
[MoP] Synergy Cementless Stem Reflection Cementless	439	1.54	0.71	[-0.47 , 1.89]	Non-inferiority not shown	0.239
[MoP] Taperloc Cementless Stem Exceed ABT	971	1.32	0.49	[-0.18 , 1.17]	Non-inferiority not shown	0.154
[MoP] Versys Cementless Stem Trilogy	282	3.38	2.55	[0.46 , 4.64]	Inferior by ≥20%	0.017

Supplemental table 8b: Difference in Kaplan-Meier failure estimate between a contemporary reference and implants with at least 250 at risk at 5 years since primary in males between 55 and 75 years

Stem/cup brand	Number at risk	Cumulative failure (%)	Difference in failure (%)	95% CI	Equivalence status	p-value
[CoP] Furlong HAC Stem CSF	1,759	1.09	[REFERENCE]			
[CoC] ABG II Monolithic Cementless Stem ABG II Cementless Cup	392	2.82	1.73	[0.09 , 3.37]	Non-inferiority not shown	0.038
[CoC] ABG II Monolithic Cementless Stem Trident	306	3.89	2.80	[0.74 , 4.85]	Inferior by ≥20%	0.008
[CoC] Accolade Trident	1,624	2.87	1.77	[0.94 , 2.60]	Inferior by ≥20%	<0.001
[CoC] Corail Duraloc Option	303	2.50	1.41	[-0.36 , 3.18]	Non-inferiority not shown	0.119
[CoC] Corail Pinnacle	5,229	2.37	1.28	[0.74 , 1.82]	Inferior by ≥20%	<0.001
[CoC] Exeter V40 ABG II Cementless Cup	269	1.15	0.06	[-1.16 , 1.28]	Non-inferiority not shown	0.924
[CoC] Exeter V40 Trident	2,279	1.74	0.65	[0.00 , 1.29]	Non-inferiority not shown	0.049
[CoC] Furlong HAC Stem CSF	438	3.80	2.70	[0.93 , 4.48]	Inferior by ≥20%	0.003
[CoC] Furlong HAC Stem Furlong HAC CSF Plus	1,705	1.86	0.76	[0.13 , 1.40]	Non-inferiority not shown	0.018
[CoC] SL-Plus Cementless Stem EP-Fit Plus	367	5.89	4.79	[2.51 , 7.08]	Inferior by ≥100%	<0.001
[CoC] Taperloc Cementless Stem Exceed ABT	1,161	1.61	0.52	[-0.15 , 1.18]	Non-inferiority not shown	0.127
[CoP] Accolade Trident	422	1.94	0.85	[-0.03 , 1.72]	Non-inferiority not shown	0.057
[CoP] Corail Pinnacle	1,340	1.58	0.48	[-0.14 , 1.11]	Non-inferiority not shown	0.131
[CoP] Exeter V40 Elite Plus Ogee	294	0.88	-0.21	[-1.21 , 0.79]	Non-inferiority not shown	0.680
[CoP] Exeter V40 Exeter Contemporary Flanged	635	0.88	-0.21	[-0.90 , 0.48]	Non-inferiority not shown	0.548
[CoP] Exeter V40 Trident	670	0.97	-0.12	[-0.75 , 0.51]	Non-inferiority not shown	0.701
[CoP] Exeter V40 Trilogy	492	1.71	0.62	[-0.48 , 1.72]	Non-inferiority not shown	0.272
[CoP] Furlong HAC Stem Furlong HAC CSF Plus	327	1.60	0.51	[-0.48 , 1.50]	Non-inferiority not shown	0.313
[CoP] MS-30 Original ME Muller Low Profile Cup	267	0.21	-0.88	[-1.49 , -0.27]	Non-inferior	0.005
[MoP] Accolade Trident	1,703	3.33	2.24	[1.42 , 3.05]	Inferior by ≥100%	<0.001
[MoP] C-Stem AMT Cemented Stem Charnley and Elite Plus LPW	283	1.68	0.58	[-0.65 , 1.82]	Non-inferiority not shown	0.355
[MoP] C-Stem Cemented Stem Charnley and Elite Plus LPW	296	1.16	0.07	[-1.15 , 1.29]	Non-inferiority not shown	0.911
[MoP] C-Stem Cemented Stem Elite Plus Ogee	639	1.26	0.17	[-0.67 , 1.01]	Non-inferiority not shown	0.696
[MoP] C-Stem Cemented Stem Opera	288	2.29	1.20	[-0.35 , 2.75]	Non-inferiority not shown	0.129
[MoP] CPT Elite Plus Ogee	426	2.12	1.02	[-0.25 , 2.30]	Non-inferiority not shown	0.116
[MoP] CPT Trilogy	1,247	2.23	1.14	[0.35 , 1.92]	Inferior by ≥20%	0.005
[MoP] CPT ZCA	825	1.91	0.81	[-0.09 , 1.71]	Non-inferiority not shown	0.076
[MoP] Charnley Cemented Stem Charnley Cemented Cup	942	2.86	1.76	[0.67 , 2.85]	Inferior by ≥20%	0.002
[MoP] Charnley Cemented Stem Charnley Ogee	1,978	2.45	1.35	[0.58 , 2.13]	Inferior by ≥20%	0.001
[MoP] Charnley Cemented Stem Charnley and Elite Plus LPW	929	1.28	0.19	[-0.62 , 0.99]	Non-inferiority not shown	0.650
[MoP] Corail Duraloc Cementless Cup	923	2.79	1.69	[0.58 , 2.80]	Inferior by ≥20%	0.003
[MoP] Corail Marathon	326	1.77	0.67	[-0.34 , 1.69]	Non-inferiority not shown	0.193
[MoP] Corail Pinnacle	4,602	1.85	0.76	[0.24 , 1.28]	Inferior by ≥20%	0.004
[MoP] Corail Trilogy	393	2.43	1.34	[-0.05 , 2.72]	Non-inferiority not shown	0.059
[MoP] Exeter V40 Cenator Cemented Cup	337	1.97	0.87	[-0.55 , 2.30]	Non-inferiority not shown	0.229
[MoP] Exeter V40 Charnley and Elite Plus LPW	317	1.70	0.60	[-0.65 , 1.86]	Non-inferiority not shown	0.347
[MoP] Exeter V40 Duraloc Cementless Cup	252	2.92	1.83	[-0.22 , 3.87]	Non-inferiority not shown	0.080
[MoP] Exeter V40 Elite Plus Cemented Cup	583	1.39	0.30	[-0.64 , 1.23]	Non-inferiority not shown	0.533

[MoP] Exeter V40 Elite Plus Ogee	2,569	1.35	0.26	[-0.33 , 0.85]	Non-inferiority not shown	0.390
[MoP] Exeter V40 Exeter Contemporary Flanged	5,599	1.66	0.56	[0.04 , 1.08]	Non-inferiority not shown	0.033
[MoP] Exeter V40 Exeter Contemporary Hooded	2,246	2.26	1.17	[0.50 , 1.84]	Inferior by ≥20%	0.001
[MoP] Exeter V40 Exeter Duration	1,970	2.29	1.20	[0.47 , 1.92]	Inferior by ≥20%	0.001
[MoP] Exeter V40 Opera	298	1.53	0.43	[-0.86 , 1.73]	Non-inferiority not shown	0.512
[MoP] Exeter V40 Pinnacle	305	2.50	1.40	[0.04 , 2.77]	Non-inferiority not shown	0.044
[MoP] Exeter V40 Reflection Cementless	426	2.31	1.22	[-0.21 , 2.64]	Non-inferiority not shown	0.094
[MoP] Exeter V40 Trident	2,530	1.68	0.59	[0.02 , 1.16]	Non-inferiority not shown	0.044
[MoP] Exeter V40 Trilogy	1,851	1.21	0.12	[-0.50 , 0.74]	Non-inferiority not shown	0.703
[MoP] Furlong HAC Stem CSF	1,448	1.97	0.88	[0.11 , 1.65]	Non-inferiority not shown	0.026
[MoP] Furlong HAC Stem Furlong HAC CSF Plus	430	3.06	1.97	[0.70 , 3.23]	Inferior by ≥20%	0.002
[MoP] Muller-Biomet Apollo	375	1.47	0.37	[-0.80 , 1.54]	Non-inferiority not shown	0.531
[MoP] SL-Plus Cementless Stem EP-Fit Plus	554	4.71	3.62	[2.00 , 5.24]	Inferior by ≥100%	<0.001
[MoP] Stanmore Modular Stem Stanmore-Arcom Cup	434	1.51	0.41	[-0.67 , 1.50]	Non-inferiority not shown	0.455
[MoP] Synergy Cementless Stem Reflection Cementless	407	2.46	1.37	[-0.14 , 2.87]	Non-inferiority not shown	0.075
[MoP] Taperloc Cementless Stem Exceed ABT	448	1.64	0.55	[-0.32 , 1.41]	Non-inferiority not shown	0.214
[MoP] Versys Cementless Stem Trilogy	261	3.73	2.64	[0.43 , 4.85]	Inferior by ≥20%	0.019

Supplemental table 8c: Difference in Kaplan-Meier failure estimate between a contemporary reference and implants with at least 250 at risk at 7 years since primary in males between 55 and 75 years

Stem/cup brand	Number at risk	Cumulative failure (%)	Difference in failure (%)	95% CI	Equivalence status	p-value
[CoP] Furlong HAC Stem CSF	1,463	1.34	[REFERENCE]			
[CoC] ABG II Monolithic Cementless Stem ABG II Cementless Cup	345	3.88	2.53	[0.60 , 4.47]	Inferior by ≥20%	0.010
[CoC] Accolade Trident	907	3.15	1.81	[0.90 , 2.71]	Inferior by ≥20%	<0.001
[CoC] Corail Pinnacle	2,130	2.66	1.32	[0.70 , 1.94]	Inferior by ≥20%	<0.001
[CoC] Exeter V40 Trident	1,626	2.00	0.66	[-0.06 , 1.38]	Non-inferiority not shown	0.074
[CoC] Furlong HAC Stem CSF	415	4.02	2.68	[0.84 , 4.53]	Inferior by ≥20%	0.004
[CoC] Furlong HAC Stem Furlong HAC CSF Plus	674	2.13	0.79	[0.06 , 1.51]	Non-inferiority not shown	0.034
[CoC] SL-Plus Cementless Stem EP-Fit Plus	322	6.44	5.09	[2.69 , 7.50]	Inferior by ≥100%	<0.001
[CoC] Taperloc Cementless Stem Exceed ABT	469	1.99	0.65	[-0.20 , 1.49]	Non-inferiority not shown	0.132
[CoP] Corail Pinnacle	480	2.24	0.90	[-0.00 , 1.80]	Non-inferiority not shown	0.051
[CoP] Exeter V40 Exeter Contemporary Flanged	335	0.88	-0.46	[-1.19 , 0.27]	Non-inferiority not shown	0.218
[CoP] Exeter V40 Trident	362	1.17	-0.17	[-0.95 , 0.61]	Non-inferiority not shown	0.668
[CoP] Exeter V40 Trilogy	350	1.92	0.57	[-0.62 , 1.77]	Non-inferiority not shown	0.347
[MoP] Accolade Trident	787	3.94	2.60	[1.64 , 3.55]	Inferior by ≥100%	<0.001
[MoP] C-Stem Cemented Stem Charnley and Elite Plus LPW	272	1.16	-0.18	[-1.42 , 1.07]	Non-inferiority not shown	0.779
[MoP] C-Stem Cemented Stem Elite Plus Ogee	494	1.45	0.11	[-0.84 , 1.06]	Non-inferiority not shown	0.818
[MoP] CPT Elite Plus Ogee	325	2.35	1.01	[-0.37 , 2.38]	Non-inferiority not shown	0.151
[MoP] CPT Trilogy	815	2.41	1.06	[0.21 , 1.92]	Non-inferiority not shown	0.015
[MoP] CPT ZCA	599	2.69	1.35	[0.23 , 2.46]	Non-inferiority not shown	0.018
[MoP] Charnley Cemented Stem Charnley Cemented Cup	813	3.75	2.41	[1.14 , 3.68]	Inferior by ≥20%	<0.001
[MoP] Charnley Cemented Stem Charnley Ogee	1,632	3.68	2.34	[1.39 , 3.29]	Inferior by ≥100%	<0.001
[MoP] Charnley Cemented Stem Charnley and Elite Plus LPW	776	1.98	0.64	[-0.37 , 1.64]	Non-inferiority not shown	0.212
[MoP] Corail Duraloc Cementless Cup	750	3.84	2.50	[1.18 , 3.82]	Inferior by ≥20%	<0.001
[MoP] Corail Pinnacle	2,172	2.41	1.07	[0.44 , 1.70]	Inferior by ≥20%	0.001
[MoP] Corail Trilogy	254	3.58	2.24	[0.45 , 4.03]	Inferior by ≥20%	0.014
[MoP] Exeter V40 Cenator Cemented Cup	263	2.66	1.32	[-0.40 , 3.04]	Non-inferiority not shown	0.134
[MoP] Exeter V40 Elite Plus Cemented Cup	400	1.39	0.05	[-0.92 , 1.02]	Non-inferiority not shown	0.920
[MoP] Exeter V40 Elite Plus Ogee	1,861	1.79	0.45	[-0.24 , 1.14]	Non-inferiority not shown	0.203
[MoP] Exeter V40 Exeter Contemporary Flanged	3,658	2.15	0.81	[0.21 , 1.42]	Non-inferiority not shown	0.008
[MoP] Exeter V40 Exeter Contemporary Hooded	1,560	2.93	1.58	[0.78 , 2.38]	Inferior by ≥20%	<0.001
[MoP] Exeter V40 Exeter Duration	1,396	3.26	1.92	[1.02 , 2.82]	Inferior by ≥20%	<0.001
[MoP] Exeter V40 Reflection Cementless	339	2.55	1.21	[-0.31 , 2.73]	Non-inferiority not shown	0.117
[MoP] Exeter V40 Trident	1,455	1.83	0.48	[-0.16 , 1.12]	Non-inferiority not shown	0.140
[MoP] Exeter V40 Trilogy	1,299	1.71	0.37	[-0.38 , 1.12]	Non-inferiority not shown	0.332
[MoP] Furlong HAC Stem CSF	1,139	2.72	1.37	[0.45 , 2.30]	Inferior by ≥20%	0.004
[MoP] Muller-Biomet Apollo	277	1.47	0.13	[-1.07 , 1.32]	Non-inferiority not shown	0.835
[MoP] SL-Plus Cementless Stem EP-Fit Plus	411	6.01	4.67	[2.79 , 6.55]	Inferior by ≥100%	<0.001
[MoP] Stanmore Modular Stem Stanmore-Arcom Cup	312	2.00	0.66	[-0.64 , 1.96]	Non-inferiority not shown	0.320
[MoP] Synergy Cementless Stem Reflection Cementless	298	2.71	1.37	[-0.23 , 2.96]	Non-inferiority not shown	0.093

Supplemental table 8d: Difference in Kaplan-Meier failure estimate between a contemporary reference and implants with at least 250 at risk at 10 years since primary in males between 55 and 75 years

Stem/cup brand	Number at risk	Cumulative failure (%)	Difference in failure (%)	95% CI	Equivalence status	p-value
[MoP] Exeter V40 Exeter Contemporary Flanged	1,182	3.08	[REFERENCE]			
[CoC] ABG II Monolithic Cementless Stem ABG II Cementless Cup	257	5.45	2.38	[0.02 , 4.73]	Non-inferiority not shown	0.048
[CoC] Exeter V40 Trident	545	2.49	-0.59	[-1.46 , 0.27]	Non-inferior	0.179
[CoP] Furlong HAC Stem CSF	855	1.75	-1.32	[-2.14 , -0.51]	Non-inferior	0.001
[MoP] C-Stem Cemented Stem Elite Plus Ogee	254	2.48	-0.60	[-2.00 , 0.80]	Non-inferiority not shown	0.400
[MoP] CPT Trilogy	302	3.83	0.75	[-0.59 , 2.10]	Non-inferiority not shown	0.272
[MoP] Charnley Cemented Stem Charnley Cemented Cup	486	5.76	2.68	[1.04 , 4.31]	Inferior by ≥20%	0.001
[MoP] Charnley Cemented Stem Charnley Ogee	910	5.47	2.39	[1.21 , 3.58]	Inferior by ≥20%	<0.001
[MoP] Charnley Cemented Stem Charnley and Elite Plus LPW	500	3.21	0.13	[-1.19 , 1.45]	Non-inferiority not shown	0.846
[MoP] Corail Duraloc Cementless Cup	336	7.31	4.23	[2.19 , 6.27]	Inferior by ≥20%	<0.001
[MoP] Corail Pinnacle	545	3.39	0.31	[-0.53 , 1.15]	Non-inferiority not shown	0.468
[MoP] Exeter V40 Elite Plus Ogee	805	2.81	-0.27	[-1.18 , 0.65]	Non-inferiority not shown	0.568
[MoP] Exeter V40 Exeter Contemporary Hooded	600	4.02	0.94	[-0.09 , 1.98]	Non-inferiority not shown	0.074
[MoP] Exeter V40 Exeter Duration	642	5.21	2.14	[0.92 , 3.35]	Inferior by ≥20%	0.001
[MoP] Exeter V40 Trident	335	2.78	-0.30	[-1.32 , 0.72]	Non-inferiority not shown	0.566
[MoP] Exeter V40 Trilogy	482	3.05	-0.03	[-1.09 , 1.03]	Non-inferiority not shown	0.956
[MoP] Furlong HAC Stem CSF	483	4.63	1.55	[0.22 , 2.89]	Non-inferiority not shown	0.023

Supplemental table 9a: Difference in Kaplan-Meier failure estimate between a contemporary reference and implants with at least 250 at risk at 3 years since primary in males >75 years

Stem/cup brand	Number at risk	Cumulative failure (%)	Difference in failure (%)	95% CI	Equivalence status	p-value
[MoP] Exeter V40 Elite Plus Ogee	2,199	0.78	[REFERENCE]			
[CoC] Corail Pinnacle	540	2.35	1.57	[0.38 , 2.76]	Inferior by ≥20%	0.010
[CoC] Furlong HAC Stem Furlong HAC CSF Plus	350	2.46	1.68	[0.26 , 3.10]	Inferior by ≥20%	0.020
[CoP] Corail Pinnacle	306	1.43	0.65	[-0.25 , 1.56]	Non-inferiority not shown	0.159
[CoP] Furlong HAC Stem CSF	326	1.19	0.41	[-0.68 , 1.51]	Non-inferiority not shown	0.456
[MoP] Accolade Trident	911	2.78	2.00	[1.01 , 3.00]	Inferior by ≥100%	<0.001
[MoP] C-Stem AMT Cemented Stem Charnley and Elite Plus LPW	293	1.38	0.60	[-0.65 , 1.85]	Non-inferiority not shown	0.347
[MoP] C-Stem AMT Cemented Stem Elite Plus Ogee	265	0.56	-0.22	[-1.07 , 0.63]	Non-inferiority not shown	0.618
[MoP] C-Stem Cemented Stem Elite Plus Ogee	365	1.07	0.29	[-0.70 , 1.28]	Non-inferiority not shown	0.563
[MoP] CPT Elite Plus Ogee	287	2.08	1.30	[-0.26 , 2.87]	Non-inferiority not shown	0.103
[MoP] CPT Trilogy	894	1.93	1.15	[0.34 , 1.97]	Inferior by ≥20%	0.005
[MoP] CPT ZCA	1,080	1.70	0.93	[0.22 , 1.63]	Inferior by ≥20%	0.010
[MoP] Charnley Cemented Stem Charnley Cemented Cup	398	0.92	0.14	[-0.82 , 1.10]	Non-inferiority not shown	0.771
[MoP] Charnley Cemented Stem Charnley Ogee	959	1.32	0.54	[-0.20 , 1.28]	Non-inferiority not shown	0.154
[MoP] Charnley Cemented Stem Charnley and Elite Plus LPW	559	1.07	0.29	[-0.56 , 1.14]	Non-inferiority not shown	0.507
[MoP] Corail Duraloc Cementless Cup	280	0.64	-0.14	[-1.08 , 0.81]	Non-inferiority not shown	0.780
[MoP] Corail Marathon	317	1.78	1.00	[-0.14 , 2.14]	Non-inferiority not shown	0.084
[MoP] Corail Pinnacle	2,860	1.59	0.81	[0.33 , 1.30]	Inferior by ≥20%	0.001
[MoP] Exeter V40 Cenator Cemented Cup	301	1.13	0.35	[-0.80 , 1.51]	Non-inferiority not shown	0.547
[MoP] Exeter V40 Charnley and Elite Plus LPW	263	0.27	-0.51	[-1.13 , 0.12]	Non-inferior	0.113
[MoP] Exeter V40 Elite Plus Cemented Cup	394	0.78	0.00	[-0.83 , 0.84]	Non-inferiority not shown	0.994
[MoP] Exeter V40 Exeter Contemporary Flanged	5,295	1.03	0.25	[-0.15 , 0.65]	Non-inferiority not shown	0.221
[MoP] Exeter V40 Exeter Contemporary Hooded	2,098	1.84	1.06	[0.48 , 1.64]	Inferior by ≥20%	<0.001
[MoP] Exeter V40 Exeter Duration	1,426	1.51	0.73	[0.08 , 1.39]	Non-inferiority not shown	0.028
[MoP] Exeter V40 Exeter X3 Rimfit	537	1.21	0.43	[-0.28 , 1.13]	Non-inferiority not shown	0.237
[MoP] Exeter V40 Opera	290	1.47	0.69	[-0.63 , 2.01]	Non-inferiority not shown	0.306
[MoP] Exeter V40 Pinnacle	288	1.15	0.37	[-0.62 , 1.36]	Non-inferiority not shown	0.460
[MoP] Exeter V40 Reflection Cementless	256	2.46	1.68	[-0.15 , 3.51]	Non-inferiority not shown	0.072
[MoP] Exeter V40 Trident	2,148	1.41	0.63	[0.11 , 1.15]	Non-inferiority not shown	0.018
[MoP] Exeter V40 Trilogy	867	1.15	0.37	[-0.36 , 1.10]	Non-inferiority not shown	0.319
[MoP] Furlong HAC Stem CSF	770	1.99	1.21	[0.27 , 2.16]	Inferior by ≥20%	0.012
[MoP] Furlong HAC Stem Furlong HAC CSF Plus	449	3.06	2.28	[1.00 , 3.56]	Inferior by ≥100%	<0.001
[MoP] Muller-Biomet Apollo	276	0.96	0.18	[-0.95 , 1.31]	Non-inferiority not shown	0.755
[MoP] Stanmore Modular Stem Stanmore-Arcom Cup	510	1.01	0.23	[-0.64 , 1.09]	Non-inferiority not shown	0.610
[MoP] Taperloc Cementless Stem Exceed ABT	421	1.75	0.97	[0.01 , 1.94]	Non-inferiority not shown	0.049

Supplemental table 9b: Difference in Kaplan-Meier failure estimate between a contemporary reference and implants with at least 250 at risk at 5 years since primary in males >75 years

Stem/cup brand	Number at risk	Cumulative failure (%)	Difference in failure (%)	95% CI	Equivalence status	p-value
[MoP] Exeter V40 Elite Plus Ogee	1,490	1.10	[REFERENCE]			
[CoC] Corail Pinnacle	321	2.82	1.73	[0.35 , 3.11]	Inferior by ≥20%	0.014
[MoP] Accolade Trident	540	3.41	2.32	[1.12 , 3.51]	Inferior by ≥100%	<0.001
[MoP] C-Stem Cemented Stem Elite Plus Ogee	253	1.45	0.36	[-0.91 , 1.62]	Non-inferiority not shown	0.579
[MoP] CPT Trilogy	535	2.58	1.48	[0.46 , 2.50]	Inferior by ≥20%	0.004
[MoP] CPT ZCA	693	2.90	1.80	[0.78 , 2.83]	Inferior by ≥20%	0.001
[MoP] Charnley Cemented Stem Charnley Cemented Cup	325	0.92	-0.17	[-1.16 , 0.82]	Non-inferiority not shown	0.731
[MoP] Charnley Cemented Stem Charnley Ogee	760	1.91	0.81	[-0.12 , 1.75]	Non-inferiority not shown	0.088
[MoP] Charnley Cemented Stem Charnley and Elite Plus LPW	446	1.43	0.33	[-0.68 , 1.35]	Non-inferiority not shown	0.521
[MoP] Corail Pinnacle	1,479	1.98	0.88	[0.27 , 1.49]	Inferior by ≥20%	0.005
[MoP] Exeter V40 Elite Plus Cemented Cup	271	1.04	-0.06	[-1.06 , 0.95]	Non-inferiority not shown	0.910
[MoP] Exeter V40 Exeter Contemporary Flanged	3,050	1.33	0.24	[-0.26 , 0.74]	Non-inferiority not shown	0.350
[MoP] Exeter V40 Exeter Contemporary Hooded	1,283	2.09	1.00	[0.32 , 1.68]	Inferior by ≥20%	0.004
[MoP] Exeter V40 Exeter Duration	1,041	2.08	0.98	[0.17 , 1.80]	Non-inferiority not shown	0.018
[MoP] Exeter V40 Trident	1,100	1.57	0.48	[-0.13 , 1.09]	Non-inferiority not shown	0.125
[MoP] Exeter V40 Trilogy	592	2.07	0.97	[-0.05 , 1.99]	Non-inferiority not shown	0.063
[MoP] Furlong HAC Stem CSF	561	2.27	1.17	[0.12 , 2.22]	Non-inferiority not shown	0.029
[MoP] Stanmore Modular Stem Stanmore-Arcom Cup	358	1.20	0.11	[-0.88 , 1.09]	Non-inferiority not shown	0.834

Supplemental table 9c: Difference in Kaplan-Meier failure estimate between a contemporary reference and implants with at least 250 at risk at 7 years since primary in males >75 years

Stem/cup brand	Number at risk	Cumulative failure (%)	Difference in failure (%)	95% CI	Equivalence status	p-value
[MoP] Exeter V40 Exeter Contemporary Flanged	1,540	1.76	[REFERENCE]			
[MoP] CPT Trilogy	264	3.16	1.40	[0.20 , 2.60]	Non-inferiority not shown	0.022
[MoP] CPT ZCA	390	3.94	2.18	[0.82 , 3.55]	Inferior by ≥20%	0.002
[MoP] Charnley Cemented Stem Charnley Cemented Cup	252	1.25	-0.51	[-1.68 , 0.66]	Non-inferiority not shown	0.393
[MoP] Charnley Cemented Stem Charnley Ogee	502	2.56	0.80	[-0.32 , 1.92]	Non-inferiority not shown	0.162
[MoP] Charnley Cemented Stem Charnley and Elite Plus LPW	308	1.43	-0.33	[-1.34 , 0.68]	Non-inferiority not shown	0.520
[MoP] Corail Pinnacle	542	2.34	0.58	[-0.14 , 1.30]	Non-inferiority not shown	0.116
[MoP] Exeter V40 Elite Plus Ogee	868	1.43	-0.33	[-0.99 , 0.32]	Non-inferior	0.315
[MoP] Exeter V40 Exeter Contemporary Hooded	670	2.53	0.77	[-0.02 , 1.57]	Non-inferiority not shown	0.056
[MoP] Exeter V40 Exeter Duration	643	3.08	1.32	[0.29 , 2.36]	Non-inferiority not shown	0.012
[MoP] Exeter V40 Trident	538	2.35	0.59	[-0.28 , 1.46]	Non-inferiority not shown	0.185
[MoP] Exeter V40 Trilogy	321	2.07	0.31	[-0.71 , 1.32]	Non-inferiority not shown	0.553
[MoP] Furlong HAC Stem CSF	358	3.06	1.30	[0.01 , 2.60]	Non-inferiority not shown	0.049

Supplemental table 10a: Total number of implants at risk at each time-point by age-group and gender in implants with at least 500

	Time since primary procedure			
	**3 years**	**5 years**	**7 years**	**10 years**
Male, <55	7,977	3,749	No data	No data
Male, 55-75	79,299	49,540	27,512	6,584
Male, 75+	23,653	13,124	5,303	No data
Total Male	132,073	82,003	42,294	10,348
Female, <55	10,007	6,016	2,303	No data
Female, 55-75	136,561	91,874	52,940	15,848
Female, 75+	64,959	37,694	19,779	No data
Total Female	244,285	156,505	91,701	24,408
Total	415,608	268,809	156,138	41,908

Supplemental table 10b: Total number of implant failures at each time-point by age-group and gender in implants with at least 500 at risk

	Time since primary procedure			
	**3 years**	**5 years**	**7 years**	**10 years**
Male, <55	260	132	68	53
Male, 55-75	1,039	533	364	442
Male, 75+	293	133	86	57
Total Male	1,618	811	529	560
Female, <55	268	141	102	79
Female, 55-75	1,344	784	539	545
Female, 75+	458	255	161	120
Total Female	2,091	1,189	820	754
Total	3,733	2,009	1,359	1,325
