# Supplementary material for: Assessing the non-inferiority of prosthesis constructs used in hip replacement using data from the National Joint Registry of England, Wales, Northern Ireland and the Isle of Man: a benchmarking study
Source: BMJ Open. 2019 Apr 29;9(4):e026685. doi: 10.1136/bmjopen-2018-026685 (PMC6502009; doi:10.1136/bmjopen-2018-026685)
Supplement: Author's manuscript [file bmjopen-2018-026685.draft_revisions.pdf]

BMJ Open is committed to open peer review. As part of this commitment we make the peer review history of every article we publish publicly available.

When an article is published we post the peer reviewers' comments and the authors' responses online. We also post the versions of the paper that were used during peer review. These are the versions that the peer review comments apply to.

The versions of the paper that follow are the versions that were submitted during the peer review process. They are not the versions of record or the final published versions. They should not be cited or distributed as the published version of this manuscript.

BMJ Open is an open access journal and the full, final, typeset and author-corrected version of record of the manuscript is available on our site with no access controls, subscription charges or pay-per-view fees (http://bmjopen.bmj.com).

If you have any questions on BMJ Open's open peer review process please email info.bmjopen@bmj.com

BMJ Open

Assessing the non-inferiority of prosthesis constructs used in hip replacement using data from the National Joint Registry of England, Wales, Northern Ireland and the Isle of Man: A Benchmarking study

Journal:	*BMJ Open*
Manuscript ID	bmjopen-2018-026685
Article Type:	Research
Date Submitted by the Author:	21-Sep-2018

Complete List of Authors:	Deere, Kevin; University of Bristol, School of Social & Community Medicine<br>Whitehouse, Michael; University of Bristol, Musculoskeletal Research Unit<br>Porter, Martyn; Wrightington Hospital<br>Blom, AW; University of Bristol,<br>Sayers, Adrian; University of Bristol, Muscloskeletal Research Unit

Keywords:	hip arthroplasty, non-inferiority, National Joint Registry, benchmarking, medical devices

SCHOLARONE™ Manuscripts

For peer review only

Assessing the non-inferiority of prosthesis constructs used in hip replacement using data from the National Joint Registry of England, Wales, Northern Ireland and the Isle of Man: A Benchmarking study.

Kevin Deere, Michael R Whitehouse, Martyn L Porter, Ashley W Blom, Adrian Sayers

Musculoskeletal Research Unit, Bristol Medical School, 1st Floor Learning & Research Building, Southmead Hospital, Bristol, BS10 5NB, UK

Kevin Deere
Senior research Associate

Michael R Whitehouse
Consultant Senior Lecturer in Trauma and Orthopaedics

Ashley W Blom
Professor of Orthopaedic Surgery and Head of Translational Health Sciences

Adrian Sayers
Senior Research Fellow

National Institute for Health Research Bristol Biomedical Research Centre, University of Bristol

Michael R Whitehouse
Consultant Senior Lecturer in Trauma and Orthopaedics

Ashley W Blom
Professor of Orthopaedic Surgery and Head of Translational Health Sciences

Centre for Hip Surgery, Wrightington Hospital, Lancashire, WN6 9EP, UK

Martyn L Porter
Consultant Orthopaedic Surgeon

Bristol Medical School, Population Health Sciences, University of Bristol, Bristol, UK

Adrian Sayers
Senior Research Fellow

Corresponding author: Adrian Sayers

Musculoskeletal Research Unit,

1st Floor Learning & Research Building,

Southmead Hospital,

Bristol,

BS10 5NB, UK

Email: adrian.sayers@bristol.ac.uk

Tel: 0117 4147880

Word count: 4,021

For peer review only - http://bmjopen.bmj.com/site/about/guidelines.xhtml

ABSTRACT

Objectives To investigate the relative performance of hip prosthesis constructs as compared to the best performing prosthesis constructs and illustrate the substantial variability in performance of currently used prostheses.

Design A non-inferiority study.

Setting The National Joint Registry for England, Wales, Northern Ireland and the Isle of Man (NJR)

Participants All patients with a primary total hip replacement registered in the NJR between 1st April 2003 and 31st December 2016**.**

Main outcome measures Kaplan-Meier failure function for hip prosthesis constructs. Failure difference between best performing construct and remaining constructs.

Methods Using a non-inferiority analysis, the performance of hip prosthesis constructs by brand were compared to the best performing contemporary construct. Construct failure was estimated using the 1-Kaplan Meier method, i.e. an estimate of net failure. The difference in failure between the contemporary benchmark and all other constructs was tested.

Results Of the 4,442 constructs used, only 134 had ≥500 procedures at risk at 3 years post-surgery, 89 of which were not demonstrated to be inferior to the benchmark by at least 100% relative risk. By 10 years post-surgery there were 26 constructs with ≥500 at risk, 13 of which were not demonstrated to be inferior by at least 20% relative risk.

Even fewer constructs were not inferior to the benchmark when analysed by age and gender. At 5 years post-surgery there were 15 constructs in males and 11 in females, aged 55-75, not shown to be inferior.

Conclusions There is great variability in construct performance and the majority of constructs have not been demonstrated to be non-inferior to contemporary benchmarks. We have a duty to inform patients, clinicians and commissioners of this variability in performance.

For peer review only - http://bmjopen.bmj.com/site/about/guidelines.xhtml

ARTICLE SUMMARY

Strengths and limitations of this study

- Data collected from the largest joint registry in the world
- For the first time we have explicitly compared the performance of prosthesis constructs to a contemporary reference.
- Unambiguous presentation of data allows surgeons, patients and policy makers to directly compare commonly used prosthesis constructs to a reference construct.
- Residual and unmeasured confounding factors are likely to be present.
- The number of patients remaining at risk after extended follow up is low, and therefore the power to detect non-inferiority after extended follow up is also low.

For peer review only

INTRODUCTION

When patients are considering a hip replacement, they would be forgiven for thinking that all hip prostheses function equally.[1] However, all prostheses are not equal as evidenced by the failure of the 3M Capital hip implant, and Metal-on-Metal bearings.[2] The extent to which patients and clinicians are aware of this lack of equality is unclear.

The National Joint Registry for England, Wales, Northern Ireland and the Isle of Man (NJR) was established to identify poorly performing implants. It has not focused on identifying exceptionally well performing implants due to limitations inherent with routine data collection and interpreting data from a standpoint of cause and effect. The NJR publishes the unadjusted cumulative failure rates of the most commonly used stem and cup brand combinations used in hip replacement surgery.[3]

Therefore the role of promoting perceived good practice has been filled by other organisations such as the Orthopaedic Device Evaluation Panel (ODEP) in the UK,[4] NOV in the Netherlands,[5] and the Australian superior clinical performance program.[6] Benchmarking bodies typically attempt to provide some type of classification to describe whether an implant is functioning at an acceptable level or not.

In the absence of evidence from randomised control trials, benchmarking organisations and prostheses registries are currently the best sources of evidence for prosthesis performance. However, both registries and benchmarking bodies have limitations which make the interpretation of prosthesis, or prosthesis construct, performance difficult. The cumulative failure reported by the NJR gives an indication of implant construct performance in absolute terms, but head-to-head comparison of different constructs is difficult to estimate without more advanced statistical manipulation. The ODEP grading system is focused on individual implants rather than the constructs they form and is based on meeting an acceptable externally decided benchmark. This simple dichotomisation does not facilitate comparison between the many different prosthesis constructs being used today or illustrate the extensive variability in so called well performing prostheses.[7]

Sayers et al. recently proposed a method of comparison for joint replacement prostheses using a non-inferiority design against an external benchmark.[8] However, the primary limitation of this method is the arbitrary requirement for an externally specified benchmark.

In a non-inferiority clinical trial[9] that has failure as an outcome, two treatments (comparator and reference) can be directly compared to ensure that the comparator treatment is within a clinically acceptable range (non-inferiority margin) of the performance of the reference treatment at a

For peer review only - http://bmjopen.bmj.com/site/about/guidelines.xhtml

specified point in time.[10, 11] Therefore, standard methods for conducting non-inferiority trials could be applied in an orthopaedic benchmarking setting, assuming an appropriate comparator, non-inferiority margin, and time of interest can be identified.

Choosing an appropriate contemporary reference is difficult. There is no evidence from randomised trials that suggests any prosthesis construct outperforms all others, therefore the choice of reference is more heuristic. Patients would like to receive the best available care and clinicians would like to provide the best possible care, or at least care that it is non-inferior to the best. Therefore, the natural choice of reference against which all other prostheses should be compared is the construct with the lowest failure rate. However, in order to protect against chance, good fortune and a low observed failure rate, the construct should be used in large enough numbers to mitigate sampling variability.

As the failure rate of prostheses is known to be influenced by both age and gender, the choice of reference should reflect this specificity.[12] Whereas the selection of an appropriate time and non-inferiority margin to assess prosthesis performance is much more subjective, as is the reader's specific interest. For example, a surgeon interested in an older patient with lower life expectancy, may be interested in minimising short-term complications opposed to ensuring long-term implant survivorship.

The aim of this study is to investigate the relative performance of hip prosthesis constructs as compared to the best performing prosthesis constructs using a non-inferiority study design, and illustrate the substantial variability in performance of currently used prostheses. Stem, bearing and cup brand combinations (constructs) are examined against non-inferiority margins of 20% relative risk and 100% relative risk at 3, 5, 7 and 10 years following surgery.

For peer review only

For peer review only - http://bmjopen.bmj.com/site/about/guidelines.xhtml

METHODS

Patients and Data Sources

We identified all patients with a primary total hip replacement (THR) registered in the NJR between 1st April 2003 and 31st December 2016. All patients were consented to be included in the NJR as part of the standard NJR process.

Procedures were excluded if the patient age and gender were missing, or the National Health Service (NHS) number was untraceable and therefore mortality unknown. Procedures where the bearing surface was unknown or was not either Metal-on-Polyethylene (MoP), Ceramic-on-Polyethylene (CoP) or Ceramic-on-Ceramic (CoC) were also excluded from the analysis. Metal-on-Metal prosthesis constructs were excluded as their very high failure rates across all ages and both genders have already been demonstrated[2, 13] and their use no longer reflects contemporary practice.[7]

Patient and public involvement

Patient representatives sit on the committee structure of the National Joint Registry. The research priorities of the National Joint Registry are identified by this committee structure and approved by the patient representatives.

Statistical methods

Using a non-inferiority analysis, the performance of hip prosthesis constructs were compared to an internally identified reference group. Prosthesis construct failure was estimated using the 1-Kaplan Meier method i.e. an estimate of net failure.

Failure is defined using the first linked surgical revision; patients were censored at death or administratively censored on 31st December 2016. The difference in stratum specific failure probabilities compared to the reference were calculated at 3, 5, 7 and 10 years for all prosthesis (stem-cup) combinations, stratified by gender, and stratified by gender and age group (<55 years, 55 to 75 years, >75 years).

The difference and 95% confidence interval (CI) of the difference between the comparator prosthesis construct and the reference prosthesis construct was estimated at the specified time points. The standard error of the difference was constructed using a pooled estimate of the Greenwood Standard error,[14]

$$SE(\widehat{Diff}) = \sqrt{GSE_x^2 + GSE_{ref}^2},$$

For peer review only - http://bmjopen.bmj.com/site/about/guidelines.xhtml

and a z-test comparing the difference between the reference and test prosthesis was then constructed,

$$Z = \left(\left(\widehat{F_{test}} - \widehat{F_{ref}} \right) + \delta \right) / SE\left(\widehat{Diff}\right).$$

The stratum specific contemporaneous reference construct was selected as the stem-cup combination with the lowest failure rate with at least 1,000 patients at risk at the time point of interest.

Two non-inferiority margins were chosen to illustrate the sensitivity of the choice. The first margin was conservatively set at a 20% increase in relative risk of failure compared to the reference, in line with clinical trials using this methodology, albeit towards the upper end.[15] The second was a 100% increase in relative risk, i.e. a doubling in cumulative probability of failure, as this is an easily interpretable outcome.

Results are graphically reported for all comparator prosthesis constructs with at least 500 patients still at risk at the beginning of the time point of interest. Results are also reported in a tabular format for all comparator prosthesis constructs with at least 250 patients at risk at the beginning of the time point of interest (See supplementary tables).

Prosthesis constructs were either classified as non-inferior, inconclusive, or inferior. If the upper confidence interval is less than or equal to the 20% non-inferiority margin, the prosthesis construct was non-inferior. If the lower confidence interval of the difference was greater than the non-inferiority margin at either 20% or 100% the prosthesis construct was classed as inferior at 20% or 100% respectively. If the lower confidence limit is less than the non-inferiority margin, and the upper confidence greater than non-inferiority margin the construct was described as inconclusive, see figure 1 for graphical representation of the classification.

Sensitivity analysis

We repeated all analyses using a historic reference group, this specified the reference at 3, 5, and 7 years as the best performing stem-cup prosthesis construct at 10 years with at least 1,000 still at risk in the stratum of interest.

All analyses were carried out using Stata 14.2 (College Station, Texas, USA).

For peer review only - http://bmjopen.bmj.com/site/about/guidelines.xhtml

RESULTS

There were 890,681 primary hip replacements included in the NJR between 1st April 2003 and 31st December 2016. Following the application of the exclusion criteria defined above, 797,178 procedures were included in the final analysis, see supplementary figure 1. In total 4,442 different prosthesis constructs were used at least once. A detailed description of non-inferiority across all procedures is provided. Due to the large number of clinically relevant subdivisions and sensitivity analyses, results will be described more broadly. Constructs are described using by bearing and brand. Bearings are either ceramic (C), metal (M) or polythene (P). Brands are described listing the stem and cup combination [stem/cup].

Figures were produced for each stratification of gender, age group and time since primary. To view data at 3 years post primary for all men, men<55 years, men aged 55-75 and men>75 years see supplementary figures 2a, 2b, 2c and 2d respectively. To view data at 5 years post primary for all men, men<55 years, men aged 55-75 and men>75 years see supplementary figures 3a, 3b, 3c and 3d respectively. To view data at 7 years post primary for all men, men aged 55-75 and men>75 years see supplementary figures 4a, 4b, and 4c respectively. To view data at 10 years post primary for all men and men aged 55-75 see supplementary figures 5a and 5b respectively. To view data at 3 years post primary for all women, women<55 years, women aged 55-75 and women>75 years see supplementary figures 6a, 6b, 6c and 6d respectively. To view data at 5 years post primary for all women, women<55 years, women aged 55-75 and women>75 years see supplementary figures 7a, 7b, 7c and 7d respectively. To view data at 7 years post primary for all women, women<55 years, women aged 55-75 and women>75 years see supplementary figures 8a, 8b, 8c and 8d respectively. To view data at 10 years post primary for all women and women aged 55-75 see supplementary figures 9a and 9b respectively

Estimates for the difference in failure between the reference and comparator prosthesis constructs with ≥250 procedure at risk at the time of interest for all, and for each stratification of gender and age group were tabulated. To view data for all at 3, 5, 7 and 10 years post primary see supplementary tables 1a, 1b, 1c and 1d respectively. To view data in all women at 3, 5, 7 and 10 years post primary see supplementary tables 2a, 2b, 2c and 2d respectively. To view data for women <55 years at 3, 5 and 7 years post primary see supplementary tables 3a, 3b and 3c respectively. To view data for women between 55-75 years at 3, 5, 7 and 10 years post primary see supplementary tables 4a, 4b, 4c and 4d respectively. To view data for women >75 years at 3, 5 and 7 years post primary see supplementary tables 5a, 5b and 5c respectively. To view data in all men at 3, 5, 7 and 10 years post primary see supplementary tables 6a, 6b, 6c and 6d respectively. To view data in men <55 years at 3 and 5 years post primary see supplementary tables 7a and 7b respectively. To view

For peer review only - http://bmjopen.bmj.com/site/about/guidelines.xhtml

data in men between 55 and 75 years at 3, 5, 7 and 10 years post primary see supplementary tables 8a, 8b, 8c and 8d respectively. To view data in men >75 years at 3, 5 and 7 years post primary see supplementary tables 9a, 9b and 9c respectively. The total number of implants at risk and total implant failures at each time-point can be seen in supplementary tables 10a and 10b respectively.

Non-inferiority - All procedures

The reference prosthesis construct at 3 years was identified as the CoP MS-30/Low profile Muller. There were 1,554 procedures remaining at risk and the failure rate was 0.39% (95%CI 0.19 – 0.82). There were 134 prosthesis combinations with ≥500 procedures at risk. 90 combinations were classified as inferior to the reference by at least 20% relative risk of failure. 44 of the 90 were shown to be inferior by at least 100% relative risk (figure 2). No prosthesis constructs could be described as non-inferior.

The reference prosthesis construct at 5 years was again identified as CoP MS-30/Low profile Muller. There were 1,125 procedures remaining at risk and the failure rate was 0.55% (95%CI 0.29 – 1.08). There were 99 prosthesis constructs with ≥500 procedures at risk. 74 prosthesis constructs were classified as inferior to the reference by at least 20% relative risk of failure. 39 of the 74 were shown to be inferior by at least 100% relative risk (figure 3). No prosthesis constructs could be described as non-inferior.

The reference prosthesis constructs at 7 years was identified as the MoP Exeter V40/Elite Plus Cemented Cup. There were 1,173 procedures remaining at risk and the failure rate was 0.91% (95%CI 0.64 – 1.28). There were 69 prosthesis constructs with ≥500 procedures at risk. 48 prosthesis constructs were classified as inferior to the reference by at least 20% relative risk of failure. 20 of the 48 were shown to be inferior by at least 100% relative risk (figure 4). No prosthesis constructs could be described as non-inferior.

The reference prosthesis constructs at 10 years was identified as the MoP Exeter V40/Elite Plus Ogee. There were 3,580 procedures remaining at risk and the failure rate was 2.14% (95%CI 1.87 – 2.45). There were 26 prosthesis constructs with ≥500 procedures at risk. 12 prosthesis constructs were classified as inferior to the reference by at least 20% relative risk of failure. 1 of the 12 was shown to be inferior by at least 100% relative risk (figure 5). Two prosthesis constructs were identified as non-inferior.

Non-inferiority - Gender specific.

For peer review only - http://bmjopen.bmj.com/site/about/guidelines.xhtml

Gender specific non-inferiority analyses were also performed at 3, 5, 7, and 10 years after the primary operation.

At 3 years only a small number of prosthesis constructs demonstrated non-inferiority in comparison to the reference. Most striking is the large variability of prosthesis constructs used in females compared to males (58 different prosthesis constructs were used more than 500 times in males versus 93 in females), and the gender specific heterogeneity in performance. For example, the CoP Exeter V40/Exeter Contemporary Flanged is used as the reference at 3 years in males, yet is inferior by 20% compared to the reference in females at 3 years. A performance difference was also noted in the CoC SL-Plus cementless Stem/EP-Fit Plus between the genders. At 3 years the failure rate for this prosthesis constructs in all females was 1.75% yet in males after the same period the failure was 5.11% ($p<0.001$).

At 5 years the reference failure rate in females is less than half that in males. Whilst there are only 3 prosthesis constructs marked as 100% worse than the reference prosthesis construct in males, there are 24 prosthesis constructs that are 100% worse than the reference in females. Some prosthesis constructs have been used in large numbers despite having relatively poor performance.

At 7 years the reference failure rate in females remained less than half that of males. There were no prosthesis constructs, used in sufficient numbers, which could be described as non-inferior to the reference in both males and females. One prosthesis construct in males was at least 100% worse than the reference, whilst 14 prosthesis constructs were at least 100% worse in females.

At 10 years no prosthesis constructs were described as non-inferior to the reference in both males and females and there were no implants inferior by 100% in either males or females.

Non-inferiority- Gender and Age specific.

Sub-dividing procedures by age and gender highlights the paucity of information available pertaining to either male or female patients <55 years undergoing THR. Similarly, the volume of longer-term outcomes on patients beyond 7 years is relatively low in comparison to the number of implanted prosthesis constructs. Most strikingly is the preference for hard-on-hard bearing surfaces (such as CoC) in younger male patients (<55 years). Five of the six prosthesis constructs with at least 500 procedures at 3 years were CoC, contrasted with the vast majority of prosthesis constructs used in older male patients (≥55 years) where either MoP or CoP bearing couples were used. In addition, changes to the distribution of failure rates of prostheses become increasingly apparent. For example, the reference prosthesis construct in males less than 55 years has a 60% increase in relative failure rate compared to the reference procedure for males over 75 years old.

For peer review only - http://bmjopen.bmj.com/site/about/guidelines.xhtml

The paucity of constructs in each age/gender group which have been utilised over 500 times and for which non-inferiority to the reference prosthesis construct is demonstrated is notable. For males <55 years, only 4 prosthesis constructs (including the reference construct) meet this requirement at 3 years, 3 prosthesis constructs at 5 years and none thereafter. For women of the same age the numbers are 4 at 3 years, 3 at 5 years and 7 at 7 years, with none at 10 years. In the largest grouping, those aged between 55 and 75 years, for males, 16 prosthesis constructs meet this requirement at 5 years and only 7 at 10 years, whilst for women the number are 12 at 5 years and 9 at 10 years.

Sensitivity Analysis

Using the reference prosthesis construct at 10 years as the reference at 3, 5, and 7 years, illustrates the temporal improvements in failure and the selective trajectory tracking of new prostheses constructs. The reference construct in all procedures at 10 years is the Exeter V40/Elite Plus Ogee with a failure rate of 2.14% (95%CI 1.87 – 2.45). Only 1 prosthesis construct is non-inferior and statistically superior i.e. the Exeter V40/Elite Plus Cemented cup, but it does not have 1,000 implants at risk at 10 years and therefore is not considered to be the reference construct. At 7, 5, and 3 years, the contemporary reference has a 0.59%, a 0.55%, and 0.40% lower failure rate than the historical reference respectively. Whilst the good performance of many prosthesis constructs appear to track, some exhibit substantially variability in their relative performance at the times of interest. Specifically, the MoP Exeter V40/Charnley Ogee is non-inferior to the historical reference at 10 years, but is inferior by 20% at 5 years.

For peer review only - http://bmjopen.bmj.com/site/about/guidelines.xhtml

DISCUSSION

We have demonstrated in 797,178 primary total hip replacements the relative performance of implanted prosthesis constructs in comparison to an internally selected contemporary reference. There is substantial variation in the performance of prosthesis constructs. A non-inferiority approach to benchmarking provides an immediate comparison of commonly used implanted prosthesis constructs compared to an internal contemporary reference and conveys distinct advantages opposed to standard Kaplan-Meier analyses as currently reported in the NJR annual reports or categorical grades provided by ODEP. The heterogeneity in implanted constructs in females compared to males is aptly illustrated, as is the paucity of information in clinically relevant sub-strata. The marked differences in outcomes between the different age/gender sub-strata confirm the importance of comparing prosthesis constructs within these strata.[12] We present this study as a novel way of assessing hip prosthesis constructs and as such there is, in the authors opinion, no relevant evidence published to date.

What is most striking is that so few prosthesis constructs in each age/gender strata meet the criteria of 500 cases at each time point and are at least classified as “inferiority not shown”. Of the 4,442 constructs used, only 7 meet these criteria in males aged 55-75 years (supplementary figure 5b) and 9 in females aged 55-75 years at 10 years (supplementary figure 9b). None meet the criteria in any other age/gender sub-strata at 10 years. Even at the relatively short follow-up of 5 years, only 16 constructs in men (supplementary figure 3c) and 12 in women age 55-75 years (supplementary figure 7c) meet these criteria. Patients would have a reasonable expectation that the implants they receive have a proven track record and have not been demonstrated as having a 20% or more increased revision rate for patients of the same age and gender. It is important to note that some prosthesis constructs have a higher early relative failure rate and a lower relative failure rate in later years and thus are inferior at 3 and 5 years, but non-inferior at 7 and 10 years. Examples of this in men aged 55-75 years are the MoP Corail/Pinnacle and MoP Exeter/Contemporary hooded. Late failure is preferential to early failure from the patient, societal and health economic perspectives, particularly as revision is unfortunately associated with a high rate of re-revision.[16]

One of the most obvious trends across all stratifications is the outstanding performance of the Exeter V40 stem as part of various prosthesis constructs. However, the heterogeneity in acetabular prostheses paired with the Exeter V40 stem is substantial, as is the subsequent variation in performance. This aptly illustrates the need to benchmark constructs opposed to individual implants which make up prosthesis constructs, which has the potential to provide false reassurance in terms of efficacy as the individual elements of a construct are not independent. Patient specific construct

For peer review only - http://bmjopen.bmj.com/site/about/guidelines.xhtml

selection is another strong feature of the data, with the majority of younger patient receiving CoC bearing surfaces, whereas the majority of older patients receive MoP bearing surfaces.

This analysis does show that certain constructs are either the reference or are non-inferior to the reference prosthesis construct across almost all age and gender strata. This strongly suggests that they could appropriately be used as default options for the majority of patients. This is particularly relevant for inexperienced surgical teams, as they can focus training on, and become expert with, a single prosthesis construct. This has the potential to reduce the risk of technical error, to be cost saving through bulk purchasing arrangements and via a reduction in failure rates. The absolute level of failure of commonly used constructs is relatively low, and less than 5% in many instances. This apparently excellent (ODEP 10A*) performance is exhibited by nearly all prosthesis constructs with sufficient data at 10 years (≥500 patients at risk) and raises questions about whether an externally placed benchmark is the optimal way to ensure best care.

Encouragingly, the sensitivity analysis demonstrates the prognosis for patients undergoing hip replacement is continually improving, and the currently best performing implant at 10 years is unlikely to be as good as the contemporary references at 3, 5, and 7 years when these reach 10 years of follow up. Whilst the refinement of clinical practice and development of prosthesis constructs appears to be raising the bar in performance, it is clear that these improvements are not universal. This raises questions about how implants are introduced into a market safely; ensuring enough prosthesis constructs are implanted to ascertain their relative performance, but no more than the necessary number of prosthesis constructs are implanted to minimise the exposure of patients to poor performing implants, and finally to ensure there is sufficient incentive for implant manufacturers to develop new prostheses that benefit patients.

This analysis has a number of important strengths. We explicitly compare prosthesis constructs to a contemporary reference, and a historical defined reference with known performance using a non-inferiority study design. The unambiguous presentation of data allows surgeons, patients and policy makers to directly compare commonly used prosthesis constructs to a reference construct. We illustrate the paucity of information in clinically relevant sub-strata and the need to compare implant constructs opposed to implant elements. The analysis has a number of limitations; case-mix adjustment by stratification is difficult to assimilate despite the restricted set of confounding factors. Residual and unmeasured confounding factors are likely to be present, and the ability to interpret analyses from a causal perspective is limited. The number of patients remaining at risk after extended follow up is low, and therefore the power to detect non-inferiority assuming there is truly no difference in prosthesis constructs compared to the reference is also low. Data entry is mandated

For peer review only - http://bmjopen.bmj.com/site/about/guidelines.xhtml

and data capture is extremely high (over 95%),[17] thus the findings in this study are highly likely to be generalisable.

Conclusions, policy and future research implications

The use of product benchmarking has the potential to be highly informative for patients, change the practice of surgeons, and influence policy makers if presented clearly and unambiguously. The use of a contemporary reference prosthesis construct is essential to ensure that benchmarks are clinically relevant.

For peer review only

For peer review only - http://bmjopen.bmj.com/site/about/guidelines.xhtml

Acknowledgments

We thank the patients and staff of all the hospitals who have contributed data to the National Joint Registry. We are grateful to the Healthcare Quality Improvement Partnership, the National Joint Registry Steering Committee, and staff at the National Joint Registry for facilitating this work. The views expressed represent those of the authors and do not necessarily reflect those of the NHS, the National Institute for Health Research, the Department of Health, the National Joint Registry Steering Committee or Healthcare Quality Improvement Partnership, who do not vouch for how the information is presented.

Funding

Posts of members of the research team are funded by a contract grant from the National Joint Registry for England, Wales, Northern Ireland and the Isle of Man. This study was also supported by the NIHR Biomedical Research Centre at the University Hospitals Bristol NHS Foundation Trust and the University of Bristol. AS is funded by an MRC Strategic Skills Fellowship MR/L01226X/1.

Competing interest statement

All authors have completed the Unified Competing Interest form at www.icmje.org/coi_disclosure.pdf (available on request from the corresponding author) and declare: funding was received for the submitted work as described in the Funding statement; no financial relationships with any organisations that might have an interest in the submitted work in the previous three years, no other relationships or activities that could appear to have influenced the submitted work.

Author Contributions

AS, MRW and AWB conceived the study. KD, AS and AWB designed the study. The data were extracted by Northgate (Hemel Hempstead, UK). KD and AS managed and analysed data. MRW, AS, MLP and AWB reviewed the published work. All authors critically inputted into final design of the study, interpreted the data and co-wrote the manuscript. AS is the guarantor and attests on behalf of the authors that the manuscript is a true and transparent reflection of the study without omissions.

Role of the funding source

The sponsor of the study had no role in the study design, data collection, analysis, interpretation, or writing of the final report. KD and AS had full access to all of the data and AS had final responsibility for manuscript submission.

Ethical approval

For peer review only

For peer review only - http://bmjopen.bmj.com/site/about/guidelines.xhtml

Not required for this study. Patient data in the NJR is only available by consent of the patient.

Data sharing

Access to data is available from the National Joint Registry for England and Wales

For peer review only

For peer review only - http://bmjopen.bmj.com/site/about/guidelines.xhtml

Figures

Figure 1: Schematic representation of inferiority and non-inferiority

Figure 2: Difference in failure of implanted constructs compared to a contemporary reference at 3 years, using all stem-cup combinations with ≥500 procedures remaining at risk.

Figure 3: Difference in failure of implanted constructs compared to a contemporary reference at 5 years, using all stem-cup combinations with ≥500 procedures remaining at risk.

Figure 4: Difference in failure of implanted constructs compared to a contemporary reference at 7 years, using all stem-cup combinations with ≥500 procedures remaining at risk.

Figure 5: Difference in failure of implanted constructs compared to a contemporary reference at 10 years, using all stem-cup combinations with ≥500 procedures remaining at risk.

For peer review only

References

1. Warden J. Warning issued over hip implants. *BMJ* 1998;316(7132):650.
2. Smith AJ, Dieppe P, Howard PW, et al. Failure rates of metal-on-metal hip resurfacings: analysis of data from the National Joint Registry for England and Wales. *Lancet* 2012;380(9855):1759-66. doi: 10.1016/S0140-6736(12)60989-1
3. NJR Steering Committee. National Joint Registry for England, Wales, Northern Ireland and the Isle of Man: 13th Annual Report. Hemel Hempsted, 2016

4. Tucker K. Orthopaedic Data Evaluation Panel: ODEP. Northgate Public Services (UK) Ltd. 2017 [Available from: http://www.odep.org.uk/ODEPProcess/ODEPStatement.aspx.
5. Nederlandse Orthopaedische Vereniging. Classification Orthopaedic Implants: NOV Total Knee Prosthesis 2016-2017 [Available from: https://www.orthopeden.org/downloads/146/classificatie-knie-2017.pdf.
6. Prostheses and Devices Committee (PDC) and Prostheses List Advisory Committee (PLAC) Bulletins. Australian Government; Department of Health [Available from: http://www.health.gov.au/internet/main/publishing.nsf/Content/health-privatehealth-prostheseslist.htm.
7. NJR Reports Prosthesis document. National Joint Registry (NJR), 2016 [Available from: http://www.njrreports.org.uk/Portals/0/PDFdownloads/NJR%2013th%20AR%20Prostheses%20used%20in%20joint%20replacements%202015.pdf.
8. Sayers A, Crowther MJ, Judge A, et al. Determining the sample size required to establish whether a medical device is non-inferior to an external benchmark. *BMJ Open* 2017;7(8):e015397. doi: 10.1136/bmjopen-2016-015397 [published Online First: 2017/08/31]
9. Schumi J, Wittes JT. Through the looking glass: understanding non-inferiority. *Trials* 2011;12:106. doi: 10.1186/1745-6215-12-106
10. Tunes da Silva G, Logan BR, Klein JP. Methods for equivalence and noninferiority testing. *Biol Blood Marrow Transplant* 2009;15(1 Suppl):120-7. doi: 10.1016/j.bbmt.2008.10.004
11. Com-Nougue C, Rodary C, Patte C. How to establish equivalence when data are censored: a randomized trial of treatments for B non-Hodgkin lymphoma. *Stat Med* 1993;12(14):1353-64.
12. Bayliss LE, Culliford D, Monk AP, et al. The effect of patient age at intervention on risk of implant revision after total replacement of the hip or knee: a population-based cohort study. *Lancet* 2017;389(10077):1424-30. doi: 10.1016/S0140-6736(17)30059-4
13. Smith AJ, Dieppe P, Vernon K, et al. Failure rates of stemmed metal-on-metal hip replacements: analysis of data from the National Joint Registry of England and Wales. *Lancet* 2012;379(9822):1199-204. doi: 10.1016/S0140-6736(12)60353-5
14. Greenwood M. The natural duration of cancer. *Rep **Public Health** Med Subj (Lond)* 1926;33: 1-26
15. Donken R, de Melker HE, Rots NY, et al. Comparing vaccines: a systematic review of the use of the non-inferiority margin in vaccine trials. *Vaccine* 2015;33(12):1426-32. doi: 10.1016/j.vaccine.2015.01.072
16. Hunt LP WM, Beswick A, Porter ML, Howard P, Blom AW. Implications of the Introduction of New Technology: Comparative Survivorship Modelling of Metal-on-Metal Hip Replacement to Contemporary Alternatives in the National Joint Registry. *J Bone Joint Surg Am* 2017, *In press*
17. NJR Data Quality Audit 2017 [Available from: http://www.njrcentre.org.uk/njrcentre/NewsandEvents/DataQualityAudit-updateSeptember2017/tabid/1451/Default.aspx.

For peer review only - http://bmjopen.bmj.com/site/about/guidelines.xhtml

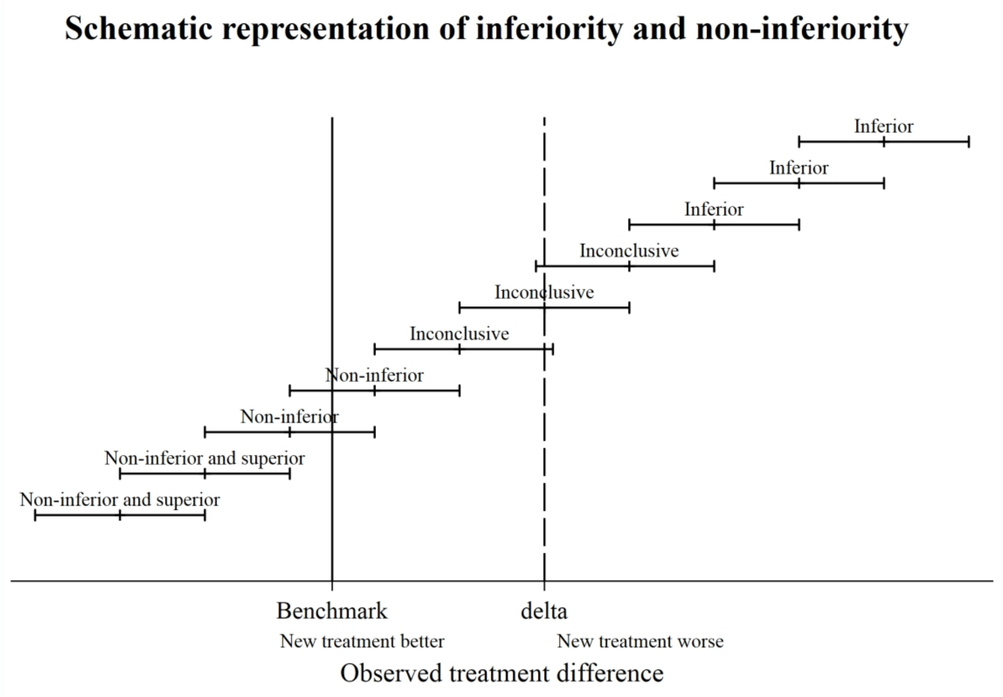

177x124mm (300 x 300 DPI)

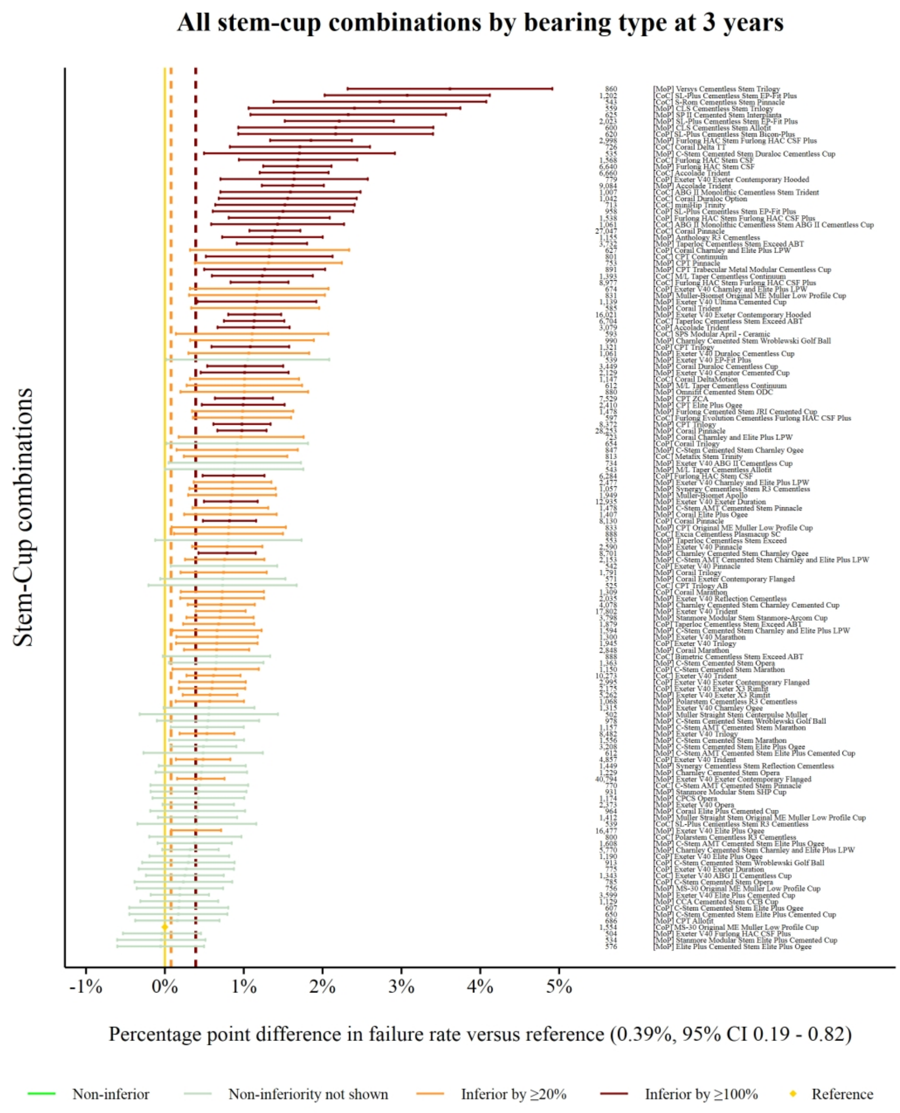

203x254mm (300 x 300 DPI)

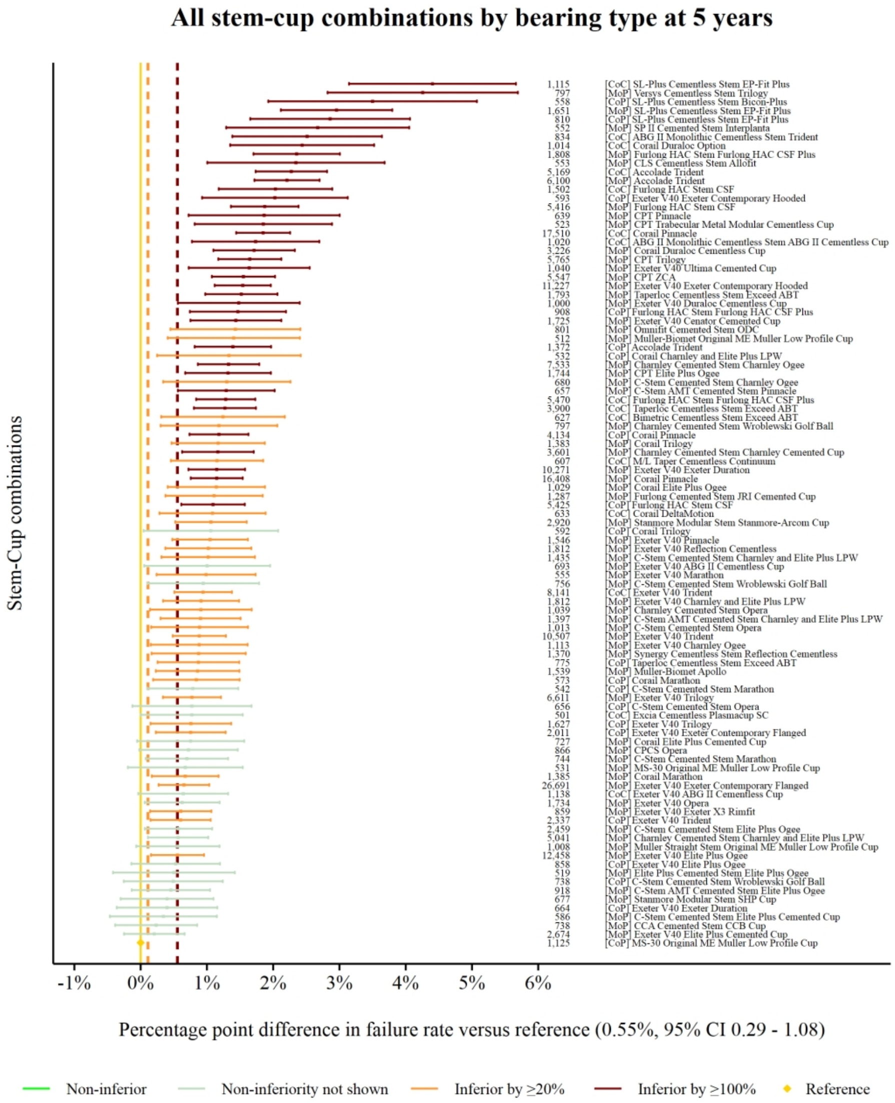

189x237mm (300 x 300 DPI)

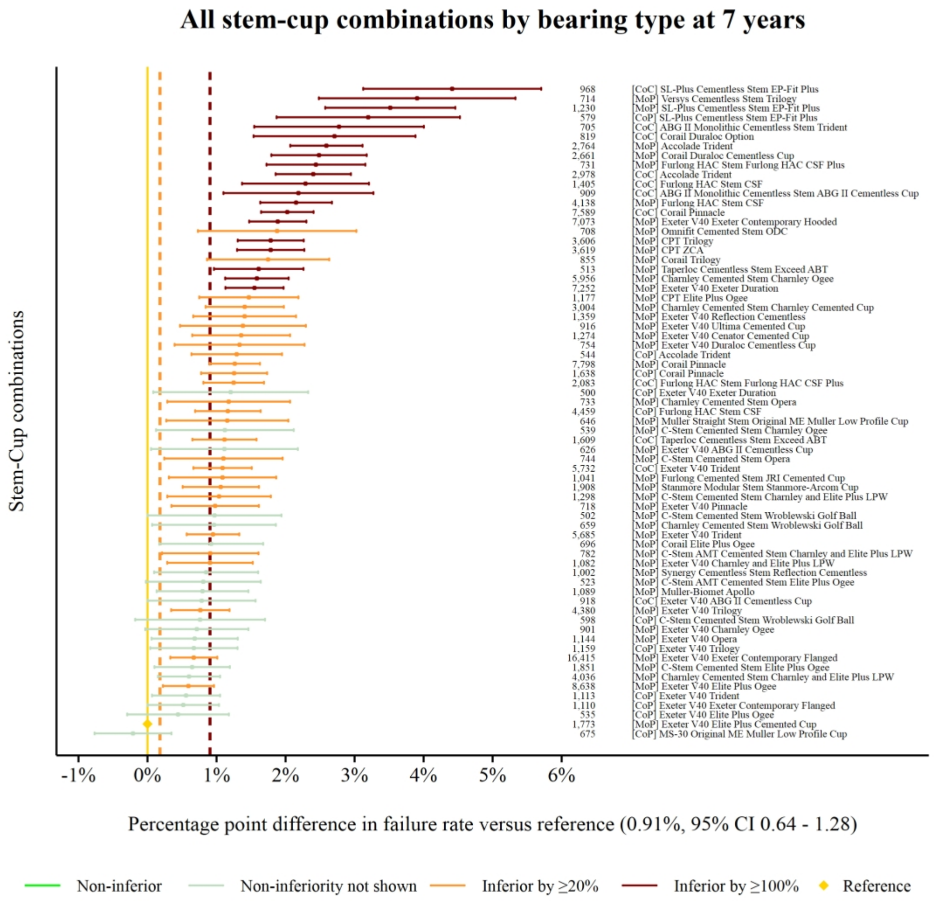

211x211mm (300 x 300 DPI)

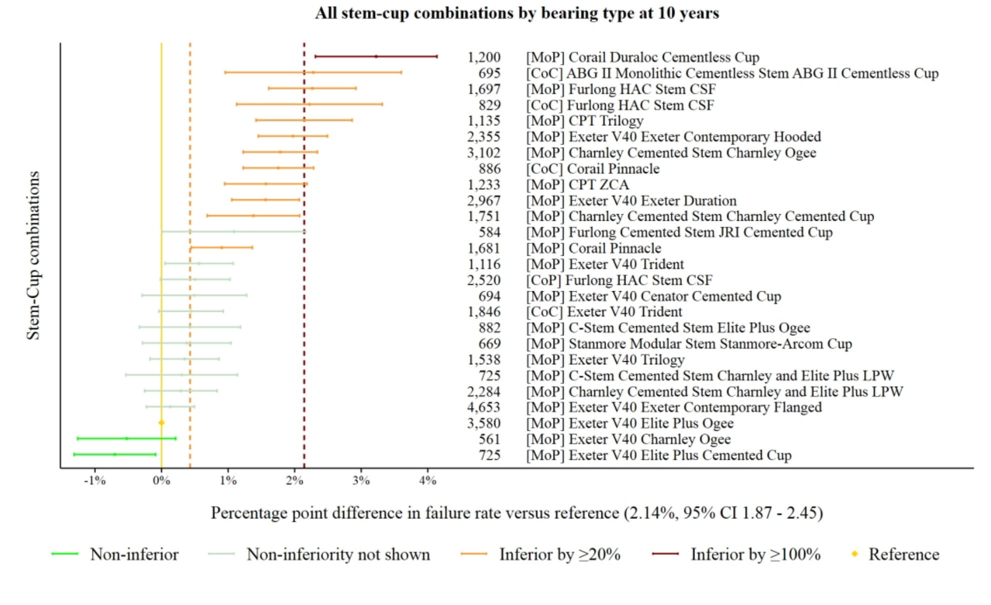

169x105mm (300 x 300 DPI)

Supplementary Figure 1: Flow diagram of showing derivation of procedures used in analyses

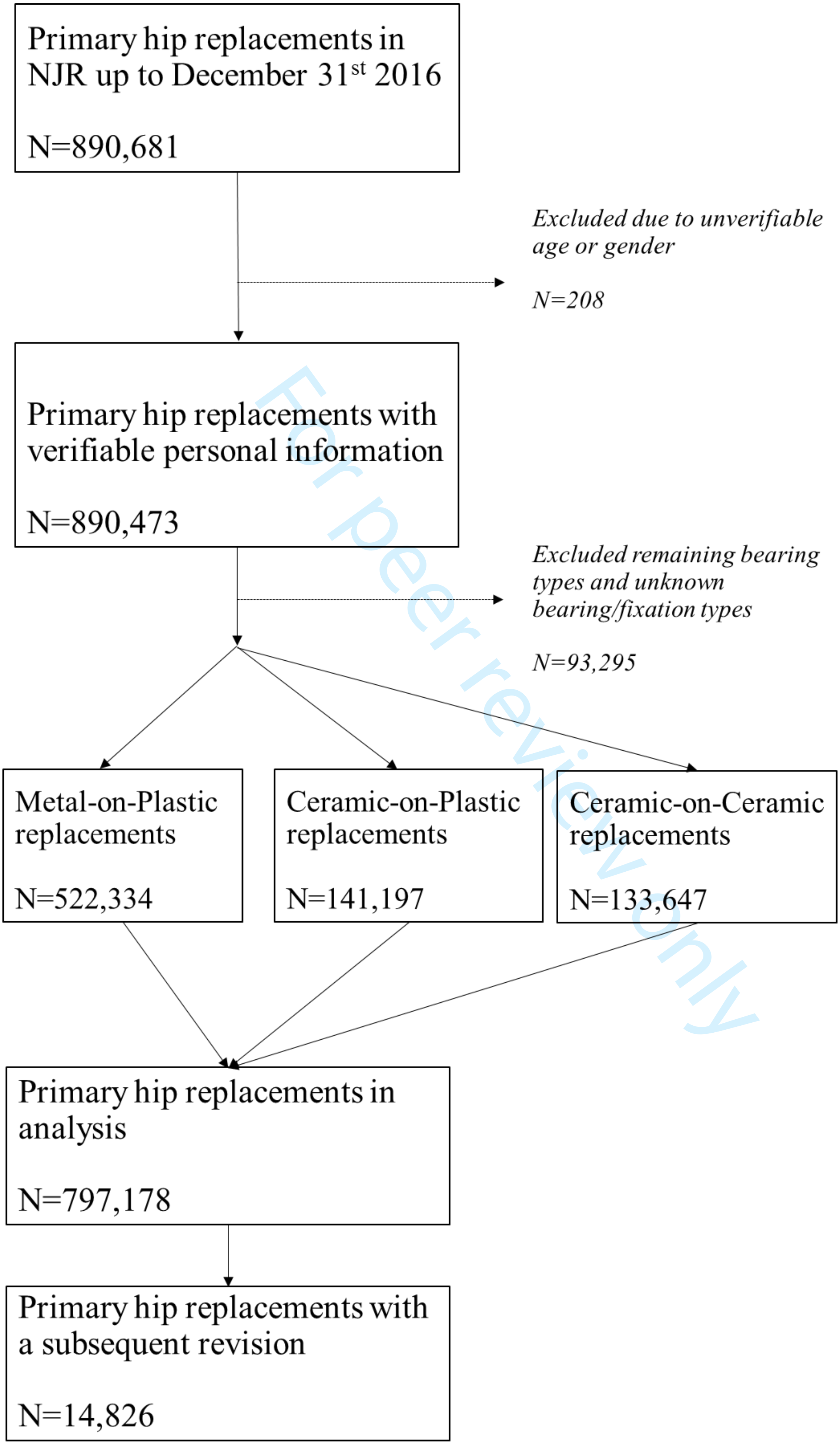

For peer review only

Supplementary Figure 2a: Difference in failure of implanted constructs compared to a contemporary reference at 3 years in men, using all stem-cup combinations with ≥500 procedures remaining at risk

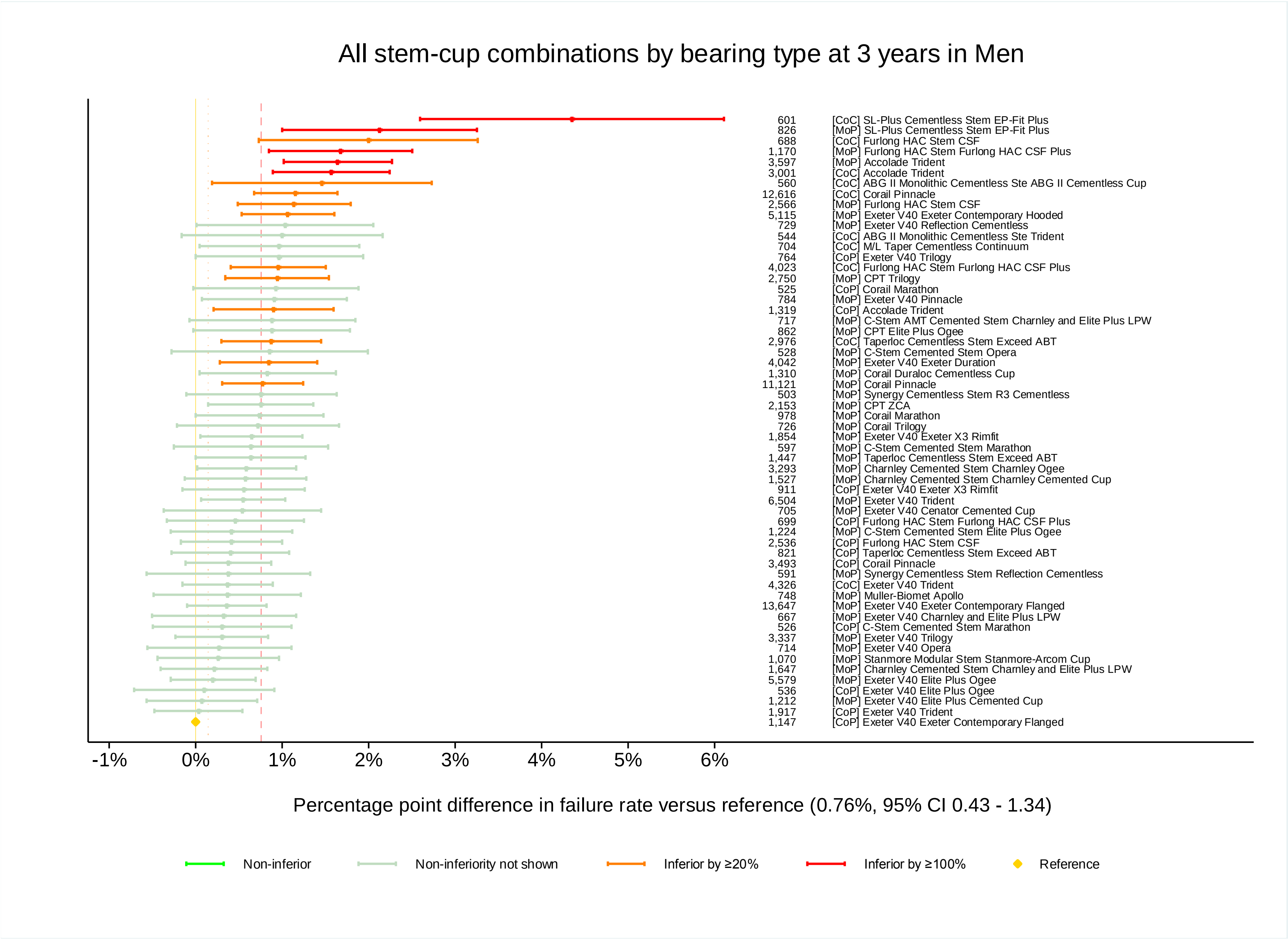

Supplementary Figure 2b: Difference in failure of implanted constructs compared to a contemporary reference at 3 years in men less than 55 years, using all stem-cup combinations with ≥500 procedures remaining at risk

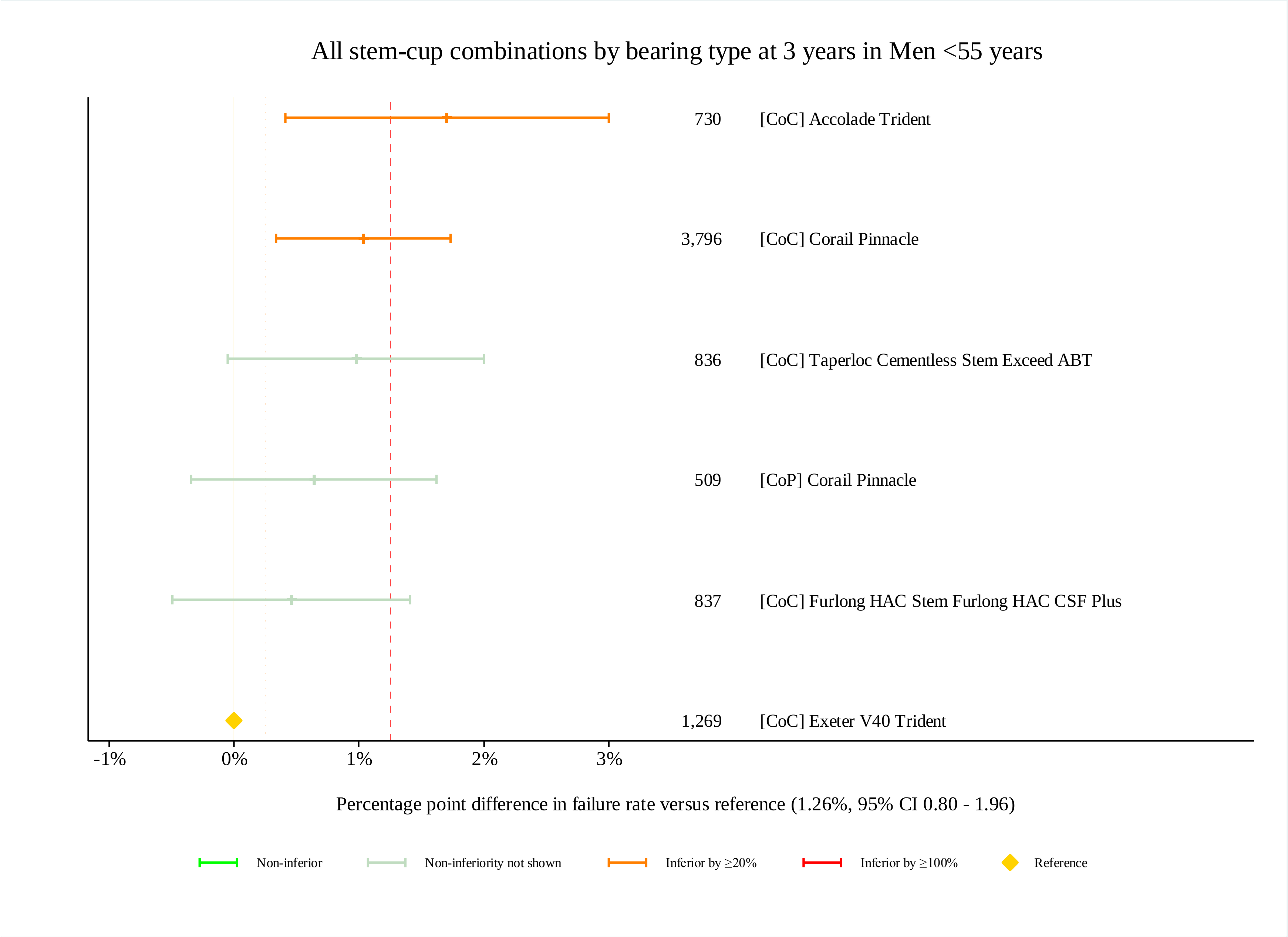

Supplementary Figure 2c: Difference in failure of implanted constructs compared to a contemporary reference at 3 years in men between 55 and 75 years, using all stem-cup combinations with ≥500 procedures remaining at risk

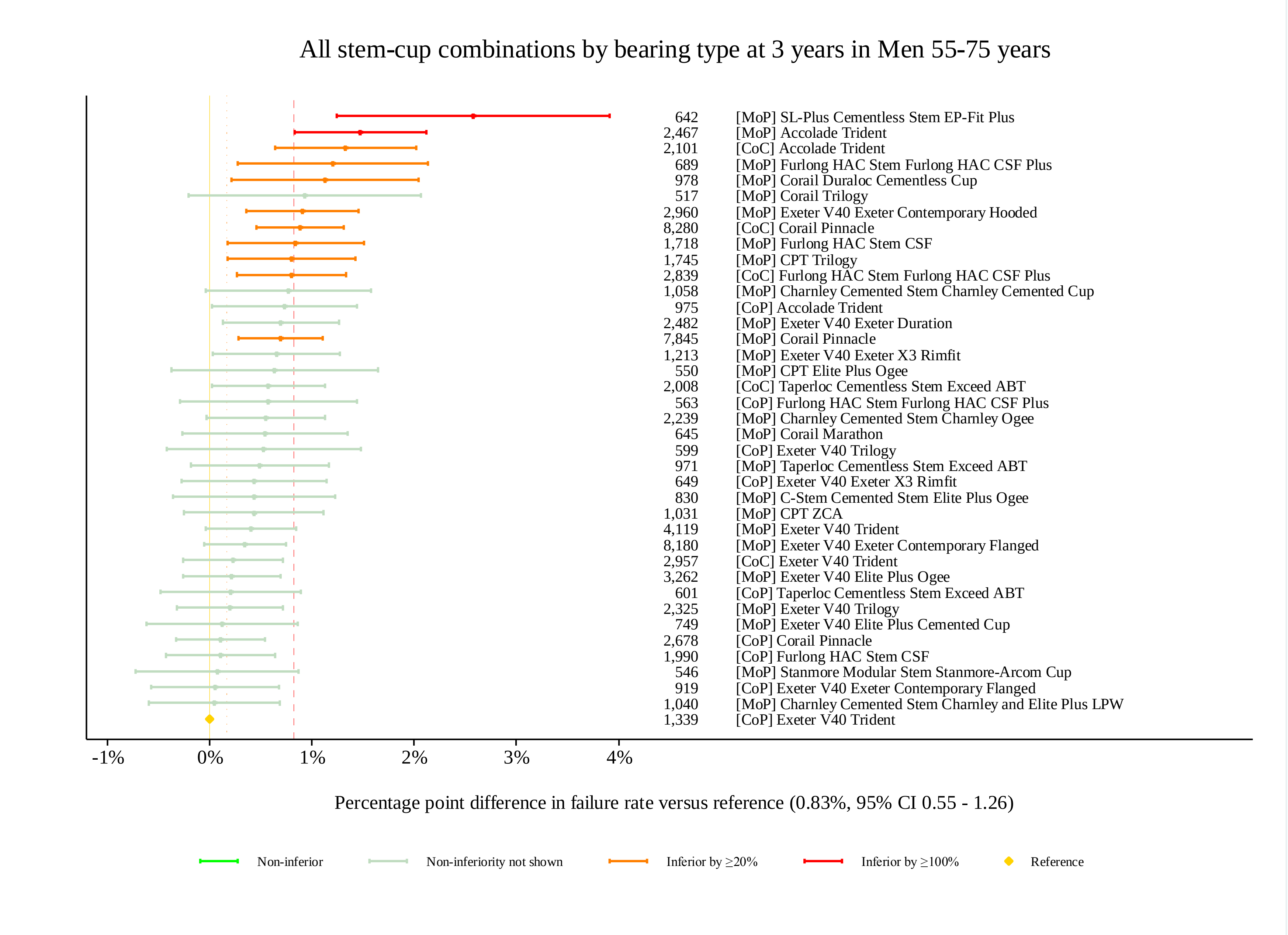

Supplementary Figure 2d: Difference in failure of implanted constructs compared to a contemporary reference at 3 years in men greater than 75 years, using all stem-cup combinations with ≥500 procedures remaining at risk

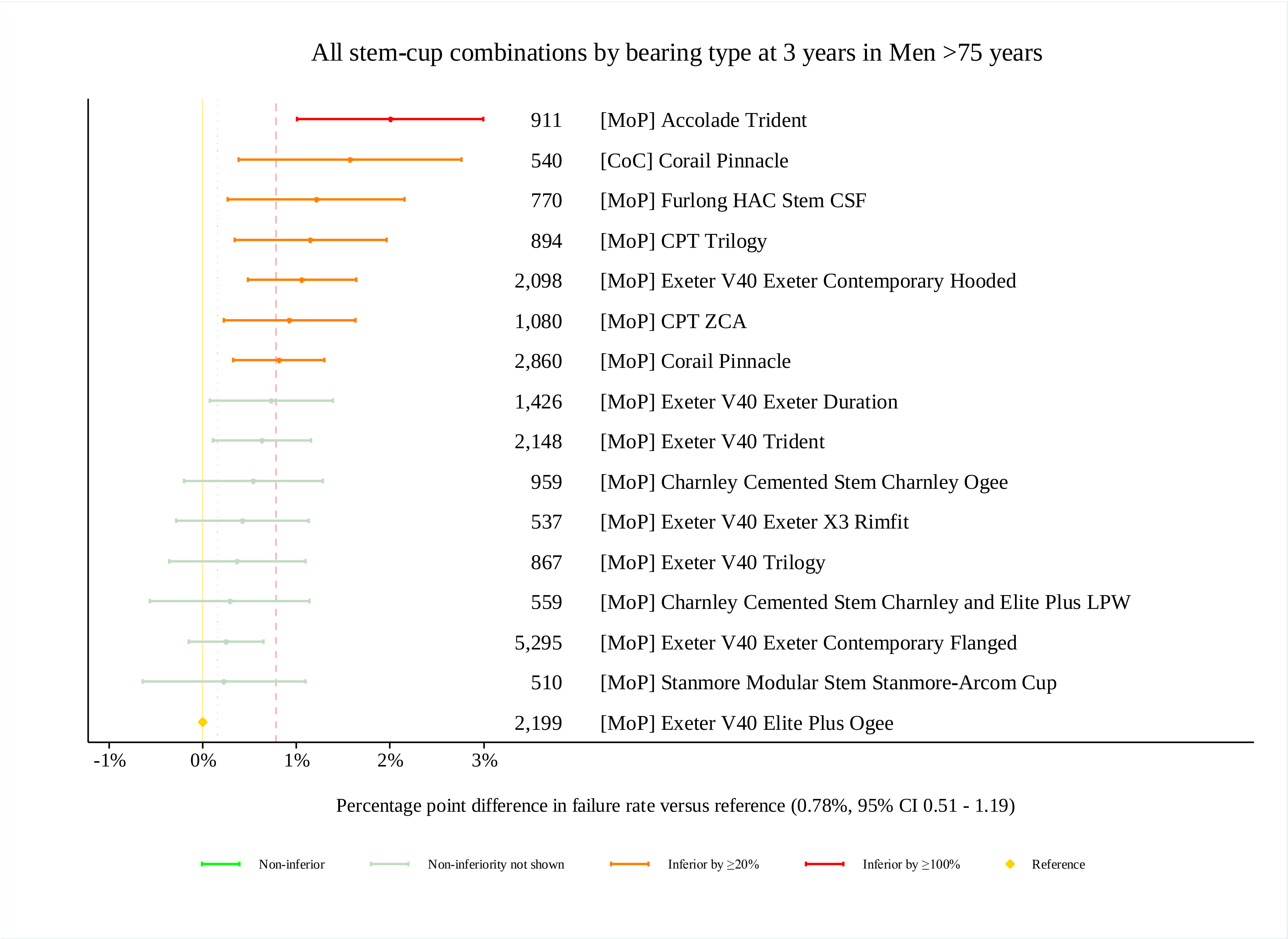

Supplementary Figure 3a: Difference in failure of implanted constructs compared to a contemporary reference at 5 years in men, using all stem-cup combinations with ≥500 procedures remaining at risk

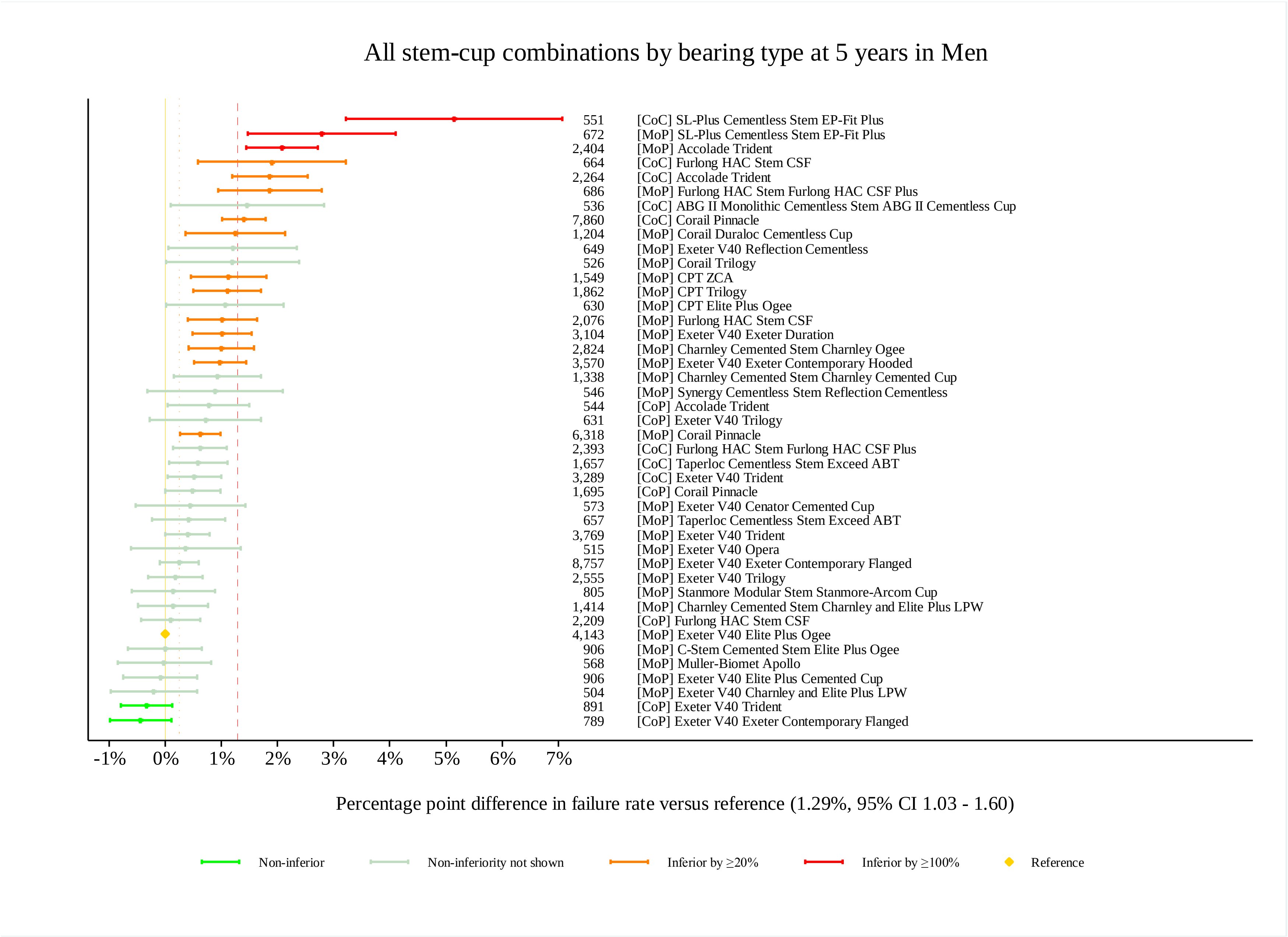

Supplementary Figure 3b: Difference in failure of implanted constructs compared to a contemporary reference at 5 years in men less than 55 years, using all stem-cup combinations with ≥500 procedures remaining at risk

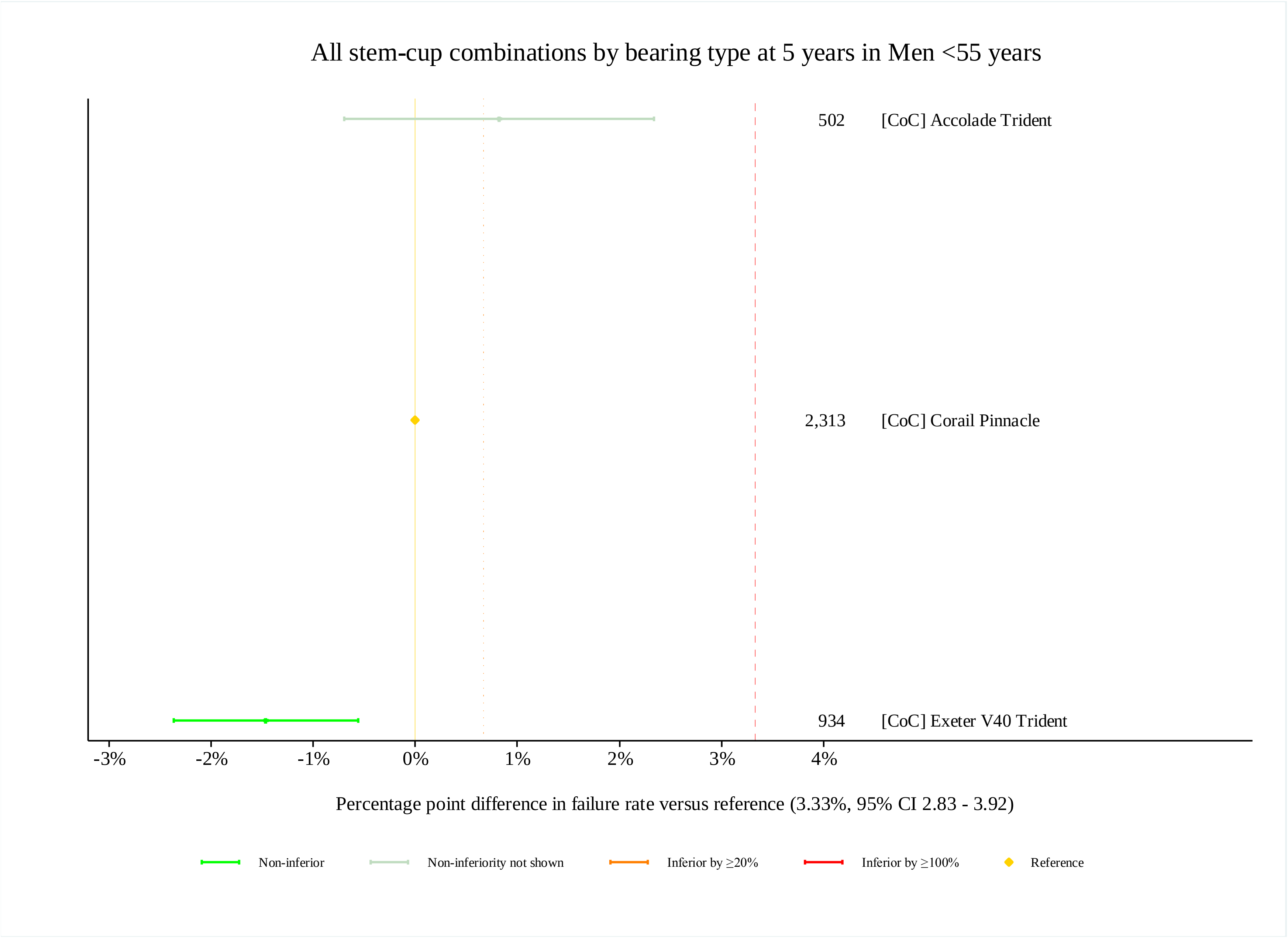

Supplementary Figure 3c: Difference in failure of implanted constructs compared to a contemporary reference at 5 years in men between 55 and 75 years, using all stem-cup combinations with ≥500 procedures remaining at risk

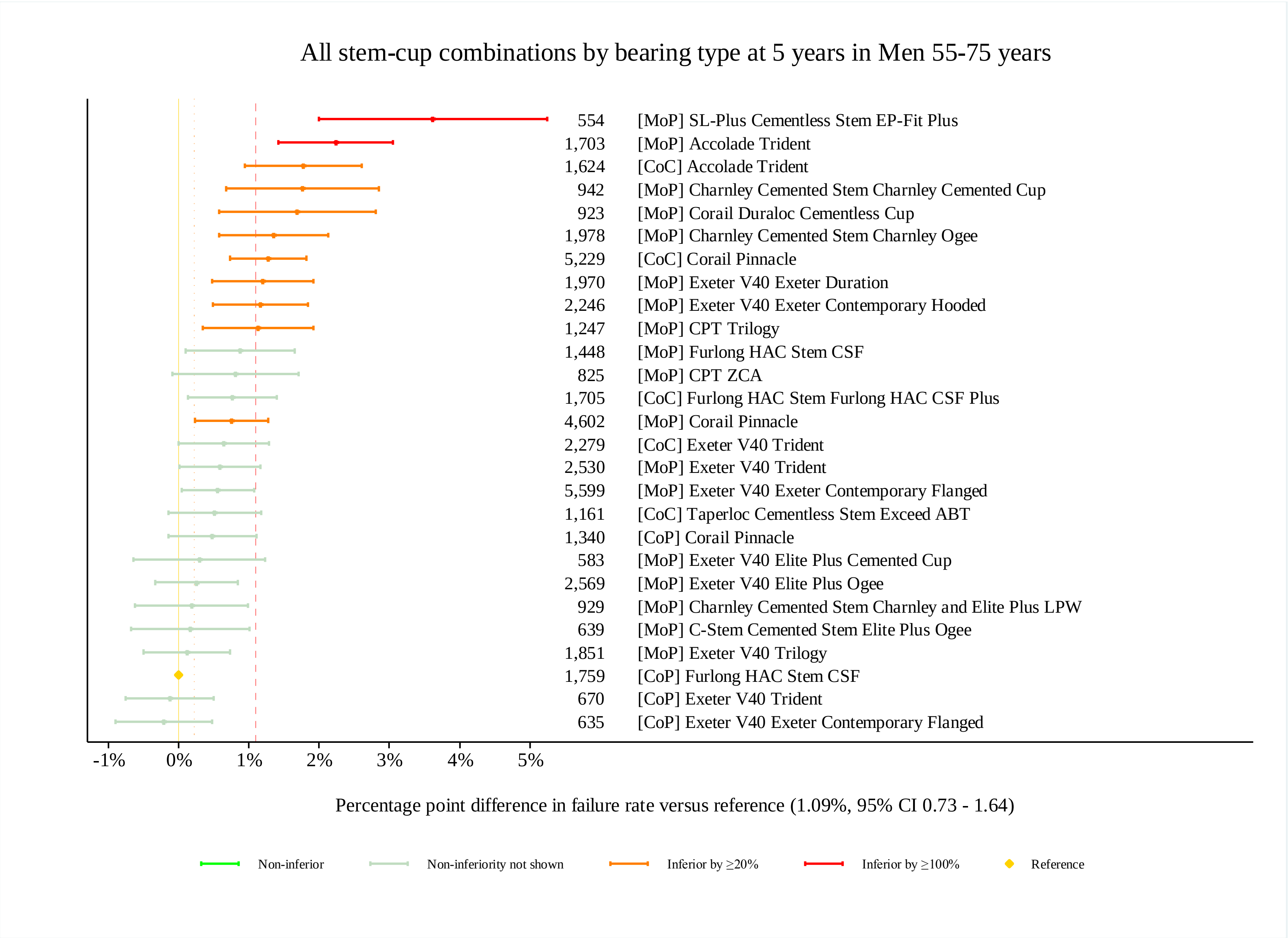

Supplementary Figure 3d: Difference in failure of implanted constructs compared to a contemporary reference at 5 years in men greater than 75 years, using all stem-cup combinations with ≥500 procedures remaining at risk

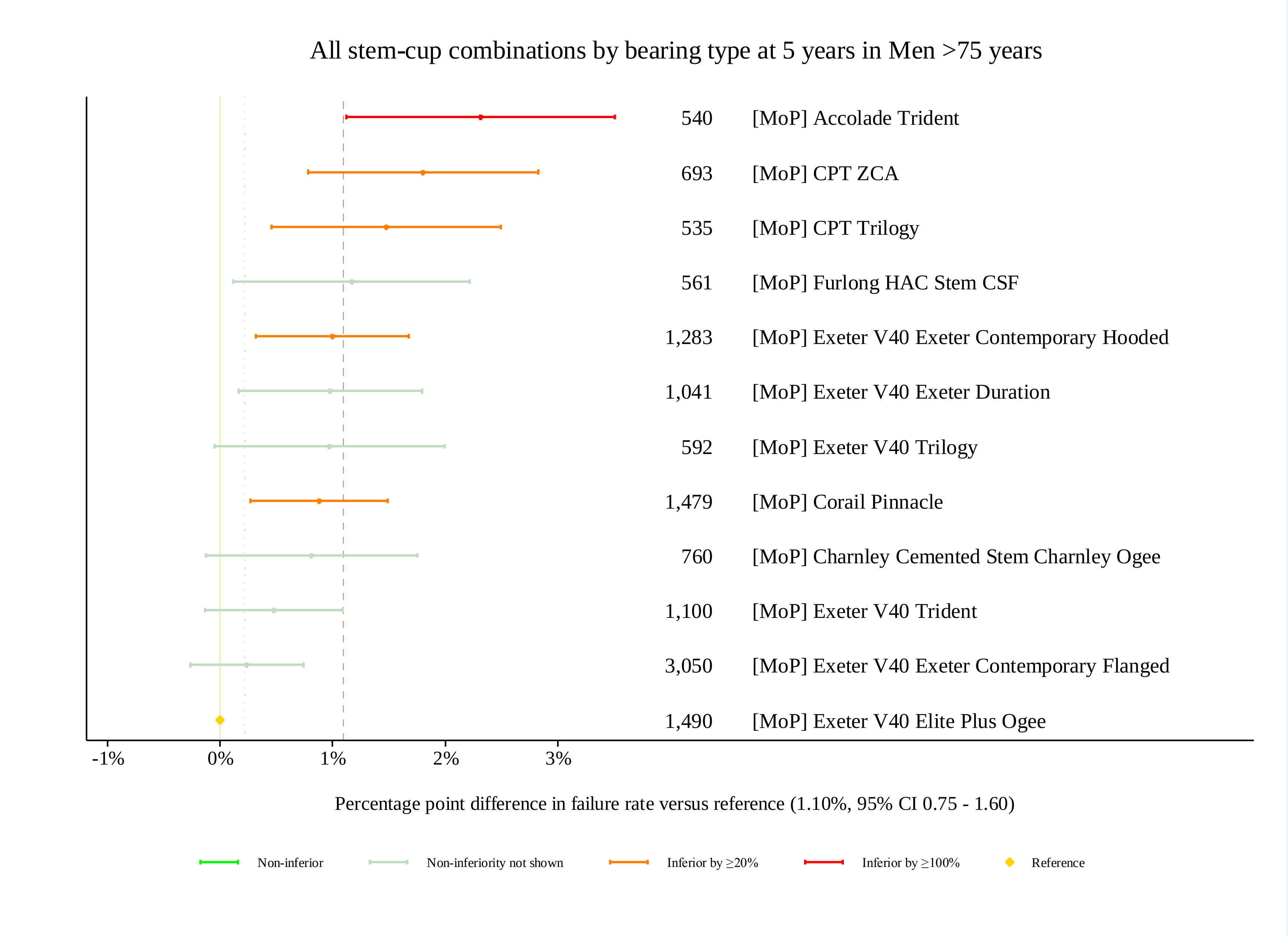

Supplementary Figure 4a: Difference in failure of implanted constructs compared to a contemporary reference at 7 years in men, using all stem-cup combinations with ≥500 procedures remaining at risk

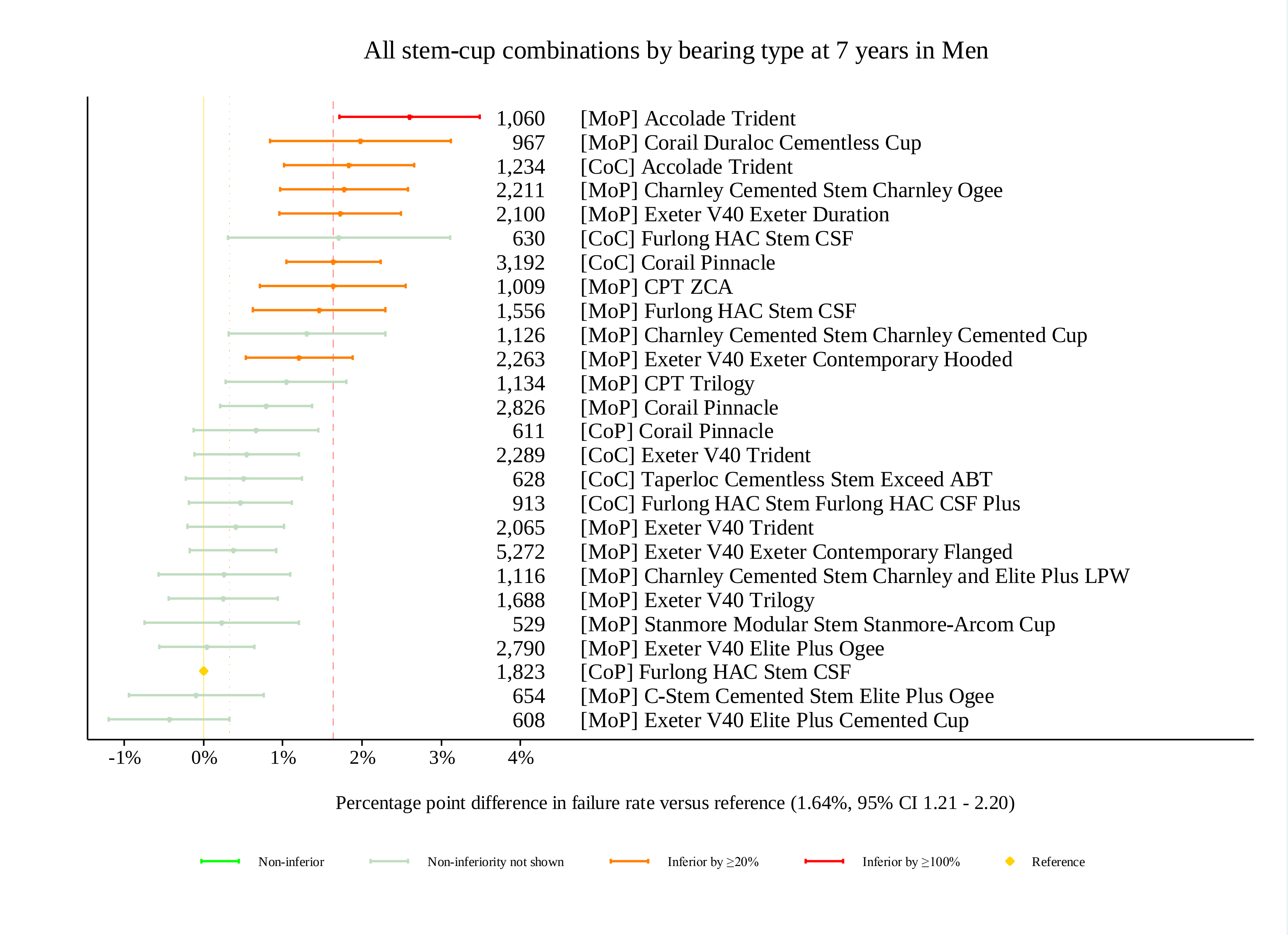

Supplementary Figure 4b: Difference in failure of implanted constructs compared to a contemporary reference at 7 years in men between 55 and 75 years, using all stem-cup combinations with ≥500 procedures remaining at risk

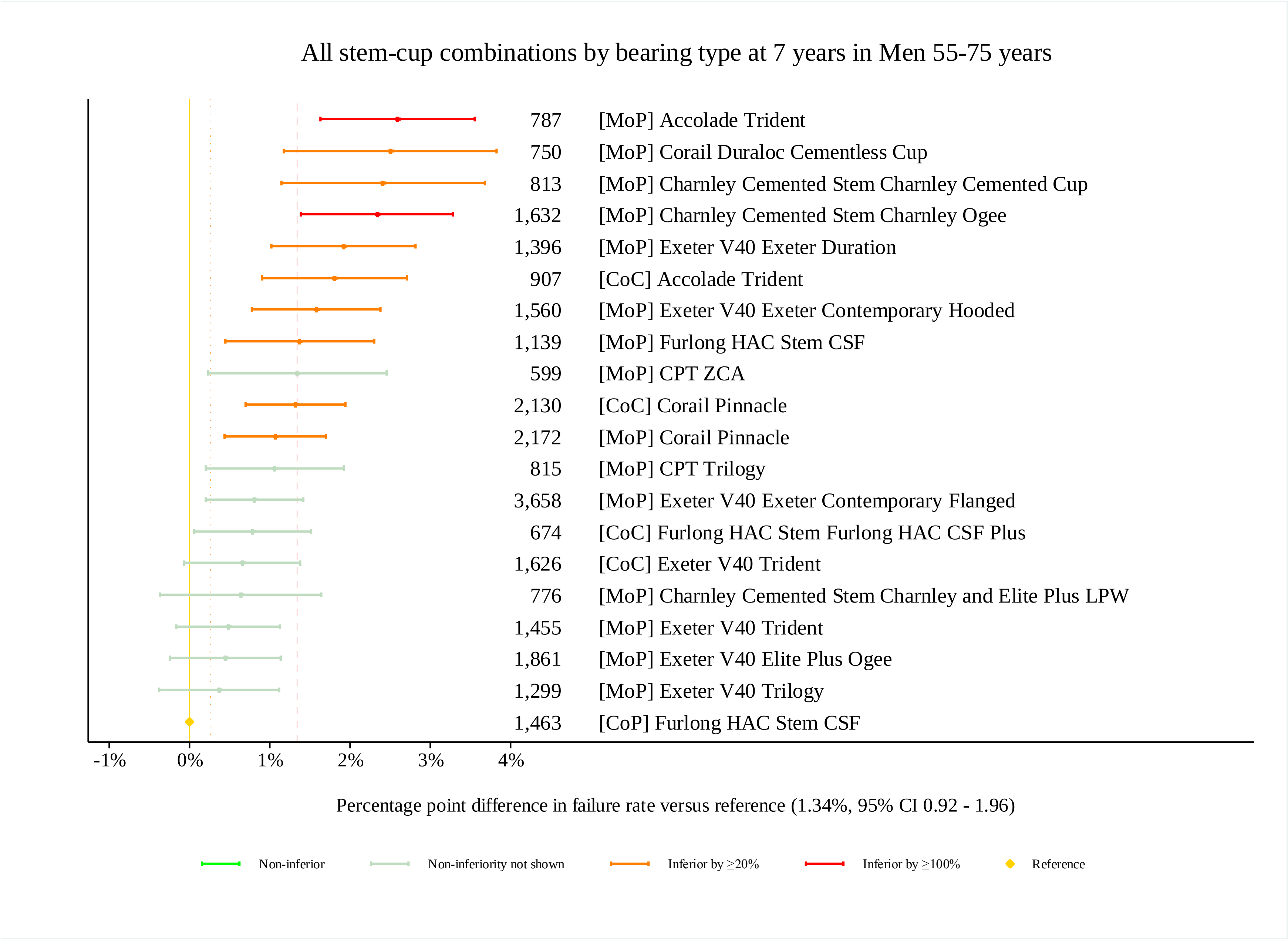

Supplementary Figure 4c: Difference in failure of implanted constructs compared to a contemporary reference at 7 years in men greater than 75 years, using all stem-cup combinations with ≥500 procedures remaining at risk

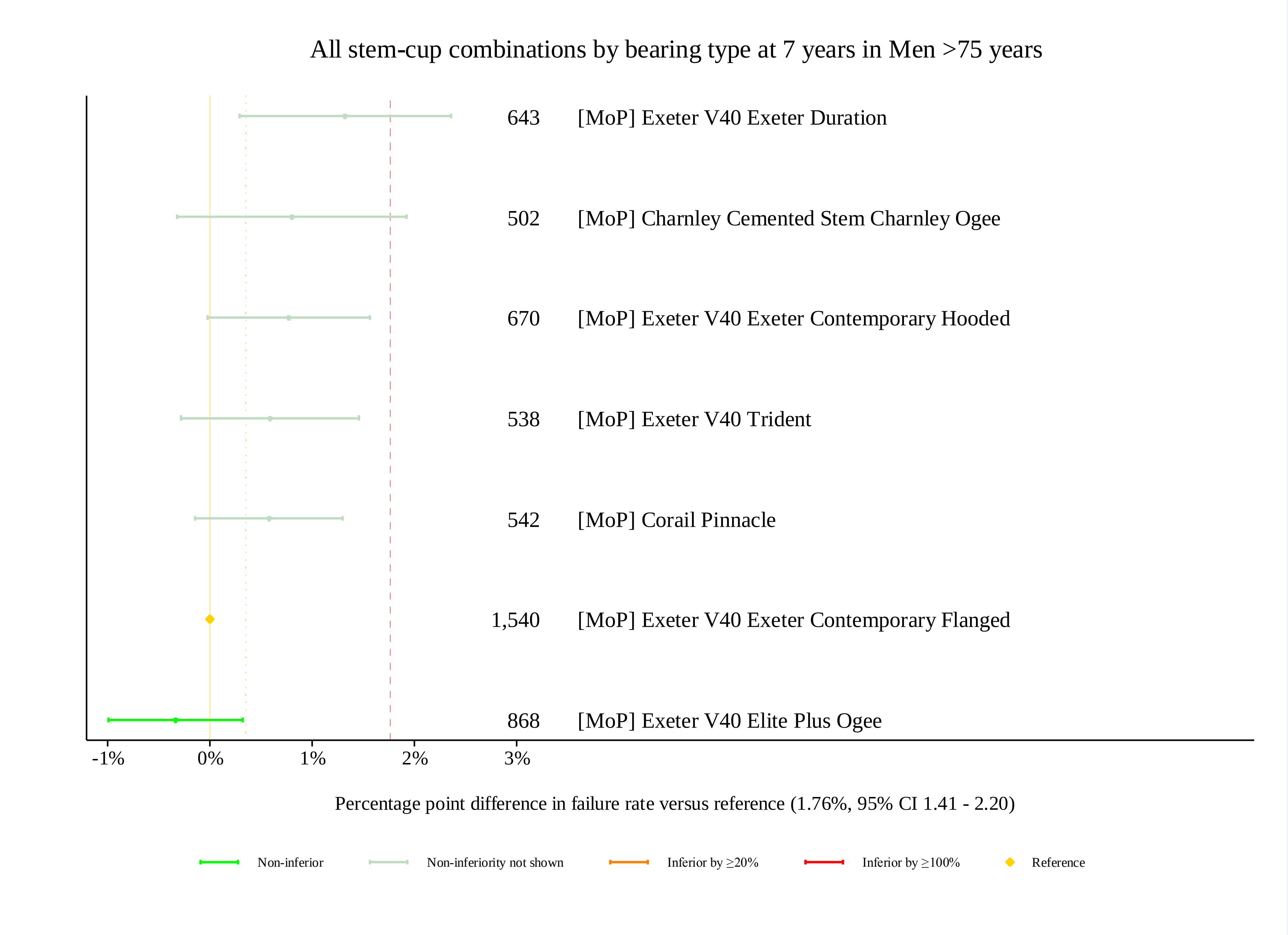

Supplementary Figure 5a: Difference in failure of implanted constructs compared to a contemporary reference at 10 years, using all stem-cup combinations with ≥500 procedures remaining at risk

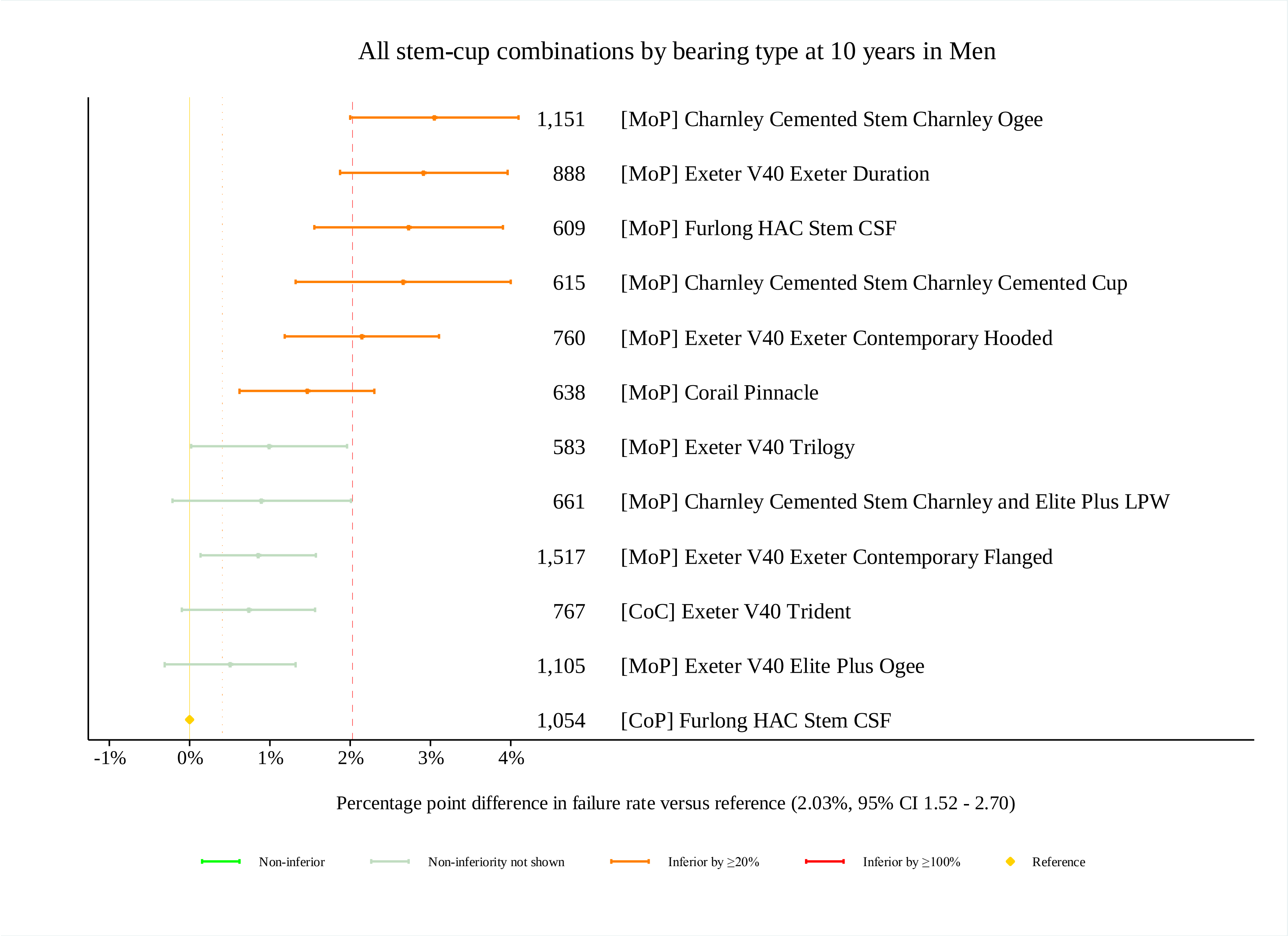

Supplementary Figure 5b: Difference in failure of implanted constructs compared to a contemporary reference at 10 years in men between 55 and 75 years, using all stem-cup combinations with ≥500 procedures remaining at risk

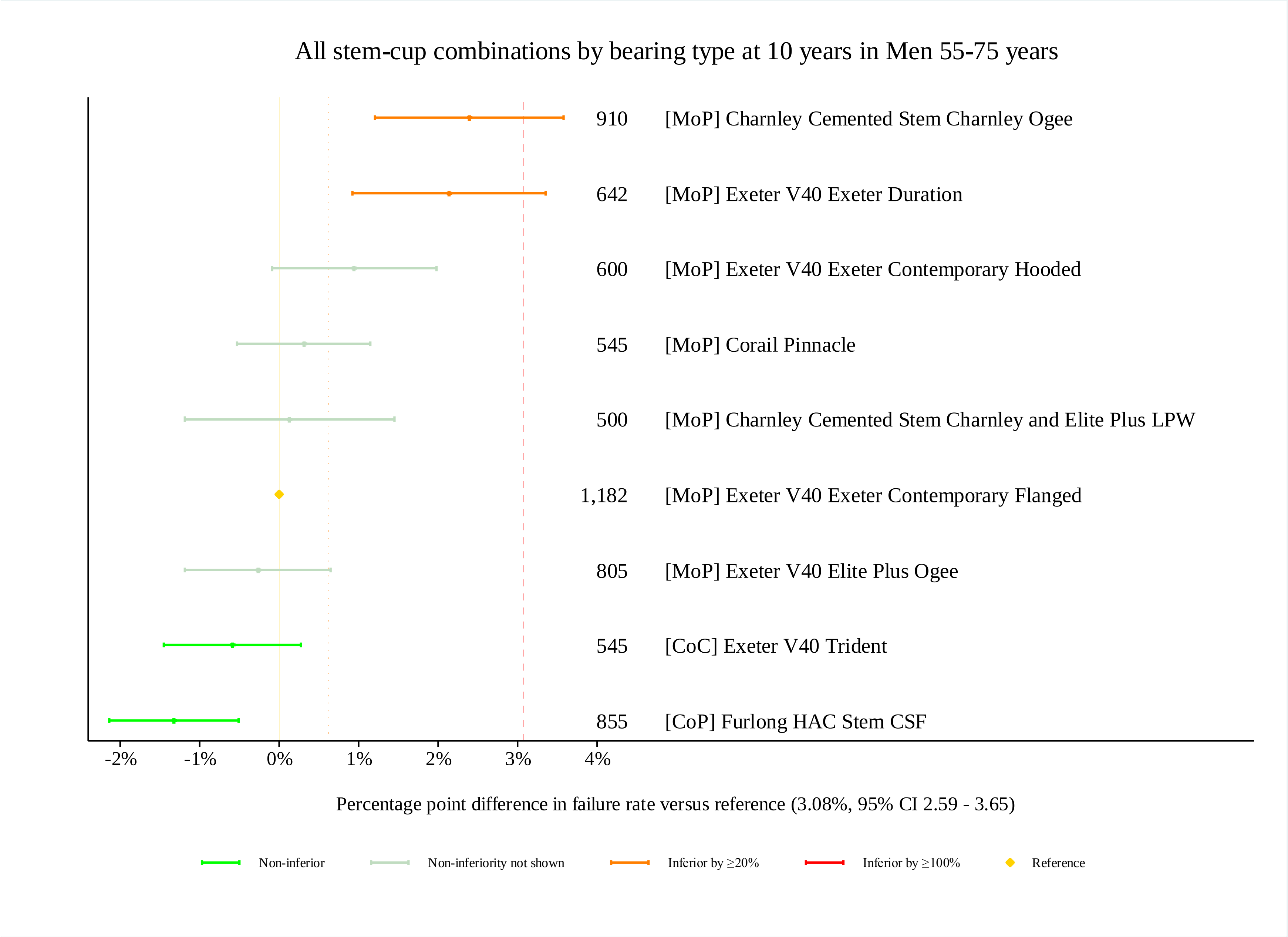

Supplementary Figure 6a: Difference in failure of implanted constructs compared to a contemporary reference at 3 years in women, using all stem-cup combinations with ≥500 procedures remaining at risk

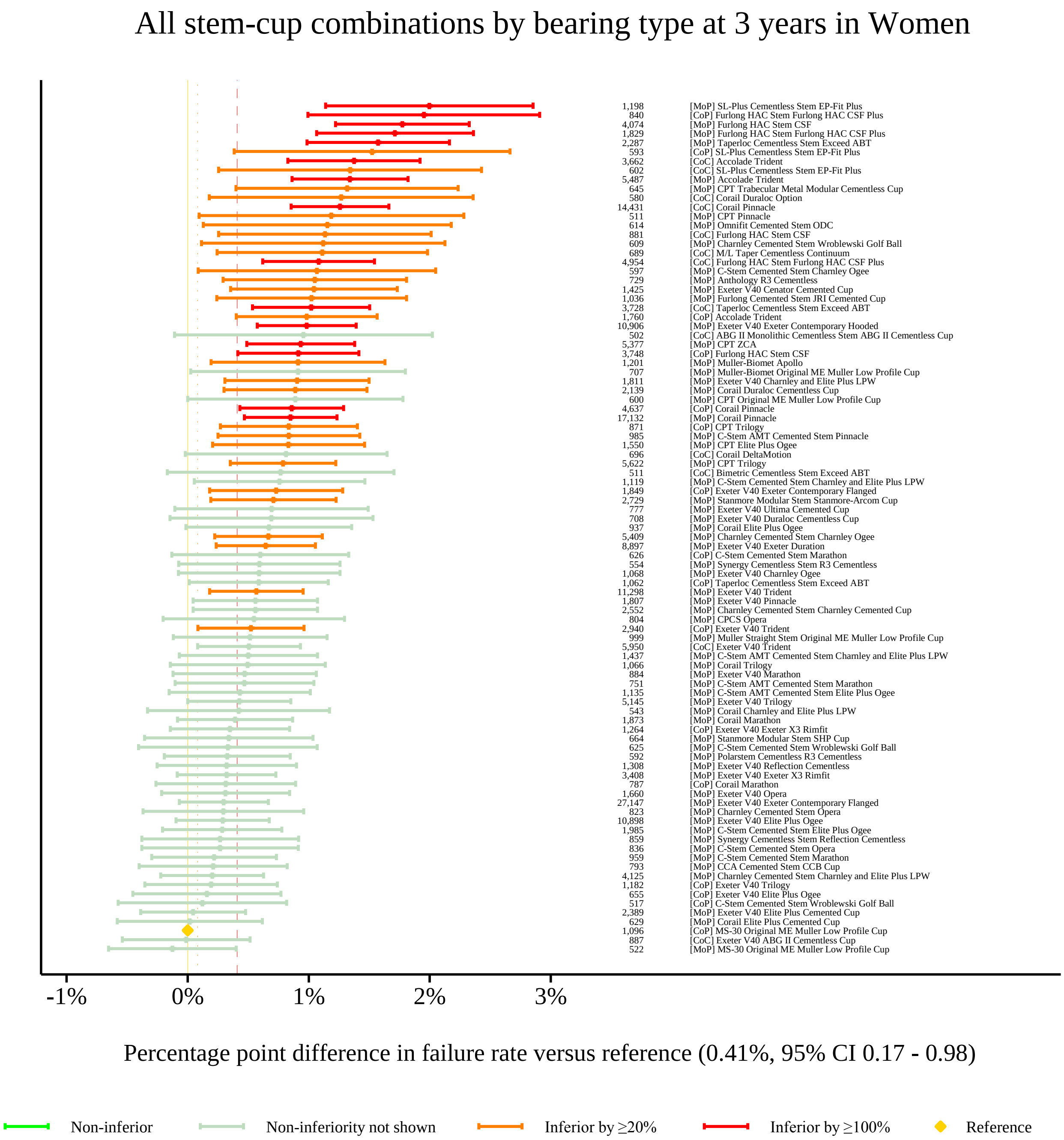

Supplementary Figure 6b: Difference in failure of implanted constructs compared to a contemporary reference at 3 years in women less than 55 years, using all stem-cup combinations with ≥500 procedures remaining at risk

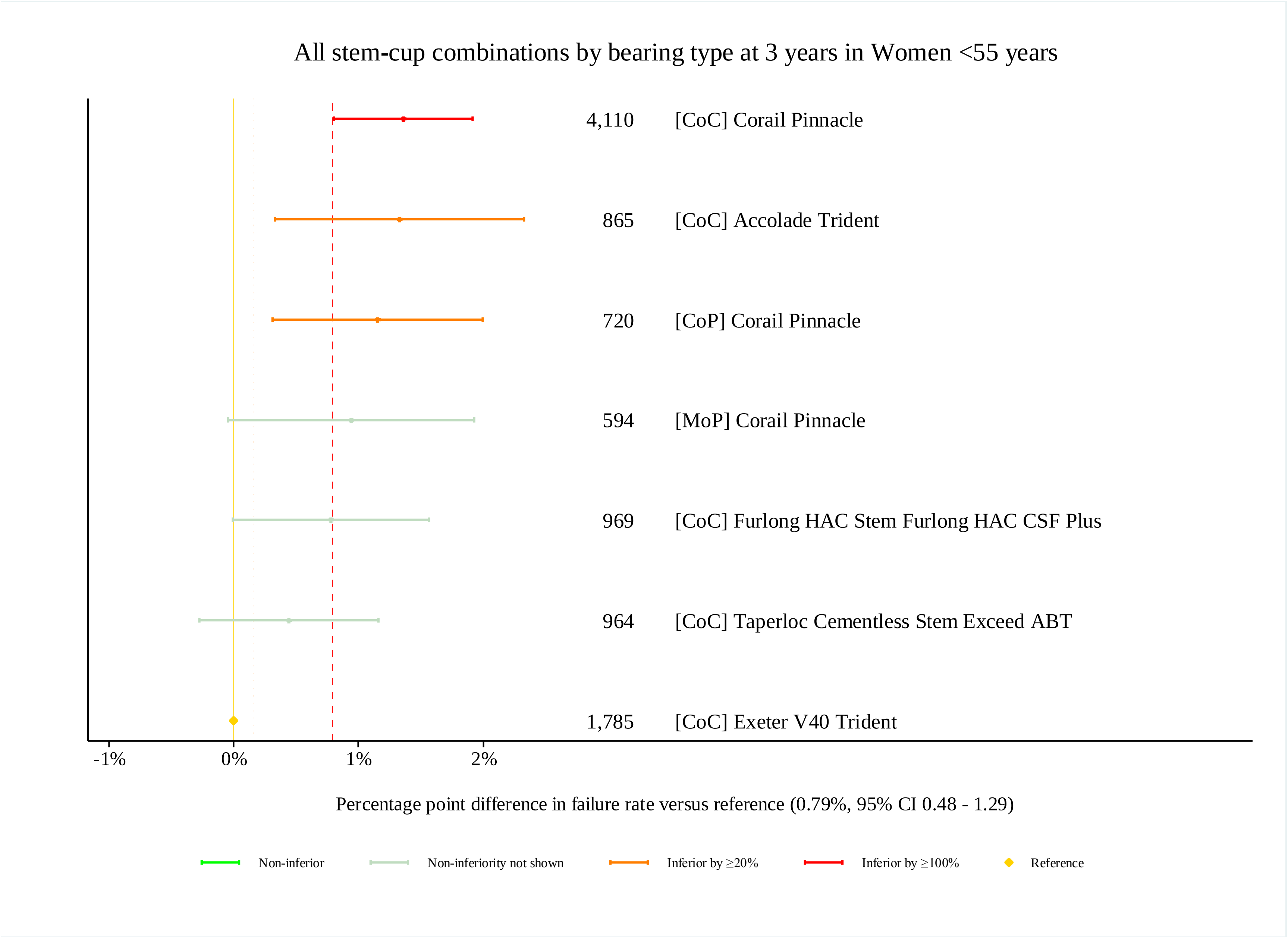

Supplementary Figure 6c: Difference in failure of implanted constructs compared to a contemporary reference at 3 years in women between 55 and 75 years, using all stem-cup combinations with ≥500 procedures remaining at risk

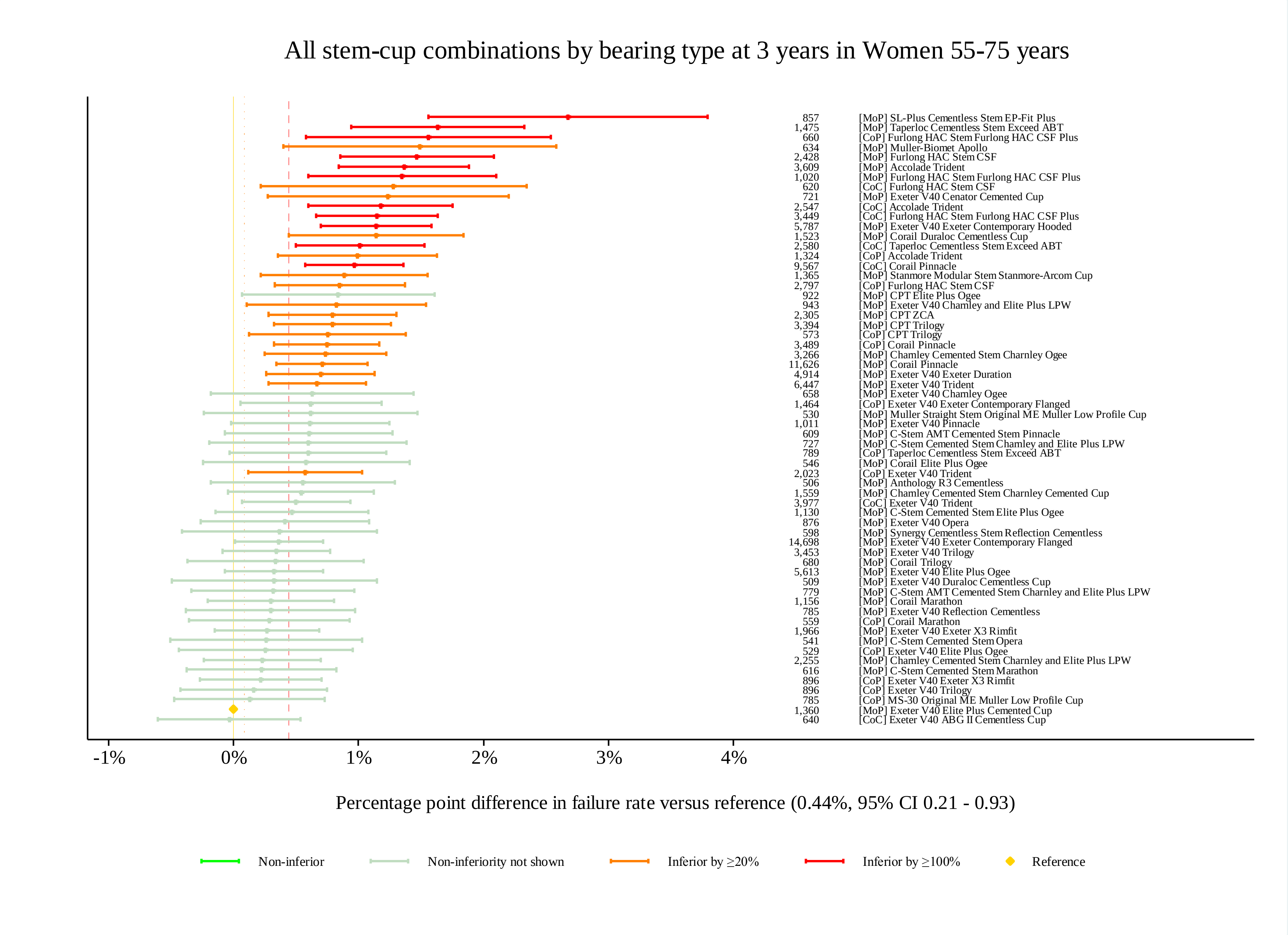

Supplementary Figure 6d: Difference in failure of implanted constructs compared to a contemporary reference at 3 years in women greater than 75 years, using all stem-cup combinations with ≥500 procedures remaining at risk

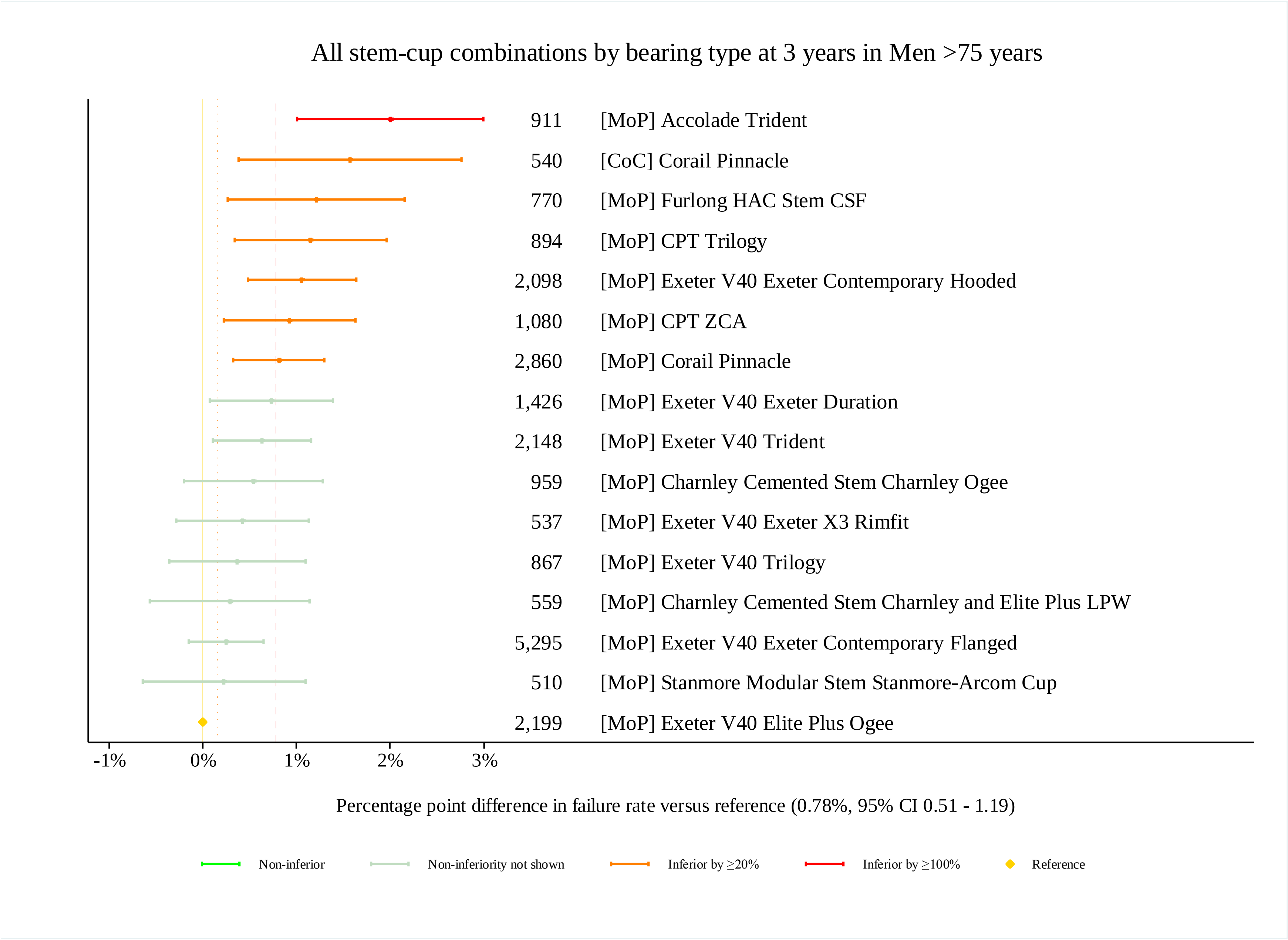

Supplementary Figure 7a: Difference in failure of implanted constructs compared to a contemporary reference at 5 years in women, using all stem-cup combinations with ≥500 procedures remaining at risk

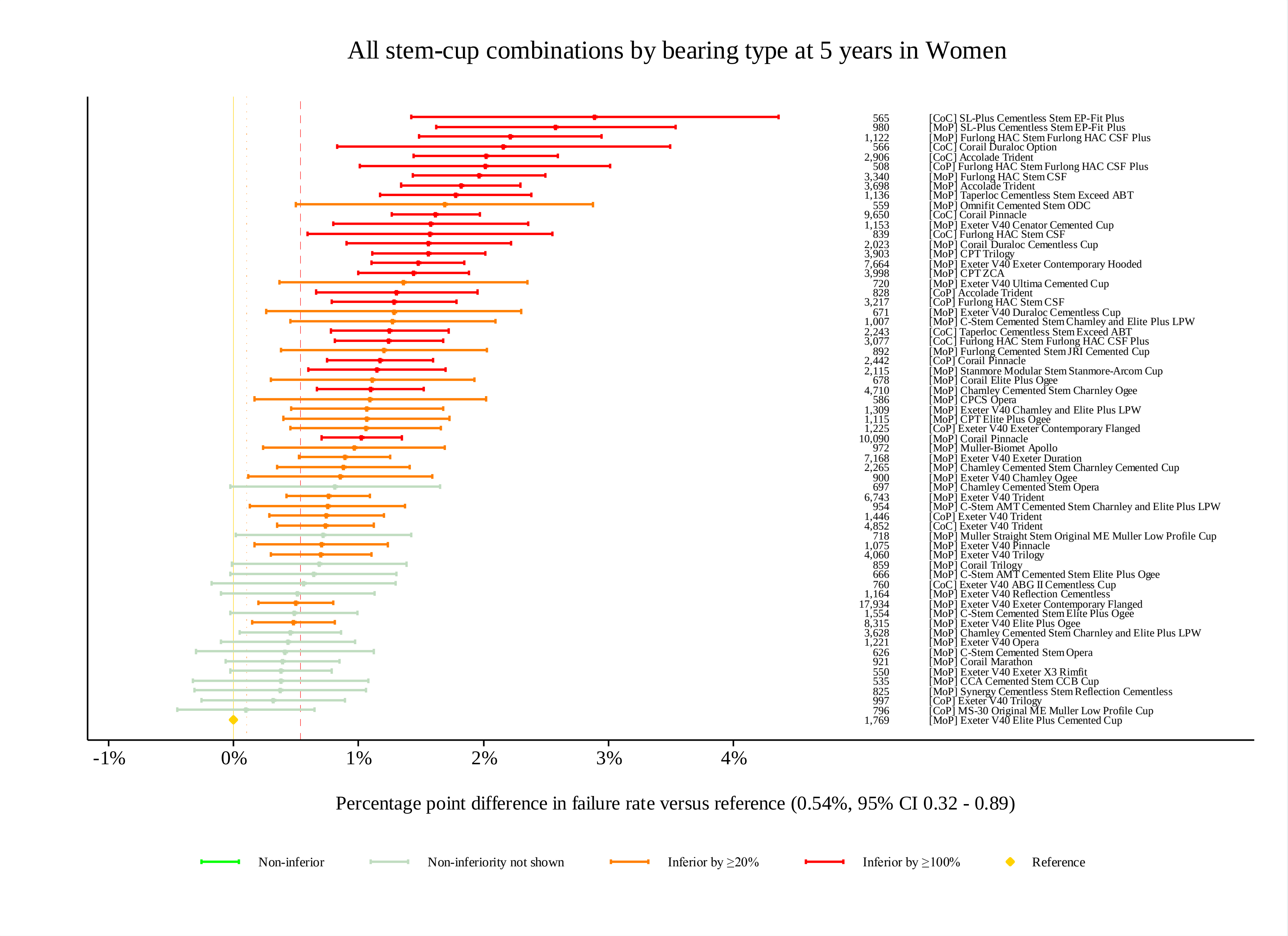

Supplementary Figure 7b: Difference in failure of implanted constructs compared to a contemporary reference at 5 years in women less than 55 years, using all stem-cup combinations with ≥500 procedures remaining at risk

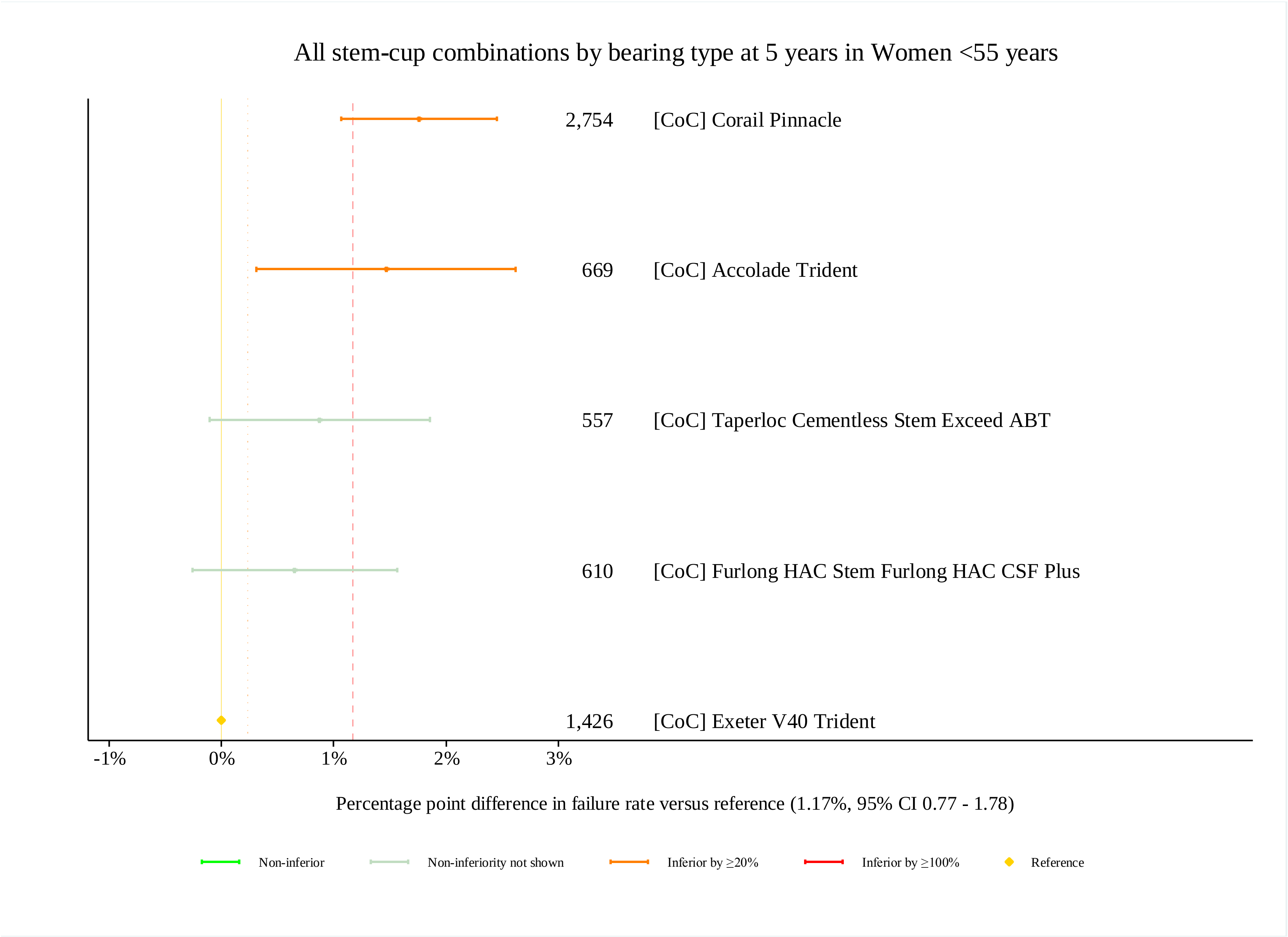

Supplementary Figure 7c: Difference in failure of implanted constructs compared to a contemporary reference at 5 years in women between 55 and 75 years, using all stem-cup combinations with ≥500 procedures remaining at risk

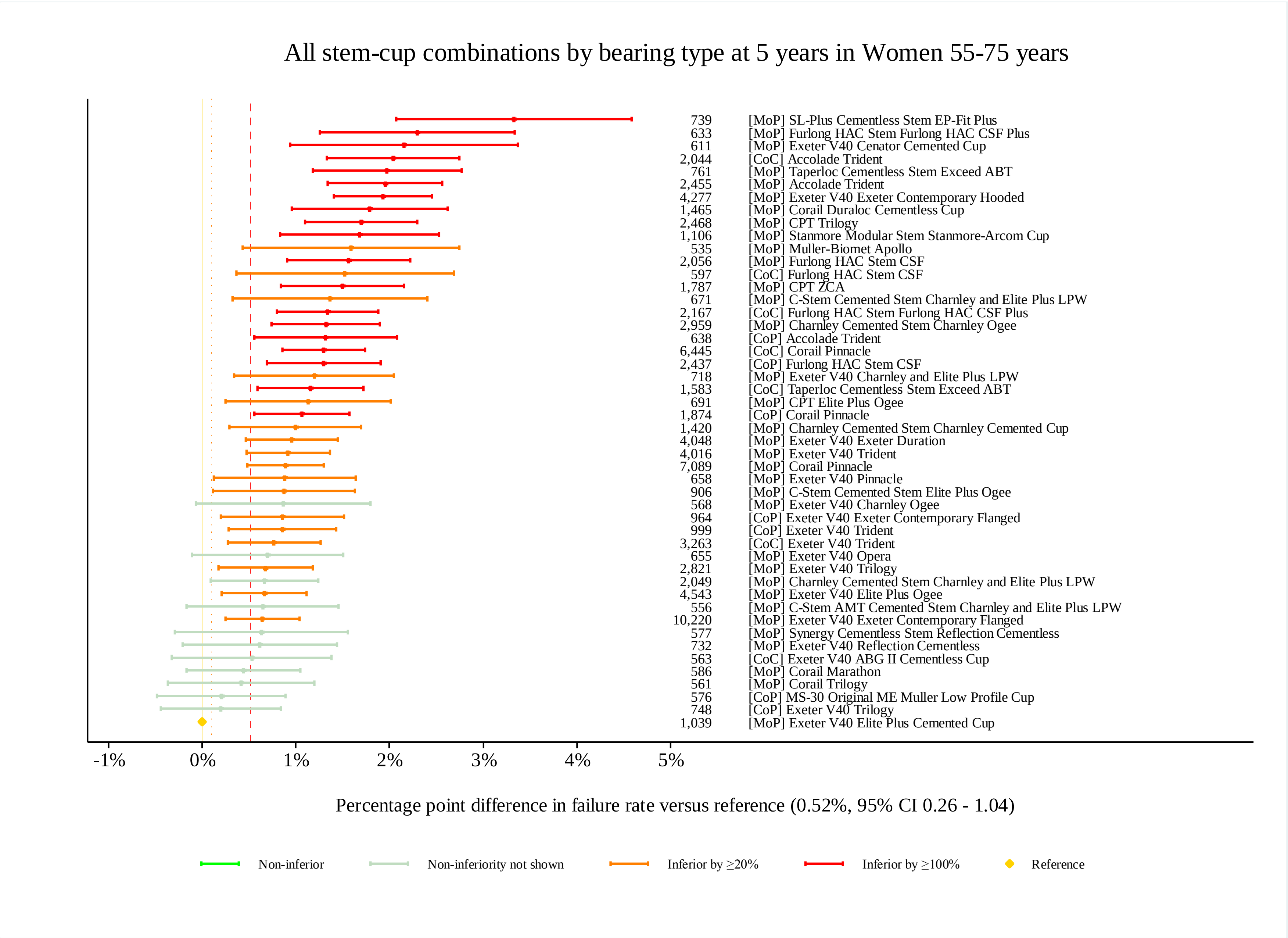

Supplementary Figure 7d: Difference in failure of implanted constructs compared to a contemporary reference at 5 years in women greater than 75 years, using all stem-cup combinations with ≥500 procedures remaining at risk

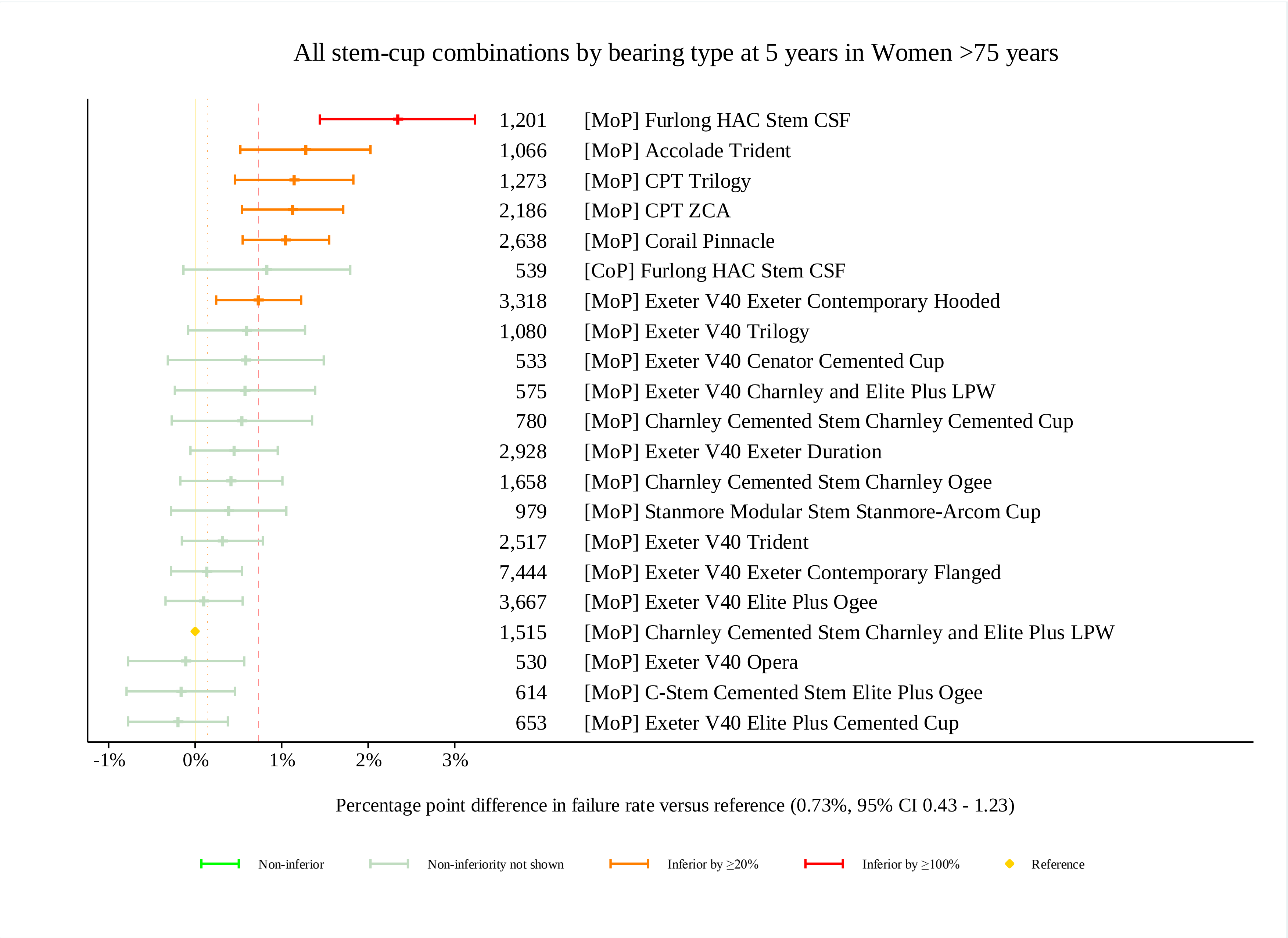

Supplementary Figure 8a: Difference in failure of implanted constructs compared to a contemporary reference at 7 years in women, using all stem-cup combinations with ≥500 procedures remaining at risk

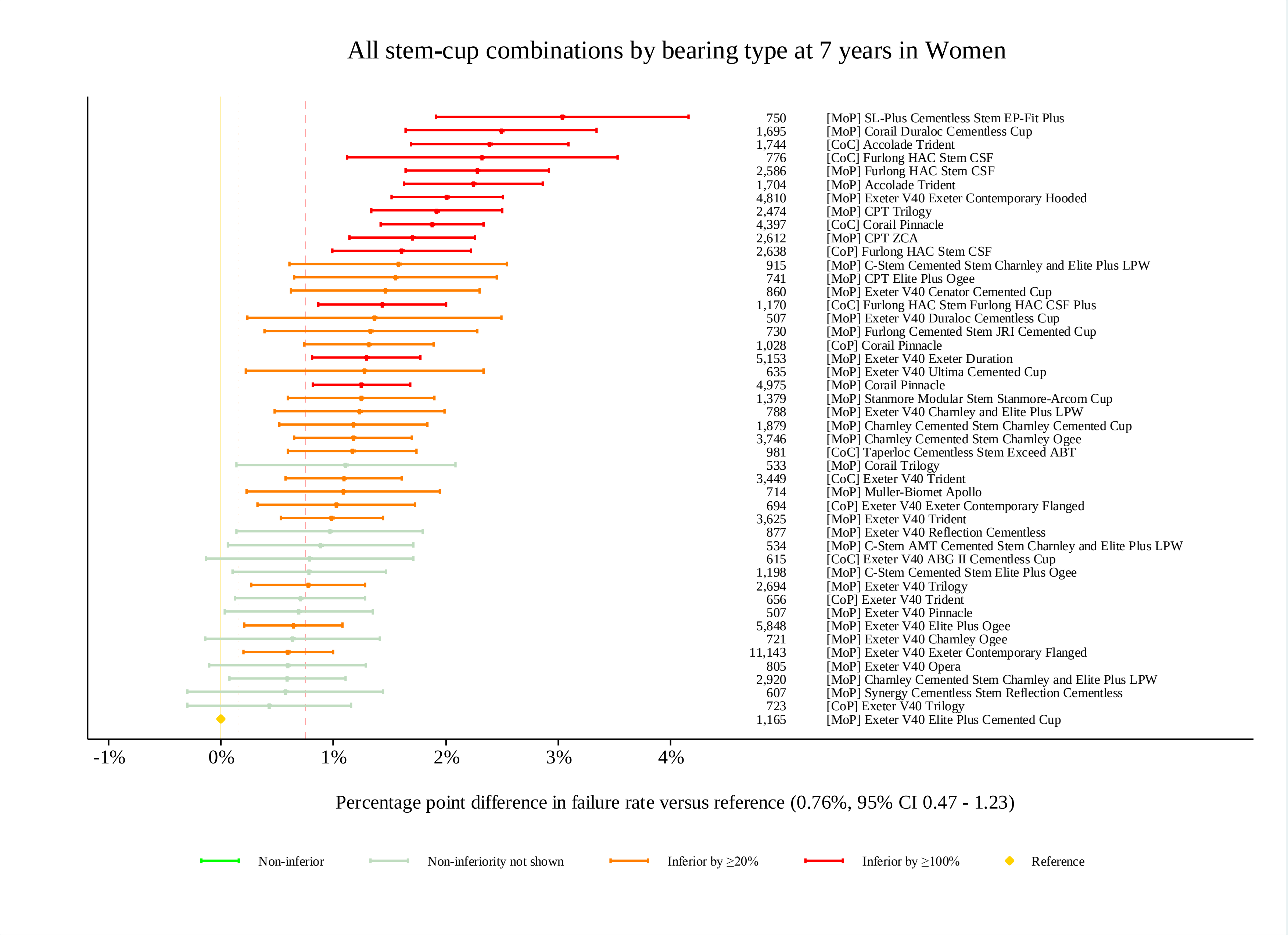

Supplementary Figure 8b: Difference in failure of implanted constructs compared to a contemporary reference at 7 years in women less than 55 years, using all stem-cup combinations with ≥500 procedures remaining at risk

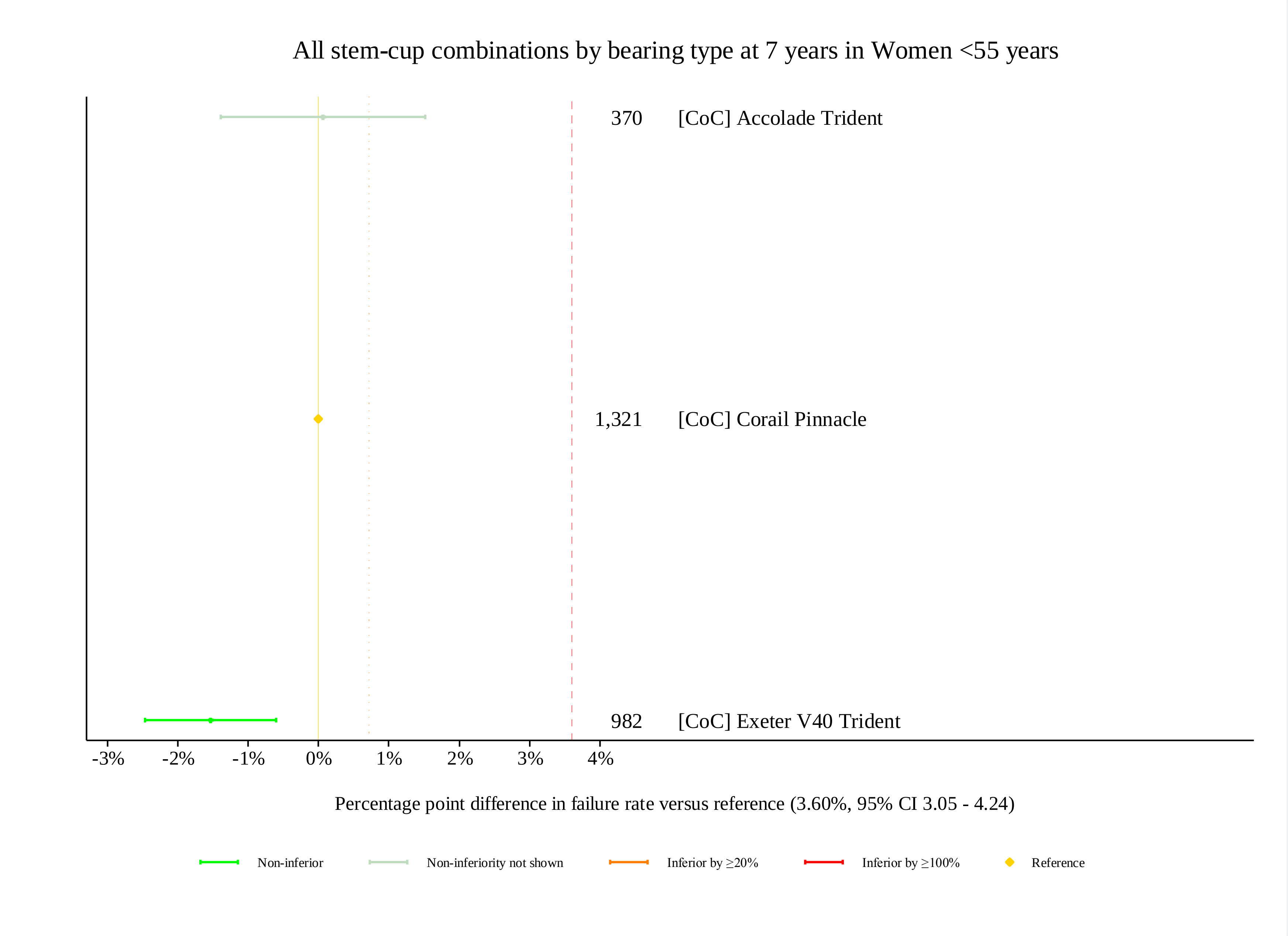

Supplementary Figure 8c: Difference in failure of implanted constructs compared to a contemporary reference at 7 years in women between 55 and 75 years, using all stem-cup combinations with ≥500 procedures remaining at risk

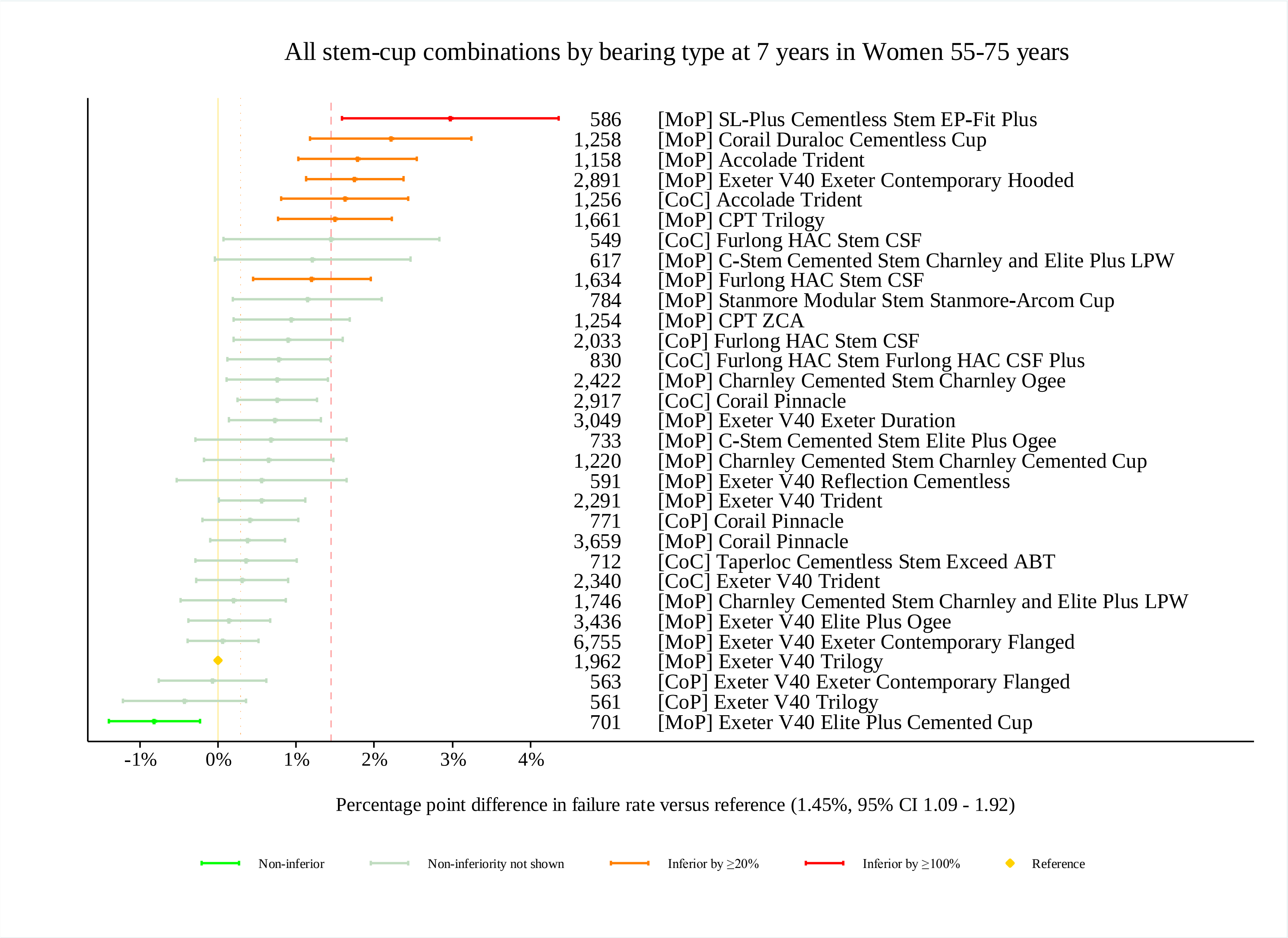

Supplementary Figure 8d: Difference in failure of implanted constructs compared to a contemporary reference at 7 years in women greater than 75 years, using all stem-cup combinations with ≥500 procedures remaining at risk

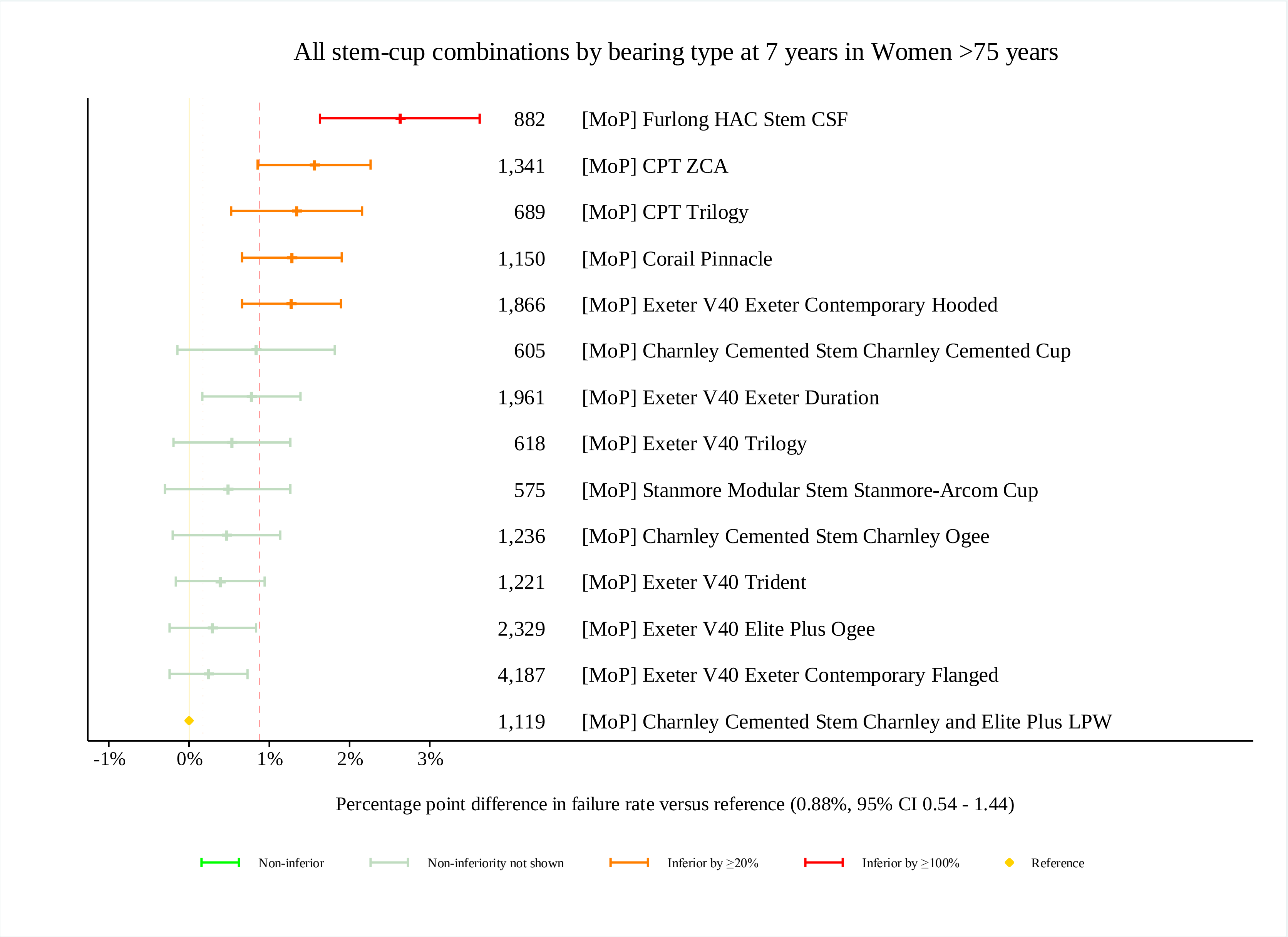

Supplementary Figure 9a: Difference in failure of implanted constructs compared to a contemporary reference at 10 years in women, using all stem-cup combinations with ≥500 procedures remaining at risk

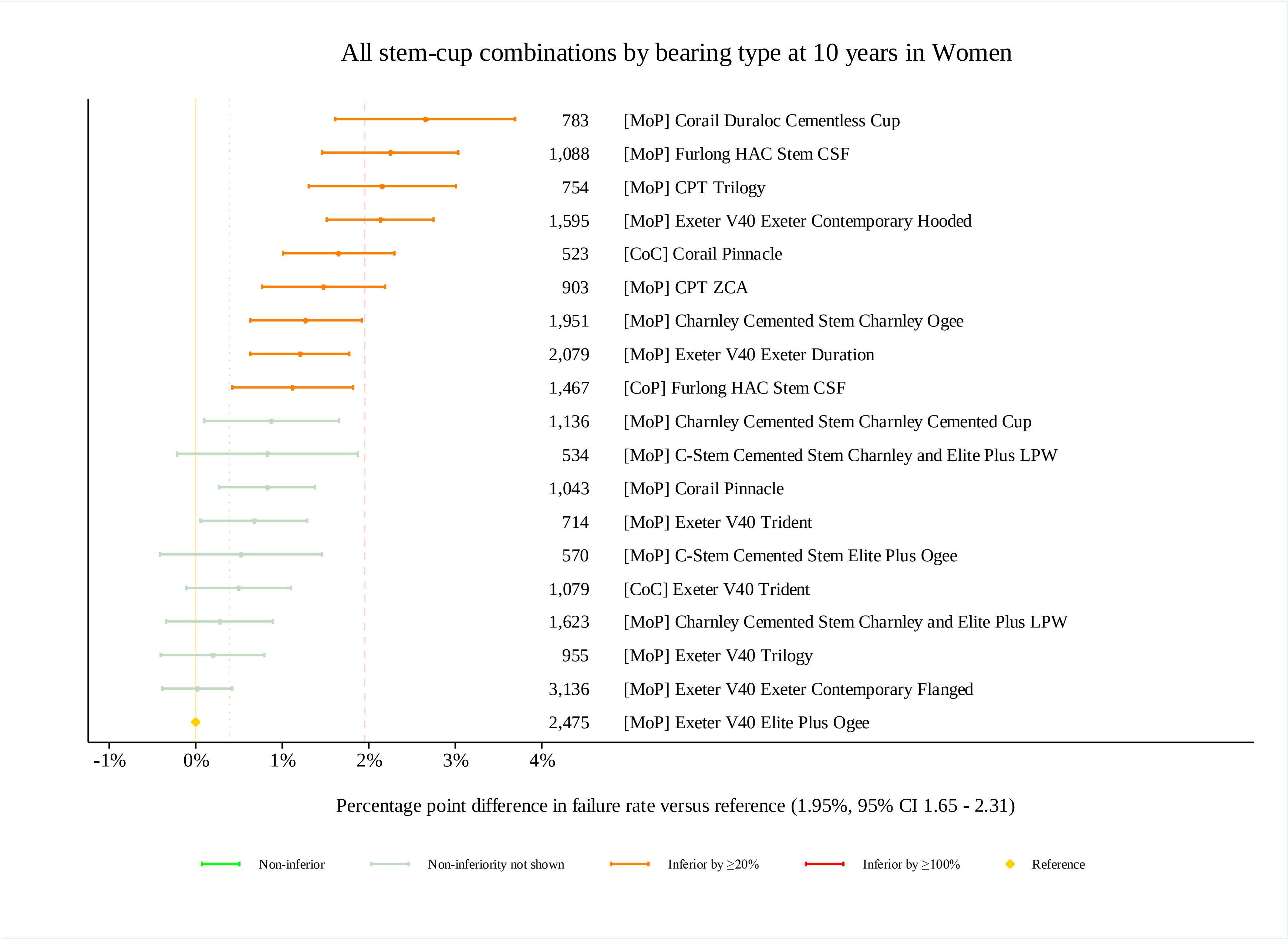

Supplementary Figure 9b: Difference in failure of implanted constructs compared to a contemporary reference at 10 years in women between 55 and 75 years, using all stem-cup combinations with ≥500 procedures remaining at risk

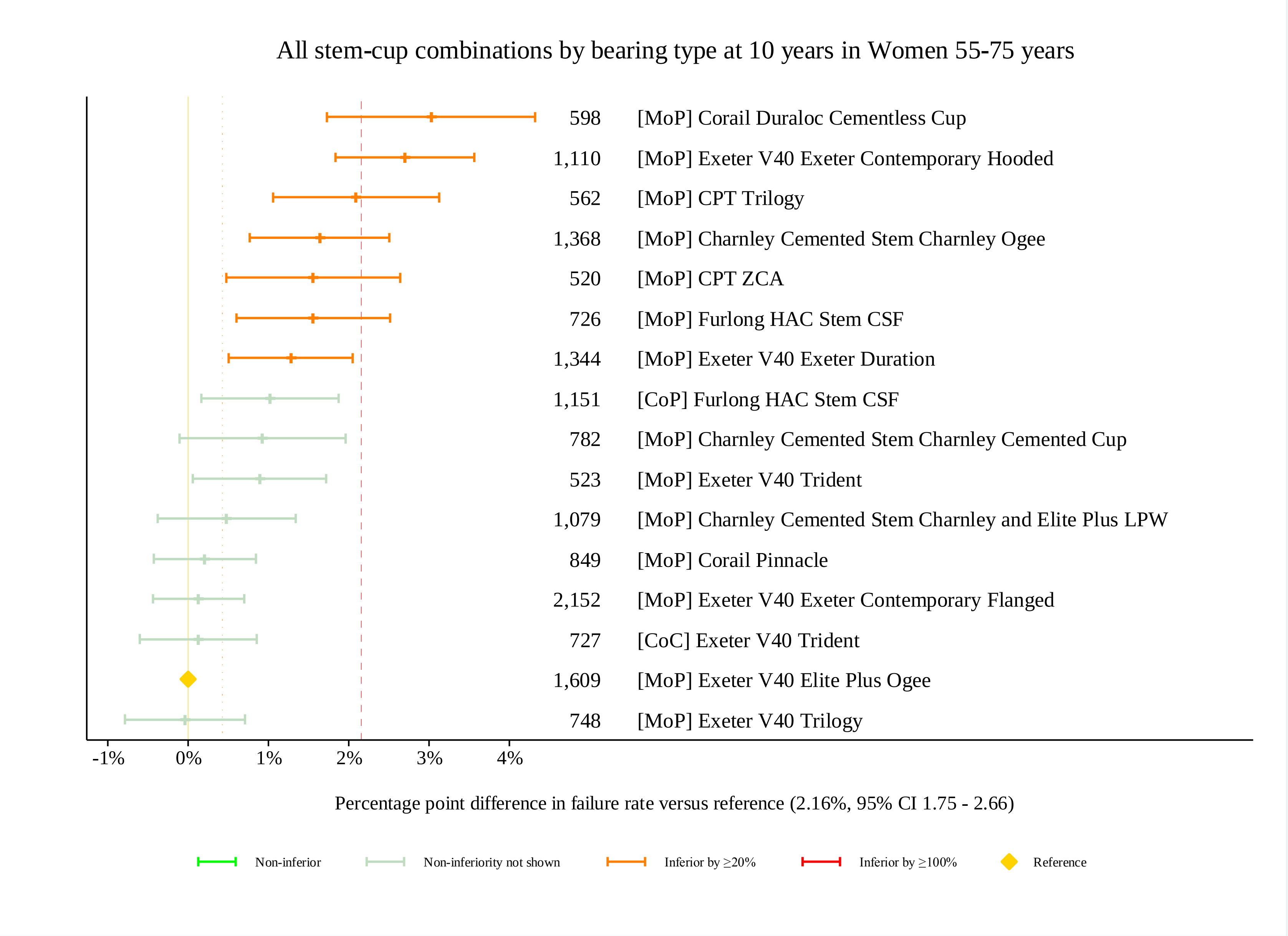

Supplementary table 1a: Difference in Kaplan-Meier failure estimate between a contemporary reference and implants with at least 250 at risk at 3 years since primary

Stem/cup brand	Number at risk	Cumulative failure (%)	Difference in failure (%)	95% CI	Equivalence status	p-value
[CoP] MS-30 Original ME Muller Low Profile Cup	1,554	0.39	[REFERENCE]			
[CoC] ABG II Monolithic Cementless Stem ABG II Cementless Cup	1,061	1.82	1.43	[0.59 , 2.27]	Inferior by ≥100%	0.001
[CoC] ABG II Monolithic Cementless Stem Trident	1,007	1.98	1.59	[0.70 , 2.48]	Inferior by ≥100%	<0.001
[CoC] AMIStem-H Versafit CC Trio	331	2.00	1.61	[0.44 , 2.77]	Inferior by ≥100%	0.007
[CoC] Accolade Trident	6,660	2.03	1.64	[1.20 , 2.08]	Inferior by ≥100%	<0.001
[CoC] Anthology R3 Cementless	327	1.13	0.74	[-0.24 , 1.72]	Non-inferiority not shown	0.140
[CoC] Bicontact Cementless Stem Plasmacup SC	346	1.01	0.62	[-0.41 , 1.65]	Non-inferiority not shown	0.237
[CoC] Bimetric Cementless Stem Exceed ABT	888	1.05	0.65	[-0.03 , 1.34]	Non-inferiority not shown	0.059
[CoC] C-Stem AMT Cemented Stem Pinnacle	770	0.83	0.44	[-0.18 , 1.06]	Non-inferiority not shown	0.168
[CoC] C-Stem Cemented Stem Pinnacle	342	0.54	0.15	[-0.65 , 0.95]	Non-inferiority not shown	0.715
[CoC] CPT Continuum	801	1.71	1.32	[0.52 , 2.13]	Inferior by ≥100%	0.001
[CoC] CPT Trilogy AB	525	1.12	0.73	[-0.21 , 1.67]	Non-inferiority not shown	0.127
[CoC] CPT Trilogy IT	314	1.14	0.74	[-0.13 , 1.62]	Non-inferiority not shown	0.095
[CoC] Charnley Modular Trilogy AB	252	0.39	0.00	[-0.82 , 0.81]	Non-inferiority not shown	0.996
[CoC] Corail Delta TT	726	2.10	1.71	[0.82 , 2.60]	Inferior by ≥100%	<0.001
[CoC] Corail DeltaMotion	1,147	1.40	1.01	[0.32 , 1.70]	Inferior by ≥20%	0.004
[CoC] Corail Duraloc Option	1,042	1.95	1.56	[0.68 , 2.43]	Inferior by ≥100%	<0.001
[CoC] Corail Pinnacle	27,047	1.79	1.40	[1.07 , 1.72]	Inferior by ≥100%	<0.001
[CoC] Corail Pinnacle Gription	452	2.41	2.02	[0.97 , 3.07]	Inferior by ≥100%	<0.001
[CoC] Corail Trinity	431	0.65	0.26	[-0.38 , 0.89]	Non-inferiority not shown	0.430
[CoC] Excia Cementless Plasmacup SC	888	1.20	0.81	[0.12 , 1.50]	Inferior by ≥20%	0.022
[CoC] Exeter V40 ABG II Cementless Cup	1,343	0.64	0.25	[-0.24 , 0.75]	Non-inferiority not shown	0.315
[CoC] Exeter V40 Trident	10,273	1.01	0.62	[0.27 , 0.96]	Inferior by ≥20%	<0.001
[CoC] Furlong Evolution Cementless Furlong HAC CSF Plus	597	1.37	0.98	[0.36 , 1.60]	Inferior by ≥20%	0.002
[CoC] Furlong HAC Stem CSF	1,568	2.08	1.69	[0.94 , 2.44]	Inferior by ≥100%	<0.001
[CoC] Furlong HAC Stem Furlong HAC CSF Plus	8,977	1.59	1.20	[0.83 , 1.57]	Inferior by ≥100%	<0.001
[CoC] Furlong HAC Stem Furlong Threaded	323	2.10	1.71	[0.14 , 3.28]	Inferior by ≥20%	0.033
[CoC] M/L Taper Cementless Continuum	1,393	1.63	1.24	[0.60 , 1.88]	Inferior by ≥100%	<0.001
[CoC] M/L Taper Cementless Trilogy IT	257	2.94	2.55	[0.93 , 4.18]	Inferior by ≥100%	0.002
[CoC] M/L Taper Kinectiv Cementless Continuum	258	3.39	3.00	[0.91 , 5.08]	Inferior by ≥100%	0.005
[CoC] Metafix Stem Trinity	813	1.29	0.90	[0.24 , 1.55]	Inferior by ≥20%	0.007
[CoC] Omnifit Cementless Stem Trident	486	1.99	1.60	[0.34 , 2.85]	Inferior by ≥20%	0.013
[CoC] Polarstem Cementless R3 Cementless	800	0.78	0.39	[-0.20 , 0.97]	Non-inferiority not shown	0.195
[CoC] S-Rom Cementless Stem Pinnacle	543	3.12	2.73	[1.38 , 4.08]	Inferior by ≥100%	<0.001
[CoC] SL-Plus Cementless Stem EP-Fit Plus	1,202	3.46	3.07	[2.03 , 4.12]	Inferior by ≥100%	<0.001
[CoC] SL-Plus Cementless Stem R3 Cementless	539	0.80	0.41	[-0.35 , 1.16]	Non-inferiority not shown	0.292
[CoC] SPS Modular April - Ceramic	593	1.50	1.11	[0.14 , 2.07]	Inferior by ≥20%	0.025
[CoC] Summit Cementless Stem Pinnacle	482	1.33	0.94	[0.02 , 1.87]	Non-inferiority not shown	0.046
[CoC] Synergy Cementless Stem R3 Cementless	280	0.96	0.57	[-0.41 , 1.54]	Non-inferiority not shown	0.257

For peer review only

[CoC] Taperloc Cementless Stem Exceed ABT	6,704	1.52	1.13	[0.75 , 1.51]	Inferior by ≥100%	<0.001
[CoC] Trilock BPS Pinnacle	341	2.06	1.67	[0.36 , 2.98]	Inferior by ≥20%	0.012
[CoC] miniHip Trinity	713	1.91	1.52	[0.64 , 2.41]	Inferior by ≥100%	0.001
[CoP] Accolade Trident	3,079	1.52	1.12	[0.67 , 1.58]	Inferior by ≥100%	<0.001
[CoP] C-Stem AMT Cemented Stem Marathon	321	0.80	0.41	[-0.34 , 1.15]	Non-inferiority not shown	0.285
[CoP] C-Stem AMT Cemented Stem Pinnacle	346	1.63	1.24	[0.36 , 2.12]	Inferior by ≥20%	0.006
[CoP] C-Stem Cemented Stem Elite Plus Ogee	607	0.57	0.18	[-0.45 , 0.80]	Non-inferiority not shown	0.583
[CoP] C-Stem Cemented Stem Marathon	1,150	1.03	0.64	[0.10 , 1.19]	Inferior by ≥20%	0.021
[CoP] C-Stem Cemented Stem Opera	785	0.63	0.23	[-0.38 , 0.85]	Non-inferiority not shown	0.457
[CoP] C-Stem Cemented Stem Wroblewski Golf Ball	913	0.69	0.30	[-0.29 , 0.88]	Non-inferiority not shown	0.320
[CoP] CLS Cementless Stem Trilogy	283	0.59	0.20	[-0.67 , 1.06]	Non-inferiority not shown	0.656
[CoP] CPT Trilogy	1,321	1.47	1.08	[0.59 , 1.58]	Inferior by ≥100%	<0.001
[CoP] CPT ZCA	349	0.39	-0.01	[-0.62 , 0.61]	Non-inferiority not shown	0.987
[CoP] Corail Charnley and Elite Plus LPW	627	1.72	1.33	[0.32 , 2.34]	Inferior by ≥20%	0.010
[CoP] Corail Duraloc Cementless Cup	301	4.40	4.01	[1.74 , 6.28]	Inferior by ≥100%	0.001
[CoP] Corail Elite Plus Cemented Cup	409	0.95	0.56	[-0.32 , 1.43]	Non-inferiority not shown	0.214
[CoP] Corail Elite Plus Ogee	464	1.70	1.31	[0.17 , 2.46]	Inferior by ≥20%	0.024
[CoP] Corail Marathon	1,309	1.12	0.73	[0.20 , 1.26]	Inferior by ≥20%	0.007
[CoP] Corail Pinnacle	8,130	1.21	0.82	[0.48 , 1.16]	Inferior by ≥100%	<0.001
[CoP] Corail Trabecular Metal Modular Cementless Cup	335	2.76	2.36	[0.85 , 3.88]	Inferior by ≥100%	0.002
[CoP] Corail Trident	300	0.26	-0.13	[-0.72 , 0.45]	Non-inferiority not shown	0.660
[CoP] Corail Trilogy	654	1.31	0.92	[0.02 , 1.81]	Non-inferiority not shown	0.045
[CoP] Excia Cementless Plasmacup SC	301	0.96	0.57	[-0.32 , 1.47]	Non-inferiority not shown	0.210
[CoP] Exeter V40 Charnley and Elite Plus LPW	674	1.59	1.20	[0.32 , 2.07]	Inferior by ≥20%	0.008
[CoP] Exeter V40 Elite Plus Ogee	1,190	0.70	0.31	[-0.20 , 0.81]	Non-inferiority not shown	0.231
[CoP] Exeter V40 Exeter Contemporary Flanged	2,995	0.99	0.60	[0.18 , 1.03]	Inferior by ≥20%	0.005
[CoP] Exeter V40 Exeter Contemporary Hooded	779	2.03	1.64	[0.70 , 2.57]	Inferior by ≥100%	0.001
[CoP] Exeter V40 Exeter Duration	775	0.66	0.27	[-0.33 , 0.88]	Non-inferiority not shown	0.378
[CoP] Exeter V40 Exeter X3 Rimfit	2,175	0.99	0.60	[0.18 , 1.02]	Inferior by ≥20%	0.005
[CoP] Exeter V40 Marathon	485	0.70	0.31	[-0.34 , 0.96]	Non-inferiority not shown	0.349
[CoP] Exeter V40 Pinnacle	542	1.14	0.75	[0.07 , 1.42]	Non-inferiority not shown	0.030
[CoP] Exeter V40 Trident	4,857	0.88	0.48	[0.14 , 0.83]	Inferior by ≥20%	0.006
[CoP] Exeter V40 Trilogy	1,945	1.05	0.66	[0.14 , 1.17]	Inferior by ≥20%	0.013
[CoP] Exeter V40 Tritanium	437	1.91	1.52	[0.60 , 2.45]	Inferior by ≥100%	0.001
[CoP] Furlong HAC Stem CSF	6,284	1.26	0.87	[0.48 , 1.26]	Inferior by ≥100%	<0.001
[CoP] Furlong HAC Stem Furlong HAC CSF Plus	1,538	1.84	1.45	[0.81 , 2.09]	Inferior by ≥100%	<0.001
[CoP] Furlong HAC Stem Furlong Threaded	388	0.50	0.11	[-0.64 , 0.86]	Non-inferiority not shown	0.778
[CoP] M/L Taper Cementless Continuum	391	2.79	2.40	[1.26 , 3.53]	Inferior by ≥100%	<0.001
[CoP] Muller Straight Stem Original ME Muller Low Profile Cup	364	1.22	0.83	[-0.19 , 1.85]	Non-inferiority not shown	0.110
[CoP] SL-Plus Cementless Stem Bicon-Plus	620	2.56	2.17	[0.93 , 3.40]	Inferior by ≥100%	0.001
[CoP] SL-Plus Cementless Stem EP-Fit Plus	958	1.89	1.50	[0.61 , 2.39]	Inferior by ≥100%	0.001
[CoP] SP II Cemented Stem Interplanta	310	0.00	- -	[-.-- , -.--]	No failures to date	
[CoP] Stanmore Modular Stem Stanmore-Arcom Cup	355	1.19	0.80	[-0.28 , 1.88]	Non-inferiority not shown	0.145
[CoP] Taperloc Cementless Stem Exceed ABT	1,879	1.07	0.68	[0.23 , 1.13]	Inferior by ≥20%	0.003
[CoP] Versys Cementless Stem Trilogy	363	1.07	0.68	[-0.40 , 1.76]	Non-inferiority not shown	0.220
[MoP] ABG II Monolithic Cementless Stem ABG II Cementless Cup	442	1.70	1.31	[0.10 , 2.51]	Inferior by ≥20%	0.033

For peer review only - http://bmjopen.bmj.com/site/about/guidelines.xhtml

[MoP] ABG II Monolithic Cementless Stem Trident	440	3.19	2.80	[1.18 , 4.42]	Inferior by ≥100%	0.001
[MoP] Accolade Trident	9,084	2.01	1.62	[1.23 , 2.02]	Inferior by ≥100%	<0.001
[MoP] Anthology R3 Cementless	1,155	1.76	1.36	[0.73 , 2.00]	Inferior by ≥100%	<0.001
[MoP] Bimetric Cementless Stem Mallory-Head Cementless Cup	359	2.05	1.66	[0.42 , 2.90]	Inferior by ≥100%	0.009
[MoP] C-Stem AMT Cemented Stem Charnley and Elite Plus LPW	2,153	1.15	0.76	[0.26 , 1.26]	Inferior by ≥20%	0.003
[MoP] C-Stem AMT Cemented Stem Elite Plus Cemented Cup	612	0.88	0.48	[-0.27 , 1.24]	Non-inferiority not shown	0.209
[MoP] C-Stem AMT Cemented Stem Elite Plus Ogee	1,608	0.77	0.38	[-0.09 , 0.85]	Non-inferiority not shown	0.116
[MoP] C-Stem AMT Cemented Stem Marathon	1,157	0.93	0.54	[0.07 , 1.00]	Non-inferiority not shown	0.023
[MoP] C-Stem AMT Cemented Stem Pinnacle	1,478	1.22	0.83	[0.35 , 1.31]	Inferior by ≥20%	0.001
[MoP] C-Stem Cemented Stem Charnley Ogee	847	1.31	0.92	[0.15 , 1.68]	Inferior by ≥20%	0.019
[MoP] C-Stem Cemented Stem Charnley and Elite Plus LPW	1,594	1.05	0.66	[0.10 , 1.23]	Inferior by ≥20%	0.022
[MoP] C-Stem Cemented Stem Duraloc Cementless Cup	535	2.10	1.71	[0.50 , 2.92]	Inferior by ≥100%	0.006
[MoP] C-Stem Cemented Stem Elite Plus Cemented Cup	650	0.56	0.17	[-0.45 , 0.79]	Non-inferiority not shown	0.590
[MoP] C-Stem Cemented Stem Elite Plus Ogee	3,208	0.88	0.49	[0.07 , 0.91]	Non-inferiority not shown	0.022
[MoP] C-Stem Cemented Stem Marathon	1,556	0.92	0.53	[0.05 , 1.00]	Non-inferiority not shown	0.029
[MoP] C-Stem Cemented Stem Opera	1,363	1.04	0.65	[0.05 , 1.25]	Non-inferiority not shown	0.033
[MoP] C-Stem Cemented Stem Pinnacle	417	1.56	1.17	[0.06 , 2.28]	Non-inferiority not shown	0.039
[MoP] C-Stem Cemented Stem Polarcup Cementless	268	0.71	0.32	[-0.70 , 1.35]	Non-inferiority not shown	0.539
[MoP] C-Stem Cemented Stem Trilogy	364	0.53	0.14	[-0.65 , 0.93]	Non-inferiority not shown	0.730
[MoP] C-Stem Cemented Stem Wroblewski Golf Ball	978	0.94	0.55	[-0.10 , 1.20]	Non-inferiority not shown	0.098
[MoP] CCA Cemented Stem CCB Cup	1,129	0.57	0.18	[-0.31 , 0.68]	Non-inferiority not shown	0.469
[MoP] CLS Cementless Stem Allofit	600	2.56	2.17	[0.93 , 3.40]	Inferior by ≥100%	0.001
[MoP] CLS Cementless Stem Trilogy	559	2.80	2.40	[1.06 , 3.75]	Inferior by ≥100%	<0.001
[MoP] CMK Modular Cemented Stem CMK Cemented Cup	462	0.41	0.02	[-0.62 , 0.67]	Non-inferiority not shown	0.943
[MoP] CPCS Opera	1,174	0.82	0.42	[-0.16 , 1.01]	Non-inferiority not shown	0.152
[MoP] CPCS Polarcup Cementless	400	0.49	0.09	[-0.64 , 0.83]	Non-inferiority not shown	0.801
[MoP] CPCS Reflection Cemented	382	1.02	0.63	[-0.14 , 1.40]	Non-inferiority not shown	0.107
[MoP] CPS Plus Cenator Cemented Cup	381	0.26	-0.13	[-0.72 , 0.46]	Non-inferiority not shown	0.662
[MoP] CPS Plus EP-Fit Plus	301	1.58	1.19	[-0.21 , 2.60]	Non-inferiority not shown	0.097
[MoP] CPS Plus Opera	490	1.55	1.16	[0.05 , 2.27]	Non-inferiority not shown	0.040
[MoP] CPT Allofit	686	0.55	0.16	[-0.38 , 0.69]	Non-inferiority not shown	0.561
[MoP] CPT Continuum	480	2.60	2.20	[1.33 , 3.08]	Inferior by ≥100%	<0.001
[MoP] CPT Elite Plus Ogee	2,410	1.38	0.99	[0.47 , 1.52]	Inferior by ≥100%	<0.001
[MoP] CPT Exceed	284	3.02	2.63	[0.66 , 4.59]	Inferior by ≥100%	0.009
[MoP] CPT Exeter Contemporary Flanged	386	2.10	1.71	[0.50 , 2.92]	Inferior by ≥100%	0.006
[MoP] CPT Opera	406	1.40	1.01	[-0.14 , 2.16]	Non-inferiority not shown	0.086
[MoP] CPT Original ME Muller Low Profile Cup	833	1.20	0.81	[0.09 , 1.53]	Inferior by ≥20%	0.028
[MoP] CPT Pinnacle	753	1.70	1.31	[0.38 , 2.24]	Inferior by ≥20%	0.006
[MoP] CPT Trabecular Metal Modular Cementless Cup	891	1.66	1.27	[0.50 , 2.03]	Inferior by ≥100%	0.001
[MoP] CPT Trilogy	8,372	1.37	0.98	[0.62 , 1.34]	Inferior by ≥100%	<0.001
[MoP] CPT Trilogy IT	450	2.89	2.50	[1.61 , 3.40]	Inferior by ≥100%	<0.001
[MoP] CPT ZCA	7,529	1.39	1.00	[0.63 , 1.37]	Inferior by ≥100%	<0.001
[MoP] Centrament Chirulen	378	0.24	-0.15	[-0.71 , 0.41]	Non-inferiority not shown	0.608
[MoP] Charnley Cemented Stem Charnley Cemented Cup	4,078	1.11	0.71	[0.29 , 1.14]	Inferior by ≥20%	0.001
[MoP] Charnley Cemented Stem Charnley Ogee	8,701	1.18	0.79	[0.43 , 1.15]	Inferior by ≥100%	<0.001
[MoP] Charnley Cemented Stem Charnley and Elite Plus LPW	5,770	0.72	0.32	[-0.03 , 0.68]	Non-inferiority not shown	0.075

For peer review only

[MoP] Charnley Cemented Stem Opera	1,229	0.85	0.46	[-0.12 , 1.04]	Non-inferiority not shown	0.119
[MoP] Charnley Cemented Stem Wroblewski Golf Ball	990	1.50	1.10	[0.32 , 1.89]	Inferior by ≥20%	0.006
[MoP] Charnley Modular Charnley and Elite Plus LPW	363	0.27	-0.12	[-0.73 , 0.49]	Non-inferiority not shown	0.702
[MoP] Corail Charnley and Elite Plus LPW	723	1.36	0.97	[0.18 , 1.76]	Inferior by ≥20%	0.017
[MoP] Corail Duraloc Cementless Cup	3,449	1.41	1.02	[0.54 , 1.50]	Inferior by ≥100%	<0.001
[MoP] Corail Elite Plus Cemented Cup	964	0.81	0.42	[-0.19 , 1.02]	Non-inferiority not shown	0.175
[MoP] Corail Elite Plus Ogee	1,407	1.22	0.83	[0.24 , 1.42]	Inferior by ≥20%	0.005
[MoP] Corail Exeter Contemporary Flanged	571	1.13	0.73	[-0.06 , 1.53]	Non-inferiority not shown	0.070
[MoP] Corail Marathon	2,848	1.05	0.66	[0.24 , 1.07]	Inferior by ≥20%	0.002
[MoP] Corail Pinnacle	28,253	1.37	0.98	[0.67 , 1.29]	Inferior by ≥100%	<0.001
[MoP] Corail Pinnacle Gription	382	1.54	1.15	[0.34 , 1.95]	Inferior by ≥20%	0.005
[MoP] Corail Trident	585	1.54	1.15	[0.33 , 1.96]	Inferior by ≥20%	0.006
[MoP] Corail Trilogy	1,791	1.14	0.74	[0.20 , 1.29]	Inferior by ≥20%	0.007
[MoP] Elite Plus Cemented Stem Charnley and Elite Plus LPW	331	1.66	1.27	[-0.08 , 2.62]	Non-inferiority not shown	0.065
[MoP] Elite Plus Cemented Stem Elite Plus Ogee	576	0.34	-0.05	[-0.60 , 0.50]	Non-inferiority not shown	0.848
[MoP] Excia Cementless Plasmacup SC	316	3.20	2.81	[1.00 , 4.62]	Inferior by ≥100%	0.002
[MoP] Exeter Elite Plus Ogee	282	0.33	-0.06	[-0.77 , 0.65]	Non-inferiority not shown	0.871
[MoP] Exeter V40 ABG II Cementless Cup	734	1.28	0.89	[0.05 , 1.73]	Non-inferiority not shown	0.038
[MoP] Exeter V40 Cenator Cemented Cup	2,129	1.40	1.01	[0.45 , 1.57]	Inferior by ≥100%	<0.001
[MoP] Exeter V40 Charnley Cemented Cup	314	0.87	0.48	[-0.55 , 1.51]	Non-inferiority not shown	0.358
[MoP] Exeter V40 Charnley Ogee	1,315	0.95	0.56	[-0.02 , 1.14]	Non-inferiority not shown	0.057
[MoP] Exeter V40 Charnley and Elite Plus LPW	2,477	1.25	0.86	[0.37 , 1.35]	Inferior by ≥20%	0.001
[MoP] Exeter V40 Duraloc Cementless Cup	1,061	1.46	1.06	[0.30 , 1.83]	Inferior by ≥20%	0.006
[MoP] Exeter V40 EP-Fit Plus	539	1.44	1.05	[0.02 , 2.08]	Non-inferiority not shown	0.046
[MoP] Exeter V40 Elite Plus Cemented Cup	3,599	0.58	0.19	[-0.18 , 0.56]	Non-inferiority not shown	0.314
[MoP] Exeter V40 Elite Plus Ogee	16,477	0.79	0.40	[0.08 , 0.71]	Inferior by ≥20%	0.014
[MoP] Exeter V40 Exceed	494	0.94	0.55	[-0.32 , 1.42]	Non-inferiority not shown	0.215
[MoP] Exeter V40 Exceed ABT	456	0.60	0.21	[-0.45 , 0.87]	Non-inferiority not shown	0.528
[MoP] Exeter V40 Exeter Contemporary Flanged	40,794	0.85	0.46	[0.15 , 0.76]	Inferior by ≥20%	0.003
[MoP] Exeter V40 Exeter Contemporary Hooded	16,021	1.53	1.14	[0.80 , 1.47]	Inferior by ≥100%	<0.001
[MoP] Exeter V40 Exeter Duration	12,935	1.23	0.84	[0.50 , 1.18]	Inferior by ≥100%	<0.001
[MoP] Exeter V40 Exeter X3 Rimfit	5,262	0.96	0.57	[0.23 , 0.92]	Inferior by ≥20%	0.001
[MoP] Exeter V40 Furlong HAC CSF Plus	504	0.35	-0.04	[-0.53 , 0.46]	Non-inferiority not shown	0.882
[MoP] Exeter V40 Marathon	1,300	1.05	0.66	[0.15 , 1.18]	Inferior by ≥20%	0.012
[MoP] Exeter V40 Opera	2,373	0.81	0.42	[-0.03 , 0.88]	Non-inferiority not shown	0.067
[MoP] Exeter V40 Pinnacle	2,590	1.18	0.79	[0.35 , 1.24]	Inferior by ≥20%	<0.001
[MoP] Exeter V40 R3 Cementless	461	1.23	0.84	[0.10 , 1.57]	Inferior by ≥20%	0.025
[MoP] Exeter V40 Reflection Cementless	2,035	1.12	0.73	[0.19 , 1.26]	Inferior by ≥20%	0.007
[MoP] Exeter V40 Trabecular Metal Modular Cementless Cup	411	1.22	0.83	[-0.12 , 1.77]	Non-inferiority not shown	0.086
[MoP] Exeter V40 Trabecular Metal Natural Cup	318	3.43	3.04	[1.11 , 4.97]	Inferior by ≥100%	0.002
[MoP] Exeter V40 Trident	17,802	1.10	0.71	[0.39 , 1.02]	Inferior by ≥20%	<0.001
[MoP] Exeter V40 Trilogy	8,482	0.92	0.53	[0.19 , 0.88]	Inferior by ≥20%	0.003
[MoP] Exeter V40 Tritanium	475	2.06	1.67	[0.71 , 2.63]	Inferior by ≥100%	0.001
[MoP] Exeter V40 Ultima Cemented Cup	1,139	1.56	1.17	[0.41 , 1.92]	Inferior by ≥100%	0.002
[MoP] Exeter V40 ZCA	274	0.36	-0.03	[-0.79 , 0.73]	Non-inferiority not shown	0.933
[MoP] Furlong Cemented Stem JRI Cemented Cup	1,478	1.38	0.99	[0.34 , 1.63]	Inferior by ≥20%	0.003

For peer review only

[MoP] Furlong HAC Stem CSF	6,640	2.07	1.68	[1.25 , 2.11]	Inferior by ≥100%	<0.001
[MoP] Furlong HAC Stem Furlong HAC CSF Plus	2,998	2.24	1.85	[1.33 , 2.37]	Inferior by ≥100%	<0.001
[MoP] Furlong HAC Stem Furlong Threaded	479	2.13	1.74	[0.46 , 3.02]	Inferior by ≥100%	0.008
[MoP] Furlong HAC Stem Trilogy	341	1.90	1.51	[0.09 , 2.94]	Inferior by ≥20%	0.038
[MoP] M/L Taper Cementless Allofit	543	1.27	0.88	[0.00 , 1.76]	Non-inferiority not shown	0.049
[MoP] M/L Taper Cementless Continuum	612	1.40	1.01	[0.28 , 1.74]	Inferior by ≥20%	0.007
[MoP] M/L Taper Cementless Trilogy	475	2.08	1.69	[0.63 , 2.74]	Inferior by ≥100%	0.002
[MoP] M/L Taper Cementless Trilogy IT	270	1.86	1.47	[0.46 , 2.47]	Inferior by ≥100%	0.004
[MoP] MS-30 Allofit	314	0.57	0.17	[-0.66 , 1.01]	Non-inferiority not shown	0.683
[MoP] MS-30 Original ME Muller Low Profile Cup	756	0.58	0.19	[-0.36 , 0.74]	Non-inferiority not shown	0.497
[MoP] Mem Original ME Muller Low Profile Cup	337	0.56	0.17	[-0.66 , 1.00]	Non-inferiority not shown	0.688
[MoP] Muller Straight Stem Centerpulse Muller	502	0.95	0.56	[-0.32 , 1.43]	Non-inferiority not shown	0.213
[MoP] Muller Straight Stem Original ME Muller Low Profile Cup	1,412	0.81	0.41	[-0.09 , 0.92]	Non-inferiority not shown	0.106
[MoP] Muller-Biomet Apollo	1,949	1.24	0.85	[0.30 , 1.41]	Inferior by ≥20%	0.003
[MoP] Muller-Biomet Original ME Muller Low Profile Cup	831	1.56	1.17	[0.31 , 2.03]	Inferior by ≥20%	0.008
[MoP] Omnifit Cemented Stem ODC	880	1.40	1.01	[0.20 , 1.82]	Inferior by ≥20%	0.015
[MoP] Omnifit Cemented Stem Trident	270	0.36	-0.03	[-0.80 , 0.74]	Non-inferiority not shown	0.947
[MoP] Omnifit Cementless Stem Secure Fit Cementless Cup	283	0.69	0.30	[-0.70 , 1.30]	Non-inferiority not shown	0.554
[MoP] Omnifit Cementless Stem Trident	426	3.34	2.95	[1.31 , 4.58]	Inferior by ≥100%	<0.001
[MoP] P10 Muller Original ME Muller Low Profile Cup	415	0.96	0.57	[-0.33 , 1.47]	Non-inferiority not shown	0.213
[MoP] Polarstem Cementless R3 Cementless	1,068	0.96	0.57	[0.14 , 1.00]	Inferior by ≥20%	0.010
[MoP] SL-Plus Cementless Stem EP-Fit Plus	2,023	2.60	2.21	[1.52 , 2.90]	Inferior by ≥100%	<0.001
[MoP] SP II Cemented Stem Interplanta	625	2.72	2.33	[1.09 , 3.57]	Inferior by ≥100%	<0.001
[MoP] SP II Cemented Stem Link Flange Cup	274	0.69	0.29	[-0.70 , 1.29]	Non-inferiority not shown	0.560
[MoP] Spectron Reflection Cementless	298	1.24	0.84	[-0.39 , 2.08]	Non-inferiority not shown	0.182
[MoP] Stanmore Modular Stem Elite Plus Cemented Cup	534	0.35	-0.05	[-0.61 , 0.51]	Non-inferiority not shown	0.872
[MoP] Stanmore Modular Stem SHP Cup	931	0.82	0.43	[-0.18 , 1.03]	Non-inferiority not shown	0.169
[MoP] Stanmore Modular Stem Stanmore-Arcom Cup	3,798	1.09	0.70	[0.27 , 1.12]	Inferior by ≥20%	0.001
[MoP] Synergy Cementless Stem R3 Cementless	1,057	1.25	0.86	[0.31 , 1.41]	Inferior by ≥20%	0.002
[MoP] Synergy Cementless Stem Reflection Cementless	1,449	0.87	0.47	[-0.08 , 1.03]	Non-inferiority not shown	0.092
[MoP] Taperfit Cemented Stem Atlas IIIp	444	0.84	0.45	[-0.34 , 1.24]	Non-inferiority not shown	0.268
[MoP] Taperloc Cemented Stem Exceed ABT	280	0.97	0.58	[-0.21 , 1.38]	Non-inferiority not shown	0.152
[MoP] Taperloc Cementless Stem Exceed	553	1.20	0.81	[-0.12 , 1.73]	Non-inferiority not shown	0.089
[MoP] Taperloc Cementless Stem Exceed ABT	3,732	1.75	1.36	[0.91 , 1.80]	Inferior by ≥100%	<0.001
[MoP] Trilock BPS Pinnacle	264	1.65	1.26	[0.01 , 2.51]	Non-inferiority not shown	0.048
[MoP] Trilock BPS Pinnacle Gription	275	1.37	0.98	[-0.19 , 2.15]	Non-inferiority not shown	0.100
[MoP] VerSys Cemented Stem Trilogy	263	1.11	0.71	[-0.56 , 1.99]	Non-inferiority not shown	0.273
[MoP] Versys Cementless Stem Trilogy	860	4.01	3.62	[2.32 , 4.91]	Inferior by ≥100%	<0.001

For peer review only

Supplementary table 1b: Difference in Kaplan-Meier failure estimate between a contemporary reference and implants with at least 250 at risk at 5 years since primary

Stem/cup brand	Number at risk	Cumulative failure (%)	Difference in failure (%)	95% CI	Equivalence status	p-value
[CoP] MS-30 Original ME Muller Low Profile Cup	1,125	0.55	[REFERENCE]			
[CoC] ABG II Monolithic Cementless Stem ABG II Cementless Cup	1,020	2.29	1.73	[0.77 , 2.70]	Inferior by ≥100%	<0.001
[CoC] ABG II Monolithic Cementless Stem Trident	834	3.07	2.51	[1.38 , 3.64]	Inferior by ≥100%	<0.001
[CoC] Accolade Trident	5,169	2.83	2.27	[1.74 , 2.81]	Inferior by ≥100%	<0.001
[CoC] Bicontact Cementless Stem Plasmacup SC	289	1.01	0.46	[-0.60 , 1.51]	Non-inferiority not shown	0.395
[CoC] Bimetric Cementless Stem Exceed ABT	627	1.80	1.24	[0.31 , 2.17]	Inferior by ≥20%	0.009
[CoC] C-Stem AMT Cemented Stem Pinnacle	427	1.30	0.74	[-0.10 , 1.59]	Non-inferiority not shown	0.085
[CoC] C-Stem Cemented Stem Pinnacle	310	1.44	0.88	[-0.42 , 2.19]	Non-inferiority not shown	0.185
[CoC] CPT Continuum	379	2.40	1.85	[0.77 , 2.92]	Inferior by ≥100%	0.001
[CoC] CPT Trilogy AB	468	1.70	1.15	[-0.02 , 2.31]	Non-inferiority not shown	0.053
[CoC] Corail DeltaMotion	633	1.64	1.08	[0.28 , 1.89]	Inferior by ≥20%	0.008
[CoC] Corail Duraloc Option	1,014	2.99	2.44	[1.35 , 3.52]	Inferior by ≥100%	<0.001
[CoC] Corail Pinnacle	17,510	2.40	1.85	[1.44 , 2.26]	Inferior by ≥100%	<0.001
[CoC] Excia Cementless Plasmacup SC	501	1.33	0.77	[0.00 , 1.54]	Non-inferiority not shown	0.049
[CoC] Exeter V40 ABG II Cementless Cup	1,138	1.20	0.64	[-0.04 , 1.32]	Non-inferiority not shown	0.063
[CoC] Exeter V40 Trident	8,141	1.50	0.94	[0.51 , 1.38]	Inferior by ≥20%	<0.001
[CoC] Furlong HAC Stem CSF	1,502	2.59	2.03	[1.18 , 2.89]	Inferior by ≥100%	<0.001
[CoC] Furlong HAC Stem Furlong HAC CSF Plus	5,470	1.84	1.28	[0.84 , 1.73]	Inferior by ≥100%	<0.001
[CoC] Furlong HAC Stem Furlong Threaded	318	3.01	2.46	[0.58 , 4.34]	Inferior by ≥100%	0.010
[CoC] M/L Taper Cementless Continuum	607	1.71	1.15	[0.46 , 1.85]	Inferior by ≥20%	0.001
[CoC] Metafix Stem Trinity	280	1.52	0.96	[0.14 , 1.79]	Inferior by ≥20%	0.022
[CoC] Omnifit Cementless Stem Trident	387	3.10	2.54	[0.95 , 4.13]	Inferior by ≥100%	0.002
[CoC] Polarstem Cementless R3 Cementless	300	0.78	0.22	[-0.40 , 0.85]	Non-inferiority not shown	0.487
[CoC] S-Rom Cementless Stem Pinnacle	407	4.35	3.79	[2.12 , 5.46]	Inferior by ≥100%	<0.001
[CoC] SL-Plus Cementless Stem EP-Fit Plus	1,115	4.96	4.40	[3.14 , 5.66]	Inferior by ≥100%	<0.001
[CoC] SPS Modular April - Ceramic	321	2.99	2.43	[0.95 , 3.92]	Inferior by ≥100%	0.001
[CoC] Summit Cementless Stem Pinnacle	385	1.33	0.78	[-0.18 , 1.73]	Non-inferiority not shown	0.110
[CoC] Taperloc Cementless Stem Exceed ABT	3,900	1.83	1.27	[0.80 , 1.74]	Inferior by ≥100%	<0.001
[CoP] Accolade Trident	1,372	1.95	1.39	[0.82 , 1.97]	Inferior by ≥100%	<0.001
[CoP] C-Stem Cemented Stem Elite Plus Ogee	474	0.75	0.20	[-0.56 , 0.96]	Non-inferiority not shown	0.609
[CoP] C-Stem Cemented Stem Marathon	542	1.34	0.79	[0.10 , 1.47]	Non-inferiority not shown	0.024
[CoP] C-Stem Cemented Stem Opera	656	1.33	0.77	[-0.12 , 1.67]	Non-inferiority not shown	0.091
[CoP] C-Stem Cemented Stem Wroblewski Golf Ball	738	1.05	0.49	[-0.25 , 1.24]	Non-inferiority not shown	0.196
[CoP] CPT ZCA	261	0.39	-0.17	[-0.82 , 0.48]	Non-inferiority not shown	0.611
[CoP] Corail Charnley and Elite Plus LPW	532	1.89	1.33	[0.25 , 2.42]	Inferior by ≥20%	0.016
[CoP] Corail Duraloc Cementless Cup	289	4.72	4.17	[1.81 , 6.53]	Inferior by ≥100%	0.001
[CoP] Corail Elite Plus Cemented Cup	328	1.50	0.95	[-0.24 , 2.13]	Non-inferiority not shown	0.117
[CoP] Corail Elite Plus Ogee	340	2.64	2.08	[0.61 , 3.55]	Inferior by ≥100%	0.006
[CoP] Corail Marathon	573	1.40	0.84	[0.19 , 1.49]	Inferior by ≥20%	0.012
[CoP] Corail Pinnacle	4,134	1.74	1.18	[0.74 , 1.63]	Inferior by ≥100%	<0.001

For peer review only

[CoP] Corail Trilogy	592	1.61	1.06	[0.04 , 2.07]	Non-inferiority not shown	0.041
[CoP] Exeter V40 Charnley and Elite Plus LPW	433	2.02	1.47	[0.38 , 2.55]	Inferior by ≥20%	0.008
[CoP] Exeter V40 Elite Plus Ogee	858	1.08	0.53	[-0.14 , 1.20]	Non-inferiority not shown	0.122
[CoP] Exeter V40 Exeter Contemporary Flanged	2,011	1.31	0.76	[0.23 , 1.28]	Inferior by ≥20%	0.005
[CoP] Exeter V40 Exeter Contemporary Hooded	593	2.58	2.03	[0.93 , 3.12]	Inferior by ≥100%	<0.001
[CoP] Exeter V40 Exeter Duration	664	0.95	0.40	[-0.36 , 1.16]	Non-inferiority not shown	0.304
[CoP] Exeter V40 Exeter X3 Rimfit	363	1.16	0.60	[0.09 , 1.12]	Non-inferiority not shown	0.022
[CoP] Exeter V40 Marathon	258	0.94	0.38	[-0.44 , 1.21]	Non-inferiority not shown	0.365
[CoP] Exeter V40 Pinnacle	297	1.53	0.98	[0.08 , 1.87]	Non-inferiority not shown	0.032
[CoP] Exeter V40 Trident	2,337	1.16	0.60	[0.15 , 1.06]	Inferior by ≥20%	0.009
[CoP] Exeter V40 Trilogy	1,627	1.31	0.76	[0.15 , 1.37]	Inferior by ≥20%	0.015
[CoP] Furlong HAC Stem CSF	5,425	1.65	1.09	[0.61 , 1.57]	Inferior by ≥100%	<0.001
[CoP] Furlong HAC Stem Furlong HAC CSF Plus	908	2.02	1.47	[0.74 , 2.19]	Inferior by ≥100%	<0.001
[CoP] Furlong HAC Stem Furlong Threaded	362	1.29	0.73	[-0.45 , 1.91]	Non-inferiority not shown	0.225
[CoP] Muller Straight Stem Original ME Muller Low Profile Cup	298	1.22	0.67	[-0.38 , 1.71]	Non-inferiority not shown	0.210
[CoP] SL-Plus Cementless Stem Bicon-Plus	558	4.05	3.50	[1.93 , 5.07]	Inferior by ≥100%	<0.001
[CoP] SL-Plus Cementless Stem EP-Fit Plus	810	3.41	2.86	[1.65 , 4.06]	Inferior by ≥100%	<0.001
[CoP] Stanmore Modular Stem Stanmore-Arcom Cup	257	1.19	0.64	[-0.46 , 1.74]	Non-inferiority not shown	0.257
[CoP] Taperloc Cementless Stem Exceed ABT	775	1.43	0.87	[0.26 , 1.49]	Inferior by ≥20%	0.006
[CoP] Versys Cementless Stem Trilogy	342	1.07	0.51	[-0.59 , 1.61]	Non-inferiority not shown	0.363
[MoP] ABG II Monolithic Cementless Stem ABG II Cementless Cup	419	1.92	1.37	[0.07 , 2.66]	Non-inferiority not shown	0.039
[MoP] ABG II Monolithic Cementless Stem Trident	402	4.11	3.56	[1.71 , 5.41]	Inferior by ≥100%	<0.001
[MoP] Accolade Trident	6,100	2.76	2.21	[1.72 , 2.70]	Inferior by ≥100%	<0.001
[MoP] Anthology R3 Cementless	353	2.14	1.59	[0.69 , 2.48]	Inferior by ≥100%	<0.001
[MoP] Bimetric Cementless Stem Mallory-Head Cementless Cup	271	2.05	1.50	[0.23 , 2.76]	Inferior by ≥20%	0.020
[MoP] C-Stem AMT Cemented Stem Charnley and Elite Plus LPW	1,397	1.46	0.90	[0.30 , 1.51]	Inferior by ≥20%	0.003
[MoP] C-Stem AMT Cemented Stem Elite Plus Cemented Cup	435	1.22	0.66	[-0.26 , 1.58]	Non-inferiority not shown	0.157
[MoP] C-Stem AMT Cemented Stem Elite Plus Ogee	918	1.01	0.46	[-0.13 , 1.05]	Non-inferiority not shown	0.130
[MoP] C-Stem AMT Cemented Stem Marathon	356	1.46	0.90	[0.10 , 1.71]	Non-inferiority not shown	0.028
[MoP] C-Stem AMT Cemented Stem Pinnacle	657	1.85	1.29	[0.56 , 2.02]	Inferior by ≥100%	0.001
[MoP] C-Stem Cemented Stem Charnley Ogee	680	1.86	1.30	[0.34 , 2.26]	Inferior by ≥20%	0.008
[MoP] C-Stem Cemented Stem Charnley and Elite Plus LPW	1,435	1.57	1.02	[0.31 , 1.72]	Inferior by ≥20%	0.005
[MoP] C-Stem Cemented Stem Duraloc Cementless Cup	496	2.67	2.12	[0.73 , 3.50]	Inferior by ≥100%	0.003
[MoP] C-Stem Cemented Stem Elite Plus Cemented Cup	586	0.90	0.34	[-0.46 , 1.15]	Non-inferiority not shown	0.405
[MoP] C-Stem Cemented Stem Elite Plus Ogee	2,459	1.13	0.57	[0.06 , 1.08]	Non-inferiority not shown	0.028
[MoP] C-Stem Cemented Stem Marathon	744	1.25	0.70	[0.08 , 1.32]	Non-inferiority not shown	0.028
[MoP] C-Stem Cemented Stem Opera	1,013	1.44	0.89	[0.16 , 1.62]	Inferior by ≥20%	0.017
[MoP] C-Stem Cemented Stem Pinnacle	331	1.56	1.01	[-0.13 , 2.14]	Non-inferiority not shown	0.083
[MoP] C-Stem Cemented Stem Polarcup Cementless	255	0.71	0.16	[-0.89 , 1.21]	Non-inferiority not shown	0.768
[MoP] C-Stem Cemented Stem Trilogy	338	0.82	0.26	[-0.73 , 1.25]	Non-inferiority not shown	0.606
[MoP] C-Stem Cemented Stem Wroblewski Golf Ball	756	1.50	0.94	[0.10 , 1.79]	Non-inferiority not shown	0.028
[MoP] CCA Cemented Stem CCB Cup	738	0.79	0.23	[-0.38 , 0.85]	Non-inferiority not shown	0.459
[MoP] CLS Cementless Stem Allofit	553	2.90	2.34	[1.01 , 3.68]	Inferior by ≥100%	0.001
[MoP] CLS Cementless Stem Trilogy	419	3.18	2.63	[1.17 , 4.09]	Inferior by ≥100%	<0.001
[MoP] CMK Modular Cemented Stem CMK Cemented Cup	418	1.08	0.52	[-0.49 , 1.53]	Non-inferiority not shown	0.310
[MoP] CPCS Opera	866	1.28	0.72	[-0.02 , 1.46]	Non-inferiority not shown	0.056

For peer review only

[MoP] CPCS Polarcup Cementless	368	0.99	0.43	[-0.60 , 1.46]	Non-inferiority not shown	0.410
[MoP] CPS Plus Cenator Cemented Cup	339	0.80	0.25	[-0.73 , 1.23]	Non-inferiority not shown	0.618
[MoP] CPS Plus EP-Fit Plus	282	3.23	2.68	[0.67 , 4.69]	Inferior by ≥100%	0.009
[MoP] CPS Plus Opera	464	2.16	1.61	[0.29 , 2.92]	Inferior by ≥20%	0.017
[MoP] CPT Allofit	430	0.94	0.39	[-0.41 , 1.18]	Non-inferiority not shown	0.339
[MoP] CPT Elite Plus Ogee	1,744	1.87	1.32	[0.67 , 1.97]	Inferior by ≥100%	<0.001
[MoP] CPT Exceed	263	4.10	3.55	[1.24 , 5.85]	Inferior by ≥100%	0.003
[MoP] CPT Opera	378	2.17	1.62	[0.16 , 3.07]	Inferior by ≥20%	0.029
[MoP] CPT Original ME Muller Low Profile Cup	422	2.20	1.64	[0.54 , 2.75]	Inferior by ≥20%	0.004
[MoP] CPT Pinnacle	639	2.42	1.86	[0.72 , 3.00]	Inferior by ≥100%	0.001
[MoP] CPT Trabecular Metal Modular Cementless Cup	523	2.41	1.85	[0.82 , 2.89]	Inferior by ≥100%	<0.001
[MoP] CPT Trilogy	5,765	2.20	1.65	[1.17 , 2.12]	Inferior by ≥100%	<0.001
[MoP] CPT ZCA	5,547	2.11	1.55	[1.07 , 2.03]	Inferior by ≥100%	<0.001
[MoP] Centrament Chirulen	310	1.12	0.57	[-0.59 , 1.72]	Non-inferiority not shown	0.337
[MoP] Charnley Cemented Stem Charnley Cemented Cup	3,601	1.72	1.17	[0.63 , 1.71]	Inferior by ≥100%	<0.001
[MoP] Charnley Cemented Stem Charnley Ogee	7,533	1.88	1.33	[0.86 , 1.79]	Inferior by ≥100%	<0.001
[MoP] Charnley Cemented Stem Charnley and Elite Plus LPW	5,041	1.12	0.56	[0.11 , 1.02]	Non-inferiority not shown	0.015
[MoP] Charnley Cemented Stem Opera	1,039	1.46	0.91	[0.14 , 1.67]	Inferior by ≥20%	0.020
[MoP] Charnley Cemented Stem Wroblewski Golf Ball	797	1.74	1.18	[0.30 , 2.06]	Inferior by ≥20%	0.008
[MoP] Charnley Modular Charnley and Elite Plus LPW	277	0.27	-0.28	[-0.93 , 0.37]	Non-inferiority not shown	0.393
[MoP] Corail Charnley and Elite Plus LPW	448	1.85	1.30	[0.30 , 2.29]	Inferior by ≥20%	0.010
[MoP] Corail Duraloc Cementless Cup	3,226	2.27	1.71	[1.10 , 2.33]	Inferior by ≥100%	<0.001
[MoP] Corail Elite Plus Cemented Cup	727	1.31	0.75	[-0.05 , 1.56]	Non-inferiority not shown	0.067
[MoP] Corail Elite Plus Ogee	1,029	1.70	1.14	[0.41 , 1.88]	Inferior by ≥20%	0.002
[MoP] Corail Exeter Contemporary Flanged	328	1.35	0.79	[-0.14 , 1.72]	Non-inferiority not shown	0.095
[MoP] Corail Marathon	1,385	1.23	0.67	[0.17 , 1.18]	Inferior by ≥20%	0.009
[MoP] Corail Pinnacle	16,408	1.70	1.15	[0.76 , 1.54]	Inferior by ≥100%	<0.001
[MoP] Corail Trilogy	1,383	1.72	1.17	[0.47 , 1.87]	Inferior by ≥20%	0.001
[MoP] Elite Plus Cemented Stem Charnley and Elite Plus LPW	308	1.66	1.11	[-0.26 , 2.47]	Non-inferiority not shown	0.113
[MoP] Elite Plus Cemented Stem Elite Plus Ogee	519	1.06	0.50	[-0.42 , 1.42]	Non-inferiority not shown	0.284
[MoP] Exeter Elite Plus Ogee	259	0.71	0.15	[-0.89 , 1.20]	Non-inferiority not shown	0.775
[MoP] Exeter V40 ABG II Cementless Cup	693	1.56	1.00	[0.05 , 1.95]	Non-inferiority not shown	0.038
[MoP] Exeter V40 Cenator Cemented Cup	1,725	1.99	1.44	[0.75 , 2.12]	Inferior by ≥100%	<0.001
[MoP] Exeter V40 Charnley Cemented Cup	286	0.87	0.32	[-0.73 , 1.37]	Non-inferiority not shown	0.553
[MoP] Exeter V40 Charnley Ogee	1,113	1.44	0.88	[0.15 , 1.61]	Inferior by ≥20%	0.018
[MoP] Exeter V40 Charnley and Elite Plus LPW	1,812	1.47	0.91	[0.34 , 1.48]	Inferior by ≥20%	0.002
[MoP] Exeter V40 Duraloc Cementless Cup	1,000	2.04	1.48	[0.56 , 2.40]	Inferior by ≥100%	0.002
[MoP] Exeter V40 EP-Fit Plus	496	2.56	2.01	[0.63 , 3.38]	Inferior by ≥100%	0.004
[MoP] Exeter V40 Elite Plus Cemented Cup	2,674	0.76	0.21	[-0.25 , 0.66]	Non-inferiority not shown	0.376
[MoP] Exeter V40 Elite Plus Ogee	12,458	1.11	0.56	[0.16 , 0.96]	Inferior by ≥20%	0.006
[MoP] Exeter V40 Exceed	375	0.94	0.39	[-0.51 , 1.29]	Non-inferiority not shown	0.399
[MoP] Exeter V40 Exceed ABT	305	1.17	0.62	[-0.43 , 1.67]	Non-inferiority not shown	0.251
[MoP] Exeter V40 Exeter Contemporary Flanged	26,691	1.21	0.65	[0.27 , 1.03]	Inferior by ≥20%	0.001
[MoP] Exeter V40 Exeter Contemporary Hooded	11,227	2.10	1.54	[1.12 , 1.96]	Inferior by ≥100%	<0.001
[MoP] Exeter V40 Exeter Duration	10,271	1.70	1.15	[0.72 , 1.57]	Inferior by ≥100%	<0.001
[MoP] Exeter V40 Exeter X3 Rimfit	859	1.16	0.61	[0.14 , 1.07]	Inferior by ≥20%	0.010

For peer review only

[MoP] Exeter V40 Marathon	555	1.54	0.99	[0.24 , 1.73]	Inferior by ≥20%	0.010
[MoP] Exeter V40 Opera	1,734	1.18	0.63	[0.06 , 1.19]	Non-inferiority not shown	0.030
[MoP] Exeter V40 Pinnacle	1,546	1.60	1.05	[0.48 , 1.62]	Inferior by ≥20%	<0.001
[MoP] Exeter V40 Reflection Cementless	1,812	1.58	1.02	[0.37 , 1.67]	Inferior by ≥20%	0.002
[MoP] Exeter V40 Trabecular Metal Modular Cementless Cup	276	2.23	1.68	[0.19 , 3.17]	Inferior by ≥20%	0.028
[MoP] Exeter V40 Trabecular Metal Natural Cup	284	4.06	3.51	[1.39 , 5.63]	Inferior by ≥100%	0.001
[MoP] Exeter V40 Trident	10,507	1.44	0.88	[0.48 , 1.29]	Inferior by ≥20%	<0.001
[MoP] Exeter V40 Trilogy	6,611	1.33	0.77	[0.34 , 1.21]	Inferior by ≥20%	0.001
[MoP] Exeter V40 Ultima Cemented Cup	1,040	2.19	1.64	[0.73 , 2.55]	Inferior by ≥100%	<0.001
[MoP] Furlong Cemented Stem JRI Cemented Cup	1,287	1.66	1.11	[0.37 , 1.84]	Inferior by ≥20%	0.003
[MoP] Furlong HAC Stem CSF	5,416	2.43	1.87	[1.36 , 2.38]	Inferior by ≥100%	<0.001
[MoP] Furlong HAC Stem Furlong HAC CSF Plus	1,808	2.91	2.35	[1.70 , 3.01]	Inferior by ≥100%	<0.001
[MoP] Furlong HAC Stem Furlong Threaded	416	3.01	2.46	[0.91 , 4.00]	Inferior by ≥100%	0.002
[MoP] Furlong HAC Stem Trilogy	264	1.90	1.35	[-0.10 , 2.80]	Non-inferiority not shown	0.067
[MoP] M/L Taper Cementless Allofit	295	2.04	1.48	[0.22 , 2.74]	Inferior by ≥20%	0.021
[MoP] M/L Taper Cementless Trilogy	328	2.54	1.98	[0.73 , 3.23]	Inferior by ≥100%	0.002
[MoP] MS-30 Allofit	269	0.57	0.01	[-0.85 , 0.87]	Non-inferiority not shown	0.981
[MoP] MS-30 Original ME Muller Low Profile Cup	531	1.23	0.67	[-0.19 , 1.54]	Non-inferiority not shown	0.128
[MoP] Mem Original ME Muller Low Profile Cup	313	0.86	0.30	[-0.73 , 1.34]	Non-inferiority not shown	0.567
[MoP] Muller Straight Stem Centerpulse Muller	442	1.79	1.24	[0.02 , 2.46]	Non-inferiority not shown	0.047
[MoP] Muller Straight Stem Original ME Muller Low Profile Cup	1,008	1.12	0.56	[-0.07 , 1.19]	Non-inferiority not shown	0.080
[MoP] Muller-Biomet Apollo	1,539	1.41	0.86	[0.23 , 1.49]	Inferior by ≥20%	0.008
[MoP] Muller-Biomet Original ME Muller Low Profile Cup	512	1.96	1.41	[0.41 , 2.40]	Inferior by ≥20%	0.006
[MoP] Omnifit Cemented Stem ODC	801	1.98	1.43	[0.45 , 2.41]	Inferior by ≥20%	0.004
[MoP] Omnifit Cemented Stem Trident	250	1.89	1.34	[-0.35 , 3.02]	Non-inferiority not shown	0.120
[MoP] Omnifit Cementless Stem Secure Fit Cementless Cup	275	0.69	0.14	[-0.89 , 1.16]	Non-inferiority not shown	0.791
[MoP] Omnifit Cementless Stem Trident	385	5.46	4.90	[2.78 , 7.02]	Inferior by ≥100%	<0.001
[MoP] P10 Muller Original ME Muller Low Profile Cup	293	0.96	0.41	[-0.52 , 1.34]	Non-inferiority not shown	0.388
[MoP] SL-Plus Cementless Stem EP-Fit Plus	1,651	3.51	2.96	[2.12 , 3.80]	Inferior by ≥100%	<0.001
[MoP] SP II Cemented Stem Interplanta	552	3.23	2.67	[1.29 , 4.05]	Inferior by ≥100%	<0.001
[MoP] Spectron Reflection Cementless	267	1.24	0.68	[-0.58 , 1.94]	Non-inferiority not shown	0.289
[MoP] Stanmore Modular Stem Elite Plus Cemented Cup	406	0.98	0.42	[-0.51 , 1.36]	Non-inferiority not shown	0.375
[MoP] Stanmore Modular Stem SHP Cup	677	0.95	0.40	[-0.30 , 1.10]	Non-inferiority not shown	0.265
[MoP] Stanmore Modular Stem Stanmore-Arcom Cup	2,920	1.62	1.06	[0.52 , 1.60]	Inferior by ≥20%	<0.001
[MoP] Synergy Cementless Stem Reflection Cementless	1,370	1.43	0.87	[0.16 , 1.59]	Inferior by ≥20%	0.016
[MoP] Taperfit Cemented Stem Atlas IIIp	250	2.18	1.63	[0.20 , 3.06]	Inferior by ≥20%	0.026
[MoP] Taperloc Cementless Stem Exceed	470	1.57	1.01	[-0.07 , 2.10]	Non-inferiority not shown	0.066
[MoP] Taperloc Cementless Stem Exceed ABT	1,793	2.07	1.52	[0.98 , 2.06]	Inferior by ≥100%	<0.001
[MoP] Versys Cementless Stem Trilogy	797	4.81	4.26	[2.82 , 5.69]	Inferior by ≥100%	<0.001

For peer review only

Supplementary table 1c: Difference in Kaplan-Meier failure estimate between a contemporary reference and implants with at least 250 at risk at 7 years since primary

Stem/cup brand	Number at risk	Cumulative failure (%)	Difference in failure (%)	95% CI	Equivalence status	p-value
[MoP] Exeter V40 Elite Plus Cemented Cup	1,773	0.91	[REFERENCE]			
[CoC] ABG II Monolithic Cementless Stem ABG II Cementless Cup	909	3.09	2.19	[1.10 , 3.27]	Inferior by ≥100%	<0.001
[CoC] ABG II Monolithic Cementless Stem Trident	705	3.68	2.77	[1.55 , 4.00]	Inferior by ≥100%	<0.001
[CoC] Accolade Trident	2,978	3.31	2.40	[1.86 , 2.95]	Inferior by ≥100%	<0.001
[CoC] C-Stem Cemented Stem Pinnacle	286	1.44	0.53	[-0.76 , 1.82]	Non-inferiority not shown	0.420
[CoC] CPT Trilogy AB	265	1.93	1.03	[-0.20 , 2.26]	Non-inferiority not shown	0.101
[CoC] Corail Duraloc Option	819	3.61	2.71	[1.54 , 3.88]	Inferior by ≥100%	<0.001
[CoC] Corail Pinnacle	7,589	2.93	2.03	[1.65 , 2.40]	Inferior by ≥100%	<0.001
[CoC] Exeter V40 ABG II Cementless Cup	918	1.69	0.79	[0.01 , 1.57]	Non-inferiority not shown	0.048
[CoC] Exeter V40 Trident	5,732	1.99	1.09	[0.67 , 1.51]	Inferior by ≥20%	<0.001
[CoC] Furlong HAC Stem CSF	1,405	3.19	2.29	[1.37 , 3.21]	Inferior by ≥100%	<0.001
[CoC] Furlong HAC Stem Furlong HAC CSF Plus	2,083	2.15	1.25	[0.81 , 1.69]	Inferior by ≥20%	<0.001
[CoC] Furlong HAC Stem Furlong Threaded	308	4.24	3.34	[1.14 , 5.53]	Inferior by ≥100%	0.003
[CoC] Omnifit Cementless Stem Trident	313	4.21	3.31	[1.41 , 5.21]	Inferior by ≥100%	0.001
[CoC] SL-Plus Cementless Stem EP-Fit Plus	968	5.32	4.41	[3.12 , 5.70]	Inferior by ≥100%	<0.001
[CoC] Summit Cementless Stem Pinnacle	301	1.60	0.69	[-0.37 , 1.76]	Non-inferiority not shown	0.203
[CoC] Taperloc Cementless Stem Exceed ABT	1,609	2.02	1.12	[0.65 , 1.58]	Inferior by ≥20%	<0.001
[CoP] Accolade Trident	544	2.20	1.29	[0.64 , 1.95]	Inferior by ≥20%	<0.001
[CoP] C-Stem Cemented Stem Elite Plus Ogee	342	0.75	-0.15	[-0.89 , 0.58]	Non-inferiority not shown	0.684
[CoP] C-Stem Cemented Stem Opera	478	1.69	0.79	[-0.22 , 1.80]	Non-inferiority not shown	0.127
[CoP] C-Stem Cemented Stem Wroblewski Golf Ball	598	1.67	0.76	[-0.18 , 1.70]	Non-inferiority not shown	0.112
[CoP] Corail Charnley and Elite Plus LPW	335	2.49	1.59	[0.32 , 2.85]	Inferior by ≥20%	0.014
[CoP] Corail Duraloc Cementless Cup	266	5.06	4.15	[1.72 , 6.59]	Inferior by ≥100%	0.001
[CoP] Corail Pinnacle	1,638	2.16	1.25	[0.78 , 1.73]	Inferior by ≥20%	<0.001
[CoP] Corail Trilogy	477	1.61	0.71	[-0.29 , 1.71]	Non-inferiority not shown	0.164
[CoP] Exeter V40 Charnley and Elite Plus LPW	250	2.02	1.12	[0.05 , 2.19]	Non-inferiority not shown	0.040
[CoP] Exeter V40 Elite Plus Ogee	535	1.35	0.44	[-0.29 , 1.18]	Non-inferiority not shown	0.240
[CoP] Exeter V40 Exeter Contemporary Flanged	1,110	1.42	0.52	[0.00 , 1.03]	Non-inferiority not shown	0.048
[CoP] Exeter V40 Exeter Contemporary Hooded	410	3.14	2.23	[0.98 , 3.48]	Inferior by ≥100%	<0.001
[CoP] Exeter V40 Exeter Duration	500	2.11	1.21	[0.08 , 2.33]	Non-inferiority not shown	0.035
[CoP] Exeter V40 Trident	1,113	1.46	0.56	[0.07 , 1.05]	Non-inferiority not shown	0.026
[CoP] Exeter V40 Trilogy	1,159	1.58	0.67	[0.04 , 1.30]	Non-inferiority not shown	0.037
[CoP] Furlong HAC Stem CSF	4,459	2.07	1.16	[0.69 , 1.64]	Inferior by ≥20%	<0.001
[CoP] Furlong HAC Stem Furlong HAC CSF Plus	345	2.46	1.55	[0.69 , 2.41]	Inferior by ≥20%	<0.001
[CoP] Furlong HAC Stem Furlong Threaded	327	1.84	0.94	[-0.45 , 2.33]	Non-inferiority not shown	0.187
[CoP] MS-30 Original ME Muller Low Profile Cup	675	0.70	-0.21	[-0.77 , 0.35]	Non-inferiority not shown	0.460
[CoP] SL-Plus Cementless Stem Bicon-Plus	406	4.66	3.75	[2.06 , 5.45]	Inferior by ≥100%	<0.001
[CoP] SL-Plus Cementless Stem EP-Fit Plus	579	4.10	3.20	[1.87 , 4.53]	Inferior by ≥100%	<0.001
[CoP] Versys Cementless Stem Trilogy	303	1.36	0.46	[-0.77 , 1.69]	Non-inferiority not shown	0.465
[MoP] ABG II Monolithic Cementless Stem ABG II Cementless Cup	376	2.40	1.50	[0.06 , 2.94]	Non-inferiority not shown	0.041

For peer review only

[MoP] ABG II Monolithic Cementless Stem Trident	301	5.72	4.81	[2.60 , 7.02]	Inferior by ≥100%	<0.001
[MoP] Accolade Trident	2,764	3.50	2.59	[2.07 , 3.11]	Inferior by ≥100%	<0.001
[MoP] C-Stem AMT Cemented Stem Charnley and Elite Plus LPW	782	1.81	0.91	[0.21 , 1.61]	Inferior by ≥20%	0.011
[MoP] C-Stem AMT Cemented Stem Elite Plus Ogee	523	1.71	0.81	[-0.02 , 1.64]	Non-inferiority not shown	0.057
[MoP] C-Stem AMT Cemented Stem Pinnacle	298	2.08	1.18	[0.34 , 2.02]	Inferior by ≥20%	0.006
[MoP] C-Stem Cemented Stem Charnley Ogee	539	2.03	1.12	[0.12 , 2.12]	Non-inferiority not shown	0.028
[MoP] C-Stem Cemented Stem Charnley and Elite Plus LPW	1,298	1.94	1.04	[0.29 , 1.79]	Inferior by ≥20%	0.007
[MoP] C-Stem Cemented Stem Duraloc Cementless Cup	391	3.73	2.83	[1.18 , 4.47]	Inferior by ≥100%	0.001
[MoP] C-Stem Cemented Stem Elite Plus Cemented Cup	472	1.62	0.71	[-0.34 , 1.76]	Non-inferiority not shown	0.184
[MoP] C-Stem Cemented Stem Elite Plus Ogee	1,851	1.55	0.65	[0.10 , 1.19]	Non-inferiority not shown	0.020
[MoP] C-Stem Cemented Stem Opera	744	2.01	1.10	[0.24 , 1.96]	Inferior by ≥20%	0.012
[MoP] C-Stem Cemented Stem Pinnacle	258	1.94	1.04	[-0.30 , 2.38]	Non-inferiority not shown	0.130
[MoP] C-Stem Cemented Stem Trilogy	278	1.11	0.21	[-0.92 , 1.34]	Non-inferiority not shown	0.719
[MoP] C-Stem Cemented Stem Wroblewski Golf Ball	502	1.88	0.97	[0.00 , 1.94]	Non-inferiority not shown	0.050
[MoP] CCA Cemented Stem CCB Cup	328	1.69	0.78	[-0.20 , 1.77]	Non-inferiority not shown	0.119
[MoP] CLS Cementless Stem Allofit	350	3.77	2.87	[1.30 , 4.44]	Inferior by ≥100%	<0.001
[MoP] CMK Modular Cemented Stem CMK Cemented Cup	329	1.08	0.17	[-0.82 , 1.16]	Non-inferiority not shown	0.734
[MoP] CPCS Opera	324	2.86	1.95	[0.78 , 3.12]	Inferior by ≥20%	0.001
[MoP] CPS Plus Cenator Cemented Cup	250	0.80	-0.10	[-1.06 , 0.86]	Non-inferiority not shown	0.836
[MoP] CPS Plus Opera	421	3.49	2.58	[0.92 , 4.25]	Inferior by ≥100%	0.002
[MoP] CPT Elite Plus Ogee	1,177	2.37	1.47	[0.75 , 2.19]	Inferior by ≥20%	<0.001
[MoP] CPT Opera	276	3.01	2.11	[0.39 , 3.82]	Inferior by ≥20%	0.016
[MoP] CPT Trabecular Metal Modular Cementless Cup	270	3.00	2.09	[0.78 , 3.40]	Inferior by ≥20%	0.002
[MoP] CPT Trilogy	3,606	2.69	1.78	[1.31 , 2.26]	Inferior by ≥100%	<0.001
[MoP] CPT ZCA	3,619	2.69	1.78	[1.30 , 2.27]	Inferior by ≥100%	<0.001
[MoP] Centrament Chirulen	257	1.12	0.22	[-0.93 , 1.36]	Non-inferiority not shown	0.710
[MoP] Charnley Cemented Stem Charnley Cemented Cup	3,004	2.31	1.41	[0.84 , 1.97]	Inferior by ≥20%	<0.001
[MoP] Charnley Cemented Stem Charnley Ogee	5,956	2.49	1.58	[1.13 , 2.04]	Inferior by ≥100%	<0.001
[MoP] Charnley Cemented Stem Charnley and Elite Plus LPW	4,036	1.51	0.60	[0.15 , 1.05]	Non-inferiority not shown	0.009
[MoP] Charnley Cemented Stem Opera	733	2.08	1.18	[0.29 , 2.07]	Inferior by ≥20%	0.009
[MoP] Charnley Cemented Stem Wroblewski Golf Ball	659	1.87	0.96	[0.07 , 1.86]	Non-inferiority not shown	0.035
[MoP] Corail Duraloc Cementless Cup	2,661	3.39	2.48	[1.79 , 3.18]	Inferior by ≥100%	<0.001
[MoP] Corail Elite Plus Cemented Cup	467	1.82	0.91	[-0.06 , 1.88]	Non-inferiority not shown	0.065
[MoP] Corail Elite Plus Ogee	696	1.83	0.92	[0.17 , 1.68]	Non-inferiority not shown	0.016
[MoP] Corail Marathon	354	1.49	0.59	[0.03 , 1.15]	Non-inferiority not shown	0.038
[MoP] Corail Pinnacle	7,798	2.17	1.26	[0.90 , 1.63]	Inferior by ≥20%	<0.001
[MoP] Corail Trilogy	855	2.65	1.75	[0.86 , 2.63]	Inferior by ≥20%	<0.001
[MoP] Elite Plus Cemented Stem Charnley and Elite Plus LPW	281	2.00	1.10	[-0.41 , 2.60]	Non-inferiority not shown	0.154
[MoP] Elite Plus Cemented Stem Elite Plus Ogee	465	1.26	0.35	[-0.63 , 1.33]	Non-inferiority not shown	0.480
[MoP] Exeter V40 ABG II Cementless Cup	626	2.02	1.11	[0.05 , 2.18]	Non-inferiority not shown	0.040
[MoP] Exeter V40 Cenator Cemented Cup	1,274	2.26	1.36	[0.65 , 2.07]	Inferior by ≥20%	<0.001
[MoP] Exeter V40 Charnley Ogee	901	1.62	0.71	[-0.03 , 1.46]	Non-inferiority not shown	0.061
[MoP] Exeter V40 Charnley and Elite Plus LPW	1,082	1.81	0.91	[0.29 , 1.53]	Inferior by ≥20%	0.004
[MoP] Exeter V40 Duraloc Cementless Cup	754	2.24	1.33	[0.39 , 2.27]	Inferior by ≥20%	0.005
[MoP] Exeter V40 EP-Fit Plus	424	3.64	2.74	[1.09 , 4.38]	Inferior by ≥100%	0.001
[MoP] Exeter V40 Elite Plus Ogee	8,638	1.50	0.59	[0.22 , 0.96]	Inferior by ≥20%	0.002

For peer review only

[MoP] Exeter V40 Exceed	265	0.94	0.04	[-0.84 , 0.92]	Non-inferiority not shown	0.934
[MoP] Exeter V40 Exeter Contemporary Flanged	16,415	1.58	0.67	[0.33 , 1.01]	Inferior by ≥20%	<0.001
[MoP] Exeter V40 Exeter Contemporary Hooded	7,073	2.79	1.89	[1.47 , 2.30]	Inferior by ≥100%	<0.001
[MoP] Exeter V40 Exeter Duration	7,252	2.45	1.55	[1.13 , 1.97]	Inferior by ≥100%	<0.001
[MoP] Exeter V40 Opera	1,144	1.59	0.68	[0.06 , 1.31]	Non-inferiority not shown	0.032
[MoP] Exeter V40 Pinnacle	718	1.89	0.98	[0.35 , 1.61]	Inferior by ≥20%	0.002
[MoP] Exeter V40 Reflection Cementless	1,359	2.31	1.41	[0.66 , 2.15]	Inferior by ≥20%	<0.001
[MoP] Exeter V40 Trident	5,685	1.85	0.95	[0.57 , 1.33]	Inferior by ≥20%	<0.001
[MoP] Exeter V40 Trilogy	4,380	1.67	0.77	[0.34 , 1.19]	Inferior by ≥20%	<0.001
[MoP] Exeter V40 Ultima Cemented Cup	916	2.29	1.38	[0.47 , 2.29]	Inferior by ≥20%	0.003
[MoP] Furlong Cemented Stem JRI Cemented Cup	1,041	1.99	1.09	[0.31 , 1.86]	Inferior by ≥20%	0.006
[MoP] Furlong HAC Stem CSF	4,138	3.06	2.15	[1.63 , 2.67]	Inferior by ≥100%	<0.001
[MoP] Furlong HAC Stem Furlong HAC CSF Plus	731	3.34	2.44	[1.72 , 3.16]	Inferior by ≥100%	<0.001
[MoP] Furlong HAC Stem Furlong Threaded	348	3.27	2.37	[0.75 , 3.98]	Inferior by ≥20%	0.004
[MoP] MS-30 Original ME Muller Low Profile Cup	334	1.74	0.83	[-0.27 , 1.93]	Non-inferiority not shown	0.139
[MoP] Mem Original ME Muller Low Profile Cup	286	0.86	-0.05	[-1.06 , 0.97]	Non-inferiority not shown	0.926
[MoP] Muller Straight Stem Centerpulse Muller	316	3.15	2.24	[0.56 , 3.92]	Inferior by ≥20%	0.009
[MoP] Muller Straight Stem Original ME Muller Low Profile Cup	646	2.06	1.16	[0.27 , 2.04]	Inferior by ≥20%	0.010
[MoP] Muller-Biomet Apollo	1,089	1.70	0.80	[0.13 , 1.46]	Non-inferiority not shown	0.019
[MoP] Muller-Biomet Original ME Muller Low Profile Cup	267	2.22	1.32	[0.21 , 2.42]	Inferior by ≥20%	0.019
[MoP] Omnifit Cemented Stem ODC	708	2.78	1.88	[0.73 , 3.03]	Inferior by ≥20%	0.001
[MoP] Omnifit Cementless Stem Secure Fit Cementless Cup	255	1.42	0.52	[-0.90 , 1.93]	Non-inferiority not shown	0.476
[MoP] Omnifit Cementless Stem Trident	321	6.51	5.61	[3.28 , 7.94]	Inferior by ≥100%	<0.001
[MoP] SL-Plus Cementless Stem EP-Fit Plus	1,230	4.42	3.52	[2.58 , 4.46]	Inferior by ≥100%	<0.001
[MoP] SP II Cemented Stem Interplanta	455	4.00	3.09	[1.54 , 4.65]	Inferior by ≥100%	<0.001
[MoP] Stanmore Modular Stem SHP Cup	480	1.46	0.55	[-0.33 , 1.44]	Non-inferiority not shown	0.222
[MoP] Stanmore Modular Stem Stanmore-Arcom Cup	1,908	1.97	1.06	[0.51 , 1.61]	Inferior by ≥20%	<0.001
[MoP] Synergy Cementless Stem Reflection Cementless	1,002	1.75	0.85	[0.10 , 1.60]	Non-inferiority not shown	0.027
[MoP] Taperloc Cementless Stem Exceed	370	2.27	1.37	[0.04 , 2.69]	Non-inferiority not shown	0.043
[MoP] Taperloc Cementless Stem Exceed ABT	513	2.52	1.61	[0.96 , 2.26]	Inferior by ≥100%	<0.001
[MoP] Versys Cementless Stem Trilogy	714	4.81	3.91	[2.48 , 5.33]	Inferior by ≥100%	<0.001

For peer review only

Supplementary table 1d: Difference in Kaplan-Meier failure estimate between a contemporary reference and implants with at least 250 at risk at 10 years since primary

Stem/cup brand	Number at risk	Cumulative failure (%)	Difference in failure (%)	95% CI	Equivalence status	p-value
[MoP] Exeter V40 Elite Plus Ogee	3,580	2.14	[REFERENCE]			
[CoC] ABG II Monolithic Cementless Stem ABG II Cementless Cup	695	4.42	2.28	[0.96 , 3.60]	Inferior by ≥20%	0.001
[CoC] ABG II Monolithic Cementless Stem Trident	412	4.15	2.01	[0.68 , 3.34]	Inferior by ≥20%	0.003
[CoC] Accolade Trident	454	4.38	2.24	[1.48 , 3.00]	Inferior by ≥20%	<0.001
[CoC] Corail Duraloc Option	383	4.45	2.31	[0.93 , 3.68]	Inferior by ≥20%	0.001
[CoC] Corail Pinnacle	886	3.90	1.76	[1.23 , 2.29]	Inferior by ≥20%	<0.001
[CoC] Exeter V40 ABG II Cementless Cup	440	2.66	0.52	[-0.54 , 1.58]	Non-inferiority not shown	0.334
[CoC] Exeter V40 Trident	1,846	2.59	0.44	[-0.04 , 0.93]	Non-inferiority not shown	0.072
[CoC] Furlong HAC Stem CSF	829	4.37	2.22	[1.13 , 3.32]	Inferior by ≥20%	<0.001
[CoC] SL-Plus Cementless Stem EP-Fit Plus	288	6.94	4.80	[3.25 , 6.35]	Inferior by ≥100%	<0.001
[CoP] C-Stem Cemented Stem Wroblewski Golf Ball	317	2.51	0.36	[-0.88 , 1.61]	Non-inferiority not shown	0.567
[CoP] Exeter V40 Exeter Contemporary Flanged	269	2.10	-0.05	[-0.90 , 0.80]	Non-inferiority not shown	0.913
[CoP] Exeter V40 Trilogy	494	1.91	-0.23	[-0.96 , 0.50]	Non-inferiority not shown	0.537
[CoP] Furlong HAC Stem CSF	2,520	2.65	0.51	[-0.02 , 1.03]	Non-inferiority not shown	0.058
[MoP] ABG II Monolithic Cementless Stem ABG II Cementless Cup	278	4.37	2.22	[0.21 , 4.24]	Non-inferiority not shown	0.031
[MoP] C-Stem Cemented Stem Charnley Ogee	293	2.25	0.11	[-0.97 , 1.19]	Non-inferiority not shown	0.846
[MoP] C-Stem Cemented Stem Charnley and Elite Plus LPW	725	2.45	0.30	[-0.54 , 1.14]	Non-inferiority not shown	0.481
[MoP] C-Stem Cemented Stem Elite Plus Cemented Cup	267	2.49	0.35	[-1.08 , 1.79]	Non-inferiority not shown	0.632
[MoP] C-Stem Cemented Stem Elite Plus Ogee	882	2.57	0.43	[-0.33 , 1.19]	Non-inferiority not shown	0.271
[MoP] C-Stem Cemented Stem Opera	356	3.79	1.64	[0.21 , 3.08]	Non-inferiority not shown	0.025
[MoP] C-Stem Cemented Stem Wroblewski Golf Ball	259	2.47	0.33	[-0.94 , 1.59]	Non-inferiority not shown	0.614
[MoP] CPT Elite Plus Ogee	408	3.16	1.01	[0.04 , 1.98]	Non-inferiority not shown	0.041
[MoP] CPT Trilogy	1,135	4.29	2.14	[1.42 , 2.86]	Inferior by ≥20%	<0.001
[MoP] CPT ZCA	1,233	3.71	1.57	[0.95 , 2.19]	Inferior by ≥20%	<0.001
[MoP] Charnley Cemented Stem Charnley Cemented Cup	1,751	3.52	1.38	[0.69 , 2.07]	Inferior by ≥20%	<0.001
[MoP] Charnley Cemented Stem Charnley Ogee	3,102	3.93	1.78	[1.23 , 2.34]	Inferior by ≥20%	<0.001
[MoP] Charnley Cemented Stem Charnley and Elite Plus LPW	2,284	2.43	0.29	[-0.25 , 0.83]	Non-inferiority not shown	0.297
[MoP] Charnley Cemented Stem Opera	254	3.99	1.85	[0.19 , 3.51]	Non-inferiority not shown	0.029
[MoP] Charnley Cemented Stem Wroblewski Golf Ball	419	2.42	0.28	[-0.81 , 1.36]	Non-inferiority not shown	0.619
[MoP] Corail Duraloc Cementless Cup	1,200	5.37	3.22	[2.31 , 4.14]	Inferior by ≥100%	<0.001
[MoP] Corail Pinnacle	1,681	3.05	0.91	[0.45 , 1.36]	Inferior by ≥20%	<0.001
[MoP] Elite Plus Cemented Stem Elite Plus Ogee	351	2.03	-0.11	[-1.41 , 1.19]	Non-inferiority not shown	0.865
[MoP] Exeter V40 ABG II Cementless Cup	429	2.62	0.48	[-0.77 , 1.73]	Non-inferiority not shown	0.456
[MoP] Exeter V40 Cenator Cemented Cup	694	2.64	0.49	[-0.29 , 1.28]	Non-inferiority not shown	0.219
[MoP] Exeter V40 Charnley Ogee	561	1.62	-0.52	[-1.26 , 0.21]	Non-inferior	0.163
[MoP] Exeter V40 Charnley and Elite Plus LPW	404	2.44	0.30	[-0.53 , 1.12]	Non-inferiority not shown	0.484
[MoP] Exeter V40 Duraloc Cementless Cup	451	4.19	2.05	[0.62 , 3.48]	Inferior by ≥20%	0.005
[MoP] Exeter V40 Elite Plus Cemented Cup	725	1.44	-0.70	[-1.31 , -0.09]	Non-inferior	0.024
[MoP] Exeter V40 Exeter Contemporary Flanged	4,653	2.28	0.13	[-0.23 , 0.49]	Non-inferiority not shown	0.469

For peer review only

[MoP] Exeter V40 Exeter Contemporary Hooded	2,355	4.12	1.97	[1.45 , 2.49]	Inferior by ≥20%	<0.001
[MoP] Exeter V40 Exeter Duration	2,967	3.71	1.56	[1.05 , 2.07]	Inferior by ≥20%	<0.001
[MoP] Exeter V40 Opera	443	3.23	1.09	[-0.03 , 2.21]	Non-inferiority not shown	0.057
[MoP] Exeter V40 Reflection Cementless	393	4.18	2.03	[0.76 , 3.30]	Inferior by ≥20%	0.002
[MoP] Exeter V40 Trident	1,116	2.71	0.56	[0.05 , 1.08]	Non-inferiority not shown	0.031
[MoP] Exeter V40 Trilogy	1,538	2.49	0.35	[-0.17 , 0.87]	Non-inferiority not shown	0.191
[MoP] Exeter V40 Ultima Cemented Cup	498	3.27	1.12	[-0.03 , 2.27]	Non-inferiority not shown	0.056
[MoP] Furlong Cemented Stem JRI Cemented Cup	584	3.23	1.09	[0.01 , 2.17]	Non-inferiority not shown	0.048
[MoP] Furlong HAC Stem CSF	1,697	4.41	2.27	[1.61 , 2.92]	Inferior by ≥20%	<0.001
[MoP] Muller-Biomet Apollo	371	2.67	0.53	[-0.56 , 1.62]	Non-inferiority not shown	0.345
[MoP] Omnifit Cemented Stem ODC	458	3.89	1.74	[0.35 , 3.14]	Non-inferiority not shown	0.014
[MoP] SP II Cemented Stem Interplanta	258	5.45	3.31	[1.32 , 5.30]	Inferior by ≥20%	0.001
[MoP] Stanmore Modular Stem Stanmore-Arcom Cup	669	2.52	0.38	[-0.29 , 1.04]	Non-inferiority not shown	0.264
[MoP] Synergy Cementless Stem Reflection Cementless	322	2.38	0.24	[-0.80 , 1.28]	Non-inferiority not shown	0.651
[MoP] Versys Cementless Stem Trilogy	464	5.47	3.32	[1.77 , 4.87]	Inferior by ≥20%	<0.001

For peer review only

Supplemental table 2a: Difference in Kaplan-Meier failure estimate between a contemporary reference and implants with at least 250 at risk at 3 years since primary in females

Stem/cup brand	Number at risk	Cumulative failure (%)	Difference in failure (%)	95% CI	Equivalence status	p-value
[CoP] MS-30 Original ME Muller Low Profile Cup	1,096	0.41	[REFERENCE]			
[CoC] ABG II Monolithic Cementless Stem ABG II Cementless Cup	502	1.36	0.96	[-0.11 , 2.02]	Non-inferiority not shown	0.079
[CoC] ABG II Monolithic Cementless Stem Trident	465	2.24	1.84	[0.48 , 3.19]	Inferior by ≥100%	0.008
[CoC] Accolade Trident	3,662	1.78	1.37	[0.83 , 1.92]	Inferior by ≥100%	<0.001
[CoC] Bimetric Cementless Stem Exceed ABT	511	1.18	0.77	[-0.17 , 1.70]	Non-inferiority not shown	0.108
[CoC] C-Stem AMT Cemented Stem Pinnacle	487	1.06	0.65	[-0.22 , 1.52]	Non-inferiority not shown	0.143
[CoC] CPT Continuum	481	1.17	0.76	[-0.13 , 1.64]	Non-inferiority not shown	0.093
[CoC] CPT Trilogy AB	305	0.97	0.56	[-0.59 , 1.71]	Non-inferiority not shown	0.339
[CoC] Corail Delta TT	453	1.90	1.49	[0.44 , 2.54]	Inferior by ≥100%	0.005
[CoC] Corail DeltaMotion	696	1.22	0.81	[-0.02 , 1.65]	Non-inferiority not shown	0.056
[CoC] Corail Duraloc Option	580	1.68	1.27	[0.18 , 2.36]	Inferior by ≥20%	0.023
[CoC] Corail Pinnacle	14,431	1.67	1.26	[0.85 , 1.66]	Inferior by ≥100%	<0.001
[CoC] Corail Pinnacle Gription	292	2.28	1.87	[0.57 , 3.17]	Inferior by ≥100%	0.005
[CoC] Corail Trinity	256	0.21	-0.20	[-0.74 , 0.35]	Non-inferiority not shown	0.474
[CoC] Excia Cementless Plasmacup SC	471	1.52	1.11	[0.06 , 2.17]	Non-inferiority not shown	0.038
[CoC] Exeter V40 ABG II Cementless Cup	887	0.40	-0.01	[-0.54 , 0.52]	Non-inferiority not shown	0.964
[CoC] Exeter V40 Trident	5,950	0.91	0.51	[0.08 , 0.93]	Non-inferiority not shown	0.020
[CoC] Furlong Evolution Cementless Furlong HAC CSF Plus	323	0.90	0.49	[-0.17 , 1.16]	Non-inferiority not shown	0.147
[CoC] Furlong HAC Stem CSF	881	1.54	1.13	[0.26 , 2.01]	Inferior by ≥20%	0.011
[CoC] Furlong HAC Stem Furlong HAC CSF Plus	4,954	1.49	1.08	[0.62 , 1.54]	Inferior by ≥100%	<0.001
[CoC] M/L Taper Cementless Continuum	689	1.52	1.11	[0.24 , 1.98]	Inferior by ≥20%	0.012
[CoC] Metafix Stem Trinity	469	1.43	1.03	[0.13 , 1.93]	Inferior by ≥20%	0.025
[CoC] Omnifit Cementless Stem Trident	267	2.20	1.79	[0.01 , 3.57]	Non-inferiority not shown	0.048
[CoC] Polarstem Cementless R3 Cementless	460	1.10	0.70	[-0.19 , 1.59]	Non-inferiority not shown	0.125
[CoC] S-Rom Cementless Stem Pinnacle	327	2.46	2.06	[0.50 , 3.61]	Inferior by ≥100%	0.010
[CoC] SL-Plus Cementless Stem EP-Fit Plus	602	1.75	1.34	[0.26 , 2.43]	Inferior by ≥20%	0.015
[CoC] SPS Modular April - Ceramic	302	2.39	1.98	[0.31 , 3.66]	Inferior by ≥20%	0.020
[CoC] Summit Cementless Stem Pinnacle	269	1.14	0.73	[-0.44 , 1.90]	Non-inferiority not shown	0.223
[CoC] Taperloc Cementless Stem Exceed ABT	3,728	1.43	1.02	[0.54 , 1.50]	Inferior by ≥100%	<0.001
[CoC] miniHip Trinity	441	1.11	0.70	[-0.19 , 1.60]	Non-inferiority not shown	0.125
[CoP] Accolade Trident	1,760	1.39	0.98	[0.40 , 1.56]	Inferior by ≥20%	0.001
[CoP] C-Stem Cemented Stem Elite Plus Ogee	342	0.28	-0.12	[-0.79 , 0.54]	Non-inferiority not shown	0.716
[CoP] C-Stem Cemented Stem Marathon	626	1.01	0.60	[-0.13 , 1.33]	Non-inferiority not shown	0.108
[CoP] C-Stem Cemented Stem Opera	445	0.22	-0.19	[-0.75 , 0.38]	Non-inferiority not shown	0.515
[CoP] C-Stem Cemented Stem Wroblewski Golf Ball	517	0.53	0.12	[-0.57 , 0.82]	Non-inferiority not shown	0.732
[CoP] CPT Trilogy	871	1.24	0.84	[0.27 , 1.40]	Inferior by ≥20%	0.004
[CoP] Corail Charnley and Elite Plus LPW	376	2.14	1.73	[0.30 , 3.16]	Inferior by ≥20%	0.018
[CoP] Corail Elite Plus Cemented Cup	267	0.91	0.50	[-0.58 , 1.58]	Non-inferiority not shown	0.367
[CoP] Corail Elite Plus Ogee	268	1.67	1.26	[-0.23 , 2.76]	Non-inferiority not shown	0.098
[CoP] Corail Marathon	787	0.72	0.31	[-0.26 , 0.89]	Non-inferiority not shown	0.285

For peer review only

[CoP] Corail Pinnacle	4,637	1.27	0.86	[0.43 , 1.29]	Inferior by ≥100%	<0.001
[CoP] Corail Trilogy	437	0.87	0.46	[-0.46 , 1.39]	Non-inferiority not shown	0.324
[CoP] Exeter V40 Charnley and Elite Plus LPW	374	1.99	1.58	[0.30 , 2.86]	Inferior by ≥20%	0.015
[CoP] Exeter V40 Elite Plus Ogee	655	0.57	0.16	[-0.45 , 0.77]	Non-inferiority not shown	0.611
[CoP] Exeter V40 Exeter Contemporary Flanged	1,849	1.14	0.73	[0.18 , 1.28]	Inferior by ≥20%	0.009
[CoP] Exeter V40 Exeter Contemporary Hooded	482	1.83	1.42	[0.29 , 2.56]	Inferior by ≥20%	0.014
[CoP] Exeter V40 Exeter Duration	476	0.93	0.52	[-0.37 , 1.40]	Non-inferiority not shown	0.251
[CoP] Exeter V40 Exeter X3 Rimfit	1,264	0.76	0.35	[-0.14 , 0.84]	Non-inferiority not shown	0.164
[CoP] Exeter V40 Marathon	284	0.92	0.51	[-0.48 , 1.49]	Non-inferiority not shown	0.312
[CoP] Exeter V40 Pinnacle	292	1.12	0.71	[-0.20 , 1.62]	Non-inferiority not shown	0.127
[CoP] Exeter V40 Trident	2,940	0.93	0.52	[0.08 , 0.96]	Inferior by ≥20%	0.020
[CoP] Exeter V40 Trilogy	1,182	0.60	0.19	[-0.36 , 0.74]	Non-inferiority not shown	0.491
[CoP] Exeter V40 Tritanium	250	1.74	1.33	[0.12 , 2.55]	Inferior by ≥20%	0.032
[CoP] Furlong HAC Stem CSF	3,748	1.32	0.91	[0.41 , 1.41]	Inferior by ≥100%	<0.001
[CoP] Furlong HAC Stem Furlong HAC CSF Plus	840	2.36	1.95	[0.99 , 2.91]	Inferior by ≥100%	<0.001
[CoP] SL-Plus Cementless Stem Bicon-Plus	342	1.66	1.25	[-0.11 , 2.61]	Non-inferiority not shown	0.073
[CoP] SL-Plus Cementless Stem EP-Fit Plus	593	1.93	1.52	[0.38 , 2.66]	Inferior by ≥20%	0.009
[CoP] Stanmore Modular Stem Stanmore-Arcom Cup	262	1.58	1.17	[-0.25 , 2.59]	Non-inferiority not shown	0.106
[CoP] Taperloc Cementless Stem Exceed ABT	1,062	0.99	0.59	[0.01 , 1.16]	Non-inferiority not shown	0.046
[MoP] Accolade Trident	5,487	1.75	1.34	[0.86 , 1.82]	Inferior by ≥100%	<0.001
[MoP] Anthology R3 Cementless	729	1.46	1.05	[0.29 , 1.81]	Inferior by ≥20%	0.007
[MoP] C-Stem AMT Cemented Stem Charnley and Elite Plus LPW	1,437	0.91	0.50	[-0.07 , 1.07]	Non-inferiority not shown	0.085
[MoP] C-Stem AMT Cemented Stem Elite Plus Cemented Cup	438	0.61	0.20	[-0.57 , 0.97]	Non-inferiority not shown	0.614
[MoP] C-Stem AMT Cemented Stem Elite Plus Ogee	1,135	0.84	0.43	[-0.15 , 1.01]	Non-inferiority not shown	0.149
[MoP] C-Stem AMT Cemented Stem Marathon	751	0.88	0.47	[-0.10 , 1.04]	Non-inferiority not shown	0.108
[MoP] C-Stem AMT Cemented Stem Pinnacle	985	1.24	0.84	[0.25 , 1.42]	Inferior by ≥20%	0.005
[MoP] C-Stem Cemented Stem Charnley Ogee	597	1.48	1.07	[0.09 , 2.05]	Inferior by ≥20%	0.033
[MoP] C-Stem Cemented Stem Charnley and Elite Plus LPW	1,119	1.17	0.76	[0.05 , 1.46]	Non-inferiority not shown	0.035
[MoP] C-Stem Cemented Stem Duraloc Cementless Cup	420	1.59	1.18	[-0.04 , 2.41]	Non-inferiority not shown	0.058
[MoP] C-Stem Cemented Stem Elite Plus Cemented Cup	390	0.47	0.06	[-0.68 , 0.80]	Non-inferiority not shown	0.870
[MoP] C-Stem Cemented Stem Elite Plus Ogee	1,985	0.69	0.28	[-0.21 , 0.78]	Non-inferiority not shown	0.257
[MoP] C-Stem Cemented Stem Marathon	959	0.63	0.22	[-0.30 , 0.73]	Non-inferiority not shown	0.407
[MoP] C-Stem Cemented Stem Opera	836	0.68	0.27	[-0.38 , 0.91]	Non-inferiority not shown	0.417
[MoP] C-Stem Cemented Stem Pinnacle	301	1.59	1.19	[-0.13 , 2.50]	Non-inferiority not shown	0.077
[MoP] C-Stem Cemented Stem Wroblewski Golf Ball	625	0.74	0.33	[-0.41 , 1.07]	Non-inferiority not shown	0.379
[MoP] CCA Cemented Stem CCB Cup	793	0.62	0.21	[-0.40 , 0.82]	Non-inferiority not shown	0.501
[MoP] CLS Cementless Stem Allofit	336	1.93	1.52	[0.06 , 2.99]	Non-inferiority not shown	0.041
[MoP] CLS Cementless Stem Trilogy	321	3.16	2.75	[0.88 , 4.62]	Inferior by ≥100%	0.004
[MoP] CMK Modular Cemented Stem CMK Cemented Cup	319	0.30	-0.11	[-0.79 , 0.58]	Non-inferiority not shown	0.755
[MoP] CPCS Opera	804	0.95	0.55	[-0.20 , 1.30]	Non-inferiority not shown	0.153
[MoP] CPCS Polarcup Cementless	259	0.76	0.35	[-0.76 , 1.46]	Non-inferiority not shown	0.535
[MoP] CPCS Reflection Cemented	264	1.10	0.69	[-0.26 , 1.64]	Non-inferiority not shown	0.156
[MoP] CPS Plus Cenator Cemented Cup	268	0.37	-0.04	[-0.85 , 0.77]	Non-inferiority not shown	0.931
[MoP] CPS Plus Opera	327	1.17	0.76	[-0.43 , 1.96]	Non-inferiority not shown	0.211
[MoP] CPT Allofit	485	0.32	-0.09	[-0.66 , 0.48]	Non-inferiority not shown	0.752
[MoP] CPT Continuum	315	2.66	2.25	[1.17 , 3.33]	Inferior by ≥100%	<0.001

For peer review only

[MoP] CPT Elite Plus Ogee	1,550	1.24	0.83	[0.21 , 1.46]	Inferior by ≥20%	0.009
[MoP] CPT Exeter Contemporary Flanged	283	2.17	1.76	[0.31 , 3.21]	Inferior by ≥20%	0.017
[MoP] CPT Opera	268	1.06	0.66	[-0.59 , 1.91]	Non-inferiority not shown	0.303
[MoP] CPT Original ME Muller Low Profile Cup	600	1.30	0.89	[0.00 , 1.78]	Non-inferiority not shown	0.050
[MoP] CPT Pinnacle	511	1.59	1.19	[0.09 , 2.28]	Inferior by ≥20%	0.033
[MoP] CPT Trabecular Metal Modular Cementless Cup	645	1.72	1.32	[0.40 , 2.23]	Inferior by ≥20%	0.005
[MoP] CPT Trilogy	5,622	1.20	0.79	[0.35 , 1.22]	Inferior by ≥20%	<0.001
[MoP] CPT Trilogy IT	304	2.93	2.52	[1.48 , 3.57]	Inferior by ≥100%	<0.001
[MoP] CPT ZCA	5,377	1.34	0.93	[0.49 , 1.38]	Inferior by ≥100%	<0.001
[MoP] Centrament Chirulen	294	0.00	- -	[-.-- , -.--]	No failures to date	
[MoP] Charnley Cemented Stem Charnley Cemented Cup	2,552	0.97	0.56	[0.04 , 1.07]	Non-inferiority not shown	0.033
[MoP] Charnley Cemented Stem Charnley Ogee	5,409	1.08	0.67	[0.22 , 1.11]	Inferior by ≥20%	0.003
[MoP] Charnley Cemented Stem Charnley and Elite Plus LPW	4,125	0.61	0.20	[-0.22 , 0.63]	Non-inferiority not shown	0.352
[MoP] Charnley Cemented Stem Opera	823	0.70	0.29	[-0.37 , 0.96]	Non-inferiority not shown	0.385
[MoP] Charnley Cemented Stem Wroblewski Golf Ball	609	1.53	1.12	[0.11 , 2.12]	Inferior by ≥20%	0.029
[MoP] Charnley Modular Charnley and Elite Plus LPW	275	0.36	-0.05	[-0.84 , 0.75]	Non-inferiority not shown	0.908
[MoP] Corail Charnley and Elite Plus LPW	543	0.83	0.42	[-0.33 , 1.17]	Non-inferiority not shown	0.274
[MoP] Corail Duraloc Cementless Cup	2,139	1.30	0.89	[0.30 , 1.48]	Inferior by ≥20%	0.003
[MoP] Corail Elite Plus Cemented Cup	629	0.42	0.02	[-0.58 , 0.62]	Non-inferiority not shown	0.955
[MoP] Corail Elite Plus Ogee	937	1.08	0.67	[-0.01 , 1.36]	Non-inferiority not shown	0.055
[MoP] Corail Exeter Contemporary Flanged	376	0.95	0.54	[-0.37 , 1.45]	Non-inferiority not shown	0.246
[MoP] Corail Marathon	1,873	0.80	0.39	[-0.08 , 0.87]	Non-inferiority not shown	0.107
[MoP] Corail Pinnacle	17,132	1.26	0.85	[0.47 , 1.23]	Inferior by ≥100%	<0.001
[MoP] Corail Trident	385	1.01	0.60	[-0.23 , 1.43]	Non-inferiority not shown	0.154
[MoP] Corail Trilogy	1,066	0.90	0.50	[-0.15 , 1.14]	Non-inferiority not shown	0.129
[MoP] Elite Plus Cemented Stem Elite Plus Ogee	402	0.49	0.08	[-0.68 , 0.84]	Non-inferiority not shown	0.842
[MoP] Exeter V40 ABG II Cementless Cup	477	0.80	0.39	[-0.47 , 1.25]	Non-inferiority not shown	0.373
[MoP] Exeter V40 Cenator Cemented Cup	1,425	1.45	1.04	[0.35 , 1.73]	Inferior by ≥20%	0.003
[MoP] Exeter V40 Charnley Ogee	1,068	1.00	0.59	[-0.08 , 1.26]	Non-inferiority not shown	0.083
[MoP] Exeter V40 Charnley and Elite Plus LPW	1,811	1.31	0.90	[0.31 , 1.50]	Inferior by ≥20%	0.003
[MoP] Exeter V40 Duraloc Cementless Cup	708	1.10	0.69	[-0.15 , 1.53]	Non-inferiority not shown	0.106
[MoP] Exeter V40 EP-Fit Plus	333	1.17	0.77	[-0.43 , 1.96]	Non-inferiority not shown	0.210
[MoP] Exeter V40 Elite Plus Cemented Cup	2,389	0.45	0.04	[-0.39 , 0.48]	Non-inferiority not shown	0.842
[MoP] Exeter V40 Elite Plus Ogee	10,898	0.70	0.29	[-0.10 , 0.67]	Non-inferiority not shown	0.142
[MoP] Exeter V40 Exceed	333	0.85	0.44	[-0.58 , 1.46]	Non-inferiority not shown	0.397
[MoP] Exeter V40 Exceed ABT	284	0.94	0.53	[-0.45 , 1.52]	Non-inferiority not shown	0.290
[MoP] Exeter V40 Exeter Contemporary Flanged	27,147	0.71	0.30	[-0.07 , 0.67]	Non-inferiority not shown	0.113
[MoP] Exeter V40 Exeter Contemporary Hooded	10,906	1.39	0.98	[0.58 , 1.39]	Inferior by ≥100%	<0.001
[MoP] Exeter V40 Exeter Duration	8,897	1.05	0.64	[0.23 , 1.05]	Inferior by ≥20%	0.002
[MoP] Exeter V40 Exeter X3 Rimfit	3,408	0.73	0.32	[-0.09 , 0.73]	Non-inferiority not shown	0.123
[MoP] Exeter V40 Furlong HAC CSF Plus	340	0.17	-0.24	[-0.73 , 0.25]	Non-inferiority not shown	0.333
[MoP] Exeter V40 Marathon	884	0.88	0.47	[-0.12 , 1.06]	Non-inferiority not shown	0.119
[MoP] Exeter V40 Opera	1,660	0.72	0.31	[-0.22 , 0.84]	Non-inferiority not shown	0.248
[MoP] Exeter V40 Pinnacle	1,807	0.97	0.56	[0.05 , 1.07]	Non-inferiority not shown	0.033
[MoP] Exeter V40 R3 Cementless	363	0.90	0.49	[-0.27 , 1.26]	Non-inferiority not shown	0.205
[MoP] Exeter V40 Reflection Cementless	1,308	0.73	0.32	[-0.25 , 0.90]	Non-inferiority not shown	0.273

For peer review only

[MoP] Exeter V40 Trabecular Metal Modular Cementless Cup	286	1.55	1.14	[-0.14 , 2.42]	Non-inferiority not shown	0.081
[MoP] Exeter V40 Trident	11,298	0.97	0.57	[0.18 , 0.95]	Inferior by ≥20%	0.004
[MoP] Exeter V40 Trilogy	5,145	0.83	0.43	[0.00 , 0.85]	Non-inferiority not shown	0.050
[MoP] Exeter V40 Tritanium	282	1.60	1.19	[0.12 , 2.25]	Inferior by ≥20%	0.029
[MoP] Exeter V40 Ultima Cemented Cup	777	1.10	0.69	[-0.11 , 1.49]	Non-inferiority not shown	0.090
[MoP] Furlong Cemented Stem JRI Cemented Cup	1,036	1.43	1.02	[0.24 , 1.81]	Inferior by ≥20%	0.010
[MoP] Furlong HAC Stem CSF	4,074	2.18	1.77	[1.22 , 2.33]	Inferior by ≥100%	<0.001
[MoP] Furlong HAC Stem Furlong HAC CSF Plus	1,829	2.12	1.71	[1.07 , 2.36]	Inferior by ≥100%	<0.001
[MoP] Furlong HAC Stem Furlong Threaded	311	1.83	1.42	[-0.07 , 2.92]	Non-inferiority not shown	0.062
[MoP] M/L Taper Cementless Continuum	381	1.72	1.31	[0.24 , 2.37]	Inferior by ≥20%	0.016
[MoP] M/L Taper Cementless Trilogy	281	2.08	1.68	[0.34 , 3.01]	Inferior by ≥20%	0.014
[MoP] MS-30 Original ME Muller Low Profile Cup	522	0.28	-0.13	[-0.66 , 0.40]	Non-inferiority not shown	0.637
[MoP] Muller Straight Stem Centerpulse Muller	328	0.88	0.47	[-0.58 , 1.52]	Non-inferiority not shown	0.382
[MoP] Muller Straight Stem Original ME Muller Low Profile Cup	999	0.92	0.52	[-0.12 , 1.15]	Non-inferiority not shown	0.112
[MoP] Muller-Biomet Apollo	1,201	1.32	0.91	[0.19 , 1.63]	Inferior by ≥20%	0.013
[MoP] Muller-Biomet Original ME Muller Low Profile Cup	707	1.32	0.91	[0.02 , 1.80]	Non-inferiority not shown	0.044
[MoP] Omnifit Cemented Stem ODC	614	1.56	1.15	[0.13 , 2.18]	Inferior by ≥20%	0.027
[MoP] Omnifit Cementless Stem Trident	251	2.95	2.55	[0.50 , 4.60]	Inferior by ≥100%	0.015
[MoP] P10 Muller Original ME Muller Low Profile Cup	268	1.03	0.62	[-0.59 , 1.83]	Non-inferiority not shown	0.317
[MoP] Polarstem Cementless R3 Cementless	592	0.73	0.33	[-0.19 , 0.85]	Non-inferiority not shown	0.217
[MoP] SL-Plus Cementless Stem EP-Fit Plus	1,198	2.40	2.00	[1.14 , 2.85]	Inferior by ≥100%	<0.001
[MoP] SP II Cemented Stem Interplanta	444	1.84	1.43	[0.19 , 2.68]	Inferior by ≥20%	0.024
[MoP] Stanmore Modular Stem Elite Plus Cemented Cup	367	0.26	-0.15	[-0.77 , 0.48]	Non-inferiority not shown	0.647
[MoP] Stanmore Modular Stem SHP Cup	664	0.75	0.34	[-0.36 , 1.04]	Non-inferiority not shown	0.339
[MoP] Stanmore Modular Stem Stanmore-Arcom Cup	2,729	1.12	0.71	[0.19 , 1.23]	Inferior by ≥20%	0.007
[MoP] Synergy Cementless Stem R3 Cementless	554	1.00	0.59	[-0.08 , 1.26]	Non-inferiority not shown	0.082
[MoP] Synergy Cementless Stem Reflection Cementless	859	0.68	0.27	[-0.38 , 0.92]	Non-inferiority not shown	0.416
[MoP] Taperfit Cemented Stem Atlas IIIp	327	1.14	0.73	[-0.33 , 1.79]	Non-inferiority not shown	0.177
[MoP] Taperloc Cementless Stem Exceed	318	1.20	0.79	[-0.43 , 2.01]	Non-inferiority not shown	0.204
[MoP] Taperloc Cementless Stem Exceed ABT	2,287	1.98	1.57	[0.98 , 2.16]	Inferior by ≥100%	<0.001
[MoP] Versys Cementless Stem Trilogy	492	3.79	3.38	[1.71 , 5.05]	Inferior by ≥100%	<0.001

For peer review only

Supplemental table 2b: Difference in Kaplan-Meier failure estimate between a contemporary reference and implants with at least 250 at risk at 5 years since primary in females

Stem/cup brand	Number at risk	Cumulative failure (%)	Difference in failure (%)	95% CI	Equivalence status	p-value
[MoP] Exeter V40 Elite Plus Cemented Cup	1,769	0.54	[REFERENCE]			
[CoC] ABG II Monolithic Cementless Stem ABG II Cementless Cup	485	1.76	1.22	[0.05 , 2.40]	Non-inferiority not shown	0.041
[CoC] ABG II Monolithic Cementless Stem Trident	372	2.95	2.41	[0.86 , 3.96]	Inferior by ≥100%	0.002
[CoC] Accolade Trident	2,906	2.56	2.02	[1.44 , 2.59]	Inferior by ≥100%	<0.001
[CoC] Bimetric Cementless Stem Exceed ABT	369	1.84	1.30	[0.13 , 2.48]	Inferior by ≥20%	0.030
[CoC] C-Stem AMT Cemented Stem Pinnacle	268	1.80	1.26	[0.08 , 2.44]	Non-inferiority not shown	0.037
[CoC] CPT Trilogy AB	278	1.64	1.10	[-0.35 , 2.55]	Non-inferiority not shown	0.138
[CoC] Corail DeltaMotion	373	1.47	0.93	[-0.01 , 1.86]	Non-inferiority not shown	0.051
[CoC] Corail Duraloc Option	566	2.70	2.16	[0.83 , 3.49]	Inferior by ≥100%	0.001
[CoC] Corail Pinnacle	9,650	2.16	1.62	[1.26 , 1.97]	Inferior by ≥100%	<0.001
[CoC] Excia Cementless Plasmacup SC	266	1.52	0.98	[-0.04 , 2.01]	Non-inferiority not shown	0.061
[CoC] Exeter V40 ABG II Cementless Cup	760	1.10	0.56	[-0.17 , 1.30]	Non-inferiority not shown	0.133
[CoC] Exeter V40 Trident	4,852	1.27	0.74	[0.35 , 1.13]	Inferior by ≥20%	<0.001
[CoC] Furlong HAC Stem CSF	839	2.11	1.57	[0.59 , 2.55]	Inferior by ≥100%	0.002
[CoC] Furlong HAC Stem Furlong HAC CSF Plus	3,077	1.78	1.24	[0.81 , 1.68]	Inferior by ≥100%	<0.001
[CoC] M/L Taper Cementless Continuum	304	1.68	1.14	[0.25 , 2.04]	Inferior by ≥20%	0.012
[CoC] S-Rom Cementless Stem Pinnacle	251	3.21	2.67	[0.83 , 4.52]	Inferior by ≥100%	0.004
[CoC] SL-Plus Cementless Stem EP-Fit Plus	565	3.43	2.89	[1.42 , 4.36]	Inferior by ≥100%	<0.001
[CoC] Taperloc Cementless Stem Exceed ABT	2,243	1.79	1.25	[0.78 , 1.72]	Inferior by ≥100%	<0.001
[CoP] Accolade Trident	828	1.84	1.31	[0.66 , 1.95]	Inferior by ≥100%	<0.001
[CoP] C-Stem Cemented Stem Elite Plus Ogee	262	0.62	0.08	[-0.82 , 0.98]	Non-inferiority not shown	0.860
[CoP] C-Stem Cemented Stem Marathon	297	1.21	0.67	[-0.13 , 1.48]	Non-inferiority not shown	0.099
[CoP] C-Stem Cemented Stem Opera	374	1.20	0.66	[-0.42 , 1.74]	Non-inferiority not shown	0.232
[CoP] C-Stem Cemented Stem Wroblewski Golf Ball	417	0.73	0.19	[-0.57 , 0.95]	Non-inferiority not shown	0.628
[CoP] Corail Charnley and Elite Plus LPW	321	2.42	1.88	[0.37 , 3.39]	Inferior by ≥20%	0.015
[CoP] Corail Marathon	333	1.04	0.50	[-0.19 , 1.19]	Non-inferiority not shown	0.153
[CoP] Corail Pinnacle	2,442	1.71	1.17	[0.75 , 1.59]	Inferior by ≥100%	<0.001
[CoP] Corail Trilogy	397	1.10	0.56	[-0.44 , 1.56]	Non-inferiority not shown	0.269
[CoP] Exeter V40 Elite Plus Ogee	478	1.06	0.52	[-0.27 , 1.31]	Non-inferiority not shown	0.198
[CoP] Exeter V40 Exeter Contemporary Flanged	1,225	1.60	1.06	[0.45 , 1.66]	Inferior by ≥20%	0.001
[CoP] Exeter V40 Exeter Contemporary Hooded	358	2.31	1.77	[0.48 , 3.06]	Inferior by ≥20%	0.007
[CoP] Exeter V40 Exeter Duration	405	0.93	0.39	[-0.47 , 1.24]	Non-inferiority not shown	0.374
[CoP] Exeter V40 Trident	1,446	1.28	0.75	[0.29 , 1.20]	Inferior by ≥20%	0.001
[CoP] Exeter V40 Trilogy	997	0.86	0.32	[-0.26 , 0.89]	Non-inferiority not shown	0.278
[CoP] Furlong HAC Stem CSF	3,217	1.83	1.29	[0.79 , 1.79]	Inferior by ≥100%	<0.001
[CoP] Furlong HAC Stem Furlong HAC CSF Plus	508	2.55	2.01	[1.01 , 3.01]	Inferior by ≥100%	<0.001
[CoP] MS-30 Original ME Muller Low Profile Cup	796	0.64	0.10	[-0.45 , 0.65]	Non-inferiority not shown	0.725
[CoP] SL-Plus Cementless Stem Bicon-Plus	306	3.49	2.95	[0.99 , 4.91]	Inferior by ≥100%	0.003
[CoP] SL-Plus Cementless Stem EP-Fit Plus	499	3.49	2.95	[1.46 , 4.45]	Inferior by ≥100%	<0.001
[CoP] Taperloc Cementless Stem Exceed ABT	466	1.23	0.69	[0.07 , 1.31]	Non-inferiority not shown	0.029

For peer review only

[MoP] Accolade Trident	3,698	2.36	1.82	[1.34 , 2.29]	Inferior by ≥100%	<0.001
[MoP] C-Stem AMT Cemented Stem Charnley and Elite Plus LPW	954	1.29	0.75	[0.13 , 1.37]	Inferior by ≥20%	0.017
[MoP] C-Stem AMT Cemented Stem Elite Plus Cemented Cup	313	0.85	0.31	[-0.57 , 1.18]	Non-inferiority not shown	0.488
[MoP] C-Stem AMT Cemented Stem Elite Plus Ogee	666	1.18	0.64	[-0.02 , 1.30]	Non-inferiority not shown	0.058
[MoP] C-Stem AMT Cemented Stem Pinnacle	426	1.66	1.12	[0.40 , 1.83]	Inferior by ≥20%	0.002
[MoP] C-Stem Cemented Stem Charnley Ogee	480	2.25	1.71	[0.50 , 2.92]	Inferior by ≥20%	0.006
[MoP] C-Stem Cemented Stem Charnley and Elite Plus LPW	1,007	1.81	1.28	[0.46 , 2.09]	Inferior by ≥20%	0.002
[MoP] C-Stem Cemented Stem Duraloc Cementless Cup	388	2.33	1.79	[0.34 , 3.24]	Inferior by ≥20%	0.016
[MoP] C-Stem Cemented Stem Elite Plus Cemented Cup	357	0.47	-0.07	[-0.77 , 0.64]	Non-inferiority not shown	0.848
[MoP] C-Stem Cemented Stem Elite Plus Ogee	1,554	1.02	0.49	[-0.02 , 0.99]	Non-inferiority not shown	0.062
[MoP] C-Stem Cemented Stem Marathon	473	0.91	0.37	[-0.24 , 0.98]	Non-inferiority not shown	0.235
[MoP] C-Stem Cemented Stem Opera	626	0.95	0.41	[-0.30 , 1.13]	Non-inferiority not shown	0.259
[MoP] C-Stem Cemented Stem Wroblewski Golf Ball	488	1.61	1.07	[0.04 , 2.11]	Non-inferiority not shown	0.042
[MoP] CCA Cemented Stem CCB Cup	535	0.92	0.38	[-0.32 , 1.08]	Non-inferiority not shown	0.289
[MoP] CLS Cementless Stem Allofit	316	2.53	1.99	[0.33 , 3.64]	Inferior by ≥20%	0.019
[MoP] CMK Modular Cemented Stem CMK Cemented Cup	291	1.25	0.72	[-0.54 , 1.97]	Non-inferiority not shown	0.263
[MoP] CPCS Opera	586	1.63	1.09	[0.17 , 2.02]	Inferior by ≥20%	0.020
[MoP] CPS Plus Opera	309	1.78	1.25	[-0.20 , 2.69]	Non-inferiority not shown	0.090
[MoP] CPT Allofit	308	0.58	0.04	[-0.69 , 0.77]	Non-inferiority not shown	0.921
[MoP] CPT Elite Plus Ogee	1,115	1.60	1.06	[0.40 , 1.73]	Inferior by ≥20%	0.002
[MoP] CPT Opera	252	1.84	1.30	[-0.32 , 2.92]	Non-inferiority not shown	0.116
[MoP] CPT Original ME Muller Low Profile Cup	322	1.98	1.44	[0.28 , 2.60]	Inferior by ≥20%	0.015
[MoP] CPT Pinnacle	433	2.03	1.49	[0.27 , 2.72]	Inferior by ≥20%	0.017
[MoP] CPT Trabecular Metal Modular Cementless Cup	399	2.48	1.95	[0.79 , 3.10]	Inferior by ≥100%	0.001
[MoP] CPT Trilogy	3,903	2.10	1.56	[1.11 , 2.01]	Inferior by ≥100%	<0.001
[MoP] CPT ZCA	3,998	1.98	1.44	[1.00 , 1.88]	Inferior by ≥100%	<0.001
[MoP] Charnley Cemented Stem Charnley Cemented Cup	2,265	1.42	0.88	[0.35 , 1.41]	Inferior by ≥20%	0.001
[MoP] Charnley Cemented Stem Charnley Ogee	4,710	1.63	1.10	[0.67 , 1.53]	Inferior by ≥100%	<0.001
[MoP] Charnley Cemented Stem Charnley and Elite Plus LPW	3,628	1.00	0.46	[0.05 , 0.86]	Non-inferiority not shown	0.027
[MoP] Charnley Cemented Stem Opera	697	1.35	0.81	[-0.03 , 1.65]	Non-inferiority not shown	0.058
[MoP] Charnley Cemented Stem Wroblewski Golf Ball	487	1.73	1.19	[0.14 , 2.24]	Inferior by ≥20%	0.027
[MoP] Corail Charnley and Elite Plus LPW	332	1.29	0.75	[-0.21 , 1.71]	Non-inferiority not shown	0.126
[MoP] Corail Duraloc Cementless Cup	2,023	2.10	1.56	[0.90 , 2.22]	Inferior by ≥100%	<0.001
[MoP] Corail Elite Plus Cemented Cup	481	0.99	0.45	[-0.39 , 1.29]	Non-inferiority not shown	0.295
[MoP] Corail Elite Plus Ogee	678	1.65	1.11	[0.30 , 1.93]	Inferior by ≥20%	0.007
[MoP] Corail Marathon	921	0.93	0.40	[-0.06 , 0.85]	Non-inferiority not shown	0.089
[MoP] Corail Pinnacle	10,090	1.56	1.03	[0.71 , 1.35]	Inferior by ≥100%	<0.001
[MoP] Corail Trilogy	859	1.22	0.69	[-0.01 , 1.38]	Non-inferiority not shown	0.054
[MoP] Elite Plus Cemented Stem Elite Plus Ogee	362	1.26	0.72	[-0.41 , 1.86]	Non-inferiority not shown	0.211
[MoP] Exeter V40 ABG II Cementless Cup	458	1.01	0.47	[-0.45 , 1.40]	Non-inferiority not shown	0.315
[MoP] Exeter V40 Cenator Cemented Cup	1,153	2.12	1.58	[0.80 , 2.36]	Inferior by ≥100%	<0.001
[MoP] Exeter V40 Charnley Ogee	900	1.39	0.85	[0.12 , 1.59]	Inferior by ≥20%	0.023
[MoP] Exeter V40 Charnley and Elite Plus LPW	1,309	1.61	1.07	[0.46 , 1.68]	Inferior by ≥20%	0.001
[MoP] Exeter V40 Duraloc Cementless Cup	671	1.82	1.28	[0.26 , 2.30]	Inferior by ≥20%	0.014
[MoP] Exeter V40 EP-Fit Plus	312	2.98	2.44	[0.60 , 4.29]	Inferior by ≥100%	0.009
[MoP] Exeter V40 Elite Plus Ogee	8,315	1.02	0.48	[0.15 , 0.81]	Inferior by ≥20%	0.004

For peer review only

[MoP] Exeter V40 Exceed	250	0.85	0.31	[-0.68 , 1.30]	Non-inferiority not shown	0.541
[MoP] Exeter V40 Exeter Contemporary Flanged	17,934	1.04	0.50	[0.20 , 0.80]	Inferior by ≥20%	0.001
[MoP] Exeter V40 Exeter Contemporary Hooded	7,664	2.02	1.48	[1.10 , 1.85]	Inferior by ≥100%	<0.001
[MoP] Exeter V40 Exeter Duration	7,168	1.43	0.89	[0.53 , 1.25]	Inferior by ≥20%	<0.001
[MoP] Exeter V40 Exeter X3 Rimfit	550	0.92	0.38	[-0.02 , 0.79]	Non-inferiority not shown	0.066
[MoP] Exeter V40 Marathon	364	1.25	0.71	[-0.04 , 1.46]	Non-inferiority not shown	0.064
[MoP] Exeter V40 Opera	1,221	0.98	0.44	[-0.10 , 0.98]	Non-inferiority not shown	0.110
[MoP] Exeter V40 Pinnacle	1,075	1.24	0.70	[0.17 , 1.24]	Inferior by ≥20%	0.010
[MoP] Exeter V40 Reflection Cementless	1,164	1.05	0.51	[-0.10 , 1.13]	Non-inferiority not shown	0.100
[MoP] Exeter V40 Trident	6,743	1.30	0.76	[0.43 , 1.09]	Inferior by ≥20%	<0.001
[MoP] Exeter V40 Trilogy	4,060	1.24	0.70	[0.30 , 1.10]	Inferior by ≥20%	0.001
[MoP] Exeter V40 Ultima Cemented Cup	720	1.90	1.36	[0.37 , 2.35]	Inferior by ≥20%	0.007
[MoP] Furlong Cemented Stem JRI Cemented Cup	892	1.74	1.20	[0.38 , 2.03]	Inferior by ≥20%	0.004
[MoP] Furlong HAC Stem CSF	3,340	2.50	1.96	[1.43 , 2.50]	Inferior by ≥100%	<0.001
[MoP] Furlong HAC Stem Furlong HAC CSF Plus	1,122	2.75	2.21	[1.48 , 2.94]	Inferior by ≥100%	<0.001
[MoP] Furlong HAC Stem Furlong Threaded	269	2.84	2.30	[0.45 , 4.15]	Inferior by ≥20%	0.015
[MoP] MS-30 Original ME Muller Low Profile Cup	366	0.98	0.44	[-0.48 , 1.36]	Non-inferiority not shown	0.351
[MoP] Muller Straight Stem Centerpulse Muller	291	1.85	1.31	[-0.18 , 2.81]	Non-inferiority not shown	0.085
[MoP] Muller Straight Stem Original ME Muller Low Profile Cup	718	1.26	0.72	[0.02 , 1.42]	Non-inferiority not shown	0.044
[MoP] Muller-Biomet Apollo	972	1.50	0.97	[0.24 , 1.69]	Inferior by ≥20%	0.009
[MoP] Muller-Biomet Original ME Muller Low Profile Cup	442	1.79	1.25	[0.25 , 2.26]	Inferior by ≥20%	0.015
[MoP] Omnifit Cemented Stem ODC	559	2.23	1.69	[0.50 , 2.87]	Inferior by ≥20%	0.005
[MoP] SL-Plus Cementless Stem EP-Fit Plus	980	3.12	2.58	[1.62 , 3.54]	Inferior by ≥100%	<0.001
[MoP] SP II Cemented Stem Interplanta	392	2.33	1.79	[0.40 , 3.18]	Inferior by ≥20%	0.012
[MoP] Stanmore Modular Stem Elite Plus Cemented Cup	273	1.19	0.65	[-0.55 , 1.85]	Non-inferiority not shown	0.287
[MoP] Stanmore Modular Stem SHP Cup	483	0.75	0.21	[-0.45 , 0.87]	Non-inferiority not shown	0.534
[MoP] Stanmore Modular Stem Stanmore-Arcom Cup	2,115	1.69	1.15	[0.60 , 1.70]	Inferior by ≥100%	<0.001
[MoP] Synergy Cementless Stem Reflection Cementless	825	0.91	0.38	[-0.31 , 1.06]	Non-inferiority not shown	0.284
[MoP] Taperloc Cementless Stem Exceed	277	1.84	1.30	[-0.18 , 2.79]	Non-inferiority not shown	0.086
[MoP] Taperloc Cementless Stem Exceed ABT	1,136	2.32	1.78	[1.17 , 2.38]	Inferior by ≥100%	<0.001
[MoP] Versys Cementless Stem Trilogy	462	4.99	4.45	[2.56 , 6.34]	Inferior by ≥100%	<0.001

For peer review only

Supplemental table 2c: Difference in Kaplan-Meier failure estimate between a contemporary reference and implants with at least 250 at risk at 7 years since primary in females

Stem/cup brand	Number at risk	Cumulative failure (%)	Difference in failure (%)	95% CI	Equivalence status	p-value
[MoP] Exeter V40 Elite Plus Cemented Cup	1,165	0.76	[REFERENCE]			
[CoC] ABG II Monolithic Cementless Stem ABG II Cementless Cup	441	2.60	1.84	[0.40 , 3.29]	Inferior by ≥20%	0.012
[CoC] ABG II Monolithic Cementless Stem Trident	341	3.21	2.45	[0.80 , 4.10]	Inferior by ≥100%	0.004
[CoC] Accolade Trident	1,744	3.15	2.39	[1.69 , 3.09]	Inferior by ≥100%	<0.001
[CoC] Corail Duraloc Option	466	3.09	2.33	[0.88 , 3.79]	Inferior by ≥100%	0.002
[CoC] Corail Pinnacle	4,397	2.64	1.88	[1.42 , 2.34]	Inferior by ≥100%	<0.001
[CoC] Exeter V40 ABG II Cementless Cup	615	1.55	0.79	[-0.13 , 1.71]	Non-inferiority not shown	0.094
[CoC] Exeter V40 Trident	3,449	1.85	1.09	[0.58 , 1.61]	Inferior by ≥20%	<0.001
[CoC] Furlong HAC Stem CSF	776	3.08	2.32	[1.12 , 3.53]	Inferior by ≥100%	<0.001
[CoC] Furlong HAC Stem Furlong HAC CSF Plus	1,170	2.19	1.43	[0.87 , 2.00]	Inferior by ≥100%	<0.001
[CoC] SL-Plus Cementless Stem EP-Fit Plus	486	3.61	2.85	[1.32 , 4.38]	Inferior by ≥100%	<0.001
[CoC] Taperloc Cementless Stem Exceed ABT	981	1.93	1.17	[0.60 , 1.74]	Inferior by ≥20%	<0.001
[CoP] Accolade Trident	345	1.98	1.22	[0.48 , 1.96]	Inferior by ≥20%	0.001
[CoP] C-Stem Cemented Stem Opera	274	1.84	1.08	[-0.34 , 2.49]	Non-inferiority not shown	0.135
[CoP] C-Stem Cemented Stem Wroblewski Golf Ball	347	1.80	1.05	[-0.27 , 2.37]	Non-inferiority not shown	0.120
[CoP] Corail Pinnacle	1,028	2.07	1.32	[0.74 , 1.89]	Inferior by ≥20%	<0.001
[CoP] Corail Trilogy	310	1.10	0.34	[-0.69 , 1.37]	Non-inferiority not shown	0.513
[CoP] Exeter V40 Elite Plus Ogee	304	1.29	0.54	[-0.41 , 1.49]	Non-inferiority not shown	0.268
[CoP] Exeter V40 Exeter Contemporary Flanged	694	1.78	1.02	[0.32 , 1.73]	Inferior by ≥20%	0.004
[CoP] Exeter V40 Exeter Duration	309	2.02	1.26	[-0.12 , 2.65]	Non-inferiority not shown	0.074
[CoP] Exeter V40 Trident	656	1.46	0.71	[0.13 , 1.28]	Non-inferiority not shown	0.017
[CoP] Exeter V40 Trilogy	723	1.19	0.43	[-0.30 , 1.16]	Non-inferiority not shown	0.249
[CoP] Furlong HAC Stem CSF	2,638	2.37	1.61	[0.99 , 2.22]	Inferior by ≥100%	<0.001
[CoP] MS-30 Original ME Muller Low Profile Cup	489	0.64	-0.12	[-0.72 , 0.48]	Non-inferiority not shown	0.696
[CoP] SL-Plus Cementless Stem EP-Fit Plus	364	4.38	3.63	[1.89 , 5.37]	Inferior by ≥100%	<0.001
[MoP] Accolade Trident	1,704	3.00	2.25	[1.63 , 2.86]	Inferior by ≥100%	<0.001
[MoP] C-Stem AMT Cemented Stem Charnley and Elite Plus LPW	534	1.65	0.89	[0.06 , 1.71]	Non-inferiority not shown	0.035
[MoP] C-Stem AMT Cemented Stem Elite Plus Ogee	378	1.98	1.22	[0.17 , 2.28]	Inferior by ≥20%	0.023
[MoP] C-Stem Cemented Stem Charnley Ogee	384	2.49	1.73	[0.41 , 3.05]	Inferior by ≥20%	0.010
[MoP] C-Stem Cemented Stem Charnley and Elite Plus LPW	915	2.34	1.58	[0.61 , 2.54]	Inferior by ≥20%	0.001
[MoP] C-Stem Cemented Stem Duraloc Cementless Cup	300	3.44	2.68	[0.86 , 4.49]	Inferior by ≥100%	0.004
[MoP] C-Stem Cemented Stem Elite Plus Cemented Cup	295	1.05	0.30	[-0.80 , 1.39]	Non-inferiority not shown	0.598
[MoP] C-Stem Cemented Stem Elite Plus Ogee	1,198	1.54	0.79	[0.10 , 1.47]	Non-inferiority not shown	0.024
[MoP] C-Stem Cemented Stem Opera	463	1.66	0.90	[-0.12 , 1.93]	Non-inferiority not shown	0.084
[MoP] C-Stem Cemented Stem Wroblewski Golf Ball	333	2.18	1.42	[0.10 , 2.73]	Non-inferiority not shown	0.034
[MoP] CPS Plus Opera	279	2.13	1.37	[-0.24 , 2.97]	Non-inferiority not shown	0.095
[MoP] CPT Elite Plus Ogee	741	2.31	1.55	[0.65 , 2.45]	Inferior by ≥20%	0.001
[MoP] CPT Trilogy	2,474	2.68	1.92	[1.34 , 2.50]	Inferior by ≥100%	<0.001
[MoP] CPT ZCA	2,612	2.46	1.70	[1.15 , 2.26]	Inferior by ≥100%	<0.001
[MoP] Charnley Cemented Stem Charnley Cemented Cup	1,879	1.93	1.18	[0.52 , 1.83]	Inferior by ≥20%	<0.001

For peer review only

[MoP] Charnley Cemented Stem Charnley Ogee	3,746	1.93	1.18	[0.65 , 1.70]	Inferior by ≥20%	<0.001
[MoP] Charnley Cemented Stem Charnley and Elite Plus LPW	2,920	1.35	0.59	[0.08 , 1.11]	Non-inferiority not shown	0.024
[MoP] Charnley Cemented Stem Opera	496	2.12	1.37	[0.26 , 2.47]	Inferior by ≥20%	0.015
[MoP] Charnley Cemented Stem Wroblewski Golf Ball	404	1.73	0.97	[-0.11 , 2.05]	Non-inferiority not shown	0.079
[MoP] Corail Duraloc Cementless Cup	1,695	3.25	2.49	[1.65 , 3.34]	Inferior by ≥100%	<0.001
[MoP] Corail Elite Plus Cemented Cup	322	1.46	0.70	[-0.39 , 1.79]	Non-inferiority not shown	0.208
[MoP] Corail Elite Plus Ogee	456	1.85	1.09	[0.16 , 2.03]	Inferior by ≥20%	0.022
[MoP] Corail Pinnacle	4,975	2.01	1.25	[0.82 , 1.68]	Inferior by ≥100%	<0.001
[MoP] Corail Trilogy	533	1.87	1.11	[0.14 , 2.08]	Non-inferiority not shown	0.025
[MoP] Elite Plus Cemented Stem Elite Plus Ogee	329	1.26	0.50	[-0.66 , 1.67]	Non-inferiority not shown	0.394
[MoP] Exeter V40 ABG II Cementless Cup	416	1.25	0.49	[-0.57 , 1.55]	Non-inferiority not shown	0.365
[MoP] Exeter V40 Cenator Cemented Cup	860	2.22	1.46	[0.62 , 2.30]	Inferior by ≥20%	0.001
[MoP] Exeter V40 Charnley Ogee	721	1.39	0.64	[-0.14 , 1.41]	Non-inferiority not shown	0.108
[MoP] Exeter V40 Charnley and Elite Plus LPW	788	1.99	1.23	[0.48 , 1.99]	Inferior by ≥20%	0.001
[MoP] Exeter V40 Duraloc Cementless Cup	507	2.12	1.37	[0.24 , 2.49]	Inferior by ≥20%	0.017
[MoP] Exeter V40 EP-Fit Plus	268	3.64	2.88	[0.82 , 4.94]	Inferior by ≥100%	0.006
[MoP] Exeter V40 Elite Plus Ogee	5,848	1.40	0.65	[0.21 , 1.08]	Inferior by ≥20%	0.004
[MoP] Exeter V40 Exeter Contemporary Flanged	11,143	1.36	0.60	[0.20 , 1.00]	Inferior by ≥20%	0.003
[MoP] Exeter V40 Exeter Contemporary Hooded	4,810	2.77	2.01	[1.52 , 2.51]	Inferior by ≥100%	<0.001
[MoP] Exeter V40 Exeter Duration	5,153	2.05	1.29	[0.81 , 1.77]	Inferior by ≥100%	<0.001
[MoP] Exeter V40 Opera	805	1.35	0.59	[-0.10 , 1.29]	Non-inferiority not shown	0.095
[MoP] Exeter V40 Pinnacle	507	1.45	0.69	[0.04 , 1.35]	Non-inferiority not shown	0.038
[MoP] Exeter V40 Reflection Cementless	877	1.73	0.97	[0.14 , 1.80]	Non-inferiority not shown	0.022
[MoP] Exeter V40 Trident	3,625	1.74	0.99	[0.53 , 1.44]	Inferior by ≥20%	<0.001
[MoP] Exeter V40 Trilogy	2,694	1.53	0.77	[0.27 , 1.28]	Inferior by ≥20%	0.003
[MoP] Exeter V40 Ultima Cemented Cup	635	2.04	1.28	[0.22 , 2.33]	Inferior by ≥20%	0.018
[MoP] Furlong Cemented Stem JRI Cemented Cup	730	2.09	1.33	[0.39 , 2.28]	Inferior by ≥20%	0.006
[MoP] Furlong HAC Stem CSF	2,586	3.04	2.28	[1.64 , 2.92]	Inferior by ≥100%	<0.001
[MoP] Furlong HAC Stem Furlong HAC CSF Plus	469	3.16	2.40	[1.53 , 3.27]	Inferior by ≥100%	<0.001
[MoP] Muller Straight Stem Original ME Muller Low Profile Cup	474	2.09	1.33	[0.29 , 2.37]	Inferior by ≥20%	0.012
[MoP] Muller-Biomet Apollo	714	1.85	1.09	[0.23 , 1.95]	Inferior by ≥20%	0.013
[MoP] Omnifit Cemented Stem ODC	495	3.36	2.60	[1.10 , 4.10]	Inferior by ≥100%	0.001
[MoP] SL-Plus Cementless Stem EP-Fit Plus	750	3.80	3.04	[1.92 , 4.16]	Inferior by ≥100%	<0.001
[MoP] SP II Cemented Stem Interplanta	326	2.88	2.12	[0.52 , 3.72]	Inferior by ≥20%	0.009
[MoP] Stanmore Modular Stem SHP Cup	352	1.24	0.48	[-0.50 , 1.46]	Non-inferiority not shown	0.338
[MoP] Stanmore Modular Stem Stanmore-Arcom Cup	1,379	2.01	1.25	[0.59 , 1.90]	Inferior by ≥20%	<0.001
[MoP] Synergy Cementless Stem Reflection Cementless	607	1.33	0.57	[-0.30 , 1.44]	Non-inferiority not shown	0.196
[MoP] Taperloc Cementless Stem Exceed ABT	346	2.99	2.24	[1.34 , 3.13]	Inferior by ≥100%	<0.001
[MoP] Versys Cementless Stem Trilogy	418	4.99	4.23	[2.32 , 6.14]	Inferior by ≥100%	<0.001

For peer review only

Supplemental table 2d: Difference in Kaplan-Meier failure estimate between a contemporary reference and implants with at least 250 at risk at 10 years since primary in females

Stem/cup brand	Number at risk	Cumulative failure (%)	Difference in failure (%)	95% CI	Equivalence status	p-value
[MoP] Exeter V40 Elite Plus Ogee	2,475	1.95	[REFERENCE]			
[CoC] ABG II Monolithic Cementless Stem ABG II Cementless Cup	347	3.34	1.38	[-0.27 , 3.03]	Non-inferiority not shown	0.100
[CoC] Accolade Trident	255	4.07	2.11	[1.17 , 3.05]	Inferior by ≥20%	<0.001
[CoC] Corail Pinnacle	523	3.61	1.65	[1.01 , 2.30]	Inferior by ≥20%	<0.001
[CoC] Exeter V40 ABG II Cementless Cup	302	2.63	0.67	[-0.64 , 1.98]	Non-inferiority not shown	0.317
[CoC] Exeter V40 Trident	1,079	2.45	0.50	[-0.11 , 1.11]	Non-inferiority not shown	0.106
[CoC] Furlong HAC Stem CSF	470	4.44	2.49	[1.01 , 3.97]	Inferior by ≥20%	0.001
[CoP] Exeter V40 Trilogy	300	1.58	-0.37	[-1.28 , 0.53]	Non-inferiority not shown	0.417
[CoP] Furlong HAC Stem CSF	1,467	3.08	1.12	[0.42 , 1.82]	Inferior by ≥20%	0.002
[MoP] C-Stem Cemented Stem Charnley and Elite Plus LPW	534	2.79	0.84	[-0.21 , 1.88]	Non-inferiority not shown	0.117
[MoP] C-Stem Cemented Stem Elite Plus Ogee	570	2.48	0.53	[-0.41 , 1.46]	Non-inferiority not shown	0.271
[MoP] CPT Elite Plus Ogee	256	2.80	0.85	[-0.31 , 2.01]	Non-inferiority not shown	0.151
[MoP] CPT Trilogy	754	4.11	2.16	[1.31 , 3.01]	Inferior by ≥20%	<0.001
[MoP] CPT ZCA	903	3.43	1.48	[0.76 , 2.19]	Inferior by ≥20%	<0.001
[MoP] Charnley Cemented Stem Charnley Cemented Cup	1,136	2.83	0.88	[0.10 , 1.66]	Non-inferiority not shown	0.027
[MoP] Charnley Cemented Stem Charnley Ogee	1,951	3.23	1.28	[0.63 , 1.92]	Inferior by ≥20%	<0.001
[MoP] Charnley Cemented Stem Charnley and Elite Plus LPW	1,623	2.23	0.28	[-0.34 , 0.90]	Non-inferiority not shown	0.376
[MoP] Charnley Cemented Stem Wroblewski Golf Ball	252	2.62	0.67	[-0.81 , 2.14]	Non-inferiority not shown	0.375
[MoP] Corail Duraloc Cementless Cup	783	4.61	2.66	[1.62 , 3.70]	Inferior by ≥20%	<0.001
[MoP] Corail Pinnacle	1,043	2.78	0.83	[0.28 , 1.38]	Non-inferiority not shown	0.003
[MoP] Exeter V40 ABG II Cementless Cup	279	1.55	-0.40	[-1.60 , 0.80]	Non-inferiority not shown	0.515
[MoP] Exeter V40 Cenator Cemented Cup	468	2.50	0.55	[-0.36 , 1.45]	Non-inferiority not shown	0.239
[MoP] Exeter V40 Charnley Ogee	434	1.39	-0.56	[-1.32 , 0.20]	Non-inferior	0.146
[MoP] Exeter V40 Charnley and Elite Plus LPW	292	2.60	0.65	[-0.30 , 1.60]	Non-inferiority not shown	0.180
[MoP] Exeter V40 Duraloc Cementless Cup	301	3.88	1.92	[0.23 , 3.62]	Non-inferiority not shown	0.026
[MoP] Exeter V40 Elite Plus Cemented Cup	480	1.04	-0.91	[-1.55 , -0.28]	Non-inferior	0.005
[MoP] Exeter V40 Exeter Contemporary Flanged	3,136	1.98	0.02	[-0.39 , 0.43]	Non-inferiority not shown	0.919
[MoP] Exeter V40 Exeter Contemporary Hooded	1,595	4.09	2.14	[1.52 , 2.75]	Inferior by ≥20%	<0.001
[MoP] Exeter V40 Exeter Duration	2,079	3.16	1.21	[0.64 , 1.78]	Inferior by ≥20%	<0.001
[MoP] Exeter V40 Opera	315	2.79	0.83	[-0.44 , 2.11]	Non-inferiority not shown	0.200
[MoP] Exeter V40 Trident	714	2.63	0.68	[0.06 , 1.29]	Non-inferiority not shown	0.031
[MoP] Exeter V40 Trilogy	955	2.15	0.20	[-0.40 , 0.80]	Non-inferiority not shown	0.518
[MoP] Exeter V40 Ultima Cemented Cup	334	2.65	0.70	[-0.55 , 1.94]	Non-inferiority not shown	0.275
[MoP] Furlong Cemented Stem JRI Cemented Cup	413	3.49	1.53	[0.20 , 2.87]	Non-inferiority not shown	0.024
[MoP] Furlong HAC Stem CSF	1,088	4.21	2.25	[1.46 , 3.04]	Inferior by ≥20%	<0.001
[MoP] Muller-Biomet Apollo	256	2.18	0.23	[-0.84 , 1.30]	Non-inferiority not shown	0.673
[MoP] Omnifit Cemented Stem ODC	321	4.49	2.54	[0.76 , 4.32]	Inferior by ≥20%	0.005
[MoP] Stanmore Modular Stem Stanmore-Arcom Cup	489	2.78	0.83	[-0.01 , 1.66]	Non-inferiority not shown	0.052
[MoP] Versys Cementless Stem Trilogy	263	5.52	3.57	[1.54 , 5.60]	Inferior by ≥20%	0.001

For peer review only

For peer review only

Supplemental table 3a: Difference in Kaplan-Meier failure estimate between a contemporary reference and implants with at least 250 at risk at 3 years since primary in females <55 years

Stem/cup brand	Number at risk	Cumulative failure (%)	Difference in failure (%)	95% CI	Equivalence status	p-value
[CoC] Exeter V40 Trident	1,785	0.79	[REFERENCE]			
[CoC] Accolade Trident	865	2.12	1.33	[0.33 , 2.33]	Inferior by ≥20%	0.009
[CoC] Corail DeltaMotion	342	1.53	0.74	[-0.54 , 2.02]	Non-inferiority not shown	0.256
[CoC] Corail Pinnacle	4,110	2.15	1.36	[0.81 , 1.92]	Inferior by ≥100%	<0.001
[CoC] Furlong HAC Stem Furlong HAC CSF Plus	969	1.57	0.78	[-0.01 , 1.57]	Non-inferiority not shown	0.052
[CoC] M/L Taper Cementless Continuum	324	1.22	0.43	[-0.70 , 1.57]	Non-inferiority not shown	0.456
[CoC] Taperloc Cementless Stem Exceed ABT	964	1.23	0.44	[-0.27 , 1.16]	Non-inferiority not shown	0.225
[CoP] Accolade Trident	313	1.01	0.22	[-0.67 , 1.12]	Non-inferiority not shown	0.629
[CoP] C-Stem Cemented Stem Marathon	251	1.23	0.44	[-0.71 , 1.58]	Non-inferiority not shown	0.457
[CoP] Corail Pinnacle	720	1.95	1.16	[0.31 , 2.00]	Inferior by ≥20%	0.007
[CoP] Exeter V40 Exeter X3 Rimfit	309	1.29	0.50	[-0.55 , 1.56]	Non-inferiority not shown	0.351
[CoP] Exeter V40 Trident	493	1.06	0.27	[-0.51 , 1.05]	Non-inferiority not shown	0.501
[CoP] Furlong HAC Stem CSF	259	1.81	1.02	[-0.60 , 2.64]	Non-inferiority not shown	0.217
[MoP] Accolade Trident	256	1.87	1.08	[-0.45 , 2.61]	Non-inferiority not shown	0.168
[MoP] Corail Pinnacle	594	1.73	0.94	[-0.05 , 1.93]	Non-inferiority not shown	0.062
[MoP] Exeter V40 Exeter Contemporary Flanged	366	0.56	-0.23	[-0.97 , 0.50]	Non-inferiority not shown	0.535
[MoP] Exeter V40 Trident	344	1.05	0.26	[-0.74 , 1.27]	Non-inferiority not shown	0.610

For peer review only

Supplemental table 3b: Difference in Kaplan-Meier failure estimate between a contemporary reference and implants with at least 250 at risk at 5 years since primary in females <55 years

Stem/cup brand	Number at risk	Cumulative failure (%)	Difference in failure (%)	95% CI	Equivalence status	p-value
[CoC] Exeter V40 Trident	1,426	1.17	[REFERENCE]			
[CoC] Accolade Trident	669	2.64	1.47	[0.31 , 2.62]	Inferior by ≥20%	0.013
[CoC] Corail Pinnacle	2,754	2.93	1.76	[1.07 , 2.45]	Inferior by ≥20%	<0.001
[CoC] Furlong HAC Stem Furlong HAC CSF Plus	610	1.82	0.65	[-0.26 , 1.57]	Non-inferiority not shown	0.161
[CoC] Taperloc Cementless Stem Exceed ABT	557	2.05	0.88	[-0.11 , 1.86]	Non-inferiority not shown	0.080
[CoP] Corail Pinnacle	369	2.59	1.42	[0.33 , 2.52]	Inferior by ≥20%	0.011
[MoP] Corail Pinnacle	365	2.65	1.48	[0.18 , 2.78]	Non-inferiority not shown	0.026
[MoP] Exeter V40 Exeter Contemporary Flanged	279	1.81	0.64	[-0.82 , 2.10]	Non-inferiority not shown	0.391

For peer review only

Supplemental table 3c: Difference in Kaplan-Meier failure estimate between a contemporary reference and implants with at least 250 at risk at 7 years since primary in females <55 years

Stem/cup brand	Number at risk	Cumulative failure (%)	Difference in failure (%)	95% CI	Equivalence status	p-value
[CoC] Corail Pinnacle	1,321	3.60	[REFERENCE]			
[CoC] Accolade Trident	370	3.66	0.06	[-1.38 , 1.51]	Non-inferiority not shown	0.930
[CoC] Exeter V40 Trident	982	2.07	-1.53	[-2.47 , -0.60]	Non-inferior	0.001

For peer review only

Supplemental table 4a: Difference in Kaplan-Meier failure estimate between a contemporary reference and implants with at least 250 at risk at 3 years since primary in females between 55 and 75 years

Stem/cup brand	Number at risk	Cumulative failure (%)	Difference in failure (%)	95% CI	Equivalence status	p-value
[MoP] Exeter V40 Elite Plus Cemented Cup	1,360	0.44	[REFERENCE]			
[CoC] ABG II Monolithic Cementless Stem ABG II Cementless Cup	375	1.57	1.13	[-0.16 , 2.42]	Non-inferiority not shown	0.087
[CoC] ABG II Monolithic Cementless Stem Trident	325	2.05	1.61	[0.07 , 3.14]	Non-inferiority not shown	0.041
[CoC] Accolade Trident	2,547	1.62	1.18	[0.60 , 1.75]	Inferior by ≥100%	<0.001
[CoC] Bimetric Cementless Stem Exceed ABT	407	1.48	1.04	[-0.10 , 2.17]	Non-inferiority not shown	0.074
[CoC] C-Stem AMT Cemented Stem Pinnacle	368	1.00	0.56	[-0.38 , 1.50]	Non-inferiority not shown	0.245
[CoC] CPT Continuum	275	0.90	0.45	[-0.61 , 1.52]	Non-inferiority not shown	0.405
[CoC] Corail Delta TT	293	2.02	1.58	[0.29 , 2.86]	Inferior by ≥20%	0.016
[CoC] Corail DeltaMotion	347	0.95	0.51	[-0.48 , 1.50]	Non-inferiority not shown	0.312
[CoC] Corail Duraloc Option	382	1.52	1.08	[-0.17 , 2.33]	Non-inferiority not shown	0.092
[CoC] Corail Pinnacle	9,567	1.41	0.97	[0.57 , 1.36]	Inferior by ≥100%	<0.001
[CoC] Excia Cementless Plasmacup SC	325	1.03	0.58	[-0.48 , 1.64]	Non-inferiority not shown	0.281
[CoC] Exeter V40 ABG II Cementless Cup	640	0.41	-0.03	[-0.60 , 0.54]	Non-inferiority not shown	0.909
[CoC] Exeter V40 Trident	3,977	0.95	0.50	[0.07 , 0.94]	Non-inferiority not shown	0.024
[CoC] Furlong HAC Stem CSF	620	1.73	1.28	[0.22 , 2.34]	Inferior by ≥20%	0.018
[CoC] Furlong HAC Stem Furlong HAC CSF Plus	3,449	1.59	1.15	[0.66 , 1.63]	Inferior by ≥100%	<0.001
[CoC] M/L Taper Cementless Continuum	363	1.61	1.16	[0.01 , 2.32]	Non-inferiority not shown	0.048
[CoC] Metafix Stem Trinity	290	1.39	0.94	[-0.16 , 2.04]	Non-inferiority not shown	0.093
[CoC] Polarstem Cementless R3 Cementless	370	0.84	0.40	[-0.49 , 1.29]	Non-inferiority not shown	0.377
[CoC] SL-Plus Cementless Stem EP-Fit Plus	376	1.53	1.09	[-0.17 , 2.34]	Non-inferiority not shown	0.091
[CoC] Taperloc Cementless Stem Exceed ABT	2,580	1.46	1.01	[0.50 , 1.53]	Inferior by ≥100%	<0.001
[CoP] Accolade Trident	1,324	1.44	0.99	[0.36 , 1.63]	Inferior by ≥20%	0.002
[CoP] C-Stem Cemented Stem Elite Plus Ogee	279	0.35	-0.09	[-0.85 , 0.66]	Non-inferiority not shown	0.807
[CoP] C-Stem Cemented Stem Marathon	364	0.59	0.15	[-0.62 , 0.91]	Non-inferiority not shown	0.706
[CoP] C-Stem Cemented Stem Opera	295	0.00	- -	[-.-- , -.--]	No failures to date	
[CoP] C-Stem Cemented Stem Wroblewski Golf Ball	346	0.53	0.08	[-0.72 , 0.89]	Non-inferiority not shown	0.836
[CoP] CPT Trilogy	573	1.20	0.75	[0.13 , 1.38]	Inferior by ≥20%	0.018
[CoP] Corail Charnley and Elite Plus LPW	296	2.41	1.97	[0.28 , 3.65]	Inferior by ≥20%	0.022
[CoP] Corail Marathon	559	0.73	0.29	[-0.35 , 0.93]	Non-inferiority not shown	0.378
[CoP] Corail Pinnacle	3,489	1.19	0.75	[0.33 , 1.17]	Inferior by ≥20%	<0.001
[CoP] Corail Trilogy	355	0.81	0.37	[-0.60 , 1.34]	Non-inferiority not shown	0.460
[CoP] Exeter V40 Charnley and Elite Plus LPW	355	1.69	1.24	[0.03 , 2.45]	Non-inferiority not shown	0.044
[CoP] Exeter V40 Elite Plus Ogee	529	0.70	0.26	[-0.44 , 0.95]	Non-inferiority not shown	0.468
[CoP] Exeter V40 Exeter Contemporary Flanged	1,464	1.06	0.62	[0.05 , 1.18]	Non-inferiority not shown	0.032
[CoP] Exeter V40 Exeter Contemporary Hooded	376	1.55	1.11	[-0.08 , 2.30]	Non-inferiority not shown	0.068
[CoP] Exeter V40 Exeter Duration	369	0.98	0.54	[-0.48 , 1.55]	Non-inferiority not shown	0.299
[CoP] Exeter V40 Exeter X3 Rimfit	896	0.66	0.22	[-0.27 , 0.70]	Non-inferiority not shown	0.385
[CoP] Exeter V40 Trident	2,023	1.02	0.57	[0.12 , 1.03]	Inferior by ≥20%	0.014
[CoP] Exeter V40 Trilogy	896	0.61	0.16	[-0.42 , 0.75]	Non-inferiority not shown	0.587
[CoP] Furlong HAC Stem CSF	2,797	1.29	0.85	[0.33 , 1.37]	Inferior by ≥20%	0.001

For peer review only

[CoP] Furlong HAC Stem Furlong HAC CSF Plus	660	2.00	1.56	[0.58 , 2.54]	Inferior by ≥100%	0.002
[CoP] MS-30 Original ME Muller Low Profile Cup	785	0.57	0.13	[-0.47 , 0.73]	Non-inferiority not shown	0.674
[CoP] SL-Plus Cementless Stem EP-Fit Plus	368	1.82	1.38	[-0.00 , 2.75]	Non-inferiority not shown	0.050
[CoP] Taperloc Cementless Stem Exceed ABT	789	1.04	0.60	[-0.03 , 1.22]	Non-inferiority not shown	0.062
[MoP] Accolade Trident	3,609	1.81	1.36	[0.84 , 1.89]	Inferior by ≥100%	<0.001
[MoP] Anthology R3 Cementless	506	1.00	0.56	[-0.18 , 1.29]	Non-inferiority not shown	0.138
[MoP] C-Stem AMT Cemented Stem Charnley and Elite Plus LPW	779	0.76	0.32	[-0.34 , 0.97]	Non-inferiority not shown	0.341
[MoP] C-Stem AMT Cemented Stem Elite Plus Ogee	482	0.79	0.35	[-0.43 , 1.12]	Non-inferiority not shown	0.380
[MoP] C-Stem AMT Cemented Stem Marathon	406	1.09	0.65	[-0.12 , 1.42]	Non-inferiority not shown	0.099
[MoP] C-Stem AMT Cemented Stem Pinnacle	609	1.05	0.60	[-0.07 , 1.27]	Non-inferiority not shown	0.077
[MoP] C-Stem Cemented Stem Charnley Ogee	400	1.80	1.35	[0.07 , 2.64]	Non-inferiority not shown	0.038
[MoP] C-Stem Cemented Stem Charnley and Elite Plus LPW	727	1.04	0.60	[-0.19 , 1.39]	Non-inferiority not shown	0.138
[MoP] C-Stem Cemented Stem Duraloc Cementless Cup	298	1.64	1.20	[-0.27 , 2.67]	Non-inferiority not shown	0.109
[MoP] C-Stem Cemented Stem Elite Plus Ogee	1,130	0.91	0.47	[-0.14 , 1.08]	Non-inferiority not shown	0.132
[MoP] C-Stem Cemented Stem Marathon	616	0.67	0.22	[-0.37 , 0.82]	Non-inferiority not shown	0.461
[MoP] C-Stem Cemented Stem Opera	541	0.71	0.26	[-0.50 , 1.03]	Non-inferiority not shown	0.500
[MoP] C-Stem Cemented Stem Wroblewski Golf Ball	397	0.48	0.04	[-0.71 , 0.78]	Non-inferiority not shown	0.925
[MoP] CCA Cemented Stem CCB Cup	457	0.34	-0.10	[-0.68 , 0.47]	Non-inferiority not shown	0.728
[MoP] CLS Cementless Stem Allofit	259	1.45	1.01	[-0.44 , 2.46]	Non-inferiority not shown	0.174
[MoP] CPCS Opera	488	1.19	0.75	[-0.26 , 1.75]	Non-inferiority not shown	0.145
[MoP] CPT Allofit	386	0.16	-0.28	[-0.74 , 0.17]	Non-inferiority not shown	0.220
[MoP] CPT Elite Plus Ogee	922	1.28	0.84	[0.07 , 1.61]	Non-inferiority not shown	0.032
[MoP] CPT Original ME Muller Low Profile Cup	324	0.92	0.48	[-0.48 , 1.44]	Non-inferiority not shown	0.329
[MoP] CPT Pinnacle	290	0.64	0.20	[-0.75 , 1.15]	Non-inferiority not shown	0.680
[MoP] CPT Trabecular Metal Modular Cementless Cup	317	2.73	2.29	[0.72 , 3.86]	Inferior by ≥100%	0.004
[MoP] CPT Trilogy	3,394	1.23	0.79	[0.32 , 1.26]	Inferior by ≥20%	0.001
[MoP] CPT ZCA	2,305	1.24	0.79	[0.28 , 1.31]	Inferior by ≥20%	0.003
[MoP] Charnley Cemented Stem Charnley Cemented Cup	1,559	0.98	0.54	[-0.04 , 1.12]	Non-inferiority not shown	0.069
[MoP] Charnley Cemented Stem Charnley Ogee	3,266	1.18	0.73	[0.25 , 1.22]	Inferior by ≥20%	0.003
[MoP] Charnley Cemented Stem Charnley and Elite Plus LPW	2,255	0.68	0.23	[-0.24 , 0.70]	Non-inferiority not shown	0.331
[MoP] Charnley Cemented Stem Opera	498	0.39	-0.05	[-0.69 , 0.58]	Non-inferiority not shown	0.868
[MoP] Charnley Cemented Stem Wroblewski Golf Ball	333	1.43	0.98	[-0.30 , 2.27]	Non-inferiority not shown	0.134
[MoP] Corail Charnley and Elite Plus LPW	334	0.23	-0.21	[-0.77 , 0.35]	Non-inferiority not shown	0.454
[MoP] Corail Duraloc Cementless Cup	1,523	1.59	1.14	[0.44 , 1.84]	Inferior by ≥20%	0.001
[MoP] Corail Elite Plus Cemented Cup	354	0.25	-0.20	[-0.78 , 0.39]	Non-inferiority not shown	0.510
[MoP] Corail Elite Plus Ogee	546	1.03	0.58	[-0.24 , 1.41]	Non-inferiority not shown	0.167
[MoP] Corail Marathon	1,156	0.74	0.30	[-0.21 , 0.80]	Non-inferiority not shown	0.246
[MoP] Corail Pinnacle	11,626	1.15	0.71	[0.34 , 1.08]	Inferior by ≥20%	<0.001
[MoP] Corail Trilogy	680	0.78	0.34	[-0.37 , 1.04]	Non-inferiority not shown	0.350
[MoP] Exeter V40 ABG II Cementless Cup	288	1.01	0.56	[-0.62 , 1.74]	Non-inferiority not shown	0.351
[MoP] Exeter V40 Cenator Cemented Cup	721	1.68	1.24	[0.27 , 2.20]	Inferior by ≥20%	0.012
[MoP] Exeter V40 Charnley Ogee	658	1.07	0.63	[-0.18 , 1.44]	Non-inferiority not shown	0.129
[MoP] Exeter V40 Charnley and Elite Plus LPW	943	1.27	0.82	[0.11 , 1.54]	Inferior by ≥20%	0.025
[MoP] Exeter V40 Duraloc Cementless Cup	509	0.77	0.33	[-0.49 , 1.15]	Non-inferiority not shown	0.437
[MoP] Exeter V40 EP-Fit Plus	265	1.11	0.67	[-0.63 , 1.96]	Non-inferiority not shown	0.312
[MoP] Exeter V40 Elite Plus Ogee	5,613	0.77	0.33	[-0.07 , 0.72]	Non-inferiority not shown	0.103

For peer review only

[MoP] Exeter V40 Exeter Contemporary Flanged	14,698	0.81	0.36	[0.01 , 0.72]	Non-inferiority not shown	0.043
[MoP] Exeter V40 Exeter Contemporary Hooded	5,787	1.59	1.14	[0.70 , 1.58]	Inferior by ≥100%	<0.001
[MoP] Exeter V40 Exeter Duration	4,914	1.14	0.70	[0.26 , 1.13]	Inferior by ≥20%	0.002
[MoP] Exeter V40 Exeter X3 Rimfit	1,966	0.71	0.27	[-0.15 , 0.68]	Non-inferiority not shown	0.210
[MoP] Exeter V40 Marathon	486	0.87	0.42	[-0.31 , 1.16]	Non-inferiority not shown	0.261
[MoP] Exeter V40 Opera	876	0.86	0.41	[-0.26 , 1.09]	Non-inferiority not shown	0.232
[MoP] Exeter V40 Pinnacle	1,011	1.06	0.61	[-0.02 , 1.25]	Non-inferiority not shown	0.058
[MoP] Exeter V40 Reflection Cementless	785	0.74	0.30	[-0.38 , 0.97]	Non-inferiority not shown	0.389
[MoP] Exeter V40 Trident	6,447	1.11	0.67	[0.28 , 1.06]	Inferior by ≥20%	0.001
[MoP] Exeter V40 Trilogy	3,453	0.79	0.34	[-0.09 , 0.77]	Non-inferiority not shown	0.119
[MoP] Exeter V40 Ultima Cemented Cup	425	1.38	0.93	[-0.21 , 2.08]	Non-inferiority not shown	0.109
[MoP] Furlong Cemented Stem JRI Cemented Cup	495	1.90	1.46	[0.24 , 2.67]	Inferior by ≥20%	0.019
[MoP] Furlong HAC Stem CSF	2,428	1.91	1.47	[0.85 , 2.08]	Inferior by ≥100%	<0.001
[MoP] Furlong HAC Stem Furlong HAC CSF Plus	1,020	1.79	1.35	[0.60 , 2.10]	Inferior by ≥100%	<0.001
[MoP] M/L Taper Cementless Continuum	253	1.51	1.07	[-0.14 , 2.28]	Non-inferiority not shown	0.084
[MoP] Muller Straight Stem Original ME Muller Low Profile Cup	530	1.06	0.62	[-0.24 , 1.47]	Non-inferiority not shown	0.157
[MoP] Muller-Biomet Apollo	634	1.93	1.49	[0.40 , 2.58]	Inferior by ≥20%	0.008
[MoP] Muller-Biomet Original ME Muller Low Profile Cup	362	1.57	1.12	[-0.16 , 2.41]	Non-inferiority not shown	0.087
[MoP] Omnifit Cemented Stem ODC	358	1.63	1.19	[-0.15 , 2.53]	Non-inferiority not shown	0.081
[MoP] Polarstem Cementless R3 Cementless	355	0.64	0.20	[-0.35 , 0.75]	Non-inferiority not shown	0.479
[MoP] SL-Plus Cementless Stem EP-Fit Plus	857	3.12	2.68	[1.56 , 3.79]	Inferior by ≥100%	<0.001
[MoP] SP II Cemented Stem Interplanta	258	2.11	1.67	[-0.04 , 3.37]	Non-inferiority not shown	0.055
[MoP] Stanmore Modular Stem SHP Cup	298	0.89	0.44	[-0.61 , 1.49]	Non-inferiority not shown	0.411
[MoP] Stanmore Modular Stem Stanmore-Arcom Cup	1,365	1.33	0.89	[0.22 , 1.56]	Inferior by ≥20%	0.009
[MoP] Synergy Cementless Stem R3 Cementless	363	0.63	0.19	[-0.46 , 0.83]	Non-inferiority not shown	0.571
[MoP] Synergy Cementless Stem Reflection Cementless	598	0.81	0.37	[-0.41 , 1.15]	Non-inferiority not shown	0.357
[MoP] Taperloc Cementless Stem Exceed ABT	1,475	2.08	1.63	[0.94 , 2.33]	Inferior by ≥100%	<0.001
[MoP] Versys Cementless Stem Trilogy	363	3.38	2.94	[1.10 , 4.78]	Inferior by ≥100%	0.002

Supplemental table 4b: Difference in Kaplan-Meier failure estimate between a contemporary reference and implants with at least 250 at risk at 5 years since primary in females between 55 and 75 years

Stem/cup brand	Number at risk	Cumulative failure (%)	Difference in failure (%)	95% CI	Equivalence status	p-value
[MoP] Exeter V40 Elite Plus Cemented Cup	1,039	0.52	[REFERENCE]			
[CoC] ABG II Monolithic Cementless Stem ABG II Cementless Cup	363	1.84	1.32	[-0.08 , 2.71]	Non-inferiority not shown	0.064
[CoC] ABG II Monolithic Cementless Stem Trident	250	3.09	2.57	[0.64 , 4.49]	Inferior by ≥100%	0.009
[CoC] Accolade Trident	2,044	2.56	2.04	[1.33 , 2.75]	Inferior by ≥100%	<0.001
[CoC] Bimetric Cementless Stem Exceed ABT	289	2.32	1.80	[0.32 , 3.28]	Inferior by ≥20%	0.017
[CoC] Corail Duraloc Option	370	2.82	2.30	[0.62 , 3.99]	Inferior by ≥100%	0.007
[CoC] Corail Pinnacle	6,445	1.82	1.30	[0.86 , 1.74]	Inferior by ≥100%	<0.001

For peer review only

[CoC] Exeter V40 ABG II Cementless Cup	563	1.05	0.53	[-0.33 , 1.38]	Non-inferiority not shown	0.225
[CoC] Exeter V40 Trident	3,263	1.29	0.77	[0.27 , 1.26]	Inferior by ≥20%	0.002
[CoC] Furlong HAC Stem CSF	597	2.05	1.53	[0.37 , 2.68]	Inferior by ≥20%	0.010
[CoC] Furlong HAC Stem Furlong HAC CSF Plus	2,167	1.86	1.34	[0.80 , 1.88]	Inferior by ≥100%	<0.001
[CoC] SL-Plus Cementless Stem EP-Fit Plus	352	3.15	2.63	[0.84 , 4.43]	Inferior by ≥100%	0.004
[CoC] Taperloc Cementless Stem Exceed ABT	1,583	1.68	1.16	[0.59 , 1.72]	Inferior by ≥100%	<0.001
[CoP] Accolade Trident	638	1.84	1.32	[0.56 , 2.08]	Inferior by ≥100%	0.001
[CoP] C-Stem Cemented Stem Wroblewski Golf Ball	283	0.82	0.30	[-0.69 , 1.30]	Non-inferiority not shown	0.549
[CoP] Corail Pinnacle	1,874	1.58	1.06	[0.56 , 1.57]	Inferior by ≥100%	<0.001
[CoP] Corail Trilogy	327	1.09	0.57	[-0.55 , 1.70]	Non-inferiority not shown	0.317
[CoP] Exeter V40 Elite Plus Ogee	397	1.31	0.79	[-0.20 , 1.77]	Non-inferiority not shown	0.117
[CoP] Exeter V40 Exeter Contemporary Flanged	964	1.38	0.86	[0.20 , 1.52]	Inferior by ≥20%	0.011
[CoP] Exeter V40 Exeter Contemporary Hooded	290	1.82	1.30	[-0.01 , 2.60]	Non-inferiority not shown	0.051
[CoP] Exeter V40 Exeter Duration	325	0.98	0.46	[-0.56 , 1.49]	Non-inferiority not shown	0.377
[CoP] Exeter V40 Trident	999	1.37	0.86	[0.28 , 1.43]	Inferior by ≥20%	0.003
[CoP] Exeter V40 Trilogy	748	0.72	0.20	[-0.44 , 0.84]	Non-inferiority not shown	0.540
[CoP] Furlong HAC Stem CSF	2,437	1.82	1.30	[0.69 , 1.90]	Inferior by ≥100%	<0.001
[CoP] Furlong HAC Stem Furlong HAC CSF Plus	397	2.25	1.73	[0.63 , 2.83]	Inferior by ≥100%	0.002
[CoP] MS-30 Original ME Muller Low Profile Cup	576	0.72	0.21	[-0.48 , 0.89]	Non-inferiority not shown	0.557
[CoP] SL-Plus Cementless Stem EP-Fit Plus	317	3.48	2.96	[1.07 , 4.86]	Inferior by ≥100%	0.002
[CoP] Taperloc Cementless Stem Exceed ABT	355	1.36	0.84	[0.06 , 1.61]	Non-inferiority not shown	0.035
[MoP] Accolade Trident	2,455	2.47	1.95	[1.34 , 2.56]	Inferior by ≥100%	<0.001
[MoP] C-Stem AMT Cemented Stem Charnley and Elite Plus LPW	556	1.17	0.65	[-0.16 , 1.46]	Non-inferiority not shown	0.117
[MoP] C-Stem AMT Cemented Stem Elite Plus Ogee	319	1.54	1.02	[-0.14 , 2.18]	Non-inferiority not shown	0.084
[MoP] C-Stem AMT Cemented Stem Pinnacle	308	1.68	1.16	[0.17 , 2.14]	Inferior by ≥20%	0.021
[MoP] C-Stem Cemented Stem Charnley Ogee	335	2.64	2.12	[0.53 , 3.71]	Inferior by ≥100%	0.009
[MoP] C-Stem Cemented Stem Charnley and Elite Plus LPW	671	1.88	1.36	[0.32 , 2.41]	Inferior by ≥20%	0.010
[MoP] C-Stem Cemented Stem Duraloc Cementless Cup	285	2.32	1.80	[0.06 , 3.54]	Non-inferiority not shown	0.042
[MoP] C-Stem Cemented Stem Elite Plus Ogee	906	1.40	0.88	[0.12 , 1.63]	Inferior by ≥20%	0.023
[MoP] C-Stem Cemented Stem Marathon	330	0.67	0.15	[-0.46 , 0.76]	Non-inferiority not shown	0.633
[MoP] C-Stem Cemented Stem Opera	407	0.90	0.38	[-0.48 , 1.24]	Non-inferiority not shown	0.390
[MoP] C-Stem Cemented Stem Wroblewski Golf Ball	324	1.57	1.05	[-0.25 , 2.36]	Non-inferiority not shown	0.113
[MoP] CCA Cemented Stem CCB Cup	327	0.61	0.09	[-0.70 , 0.88]	Non-inferiority not shown	0.827
[MoP] CPCS Opera	367	2.09	1.57	[0.24 , 2.91]	Inferior by ≥20%	0.021
[MoP] CPT Elite Plus Ogee	691	1.65	1.13	[0.25 , 2.01]	Inferior by ≥20%	0.012
[MoP] CPT Pinnacle	254	1.40	0.88	[-0.53 , 2.29]	Non-inferiority not shown	0.223
[MoP] CPT Trilogy	2,468	2.22	1.70	[1.10 , 2.30]	Inferior by ≥100%	<0.001
[MoP] CPT ZCA	1,787	2.02	1.50	[0.84 , 2.15]	Inferior by ≥100%	<0.001
[MoP] Charnley Cemented Stem Charnley Cemented Cup	1,420	1.52	1.00	[0.30 , 1.70]	Inferior by ≥20%	0.005
[MoP] Charnley Cemented Stem Charnley Ogee	2,959	1.84	1.32	[0.74 , 1.90]	Inferior by ≥100%	<0.001
[MoP] Charnley Cemented Stem Charnley and Elite Plus LPW	2,049	1.18	0.67	[0.09 , 1.24]	Non-inferiority not shown	0.023
[MoP] Charnley Cemented Stem Opera	454	1.22	0.70	[-0.34 , 1.73]	Non-inferiority not shown	0.185
[MoP] Charnley Cemented Stem Wroblewski Golf Ball	277	1.77	1.26	[-0.20 , 2.71]	Non-inferiority not shown	0.091
[MoP] Corail Duraloc Cementless Cup	1,465	2.31	1.79	[0.96 , 2.62]	Inferior by ≥100%	<0.001
[MoP] Corail Elite Plus Cemented Cup	277	0.55	0.03	[-0.81 , 0.87]	Non-inferiority not shown	0.947
[MoP] Corail Elite Plus Ogee	418	1.62	1.10	[0.03 , 2.17]	Non-inferiority not shown	0.044

For peer review only

[MoP] Corail Marathon	586	0.96	0.44	[-0.16 , 1.05]	Non-inferiority not shown	0.153
[MoP] Corail Pinnacle	7,089	1.41	0.89	[0.49 , 1.30]	Inferior by ≥20%	<0.001
[MoP] Corail Trilogy	561	0.94	0.42	[-0.36 , 1.20]	Non-inferiority not shown	0.293
[MoP] Exeter V40 ABG II Cementless Cup	283	1.01	0.49	[-0.70 , 1.67]	Non-inferiority not shown	0.422
[MoP] Exeter V40 Cenator Cemented Cup	611	2.67	2.15	[0.94 , 3.37]	Inferior by ≥100%	<0.001
[MoP] Exeter V40 Charnley Ogee	568	1.39	0.87	[-0.06 , 1.79]	Non-inferiority not shown	0.068
[MoP] Exeter V40 Charnley and Elite Plus LPW	718	1.72	1.20	[0.34 , 2.05]	Inferior by ≥20%	0.006
[MoP] Exeter V40 Duraloc Cementless Cup	488	1.37	0.85	[-0.22 , 1.93]	Non-inferiority not shown	0.119
[MoP] Exeter V40 EP-Fit Plus	250	3.01	2.49	[0.40 , 4.57]	Inferior by ≥20%	0.019
[MoP] Exeter V40 Elite Plus Ogee	4,543	1.18	0.66	[0.21 , 1.12]	Inferior by ≥20%	0.004
[MoP] Exeter V40 Exeter Contemporary Flanged	10,220	1.16	0.64	[0.25 , 1.04]	Inferior by ≥20%	0.001
[MoP] Exeter V40 Exeter Contemporary Hooded	4,277	2.45	1.93	[1.40 , 2.46]	Inferior by ≥100%	<0.001
[MoP] Exeter V40 Exeter Duration	4,048	1.48	0.96	[0.47 , 1.44]	Inferior by ≥20%	<0.001
[MoP] Exeter V40 Exeter X3 Rimfit	319	0.71	0.19	[-0.25 , 0.63]	Non-inferiority not shown	0.395
[MoP] Exeter V40 Opera	655	1.22	0.70	[-0.10 , 1.50]	Non-inferiority not shown	0.088
[MoP] Exeter V40 Pinnacle	658	1.40	0.88	[0.12 , 1.64]	Inferior by ≥20%	0.023
[MoP] Exeter V40 Reflection Cementless	732	1.14	0.62	[-0.20 , 1.44]	Non-inferiority not shown	0.141
[MoP] Exeter V40 Trident	4,016	1.44	0.92	[0.47 , 1.36]	Inferior by ≥20%	<0.001
[MoP] Exeter V40 Trilogy	2,821	1.20	0.68	[0.17 , 1.18]	Inferior by ≥20%	0.009
[MoP] Exeter V40 Ultima Cemented Cup	408	2.56	2.04	[0.51 , 3.58]	Inferior by ≥20%	0.009
[MoP] Furlong Cemented Stem JRI Cemented Cup	458	2.52	2.00	[0.60 , 3.41]	Inferior by ≥100%	0.005
[MoP] Furlong HAC Stem CSF	2,056	2.08	1.56	[0.91 , 2.22]	Inferior by ≥100%	<0.001
[MoP] Furlong HAC Stem Furlong HAC CSF Plus	633	2.82	2.30	[1.26 , 3.34]	Inferior by ≥100%	<0.001
[MoP] Muller Straight Stem Original ME Muller Low Profile Cup	395	1.47	0.95	[-0.08 , 1.98]	Non-inferiority not shown	0.071
[MoP] Muller-Biomet Apollo	535	2.11	1.59	[0.44 , 2.74]	Inferior by ≥20%	0.007
[MoP] Omnifit Cemented Stem ODC	340	2.48	1.96	[0.32 , 3.60]	Inferior by ≥20%	0.019
[MoP] SL-Plus Cementless Stem EP-Fit Plus	739	3.85	3.33	[2.07 , 4.59]	Inferior by ≥100%	<0.001
[MoP] Stanmore Modular Stem Stanmore-Arcom Cup	1,106	2.20	1.68	[0.83 , 2.53]	Inferior by ≥100%	<0.001
[MoP] Synergy Cementless Stem Reflection Cementless	577	1.15	0.63	[-0.29 , 1.55]	Non-inferiority not shown	0.179
[MoP] Taperloc Cementless Stem Exceed ABT	761	2.49	1.97	[1.18 , 2.77]	Inferior by ≥100%	<0.001
[MoP] Versys Cementless Stem Trilogy	342	4.74	4.22	[2.05 , 6.38]	Inferior by ≥100%	<0.001

For peer review only

Supplemental table 4c: Difference in Kaplan-Meier failure estimate between a contemporary reference and implants with at least 250 at risk at 7 years since primary in females between 55 and 75 years

Stem/cup brand	Number at risk	Cumulative failure (%)	Difference in failure (%)	95% CI	Equivalence status	p-value
[MoP] Exeter V40 Trilogy	1,962	1.45	[REFERENCE]			
[CoC] ABG II Monolithic Cementless Stem ABG II Cementless Cup	335	2.11	0.66	[-0.84 , 2.16]	Non-inferiority not shown	0.389
[CoC] Accolade Trident	1,256	3.07	1.62	[0.81 , 2.44]	Inferior by ≥20%	<0.001
[CoC] Corail Duraloc Option	301	3.15	1.70	[-0.11 , 3.50]	Non-inferiority not shown	0.065
[CoC] Corail Pinnacle	2,917	2.20	0.76	[0.25 , 1.26]	Non-inferiority not shown	0.004
[CoC] Exeter V40 ABG II Cementless Cup	454	1.65	0.20	[-0.90 , 1.31]	Non-inferiority not shown	0.721
[CoC] Exeter V40 Trident	2,340	1.76	0.31	[-0.28 , 0.90]	Non-inferiority not shown	0.300
[CoC] Furlong HAC Stem CSF	549	2.90	1.45	[0.07 , 2.83]	Non-inferiority not shown	0.040
[CoC] Furlong HAC Stem Furlong HAC CSF Plus	830	2.23	0.78	[0.12 , 1.44]	Non-inferiority not shown	0.021
[CoC] SL-Plus Cementless Stem EP-Fit Plus	295	3.46	2.01	[0.11 , 3.90]	Non-inferiority not shown	0.038
[CoC] Taperloc Cementless Stem Exceed ABT	712	1.81	0.36	[-0.29 , 1.01]	Non-inferiority not shown	0.279
[CoP] Accolade Trident	257	2.02	0.57	[-0.29 , 1.43]	Non-inferiority not shown	0.195
[CoP] Corail Pinnacle	771	1.86	0.41	[-0.20 , 1.02]	Non-inferiority not shown	0.183
[CoP] Corail Trilogy	258	1.09	-0.35	[-1.50 , 0.79]	Non-inferiority not shown	0.543
[CoP] Exeter V40 Elite Plus Ogee	251	1.59	0.15	[-1.00 , 1.29]	Non-inferiority not shown	0.804
[CoP] Exeter V40 Exeter Contemporary Flanged	563	1.38	-0.07	[-0.76 , 0.61]	Non-inferiority not shown	0.839
[CoP] Exeter V40 Exeter Duration	255	1.30	-0.14	[-1.36 , 1.07]	Non-inferiority not shown	0.818
[CoP] Exeter V40 Trident	463	1.63	0.19	[-0.52 , 0.89]	Non-inferiority not shown	0.606
[CoP] Exeter V40 Trilogy	561	1.02	-0.43	[-1.22 , 0.35]	Non-inferiority not shown	0.282
[CoP] Furlong HAC Stem CSF	2,033	2.34	0.90	[0.20 , 1.60]	Non-inferiority not shown	0.012
[CoP] MS-30 Original ME Muller Low Profile Cup	374	0.72	-0.72	[-1.43 , -0.01]	Non-inferior	0.046
[MoP] Accolade Trident	1,158	3.23	1.79	[1.03 , 2.54]	Inferior by ≥20%	<0.001
[MoP] C-Stem AMT Cemented Stem Charnley and Elite Plus LPW	344	1.72	0.28	[-0.85 , 1.41]	Non-inferiority not shown	0.632
[MoP] C-Stem Cemented Stem Charnley Ogee	284	2.97	1.52	[-0.20 , 3.24]	Non-inferiority not shown	0.084
[MoP] C-Stem Cemented Stem Charnley and Elite Plus LPW	617	2.66	1.21	[-0.04 , 2.46]	Non-inferiority not shown	0.058
[MoP] C-Stem Cemented Stem Elite Plus Ogee	733	2.13	0.68	[-0.29 , 1.65]	Non-inferiority not shown	0.170
[MoP] C-Stem Cemented Stem Opera	311	1.69	0.24	[-1.01 , 1.49]	Non-inferiority not shown	0.709
[MoP] CPT Elite Plus Ogee	470	2.58	1.13	[-0.08 , 2.34]	Non-inferiority not shown	0.067
[MoP] CPT Trilogy	1,661	2.95	1.50	[0.77 , 2.23]	Inferior by ≥20%	<0.001
[MoP] CPT ZCA	1,254	2.39	0.94	[0.20 , 1.68]	Non-inferiority not shown	0.013
[MoP] Charnley Cemented Stem Charnley Cemented Cup	1,220	2.10	0.65	[-0.18 , 1.48]	Non-inferiority not shown	0.125
[MoP] Charnley Cemented Stem Charnley Ogee	2,422	2.20	0.76	[0.11 , 1.40]	Non-inferiority not shown	0.022
[MoP] Charnley Cemented Stem Charnley and Elite Plus LPW	1,746	1.64	0.20	[-0.48 , 0.87]	Non-inferiority not shown	0.569
[MoP] Charnley Cemented Stem Opera	345	2.16	0.71	[-0.68 , 2.10]	Non-inferiority not shown	0.316
[MoP] Corail Duraloc Cementless Cup	1,258	3.66	2.21	[1.18 , 3.25]	Inferior by ≥20%	<0.001
[MoP] Corail Elite Plus Ogee	292	1.93	0.48	[-0.76 , 1.72]	Non-inferiority not shown	0.449
[MoP] Corail Pinnacle	3,659	1.82	0.38	[-0.10 , 0.86]	Non-inferiority not shown	0.123
[MoP] Corail Trilogy	369	1.65	0.20	[-0.93 , 1.34]	Non-inferiority not shown	0.725
[MoP] Exeter V40 ABG II Cementless Cup	272	1.01	-0.44	[-1.65 , 0.76]	Non-inferiority not shown	0.472

For peer review only - http://bmjopen.bmj.com/site/about/guidelines.xhtml

[MoP] Exeter V40 Cenator Cemented Cup	478	2.86	1.41	[0.13 , 2.69]	Non-inferiority not shown	0.030
[MoP] Exeter V40 Charnley Ogee	467	1.39	-0.06	[-1.01 , 0.89]	Non-inferiority not shown	0.897
[MoP] Exeter V40 Charnley and Elite Plus LPW	470	2.39	0.94	[-0.15 , 2.03]	Non-inferiority not shown	0.091
[MoP] Exeter V40 Duraloc Cementless Cup	392	1.79	0.34	[-0.89 , 1.56]	Non-inferiority not shown	0.589
[MoP] Exeter V40 Elite Plus Cemented Cup	701	0.63	-0.82	[-1.40 , -0.23]	Non-inferior	0.006
[MoP] Exeter V40 Elite Plus Ogee	3,436	1.59	0.14	[-0.38 , 0.67]	Non-inferiority not shown	0.593
[MoP] Exeter V40 Exeter Contemporary Flanged	6,755	1.51	0.06	[-0.39 , 0.52]	Non-inferiority not shown	0.788
[MoP] Exeter V40 Exeter Contemporary Hooded	2,891	3.20	1.75	[1.12 , 2.38]	Inferior by ≥20%	<0.001
[MoP] Exeter V40 Exeter Duration	3,049	2.17	0.72	[0.14 , 1.31]	Non-inferiority not shown	0.016
[MoP] Exeter V40 Opera	462	1.54	0.09	[-0.84 , 1.02]	Non-inferiority not shown	0.850
[MoP] Exeter V40 Pinnacle	346	1.56	0.11	[-0.73 , 0.95]	Non-inferiority not shown	0.797
[MoP] Exeter V40 Reflection Cementless	591	2.01	0.56	[-0.53 , 1.65]	Non-inferiority not shown	0.312
[MoP] Exeter V40 Trident	2,291	2.01	0.56	[0.01 , 1.11]	Non-inferiority not shown	0.046
[MoP] Exeter V40 Ultima Cemented Cup	379	2.80	1.36	[-0.26 , 2.97]	Non-inferiority not shown	0.100
[MoP] Furlong Cemented Stem JRI Cemented Cup	416	2.97	1.52	[-0.02 , 3.05]	Non-inferiority not shown	0.053
[MoP] Furlong HAC Stem CSF	1,634	2.65	1.20	[0.44 , 1.96]	Inferior by ≥20%	0.002
[MoP] Furlong HAC Stem Furlong HAC CSF Plus	270	3.32	1.87	[0.67 , 3.06]	Inferior by ≥20%	0.002
[MoP] Muller Straight Stem Original ME Muller Low Profile Cup	264	2.38	0.93	[-0.53 , 2.40]	Non-inferiority not shown	0.211
[MoP] Muller-Biomet Apollo	414	2.72	1.27	[-0.08 , 2.62]	Non-inferiority not shown	0.065
[MoP] Omnifit Cemented Stem ODC	309	3.99	2.54	[0.45 , 4.63]	Inferior by ≥20%	0.017
[MoP] SL-Plus Cementless Stem EP-Fit Plus	586	4.42	2.97	[1.59 , 4.36]	Inferior by ≥100%	<0.001
[MoP] Stanmore Modular Stem Stanmore-Arcom Cup	784	2.59	1.14	[0.19 , 2.09]	Non-inferiority not shown	0.018
[MoP] Synergy Cementless Stem Reflection Cementless	432	1.74	0.29	[-0.86 , 1.44]	Non-inferiority not shown	0.617
[MoP] Versys Cementless Stem Trilogy	318	4.74	3.29	[1.11 , 5.46]	Inferior by ≥20%	0.003

For peer review only

Supplemental table 4d: Difference in Kaplan-Meier failure estimate between a contemporary reference and implants with at least 250 at risk at 10 years since primary in females between 55 and 75 years

Stem/cup brand	Number at risk	Cumulative failure (%)	Difference in failure (%)	95% CI	Equivalence status	p-value
[MoP] Exeter V40 Elite Plus Ogee	1,609	2.16	[REFERENCE]			
[CoC] ABG II Monolithic Cementless Stem ABG II Cementless Cup	259	2.44	0.28	[-1.36 , 1.92]	Non-inferiority not shown	0.739
[CoC] Corail Pinnacle	342	2.97	0.81	[0.10 , 1.52]	Non-inferiority not shown	0.025
[CoC] Exeter V40 Trident	727	2.29	0.13	[-0.60 , 0.85]	Non-inferiority not shown	0.731
[CoC] Furlong HAC Stem CSF	322	3.61	1.45	[-0.16 , 3.06]	Non-inferiority not shown	0.077
[CoP] Exeter V40 Trilogy	250	1.52	-0.64	[-1.72 , 0.43]	Non-inferiority not shown	0.243
[CoP] Furlong HAC Stem CSF	1,151	3.18	1.02	[0.17 , 1.88]	Non-inferiority not shown	0.019
[MoP] C-Stem Cemented Stem Charnley and Elite Plus LPW	376	3.32	1.16	[-0.26 , 2.57]	Non-inferiority not shown	0.108
[MoP] C-Stem Cemented Stem Elite Plus Ogee	373	2.97	0.81	[-0.42 , 2.04]	Non-inferiority not shown	0.198
[MoP] CPT Trilogy	562	4.25	2.09	[1.06 , 3.12]	Inferior by ≥20%	<0.001
[MoP] CPT ZCA	520	3.72	1.56	[0.48 , 2.64]	Inferior by ≥20%	0.005
[MoP] Charnley Cemented Stem Charnley Cemented Cup	782	3.09	0.93	[-0.10 , 1.96]	Non-inferiority not shown	0.076
[MoP] Charnley Cemented Stem Charnley Ogee	1,368	3.80	1.64	[0.77 , 2.51]	Inferior by ≥20%	<0.001
[MoP] Charnley Cemented Stem Charnley and Elite Plus LPW	1,079	2.64	0.48	[-0.38 , 1.34]	Non-inferiority not shown	0.275
[MoP] Corail Duraloc Cementless Cup	598	5.19	3.03	[1.73 , 4.32]	Inferior by ≥20%	<0.001
[MoP] Corail Pinnacle	849	2.37	0.21	[-0.43 , 0.84]	Non-inferiority not shown	0.521
[MoP] Exeter V40 Cenator Cemented Cup	312	3.32	1.16	[-0.28 , 2.59]	Non-inferiority not shown	0.114
[MoP] Exeter V40 Charnley Ogee	312	1.39	-0.77	[-1.74 , 0.19]	Non-inferior	0.116
[MoP] Exeter V40 Duraloc Cementless Cup	257	3.96	1.80	[-0.21 , 3.82]	Non-inferiority not shown	0.079
[MoP] Exeter V40 Elite Plus Cemented Cup	305	0.89	-1.27	[-2.07 , -0.48]	Non-inferior	0.002
[MoP] Exeter V40 Exeter Contemporary Flanged	2,152	2.29	0.13	[-0.43 , 0.70]	Non-inferiority not shown	0.644
[MoP] Exeter V40 Exeter Contemporary Hooded	1,110	4.86	2.70	[1.83 , 3.56]	Inferior by ≥20%	<0.001
[MoP] Exeter V40 Exeter Duration	1,344	3.44	1.28	[0.51 , 2.05]	Inferior by ≥20%	0.001
[MoP] Exeter V40 Trident	523	3.05	0.89	[0.06 , 1.72]	Non-inferiority not shown	0.035
[MoP] Exeter V40 Trilogy	748	2.13	-0.03	[-0.78 , 0.72]	Non-inferiority not shown	0.931
[MoP] Furlong Cemented Stem JRI Cemented Cup	268	4.14	1.98	[0.07 , 3.89]	Non-inferiority not shown	0.043
[MoP] Furlong HAC Stem CSF	726	3.72	1.56	[0.60 , 2.51]	Inferior by ≥20%	0.001
[MoP] Stanmore Modular Stem Stanmore-Arcom Cup	329	3.31	1.15	[-0.00 , 2.30]	Non-inferiority not shown	0.050

For peer review only

Supplemental table 5a: Difference in Kaplan-Meier failure estimate between a contemporary reference and implants with at least 250 at risk at 3 years since primary in females >75 years

Stem/cup brand	Number at risk	Cumulative failure (%)	Difference in failure (%)	95% CI	Equivalence status	p-value
[MoP] Charnley Cemented Stem Charnley and Elite Plus LPW	1,794	0.55	[REFERENCE]			
[CoC] Accolade Trident	251	2.21	1.66	[-0.12 , 3.44]	Non-inferiority not shown	0.067
[CoC] Corail Pinnacle	757	2.22	1.67	[0.67 , 2.66]	Inferior by ≥100%	0.001
[CoC] Furlong HAC Stem Furlong HAC CSF Plus	540	0.68	0.13	[-0.55 , 0.81]	Non-inferiority not shown	0.700
[CoP] Corail Pinnacle	431	0.69	0.14	[-0.48 , 0.76]	Non-inferiority not shown	0.653
[CoP] Exeter V40 Trident	424	0.34	-0.21	[-0.72 , 0.30]	Non-inferiority not shown	0.417
[CoP] Furlong HAC Stem CSF	693	1.24	0.69	[-0.14 , 1.52]	Non-inferiority not shown	0.103
[CoP] MS-30 Original ME Muller Low Profile Cup	300	0.00	- -	[-.-- , -.--]	No failures to date	
[MoP] Accolade Trident	1,623	1.59	1.04	[0.40 , 1.69]	Inferior by ≥20%	0.002
[MoP] C-Stem AMT Cemented Stem Charnley and Elite Plus LPW	646	1.09	0.54	[-0.24 , 1.32]	Non-inferiority not shown	0.176
[MoP] C-Stem AMT Cemented Stem Elite Plus Ogee	646	0.88	0.33	[-0.37 , 1.03]	Non-inferiority not shown	0.354
[MoP] C-Stem AMT Cemented Stem Marathon	343	0.68	0.13	[-0.52 , 0.78]	Non-inferiority not shown	0.691
[MoP] C-Stem AMT Cemented Stem Pinnacle	369	1.48	0.93	[0.12 , 1.74]	Inferior by ≥20%	0.024
[MoP] C-Stem Cemented Stem Charnley and Elite Plus LPW	384	1.43	0.88	[-0.30 , 2.06]	Non-inferiority not shown	0.145
[MoP] C-Stem Cemented Stem Elite Plus Ogee	808	0.43	-0.12	[-0.65 , 0.42]	Non-inferiority not shown	0.672
[MoP] C-Stem Cemented Stem Marathon	333	0.58	0.03	[-0.63 , 0.69]	Non-inferiority not shown	0.928
[MoP] C-Stem Cemented Stem Opera	270	0.34	-0.21	[-0.95 , 0.52]	Non-inferiority not shown	0.568
[MoP] CCA Cemented Stem CCB Cup	329	1.00	0.45	[-0.59 , 1.49]	Non-inferiority not shown	0.394
[MoP] CPCS Opera	309	0.61	0.06	[-0.84 , 0.95]	Non-inferiority not shown	0.904
[MoP] CPT Elite Plus Ogee	585	1.11	0.56	[-0.27 , 1.40]	Non-inferiority not shown	0.186
[MoP] CPT Original ME Muller Low Profile Cup	272	1.78	1.23	[-0.25 , 2.70]	Non-inferiority not shown	0.104
[MoP] CPT Trabecular Metal Modular Cementless Cup	306	0.92	0.37	[-0.60 , 1.34]	Non-inferiority not shown	0.451
[MoP] CPT Trilogy	2,024	1.12	0.57	[0.07 , 1.08]	Non-inferiority not shown	0.027
[MoP] CPT ZCA	3,039	1.37	0.82	[0.34 , 1.30]	Inferior by ≥20%	0.001
[MoP] Charnley Cemented Stem Charnley Cemented Cup	926	0.92	0.37	[-0.31 , 1.05]	Non-inferiority not shown	0.289
[MoP] Charnley Cemented Stem Charnley Ogee	2,041	0.79	0.24	[-0.25 , 0.72]	Non-inferiority not shown	0.344
[MoP] Charnley Cemented Stem Opera	306	0.93	0.38	[-0.72 , 1.49]	Non-inferiority not shown	0.494
[MoP] Charnley Cemented Stem Wroblewski Golf Ball	275	1.66	1.11	[-0.37 , 2.59]	Non-inferiority not shown	0.142
[MoP] Corail Duraloc Cementless Cup	535	0.68	0.13	[-0.61 , 0.87]	Non-inferiority not shown	0.727
[MoP] Corail Elite Plus Cemented Cup	272	0.65	0.10	[-0.86 , 1.06]	Non-inferiority not shown	0.841
[MoP] Corail Elite Plus Ogee	376	1.19	0.64	[-0.36 , 1.64]	Non-inferiority not shown	0.211
[MoP] Corail Marathon	690	0.92	0.37	[-0.28 , 1.01]	Non-inferiority not shown	0.264
[MoP] Corail Pinnacle	4,912	1.43	0.88	[0.46 , 1.30]	Inferior by ≥20%	<0.001
[MoP] Corail Trilogy	336	0.77	0.22	[-0.71 , 1.15]	Non-inferiority not shown	0.642
[MoP] Exeter V40 Cenator Cemented Cup	694	0.99	0.44	[-0.32 , 1.20]	Non-inferiority not shown	0.256
[MoP] Exeter V40 Charnley Ogee	321	0.59	0.04	[-0.84 , 0.92]	Non-inferiority not shown	0.930
[MoP] Exeter V40 Charnley and Elite Plus LPW	847	1.31	0.76	[-0.02 , 1.54]	Non-inferiority not shown	0.057
[MoP] Exeter V40 Elite Plus Cemented Cup	906	0.42	-0.14	[-0.62 , 0.35]	Non-inferiority not shown	0.587
[MoP] Exeter V40 Elite Plus Ogee	5,144	0.61	0.06	[-0.32 , 0.43]	Non-inferiority not shown	0.772

For peer review only

[MoP] Exeter V40 Exeter Contemporary Flanged	12,084	0.59	0.04	[-0.31 , 0.39]	Non-inferiority not shown	0.821
[MoP] Exeter V40 Exeter Contemporary Hooded	5,022	1.11	0.56	[0.15 , 0.97]	Inferior by ≥20%	0.007
[MoP] Exeter V40 Exeter Duration	3,745	0.79	0.24	[-0.18 , 0.65]	Non-inferiority not shown	0.265
[MoP] Exeter V40 Exeter X3 Rimfit	1,286	0.68	0.13	[-0.31 , 0.57]	Non-inferiority not shown	0.567
[MoP] Exeter V40 Marathon	365	0.95	0.40	[-0.38 , 1.18]	Non-inferiority not shown	0.319
[MoP] Exeter V40 Opera	732	0.48	-0.07	[-0.64 , 0.50]	Non-inferiority not shown	0.815
[MoP] Exeter V40 Pinnacle	736	0.83	0.28	[-0.31 , 0.87]	Non-inferiority not shown	0.358
[MoP] Exeter V40 Reflection Cementless	494	0.75	0.20	[-0.60 , 1.00]	Non-inferiority not shown	0.623
[MoP] Exeter V40 Trident	4,508	0.77	0.22	[-0.16 , 0.60]	Non-inferiority not shown	0.261
[MoP] Exeter V40 Trilogy	1,511	0.87	0.32	[-0.21 , 0.86]	Non-inferiority not shown	0.240
[MoP] Exeter V40 Ultima Cemented Cup	337	0.82	0.27	[-0.71 , 1.25]	Non-inferiority not shown	0.591
[MoP] Furlong Cemented Stem JRI Cemented Cup	531	0.88	0.33	[-0.51 , 1.16]	Non-inferiority not shown	0.442
[MoP] Furlong HAC Stem CSF	1,553	2.48	1.93	[1.15 , 2.70]	Inferior by ≥100%	<0.001
[MoP] Furlong HAC Stem Furlong HAC CSF Plus	792	2.58	2.03	[1.08 , 2.97]	Inferior by ≥100%	<0.001
[MoP] MS-30 Original ME Muller Low Profile Cup	401	0.36	-0.20	[-0.78 , 0.39]	Non-inferiority not shown	0.516
[MoP] Muller Straight Stem Original ME Muller Low Profile Cup	461	0.78	0.23	[-0.53 , 0.99]	Non-inferiority not shown	0.550
[MoP] Muller-Biomet Apollo	551	0.66	0.11	[-0.61 , 0.83]	Non-inferiority not shown	0.763
[MoP] Muller-Biomet Original ME Muller Low Profile Cup	342	1.08	0.53	[-0.57 , 1.63]	Non-inferiority not shown	0.346
[MoP] Omnifit Cemented Stem ODC	253	1.47	0.92	[-0.55 , 2.39]	Non-inferiority not shown	0.218
[MoP] SL-Plus Cementless Stem EP-Fit Plus	290	0.71	0.16	[-0.70 , 1.02]	Non-inferiority not shown	0.720
[MoP] Stanmore Modular Stem SHP Cup	367	0.64	0.09	[-0.70 , 0.89]	Non-inferiority not shown	0.819
[MoP] Stanmore Modular Stem Stanmore-Arcom Cup	1,325	0.86	0.31	[-0.26 , 0.87]	Non-inferiority not shown	0.289
[MoP] Taperloc Cementless Stem Exceed ABT	733	1.73	1.18	[0.37 , 1.99]	Inferior by ≥20%	0.004

For peer review only

Supplemental table 5b: Difference in Kaplan-Meier failure estimate between a contemporary reference and implants with at least 250 at risk at 5 years since primary in females >75 years

Stem/cup brand	Number at risk	Cumulative failure (%)	Difference in failure (%)	95% CI	Equivalence status	p-value
[MoP] Charnley Cemented Stem Charnley and Elite Plus LPW	1,515	0.73	[REFERENCE]			
[CoC] Corail Pinnacle	454	2.22	1.49	[0.47 , 2.50]	Inferior by ≥20%	0.004
[CoC] Furlong HAC Stem Furlong HAC CSF Plus	302	1.22	0.49	[-0.55 , 1.53]	Non-inferiority not shown	0.352
[CoP] Furlong HAC Stem CSF	539	1.56	0.83	[-0.13 , 1.80]	Non-inferiority not shown	0.089
[MoP] Accolade Trident	1,066	2.01	1.28	[0.53 , 2.03]	Inferior by ≥20%	0.001
[MoP] C-Stem AMT Cemented Stem Charnley and Elite Plus LPW	388	1.25	0.52	[-0.34 , 1.39]	Non-inferiority not shown	0.236
[MoP] C-Stem AMT Cemented Stem Elite Plus Ogee	345	0.88	0.15	[-0.57 , 0.88]	Non-inferiority not shown	0.682
[MoP] C-Stem Cemented Stem Charnley and Elite Plus LPW	328	1.73	1.00	[-0.33 , 2.33]	Non-inferiority not shown	0.141
[MoP] C-Stem Cemented Stem Elite Plus Ogee	614	0.57	-0.16	[-0.79 , 0.47]	Non-inferiority not shown	0.615
[MoP] CPT Elite Plus Ogee	388	1.49	0.76	[-0.24 , 1.77]	Non-inferiority not shown	0.136
[MoP] CPT Trilogy	1,273	1.88	1.15	[0.47 , 1.83]	Inferior by ≥20%	0.001
[MoP] CPT ZCA	2,186	1.86	1.13	[0.55 , 1.71]	Inferior by ≥20%	<0.001
[MoP] Charnley Cemented Stem Charnley Cemented Cup	780	1.27	0.54	[-0.27 , 1.36]	Non-inferiority not shown	0.190
[MoP] Charnley Cemented Stem Charnley Ogee	1,658	1.15	0.42	[-0.17 , 1.01]	Non-inferiority not shown	0.162
[MoP] Corail Duraloc Cementless Cup	479	1.66	0.93	[-0.21 , 2.07]	Non-inferiority not shown	0.111
[MoP] Corail Marathon	325	0.92	0.19	[-0.49 , 0.86]	Non-inferiority not shown	0.585
[MoP] Corail Pinnacle	2,638	1.78	1.05	[0.55 , 1.55]	Inferior by ≥20%	<0.001
[MoP] Corail Trilogy	261	1.11	0.39	[-0.78 , 1.55]	Non-inferiority not shown	0.515
[MoP] Exeter V40 Cenator Cemented Cup	533	1.32	0.59	[-0.31 , 1.49]	Non-inferiority not shown	0.201
[MoP] Exeter V40 Charnley Ogee	264	1.27	0.54	[-0.76 , 1.85]	Non-inferiority not shown	0.414
[MoP] Exeter V40 Charnley and Elite Plus LPW	575	1.31	0.58	[-0.23 , 1.39]	Non-inferiority not shown	0.159
[MoP] Exeter V40 Elite Plus Cemented Cup	653	0.53	-0.20	[-0.77 , 0.38]	Non-inferiority not shown	0.502
[MoP] Exeter V40 Elite Plus Ogee	3,667	0.84	0.11	[-0.34 , 0.56]	Non-inferiority not shown	0.644
[MoP] Exeter V40 Exeter Contemporary Flanged	7,444	0.86	0.13	[-0.28 , 0.55]	Non-inferiority not shown	0.524
[MoP] Exeter V40 Exeter Contemporary Hooded	3,318	1.47	0.74	[0.24 , 1.23]	Inferior by ≥20%	0.003
[MoP] Exeter V40 Exeter Duration	2,928	1.18	0.46	[-0.05 , 0.96]	Non-inferiority not shown	0.078
[MoP] Exeter V40 Opera	530	0.63	-0.10	[-0.77 , 0.57]	Non-inferiority not shown	0.768
[MoP] Exeter V40 Pinnacle	386	1.03	0.30	[-0.44 , 1.04]	Non-inferiority not shown	0.422
[MoP] Exeter V40 Reflection Cementless	402	0.96	0.23	[-0.69 , 1.16]	Non-inferiority not shown	0.620
[MoP] Exeter V40 Trident	2,517	1.05	0.32	[-0.15 , 0.79]	Non-inferiority not shown	0.179
[MoP] Exeter V40 Trilogy	1,080	1.32	0.59	[-0.08 , 1.27]	Non-inferiority not shown	0.085
[MoP] Exeter V40 Ultima Cemented Cup	300	1.15	0.42	[-0.77 , 1.61]	Non-inferiority not shown	0.488
[MoP] Furlong Cemented Stem JRI Cemented Cup	426	0.88	0.15	[-0.71 , 1.00]	Non-inferiority not shown	0.734
[MoP] Furlong HAC Stem CSF	1,201	3.07	2.34	[1.44 , 3.24]	Inferior by ≥100%	<0.001
[MoP] Furlong HAC Stem Furlong HAC CSF Plus	481	2.71	1.98	[0.98 , 2.98]	Inferior by ≥100%	<0.001
[MoP] MS-30 Original ME Muller Low Profile Cup	259	1.30	0.57	[-0.67 , 1.81]	Non-inferiority not shown	0.366
[MoP] Muller Straight Stem Original ME Muller Low Profile Cup	319	1.04	0.31	[-0.62 , 1.25]	Non-inferiority not shown	0.514
[MoP] Muller-Biomet Apollo	422	0.86	0.13	[-0.71 , 0.98]	Non-inferiority not shown	0.759
[MoP] Stanmore Modular Stem Stanmore-Arcom Cup	979	1.12	0.39	[-0.28 , 1.06]	Non-inferiority not shown	0.253

For peer review only

[MoP] Taperloc Cementless Stem Exceed ABT	337	1.95	1.22	[0.29 , 2.15]	Inferior by ≥20%	0.010

For peer review only

Supplemental table 5c: Difference in Kaplan-Meier failure estimate between a contemporary reference and implants with at least 250 at risk at 7 years since primary in females >75 years

Stem/cup brand	Number at risk	Cumulative failure (%)	Difference in failure (%)	95% CI	Equivalence status	p-value
[MoP] Charnley Cemented Stem Charnley and Elite Plus LPW	1,119	0.88	[REFERENCE]			
[CoP] Furlong HAC Stem CSF	391	2.04	1.16	[-0.02 , 2.34]	Non-inferiority not shown	0.055
[MoP] Accolade Trident	460	2.40	1.52	[0.61 , 2.42]	Inferior by ≥20%	0.001
[MoP] C-Stem Cemented Stem Charnley and Elite Plus LPW	292	1.73	0.85	[-0.50 , 2.19]	Non-inferiority not shown	0.218
[MoP] C-Stem Cemented Stem Elite Plus Ogee	444	0.57	-0.31	[-0.98 , 0.35]	Non-inferiority not shown	0.354
[MoP] CPT Trilogy	689	2.22	1.34	[0.53 , 2.16]	Inferior by ≥20%	0.001
[MoP] CPT ZCA	1,341	2.44	1.56	[0.85 , 2.27]	Inferior by ≥20%	<0.001
[MoP] Charnley Cemented Stem Charnley Cemented Cup	605	1.72	0.84	[-0.14 , 1.81]	Non-inferiority not shown	0.093
[MoP] Charnley Cemented Stem Charnley Ogee	1,236	1.35	0.47	[-0.20 , 1.13]	Non-inferiority not shown	0.167
[MoP] Corail Duraloc Cementless Cup	363	2.36	1.48	[0.08 , 2.88]	Non-inferiority not shown	0.039
[MoP] Corail Pinnacle	1,150	2.17	1.28	[0.67 , 1.90]	Inferior by ≥20%	<0.001
[MoP] Exeter V40 Cenator Cemented Cup	373	1.32	0.44	[-0.49 , 1.37]	Non-inferiority not shown	0.355
[MoP] Exeter V40 Charnley and Elite Plus LPW	309	1.31	0.43	[-0.41 , 1.26]	Non-inferiority not shown	0.313
[MoP] Exeter V40 Elite Plus Cemented Cup	429	0.95	0.07	[-0.77 , 0.90]	Non-inferiority not shown	0.879
[MoP] Exeter V40 Elite Plus Ogee	2,329	1.18	0.30	[-0.24 , 0.83]	Non-inferiority not shown	0.283
[MoP] Exeter V40 Exeter Contemporary Flanged	4,187	1.13	0.24	[-0.24 , 0.73]	Non-inferiority not shown	0.320
[MoP] Exeter V40 Exeter Contemporary Hooded	1,866	2.16	1.28	[0.66 , 1.90]	Inferior by ≥20%	<0.001
[MoP] Exeter V40 Exeter Duration	1,961	1.66	0.78	[0.17 , 1.39]	Non-inferiority not shown	0.012
[MoP] Exeter V40 Opera	317	0.85	-0.03	[-0.86 , 0.80]	Non-inferiority not shown	0.950
[MoP] Exeter V40 Reflection Cementless	258	1.34	0.46	[-0.74 , 1.66]	Non-inferiority not shown	0.452
[MoP] Exeter V40 Trident	1,221	1.27	0.39	[-0.17 , 0.95]	Non-inferiority not shown	0.168
[MoP] Exeter V40 Trilogy	618	1.41	0.53	[-0.20 , 1.26]	Non-inferiority not shown	0.151
[MoP] Furlong Cemented Stem JRI Cemented Cup	307	1.11	0.23	[-0.76 , 1.22]	Non-inferiority not shown	0.649
[MoP] Furlong HAC Stem CSF	882	3.51	2.63	[1.63 , 3.63]	Inferior by ≥100%	<0.001
[MoP] Muller-Biomet Apollo	288	0.86	-0.02	[-0.89 , 0.85]	Non-inferiority not shown	0.965
[MoP] Stanmore Modular Stem Stanmore-Arcom Cup	575	1.36	0.48	[-0.29 , 1.26]	Non-inferiority not shown	0.223

For peer review only

Supplemental table 6a: Difference in Kaplan-Meier failure estimate between a contemporary reference and implants with at least 250 at risk at 3 years since primary in males

Stem/cup brand	Number at risk	Cumulative failure (%)	Difference in failure (%)	95% CI	Equivalence status	p-value
[CoP] Exeter V40 Exeter Contemporary Flanged	1,147	0.76	[REFERENCE]			
[CoC] ABG II Monolithic Cementless Stem ABG II Cementless Cup	560	2.22	1.46	[0.19 , 2.73]	Inferior by ≥20%	0.024
[CoC] ABG II Monolithic Cementless Stem Trident	544	1.76	1.00	[-0.16 , 2.17]	Non-inferiority not shown	0.091
[CoC] Accolade Trident	3,001	2.33	1.57	[0.90 , 2.25]	Inferior by ≥100%	<0.001
[CoC] Bimetric Cementless Stem Exceed ABT	377	0.89	0.14	[-0.84 , 1.11]	Non-inferiority not shown	0.784
[CoC] C-Stem AMT Cemented Stem Pinnacle	284	0.43	-0.33	[-1.06 , 0.40]	Non-inferiority not shown	0.380
[CoC] CPT Continuum	321	2.54	1.78	[0.30 , 3.27]	Inferior by ≥20%	0.019
[CoC] Corail Delta TT	274	2.45	1.69	[0.11 , 3.27]	Non-inferiority not shown	0.036
[CoC] Corail DeltaMotion	452	1.67	0.91	[-0.25 , 2.08]	Non-inferiority not shown	0.125
[CoC] Corail Duraloc Option	463	2.29	1.53	[0.13 , 2.94]	Non-inferiority not shown	0.033
[CoC] Corail Pinnacle	12,616	1.92	1.16	[0.68 , 1.64]	Inferior by ≥20%	<0.001
[CoC] Excia Cementless Plasmacup SC	419	0.85	0.09	[-0.77 , 0.95]	Non-inferiority not shown	0.832
[CoC] Exeter V40 ABG II Cementless Cup	457	1.13	0.38	[-0.62 , 1.38]	Non-inferiority not shown	0.460
[CoC] Exeter V40 Trident	4,326	1.13	0.37	[-0.15 , 0.89]	Non-inferiority not shown	0.158
[CoC] Furlong Evolution Cementless Furlong HAC CSF Plus	275	2.00	1.24	[0.12 , 2.36]	Non-inferiority not shown	0.030
[CoC] Furlong HAC Stem CSF	688	2.76	2.00	[0.73 , 3.27]	Inferior by ≥20%	0.002
[CoC] Furlong HAC Stem Furlong HAC CSF Plus	4,023	1.72	0.96	[0.41 , 1.51]	Inferior by ≥20%	0.001
[CoC] M/L Taper Cementless Continuum	704	1.73	0.97	[0.05 , 1.90]	Non-inferiority not shown	0.040
[CoC] Metafix Stem Trinity	344	1.09	0.33	[-0.59 , 1.26]	Non-inferiority not shown	0.479
[CoC] Polarstem Cementless R3 Cementless	341	0.37	-0.39	[-1.06 , 0.27]	Non-inferiority not shown	0.248
[CoC] SL-Plus Cementless Stem EP-Fit Plus	601	5.11	4.36	[2.60 , 6.11]	Inferior by ≥100%	<0.001
[CoC] SL-Plus Cementless Stem R3 Cementless	298	1.48	0.72	[-0.64 , 2.08]	Non-inferiority not shown	0.298
[CoC] SPS Modular April - Ceramic	291	0.59	-0.17	[-1.09 , 0.75]	Non-inferiority not shown	0.722
[CoC] Taperloc Cementless Stem Exceed ABT	2,976	1.64	0.88	[0.30 , 1.46]	Inferior by ≥20%	0.003
[CoC] miniHip Trinity	273	3.21	2.46	[0.68 , 4.24]	Inferior by ≥20%	0.007
[CoP] Accolade Trident	1,319	1.66	0.91	[0.21 , 1.60]	Inferior by ≥20%	0.011
[CoP] C-Stem Cemented Stem Elite Plus Ogee	265	0.93	0.17	[-0.96 , 1.30]	Non-inferiority not shown	0.771
[CoP] C-Stem Cemented Stem Marathon	526	1.07	0.31	[-0.49 , 1.11]	Non-inferiority not shown	0.447
[CoP] C-Stem Cemented Stem Opera	341	1.15	0.39	[-0.81 , 1.60]	Non-inferiority not shown	0.520
[CoP] C-Stem Cemented Stem Wroblewski Golf Ball	396	0.90	0.14	[-0.84 , 1.12]	Non-inferiority not shown	0.779
[CoP] CPT Trilogy	450	1.91	1.15	[0.24 , 2.05]	Inferior by ≥20%	0.013
[CoP] Corail Charnley and Elite Plus LPW	252	1.10	0.35	[-0.97 , 1.66]	Non-inferiority not shown	0.606
[CoP] Corail Marathon	525	1.69	0.93	[-0.02 , 1.89]	Non-inferiority not shown	0.055
[CoP] Corail Pinnacle	3,493	1.14	0.38	[-0.11 , 0.88]	Non-inferiority not shown	0.131
[CoP] Exeter V40 Charnley and Elite Plus LPW	300	1.07	0.31	[-0.82 , 1.44]	Non-inferiority not shown	0.593
[CoP] Exeter V40 Elite Plus Ogee	536	0.86	0.10	[-0.71 , 0.92]	Non-inferiority not shown	0.803
[CoP] Exeter V40 Exeter Contemporary Hooded	298	2.35	1.59	[-0.00 , 3.18]	Non-inferiority not shown	0.050
[CoP] Exeter V40 Exeter Duration	300	0.27	-0.49	[-1.17 , 0.18]	Non-inferiority not shown	0.152
[CoP] Exeter V40 Exeter X3 Rimfit	911	1.32	0.56	[-0.15 , 1.27]	Non-inferiority not shown	0.120
[CoP] Exeter V40 Pinnacle	251	1.17	0.41	[-0.58 , 1.40]	Non-inferiority not shown	0.418

For peer review only

[CoP] Exeter V40 Trident	1,917	0.79	0.04	[-0.47 , 0.55]	Non-inferiority not shown	0.887
[CoP] Exeter V40 Trilogy	764	1.73	0.97	[0.00 , 1.94]	Non-inferiority not shown	0.050
[CoP] Furlong HAC Stem CSF	2,536	1.18	0.42	[-0.17 , 1.01]	Non-inferiority not shown	0.162
[CoP] Furlong HAC Stem Furlong HAC CSF Plus	699	1.22	0.46	[-0.33 , 1.26]	Non-inferiority not shown	0.250
[CoP] MS-30 Original ME Muller Low Profile Cup	459	0.35	-0.41	[-1.06 , 0.24]	Non-inferiority not shown	0.217
[CoP] SL-Plus Cementless Stem Bicon-Plus	279	3.64	2.88	[0.73 , 5.04]	Inferior by ≥20%	0.009
[CoP] SL-Plus Cementless Stem EP-Fit Plus	366	1.82	1.06	[-0.34 , 2.47]	Non-inferiority not shown	0.138
[CoP] Taperloc Cementless Stem Exceed ABT	821	1.17	0.41	[-0.27 , 1.09]	Non-inferiority not shown	0.240
[MoP] Accolade Trident	3,597	2.40	1.65	[1.02 , 2.27]	Inferior by ≥100%	<0.001
[MoP] Anthology R3 Cementless	427	2.22	1.46	[0.36 , 2.56]	Inferior by ≥20%	0.009
[MoP] C-Stem AMT Cemented Stem Charnley and Elite Plus LPW	717	1.65	0.89	[-0.07 , 1.85]	Non-inferiority not shown	0.070
[MoP] C-Stem AMT Cemented Stem Elite Plus Ogee	473	0.61	-0.15	[-0.89 , 0.59]	Non-inferiority not shown	0.690
[MoP] C-Stem AMT Cemented Stem Marathon	406	1.04	0.28	[-0.47 , 1.03]	Non-inferiority not shown	0.461
[MoP] C-Stem AMT Cemented Stem Pinnacle	495	1.18	0.42	[-0.37 , 1.22]	Non-inferiority not shown	0.296
[MoP] C-Stem Cemented Stem Charnley Ogee	251	0.91	0.15	[-0.96 , 1.26]	Non-inferiority not shown	0.794
[MoP] C-Stem Cemented Stem Charnley and Elite Plus LPW	476	0.79	0.03	[-0.85 , 0.92]	Non-inferiority not shown	0.942
[MoP] C-Stem Cemented Stem Elite Plus Cemented Cup	261	0.70	-0.06	[-1.12 , 1.00]	Non-inferiority not shown	0.918
[MoP] C-Stem Cemented Stem Elite Plus Ogee	1,224	1.18	0.42	[-0.29 , 1.12]	Non-inferiority not shown	0.243
[MoP] C-Stem Cemented Stem Marathon	597	1.40	0.65	[-0.25 , 1.54]	Non-inferiority not shown	0.155
[MoP] C-Stem Cemented Stem Opera	528	1.62	0.86	[-0.27 , 2.00]	Non-inferiority not shown	0.137
[MoP] C-Stem Cemented Stem Wroblewski Golf Ball	354	1.29	0.53	[-0.67 , 1.73]	Non-inferiority not shown	0.388
[MoP] CCA Cemented Stem CCB Cup	337	0.47	-0.28	[-1.07 , 0.50]	Non-inferiority not shown	0.480
[MoP] CLS Cementless Stem Allofit	265	3.32	2.56	[0.49 , 4.63]	Inferior by ≥20%	0.015
[MoP] CPCS Opera	371	0.51	-0.24	[-1.07 , 0.59]	Non-inferiority not shown	0.567
[MoP] CPT Elite Plus Ogee	862	1.64	0.88	[-0.02 , 1.79]	Non-inferiority not shown	0.056
[MoP] CPT Trilogy	2,750	1.70	0.95	[0.35 , 1.54]	Inferior by ≥20%	0.002
[MoP] CPT ZCA	2,153	1.52	0.76	[0.15 , 1.37]	Non-inferiority not shown	0.015
[MoP] Charnley Cemented Stem Charnley Cemented Cup	1,527	1.34	0.58	[-0.12 , 1.28]	Non-inferiority not shown	0.107
[MoP] Charnley Cemented Stem Charnley Ogee	3,293	1.35	0.59	[0.02 , 1.16]	Non-inferiority not shown	0.043
[MoP] Charnley Cemented Stem Charnley and Elite Plus LPW	1,647	0.97	0.22	[-0.41 , 0.84]	Non-inferiority not shown	0.496
[MoP] Charnley Cemented Stem Opera	407	1.15	0.39	[-0.70 , 1.49]	Non-inferiority not shown	0.479
[MoP] Charnley Cemented Stem Wroblewski Golf Ball	382	1.45	0.69	[-0.54 , 1.92]	Non-inferiority not shown	0.270
[MoP] Corail Duraloc Cementless Cup	1,310	1.59	0.83	[0.05 , 1.62]	Non-inferiority not shown	0.038
[MoP] Corail Elite Plus Cemented Cup	337	1.52	0.76	[-0.52 , 2.04]	Non-inferiority not shown	0.245
[MoP] Corail Elite Plus Ogee	471	1.51	0.75	[-0.32 , 1.82]	Non-inferiority not shown	0.171
[MoP] Corail Marathon	978	1.50	0.74	[-0.00 , 1.48]	Non-inferiority not shown	0.050
[MoP] Corail Pinnacle	11,121	1.53	0.78	[0.31 , 1.25]	Inferior by ≥20%	0.001
[MoP] Corail Trilogy	726	1.48	0.72	[-0.21 , 1.66]	Non-inferiority not shown	0.131
[MoP] Exeter V40 ABG II Cementless Cup	258	2.15	1.39	[-0.36 , 3.15]	Non-inferiority not shown	0.120
[MoP] Exeter V40 Cenator Cemented Cup	705	1.30	0.55	[-0.37 , 1.46]	Non-inferiority not shown	0.240
[MoP] Exeter V40 Charnley and Elite Plus LPW	667	1.09	0.33	[-0.50 , 1.16]	Non-inferiority not shown	0.437
[MoP] Exeter V40 Duraloc Cementless Cup	355	2.15	1.40	[-0.14 , 2.93]	Non-inferiority not shown	0.075
[MoP] Exeter V40 Elite Plus Cemented Cup	1,212	0.84	0.08	[-0.56 , 0.72]	Non-inferiority not shown	0.809
[MoP] Exeter V40 Elite Plus Ogee	5,579	0.96	0.21	[-0.29 , 0.70]	Non-inferiority not shown	0.413
[MoP] Exeter V40 Exeter Contemporary Flanged	13,647	1.12	0.36	[-0.09 , 0.82]	Non-inferiority not shown	0.118
[MoP] Exeter V40 Exeter Contemporary Hooded	5,115	1.83	1.07	[0.53 , 1.60]	Inferior by ≥20%	<0.001

For peer review only - http://bmjopen.bmj.com/site/about/guidelines.xhtml

[MoP] Exeter V40 Exeter Duration	4,042	1.60	0.85	[0.29 , 1.41]	Inferior by ≥20%	0.003
[MoP] Exeter V40 Exeter X3 Rimfit	1,854	1.41	0.65	[0.06 , 1.24]	Non-inferiority not shown	0.031
[MoP] Exeter V40 Marathon	418	1.41	0.65	[-0.32 , 1.61]	Non-inferiority not shown	0.188
[MoP] Exeter V40 Opera	714	1.04	0.28	[-0.56 , 1.11]	Non-inferiority not shown	0.514
[MoP] Exeter V40 Pinnacle	784	1.67	0.91	[0.08 , 1.75]	Non-inferiority not shown	0.033
[MoP] Exeter V40 Reflection Cementless	729	1.79	1.04	[0.01 , 2.06]	Non-inferiority not shown	0.048
[MoP] Exeter V40 Trident	6,504	1.31	0.55	[0.07 , 1.04]	Non-inferiority not shown	0.025
[MoP] Exeter V40 Trilogy	3,337	1.06	0.31	[-0.23 , 0.85]	Non-inferiority not shown	0.265
[MoP] Exeter V40 Ultima Cemented Cup	363	2.49	1.73	[0.15 , 3.32]	Non-inferiority not shown	0.032
[MoP] Furlong Cemented Stem JRI Cemented Cup	443	1.25	0.50	[-0.59 , 1.58]	Non-inferiority not shown	0.370
[MoP] Furlong HAC Stem CSF	2,566	1.90	1.14	[0.49 , 1.79]	Inferior by ≥20%	0.001
[MoP] Furlong HAC Stem Furlong HAC CSF Plus	1,170	2.43	1.68	[0.85 , 2.50]	Inferior by ≥100%	<0.001
[MoP] M/L Taper Cementless Allofit	339	1.83	1.08	[-0.26 , 2.41]	Non-inferiority not shown	0.113
[MoP] Muller Straight Stem Original ME Muller Low Profile Cup	414	0.52	-0.24	[-0.97 , 0.49]	Non-inferiority not shown	0.522
[MoP] Muller-Biomet Apollo	748	1.13	0.37	[-0.48 , 1.22]	Non-inferiority not shown	0.395
[MoP] Omnifit Cemented Stem ODC	267	1.04	0.28	[-0.96 , 1.52]	Non-inferiority not shown	0.661
[MoP] Polarstem Cementless R3 Cementless	476	1.24	0.48	[-0.21 , 1.18]	Non-inferiority not shown	0.174
[MoP] SL-Plus Cementless Stem EP-Fit Plus	826	2.89	2.13	[1.01 , 3.26]	Inferior by ≥100%	<0.001
[MoP] Stanmore Modular Stem SHP Cup	267	0.99	0.23	[-0.97 , 1.44]	Non-inferiority not shown	0.702
[MoP] Stanmore Modular Stem Stanmore-Arcom Cup	1,070	1.02	0.26	[-0.44 , 0.97]	Non-inferiority not shown	0.461
[MoP] Synergy Cementless Stem R3 Cementless	503	1.52	0.76	[-0.10 , 1.63]	Non-inferiority not shown	0.083
[MoP] Synergy Cementless Stem Reflection Cementless	591	1.14	0.38	[-0.56 , 1.32]	Non-inferiority not shown	0.429
[MoP] Taperloc Cementless Stem Exceed ABT	1,447	1.40	0.64	[0.00 , 1.28]	Non-inferiority not shown	0.049
[MoP] Versys Cementless Stem Trilogy	369	4.30	3.54	[1.49 , 5.58]	Inferior by ≥100%	0.001

Supplemental table 6b: Difference in Kaplan-Meier failure estimate between a contemporary reference and implants with at least 250 at risk at 5 years since primary in males

Stem/cup brand	Number at risk	Cumulative failure (%)	Difference in failure (%)	95% CI	Equivalence status	p-value
[MoP] Exeter V40 Elite Plus Ogee	4,143	1.29	[REFERENCE]			
[CoC] ABG II Monolithic Cementless Stem ABG II Cementless Cup	536	2.75	1.47	[0.11 , 2.82]	Non-inferiority not shown	0.035
[CoC] ABG II Monolithic Cementless Stem Trident	463	3.15	1.86	[0.36 , 3.37]	Inferior by ≥20%	0.015
[CoC] Accolade Trident	2,264	3.15	1.87	[1.19 , 2.54]	Inferior by ≥20%	<0.001
[CoC] Bimetric Cementless Stem Exceed ABT	259	1.75	0.46	[-0.86 , 1.79]	Non-inferiority not shown	0.494
[CoC] Corail DeltaMotion	261	1.91	0.62	[-0.59 , 1.83]	Non-inferiority not shown	0.315
[CoC] Corail Duraloc Option	449	3.36	2.07	[0.43 , 3.72]	Inferior by ≥20%	0.013
[CoC] Corail Pinnacle	7,860	2.68	1.40	[1.01 , 1.79]	Inferior by ≥20%	<0.001
[CoC] Exeter V40 ABG II Cementless Cup	379	1.39	0.10	[-0.97 , 1.17]	Non-inferiority not shown	0.852
[CoC] Exeter V40 Trident	3,289	1.81	0.52	[0.04 , 1.00]	Non-inferiority not shown	0.034
[CoC] Furlong HAC Stem CSF	664	3.19	1.91	[0.59 , 3.22]	Inferior by ≥20%	0.005
[CoC] Furlong HAC Stem Furlong HAC CSF Plus	2,393	1.91	0.62	[0.15 , 1.10]	Non-inferiority not shown	0.010
[CoC] M/L Taper Cementless Continuum	304	1.73	0.44	[-0.42 , 1.31]	Non-inferiority not shown	0.316
[CoC] SL-Plus Cementless Stem EP-Fit Plus	551	6.43	5.14	[3.22 , 7.07]	Inferior by ≥100%	<0.001

For peer review only

For peer review only - http://bmjopen.bmj.com/site/about/guidelines.xhtml

[CoC] Taperloc Cementless Stem Exceed ABT	1,657	1.87	0.59	[0.07 , 1.11]	Non-inferiority not shown	0.027
[CoP] Accolade Trident	544	2.06	0.77	[0.05 , 1.50]	Non-inferiority not shown	0.037
[CoP] C-Stem Cemented Stem Opera	283	1.49	0.21	[-1.13 , 1.54]	Non-inferiority not shown	0.759
[CoP] C-Stem Cemented Stem Wroblewski Golf Ball	321	1.47	0.18	[-1.03 , 1.39]	Non-inferiority not shown	0.769
[CoP] Corail Pinnacle	1,695	1.78	0.49	[0.01 , 0.98]	Non-inferiority not shown	0.047
[CoP] Exeter V40 Elite Plus Ogee	381	1.11	-0.18	[-1.06 , 0.71]	Non-inferiority not shown	0.697
[CoP] Exeter V40 Exeter Contemporary Flanged	789	0.85	-0.44	[-0.98 , 0.11]	Non-inferior	0.116
[CoP] Exeter V40 Exeter Duration	261	1.01	-0.28	[-1.46 , 0.90]	Non-inferiority not shown	0.641
[CoP] Exeter V40 Trident	891	0.96	-0.33	[-0.79 , 0.13]	Non-inferior	0.162
[CoP] Exeter V40 Trilogy	631	2.00	0.72	[-0.27 , 1.71]	Non-inferiority not shown	0.154
[CoP] Furlong HAC Stem CSF	2,209	1.39	0.10	[-0.42 , 0.62]	Non-inferiority not shown	0.703
[CoP] Furlong HAC Stem Furlong HAC CSF Plus	400	1.38	0.10	[-0.69 , 0.88]	Non-inferiority not shown	0.809
[CoP] MS-30 Original ME Muller Low Profile Cup	330	0.35	-0.94	[-1.50 , -0.38]	Non-inferior	0.001
[CoP] SL-Plus Cementless Stem Bicon-Plus	253	4.74	3.46	[1.01 , 5.90]	Inferior by ≥20%	0.006
[CoP] SL-Plus Cementless Stem EP-Fit Plus	312	3.28	1.99	[0.14 , 3.85]	Non-inferiority not shown	0.035
[CoP] Taperloc Cementless Stem Exceed ABT	309	1.70	0.42	[-0.54 , 1.37]	Non-inferiority not shown	0.391
[MoP] Accolade Trident	2,404	3.37	2.08	[1.45 , 2.71]	Inferior by ≥100%	<0.001
[MoP] C-Stem AMT Cemented Stem Charnley and Elite Plus LPW	444	1.81	0.52	[-0.44 , 1.47]	Non-inferiority not shown	0.286
[MoP] C-Stem AMT Cemented Stem Elite Plus Ogee	253	0.61	-0.68	[-1.34 , -0.01]	Non-inferior	0.045
[MoP] C-Stem Cemented Stem Charnley and Elite Plus LPW	429	1.01	-0.27	[-1.20 , 0.66]	Non-inferiority not shown	0.564
[MoP] C-Stem Cemented Stem Elite Plus Ogee	906	1.28	0.00	[-0.66 , 0.66]	Non-inferiority not shown	0.997
[MoP] C-Stem Cemented Stem Marathon	274	1.82	0.54	[-0.48 , 1.55]	Non-inferiority not shown	0.300
[MoP] C-Stem Cemented Stem Opera	389	2.23	0.94	[-0.34 , 2.23]	Non-inferiority not shown	0.149
[MoP] C-Stem Cemented Stem Wroblewski Golf Ball	269	1.29	0.00	[-1.16 , 1.16]	Non-inferiority not shown	0.999
[MoP] CPCS Opera	281	0.51	-0.77	[-1.54 , -0.01]	Non-inferior	0.048
[MoP] CPT Elite Plus Ogee	630	2.35	1.07	[0.02 , 2.11]	Non-inferiority not shown	0.046
[MoP] CPT Trilogy	1,862	2.39	1.11	[0.50 , 1.71]	Inferior by ≥20%	<0.001
[MoP] CPT ZCA	1,549	2.42	1.13	[0.46 , 1.80]	Inferior by ≥20%	0.001
[MoP] Charnley Cemented Stem Charnley Cemented Cup	1,338	2.22	0.93	[0.15 , 1.71]	Non-inferiority not shown	0.019
[MoP] Charnley Cemented Stem Charnley Ogee	2,824	2.29	1.00	[0.42 , 1.58]	Inferior by ≥20%	0.001
[MoP] Charnley Cemented Stem Charnley and Elite Plus LPW	1,414	1.42	0.14	[-0.49 , 0.76]	Non-inferiority not shown	0.667
[MoP] Charnley Cemented Stem Opera	343	1.68	0.40	[-0.88 , 1.67]	Non-inferiority not shown	0.540
[MoP] Charnley Cemented Stem Wroblewski Golf Ball	311	1.75	0.47	[-0.86 , 1.79]	Non-inferiority not shown	0.489
[MoP] Corail Duraloc Cementless Cup	1,204	2.54	1.25	[0.36 , 2.14]	Inferior by ≥20%	0.006
[MoP] Corail Elite Plus Ogee	352	1.79	0.50	[-0.66 , 1.66]	Non-inferiority not shown	0.397
[MoP] Corail Marathon	465	1.77	0.48	[-0.28 , 1.24]	Non-inferiority not shown	0.216
[MoP] Corail Pinnacle	6,318	1.91	0.63	[0.26 , 0.99]	Inferior by ≥20%	0.001
[MoP] Corail Trilogy	526	2.48	1.20	[0.01 , 2.38]	Non-inferiority not shown	0.047
[MoP] Exeter V40 Cenator Cemented Cup	573	1.74	0.45	[-0.53 , 1.43]	Non-inferiority not shown	0.366
[MoP] Exeter V40 Charnley and Elite Plus LPW	504	1.09	-0.20	[-0.97 , 0.57]	Non-inferiority not shown	0.612
[MoP] Exeter V40 Duraloc Cementless Cup	330	2.45	1.16	[-0.44 , 2.77]	Non-inferiority not shown	0.156
[MoP] Exeter V40 Elite Plus Cemented Cup	906	1.20	-0.09	[-0.74 , 0.57]	Non-inferiority not shown	0.797
[MoP] Exeter V40 Exeter Contemporary Flanged	8,757	1.54	0.26	[-0.09 , 0.60]	Non-inferiority not shown	0.145
[MoP] Exeter V40 Exeter Contemporary Hooded	3,570	2.27	0.98	[0.51 , 1.45]	Inferior by ≥20%	<0.001
[MoP] Exeter V40 Exeter Duration	3,104	2.30	1.02	[0.49 , 1.54]	Inferior by ≥20%	<0.001
[MoP] Exeter V40 Exeter X3 Rimfit	309	1.61	0.33	[-0.31 , 0.96]	Non-inferiority not shown	0.314

For peer review only

[MoP] Exeter V40 Opera	515	1.66	0.37	[-0.61 , 1.35]	Non-inferiority not shown	0.457
[MoP] Exeter V40 Pinnacle	471	2.43	1.14	[0.13 , 2.15]	Non-inferiority not shown	0.027
[MoP] Exeter V40 Reflection Cementless	649	2.49	1.21	[0.06 , 2.35]	Non-inferiority not shown	0.039
[MoP] Exeter V40 Trident	3,769	1.68	0.40	[-0.00 , 0.80]	Non-inferiority not shown	0.051
[MoP] Exeter V40 Trilogy	2,555	1.46	0.18	[-0.31 , 0.66]	Non-inferiority not shown	0.473
[MoP] Exeter V40 Ultima Cemented Cup	321	2.77	1.48	[-0.16 , 3.12]	Non-inferiority not shown	0.077
[MoP] Furlong Cemented Stem JRI Cemented Cup	396	1.48	0.19	[-0.93 , 1.32]	Non-inferiority not shown	0.735
[MoP] Furlong HAC Stem CSF	2,076	2.31	1.02	[0.40 , 1.64]	Inferior by ≥20%	0.001
[MoP] Furlong HAC Stem Furlong HAC CSF Plus	686	3.15	1.86	[0.94 , 2.79]	Inferior by ≥20%	<0.001
[MoP] Muller Straight Stem Original ME Muller Low Profile Cup	291	0.78	-0.51	[-1.33 , 0.31]	Non-inferiority not shown	0.224
[MoP] Muller-Biomet Apollo	568	1.27	-0.02	[-0.85 , 0.81]	Non-inferiority not shown	0.967
[MoP] SL-Plus Cementless Stem EP-Fit Plus	672	4.08	2.79	[1.48 , 4.11]	Inferior by ≥100%	<0.001
[MoP] Stanmore Modular Stem Stanmore-Arcom Cup	805	1.43	0.15	[-0.59 , 0.89]	Non-inferiority not shown	0.697
[MoP] Synergy Cementless Stem Reflection Cementless	546	2.18	0.90	[-0.31 , 2.10]	Non-inferiority not shown	0.146
[MoP] Taperloc Cementless Stem Exceed ABT	657	1.71	0.42	[-0.23 , 1.08]	Non-inferiority not shown	0.208
[MoP] Versys Cementless Stem Trilogy	336	4.56	3.28	[1.20 , 5.36]	Inferior by ≥20%	0.002

For peer review only

Supplemental table 6c: Difference in Kaplan-Meier failure estimate between a contemporary reference and implants with at least 250 at risk at 7 years since primary in males

Stem/cup brand	Number at risk	Cumulative failure (%)	Difference in failure (%)	95% CI	Equivalence status	p-value
[CoP] Furlong HAC Stem CSF	1,823	1.64	[REFERENCE]			
[CoC] ABG II Monolithic Cementless Stem ABG II Cementless Cup	469	3.52	1.89	[0.29 , 3.48]	Non-inferiority not shown	0.020
[CoC] ABG II Monolithic Cementless Stem Trident	365	4.06	2.42	[0.64 , 4.20]	Inferior by ≥20%	0.008
[CoC] Accolade Trident	1,234	3.47	1.84	[1.01 , 2.66]	Inferior by ≥20%	<0.001
[CoC] Corail Duraloc Option	354	4.27	2.64	[0.74 , 4.54]	Inferior by ≥20%	0.006
[CoC] Corail Pinnacle	3,192	3.28	1.64	[1.05 , 2.23]	Inferior by ≥20%	<0.001
[CoC] Exeter V40 ABG II Cementless Cup	304	1.98	0.35	[-1.05 , 1.75]	Non-inferiority not shown	0.627
[CoC] Exeter V40 Trident	2,289	2.18	0.54	[-0.12 , 1.20]	Non-inferiority not shown	0.108
[CoC] Furlong HAC Stem CSF	630	3.34	1.71	[0.30 , 3.11]	Non-inferiority not shown	0.017
[CoC] Furlong HAC Stem Furlong HAC CSF Plus	913	2.10	0.47	[-0.18 , 1.11]	Non-inferiority not shown	0.158
[CoC] SL-Plus Cementless Stem EP-Fit Plus	483	6.96	5.33	[3.28 , 7.38]	Inferior by ≥100%	<0.001
[CoC] Taperloc Cementless Stem Exceed ABT	628	2.14	0.51	[-0.23 , 1.24]	Non-inferiority not shown	0.175
[CoP] C-Stem Cemented Stem Wroblewski Golf Ball	252	1.47	-0.17	[-1.44 , 1.11]	Non-inferiority not shown	0.796
[CoP] Corail Pinnacle	611	2.30	0.66	[-0.12 , 1.45]	Non-inferiority not shown	0.098
[CoP] Exeter V40 Exeter Contemporary Flanged	417	0.85	-0.79	[-1.46 , -0.11]	Non-inferior	0.022
[CoP] Exeter V40 Trident	459	1.46	-0.18	[-1.01 , 0.65]	Non-inferiority not shown	0.676
[CoP] Exeter V40 Trilogy	438	2.16	0.53	[-0.58 , 1.64]	Non-inferiority not shown	0.351
[MoP] Accolade Trident	1,060	4.24	2.60	[1.71 , 3.49]	Inferior by ≥100%	<0.001
[MoP] C-Stem Cemented Stem Charnley and Elite Plus LPW	384	1.01	-0.62	[-1.63 , 0.39]	Non-inferiority not shown	0.227
[MoP] C-Stem Cemented Stem Elite Plus Ogee	654	1.54	-0.09	[-0.94 , 0.76]	Non-inferiority not shown	0.830
[MoP] C-Stem Cemented Stem Opera	282	2.55	0.92	[-0.56 , 2.40]	Non-inferiority not shown	0.225
[MoP] CPT Elite Plus Ogee	436	2.51	0.87	[-0.29 , 2.03]	Non-inferiority not shown	0.140
[MoP] CPT Trilogy	1,134	2.68	1.04	[0.28 , 1.81]	Non-inferiority not shown	0.008
[MoP] CPT ZCA	1,009	3.27	1.63	[0.71 , 2.56]	Inferior by ≥20%	0.001
[MoP] Charnley Cemented Stem Charnley Cemented Cup	1,126	2.94	1.31	[0.31 , 2.30]	Non-inferiority not shown	0.010
[MoP] Charnley Cemented Stem Charnley Ogee	2,211	3.41	1.77	[0.96 , 2.58]	Inferior by ≥20%	<0.001
[MoP] Charnley Cemented Stem Charnley and Elite Plus LPW	1,116	1.90	0.26	[-0.57 , 1.09]	Non-inferiority not shown	0.539
[MoP] Charnley Cemented Stem Wroblewski Golf Ball	256	2.09	0.46	[-1.07 , 1.98]	Non-inferiority not shown	0.559
[MoP] Corail Duraloc Cementless Cup	967	3.62	1.98	[0.84 , 3.13]	Inferior by ≥20%	0.001
[MoP] Corail Pinnacle	2,826	2.42	0.79	[0.21 , 1.37]	Non-inferiority not shown	0.008
[MoP] Corail Trilogy	323	3.87	2.23	[0.58 , 3.89]	Inferior by ≥20%	0.008
[MoP] Exeter V40 Cenator Cemented Cup	415	2.35	0.71	[-0.55 , 1.97]	Non-inferiority not shown	0.267
[MoP] Exeter V40 Charnley and Elite Plus LPW	294	1.33	-0.30	[-1.29 , 0.68]	Non-inferiority not shown	0.547
[MoP] Exeter V40 Elite Plus Cemented Cup	608	1.20	-0.44	[-1.20 , 0.33]	Non-inferiority not shown	0.266
[MoP] Exeter V40 Elite Plus Ogee	2,790	1.68	0.04	[-0.56 , 0.64]	Non-inferiority not shown	0.888
[MoP] Exeter V40 Exeter Contemporary Flanged	5,272	2.01	0.37	[-0.18 , 0.92]	Non-inferiority not shown	0.183
[MoP] Exeter V40 Exeter Contemporary Hooded	2,263	2.84	1.20	[0.53 , 1.88]	Inferior by ≥20%	<0.001
[MoP] Exeter V40 Exeter Duration	2,100	3.36	1.72	[0.96 , 2.49]	Inferior by ≥20%	<0.001
[MoP] Exeter V40 Opera	340	2.14	0.50	[-0.75 , 1.75]	Non-inferiority not shown	0.432
[MoP] Exeter V40 Reflection Cementless	483	3.34	1.70	[0.29 , 3.12]	Non-inferiority not shown	0.018

For peer review only

[MoP] Exeter V40 Trident	2,065	2.04	0.41	[-0.20 , 1.01]	Non-inferiority not shown	0.191
[MoP] Exeter V40 Trilogy	1,688	1.88	0.25	[-0.44 , 0.93]	Non-inferiority not shown	0.477
[MoP] Exeter V40 Ultima Cemented Cup	282	2.77	1.13	[-0.55 , 2.82]	Non-inferiority not shown	0.188
[MoP] Furlong Cemented Stem JRI Cemented Cup	312	1.77	0.13	[-1.18 , 1.45]	Non-inferiority not shown	0.843
[MoP] Furlong HAC Stem CSF	1,556	3.09	1.46	[0.62 , 2.29]	Inferior by ≥20%	0.001
[MoP] Furlong HAC Stem Furlong HAC CSF Plus	265	3.66	2.02	[0.80 , 3.25]	Inferior by ≥20%	0.001
[MoP] Muller-Biomet Apollo	376	1.46	-0.18	[-1.17 , 0.81]	Non-inferiority not shown	0.722
[MoP] SL-Plus Cementless Stem EP-Fit Plus	483	5.32	3.69	[2.08 , 5.29]	Inferior by ≥100%	<0.001
[MoP] Stanmore Modular Stem Stanmore-Arcom Cup	529	1.86	0.23	[-0.74 , 1.20]	Non-inferiority not shown	0.644
[MoP] Synergy Cementless Stem Reflection Cementless	396	2.37	0.73	[-0.59 , 2.05]	Non-inferiority not shown	0.277
[MoP] Versys Cementless Stem Trilogy	297	4.56	2.93	[0.81 , 5.05]	Inferior by ≥20%	0.007

For peer review only

Supplemental table 6d: Difference in Kaplan-Meier failure estimate between a contemporary reference and implants with at least 250 at risk at 10 years since primary in males

Stem/cup brand	Number at risk	Cumulative failure (%)	Difference in failure (%)	95% CI	Equivalence status	p-value
[CoP] Furlong HAC Stem CSF	1,054	2.03	[REFERENCE]			
[CoC] ABG II Monolithic Cementless Stem ABG II Cementless Cup	348	5.43	3.40	[1.33 , 5.47]	Inferior by ≥20%	0.001
[CoC] Corail Pinnacle	363	4.24	2.21	[1.28 , 3.15]	Inferior by ≥20%	<0.001
[CoC] Exeter V40 Trident	767	2.76	0.74	[-0.10 , 1.57]	Non-inferiority not shown	0.083
[CoC] Furlong HAC Stem CSF	360	4.28	2.25	[0.60 , 3.90]	Inferior by ≥20%	0.008
[MoP] C-Stem Cemented Stem Elite Plus Ogee	313	2.71	0.68	[-0.63 , 1.98]	Non-inferiority not shown	0.308
[MoP] CPT Trilogy	382	4.61	2.58	[1.24 , 3.92]	Inferior by ≥20%	<0.001
[MoP] CPT ZCA	332	4.41	2.38	[1.15 , 3.61]	Inferior by ≥20%	<0.001
[MoP] Charnley Cemented Stem Charnley Cemented Cup	615	4.69	2.66	[1.32 , 4.00]	Inferior by ≥20%	<0.001
[MoP] Charnley Cemented Stem Charnley Ogee	1,151	5.08	3.05	[2.00 , 4.10]	Inferior by ≥20%	<0.001
[MoP] Charnley Cemented Stem Charnley and Elite Plus LPW	661	2.93	0.90	[-0.21 , 2.01]	Non-inferiority not shown	0.114
[MoP] Corail Duraloc Cementless Cup	418	6.69	4.66	[2.92 , 6.41]	Inferior by ≥100%	<0.001
[MoP] Corail Pinnacle	638	3.49	1.47	[0.63 , 2.30]	Inferior by ≥20%	0.001
[MoP] Exeter V40 Elite Plus Ogee	1,105	2.53	0.51	[-0.31 , 1.32]	Non-inferiority not shown	0.226
[MoP] Exeter V40 Exeter Contemporary Flanged	1,517	2.88	0.85	[0.13 , 1.57]	Non-inferiority not shown	0.020
[MoP] Exeter V40 Exeter Contemporary Hooded	760	4.17	2.15	[1.19 , 3.11]	Inferior by ≥20%	<0.001
[MoP] Exeter V40 Exeter Duration	888	4.95	2.92	[1.88 , 3.96]	Inferior by ≥20%	<0.001
[MoP] Exeter V40 Trident	402	2.84	0.81	[-0.12 , 1.74]	Non-inferiority not shown	0.089
[MoP] Exeter V40 Trilogy	583	3.02	0.99	[0.02 , 1.96]	Non-inferiority not shown	0.045
[MoP] Furlong HAC Stem CSF	609	4.76	2.73	[1.56 , 3.90]	Inferior by ≥20%	<0.001

For peer review only

Supplemental table 7a: Difference in Kaplan-Meier failure estimate between a contemporary reference and implants with at least 250 at risk at 3 years since primary in males <55 years

Stem/cup brand	Number at risk	Cumulative failure (%)	Difference in failure (%)	95% CI	Equivalence status	p-value
[CoC] Exeter V40 Trident	1,269	1.26	[REFERENCE]			
[CoC] Accolade Trident	730	2.96	1.70	[0.41 , 3.00]	Inferior by ≥20%	0.010
[CoC] Corail Pinnacle	3,796	2.29	1.03	[0.33 , 1.74]	Inferior by ≥20%	0.004
[CoC] Furlong HAC Stem Furlong HAC CSF Plus	837	1.71	0.46	[-0.49 , 1.41]	Non-inferiority not shown	0.345
[CoC] M/L Taper Cementless Continuum	296	2.21	0.95	[-0.59 , 2.49]	Non-inferiority not shown	0.226
[CoC] Taperloc Cementless Stem Exceed ABT	836	2.23	0.98	[-0.05 , 2.00]	Non-inferiority not shown	0.062
[CoP] Accolade Trident	251	2.12	0.87	[-0.64 , 2.37]	Non-inferiority not shown	0.259
[CoP] Corail Pinnacle	509	1.90	0.64	[-0.34 , 1.62]	Non-inferiority not shown	0.201
[CoP] Exeter V40 Trident	369	0.81	-0.44	[-1.25 , 0.37]	Non-inferiority not shown	0.286
[MoP] Corail Pinnacle	417	1.20	-0.06	[-1.12 , 1.00]	Non-inferiority not shown	0.915

For peer review only

Supplemental table 7b: Difference in Kaplan-Meier failure estimate between a contemporary reference and implants with at least 250 at risk at 5 years since primary in males <55 years

Stem/cup brand	Number at risk	Cumulative failure (%)	Difference in failure (%)	95% CI	Equivalence status	p-value
[CoC] Corail Pinnacle	2,313	3.33	[REFERENCE]			
[CoC] Accolade Trident	502	4.15	0.82	[-0.70 , 2.34]	Non-inferiority not shown	0.290
[CoC] Exeter V40 Trident	934	1.87	-1.46	[-2.36 , -0.56]	Non-inferior	0.001
[CoC] Furlong HAC Stem Furlong HAC CSF Plus	494	1.85	-1.48	[-2.45 , -0.50]	Non-inferior	0.003
[CoC] Taperloc Cementless Stem Exceed ABT	430	2.57	-0.76	[-1.88 , 0.35]	Non-inferior	0.179

For peer review only

Supplemental table 8a: Difference in Kaplan-Meier failure estimate between a contemporary reference and implants with at least 250 at risk at 3 years since primary in males between 55 and 75 years

Stem/cup brand	Number at risk	Cumulative failure (%)	Difference in failure (%)	95% CI	Equivalence status	p-value
[CoP] Exeter V40 Trident	1,339	0.83	[REFERENCE]			
[CoC] ABG II Monolithic Cementless Stem ABG II Cementless Cup	408	2.58	1.75	[0.21 , 3.30]	Inferior by ≥20%	0.026
[CoC] ABG II Monolithic Cementless Stem Trident	368	1.82	0.99	[-0.39 , 2.37]	Non-inferiority not shown	0.159
[CoC] Accolade Trident	2,101	2.16	1.33	[0.64 , 2.02]	Inferior by ≥20%	<0.001
[CoC] Bimetric Cementless Stem Exceed ABT	279	0.60	-0.22	[-1.13 , 0.68]	Non-inferiority not shown	0.628
[CoC] Corail Duraloc Option	311	1.86	1.04	[-0.48 , 2.55]	Non-inferiority not shown	0.181
[CoC] Corail Pinnacle	8,280	1.71	0.89	[0.46 , 1.31]	Inferior by ≥20%	<0.001
[CoC] Excia Cementless Plasmacup SC	284	1.25	0.42	[-0.72 , 1.57]	Non-inferiority not shown	0.468
[CoC] Exeter V40 ABG II Cementless Cup	324	0.80	-0.03	[-0.99 , 0.93]	Non-inferiority not shown	0.949
[CoC] Exeter V40 Trident	2,957	1.06	0.23	[-0.26 , 0.72]	Non-inferiority not shown	0.350
[CoC] Furlong HAC Stem CSF	452	3.36	2.53	[0.88 , 4.19]	Inferior by ≥100%	0.003
[CoC] Furlong HAC Stem Furlong HAC CSF Plus	2,839	1.63	0.80	[0.27 , 1.33]	Inferior by ≥20%	0.003
[CoC] M/L Taper Cementless Continuum	405	1.41	0.58	[-0.45 , 1.61]	Non-inferiority not shown	0.269
[CoC] Polarstem Cementless R3 Cementless	256	0.54	-0.29	[-1.11 , 0.54]	Non-inferiority not shown	0.496
[CoC] SL-Plus Cementless Stem EP-Fit Plus	398	4.90	4.07	[2.00 , 6.14]	Inferior by ≥100%	<0.001
[CoC] Taperloc Cementless Stem Exceed ABT	2,008	1.40	0.58	[0.02 , 1.13]	Non-inferiority not shown	0.041
[CoP] Accolade Trident	975	1.56	0.73	[0.03 , 1.44]	Non-inferiority not shown	0.042
[CoP] C-Stem Cemented Stem Marathon	344	0.69	-0.14	[-0.92 , 0.64]	Non-inferiority not shown	0.726
[CoP] C-Stem Cemented Stem Wroblewski Golf Ball	273	0.31	-0.52	[-1.21 , 0.17]	Non-inferiority not shown	0.142
[CoP] CPT Trilogy	274	1.73	0.90	[-0.14 , 1.94]	Non-inferiority not shown	0.089
[CoP] Corail Marathon	368	1.84	1.02	[-0.12 , 2.15]	Non-inferiority not shown	0.079
[CoP] Corail Pinnacle	2,678	0.94	0.11	[-0.33 , 0.54]	Non-inferiority not shown	0.630
[CoP] Exeter V40 Charnley and Elite Plus LPW	275	1.16	0.33	[-0.85 , 1.52]	Non-inferiority not shown	0.585
[CoP] Exeter V40 Elite Plus Ogee	420	0.56	-0.27	[-0.99 , 0.45]	Non-inferiority not shown	0.467
[CoP] Exeter V40 Exeter Contemporary Flanged	919	0.88	0.06	[-0.57 , 0.68]	Non-inferiority not shown	0.862
[CoP] Exeter V40 Exeter X3 Rimfit	649	1.27	0.44	[-0.27 , 1.15]	Non-inferiority not shown	0.225
[CoP] Exeter V40 Trilogy	599	1.36	0.53	[-0.42 , 1.48]	Non-inferiority not shown	0.274
[CoP] Furlong HAC Stem CSF	1,990	0.93	0.11	[-0.43 , 0.64]	Non-inferiority not shown	0.699
[CoP] Furlong HAC Stem Furlong HAC CSF Plus	563	1.40	0.58	[-0.29 , 1.44]	Non-inferiority not shown	0.193
[CoP] MS-30 Original ME Muller Low Profile Cup	375	0.21	-0.62	[-1.16 , -0.07]	Non-inferior	0.026
[CoP] SL-Plus Cementless Stem EP-Fit Plus	268	1.45	0.62	[-0.83 , 2.07]	Non-inferiority not shown	0.404
[CoP] Taperloc Cementless Stem Exceed ABT	601	1.04	0.21	[-0.48 , 0.89]	Non-inferiority not shown	0.552
[MoP] Accolade Trident	2,467	2.30	1.47	[0.83 , 2.12]	Inferior by ≥100%	<0.001
[MoP] Anthology R3 Cementless	292	2.08	1.25	[0.01 , 2.49]	Non-inferiority not shown	0.048
[MoP] C-Stem AMT Cemented Stem Charnley and Elite Plus LPW	419	1.68	0.85	[-0.36 , 2.06]	Non-inferiority not shown	0.167
[MoP] C-Stem AMT Cemented Stem Marathon	256	1.18	0.35	[-0.62 , 1.32]	Non-inferiority not shown	0.475
[MoP] C-Stem AMT Cemented Stem Pinnacle	322	1.15	0.32	[-0.55 , 1.19]	Non-inferiority not shown	0.474
[MoP] C-Stem Cemented Stem Charnley and Elite Plus LPW	322	1.16	0.34	[-0.85 , 1.52]	Non-inferiority not shown	0.579
[MoP] C-Stem Cemented Stem Elite Plus Ogee	830	1.26	0.43	[-0.36 , 1.23]	Non-inferiority not shown	0.283

For peer review only

[MoP] C-Stem Cemented Stem Marathon	418	1.27	0.44	[-0.53 , 1.41]	Non-inferiority not shown	0.372
[MoP] C-Stem Cemented Stem Opera	391	1.47	0.64	[-0.58 , 1.86]	Non-inferiority not shown	0.301
[MoP] CPT Elite Plus Ogee	550	1.47	0.64	[-0.37 , 1.65]	Non-inferiority not shown	0.217
[MoP] CPT Trilogy	1,745	1.63	0.80	[0.18 , 1.42]	Inferior by ≥20%	0.012
[MoP] CPT ZCA	1,031	1.26	0.43	[-0.25 , 1.12]	Non-inferiority not shown	0.213
[MoP] Charnley Cemented Stem Charnley Cemented Cup	1,058	1.60	0.77	[-0.04 , 1.58]	Non-inferiority not shown	0.063
[MoP] Charnley Cemented Stem Charnley Ogee	2,239	1.38	0.55	[-0.03 , 1.13]	Non-inferiority not shown	0.064
[MoP] Charnley Cemented Stem Charnley and Elite Plus LPW	1,040	0.88	0.05	[-0.59 , 0.69]	Non-inferiority not shown	0.886
[MoP] Charnley Cemented Stem Opera	280	1.36	0.53	[-0.84 , 1.90]	Non-inferiority not shown	0.446
[MoP] Corail Duraloc Cementless Cup	978	1.96	1.13	[0.21 , 2.05]	Inferior by ≥20%	0.016
[MoP] Corail Elite Plus Ogee	308	1.86	1.03	[-0.38 , 2.44]	Non-inferiority not shown	0.153
[MoP] Corail Marathon	645	1.37	0.54	[-0.27 , 1.35]	Non-inferiority not shown	0.188
[MoP] Corail Pinnacle	7,845	1.52	0.69	[0.28 , 1.11]	Inferior by ≥20%	0.001
[MoP] Corail Trilogy	517	1.76	0.93	[-0.20 , 2.07]	Non-inferiority not shown	0.108
[MoP] Exeter V40 Cenator Cemented Cup	395	1.46	0.63	[-0.58 , 1.84]	Non-inferiority not shown	0.307
[MoP] Exeter V40 Charnley and Elite Plus LPW	397	1.70	0.87	[-0.35 , 2.09]	Non-inferiority not shown	0.164
[MoP] Exeter V40 Duraloc Cementless Cup	265	2.54	1.71	[-0.18 , 3.60]	Non-inferiority not shown	0.076
[MoP] Exeter V40 Elite Plus Cemented Cup	749	0.95	0.12	[-0.62 , 0.86]	Non-inferiority not shown	0.748
[MoP] Exeter V40 Elite Plus Ogee	3,262	1.05	0.22	[-0.26 , 0.69]	Non-inferiority not shown	0.369
[MoP] Exeter V40 Exeter Contemporary Flanged	8,180	1.18	0.35	[-0.05 , 0.75]	Non-inferiority not shown	0.090
[MoP] Exeter V40 Exeter Contemporary Hooded	2,960	1.74	0.91	[0.36 , 1.45]	Inferior by ≥20%	0.001
[MoP] Exeter V40 Exeter Duration	2,482	1.53	0.70	[0.13 , 1.27]	Non-inferiority not shown	0.016
[MoP] Exeter V40 Exeter X3 Rimfit	1,213	1.48	0.65	[0.03 , 1.28]	Non-inferiority not shown	0.039
[MoP] Exeter V40 Marathon	261	0.86	0.03	[-0.80 , 0.85]	Non-inferiority not shown	0.946
[MoP] Exeter V40 Opera	414	0.71	-0.12	[-0.99 , 0.75]	Non-inferiority not shown	0.785
[MoP] Exeter V40 Pinnacle	462	1.75	0.92	[-0.12 , 1.97]	Non-inferiority not shown	0.084
[MoP] Exeter V40 Reflection Cementless	467	1.44	0.61	[-0.50 , 1.72]	Non-inferiority not shown	0.284
[MoP] Exeter V40 Trident	4,119	1.23	0.40	[-0.04 , 0.85]	Non-inferiority not shown	0.074
[MoP] Exeter V40 Trilogy	2,325	1.03	0.20	[-0.32 , 0.71]	Non-inferiority not shown	0.455
[MoP] Furlong Cemented Stem JRI Cemented Cup	250	1.52	0.69	[-0.83 , 2.21]	Non-inferiority not shown	0.371
[MoP] Furlong HAC Stem CSF	1,718	1.67	0.84	[0.17 , 1.51]	Inferior by ≥20%	0.014
[MoP] Furlong HAC Stem Furlong HAC CSF Plus	689	2.04	1.21	[0.27 , 2.14]	Inferior by ≥20%	0.011
[MoP] M/L Taper Cementless Allofit	280	1.41	0.58	[-0.69 , 1.85]	Non-inferiority not shown	0.373
[MoP] Muller-Biomet Apollo	460	1.24	0.41	[-0.63 , 1.46]	Non-inferiority not shown	0.438
[MoP] Polarstem Cementless R3 Cementless	330	1.22	0.39	[-0.35 , 1.13]	Non-inferiority not shown	0.302
[MoP] SL-Plus Cementless Stem EP-Fit Plus	642	3.41	2.58	[1.24 , 3.91]	Inferior by ≥100%	<0.001
[MoP] Stanmore Modular Stem Stanmore-Arcom Cup	546	0.90	0.07	[-0.72 , 0.87]	Non-inferiority not shown	0.856
[MoP] Synergy Cementless Stem R3 Cementless	318	1.21	0.38	[-0.54 , 1.29]	Non-inferiority not shown	0.416
[MoP] Synergy Cementless Stem Reflection Cementless	439	1.54	0.71	[-0.47 , 1.89]	Non-inferiority not shown	0.239
[MoP] Taperloc Cementless Stem Exceed ABT	971	1.32	0.49	[-0.18 , 1.17]	Non-inferiority not shown	0.154
[MoP] Versys Cementless Stem Trilogy	282	3.38	2.55	[0.46 , 4.64]	Inferior by ≥20%	0.017

For peer review only

Supplemental table 8b: Difference in Kaplan-Meier failure estimate between a contemporary reference and implants with at least 250 at risk at 5 years since primary in males between 55 and 75 years

Stem/cup brand	Number at risk	Cumulative failure (%)	Difference in failure (%)	95% CI	Equivalence status	p-value
[CoP] Furlong HAC Stem CSF	1,759	1.09	[REFERENCE]			
[CoC] ABG II Monolithic Cementless Stem ABG II Cementless Cup	392	2.82	1.73	[0.09 , 3.37]	Non-inferiority not shown	0.038
[CoC] ABG II Monolithic Cementless Stem Trident	306	3.89	2.80	[0.74 , 4.85]	Inferior by ≥20%	0.008
[CoC] Accolade Trident	1,624	2.87	1.77	[0.94 , 2.60]	Inferior by ≥20%	<0.001
[CoC] Corail Duraloc Option	303	2.50	1.41	[-0.36 , 3.18]	Non-inferiority not shown	0.119
[CoC] Corail Pinnacle	5,229	2.37	1.28	[0.74 , 1.82]	Inferior by ≥20%	<0.001
[CoC] Exeter V40 ABG II Cementless Cup	269	1.15	0.06	[-1.16 , 1.28]	Non-inferiority not shown	0.924
[CoC] Exeter V40 Trident	2,279	1.74	0.65	[0.00 , 1.29]	Non-inferiority not shown	0.049
[CoC] Furlong HAC Stem CSF	438	3.80	2.70	[0.93 , 4.48]	Inferior by ≥20%	0.003
[CoC] Furlong HAC Stem Furlong HAC CSF Plus	1,705	1.86	0.76	[0.13 , 1.40]	Non-inferiority not shown	0.018
[CoC] SL-Plus Cementless Stem EP-Fit Plus	367	5.89	4.79	[2.51 , 7.08]	Inferior by ≥100%	<0.001
[CoC] Taperloc Cementless Stem Exceed ABT	1,161	1.61	0.52	[-0.15 , 1.18]	Non-inferiority not shown	0.127
[CoP] Accolade Trident	422	1.94	0.85	[-0.03 , 1.72]	Non-inferiority not shown	0.057
[CoP] Corail Pinnacle	1,340	1.58	0.48	[-0.14 , 1.11]	Non-inferiority not shown	0.131
[CoP] Exeter V40 Elite Plus Ogee	294	0.88	-0.21	[-1.21 , 0.79]	Non-inferiority not shown	0.680
[CoP] Exeter V40 Exeter Contemporary Flanged	635	0.88	-0.21	[-0.90 , 0.48]	Non-inferiority not shown	0.548
[CoP] Exeter V40 Trident	670	0.97	-0.12	[-0.75 , 0.51]	Non-inferiority not shown	0.701
[CoP] Exeter V40 Trilogy	492	1.71	0.62	[-0.48 , 1.72]	Non-inferiority not shown	0.272
[CoP] Furlong HAC Stem Furlong HAC CSF Plus	327	1.60	0.51	[-0.48 , 1.50]	Non-inferiority not shown	0.313
[CoP] MS-30 Original ME Muller Low Profile Cup	267	0.21	-0.88	[-1.49 , -0.27]	Non-inferior	0.005
[MoP] Accolade Trident	1,703	3.33	2.24	[1.42 , 3.05]	Inferior by ≥100%	<0.001
[MoP] C-Stem AMT Cemented Stem Charnley and Elite Plus LPW	283	1.68	0.58	[-0.65 , 1.82]	Non-inferiority not shown	0.355
[MoP] C-Stem Cemented Stem Charnley and Elite Plus LPW	296	1.16	0.07	[-1.15 , 1.29]	Non-inferiority not shown	0.911
[MoP] C-Stem Cemented Stem Elite Plus Ogee	639	1.26	0.17	[-0.67 , 1.01]	Non-inferiority not shown	0.696
[MoP] C-Stem Cemented Stem Opera	288	2.29	1.20	[-0.35 , 2.75]	Non-inferiority not shown	0.129
[MoP] CPT Elite Plus Ogee	426	2.12	1.02	[-0.25 , 2.30]	Non-inferiority not shown	0.116
[MoP] CPT Trilogy	1,247	2.23	1.14	[0.35 , 1.92]	Inferior by ≥20%	0.005
[MoP] CPT ZCA	825	1.91	0.81	[-0.09 , 1.71]	Non-inferiority not shown	0.076
[MoP] Charnley Cemented Stem Charnley Cemented Cup	942	2.86	1.76	[0.67 , 2.85]	Inferior by ≥20%	0.002
[MoP] Charnley Cemented Stem Charnley Ogee	1,978	2.45	1.35	[0.58 , 2.13]	Inferior by ≥20%	0.001
[MoP] Charnley Cemented Stem Charnley and Elite Plus LPW	929	1.28	0.19	[-0.62 , 0.99]	Non-inferiority not shown	0.650
[MoP] Corail Duraloc Cementless Cup	923	2.79	1.69	[0.58 , 2.80]	Inferior by ≥20%	0.003
[MoP] Corail Marathon	326	1.77	0.67	[-0.34 , 1.69]	Non-inferiority not shown	0.193
[MoP] Corail Pinnacle	4,602	1.85	0.76	[0.24 , 1.28]	Inferior by ≥20%	0.004
[MoP] Corail Trilogy	393	2.43	1.34	[-0.05 , 2.72]	Non-inferiority not shown	0.059
[MoP] Exeter V40 Cenator Cemented Cup	337	1.97	0.87	[-0.55 , 2.30]	Non-inferiority not shown	0.229
[MoP] Exeter V40 Charnley and Elite Plus LPW	317	1.70	0.60	[-0.65 , 1.86]	Non-inferiority not shown	0.347
[MoP] Exeter V40 Duraloc Cementless Cup	252	2.92	1.83	[-0.22 , 3.87]	Non-inferiority not shown	0.080
[MoP] Exeter V40 Elite Plus Cemented Cup	583	1.39	0.30	[-0.64 , 1.23]	Non-inferiority not shown	0.533

For peer review only

[MoP] Exeter V40 Elite Plus Ogee	2,569	1.35	0.26	[-0.33 , 0.85]	Non-inferiority not shown	0.390
[MoP] Exeter V40 Exeter Contemporary Flanged	5,599	1.66	0.56	[0.04 , 1.08]	Non-inferiority not shown	0.033
[MoP] Exeter V40 Exeter Contemporary Hooded	2,246	2.26	1.17	[0.50 , 1.84]	Inferior by ≥20%	0.001
[MoP] Exeter V40 Exeter Duration	1,970	2.29	1.20	[0.47 , 1.92]	Inferior by ≥20%	0.001
[MoP] Exeter V40 Opera	298	1.53	0.43	[-0.86 , 1.73]	Non-inferiority not shown	0.512
[MoP] Exeter V40 Pinnacle	305	2.50	1.40	[0.04 , 2.77]	Non-inferiority not shown	0.044
[MoP] Exeter V40 Reflection Cementless	426	2.31	1.22	[-0.21 , 2.64]	Non-inferiority not shown	0.094
[MoP] Exeter V40 Trident	2,530	1.68	0.59	[0.02 , 1.16]	Non-inferiority not shown	0.044
[MoP] Exeter V40 Trilogy	1,851	1.21	0.12	[-0.50 , 0.74]	Non-inferiority not shown	0.703
[MoP] Furlong HAC Stem CSF	1,448	1.97	0.88	[0.11 , 1.65]	Non-inferiority not shown	0.026
[MoP] Furlong HAC Stem Furlong HAC CSF Plus	430	3.06	1.97	[0.70 , 3.23]	Inferior by ≥20%	0.002
[MoP] Muller-Biomet Apollo	375	1.47	0.37	[-0.80 , 1.54]	Non-inferiority not shown	0.531
[MoP] SL-Plus Cementless Stem EP-Fit Plus	554	4.71	3.62	[2.00 , 5.24]	Inferior by ≥100%	<0.001
[MoP] Stanmore Modular Stem Stanmore-Arcom Cup	434	1.51	0.41	[-0.67 , 1.50]	Non-inferiority not shown	0.455
[MoP] Synergy Cementless Stem Reflection Cementless	407	2.46	1.37	[-0.14 , 2.87]	Non-inferiority not shown	0.075
[MoP] Taperloc Cementless Stem Exceed ABT	448	1.64	0.55	[-0.32 , 1.41]	Non-inferiority not shown	0.214
[MoP] Versys Cementless Stem Trilogy	261	3.73	2.64	[0.43 , 4.85]	Inferior by ≥20%	0.019

For peer review only

Supplemental table 8c: Difference in Kaplan-Meier failure estimate between a contemporary reference and implants with at least 250 at risk at 7 years since primary in males between 55 and 75 years

Stem/cup brand	Number at risk	Cumulative failure (%)	Difference in failure (%)	95% CI	Equivalence status	p-value
[CoP] Furlong HAC Stem CSF	1,463	1.34	[REFERENCE]			
[CoC] ABG II Monolithic Cementless Stem ABG II Cementless Cup	345	3.88	2.53	[0.60 , 4.47]	Inferior by ≥20%	0.010
[CoC] Accolade Trident	907	3.15	1.81	[0.90 , 2.71]	Inferior by ≥20%	<0.001
[CoC] Corail Pinnacle	2,130	2.66	1.32	[0.70 , 1.94]	Inferior by ≥20%	<0.001
[CoC] Exeter V40 Trident	1,626	2.00	0.66	[-0.06 , 1.38]	Non-inferiority not shown	0.074
[CoC] Furlong HAC Stem CSF	415	4.02	2.68	[0.84 , 4.53]	Inferior by ≥20%	0.004
[CoC] Furlong HAC Stem Furlong HAC CSF Plus	674	2.13	0.79	[0.06 , 1.51]	Non-inferiority not shown	0.034
[CoC] SL-Plus Cementless Stem EP-Fit Plus	322	6.44	5.09	[2.69 , 7.50]	Inferior by ≥100%	<0.001
[CoC] Taperloc Cementless Stem Exceed ABT	469	1.99	0.65	[-0.20 , 1.49]	Non-inferiority not shown	0.132
[CoP] Corail Pinnacle	480	2.24	0.90	[-0.00 , 1.80]	Non-inferiority not shown	0.051
[CoP] Exeter V40 Exeter Contemporary Flanged	335	0.88	-0.46	[-1.19 , 0.27]	Non-inferiority not shown	0.218
[CoP] Exeter V40 Trident	362	1.17	-0.17	[-0.95 , 0.61]	Non-inferiority not shown	0.668
[CoP] Exeter V40 Trilogy	350	1.92	0.57	[-0.62 , 1.77]	Non-inferiority not shown	0.347
[MoP] Accolade Trident	787	3.94	2.60	[1.64 , 3.55]	Inferior by ≥100%	<0.001
[MoP] C-Stem Cemented Stem Charnley and Elite Plus LPW	272	1.16	-0.18	[-1.42 , 1.07]	Non-inferiority not shown	0.779
[MoP] C-Stem Cemented Stem Elite Plus Ogee	494	1.45	0.11	[-0.84 , 1.06]	Non-inferiority not shown	0.818
[MoP] CPT Elite Plus Ogee	325	2.35	1.01	[-0.37 , 2.38]	Non-inferiority not shown	0.151
[MoP] CPT Trilogy	815	2.41	1.06	[0.21 , 1.92]	Non-inferiority not shown	0.015
[MoP] CPT ZCA	599	2.69	1.35	[0.23 , 2.46]	Non-inferiority not shown	0.018
[MoP] Charnley Cemented Stem Charnley Cemented Cup	813	3.75	2.41	[1.14 , 3.68]	Inferior by ≥20%	<0.001
[MoP] Charnley Cemented Stem Charnley Ogee	1,632	3.68	2.34	[1.39 , 3.29]	Inferior by ≥100%	<0.001
[MoP] Charnley Cemented Stem Charnley and Elite Plus LPW	776	1.98	0.64	[-0.37 , 1.64]	Non-inferiority not shown	0.212
[MoP] Corail Duraloc Cementless Cup	750	3.84	2.50	[1.18 , 3.82]	Inferior by ≥20%	<0.001
[MoP] Corail Pinnacle	2,172	2.41	1.07	[0.44 , 1.70]	Inferior by ≥20%	0.001
[MoP] Corail Trilogy	254	3.58	2.24	[0.45 , 4.03]	Inferior by ≥20%	0.014
[MoP] Exeter V40 Cenator Cemented Cup	263	2.66	1.32	[-0.40 , 3.04]	Non-inferiority not shown	0.134
[MoP] Exeter V40 Elite Plus Cemented Cup	400	1.39	0.05	[-0.92 , 1.02]	Non-inferiority not shown	0.920
[MoP] Exeter V40 Elite Plus Ogee	1,861	1.79	0.45	[-0.24 , 1.14]	Non-inferiority not shown	0.203
[MoP] Exeter V40 Exeter Contemporary Flanged	3,658	2.15	0.81	[0.21 , 1.42]	Non-inferiority not shown	0.008
[MoP] Exeter V40 Exeter Contemporary Hooded	1,560	2.93	1.58	[0.78 , 2.38]	Inferior by ≥20%	<0.001
[MoP] Exeter V40 Exeter Duration	1,396	3.26	1.92	[1.02 , 2.82]	Inferior by ≥20%	<0.001
[MoP] Exeter V40 Reflection Cementless	339	2.55	1.21	[-0.31 , 2.73]	Non-inferiority not shown	0.117
[MoP] Exeter V40 Trident	1,455	1.83	0.48	[-0.16 , 1.12]	Non-inferiority not shown	0.140
[MoP] Exeter V40 Trilogy	1,299	1.71	0.37	[-0.38 , 1.12]	Non-inferiority not shown	0.332
[MoP] Furlong HAC Stem CSF	1,139	2.72	1.37	[0.45 , 2.30]	Inferior by ≥20%	0.004
[MoP] Muller-Biomet Apollo	277	1.47	0.13	[-1.07 , 1.32]	Non-inferiority not shown	0.835
[MoP] SL-Plus Cementless Stem EP-Fit Plus	411	6.01	4.67	[2.79 , 6.55]	Inferior by ≥100%	<0.001
[MoP] Stanmore Modular Stem Stanmore-Arcom Cup	312	2.00	0.66	[-0.64 , 1.96]	Non-inferiority not shown	0.320
[MoP] Synergy Cementless Stem Reflection Cementless	298	2.71	1.37	[-0.23 , 2.96]	Non-inferiority not shown	0.093

For peer review only

For peer review only

Supplemental table 8d: Difference in Kaplan-Meier failure estimate between a contemporary reference and implants with at least 250 at risk at 10 years since primary in males between 55 and 75 years

Stem/cup brand	Number at risk	Cumulative failure (%)	Difference in failure (%)	95% CI	Equivalence status	p-value
[MoP] Exeter V40 Exeter Contemporary Flanged	1,182	3.08	[REFERENCE]			
[CoC] ABG II Monolithic Cementless Stem ABG II Cementless Cup	257	5.45	2.38	[0.02 , 4.73]	Non-inferiority not shown	0.048
[CoC] Exeter V40 Trident	545	2.49	-0.59	[-1.46 , 0.27]	Non-inferior	0.179
[CoP] Furlong HAC Stem CSF	855	1.75	-1.32	[-2.14 , -0.51]	Non-inferior	0.001
[MoP] C-Stem Cemented Stem Elite Plus Ogee	254	2.48	-0.60	[-2.00 , 0.80]	Non-inferiority not shown	0.400
[MoP] CPT Trilogy	302	3.83	0.75	[-0.59 , 2.10]	Non-inferiority not shown	0.272
[MoP] Charnley Cemented Stem Charnley Cemented Cup	486	5.76	2.68	[1.04 , 4.31]	Inferior by ≥20%	0.001
[MoP] Charnley Cemented Stem Charnley Ogee	910	5.47	2.39	[1.21 , 3.58]	Inferior by ≥20%	<0.001
[MoP] Charnley Cemented Stem Charnley and Elite Plus LPW	500	3.21	0.13	[-1.19 , 1.45]	Non-inferiority not shown	0.846
[MoP] Corail Duraloc Cementless Cup	336	7.31	4.23	[2.19 , 6.27]	Inferior by ≥20%	<0.001
[MoP] Corail Pinnacle	545	3.39	0.31	[-0.53 , 1.15]	Non-inferiority not shown	0.468
[MoP] Exeter V40 Elite Plus Ogee	805	2.81	-0.27	[-1.18 , 0.65]	Non-inferiority not shown	0.568
[MoP] Exeter V40 Exeter Contemporary Hooded	600	4.02	0.94	[-0.09 , 1.98]	Non-inferiority not shown	0.074
[MoP] Exeter V40 Exeter Duration	642	5.21	2.14	[0.92 , 3.35]	Inferior by ≥20%	0.001
[MoP] Exeter V40 Trident	335	2.78	-0.30	[-1.32 , 0.72]	Non-inferiority not shown	0.566
[MoP] Exeter V40 Trilogy	482	3.05	-0.03	[-1.09 , 1.03]	Non-inferiority not shown	0.956
[MoP] Furlong HAC Stem CSF	483	4.63	1.55	[0.22 , 2.89]	Non-inferiority not shown	0.023

For peer review only

Supplemental table 9a: Difference in Kaplan-Meier failure estimate between a contemporary reference and implants with at least 250 at risk at 3 years since primary in males >75 years

Stem/cup brand	Number at risk	Cumulative failure (%)	Difference in failure (%)	95% CI	Equivalence status	p-value
[MoP] Exeter V40 Elite Plus Ogee	2,199	0.78	[REFERENCE]			
[CoC] Corail Pinnacle	540	2.35	1.57	[0.38 , 2.76]	Inferior by ≥20%	0.010
[CoC] Furlong HAC Stem Furlong HAC CSF Plus	350	2.46	1.68	[0.26 , 3.10]	Inferior by ≥20%	0.020
[CoP] Corail Pinnacle	306	1.43	0.65	[-0.25 , 1.56]	Non-inferiority not shown	0.159
[CoP] Furlong HAC Stem CSF	326	1.19	0.41	[-0.68 , 1.51]	Non-inferiority not shown	0.456
[MoP] Accolade Trident	911	2.78	2.00	[1.01 , 3.00]	Inferior by ≥100%	<0.001
[MoP] C-Stem AMT Cemented Stem Charnley and Elite Plus LPW	293	1.38	0.60	[-0.65 , 1.85]	Non-inferiority not shown	0.347
[MoP] C-Stem AMT Cemented Stem Elite Plus Ogee	265	0.56	-0.22	[-1.07 , 0.63]	Non-inferiority not shown	0.618
[MoP] C-Stem Cemented Stem Elite Plus Ogee	365	1.07	0.29	[-0.70 , 1.28]	Non-inferiority not shown	0.563
[MoP] CPT Elite Plus Ogee	287	2.08	1.30	[-0.26 , 2.87]	Non-inferiority not shown	0.103
[MoP] CPT Trilogy	894	1.93	1.15	[0.34 , 1.97]	Inferior by ≥20%	0.005
[MoP] CPT ZCA	1,080	1.70	0.93	[0.22 , 1.63]	Inferior by ≥20%	0.010
[MoP] Charnley Cemented Stem Charnley Cemented Cup	398	0.92	0.14	[-0.82 , 1.10]	Non-inferiority not shown	0.771
[MoP] Charnley Cemented Stem Charnley Ogee	959	1.32	0.54	[-0.20 , 1.28]	Non-inferiority not shown	0.154
[MoP] Charnley Cemented Stem Charnley and Elite Plus LPW	559	1.07	0.29	[-0.56 , 1.14]	Non-inferiority not shown	0.507
[MoP] Corail Duraloc Cementless Cup	280	0.64	-0.14	[-1.08 , 0.81]	Non-inferiority not shown	0.780
[MoP] Corail Marathon	317	1.78	1.00	[-0.14 , 2.14]	Non-inferiority not shown	0.084
[MoP] Corail Pinnacle	2,860	1.59	0.81	[0.33 , 1.30]	Inferior by ≥20%	0.001
[MoP] Exeter V40 Cenator Cemented Cup	301	1.13	0.35	[-0.80 , 1.51]	Non-inferiority not shown	0.547
[MoP] Exeter V40 Charnley and Elite Plus LPW	263	0.27	-0.51	[-1.13 , 0.12]	Non-inferior	0.113
[MoP] Exeter V40 Elite Plus Cemented Cup	394	0.78	0.00	[-0.83 , 0.84]	Non-inferiority not shown	0.994
[MoP] Exeter V40 Exeter Contemporary Flanged	5,295	1.03	0.25	[-0.15 , 0.65]	Non-inferiority not shown	0.221
[MoP] Exeter V40 Exeter Contemporary Hooded	2,098	1.84	1.06	[0.48 , 1.64]	Inferior by ≥20%	<0.001
[MoP] Exeter V40 Exeter Duration	1,426	1.51	0.73	[0.08 , 1.39]	Non-inferiority not shown	0.028
[MoP] Exeter V40 Exeter X3 Rimfit	537	1.21	0.43	[-0.28 , 1.13]	Non-inferiority not shown	0.237
[MoP] Exeter V40 Opera	290	1.47	0.69	[-0.63 , 2.01]	Non-inferiority not shown	0.306
[MoP] Exeter V40 Pinnacle	288	1.15	0.37	[-0.62 , 1.36]	Non-inferiority not shown	0.460
[MoP] Exeter V40 Reflection Cementless	256	2.46	1.68	[-0.15 , 3.51]	Non-inferiority not shown	0.072
[MoP] Exeter V40 Trident	2,148	1.41	0.63	[0.11 , 1.15]	Non-inferiority not shown	0.018
[MoP] Exeter V40 Trilogy	867	1.15	0.37	[-0.36 , 1.10]	Non-inferiority not shown	0.319
[MoP] Furlong HAC Stem CSF	770	1.99	1.21	[0.27 , 2.16]	Inferior by ≥20%	0.012
[MoP] Furlong HAC Stem Furlong HAC CSF Plus	449	3.06	2.28	[1.00 , 3.56]	Inferior by ≥100%	<0.001
[MoP] Muller-Biomet Apollo	276	0.96	0.18	[-0.95 , 1.31]	Non-inferiority not shown	0.755
[MoP] Stanmore Modular Stem Stanmore-Arcom Cup	510	1.01	0.23	[-0.64 , 1.09]	Non-inferiority not shown	0.610
[MoP] Taperloc Cementless Stem Exceed ABT	421	1.75	0.97	[0.01 , 1.94]	Non-inferiority not shown	0.049

For peer review only

Supplemental table 9b: Difference in Kaplan-Meier failure estimate between a contemporary reference and implants with at least 250 at risk at 5 years since primary in males >75 years

Stem/cup brand	Number at risk	Cumulative failure (%)	Difference in failure (%)	95% CI	Equivalence status	p-value
[MoP] Exeter V40 Elite Plus Ogee	1,490	1.10	[REFERENCE]			
[CoC] Corail Pinnacle	321	2.82	1.73	[0.35 , 3.11]	Inferior by ≥20%	0.014
[MoP] Accolade Trident	540	3.41	2.32	[1.12 , 3.51]	Inferior by ≥100%	<0.001
[MoP] C-Stem Cemented Stem Elite Plus Ogee	253	1.45	0.36	[-0.91 , 1.62]	Non-inferiority not shown	0.579
[MoP] CPT Trilogy	535	2.58	1.48	[0.46 , 2.50]	Inferior by ≥20%	0.004
[MoP] CPT ZCA	693	2.90	1.80	[0.78 , 2.83]	Inferior by ≥20%	0.001
[MoP] Charnley Cemented Stem Charnley Cemented Cup	325	0.92	-0.17	[-1.16 , 0.82]	Non-inferiority not shown	0.731
[MoP] Charnley Cemented Stem Charnley Ogee	760	1.91	0.81	[-0.12 , 1.75]	Non-inferiority not shown	0.088
[MoP] Charnley Cemented Stem Charnley and Elite Plus LPW	446	1.43	0.33	[-0.68 , 1.35]	Non-inferiority not shown	0.521
[MoP] Corail Pinnacle	1,479	1.98	0.88	[0.27 , 1.49]	Inferior by ≥20%	0.005
[MoP] Exeter V40 Elite Plus Cemented Cup	271	1.04	-0.06	[-1.06 , 0.95]	Non-inferiority not shown	0.910
[MoP] Exeter V40 Exeter Contemporary Flanged	3,050	1.33	0.24	[-0.26 , 0.74]	Non-inferiority not shown	0.350
[MoP] Exeter V40 Exeter Contemporary Hooded	1,283	2.09	1.00	[0.32 , 1.68]	Inferior by ≥20%	0.004
[MoP] Exeter V40 Exeter Duration	1,041	2.08	0.98	[0.17 , 1.80]	Non-inferiority not shown	0.018
[MoP] Exeter V40 Trident	1,100	1.57	0.48	[-0.13 , 1.09]	Non-inferiority not shown	0.125
[MoP] Exeter V40 Trilogy	592	2.07	0.97	[-0.05 , 1.99]	Non-inferiority not shown	0.063
[MoP] Furlong HAC Stem CSF	561	2.27	1.17	[0.12 , 2.22]	Non-inferiority not shown	0.029
[MoP] Stanmore Modular Stem Stanmore-Arcom Cup	358	1.20	0.11	[-0.88 , 1.09]	Non-inferiority not shown	0.834

For peer review only

Supplemental table 9c: Difference in Kaplan-Meier failure estimate between a contemporary reference and implants with at least 250 at risk at 7 years since primary in males >75 years

Stem/cup brand	Number at risk	Cumulative failure (%)	Difference in failure (%)	95% CI	Equivalence status	p-value
[MoP] Exeter V40 Exeter Contemporary Flanged	1,540	1.76	[REFERENCE]			
[MoP] CPT Trilogy	264	3.16	1.40	[0.20 , 2.60]	Non-inferiority not shown	0.022
[MoP] CPT ZCA	390	3.94	2.18	[0.82 , 3.55]	Inferior by ≥20%	0.002
[MoP] Charnley Cemented Stem Charnley Cemented Cup	252	1.25	-0.51	[-1.68 , 0.66]	Non-inferiority not shown	0.393
[MoP] Charnley Cemented Stem Charnley Ogee	502	2.56	0.80	[-0.32 , 1.92]	Non-inferiority not shown	0.162
[MoP] Charnley Cemented Stem Charnley and Elite Plus LPW	308	1.43	-0.33	[-1.34 , 0.68]	Non-inferiority not shown	0.520
[MoP] Corail Pinnacle	542	2.34	0.58	[-0.14 , 1.30]	Non-inferiority not shown	0.116
[MoP] Exeter V40 Elite Plus Ogee	868	1.43	-0.33	[-0.99 , 0.32]	Non-inferior	0.315
[MoP] Exeter V40 Exeter Contemporary Hooded	670	2.53	0.77	[-0.02 , 1.57]	Non-inferiority not shown	0.056
[MoP] Exeter V40 Exeter Duration	643	3.08	1.32	[0.29 , 2.36]	Non-inferiority not shown	0.012
[MoP] Exeter V40 Trident	538	2.35	0.59	[-0.28 , 1.46]	Non-inferiority not shown	0.185
[MoP] Exeter V40 Trilogy	321	2.07	0.31	[-0.71 , 1.32]	Non-inferiority not shown	0.553
[MoP] Furlong HAC Stem CSF	358	3.06	1.30	[0.01 , 2.60]	Non-inferiority not shown	0.049

For peer review only

Supplemental table 10a: Total number of implants at risk at each time-point by age-group and gender in implants with at least 500

	Time since primary procedure			
	**3 years**	**5 years**	**7 years**	**10 years**
Male, <55	7,977	3,749	No data	No data
Male, 55-75	79,299	49,540	27,512	6,584
Male, 75+	23,653	13,124	5,303	No data
Total Male	132,073	82,003	42,294	10,348
Female, <55	10,007	6,016	2,303	No data
Female, 55-75	136,561	91,874	52,940	15,848
Female, 75+	64,959	37,694	19,779	No data
Total Female	244,285	156,505	91,701	24,408
Total	415,608	268,809	156,138	41,908

For peer review only

Supplemental table 10b: Total number of implant failures at each time-point by age-group and gender in implants with at least 500 at risk

	Time since primary procedure			
	**3 years**	**5 years**	**7 years**	**10 years**
Male, <55	260	132	68	53
Male, 55-75	1,039	533	364	442
Male, 75+	293	133	86	57
Total Male	1,618	811	529	560
Female, <55	268	141	102	79
Female, 55-75	1,344	784	539	545
Female, 75+	458	255	161	120
Total Female	2,091	1,189	820	754
Total	3,733	2,009	1,359	1,325

For peer review only

The RECORD statement – checklist of items, extended from the STROBE statement, that should be reported in observational studies using routinely collected health data.

	Item No.	STROBE items	Location in manuscript where items are reported	RECORD items	Location in manuscript where items are reported
**Title and abstract**					
	1	(a) Indicate the study's design with a commonly used term in the title or the abstract (b) Provide in the abstract an informative and balanced summary of what was done and what was found	Title page 1, abstract page 2.	RECORD 1.1: The type of data used should be specified in the title or abstract. When possible, the name of the databases used should be included.<br><br>RECORD 1.2: If applicable, the geographic region and timeframe within which the study took place should be reported in the title or abstract.<br><br>RECORD 1.3: If linkage between databases was conducted for the study, this should be clearly stated in the title or abstract.	Title, abstract page 2.<br><br>Abstract page 2<br><br>N/A
**Introduction**					
Background rationale	2	Explain the scientific background and rationale for the investigation being reported	Introduction Page 4		
Objectives	3	State specific objectives, including any prespecified hypotheses	Introduction Page 5		
**Methods**					
Study Design	4	Present key elements of study design early in the paper	Methods pages 7 to 8		
Setting	5	Describe the setting, locations, and relevant dates, including periods of recruitment, exposure, follow-up, and data collection	Methods page 6 and 7		
Participants	6	*(a) Cohort study* - Give the eligibility criteria, and the sources and methods of selection of participants. Describe methods of follow-up	Methods page 6	RECORD 6.1: The methods of study population selection (such as codes or algorithms used to identify subjects) should be listed in detail. If this is not possible, an	Methods Page 6, supplemental figure 1

For peer review only

		Case-control study - Give the eligibility criteria, and the sources and methods of case ascertainment and control selection. Give the rationale for the choice of cases and controls<br>*Cross-sectional study* - Give the eligibility criteria, and the sources and methods of selection of participants<br><br>*(b) Cohort study* - For matched studies, give matching criteria and number of exposed and unexposed<br>*Case-control study* - For matched studies, give matching criteria and the number of controls per case		explanation should be provided.<br><br>RECORD 6.2: Any validation studies of the codes or algorithms used to select the population should be referenced. If validation was conducted for this study and not published elsewhere, detailed methods and results should be provided.<br><br>RECORD 6.3: If the study involved linkage of databases, consider use of a flow diagram or other graphical display to demonstrate the data linkage process, including the number of individuals with linked data at each stage.	N/A<br><br>N/A
Variables	7	Clearly define all outcomes, exposures, predictors, potential confounders, and effect modifiers. Give diagnostic criteria, if applicable.	Methods page 6 and 7	RECORD 7.1: A complete list of codes and algorithms used to classify exposures, outcomes, confounders, and effect modifiers should be provided. If these cannot be reported, an explanation should be provided.	Methods page 6 (description of data selection, no codes)
Data sources/ measurement	8	For each variable of interest, give sources of data and details of methods of assessment (measurement). Describe comparability of assessment methods if there is more than one group	Methods Page 6		
Bias	9	Describe any efforts to address potential sources of bias			
Study size	10	Explain how the study size was arrived at	Supplemental figure 1		
Quantitative variables	11	Explain how quantitative variables were handled in the analyses. If applicable, describe which groupings were chosen, and why	Methods Page 6 and 7		
Statistical methods	12	(a) Describe all statistical methods, including those used to control for confounding<br>(b) Describe any methods used to	Methods Page 6 and 7		

For peer review only

		examine subgroups and interactions<br>(c) Explain how missing data were addressed<br>(d) *Cohort study* - If applicable, explain how loss to follow-up was addressed<br>*Case-control study* - If applicable, explain how matching of cases and controls was addressed<br>*Cross-sectional study* - If applicable, describe analytical methods taking account of sampling strategy<br>(e) Describe any sensitivity analyses			
Data access and cleaning methods		..		RECORD 12.1: Authors should describe the extent to which the investigators had access to the database population used to create the study population.<br><br>RECORD 12.2: Authors should provide information on the data cleaning methods used in the study.	Acknowledgement section; contributions, page 15<br><br>Methods Page 6
Linkage		..		RECORD 12.3: State whether the study included person-level, institutional-level, or other data linkage across two or more databases. The methods of linkage and methods of linkage quality evaluation should be provided.	No linkage
**Results**					
Participants	13	(a) Report the numbers of individuals at each stage of the study (*e.g.*, numbers potentially eligible, examined for eligibility, confirmed eligible, included in the study, completing follow-up, and analysed)<br>(b) Give reasons for non-participation	Supplemental figure 1 and Results page 8	RECORD 13.1: Describe in detail the selection of the persons included in the study (*i.e.,* study population selection) including filtering based on data quality, data availability and linkage. The selection of included persons can be described in the text and/or by means of the study flow diagram.	Methods, page 6; Results page 8 and supplemental figure 1

For peer review only

		at each stage.<br>(c) Consider use of a flow diagram			
Descriptive data	14	(a) Give characteristics of study participants (*e.g.*, demographic, clinical, social) and information on exposures and potential confounders<br>(b) Indicate the number of participants with missing data for each variable of interest<br>(c) *Cohort study* - summarise follow-up time (*e.g.*, average and total amount)			
Outcome data	15	*Cohort study* - Report numbers of outcome events or summary measures over time<br>*Case-control study* - Report numbers in each exposure category, or summary measures of exposure<br>*Cross-sectional study* - Report numbers of outcome events or summary measures	All supplemental tables; supplemental tables 10a and b		
Main results	16	(a) Give unadjusted estimates and, if applicable, confounder-adjusted estimates and their precision (e.g., 95% confidence interval). Make clear which confounders were adjusted for and why they were included<br>(b) Report category boundaries when continuous variables were categorized<br>(c) If relevant, consider translating estimates of relative risk into absolute risk for a meaningful time period	Results Pages 8 to 11, Supplemental tables		
Other analyses	17	Report other analyses done—e.g., analyses of subgroups and interactions, and sensitivity analyses	Results Page 11		
**Discussion**					
Key results	18	Summarise key results with reference to study objectives	Discussion Page 12		

For peer review only

Limitations	19	Discuss limitations of the study, taking into account sources of potential bias or imprecision. Discuss both direction and magnitude of any potential bias	Discussion Pages 13	RECORD 19.1: Discuss the implications of using data that were not created or collected to answer the specific research question(s). Include discussion of misclassification bias, unmeasured confounding, missing data, and changing eligibility over time, as they pertain to the study being reported.	Discussion Pages 12 to 13
Interpretation	20	Give a cautious overall interpretation of results considering objectives, limitations, multiplicity of analyses, results from similar studies, and other relevant evidence	Discussion Pages 11-13		
Generalisability	21	Discuss the generalisability (external validity) of the study results	Discussion Page 14		
**Other Information**					
Funding	22	Give the source of funding and the role of the funders for the present study and, if applicable, for the original study on which the present article is based	Page 1 and page 15		
Accessibility of protocol, raw data, and programming code		..	N/A	RECORD 22.1: Authors should provide information on how to access any supplemental information such as the study protocol, raw data, or programming code.	N/A

For peer review only

BMJ Open

Assessing the non-inferiority of prosthesis constructs used in hip replacement using data from the National Joint Registry of England, Wales, Northern Ireland and the Isle of Man: A Benchmarking study

Journal:	*BMJ Open*
Manuscript ID	bmjopen-2018-026685.R1
Article Type:	Research
Date Submitted by the Author:	25-Jan-2019
Complete List of Authors:	Deere, Kevin; University of Bristol, School of Social & Community Medicine<br>Whitehouse, Michael; University of Bristol, Musculoskeletal Research Unit<br>Porter, Martyn; Wrightington Hospital<br>Blom, AW; University of Bristol,<br>Sayers, Adrian; University of Bristol, Muscloskeletal Research Unit
**Primary Subject Heading**:	Surgery
Secondary Subject Heading:	Patient-centred medicine
Keywords:	hip arthroplasty, non-inferiority, National Joint Registry, benchmarking, medical devices

SCHOLARONE™
Manuscripts

Assessing the non-inferiority of prosthesis constructs used in hip replacement using data from the National Joint Registry of England, Wales, Northern Ireland and the Isle of Man: A Benchmarking study.

Kevin Deere, Michael R Whitehouse, Martyn L Porter, Ashley W Blom, Adrian Sayers

Musculoskeletal Research Unit, Bristol Medical School, 1st Floor Learning & Research Building, Southmead Hospital, Bristol, BS10 5NB, UK

Kevin Deere
Senior research Associate

Michael R Whitehouse
Reader in Trauma and Orthopaedics

Ashley W Blom
Professor of Orthopaedic Surgery and Head of Translational Health Sciences

Adrian Sayers
Senior Research Fellow

National Institute for Health Research Bristol Biomedical Research Centre, University of Bristol

Michael R Whitehouse
Reader in Trauma and Orthopaedics

Ashley W Blom
Professor of Orthopaedic Surgery and Head of Translational Health Sciences

Centre for Hip Surgery, Wrightington Hospital, Lancashire, WN6 9EP, UK

Martyn L Porter
Consultant Orthopaedic Surgeon

Bristol Medical School, Population Health Sciences, University of Bristol, Bristol, UK

Adrian Sayers
Senior Research Fellow

Corresponding author: Adrian Sayers

Musculoskeletal Research Unit,

1st Floor Learning & Research Building,

Southmead Hospital,

Bristol,

BS10 5NB, UK

Email: adrian.sayers@bristol.ac.uk

Tel: 0117 4147880

Word count: 4,797

For peer review only

ABSTRACT

Objectives To investigate the relative performance of hip prosthesis constructs as compared to the best performing prosthesis constructs and illustrate the substantial variability in performance of currently used prostheses.

Design A non-inferiority study.

Setting The National Joint Registry for England, Wales, Northern Ireland and the Isle of Man (NJR)

Participants All patients with a primary total hip replacement registered in the NJR between 1st April 2003 and 31st December 2016**.**

Main outcome measures Kaplan-Meier failure function for hip prosthesis constructs. Failure difference between best performing construct and remaining constructs.

Methods Using a non-inferiority analysis, the performance of hip prosthesis constructs by brand were compared to the best performing contemporary construct. Construct failure was estimated using the 1-Kaplan Meier method, i.e. an estimate of net failure. The difference in failure between the contemporary benchmark and all other constructs was tested.

Results Of the 4,442 constructs used, only 134 had ≥500 procedures at risk at 3 years post-surgery, 89 of which were not demonstrated to be inferior to the benchmark by at least 100% relative risk. By 10 years post-surgery there were 26 constructs with ≥500 at risk, 13 of which were not demonstrated to be inferior by at least 20% relative risk.

Even fewer constructs were not inferior to the benchmark when analysed by age and gender. At 5 years post-surgery there were 15 constructs in males and 11 in females, aged 55-75, not shown to be inferior.

Conclusions There is great variability in construct performance and the majority of constructs have not been demonstrated to be non-inferior to contemporary benchmarks. We have a duty to inform patients, clinicians and commissioners of this variability in performance.

For peer review only

For peer review only - http://bmjopen.bmj.com/site/about/guidelines.xhtml

ARTICLE SUMMARY

Strengths and limitations of this study

- Data collected from the largest joint registry in the world
- For the first time we have explicitly compared the performance of prosthesis constructs to a contemporary reference.
- Unambiguous presentation of data allows surgeons, patients and policy makers to directly compare commonly used prosthesis constructs to a reference construct.
- Residual and unmeasured confounding factors are likely to be present.
- The number of patients remaining at risk after extended follow up is low, and therefore the power to detect non-inferiority after extended follow up is also low.

For peer review only

For peer review only - http://bmjopen.bmj.com/site/about/guidelines.xhtml

INTRODUCTION

When patients are considering a hip replacement, they would be forgiven for thinking that all hip prostheses function equally.[1] However, all prostheses are not equal as evidenced by the failure of the 3M Capital hip implant, and Metal-on-Metal bearings.[2] The extent to which patients and clinicians are aware of this lack of equality is unclear.

The National Joint Registry for England, Wales, Northern Ireland and the Isle of Man (NJR) was established to monitor the effectiveness of different types of joint replacement surgery, improve clinical standards and to identify poorly performing implants. It has not focused on identifying exceptionally well performing implants due to limitations inherent with routine data collection and interpreting data from a standpoint of cause and effect. The NJR publishes the unadjusted cumulative failure rates of the most commonly used stem and cup brand combinations used in hip replacement surgery.[3]

Therefore the role of promoting perceived good practice has been filled by other organisations such as the Orthopaedic Device Evaluation Panel (ODEP) in the UK,[4] NOV in the Netherlands,[5] and the Australian superior clinical performance program.[6] Benchmarking bodies typically attempt to provide some type of classification to describe whether an implant is functioning at an acceptable level or not.

In the absence of evidence from randomised control trials, benchmarking organisations and prostheses registries are currently the best sources of evidence for prosthesis performance. However, both registries and benchmarking bodies have limitations which make the interpretation of prosthesis, or prosthesis construct, performance difficult. The cumulative failure reported by the NJR gives an indication of implant construct performance in absolute terms, but head-to-head comparison of different constructs is difficult to estimate without more advanced statistical manipulation. The ODEP grading system is focused on individual implants rather than the constructs they form and is based on meeting an acceptable externally decided benchmark. This simple dichotomisation does not facilitate comparison between the many different prosthesis constructs being used today or illustrate the extensive variability in so called well performing prostheses.[7]

Sayers et al. recently proposed a method of comparison for joint replacement prostheses using a non-inferiority design against an external benchmark.[8] However, the primary limitation of this method is the arbitrary requirement for an externally specified benchmark.

In a non-inferiority clinical trial[9] that has failure as an outcome, two treatments (comparator and reference) can be directly compared to ensure that the comparator treatment is within a clinically

For peer review only - http://bmjopen.bmj.com/site/about/guidelines.xhtml

acceptable range (non-inferiority margin) of the performance of the reference treatment at a specified point in time.[10, 11] Therefore, standard methods for conducting non-inferiority trials could be applied in an orthopaedic benchmarking setting, assuming an appropriate comparator, non-inferiority margin, and time of interest can be identified. This is a method we have applied in a medical device setting, namely knee replacements using NJR data, in which we assessed the non-inferiority of knee replacement constructs as compared to a benchmark construct.[12]

Choosing an appropriate contemporary reference is difficult. There is no evidence from randomised trials that suggests any prosthesis construct outperforms all others, therefore the choice of reference is more heuristic. Patients would like to receive the best available care and clinicians would like to provide the best possible care, or at least care that it is non-inferior to the best. Therefore, the natural choice of reference against which all other prostheses should be compared is the construct with the lowest failure rate. However, in order to protect against chance, good fortune and a low observed failure rate, the construct should be used in large enough numbers to mitigate sampling variability.

As the failure rate of prostheses is known to be influenced by both age and gender, the choice of reference should reflect this specificity.[13] Whereas the selection of an appropriate time and non-inferiority margin to assess prosthesis performance is much more subjective, as is the reader's specific interest. For example, a surgeon interested in an older patient with lower life expectancy, may be interested in minimising short-term complications opposed to ensuring long-term implant survivorship.

The aim of this study is to investigate the relative performance of hip prosthesis constructs as compared to the best performing prosthesis constructs using a non-inferiority study design, and illustrate the substantial variability in performance of currently used prostheses. Stem, bearing and cup brand combinations (constructs) are examined against non-inferiority margins of 20% relative risk and 100% relative risk at 3, 5, 7 and 10 years following surgery.

For peer review only - http://bmjopen.bmj.com/site/about/guidelines.xhtml

METHODS

Patients and Data Sources

We identified all patients with a primary total hip replacement (THR) registered in the NJR between 1st April 2003 and 31st December 2016. All patients were consented to be included in the NJR as part of the standard NJR process.

Procedures were included if the bearing surface was either Metal-on-Polyethylene (MoP), Ceramic-on-Polyethylene (CoP) or Ceramic-on-Ceramic (CoC). Procedures using any other bearing surfaces were excluded as were hemiarthroplasty procedures. Procedures were also excluded if the patient age and gender were missing, or the National Health Service (NHS) number was untraceable and therefore mortality unknown. Metal-on-Metal prosthesis constructs were excluded as their very high failure rates across all ages and both genders have already been demonstrated[2, 14] and their use no longer reflects contemporary practice.[7]

Patient and public involvement

Patient representatives sit on the committee structure of the National Joint Registry. The research priorities of the National Joint Registry are identified by this committee structure and approved by the patient representatives. Patients were not involved in the setting of the research question or the outcome measures, nor were they involved in designing or implementing this work or interpretation of the results. We are unable to disseminate results of this study directly to study participants due to the anonymous nature of the data. We plan to disseminate our findings to the National Joint Registry, via their communications team, to consultations relevant to the provision of joint replacement and to the general population through the local and national press.

Primary Exposure

The primary exposure used in this analysis is hip prosthesis construct. This is defined by the femoral stem, acetabular cup combination and bearing combination. Groupings were defined using data recorded by the NJR and based on the catalogue numbers of individual hip prosthesis.

Statistical methods

Using a non-inferiority analysis, the performance of hip prosthesis constructs were compared to an internally identified reference group. Prosthesis construct failure was estimated using the 1-Kaplan Meier method i.e. an estimate of net failure.

Failure is defined using the first linked surgical revision; patients were censored at death or administratively censored on 31st December 2016. In a recent national audit of NJR procedure

For peer review only - http://bmjopen.bmj.com/site/about/guidelines.xhtml

recording compliance [15] the percentage capture rates were 95.7% and 90.3% for primary and revision procedures respectively. The difference in stratum specific failure probabilities compared to the reference were calculated at 3, 5, 7 and 10 years for all prosthesis (stem-cup) combinations, stratified by gender, and stratified by gender and age group (<55 years, 55 to 75 years, >75 years).

The difference and 95% confidence interval (CI) of the difference between the comparator prosthesis construct and the reference prosthesis construct was estimated at the specified time points. The standard error of the difference was constructed using a pooled estimate of the Greenwood Standard error,[16]

$$\widehat{SE(Diff)} = \sqrt{GSE_x^2 + GSE_{ref}^2},$$

and a z-test comparing the difference between the reference and test prosthesis was then constructed,

$$Z = \left(\left(\widehat{F_{test}} - \widehat{F_{ref}}\right) + \delta\right) / \widehat{SE(Diff)}.$$

The stratum specific contemporaneous reference construct was selected as the stem-cup combination with the lowest failure rate with at least 1,000 patients at risk at the time point of interest. The choice of 1,000 procedures of the same construct was based on simulation work by Sayers et al. which demonstrated that 1,000 procedures at risk will give rise to a confidence interval width of approximately 3% (±1.5%).[8] We believe this represents an acceptable minimal level of accuracy to be considered a suitable reference standard.

Two non-inferiority margins were chosen to illustrate the sensitivity of the choice. The first margin was conservatively set at a 20% increase in relative risk of failure compared to the reference, in line with clinical trials using this methodology, albeit towards the upper end.[17] The second was a 100% increase in relative risk, i.e. a doubling in cumulative probability of failure, as this is an easily interpretable outcome.

If a construct had 500 or more patients still at risk, at each time-point, we calculated the difference in failure between that construct and the reference construct. Results are graphically reported for all comparator prosthesis constructs meeting this criterion at each time point of interest. These figures show the failure difference for each construct against the reference and the number of constructs still at risk. The threshold for graphical presentation, 500 procedures, was chosen based on the previous work of Sayers et al.[8] as this would give risk to an individual confidence interval width of approximately 5% (±2.5%), and because it complements the number of procedures at risk used by ODEP when evaluating devices at 10 years. However, as this decision is somewhat arbitrary, we also

For peer review only - http://bmjopen.bmj.com/site/about/guidelines.xhtml

present results in a tabular format for all comparator prosthesis constructs with at least 250 patients at risk at the beginning of the time point of interest (See supplementary tables).

Prosthesis constructs were either classified as non-inferior, inconclusive, or inferior. If the upper confidence interval is less than or equal to the 20% non-inferiority margin, the prosthesis construct was non-inferior. If the lower confidence interval of the difference was greater than the non-inferiority margin at either 20% or 100% the prosthesis construct was classed as inferior at 20% or 100% respectively. If the lower confidence limit is less than the non-inferiority margin, and the upper confidence greater than non-inferiority margin the construct was described as inconclusive, see figure 1 for graphical representation of the classification.

Sensitivity analysis

We repeated all analyses using a historic reference group, this specified the reference at 3, 5, and 7 years as the best performing stem-cup prosthesis construct at 10 years with at least 1,000 still at risk in the stratum of interest.

All analyses were carried out using Stata 14.2 (College Station, Texas, USA).

For peer review only

For peer review only - http://bmjopen.bmj.com/site/about/guidelines.xhtml

RESULTS

There were 890,681 primary hip replacements included in the NJR between 1st April 2003 and 31st December 2016. Following the application of the exclusion criteria defined above, 797,178 procedures were included in the final analysis, see supplementary figure 1. In total 4,442 different prosthesis constructs were used at least once. A detailed description of non-inferiority across all procedures is provided. Due to the large number of clinically relevant subdivisions and sensitivity analyses, results will be described more broadly. Constructs are described using by bearing and brand. Bearings are either ceramic (C), metal (M) or polythene (P). Brands are described listing the stem and cup combination [stem/cup].

Figures were produced for each stratification of gender, age group and time since primary. To view data at 3 years post primary for all men, men<55 years, men aged 55-75 and men>75 years see supplementary figures 2a, 2b, 2c and 2d respectively. To view data at 5 years post primary for all men, men<55 years, men aged 55-75 and men>75 years see supplementary figures 3a, 3b, 3c and 3d respectively. To view data at 7 years post primary for all men, men aged 55-75 and men>75 years see supplementary figures 4a, 4b, and 4c respectively. To view data at 10 years post primary for all men and men aged 55-75 see supplementary figures 5a and 5b respectively. To view data at 3 years post primary for all women, women<55 years, women aged 55-75 and women>75 years see supplementary figures 6a, 6b, 6c and 6d respectively. To view data at 5 years post primary for all women, women<55 years, women aged 55-75 and women>75 years see supplementary figures 7a, 7b, 7c and 7d respectively. To view data at 7 years post primary for all women, women<55 years, women aged 55-75 and women>75 years see supplementary figures 8a, 8b, 8c and 8d respectively. To view data at 10 years post primary for all women and women aged 55-75 see supplementary figures 9a and 9b respectively

For peer review only

Estimates for the difference in failure between the reference and comparator prosthesis constructs with ≥250 procedure at risk at the time of interest for all, and for each stratification of gender and age group were tabulated. To view data for all at 3, 5, 7 and 10 years post primary see supplementary tables 1a, 1b, 1c and 1d respectively. To view data in all women at 3, 5, 7 and 10 years post primary see supplementary tables 2a, 2b, 2c and 2d respectively. To view data for women <55 years at 3, 5 and 7 years post primary see supplementary tables 3a, 3b and 3c respectively. To view data for women between 55-75 years at 3, 5, 7 and 10 years post primary see supplementary tables 4a, 4b, 4c and 4d respectively. To view data for women >75 years at 3, 5 and 7 years post primary see supplementary tables 5a, 5b and 5c respectively. To view data in all men at 3, 5, 7 and 10 years post primary see supplementary tables 6a, 6b, 6c and 6d respectively. To view data in men <55 years at 3 and 5 years post primary see supplementary tables 7a and 7b respectively. To view

For peer review only - http://bmjopen.bmj.com/site/about/guidelines.xhtml

data in men between 55 and 75 years at 3, 5, 7 and 10 years post primary see supplementary tables 8a, 8b, 8c and 8d respectively. To view data in men >75 years at 3, 5 and 7 years post primary see supplementary tables 9a, 9b and 9c respectively. In this analysis there were 415,608 implants at risk at 3 years (in constructs with at least 500 procedures) and 41,908 at 10 years. Of these there were 3,733 implant failures at 3 years and 1,325 at 10 years. The total number of implants at risk and total implant failures for each subdivision and time-point can be seen in supplementary tables 10a and 10b respectively.

Non-inferiority - All procedures

The reference prosthesis construct at 3 years was identified as the CoP MS-30/Low profile Muller. There were 1,554 procedures remaining at risk and the failure rate was 0.39% (95%CI 0.19 – 0.82). There were 134 prosthesis combinations with ≥500 procedures at risk. 90 combinations were classified as inferior to the reference by at least 20% relative risk of failure. 44 of the 90 were shown to be inferior by at least 100% relative risk (figure 2). No prosthesis constructs could be described as non-inferior.

The reference prosthesis construct at 5 years was again identified as CoP MS-30/Low profile Muller. There were 1,125 procedures remaining at risk and the failure rate was 0.55% (95%CI 0.29 – 1.08). There were 99 prosthesis constructs with ≥500 procedures at risk. 74 prosthesis constructs were classified as inferior to the reference by at least 20% relative risk of failure. 39 of the 74 were shown to be inferior by at least 100% relative risk (figure 3). No prosthesis constructs could be described as non-inferior.

The reference prosthesis constructs at 7 years was identified as the MoP Exeter V40/Elite Plus Cemented Cup. There were 1,173 procedures remaining at risk and the failure rate was 0.91% (95%CI 0.64 – 1.28). There were 69 prosthesis constructs with ≥500 procedures at risk. 48 prosthesis constructs were classified as inferior to the reference by at least 20% relative risk of failure. 20 of the 48 were shown to be inferior by at least 100% relative risk (figure 4). No prosthesis constructs could be described as non-inferior.

The reference prosthesis constructs at 10 years was identified as the MoP Exeter V40/Elite Plus Ogee. There were 3,580 procedures remaining at risk and the failure rate was 2.14% (95%CI 1.87 – 2.45). There were 26 prosthesis constructs with ≥500 procedures at risk. 12 prosthesis constructs were classified as inferior to the reference by at least 20% relative risk of failure. 1 of the 12 was

For peer review only - http://bmjopen.bmj.com/site/about/guidelines.xhtml

shown to be inferior by at least 100% relative risk (figure 5). Two prosthesis constructs were identified as non-inferior.

Non-inferiority - Gender specific.

Gender specific non-inferiority analyses were also performed at 3, 5, 7, and 10 years after the primary operation.

At 3 years only a small number of prosthesis constructs demonstrated non-inferiority in comparison to the reference. Most striking is the large variability of prosthesis constructs used in females compared to males (58 different prosthesis constructs were used more than 500 times in males versus 93 in females), and the gender specific heterogeneity in performance. For example, the CoP Exeter V40/Exeter Contemporary Flanged is used as the reference at 3 years in males, yet is inferior by 20% compared to the reference in females at 3 years. A performance difference was also noted in the CoC SL-Plus cementless Stem/EP-Fit Plus between the genders. At 3 years the failure rate for this prosthesis constructs in all females was 1.75% yet in males after the same period the failure was 5.11% ($p<0.001$).

At 5 years the reference failure rate in females is less than half that in males. Whilst there are only 3 prosthesis constructs marked as 100% worse than the reference prosthesis construct in males, there are 24 prosthesis constructs that are 100% worse than the reference in females. Some prosthesis constructs have been used in large numbers despite having relatively poor performance.

At 7 years the reference failure rate in females remained less than half that of males. There were no prosthesis constructs, used in sufficient numbers, which could be described as non-inferior to the reference in both males and females. One prosthesis construct in males was at least 100% worse than the reference, whilst 14 prosthesis constructs were at least 100% worse in females.

At 10 years no prosthesis constructs were described as non-inferior to the reference in both males and females and there were no implants inferior by 100% in either males or females.

Non-inferiority- Gender and Age specific.

Sub-dividing procedures by age and gender highlights the paucity of information available pertaining to either male or female patients <55 years undergoing THR. Similarly, the volume of longer-term outcomes on patients beyond 7 years is relatively low in comparison to the number of implanted prosthesis constructs. Most strikingly is the preference for hard-on-hard bearing surfaces (such as CoC) in younger male patients (<55 years). Five of the six prosthesis constructs with at least 500 procedures at 3 years were CoC, contrasted with the vast majority of prosthesis constructs used in

For peer review only - http://bmjopen.bmj.com/site/about/guidelines.xhtml

older male patients (≥55 years) where either MoP or CoP bearing couples were used. In addition, changes to the distribution of failure rates of prostheses become increasingly apparent. For example, the reference prosthesis construct in males less than 55 years at 3 years has a cumulative failure of 1.26%, whereas the failure rate of the reference in males over 75 years at 3 years has a cumulative failure of 0.78%. This is a 60% increase in relative failure rate of the reference procedure for younger males compared to the reference procedure for older males.

The paucity of constructs in each age/gender group which have been utilised over 500 times and for which non-inferiority to the reference prosthesis construct is demonstrated is notable. For males <55 years, only 4 prosthesis constructs (including the reference construct) meet this requirement at 3 years, 3 prosthesis constructs at 5 years and none thereafter. For women of the same age the numbers are 4 at 3 years, 3 at 5 years and 7 at 7 years, with none at 10 years. In the largest grouping, those aged between 55 and 75 years, for males, 16 prosthesis constructs meet this requirement at 5 years and only 7 at 10 years, whilst for women the number are 12 at 5 years and 9 at 10 years.

Sensitivity Analysis

We conducted a sensitivity analysis which used the reference prosthesis construct at 10 years as the reference at 3, 5, and 7 years. We assume that the failure trajectory of a non-inferior construct, will have the same, or lower failure rate compared to the reference construct at 3, 5, 7 and 10 years. This approach is conservative, as it preserves the status quo with respects implant performance. The reference construct in all procedures at 10 years is the Exeter V40/Elite Plus Ogee with a failure rate of 2.14% (95%CI 1.87 – 2.45). Only 1 prosthesis construct is non-inferior and statistically superior i.e. the Exeter V40/Elite Plus Cemented cup, but it does not have 1,000 implants at risk at 10 years and therefore is not considered to be the reference construct. At 7, 5, and 3 years, the contemporary reference has a 0.59%, a 0.55%, and 0.40% lower failure rate than the historical reference respectively. Whilst the good performance of many prosthesis constructs appear to track, some exhibit substantially variability in their relative performance at the times of interest. Specifically, the MoP Exeter V40/Charnley Ogee is non-inferior to the historical reference at 10 years, but is inferior by 20% at 5 years.

For peer review only - http://bmjopen.bmj.com/site/about/guidelines.xhtml

DISCUSSION

We have demonstrated in 797,178 primary total hip replacements the relative performance of implanted prosthesis constructs in comparison to an internally selected contemporary reference. There is substantial variation in the performance of prosthesis constructs. A non-inferiority approach to benchmarking provides an immediate comparison of commonly used implanted prosthesis constructs compared to an internal contemporary reference and conveys distinct advantages opposed to standard Kaplan-Meier analyses as currently reported in the NJR annual reports or categorical grades provided by ODEP. The heterogeneity in implanted constructs in females compared to males is aptly illustrated, as is the paucity of information in clinically relevant sub-strata. The marked differences in outcomes between the different age/gender sub-strata confirm the importance of comparing prosthesis constructs within these strata.[13] We present this study as a novel way of assessing hip prosthesis constructs and as such there is, in the authors opinion, no relevant evidence published to date.

What is most striking is that so few prosthesis constructs in each age/gender strata meet the criteria of 500 cases at each time point and are at least classified as “inferiority not shown”. Of the 4,442 constructs used, only 7 meet these criteria in males aged 55-75 years (supplementary figure 5b) and 9 in females aged 55-75 years at 10 years (supplementary figure 9b). None meet the criteria in any other age/gender sub-strata at 10 years. Even at the relatively short follow-up of 5 years, only 16 constructs in men (supplementary figure 3c) and 12 in women age 55-75 years (supplementary figure 7c) meet these criteria. Patients would have a reasonable expectation that the implants they receive have a proven track record and have not been demonstrated as having a 20% or more increased revision rate for patients of the same age and gender. It is important to note that some prosthesis constructs have a higher early relative failure rate and a lower relative failure rate in later years and thus are inferior at 3, 5 and 7 years, but inferiority is not shown at 10 years. Examples of this in men aged 55-75 years are the MoP Corail/Pinnacle and MoP Exeter/Contemporary hooded, we believe this effect is principally driven by the lower failure of the reference at earlier timepoints, i.e. a reduction in revision rate with a later cohort. Late failure is preferential to early failure from the patient, societal and health economic perspectives, particularly as revision is unfortunately associated with a high rate of re-revision.[18]

One of the most obvious trends across all stratifications is the outstanding performance of the Exeter V40 stem as part of various prosthesis constructs. However, the heterogeneity in acetabular prostheses paired with the Exeter V40 stem is substantial, as is the subsequent variation in performance. This aptly illustrates the need to benchmark constructs opposed to individual implants which make up prosthesis constructs, which has the potential to provide false reassurance in terms

For peer review only - http://bmjopen.bmj.com/site/about/guidelines.xhtml

of efficacy as the individual elements of a construct are not independent. Patient specific construct selection is another strong feature of the data, with the majority of younger patient receiving CoC bearing surfaces, whereas the majority of older patients receive MoP bearing surfaces.

This analysis does show that certain constructs are either the reference or are non-inferior to the reference prosthesis construct across almost all age and gender strata. This strongly suggests that they could appropriately be used as default options for the majority of patients. This is particularly relevant for inexperienced surgical teams, as they can focus training on, and become expert with, a single prosthesis construct. This has the potential to reduce the risk of technical error, to be cost saving through bulk purchasing arrangements and via a reduction in failure rates. The absolute level of failure of commonly used constructs is relatively low, and less than 5% in many instances. This apparently excellent (ODEP 10A*) performance is exhibited by nearly all prosthesis constructs with sufficient data at 10 years (≥500 patients at risk) and raises questions about whether an externally placed benchmark is the optimal way to ensure best care.

Encouragingly, the sensitivity analysis demonstrates the prognosis for patients undergoing hip replacement is continually improving, and the currently best performing implant at 10 years is unlikely to be as good as the contemporary references at 3, 5, and 7 years when these reach 10 years of follow up. Whilst the refinement of clinical practice and development of prosthesis constructs appears to be raising the bar in performance, it is clear that these improvements are not universal. This raises questions about how implants are introduced into a market safely; ensuring enough prosthesis constructs are implanted to ascertain their relative performance, but no more than the necessary number of prosthesis constructs are implanted to minimise the exposure of patients to poor performing implants, and finally to ensure there is sufficient incentive for implant manufacturers to develop new prostheses that benefit patients.

This analysis has a number of important strengths. We explicitly compare prosthesis constructs to a contemporary reference, and a historical defined reference with known performance using a non-inferiority study design. The unambiguous presentation of data allows surgeons, patients and policy makers to directly compare commonly used prosthesis constructs to a reference construct. We illustrate the paucity of information in clinically relevant sub-strata and the need to compare implant constructs opposed to implant elements. The analysis has a number of limitations; case-mix adjustment by stratification is difficult to assimilate despite the restricted set of confounding factors. Residual and unmeasured confounding factors are likely to be present, and the ability to interpret analyses from a causal perspective is limited. It is also known that revision rate is influenced by factors such as the primary indication. In this study there is a breadth of indications for the primary

For peer review only - http://bmjopen.bmj.com/site/about/guidelines.xhtml

procedure however 89.1% (709,902) of procedures had osteoarthritis listed as the only reason for primary whereas fractured neck of femur was the reason for primary in 1,802 (0.2%) cases. The NJR annual report illustrates that neck of femur fracture is associated with a small increase in risk of revision in the first 6 months, but then revision rate is approximately equal to other patients.[3] The number of patients remaining at risk after extended follow up is low, and therefore the power to detect non-inferiority assuming there is truly no difference in prosthesis constructs compared to the reference is also low. This phenomenon is further compounded when we compare stratum specific performance of constructs at nearly all time points, whilst reducing the number of procedures required to be at risk may be appealing to increase the number of comparisons, the precision of those comparisons would be low, and therefore not informative. It is also possible that the constructs listed in this analysis may use a mixture of liner materials (i.e. cross-linked polyethylene (XLPE) and non-XLPE) which may lead to variation of revision rates within constructs. Data entry is mandated and data capture is extremely high (over 95%),[15] thus the findings in this study are highly likely to be generalisable. Furthermore, the reasons for failing to record those remaining primaries or revisions in the NJR is unlikely to related to the choice of implant. Therefore, these data would be classified as missing completely at random and would be unlikely to bias our results.[19]

Conclusions, policy and future research implications

The use of product benchmarking has the potential to be highly informative for patients, change the practice of surgeons, and influence policy makers if presented clearly and unambiguously. Clinical implications of this research are far reaching. We are unable to definitively state which construct is the best choice for all patients, due to the presence of selection effects and residual confounding. However, we believe that the information presented here illustrates the variability, frequency and performance of different constructs currently used in clinical practice. This in turn, should be used to further inform the consenting process between the patient and the surgeon, and facilitate implant selection. We believe commissioners and policy makers should consider the variability and performance of different implants before commissioning healthcare providers. Furthermore, qualitative research is required to understand why surgeons select new implants, with limited understanding of long-term performance, in favour of constructs with demonstrably low failure rates e.g. the [MoP] Exeter V40 Elite plus Ogee, [MoP] Exeter V40 Charnley Ogee, [MoP] Exeter V40 Elite plus cemented cup which represented only approximately 1% of the 91,698 constructs implanted in the NJR in 2017. [3]

For peer review only - http://bmjopen.bmj.com/site/about/guidelines.xhtml

Acknowledgments

We thank the patients and staff of all the hospitals who have contributed data to the National Joint Registry. We are grateful to the Healthcare Quality Improvement Partnership, the National Joint Registry Steering Committee, and staff at the National Joint Registry for facilitating this work. The views expressed represent those of the authors and do not necessarily reflect those of the NHS, the National Institute for Health Research, the Department of Health, the National Joint Registry Steering Committee or Healthcare Quality Improvement Partnership, who do not vouch for how the information is presented.

Funding

Posts of members of the research team are funded by a contract grant from the National Joint Registry for England, Wales, Northern Ireland and the Isle of Man. This study was also supported by the NIHR Biomedical Research Centre at the University Hospitals Bristol NHS Foundation Trust and the University of Bristol. AS is funded by an MRC Strategic Skills Fellowship MR/L01226X/1.

Competing interest statement

All authors have completed the Unified Competing Interest form at www.icmje.org/coi_disclosure.pdf (available on request from the corresponding author) and declare: funding was received for the submitted work as described in the Funding statement; no financial relationships with any organisations that might have an interest in the submitted work in the previous three years, no other relationships or activities that could appear to have influenced the submitted work.

Author Contributions

AS, MRW and AWB conceived the study. KD, AS and AWB designed the study. The data were extracted by Northgate (Hemel Hempstead, UK). KD and AS managed and analysed data. MRW, AS, MLP and AWB reviewed the published work. All authors critically inputted into final design of the study, interpreted the data and co-wrote the manuscript. AS is the guarantor and attests on behalf of the authors that the manuscript is a true and transparent reflection of the study without omissions.

Role of the funding source

The sponsor of the study had no role in the study design, data collection, analysis, interpretation, or writing of the final report. KD and AS had full access to all of the data and AS had final responsibility for manuscript submission.

Ethical approval

For peer review only

Pseudo anonymised analysis of NJR data is considered as secondary use of clinical registry data, under HRA guidance this does not require formal ethical approval. However, all research projects are internally approved by the NJR. *The full NJR privacy notice can be found at* *http://www.njrcentre.org.uk/njrcentre/About-the-NJR/Privacy-Notice-GDPR*

Data sharing

Access to data is available from the National Joint Registry for England and Wales

For peer review only

Figures

Figure 1: Schematic representation of inferiority and non-inferiority

Figure 2: Difference in failure of implanted constructs compared to a contemporary reference at 3 years, using all stem-cup combinations with ≥500 procedures remaining at risk.

Figure 3: Difference in failure of implanted constructs compared to a contemporary reference at 5 years, using all stem-cup combinations with ≥500 procedures remaining at risk.

Figure 4: Difference in failure of implanted constructs compared to a contemporary reference at 7 years, using all stem-cup combinations with ≥500 procedures remaining at risk.

Figure 5: Difference in failure of implanted constructs compared to a contemporary reference at 10 years, using all stem-cup combinations with ≥500 procedures remaining at risk.

For peer review only

For peer review only - http://bmjopen.bmj.com/site/about/guidelines.xhtml

References

1. Warden J. Warning issued over hip implants. *BMJ* 1998;316(7132):650.
2. Smith AJ, Dieppe P, Howard PW, et al. Failure rates of metal-on-metal hip resurfacings: analysis of data from the National Joint Registry for England and Wales. *Lancet* 2012;380(9855):1759-66. doi: 10.1016/S0140-6736(12)60989-1
3. National Joint Registry for England W, Northern Ireland and the Isle of Man. 15th Annual report.http://www.njrreports.org.uk/Portals/0/PDFdownloads/NJR%2015th%20Annual%20Report%202018.pdf
4. Tucker K. Orthopaedic Data Evaluation Panel: ODEP. Northgate Public Services (UK) Ltd. 2017 [Available from: http://www.odep.org.uk/ODEPProcess/ODEPStatement.aspx.
5. Nederlandse Orthopaedische Vereniging. Classification Orthopaedic Implants: NOV Total Knee Prosthesis 2016-2017 [Available from: https://www.orthopeden.org/downloads/146/classificatie-knie-2017.pdf.
6. Prostheses and Devices Committee (PDC) and Prostheses List Advisory Committee (PLAC) Bulletins. Australian Government; Department of Health [Available from: http://www.health.gov.au/internet/main/publishing.nsf/Content/health-privatehealth-prostheseslist.htm.
7. NJR Reports Prosthesis document. National Joint Registry (NJR), 2016 [Available from: http://www.njrreports.org.uk/Portals/0/PDFdownloads/NJR%2013th%20AR%20Prostheses%20used%20in%20joint%20replacements%202015.pdf.
8. Sayers A, Crowther MJ, Judge A, et al. Determining the sample size required to establish whether a medical device is non-inferior to an external benchmark. *BMJ Open* 2017;7(8):e015397. doi: 10.1136/bmjopen-2016-015397 [published Online First: 2017/08/31]
9. Schumi J, Wittes JT. Through the looking glass: understanding non-inferiority. *Trials* 2011;12:106. doi: 10.1186/1745-6215-12-106
10. Tunes da Silva G, Logan BR, Klein JP. Methods for equivalence and noninferiority testing. *Biol Blood Marrow Transplant* 2009;15(1 Suppl):120-7. doi: 10.1016/j.bbmt.2008.10.004
11. Com-Nougue C, Rodary C, Patte C. How to establish equivalence when data are censored: a randomized trial of treatments for B non-Hodgkin lymphoma. *Stat Med* 1993;12(14):1353-64.
12. Deere K, Whitehouse MR, Porter M, et al. Assessing the non-inferiority of prosthesis used in total and unicondylar knee replacements using data from the National Joint Registry of England, Wales, Northern Ireland and the Isle of Man: A benchmarking study. *BMJ Open* 2019:xxxx. doi: xxx/bmjopen-2019-xxx
13. Bayliss LE, Culliford D, Monk AP, et al. The effect of patient age at intervention on risk of implant revision after total replacement of the hip or knee: a population-based cohort study. *Lancet* 2017;389(10077):1424-30. doi: 10.1016/S0140-6736(17)30059-4
14. Smith AJ, Dieppe P, Vernon K, et al. Failure rates of stemmed metal-on-metal hip replacements: analysis of data from the National Joint Registry of England and Wales. *Lancet* 2012;379(9822):1199-204. doi: 10.1016/S0140-6736(12)60353-5
15. NJR Data Quality Audit 2017 [Available from: http://www.njrcentre.org.uk/njrcentre/NewsandEvents/DataQualityAudit-updateSeptember2017/tabid/1451/Default.aspx.
16. Greenwood M. The natural duration of cancer. *Rep* ***Public Health*** *Med Subj (Lond)* 1926;33: 1-26
17. Donken R, de Melker HE, Rots NY, et al. Comparing vaccines: a systematic review of the use of the non-inferiority margin in vaccine trials. *Vaccine* 2015;33(12):1426-32. doi: 10.1016/j.vaccine.2015.01.072
18. Hunt LP WM, Beswick A, Porter ML, Howard P, Blom AW. Implications of the Introduction of New Technology: Comparative Survivorship Modelling of Metal-on-Metal Hip Replacement to Contemporary Alternatives in the National Joint Registry. *J Bone Joint Surg Am* 2018 Feb 7;100(3):189-196. doi: 10.2106/JBJS.17.00039

For peer review only - http://bmjopen.bmj.com/site/about/guidelines.xhtml

19. Sterne JA, White IR, Carlin JB, et al. Multiple imputation for missing data in epidemiological and clinical research: potential and pitfalls. *BMJ* 2009;338:b2393. doi: 10.1136/bmj.b2393 [published Online First: 2009/07/01]

For peer review only

For peer review only - http://bmjopen.bmj.com/site/about/guidelines.xhtml

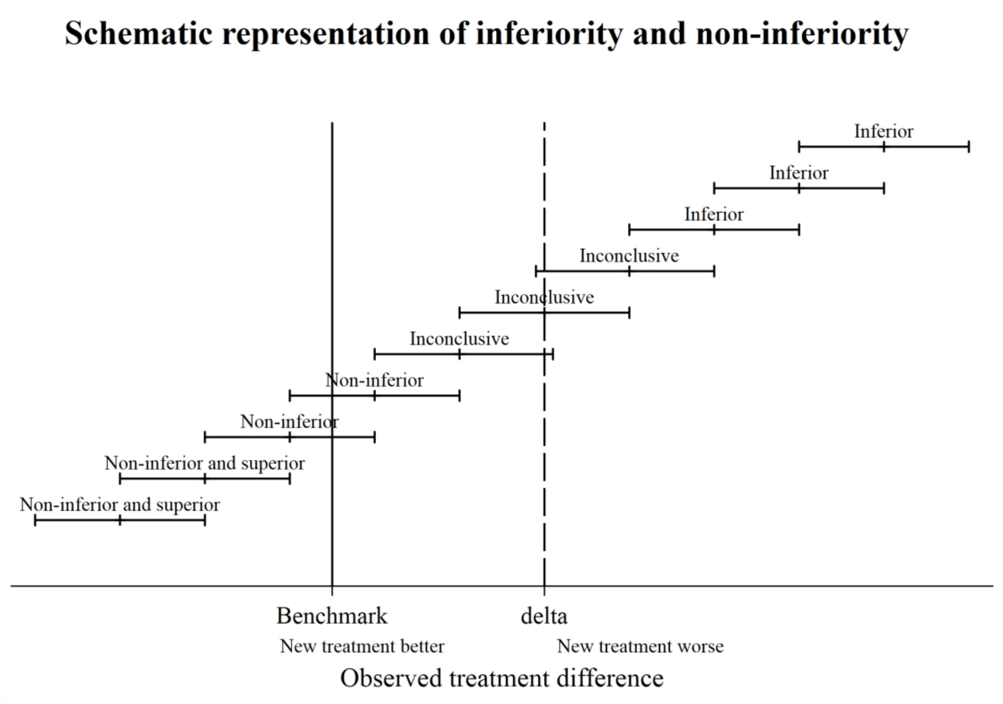

177x124mm (300 x 300 DPI)

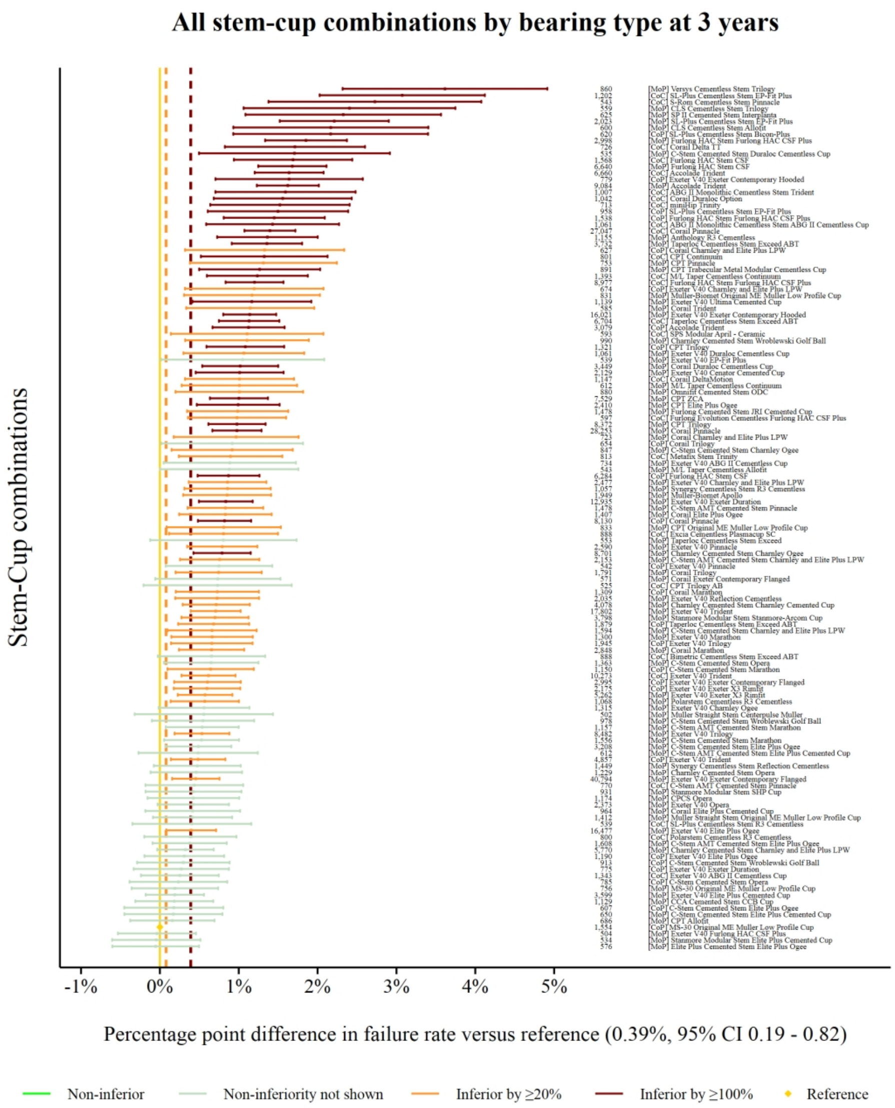

203x254mm (300 x 300 DPI)

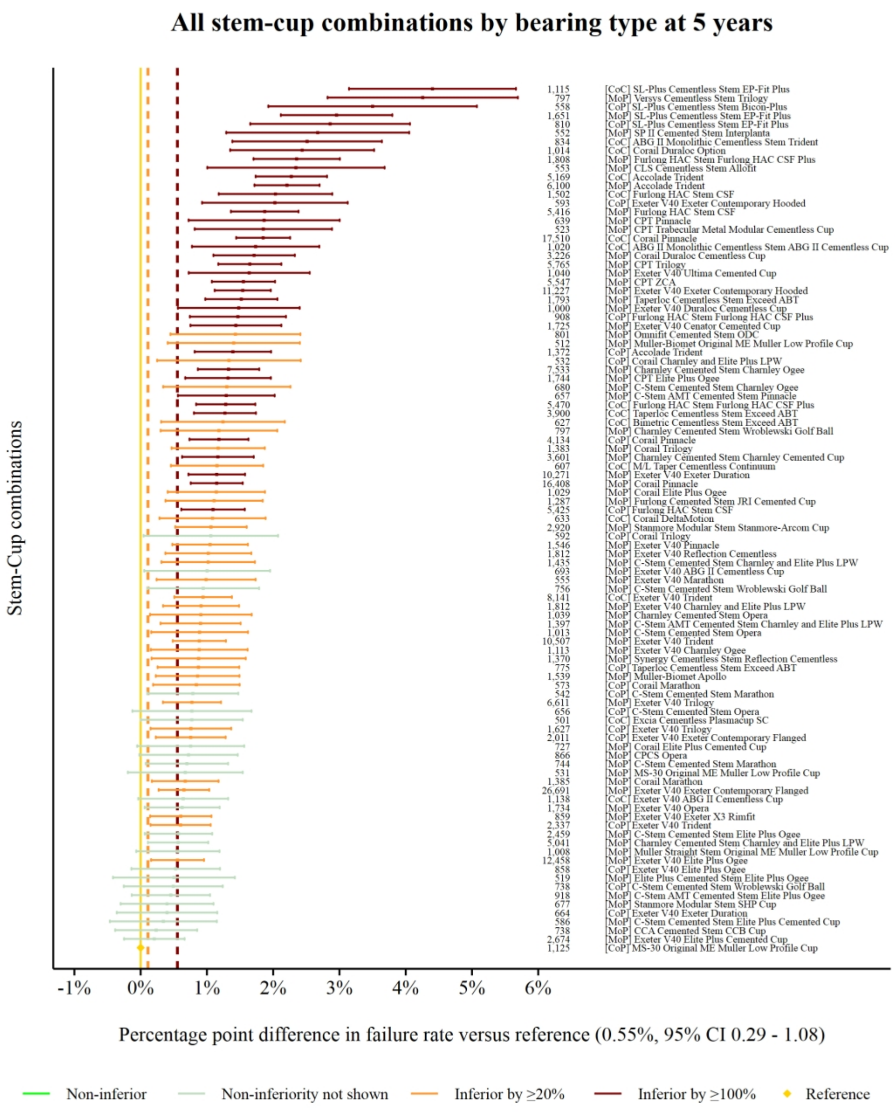

189x237mm (300 x 300 DPI)

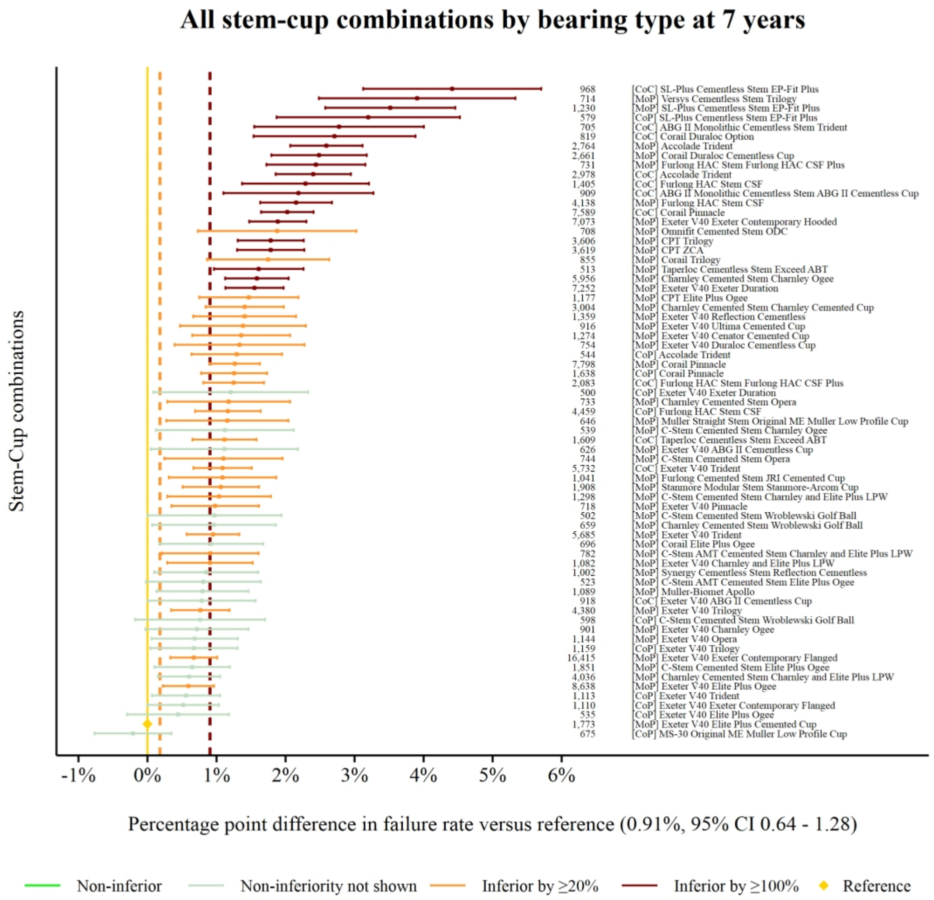

211x211mm (300 x 300 DPI)

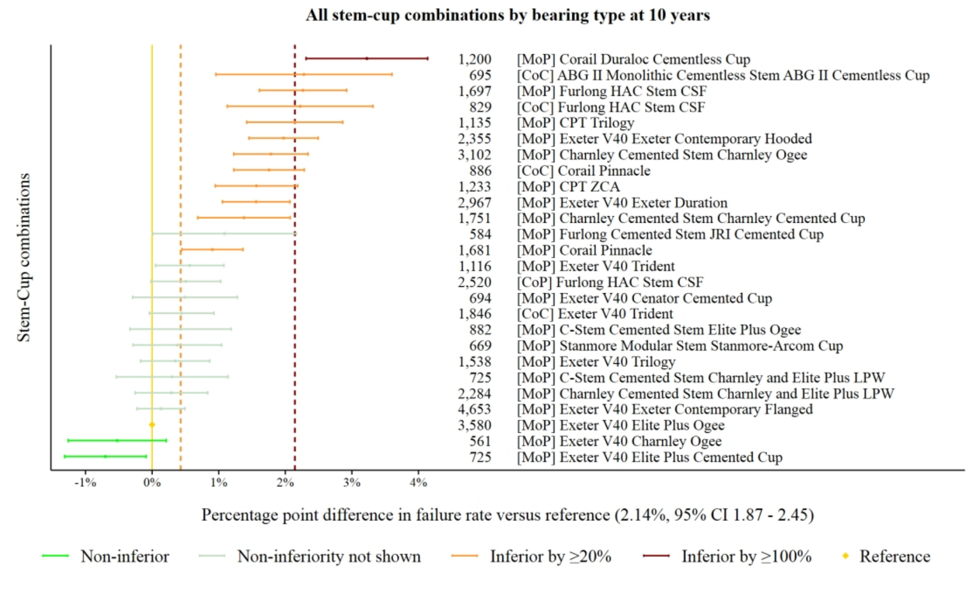

169x105mm (300 x 300 DPI)

Supplementary Figure 1: Flow diagram of showing derivation of procedures used in analyses

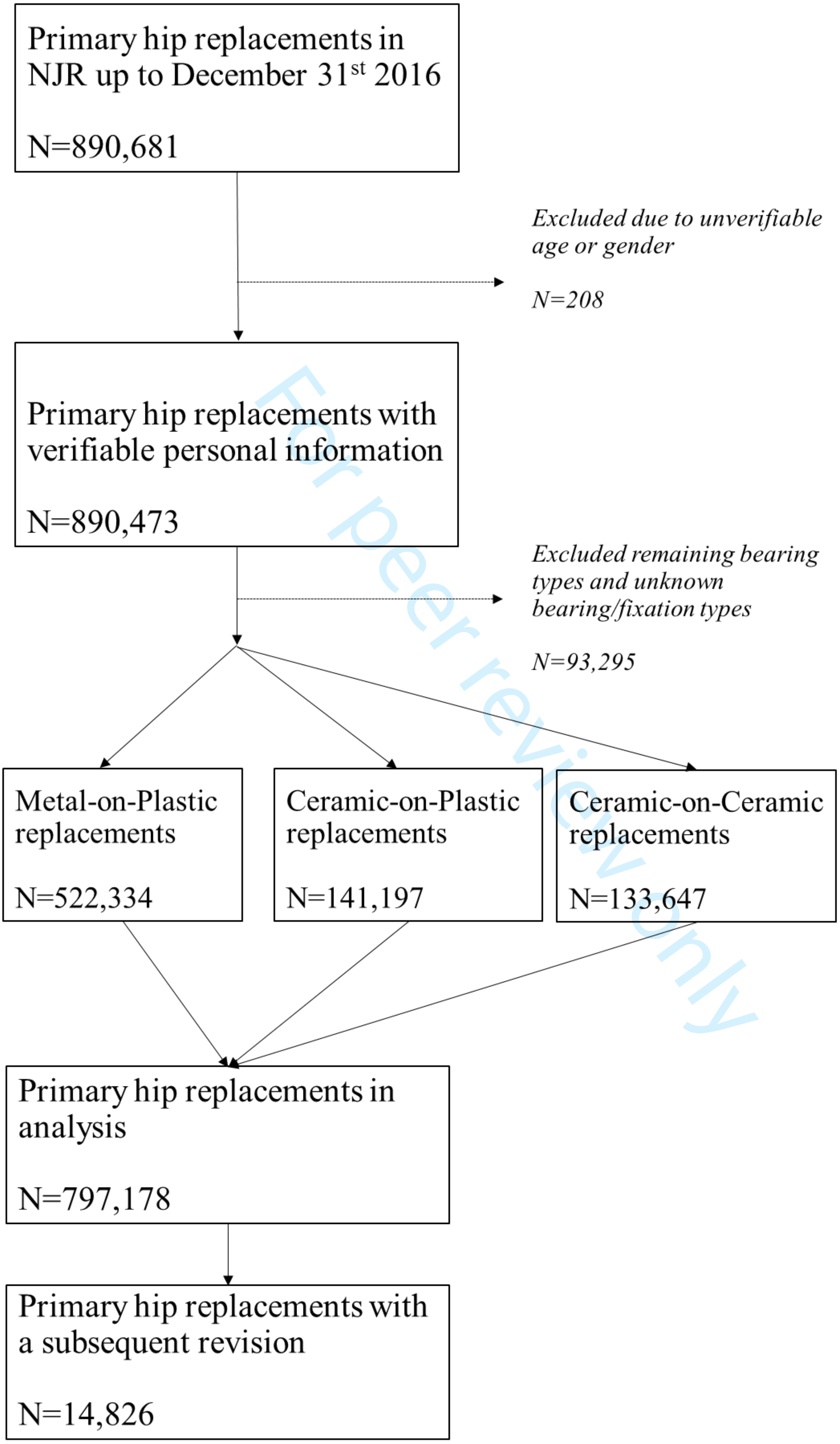

For peer review only

Supplementary Figure 2a: Difference in failure of implanted constructs compared to a contemporary reference at 3 years in men, using all stem-cup combinations with ≥500 procedures remaining at risk

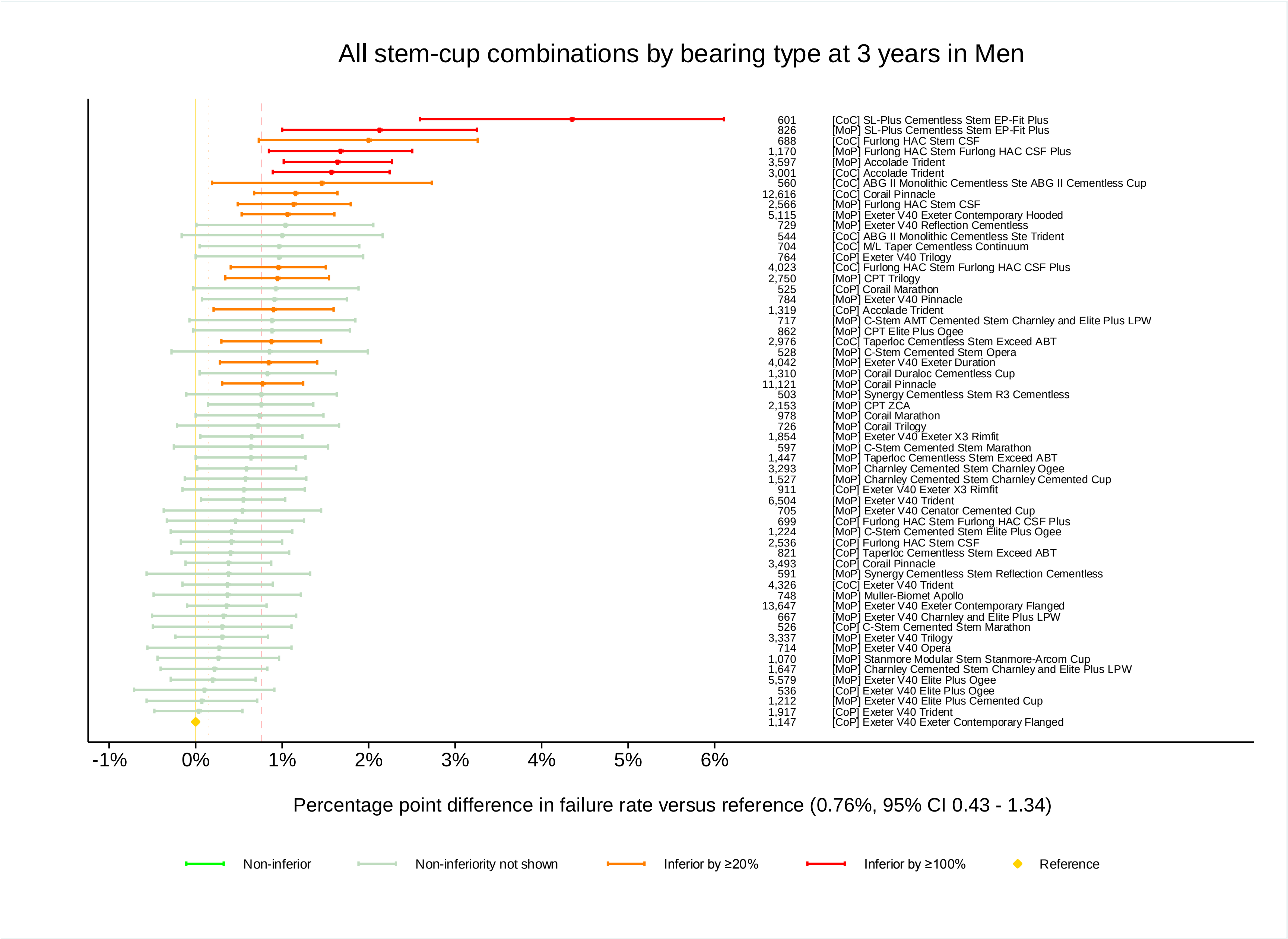

Supplementary Figure 2b: Difference in failure of implanted constructs compared to a contemporary reference at 3 years in men less than 55 years, using all stem-cup combinations with ≥500 procedures remaining at risk

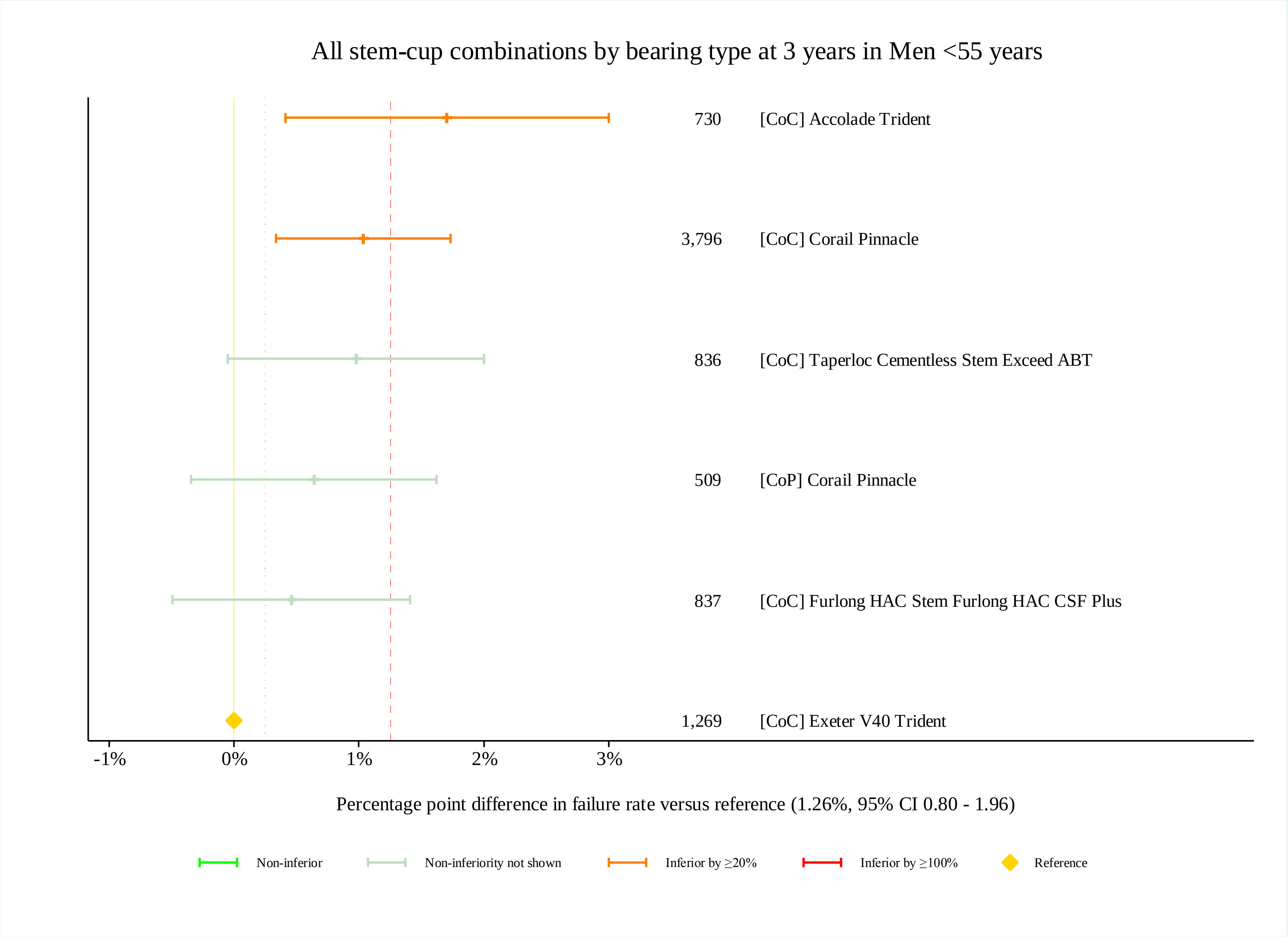

Supplementary Figure 2c: Difference in failure of implanted constructs compared to a contemporary reference at 3 years in men between 55 and 75 years, using all stem-cup combinations with ≥500 procedures remaining at risk

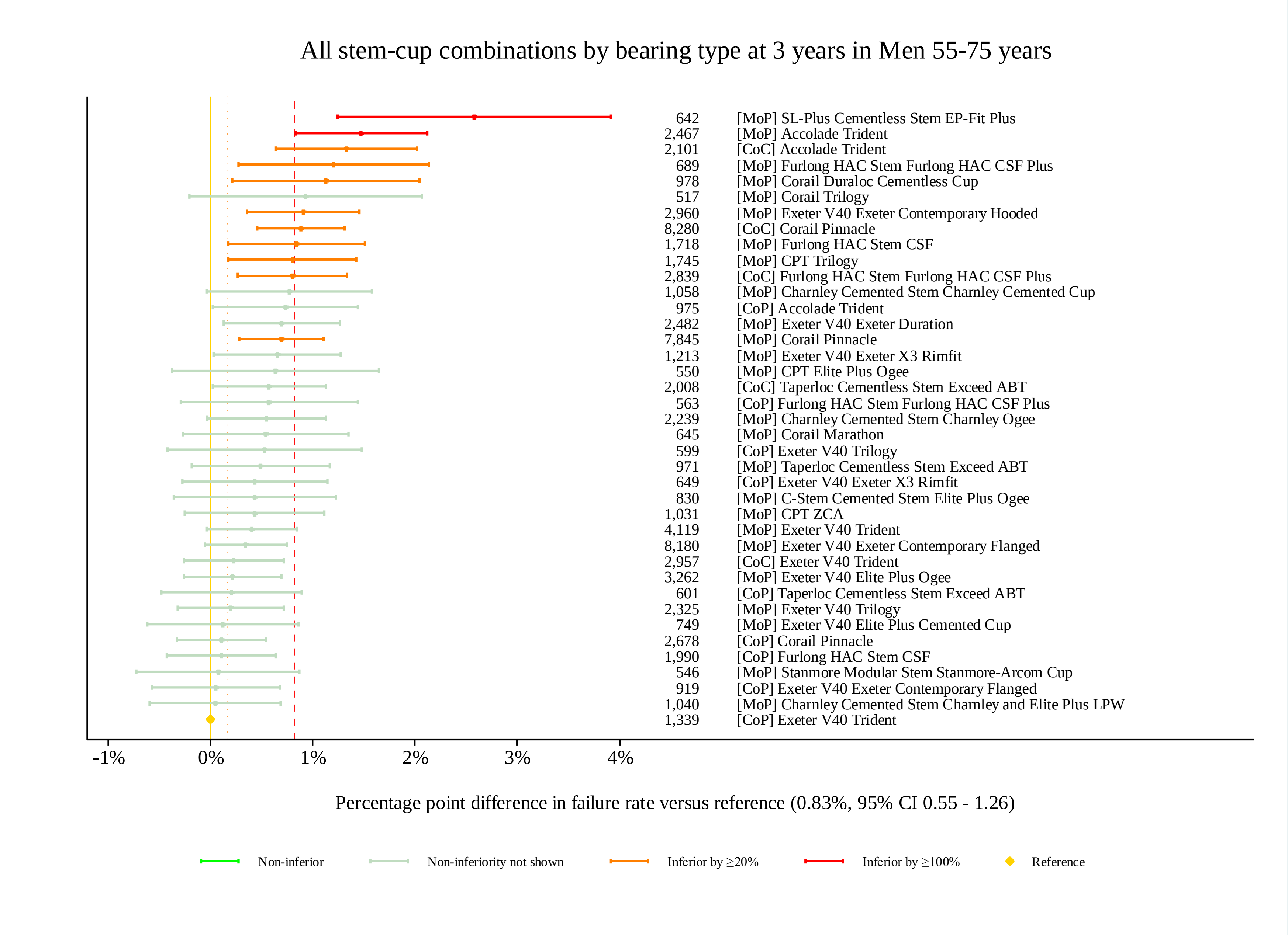

Supplementary Figure 2d: Difference in failure of implanted constructs compared to a contemporary reference at 3 years in men greater than 75 years, using all stem-cup combinations with ≥500 procedures remaining at risk

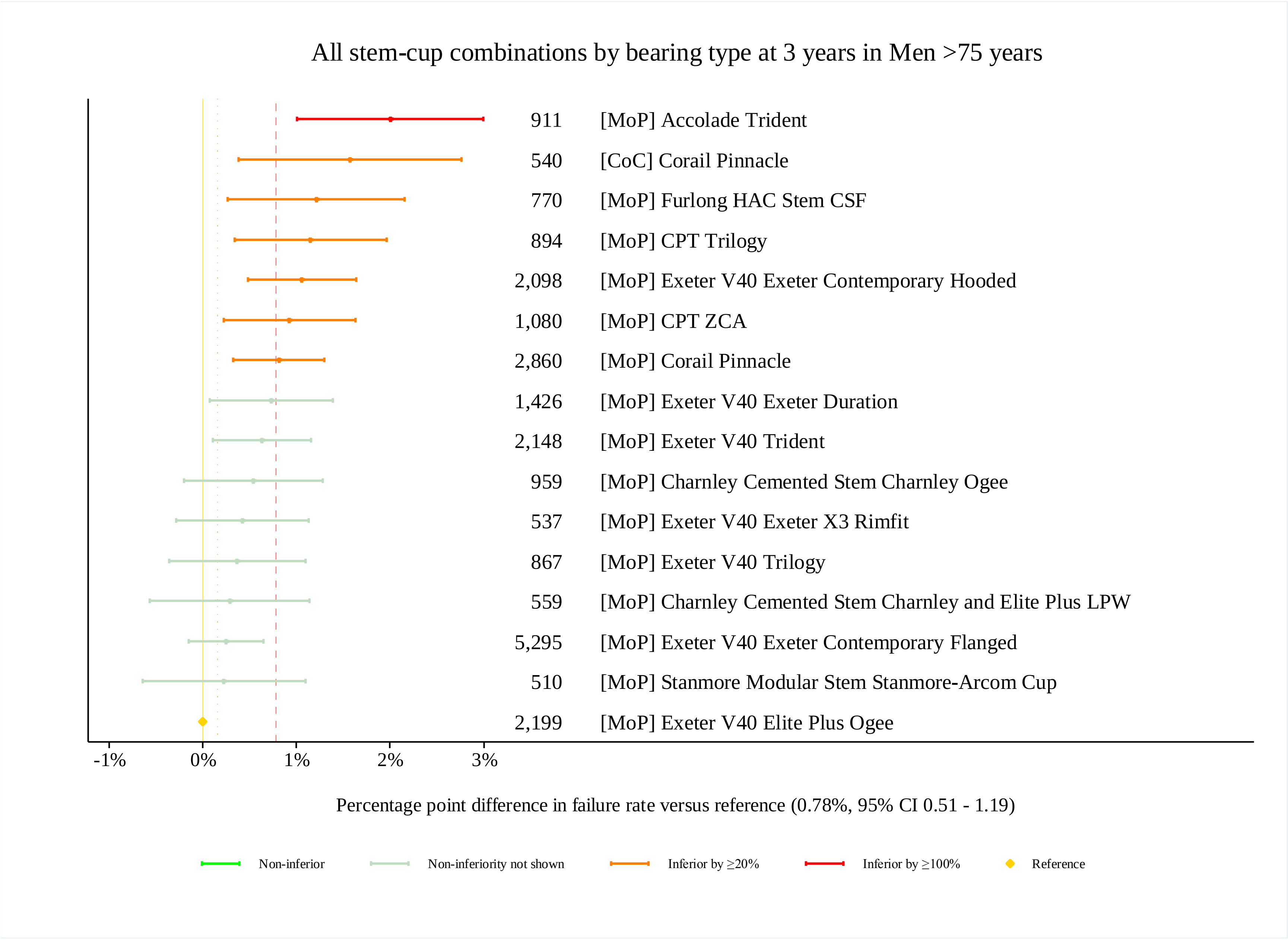

Supplementary Figure 3a: Difference in failure of implanted constructs compared to a contemporary reference at 5 years in men, using all stem-cup combinations with ≥500 procedures remaining at risk

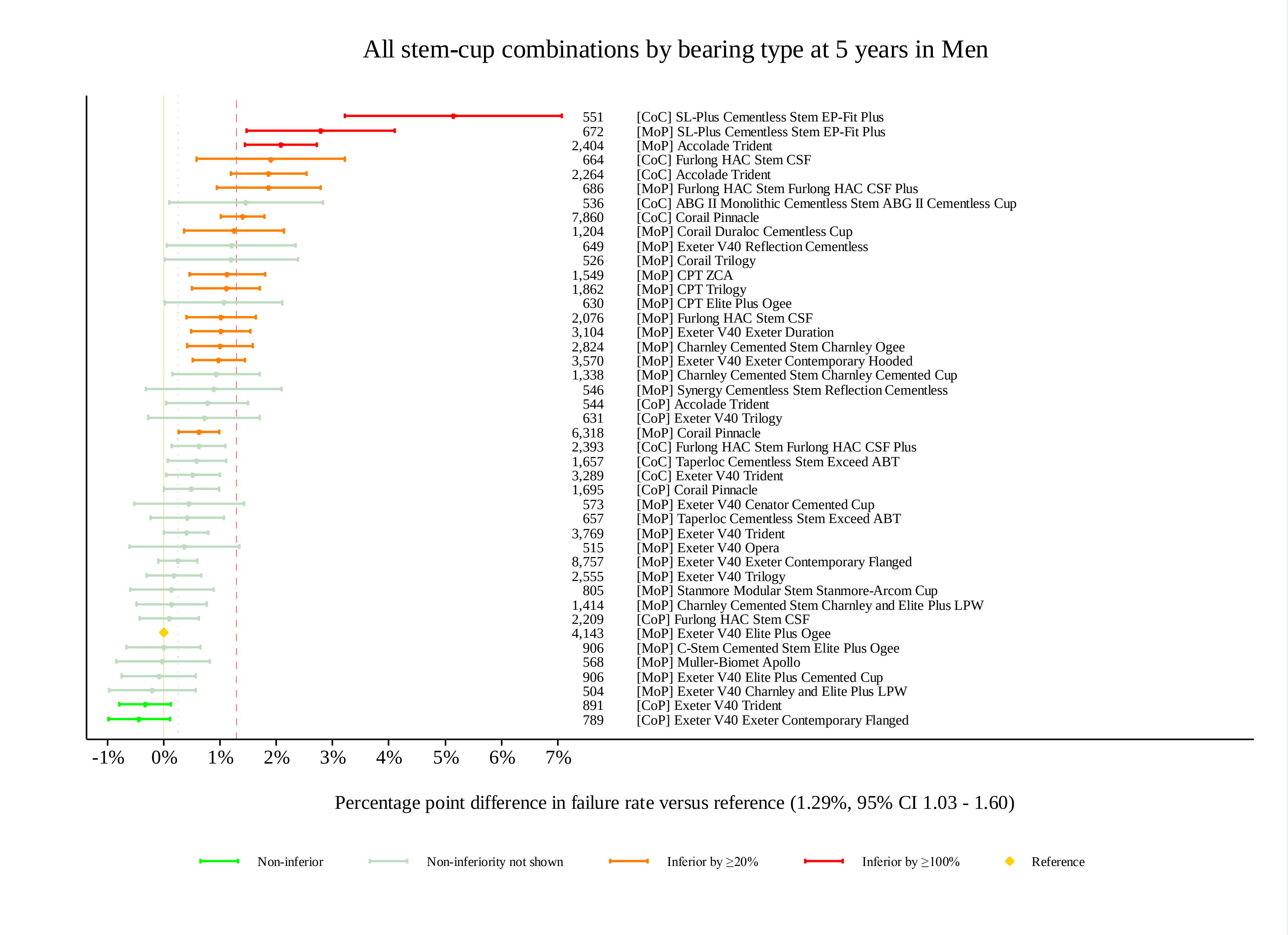

Supplementary Figure 3b: Difference in failure of implanted constructs compared to a contemporary reference at 5 years in men less than 55 years, using all stem-cup combinations with ≥500 procedures remaining at risk

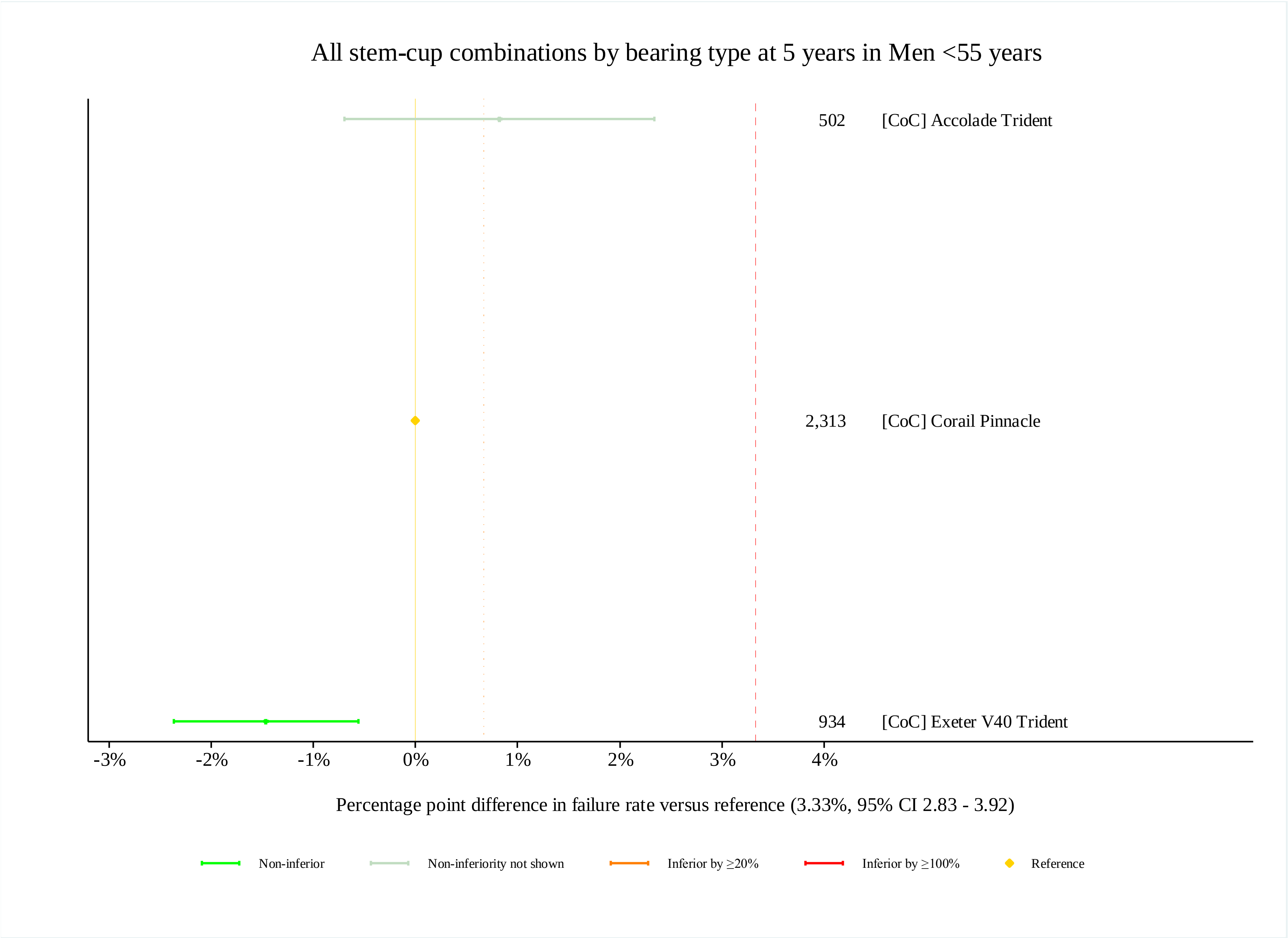

Supplementary Figure 3c: Difference in failure of implanted constructs compared to a contemporary reference at 5 years in men between 55 and 75 years, using all stem-cup combinations with ≥500 procedures remaining at risk

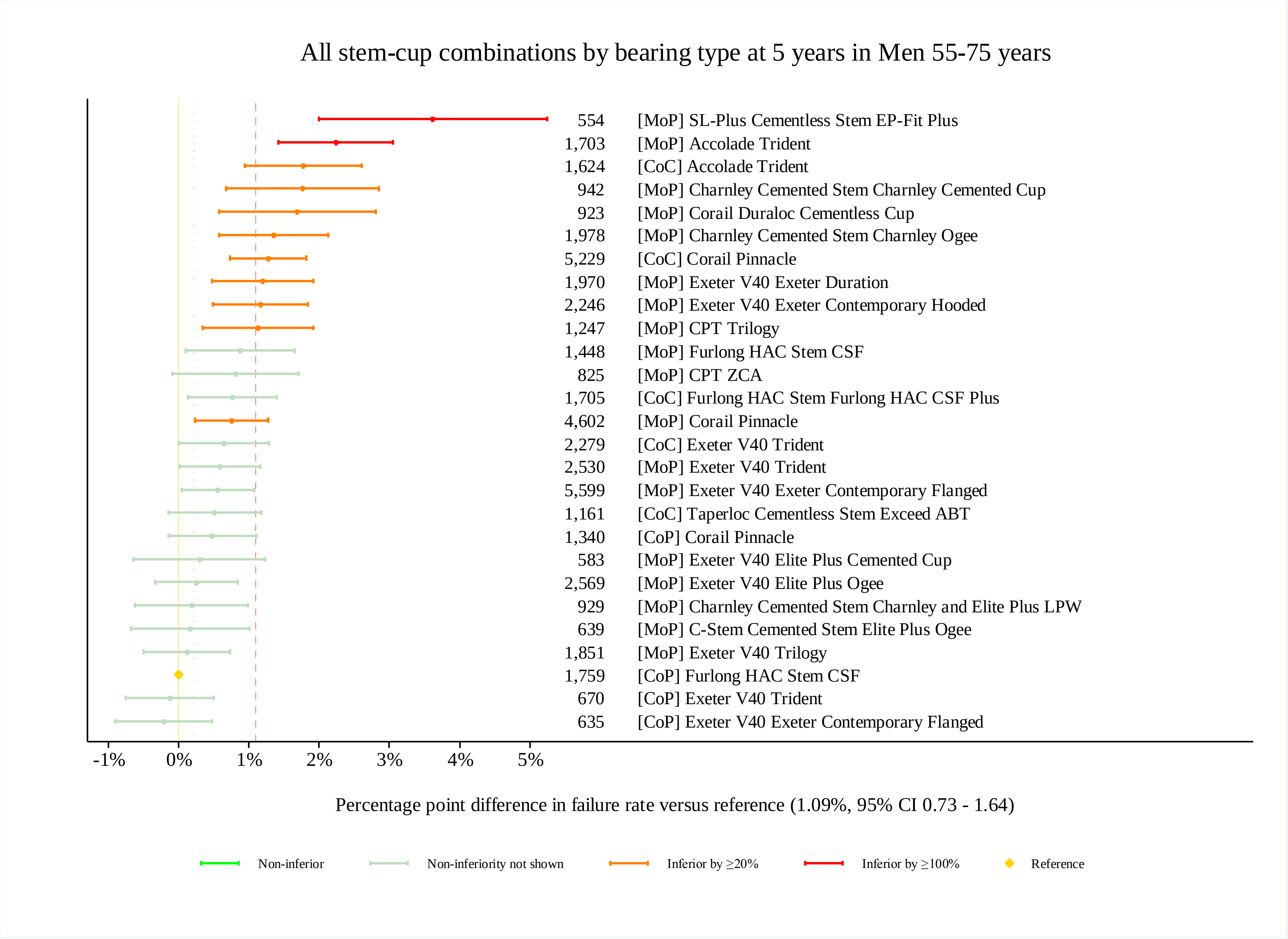

Supplementary Figure 3d: Difference in failure of implanted constructs compared to a contemporary reference at 5 years in men greater than 75 years, using all stem-cup combinations with ≥500 procedures remaining at risk

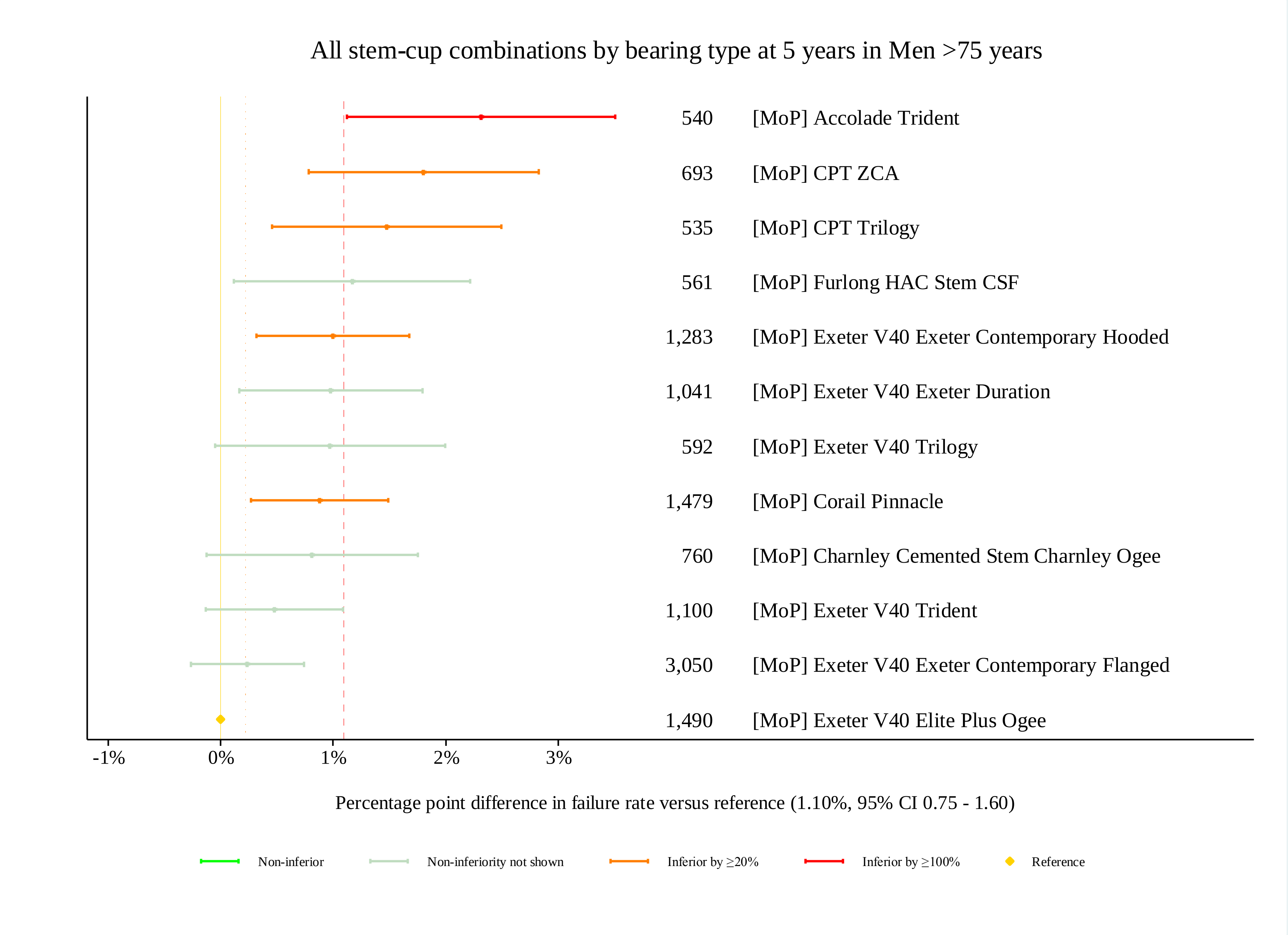

Supplementary Figure 4a: Difference in failure of implanted constructs compared to a contemporary reference at 7 years in men, using all stem-cup combinations with ≥500 procedures remaining at risk

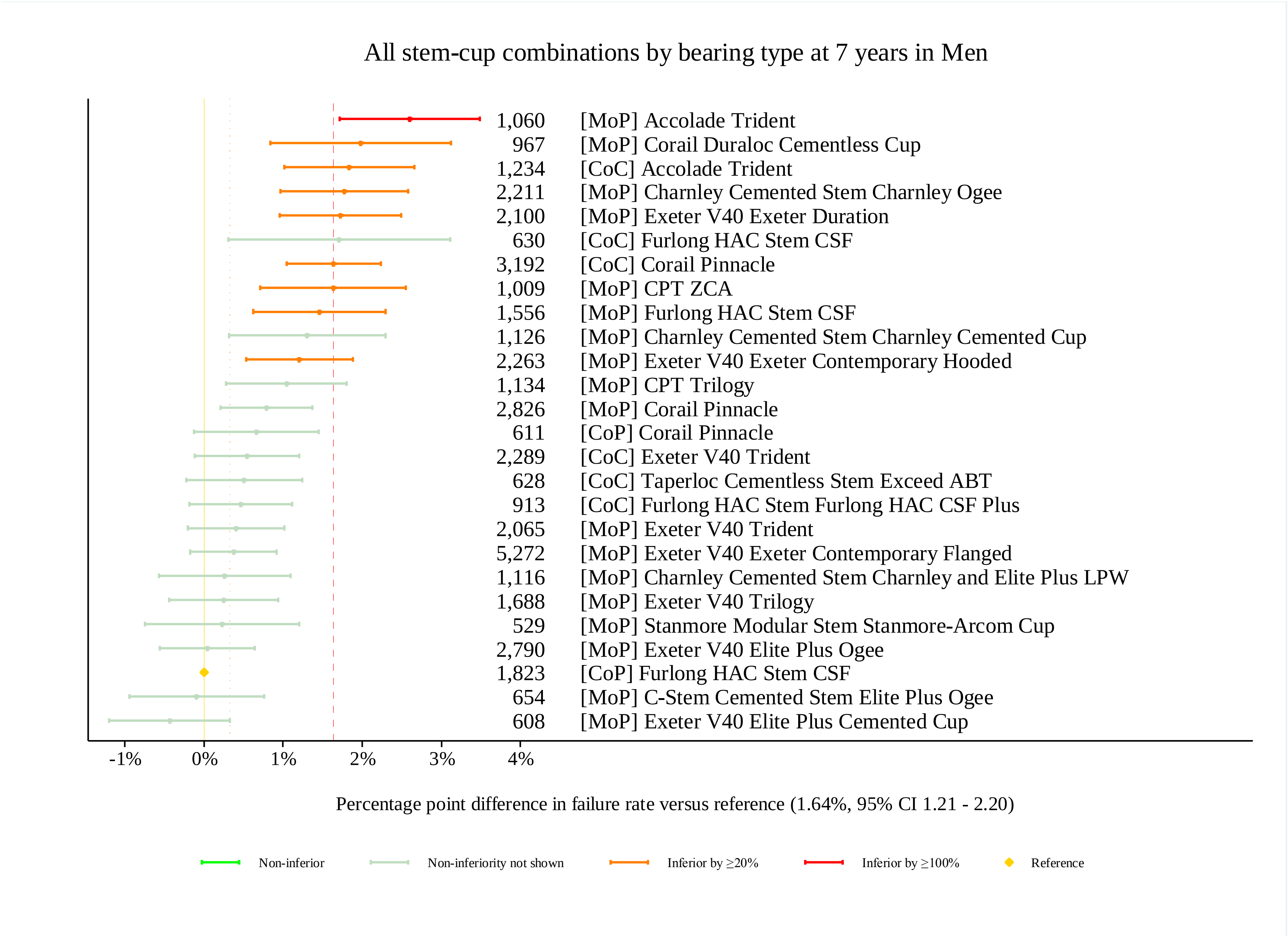

Supplementary Figure 4b: Difference in failure of implanted constructs compared to a contemporary reference at 7 years in men between 55 and 75 years, using all stem-cup combinations with ≥500 procedures remaining at risk

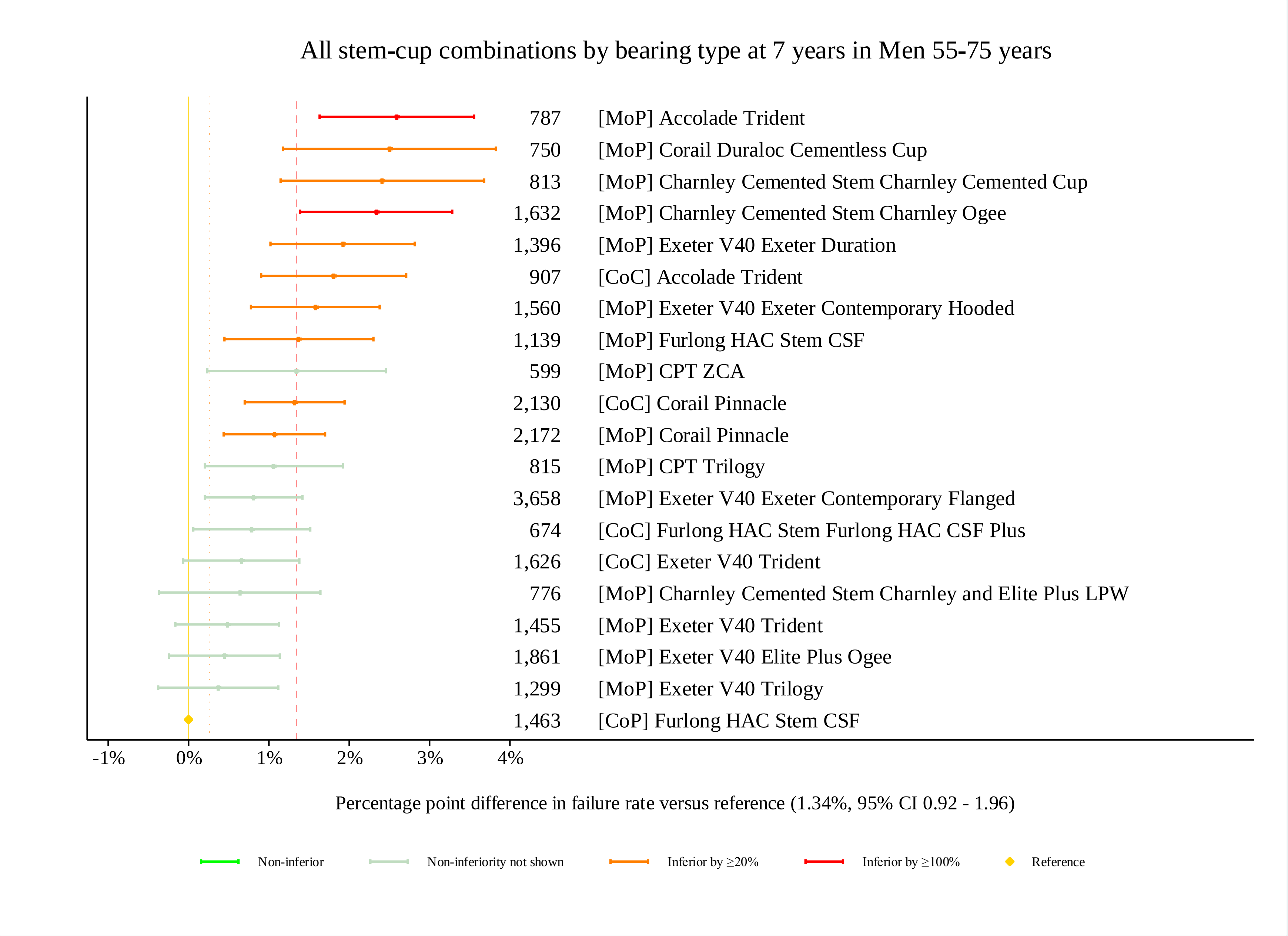

Supplementary Figure 4c: Difference in failure of implanted constructs compared to a contemporary reference at 7 years in men greater than 75 years, using all stem-cup combinations with ≥500 procedures remaining at risk

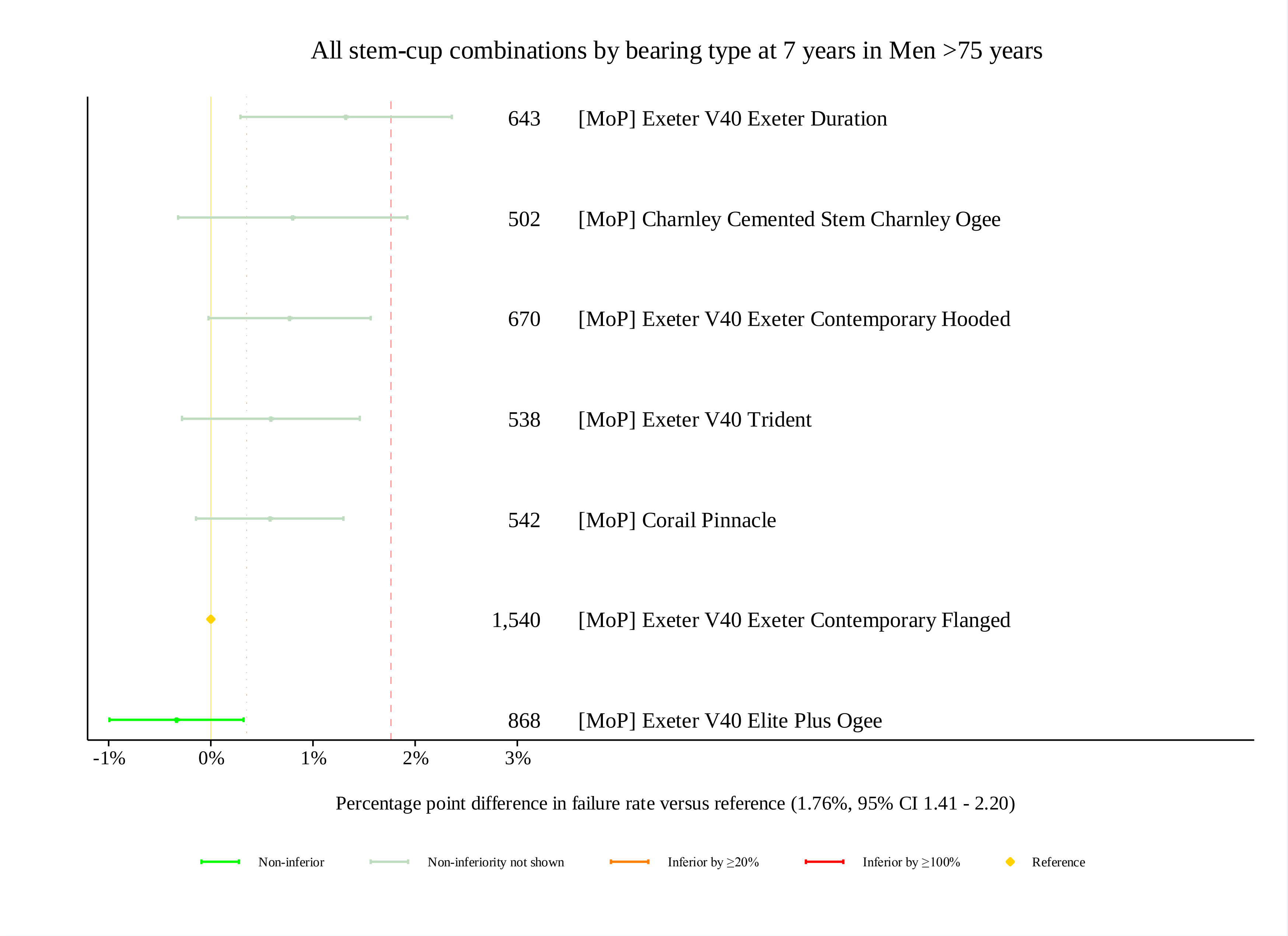

Supplementary Figure 5a: Difference in failure of implanted constructs compared to a contemporary reference at 10 years, using all stem-cup combinations with ≥500 procedures remaining at risk

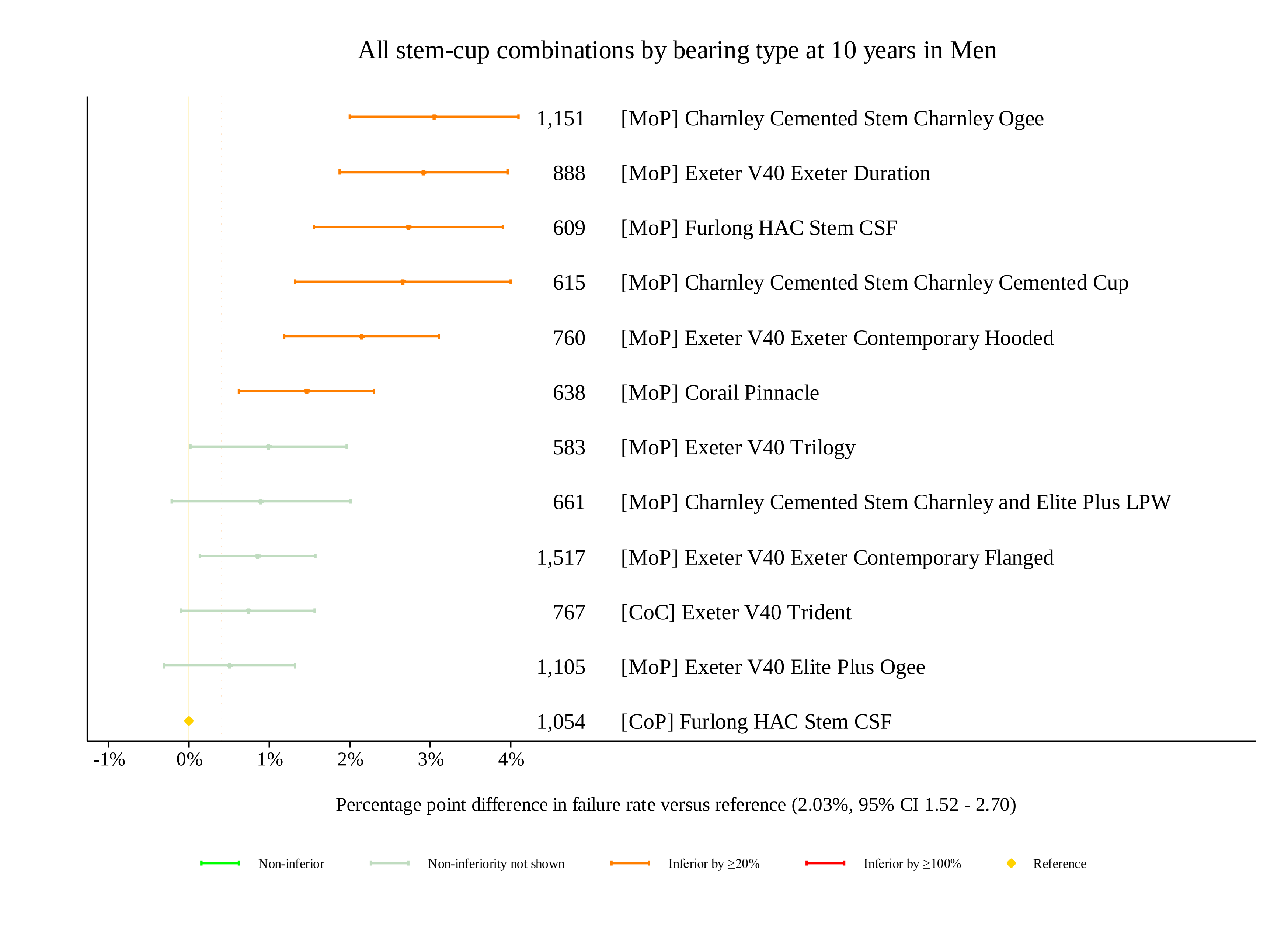

Supplementary Figure 5b: Difference in failure of implanted constructs compared to a contemporary reference at 10 years in men between 55 and 75 years, using all stem-cup combinations with ≥500 procedures remaining at risk

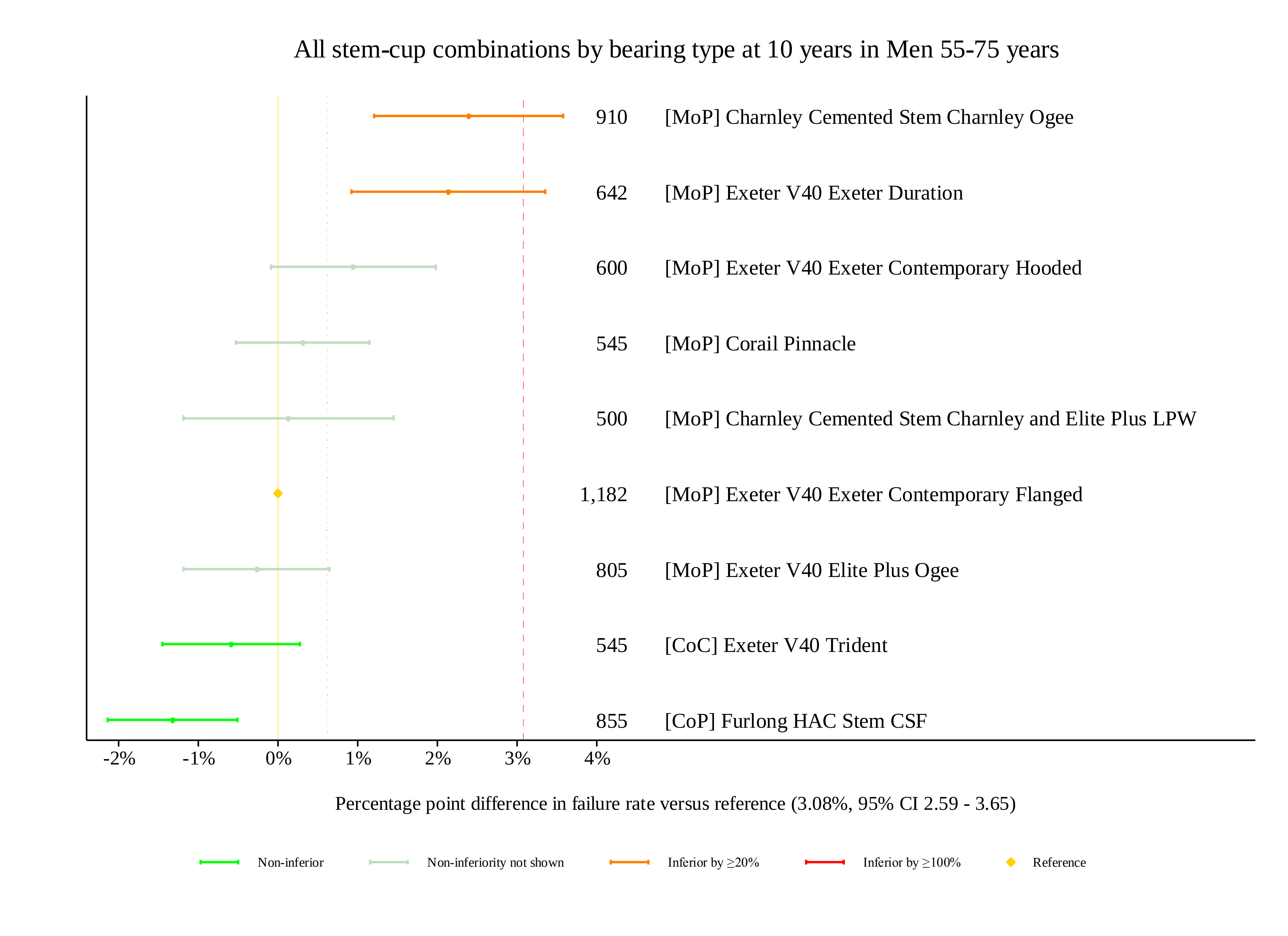

Supplementary Figure 6a: Difference in failure of implanted constructs compared to a contemporary reference at 3 years in women, using all stem-cup combinations with ≥500 procedures remaining at risk

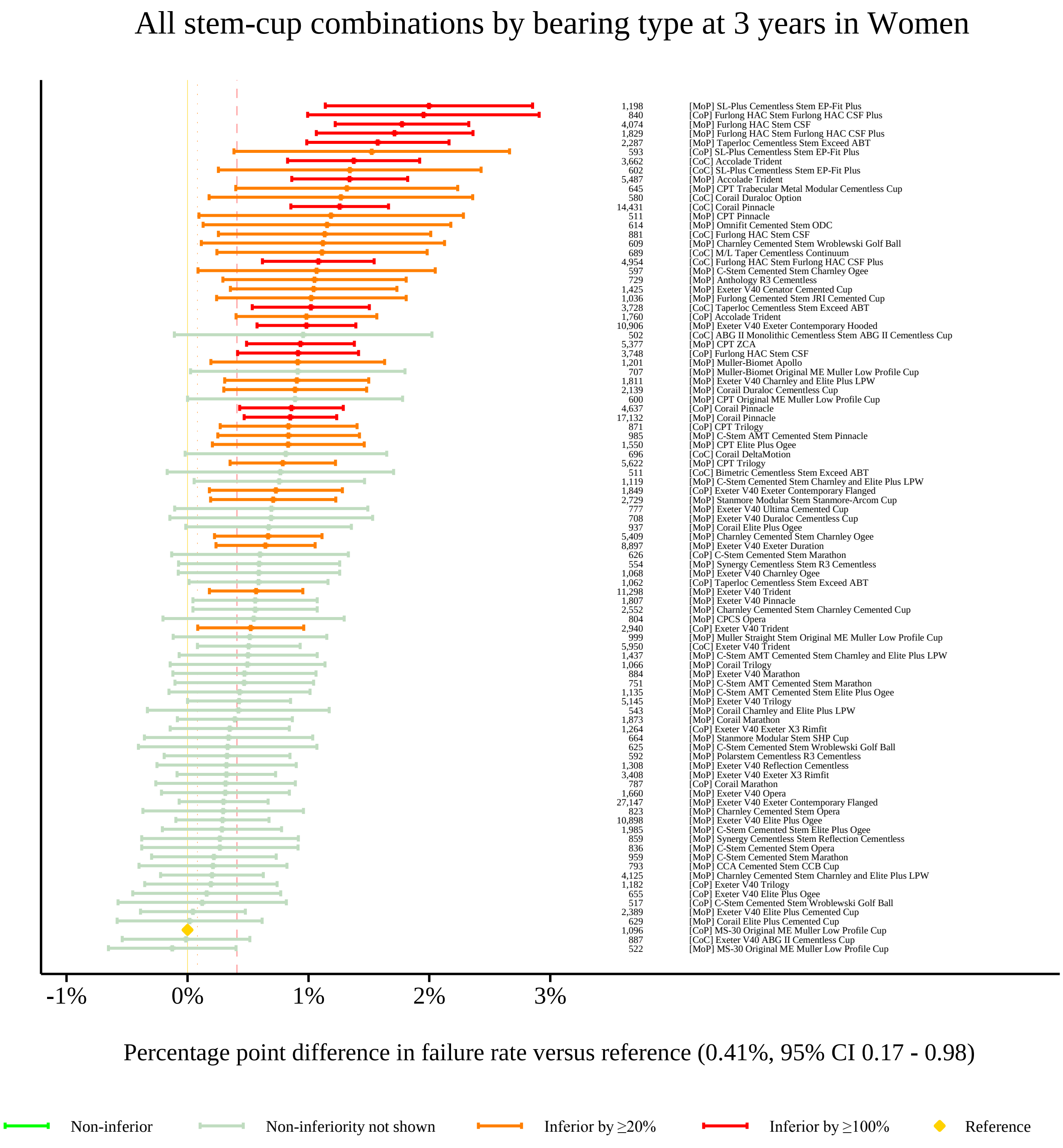

Supplementary Figure 6b: Difference in failure of implanted constructs compared to a contemporary reference at 3 years in women less than 55 years, using all stem-cup combinations with ≥500 procedures remaining at risk

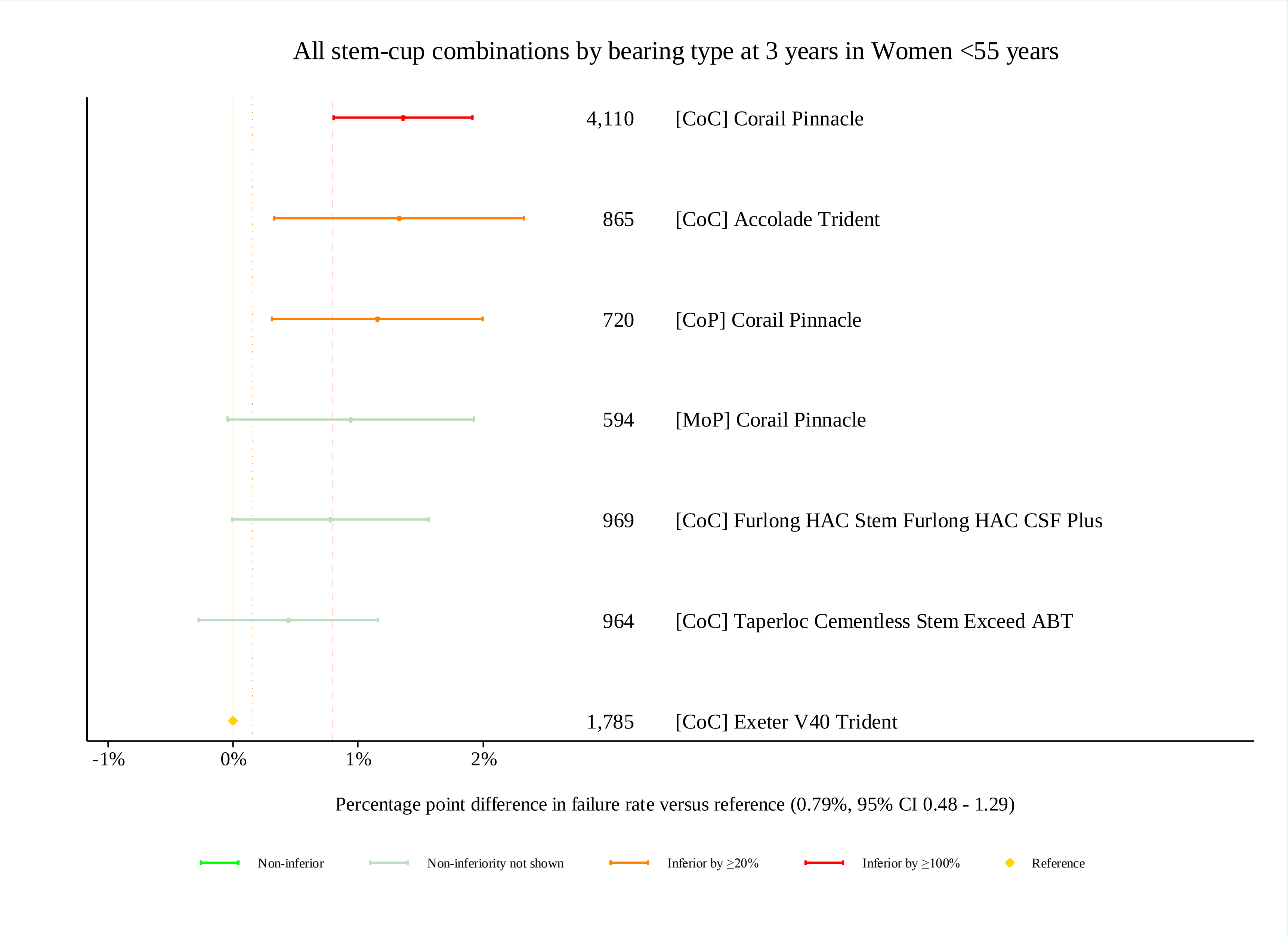

Supplementary Figure 6c: Difference in failure of implanted constructs compared to a contemporary reference at 3 years in women between 55 and 75 years, using all stem-cup combinations with ≥500 procedures remaining at risk

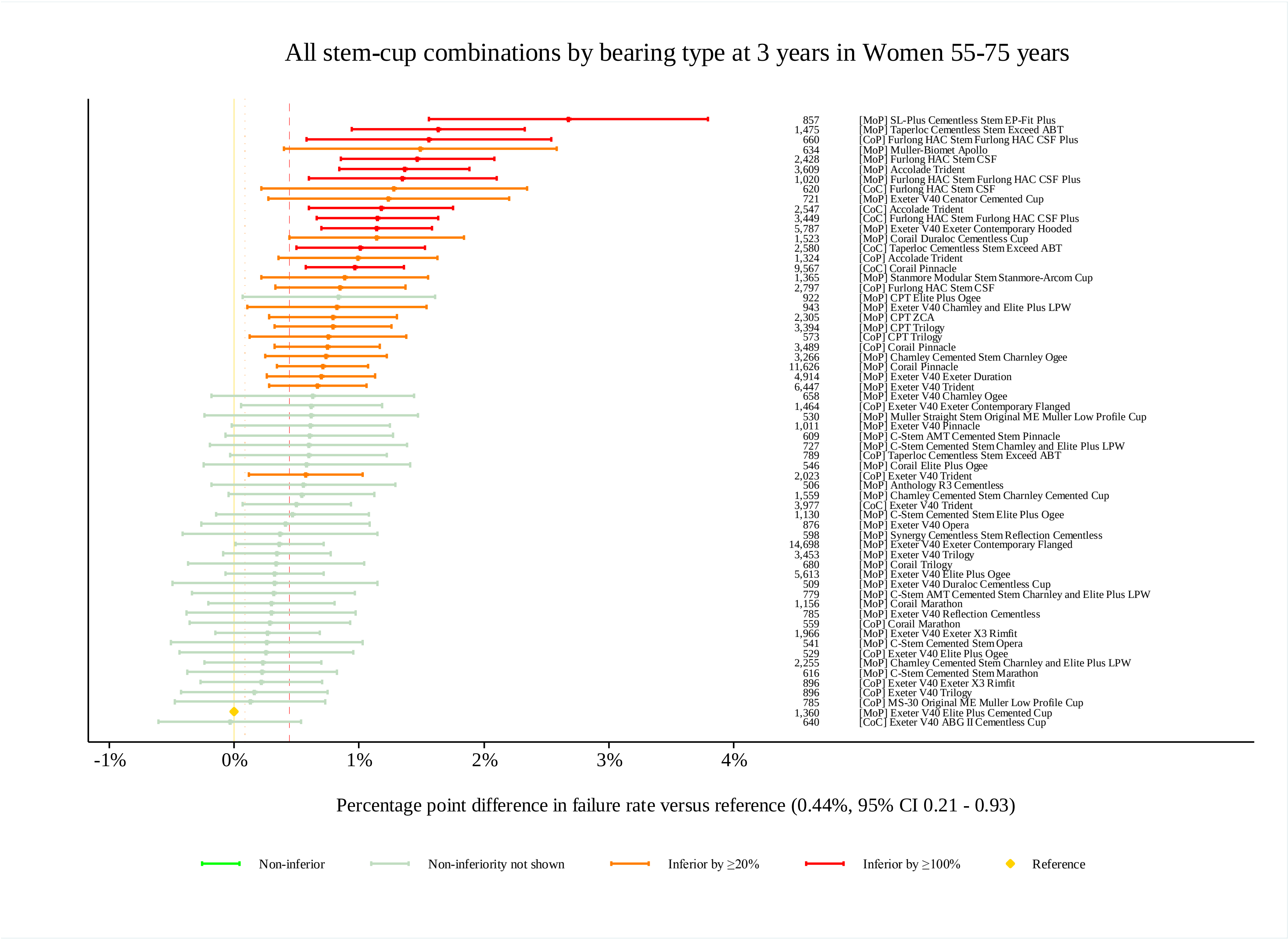

Supplementary Figure 6d: Difference in failure of implanted constructs compared to a contemporary reference at 3 years in women greater than 75 years, using all stem-cup combinations with ≥500 procedures remaining at risk

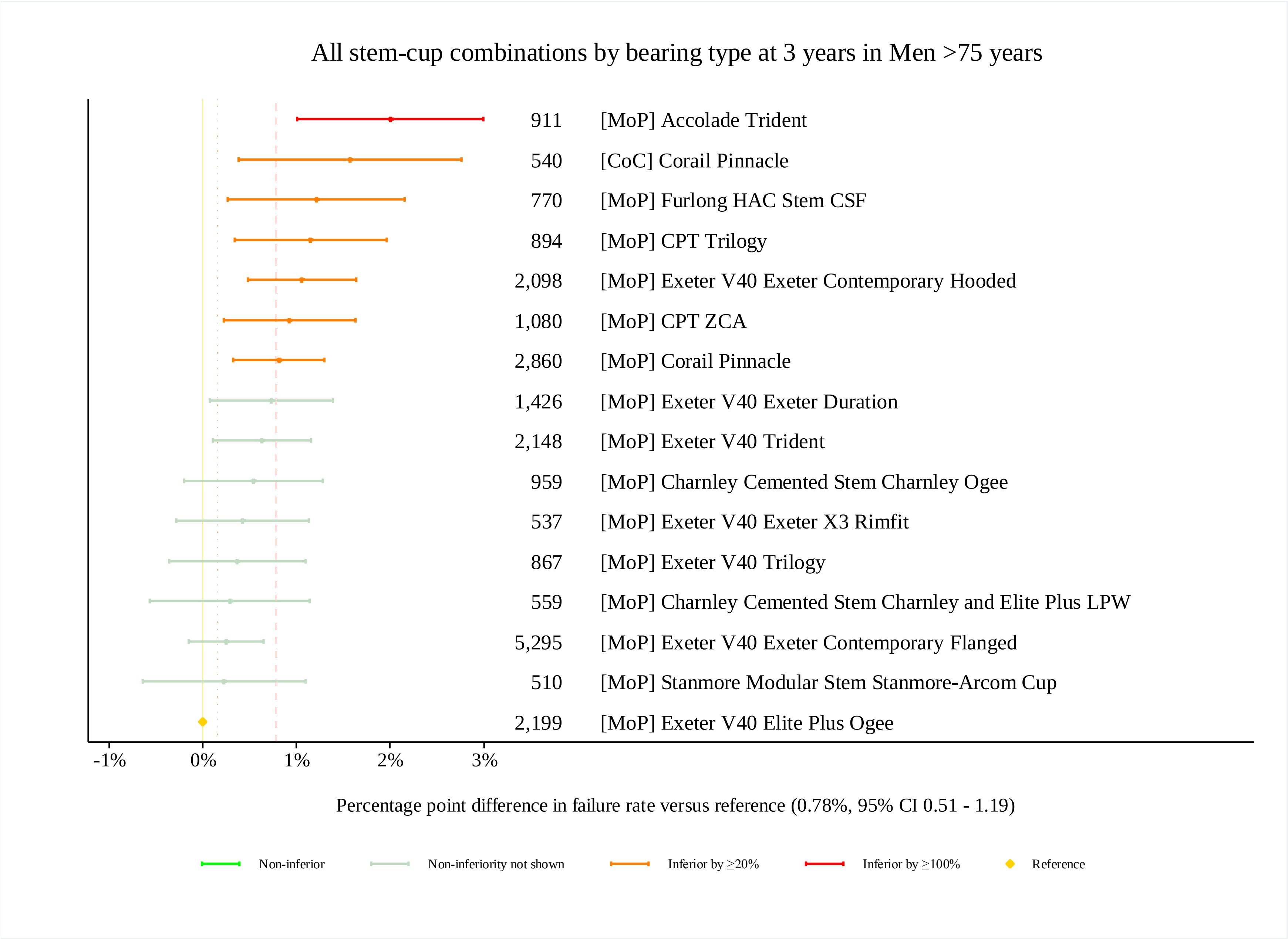

Supplementary Figure 7a: Difference in failure of implanted constructs compared to a contemporary reference at 5 years in women, using all stem-cup combinations with ≥500 procedures remaining at risk

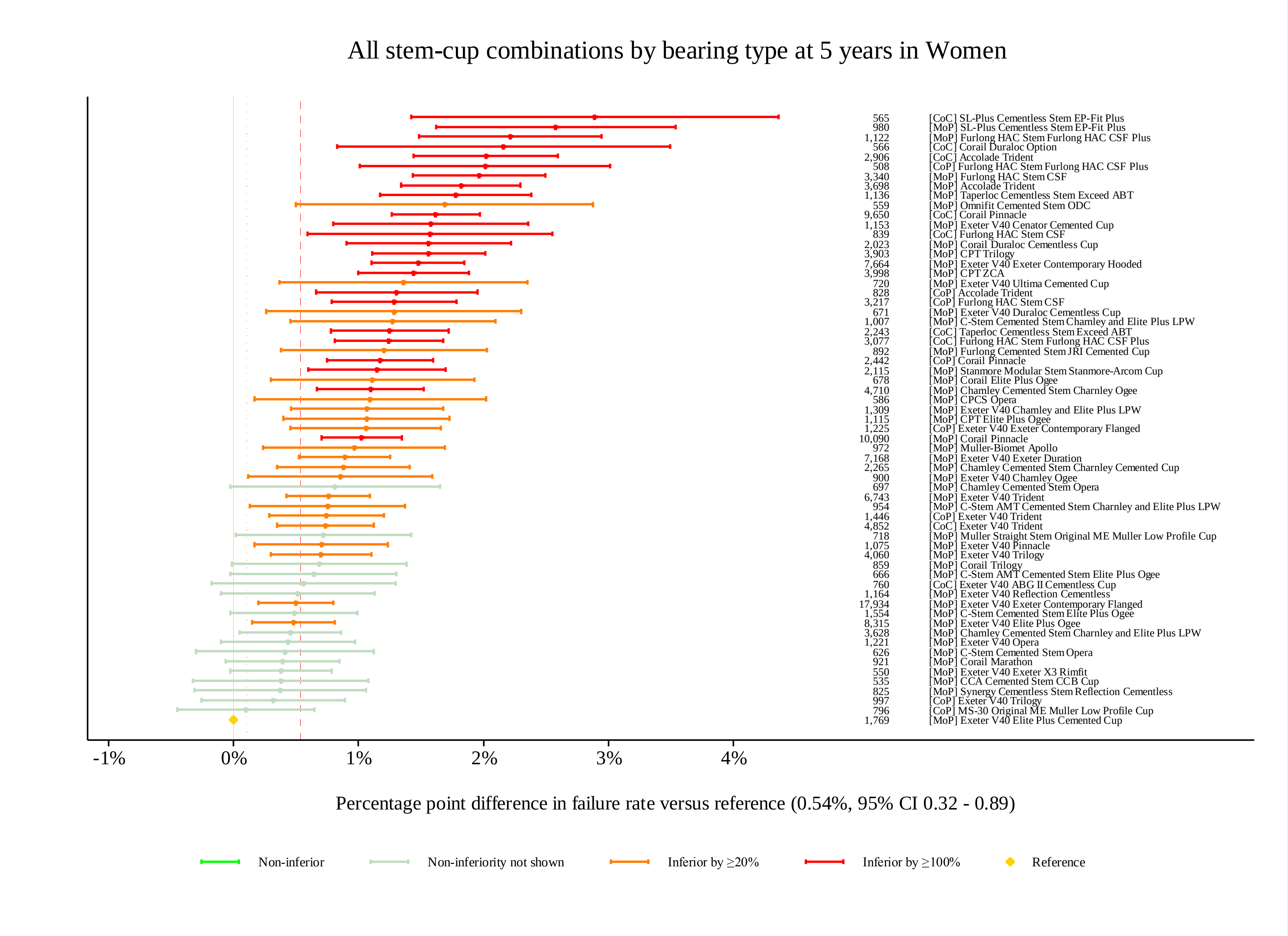

Supplementary Figure 7b: Difference in failure of implanted constructs compared to a contemporary reference at 5 years in women less than 55 years, using all stem-cup combinations with ≥500 procedures remaining at risk

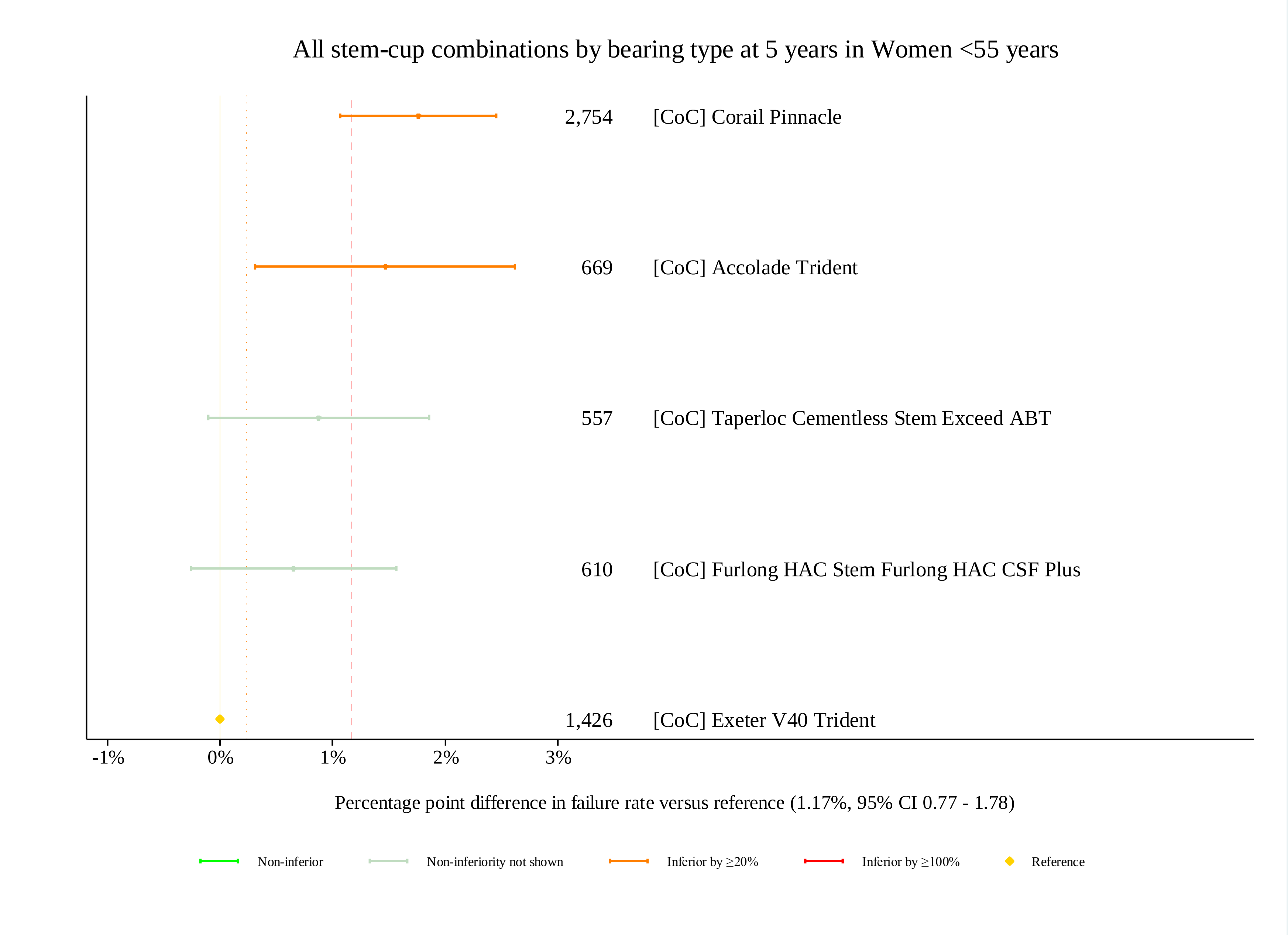

Supplementary Figure 7c: Difference in failure of implanted constructs compared to a contemporary reference at 5 years in women between 55 and 75 years, using all stem-cup combinations with ≥500 procedures remaining at risk

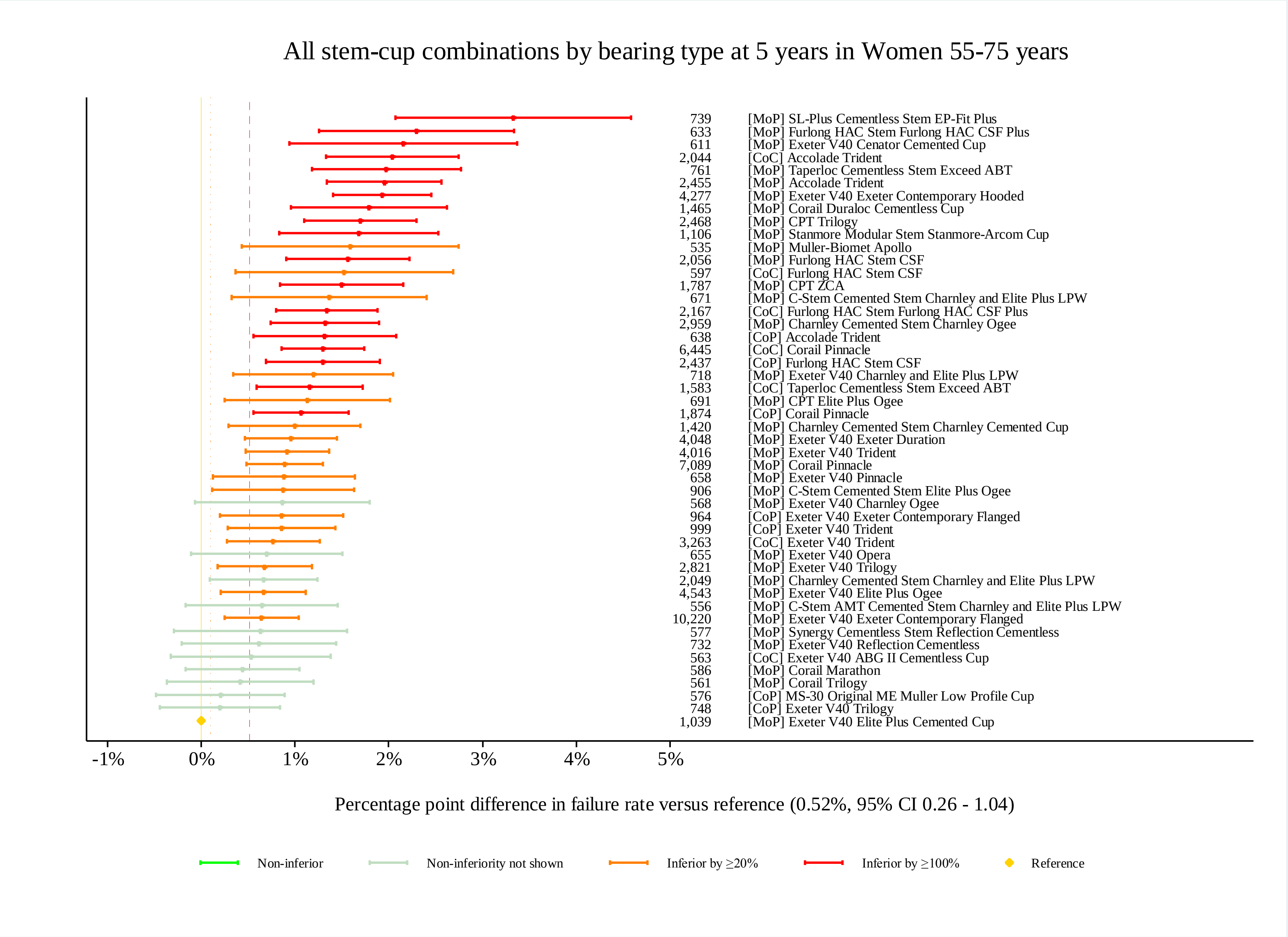

Supplementary Figure 7d: Difference in failure of implanted constructs compared to a contemporary reference at 5 years in women greater than 75 years, using all stem-cup combinations with ≥500 procedures remaining at risk

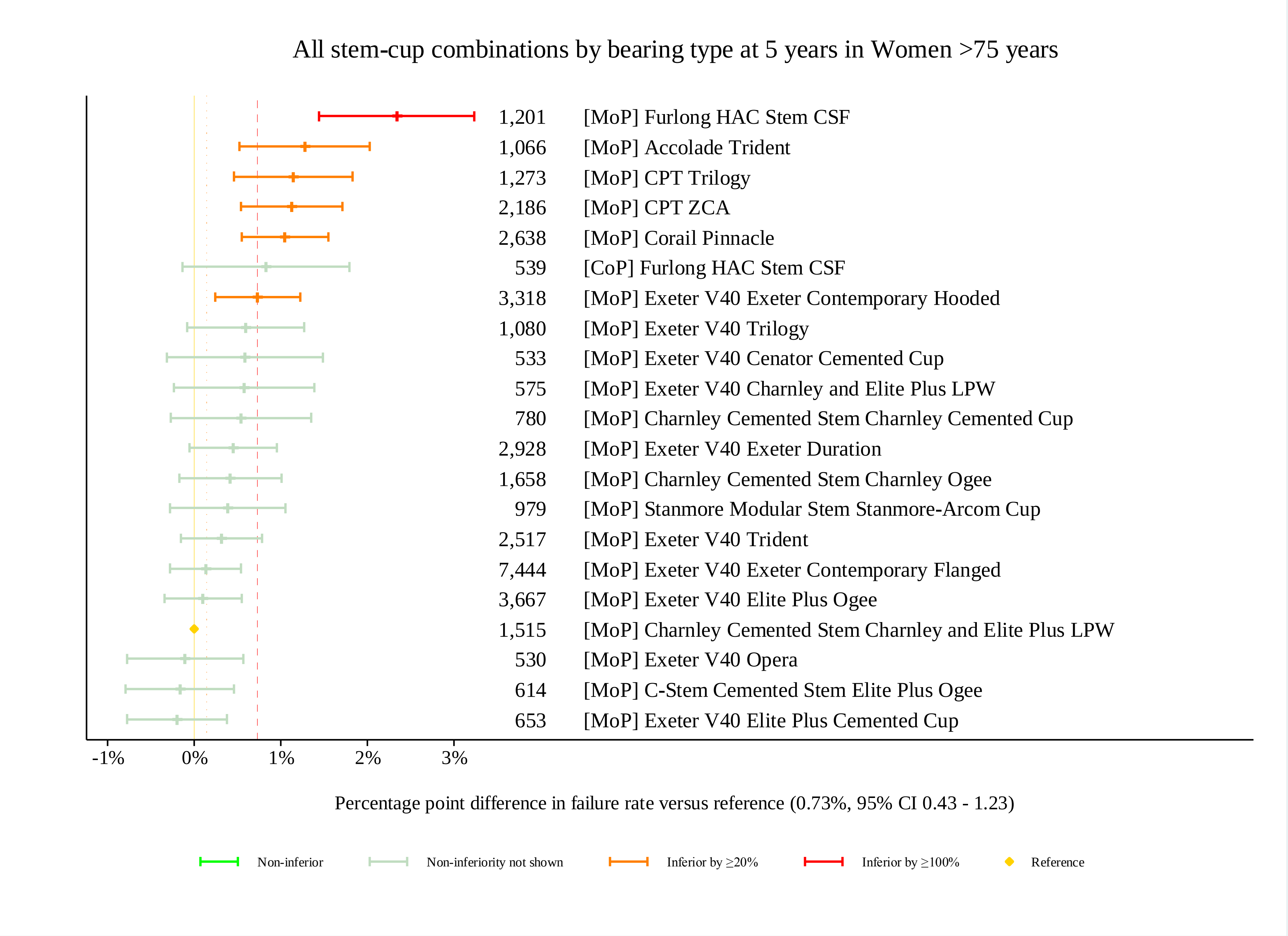

Supplementary Figure 8a: Difference in failure of implanted constructs compared to a contemporary reference at 7 years in women, using all stem-cup combinations with ≥500 procedures remaining at risk

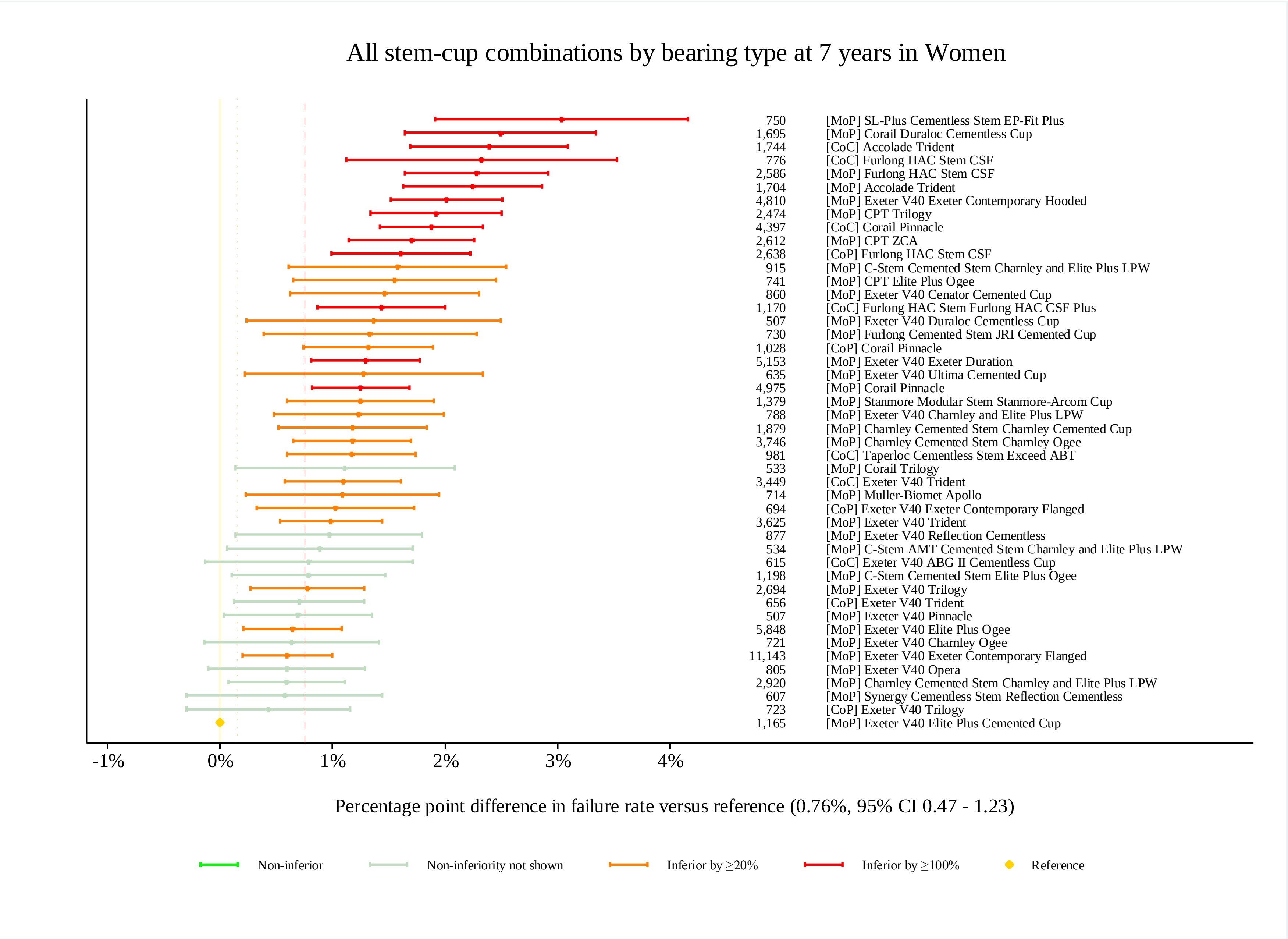

Supplementary Figure 8b: Difference in failure of implanted constructs compared to a contemporary reference at 7 years in women less than 55 years, using all stem-cup combinations with ≥500 procedures remaining at risk

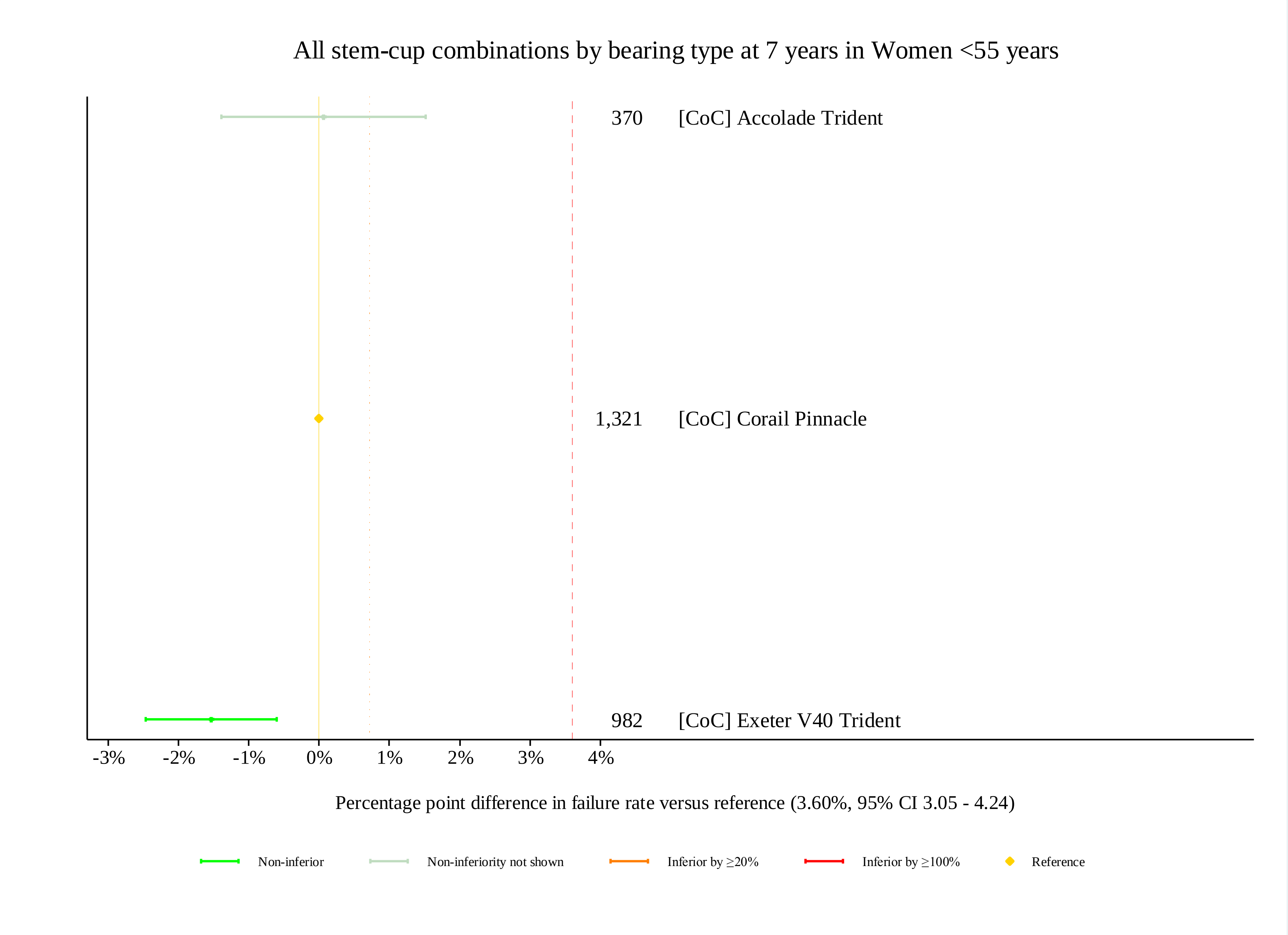

Supplementary Figure 8c: Difference in failure of implanted constructs compared to a contemporary reference at 7 years in women between 55 and 75 years, using all stem-cup combinations with ≥500 procedures remaining at risk

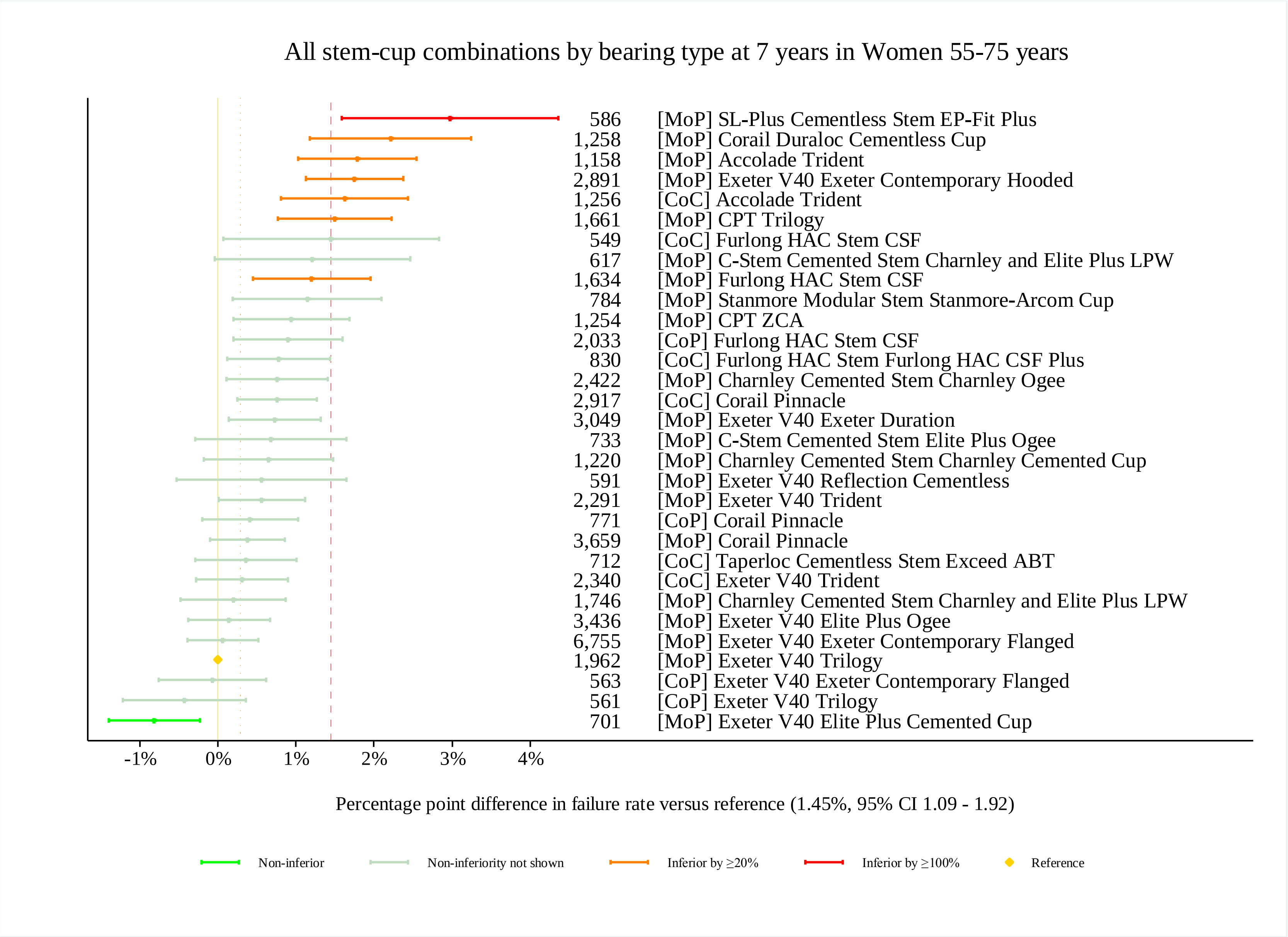

Supplementary Figure 8d: Difference in failure of implanted constructs compared to a contemporary reference at 7 years in women greater than 75 years, using all stem-cup combinations with ≥500 procedures remaining at risk

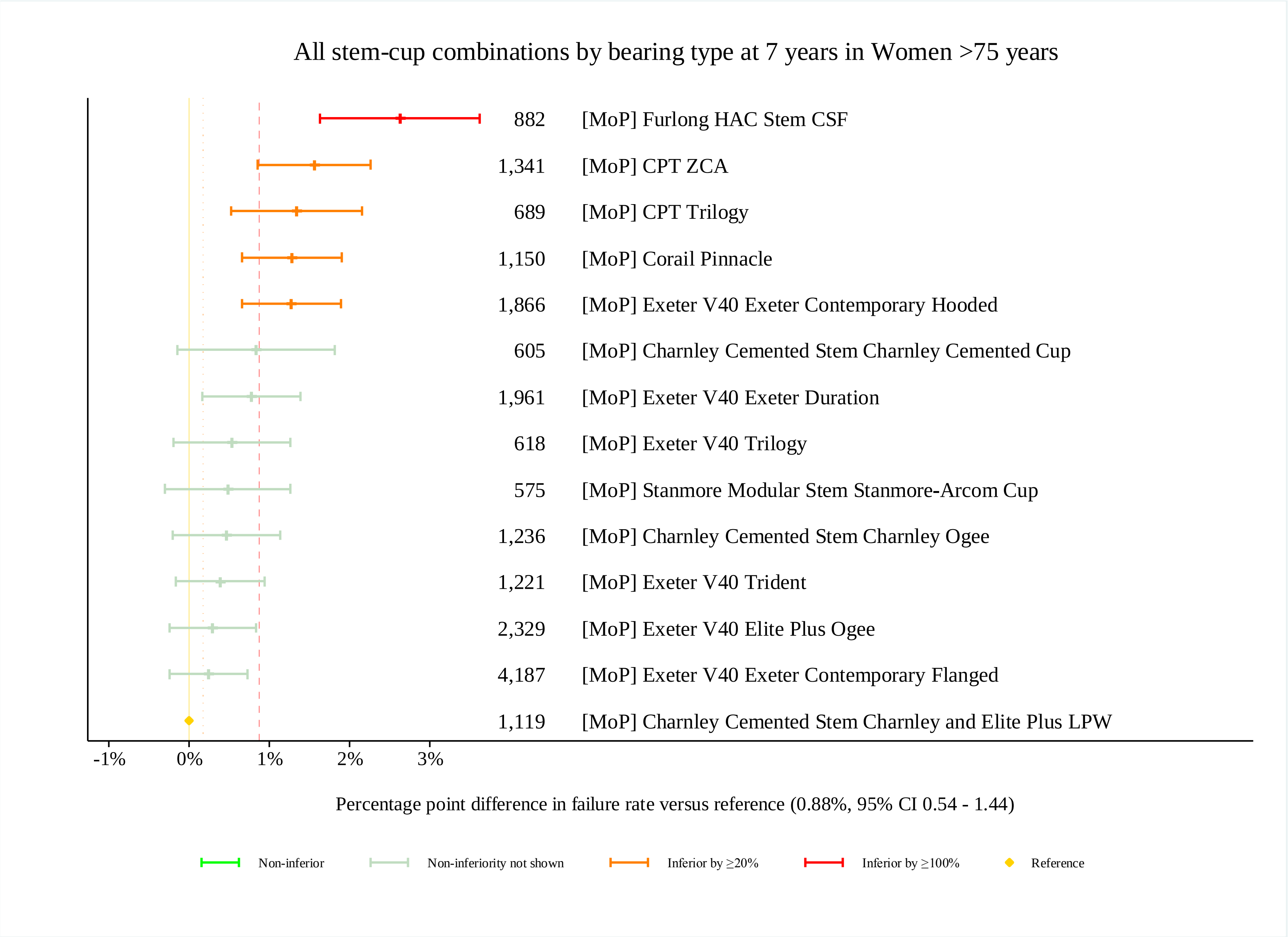

Supplementary Figure 9a: Difference in failure of implanted constructs compared to a contemporary reference at 10 years in women, using all stem-cup combinations with ≥500 procedures remaining at risk

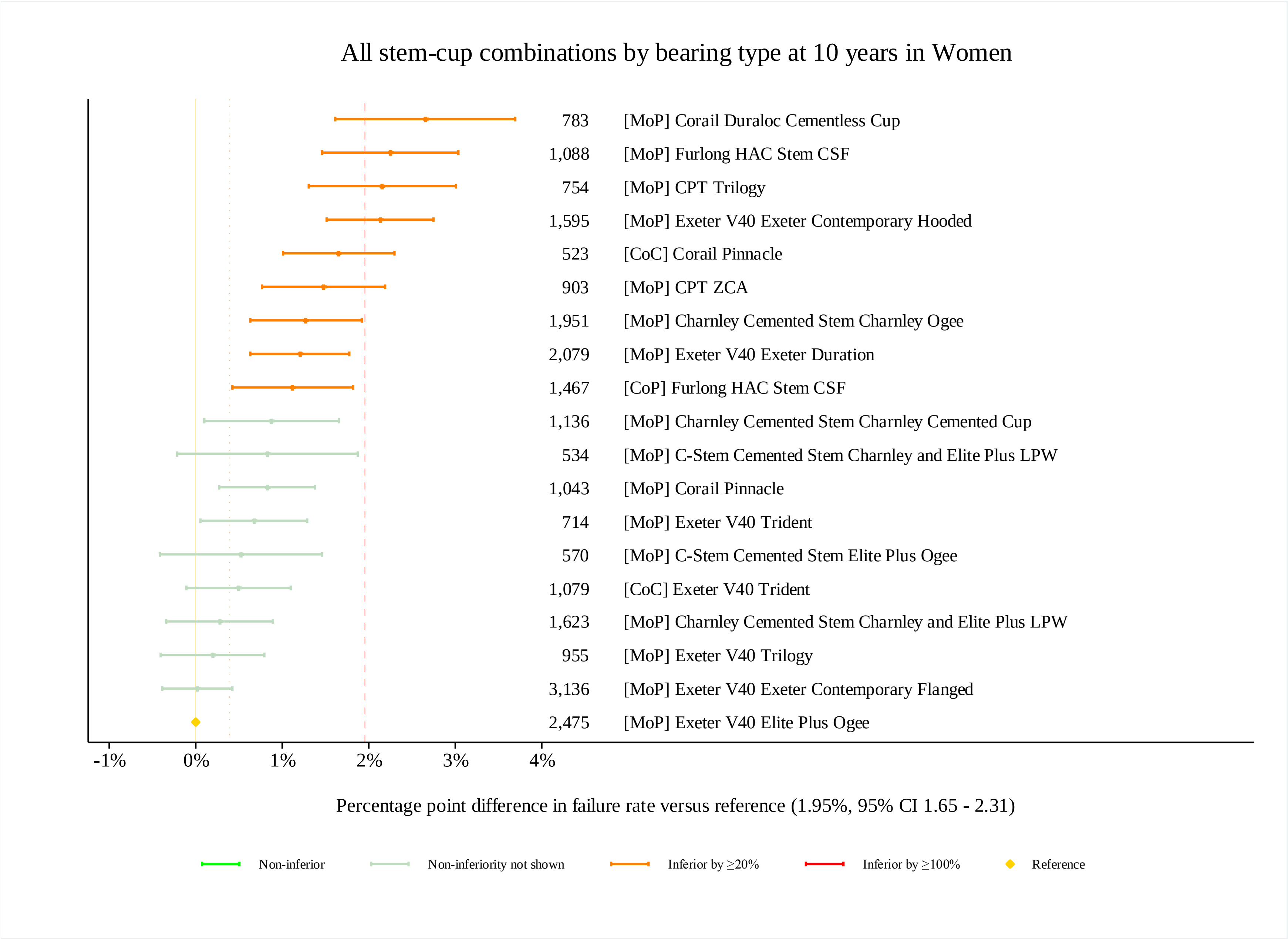

Supplementary Figure 9b: Difference in failure of implanted constructs compared to a contemporary reference at 10 years in women between 55 and 75 years, using all stem-cup combinations with ≥500 procedures remaining at risk

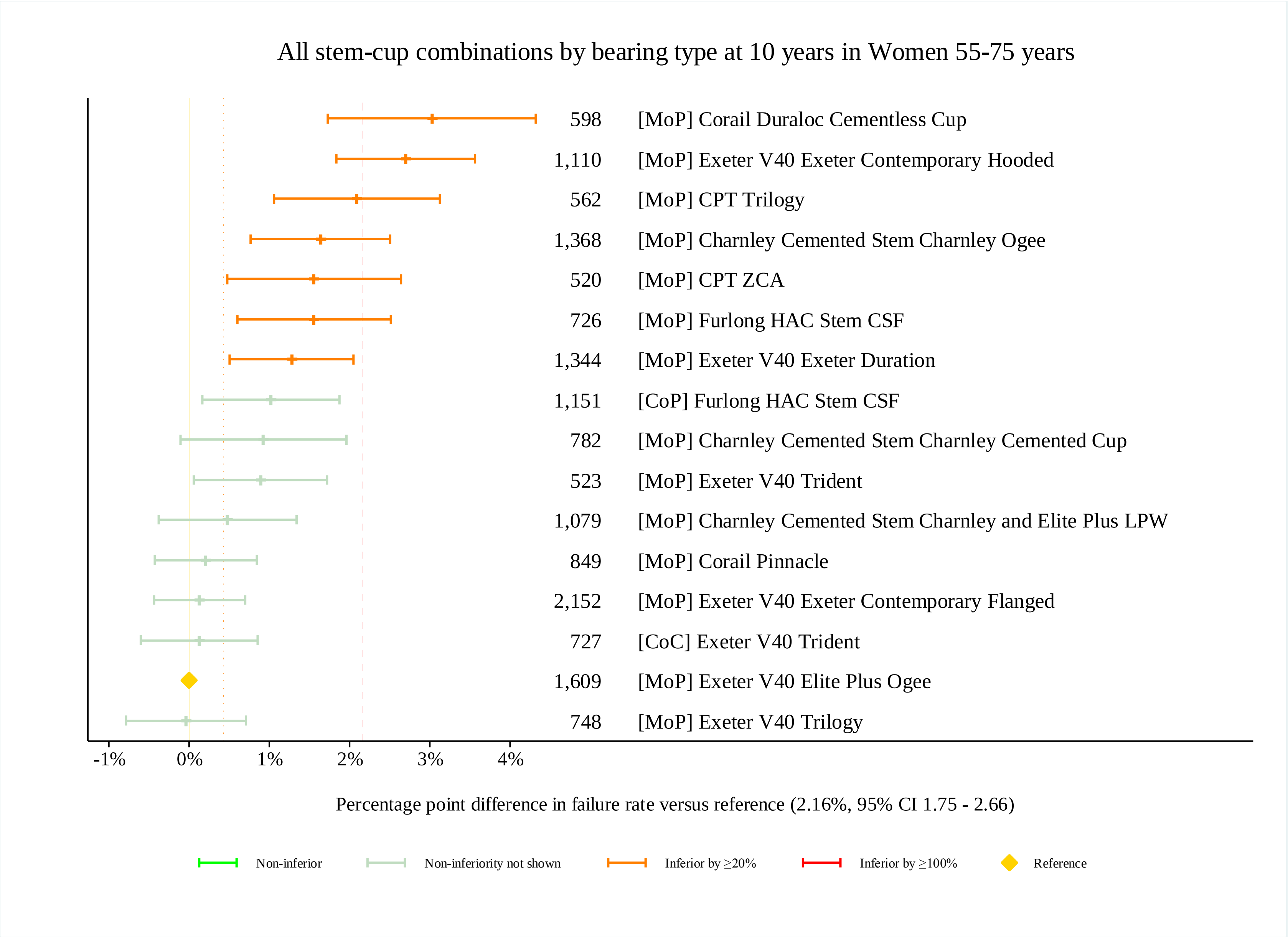

Supplementary table 1a: Difference in Kaplan-Meier failure estimate between a contemporary reference and implants with at least 250 at risk at 3 years since primary

Stem/cup brand	Number at risk	Cumulative failure (%)	Difference in failure (%)	95% CI	Equivalence status	p-value
[CoP] MS-30 Original ME Muller Low Profile Cup	1,554	0.39	[REFERENCE]			
[CoC] ABG II Monolithic Cementless Stem ABG II Cementless Cup	1,061	1.82	1.43	[0.59 , 2.27]	Inferior by ≥100%	0.001
[CoC] ABG II Monolithic Cementless Stem Trident	1,007	1.98	1.59	[0.70 , 2.48]	Inferior by ≥100%	<0.001
[CoC] AMIStem-H Versafit CC Trio	331	2.00	1.61	[0.44 , 2.77]	Inferior by ≥100%	0.007
[CoC] Accolade Trident	6,660	2.03	1.64	[1.20 , 2.08]	Inferior by ≥100%	<0.001
[CoC] Anthology R3 Cementless	327	1.13	0.74	[-0.24 , 1.72]	Non-inferiority not shown	0.140
[CoC] Bicontact Cementless Stem Plasmacup SC	346	1.01	0.62	[-0.41 , 1.65]	Non-inferiority not shown	0.237
[CoC] Bimetric Cementless Stem Exceed ABT	888	1.05	0.65	[-0.03 , 1.34]	Non-inferiority not shown	0.059
[CoC] C-Stem AMT Cemented Stem Pinnacle	770	0.83	0.44	[-0.18 , 1.06]	Non-inferiority not shown	0.168
[CoC] C-Stem Cemented Stem Pinnacle	342	0.54	0.15	[-0.65 , 0.95]	Non-inferiority not shown	0.715
[CoC] CPT Continuum	801	1.71	1.32	[0.52 , 2.13]	Inferior by ≥100%	0.001
[CoC] CPT Trilogy AB	525	1.12	0.73	[-0.21 , 1.67]	Non-inferiority not shown	0.127
[CoC] CPT Trilogy IT	314	1.14	0.74	[-0.13 , 1.62]	Non-inferiority not shown	0.095
[CoC] Charnley Modular Trilogy AB	252	0.39	0.00	[-0.82 , 0.81]	Non-inferiority not shown	0.996
[CoC] Corail Delta TT	726	2.10	1.71	[0.82 , 2.60]	Inferior by ≥100%	<0.001
[CoC] Corail DeltaMotion	1,147	1.40	1.01	[0.32 , 1.70]	Inferior by ≥20%	0.004
[CoC] Corail Duraloc Option	1,042	1.95	1.56	[0.68 , 2.43]	Inferior by ≥100%	<0.001
[CoC] Corail Pinnacle	27,047	1.79	1.40	[1.07 , 1.72]	Inferior by ≥100%	<0.001
[CoC] Corail Pinnacle Gription	452	2.41	2.02	[0.97 , 3.07]	Inferior by ≥100%	<0.001
[CoC] Corail Trinity	431	0.65	0.26	[-0.38 , 0.89]	Non-inferiority not shown	0.430
[CoC] Excia Cementless Plasmacup SC	888	1.20	0.81	[0.12 , 1.50]	Inferior by ≥20%	0.022
[CoC] Exeter V40 ABG II Cementless Cup	1,343	0.64	0.25	[-0.24 , 0.75]	Non-inferiority not shown	0.315
[CoC] Exeter V40 Trident	10,273	1.01	0.62	[0.27 , 0.96]	Inferior by ≥20%	<0.001
[CoC] Furlong Evolution Cementless Furlong HAC CSF Plus	597	1.37	0.98	[0.36 , 1.60]	Inferior by ≥20%	0.002
[CoC] Furlong HAC Stem CSF	1,568	2.08	1.69	[0.94 , 2.44]	Inferior by ≥100%	<0.001
[CoC] Furlong HAC Stem Furlong HAC CSF Plus	8,977	1.59	1.20	[0.83 , 1.57]	Inferior by ≥100%	<0.001
[CoC] Furlong HAC Stem Furlong Threaded	323	2.10	1.71	[0.14 , 3.28]	Inferior by ≥20%	0.033
[CoC] M/L Taper Cementless Continuum	1,393	1.63	1.24	[0.60 , 1.88]	Inferior by ≥100%	<0.001
[CoC] M/L Taper Cementless Trilogy IT	257	2.94	2.55	[0.93 , 4.18]	Inferior by ≥100%	0.002
[CoC] M/L Taper Kinectiv Cementless Continuum	258	3.39	3.00	[0.91 , 5.08]	Inferior by ≥100%	0.005
[CoC] Metafix Stem Trinity	813	1.29	0.90	[0.24 , 1.55]	Inferior by ≥20%	0.007
[CoC] Omnifit Cementless Stem Trident	486	1.99	1.60	[0.34 , 2.85]	Inferior by ≥20%	0.013
[CoC] Polarstem Cementless R3 Cementless	800	0.78	0.39	[-0.20 , 0.97]	Non-inferiority not shown	0.195
[CoC] S-Rom Cementless Stem Pinnacle	543	3.12	2.73	[1.38 , 4.08]	Inferior by ≥100%	<0.001
[CoC] SL-Plus Cementless Stem EP-Fit Plus	1,202	3.46	3.07	[2.03 , 4.12]	Inferior by ≥100%	<0.001
[CoC] SL-Plus Cementless Stem R3 Cementless	539	0.80	0.41	[-0.35 , 1.16]	Non-inferiority not shown	0.292
[CoC] SPS Modular April - Ceramic	593	1.50	1.11	[0.14 , 2.07]	Inferior by ≥20%	0.025
[CoC] Summit Cementless Stem Pinnacle	482	1.33	0.94	[0.02 , 1.87]	Non-inferiority not shown	0.046
[CoC] Synergy Cementless Stem R3 Cementless	280	0.96	0.57	[-0.41 , 1.54]	Non-inferiority not shown	0.257

For peer review only

[CoC] Taperloc Cementless Stem Exceed ABT	6,704	1.52	1.13	[0.75 , 1.51]	Inferior by ≥100%	<0.001
[CoC] Trilock BPS Pinnacle	341	2.06	1.67	[0.36 , 2.98]	Inferior by ≥20%	0.012
[CoC] miniHip Trinity	713	1.91	1.52	[0.64 , 2.41]	Inferior by ≥100%	0.001
[CoP] Accolade Trident	3,079	1.52	1.12	[0.67 , 1.58]	Inferior by ≥100%	<0.001
[CoP] C-Stem AMT Cemented Stem Marathon	321	0.80	0.41	[-0.34 , 1.15]	Non-inferiority not shown	0.285
[CoP] C-Stem AMT Cemented Stem Pinnacle	346	1.63	1.24	[0.36 , 2.12]	Inferior by ≥20%	0.006
[CoP] C-Stem Cemented Stem Elite Plus Ogee	607	0.57	0.18	[-0.45 , 0.80]	Non-inferiority not shown	0.583
[CoP] C-Stem Cemented Stem Marathon	1,150	1.03	0.64	[0.10 , 1.19]	Inferior by ≥20%	0.021
[CoP] C-Stem Cemented Stem Opera	785	0.63	0.23	[-0.38 , 0.85]	Non-inferiority not shown	0.457
[CoP] C-Stem Cemented Stem Wroblewski Golf Ball	913	0.69	0.30	[-0.29 , 0.88]	Non-inferiority not shown	0.320
[CoP] CLS Cementless Stem Trilogy	283	0.59	0.20	[-0.67 , 1.06]	Non-inferiority not shown	0.656
[CoP] CPT Trilogy	1,321	1.47	1.08	[0.59 , 1.58]	Inferior by ≥100%	<0.001
[CoP] CPT ZCA	349	0.39	-0.01	[-0.62 , 0.61]	Non-inferiority not shown	0.987
[CoP] Corail Charnley and Elite Plus LPW	627	1.72	1.33	[0.32 , 2.34]	Inferior by ≥20%	0.010
[CoP] Corail Duraloc Cementless Cup	301	4.40	4.01	[1.74 , 6.28]	Inferior by ≥100%	0.001
[CoP] Corail Elite Plus Cemented Cup	409	0.95	0.56	[-0.32 , 1.43]	Non-inferiority not shown	0.214
[CoP] Corail Elite Plus Ogee	464	1.70	1.31	[0.17 , 2.46]	Inferior by ≥20%	0.024
[CoP] Corail Marathon	1,309	1.12	0.73	[0.20 , 1.26]	Inferior by ≥20%	0.007
[CoP] Corail Pinnacle	8,130	1.21	0.82	[0.48 , 1.16]	Inferior by ≥100%	<0.001
[CoP] Corail Trabecular Metal Modular Cementless Cup	335	2.76	2.36	[0.85 , 3.88]	Inferior by ≥100%	0.002
[CoP] Corail Trident	300	0.26	-0.13	[-0.72 , 0.45]	Non-inferiority not shown	0.660
[CoP] Corail Trilogy	654	1.31	0.92	[0.02 , 1.81]	Non-inferiority not shown	0.045
[CoP] Excia Cementless Plasmacup SC	301	0.96	0.57	[-0.32 , 1.47]	Non-inferiority not shown	0.210
[CoP] Exeter V40 Charnley and Elite Plus LPW	674	1.59	1.20	[0.32 , 2.07]	Inferior by ≥20%	0.008
[CoP] Exeter V40 Elite Plus Ogee	1,190	0.70	0.31	[-0.20 , 0.81]	Non-inferiority not shown	0.231
[CoP] Exeter V40 Exeter Contemporary Flanged	2,995	0.99	0.60	[0.18 , 1.03]	Inferior by ≥20%	0.005
[CoP] Exeter V40 Exeter Contemporary Hooded	779	2.03	1.64	[0.70 , 2.57]	Inferior by ≥100%	0.001
[CoP] Exeter V40 Exeter Duration	775	0.66	0.27	[-0.33 , 0.88]	Non-inferiority not shown	0.378
[CoP] Exeter V40 Exeter X3 Rimfit	2,175	0.99	0.60	[0.18 , 1.02]	Inferior by ≥20%	0.005
[CoP] Exeter V40 Marathon	485	0.70	0.31	[-0.34 , 0.96]	Non-inferiority not shown	0.349
[CoP] Exeter V40 Pinnacle	542	1.14	0.75	[0.07 , 1.42]	Non-inferiority not shown	0.030
[CoP] Exeter V40 Trident	4,857	0.88	0.48	[0.14 , 0.83]	Inferior by ≥20%	0.006
[CoP] Exeter V40 Trilogy	1,945	1.05	0.66	[0.14 , 1.17]	Inferior by ≥20%	0.013
[CoP] Exeter V40 Tritanium	437	1.91	1.52	[0.60 , 2.45]	Inferior by ≥100%	0.001
[CoP] Furlong HAC Stem CSF	6,284	1.26	0.87	[0.48 , 1.26]	Inferior by ≥100%	<0.001
[CoP] Furlong HAC Stem Furlong HAC CSF Plus	1,538	1.84	1.45	[0.81 , 2.09]	Inferior by ≥100%	<0.001
[CoP] Furlong HAC Stem Furlong Threaded	388	0.50	0.11	[-0.64 , 0.86]	Non-inferiority not shown	0.778
[CoP] M/L Taper Cementless Continuum	391	2.79	2.40	[1.26 , 3.53]	Inferior by ≥100%	<0.001
[CoP] Muller Straight Stem Original ME Muller Low Profile Cup	364	1.22	0.83	[-0.19 , 1.85]	Non-inferiority not shown	0.110
[CoP] SL-Plus Cementless Stem Bicon-Plus	620	2.56	2.17	[0.93 , 3.40]	Inferior by ≥100%	0.001
[CoP] SL-Plus Cementless Stem EP-Fit Plus	958	1.89	1.50	[0.61 , 2.39]	Inferior by ≥100%	0.001
[CoP] SP II Cemented Stem Interplanta	310	0.00	- -	[-.-- , -.--]	No failures to date	
[CoP] Stanmore Modular Stem Stanmore-Arcom Cup	355	1.19	0.80	[-0.28 , 1.88]	Non-inferiority not shown	0.145
[CoP] Taperloc Cementless Stem Exceed ABT	1,879	1.07	0.68	[0.23 , 1.13]	Inferior by ≥20%	0.003
[CoP] Versys Cementless Stem Trilogy	363	1.07	0.68	[-0.40 , 1.76]	Non-inferiority not shown	0.220
[MoP] ABG II Monolithic Cementless Stem ABG II Cementless Cup	442	1.70	1.31	[0.10 , 2.51]	Inferior by ≥20%	0.033

[MoP] ABG II Monolithic Cementless Stem Trident	440	3.19	2.80	[1.18 , 4.42]	Inferior by ≥100%	0.001
[MoP] Accolade Trident	9,084	2.01	1.62	[1.23 , 2.02]	Inferior by ≥100%	<0.001
[MoP] Anthology R3 Cementless	1,155	1.76	1.36	[0.73 , 2.00]	Inferior by ≥100%	<0.001
[MoP] Bimetric Cementless Stem Mallory-Head Cementless Cup	359	2.05	1.66	[0.42 , 2.90]	Inferior by ≥100%	0.009
[MoP] C-Stem AMT Cemented Stem Charnley and Elite Plus LPW	2,153	1.15	0.76	[0.26 , 1.26]	Inferior by ≥20%	0.003
[MoP] C-Stem AMT Cemented Stem Elite Plus Cemented Cup	612	0.88	0.48	[-0.27 , 1.24]	Non-inferiority not shown	0.209
[MoP] C-Stem AMT Cemented Stem Elite Plus Ogee	1,608	0.77	0.38	[-0.09 , 0.85]	Non-inferiority not shown	0.116
[MoP] C-Stem AMT Cemented Stem Marathon	1,157	0.93	0.54	[0.07 , 1.00]	Non-inferiority not shown	0.023
[MoP] C-Stem AMT Cemented Stem Pinnacle	1,478	1.22	0.83	[0.35 , 1.31]	Inferior by ≥20%	0.001
[MoP] C-Stem Cemented Stem Charnley Ogee	847	1.31	0.92	[0.15 , 1.68]	Inferior by ≥20%	0.019
[MoP] C-Stem Cemented Stem Charnley and Elite Plus LPW	1,594	1.05	0.66	[0.10 , 1.23]	Inferior by ≥20%	0.022
[MoP] C-Stem Cemented Stem Duraloc Cementless Cup	535	2.10	1.71	[0.50 , 2.92]	Inferior by ≥100%	0.006
[MoP] C-Stem Cemented Stem Elite Plus Cemented Cup	650	0.56	0.17	[-0.45 , 0.79]	Non-inferiority not shown	0.590
[MoP] C-Stem Cemented Stem Elite Plus Ogee	3,208	0.88	0.49	[0.07 , 0.91]	Non-inferiority not shown	0.022
[MoP] C-Stem Cemented Stem Marathon	1,556	0.92	0.53	[0.05 , 1.00]	Non-inferiority not shown	0.029
[MoP] C-Stem Cemented Stem Opera	1,363	1.04	0.65	[0.05 , 1.25]	Non-inferiority not shown	0.033
[MoP] C-Stem Cemented Stem Pinnacle	417	1.56	1.17	[0.06 , 2.28]	Non-inferiority not shown	0.039
[MoP] C-Stem Cemented Stem Polarcup Cementless	268	0.71	0.32	[-0.70 , 1.35]	Non-inferiority not shown	0.539
[MoP] C-Stem Cemented Stem Trilogy	364	0.53	0.14	[-0.65 , 0.93]	Non-inferiority not shown	0.730
[MoP] C-Stem Cemented Stem Wroblewski Golf Ball	978	0.94	0.55	[-0.10 , 1.20]	Non-inferiority not shown	0.098
[MoP] CCA Cemented Stem CCB Cup	1,129	0.57	0.18	[-0.31 , 0.68]	Non-inferiority not shown	0.469
[MoP] CLS Cementless Stem Allofit	600	2.56	2.17	[0.93 , 3.40]	Inferior by ≥100%	0.001
[MoP] CLS Cementless Stem Trilogy	559	2.80	2.40	[1.06 , 3.75]	Inferior by ≥100%	<0.001
[MoP] CMK Modular Cemented Stem CMK Cemented Cup	462	0.41	0.02	[-0.62 , 0.67]	Non-inferiority not shown	0.943
[MoP] CPCS Opera	1,174	0.82	0.42	[-0.16 , 1.01]	Non-inferiority not shown	0.152
[MoP] CPCS Polarcup Cementless	400	0.49	0.09	[-0.64 , 0.83]	Non-inferiority not shown	0.801
[MoP] CPCS Reflection Cemented	382	1.02	0.63	[-0.14 , 1.40]	Non-inferiority not shown	0.107
[MoP] CPS Plus Cenator Cemented Cup	381	0.26	-0.13	[-0.72 , 0.46]	Non-inferiority not shown	0.662
[MoP] CPS Plus EP-Fit Plus	301	1.58	1.19	[-0.21 , 2.60]	Non-inferiority not shown	0.097
[MoP] CPS Plus Opera	490	1.55	1.16	[0.05 , 2.27]	Non-inferiority not shown	0.040
[MoP] CPT Allofit	686	0.55	0.16	[-0.38 , 0.69]	Non-inferiority not shown	0.561
[MoP] CPT Continuum	480	2.60	2.20	[1.33 , 3.08]	Inferior by ≥100%	<0.001
[MoP] CPT Elite Plus Ogee	2,410	1.38	0.99	[0.47 , 1.52]	Inferior by ≥100%	<0.001
[MoP] CPT Exceed	284	3.02	2.63	[0.66 , 4.59]	Inferior by ≥100%	0.009
[MoP] CPT Exeter Contemporary Flanged	386	2.10	1.71	[0.50 , 2.92]	Inferior by ≥100%	0.006
[MoP] CPT Opera	406	1.40	1.01	[-0.14 , 2.16]	Non-inferiority not shown	0.086
[MoP] CPT Original ME Muller Low Profile Cup	833	1.20	0.81	[0.09 , 1.53]	Inferior by ≥20%	0.028
[MoP] CPT Pinnacle	753	1.70	1.31	[0.38 , 2.24]	Inferior by ≥20%	0.006
[MoP] CPT Trabecular Metal Modular Cementless Cup	891	1.66	1.27	[0.50 , 2.03]	Inferior by ≥100%	0.001
[MoP] CPT Trilogy	8,372	1.37	0.98	[0.62 , 1.34]	Inferior by ≥100%	<0.001
[MoP] CPT Trilogy IT	450	2.89	2.50	[1.61 , 3.40]	Inferior by ≥100%	<0.001
[MoP] CPT ZCA	7,529	1.39	1.00	[0.63 , 1.37]	Inferior by ≥100%	<0.001
[MoP] Centrament Chirulen	378	0.24	-0.15	[-0.71 , 0.41]	Non-inferiority not shown	0.608
[MoP] Charnley Cemented Stem Charnley Cemented Cup	4,078	1.11	0.71	[0.29 , 1.14]	Inferior by ≥20%	0.001
[MoP] Charnley Cemented Stem Charnley Ogee	8,701	1.18	0.79	[0.43 , 1.15]	Inferior by ≥100%	<0.001
[MoP] Charnley Cemented Stem Charnley and Elite Plus LPW	5,770	0.72	0.32	[-0.03 , 0.68]	Non-inferiority not shown	0.075

For peer review only

[MoP] Charnley Cemented Stem Opera	1,229	0.85	0.46	[-0.12 , 1.04]	Non-inferiority not shown	0.119
[MoP] Charnley Cemented Stem Wroblewski Golf Ball	990	1.50	1.10	[0.32 , 1.89]	Inferior by ≥20%	0.006
[MoP] Charnley Modular Charnley and Elite Plus LPW	363	0.27	-0.12	[-0.73 , 0.49]	Non-inferiority not shown	0.702
[MoP] Corail Charnley and Elite Plus LPW	723	1.36	0.97	[0.18 , 1.76]	Inferior by ≥20%	0.017
[MoP] Corail Duraloc Cementless Cup	3,449	1.41	1.02	[0.54 , 1.50]	Inferior by ≥100%	<0.001
[MoP] Corail Elite Plus Cemented Cup	964	0.81	0.42	[-0.19 , 1.02]	Non-inferiority not shown	0.175
[MoP] Corail Elite Plus Ogee	1,407	1.22	0.83	[0.24 , 1.42]	Inferior by ≥20%	0.005
[MoP] Corail Exeter Contemporary Flanged	571	1.13	0.73	[-0.06 , 1.53]	Non-inferiority not shown	0.070
[MoP] Corail Marathon	2,848	1.05	0.66	[0.24 , 1.07]	Inferior by ≥20%	0.002
[MoP] Corail Pinnacle	28,253	1.37	0.98	[0.67 , 1.29]	Inferior by ≥100%	<0.001
[MoP] Corail Pinnacle Gription	382	1.54	1.15	[0.34 , 1.95]	Inferior by ≥20%	0.005
[MoP] Corail Trident	585	1.54	1.15	[0.33 , 1.96]	Inferior by ≥20%	0.006
[MoP] Corail Trilogy	1,791	1.14	0.74	[0.20 , 1.29]	Inferior by ≥20%	0.007
[MoP] Elite Plus Cemented Stem Charnley and Elite Plus LPW	331	1.66	1.27	[-0.08 , 2.62]	Non-inferiority not shown	0.065
[MoP] Elite Plus Cemented Stem Elite Plus Ogee	576	0.34	-0.05	[-0.60 , 0.50]	Non-inferiority not shown	0.848
[MoP] Excia Cementless Plasmacup SC	316	3.20	2.81	[1.00 , 4.62]	Inferior by ≥100%	0.002
[MoP] Exeter Elite Plus Ogee	282	0.33	-0.06	[-0.77 , 0.65]	Non-inferiority not shown	0.871
[MoP] Exeter V40 ABG II Cementless Cup	734	1.28	0.89	[0.05 , 1.73]	Non-inferiority not shown	0.038
[MoP] Exeter V40 Cenator Cemented Cup	2,129	1.40	1.01	[0.45 , 1.57]	Inferior by ≥100%	<0.001
[MoP] Exeter V40 Charnley Cemented Cup	314	0.87	0.48	[-0.55 , 1.51]	Non-inferiority not shown	0.358
[MoP] Exeter V40 Charnley Ogee	1,315	0.95	0.56	[-0.02 , 1.14]	Non-inferiority not shown	0.057
[MoP] Exeter V40 Charnley and Elite Plus LPW	2,477	1.25	0.86	[0.37 , 1.35]	Inferior by ≥20%	0.001
[MoP] Exeter V40 Duraloc Cementless Cup	1,061	1.46	1.06	[0.30 , 1.83]	Inferior by ≥20%	0.006
[MoP] Exeter V40 EP-Fit Plus	539	1.44	1.05	[0.02 , 2.08]	Non-inferiority not shown	0.046
[MoP] Exeter V40 Elite Plus Cemented Cup	3,599	0.58	0.19	[-0.18 , 0.56]	Non-inferiority not shown	0.314
[MoP] Exeter V40 Elite Plus Ogee	16,477	0.79	0.40	[0.08 , 0.71]	Inferior by ≥20%	0.014
[MoP] Exeter V40 Exceed	494	0.94	0.55	[-0.32 , 1.42]	Non-inferiority not shown	0.215
[MoP] Exeter V40 Exceed ABT	456	0.60	0.21	[-0.45 , 0.87]	Non-inferiority not shown	0.528
[MoP] Exeter V40 Exeter Contemporary Flanged	40,794	0.85	0.46	[0.15 , 0.76]	Inferior by ≥20%	0.003
[MoP] Exeter V40 Exeter Contemporary Hooded	16,021	1.53	1.14	[0.80 , 1.47]	Inferior by ≥100%	<0.001
[MoP] Exeter V40 Exeter Duration	12,935	1.23	0.84	[0.50 , 1.18]	Inferior by ≥100%	<0.001
[MoP] Exeter V40 Exeter X3 Rimfit	5,262	0.96	0.57	[0.23 , 0.92]	Inferior by ≥20%	0.001
[MoP] Exeter V40 Furlong HAC CSF Plus	504	0.35	-0.04	[-0.53 , 0.46]	Non-inferiority not shown	0.882
[MoP] Exeter V40 Marathon	1,300	1.05	0.66	[0.15 , 1.18]	Inferior by ≥20%	0.012
[MoP] Exeter V40 Opera	2,373	0.81	0.42	[-0.03 , 0.88]	Non-inferiority not shown	0.067
[MoP] Exeter V40 Pinnacle	2,590	1.18	0.79	[0.35 , 1.24]	Inferior by ≥20%	<0.001
[MoP] Exeter V40 R3 Cementless	461	1.23	0.84	[0.10 , 1.57]	Inferior by ≥20%	0.025
[MoP] Exeter V40 Reflection Cementless	2,035	1.12	0.73	[0.19 , 1.26]	Inferior by ≥20%	0.007
[MoP] Exeter V40 Trabecular Metal Modular Cementless Cup	411	1.22	0.83	[-0.12 , 1.77]	Non-inferiority not shown	0.086
[MoP] Exeter V40 Trabecular Metal Natural Cup	318	3.43	3.04	[1.11 , 4.97]	Inferior by ≥100%	0.002
[MoP] Exeter V40 Trident	17,802	1.10	0.71	[0.39 , 1.02]	Inferior by ≥20%	<0.001
[MoP] Exeter V40 Trilogy	8,482	0.92	0.53	[0.19 , 0.88]	Inferior by ≥20%	0.003
[MoP] Exeter V40 Tritanium	475	2.06	1.67	[0.71 , 2.63]	Inferior by ≥100%	0.001
[MoP] Exeter V40 Ultima Cemented Cup	1,139	1.56	1.17	[0.41 , 1.92]	Inferior by ≥100%	0.002
[MoP] Exeter V40 ZCA	274	0.36	-0.03	[-0.79 , 0.73]	Non-inferiority not shown	0.933
[MoP] Furlong Cemented Stem JRI Cemented Cup	1,478	1.38	0.99	[0.34 , 1.63]	Inferior by ≥20%	0.003

For peer review only

[MoP] Furlong HAC Stem CSF	6,640	2.07	1.68	[1.25 , 2.11]	Inferior by ≥100%	<0.001
[MoP] Furlong HAC Stem Furlong HAC CSF Plus	2,998	2.24	1.85	[1.33 , 2.37]	Inferior by ≥100%	<0.001
[MoP] Furlong HAC Stem Furlong Threaded	479	2.13	1.74	[0.46 , 3.02]	Inferior by ≥100%	0.008
[MoP] Furlong HAC Stem Trilogy	341	1.90	1.51	[0.09 , 2.94]	Inferior by ≥20%	0.038
[MoP] M/L Taper Cementless Allofit	543	1.27	0.88	[0.00 , 1.76]	Non-inferiority not shown	0.049
[MoP] M/L Taper Cementless Continuum	612	1.40	1.01	[0.28 , 1.74]	Inferior by ≥20%	0.007
[MoP] M/L Taper Cementless Trilogy	475	2.08	1.69	[0.63 , 2.74]	Inferior by ≥100%	0.002
[MoP] M/L Taper Cementless Trilogy IT	270	1.86	1.47	[0.46 , 2.47]	Inferior by ≥100%	0.004
[MoP] MS-30 Allofit	314	0.57	0.17	[-0.66 , 1.01]	Non-inferiority not shown	0.683
[MoP] MS-30 Original ME Muller Low Profile Cup	756	0.58	0.19	[-0.36 , 0.74]	Non-inferiority not shown	0.497
[MoP] Mem Original ME Muller Low Profile Cup	337	0.56	0.17	[-0.66 , 1.00]	Non-inferiority not shown	0.688
[MoP] Muller Straight Stem Centerpulse Muller	502	0.95	0.56	[-0.32 , 1.43]	Non-inferiority not shown	0.213
[MoP] Muller Straight Stem Original ME Muller Low Profile Cup	1,412	0.81	0.41	[-0.09 , 0.92]	Non-inferiority not shown	0.106
[MoP] Muller-Biomet Apollo	1,949	1.24	0.85	[0.30 , 1.41]	Inferior by ≥20%	0.003
[MoP] Muller-Biomet Original ME Muller Low Profile Cup	831	1.56	1.17	[0.31 , 2.03]	Inferior by ≥20%	0.008
[MoP] Omnifit Cemented Stem ODC	880	1.40	1.01	[0.20 , 1.82]	Inferior by ≥20%	0.015
[MoP] Omnifit Cemented Stem Trident	270	0.36	-0.03	[-0.80 , 0.74]	Non-inferiority not shown	0.947
[MoP] Omnifit Cementless Stem Secure Fit Cementless Cup	283	0.69	0.30	[-0.70 , 1.30]	Non-inferiority not shown	0.554
[MoP] Omnifit Cementless Stem Trident	426	3.34	2.95	[1.31 , 4.58]	Inferior by ≥100%	<0.001
[MoP] P10 Muller Original ME Muller Low Profile Cup	415	0.96	0.57	[-0.33 , 1.47]	Non-inferiority not shown	0.213
[MoP] Polarstem Cementless R3 Cementless	1,068	0.96	0.57	[0.14 , 1.00]	Inferior by ≥20%	0.010
[MoP] SL-Plus Cementless Stem EP-Fit Plus	2,023	2.60	2.21	[1.52 , 2.90]	Inferior by ≥100%	<0.001
[MoP] SP II Cemented Stem Interplanta	625	2.72	2.33	[1.09 , 3.57]	Inferior by ≥100%	<0.001
[MoP] SP II Cemented Stem Link Flange Cup	274	0.69	0.29	[-0.70 , 1.29]	Non-inferiority not shown	0.560
[MoP] Spectron Reflection Cementless	298	1.24	0.84	[-0.39 , 2.08]	Non-inferiority not shown	0.182
[MoP] Stanmore Modular Stem Elite Plus Cemented Cup	534	0.35	-0.05	[-0.61 , 0.51]	Non-inferiority not shown	0.872
[MoP] Stanmore Modular Stem SHP Cup	931	0.82	0.43	[-0.18 , 1.03]	Non-inferiority not shown	0.169
[MoP] Stanmore Modular Stem Stanmore-Arcom Cup	3,798	1.09	0.70	[0.27 , 1.12]	Inferior by ≥20%	0.001
[MoP] Synergy Cementless Stem R3 Cementless	1,057	1.25	0.86	[0.31 , 1.41]	Inferior by ≥20%	0.002
[MoP] Synergy Cementless Stem Reflection Cementless	1,449	0.87	0.47	[-0.08 , 1.03]	Non-inferiority not shown	0.092
[MoP] Taperfit Cemented Stem Atlas IIIp	444	0.84	0.45	[-0.34 , 1.24]	Non-inferiority not shown	0.268
[MoP] Taperloc Cemented Stem Exceed ABT	280	0.97	0.58	[-0.21 , 1.38]	Non-inferiority not shown	0.152
[MoP] Taperloc Cementless Stem Exceed	553	1.20	0.81	[-0.12 , 1.73]	Non-inferiority not shown	0.089
[MoP] Taperloc Cementless Stem Exceed ABT	3,732	1.75	1.36	[0.91 , 1.80]	Inferior by ≥100%	<0.001
[MoP] Trilock BPS Pinnacle	264	1.65	1.26	[0.01 , 2.51]	Non-inferiority not shown	0.048
[MoP] Trilock BPS Pinnacle Gription	275	1.37	0.98	[-0.19 , 2.15]	Non-inferiority not shown	0.100
[MoP] VerSys Cemented Stem Trilogy	263	1.11	0.71	[-0.56 , 1.99]	Non-inferiority not shown	0.273
[MoP] Versys Cementless Stem Trilogy	860	4.01	3.62	[2.32 , 4.91]	Inferior by ≥100%	<0.001

For peer review only

Supplementary table 1b: Difference in Kaplan-Meier failure estimate between a contemporary reference and implants with at least 250 at risk at 5 years since primary

Stem/cup brand	Number at risk	Cumulative failure (%)	Difference in failure (%)	95% CI	Equivalence status	p-value
[CoP] MS-30 Original ME Muller Low Profile Cup	1,125	0.55	[REFERENCE]			
[CoC] ABG II Monolithic Cementless Stem ABG II Cementless Cup	1,020	2.29	1.73	[0.77 , 2.70]	Inferior by ≥100%	<0.001
[CoC] ABG II Monolithic Cementless Stem Trident	834	3.07	2.51	[1.38 , 3.64]	Inferior by ≥100%	<0.001
[CoC] Accolade Trident	5,169	2.83	2.27	[1.74 , 2.81]	Inferior by ≥100%	<0.001
[CoC] Bicontact Cementless Stem Plasmacup SC	289	1.01	0.46	[-0.60 , 1.51]	Non-inferiority not shown	0.395
[CoC] Bimetric Cementless Stem Exceed ABT	627	1.80	1.24	[0.31 , 2.17]	Inferior by ≥20%	0.009
[CoC] C-Stem AMT Cemented Stem Pinnacle	427	1.30	0.74	[-0.10 , 1.59]	Non-inferiority not shown	0.085
[CoC] C-Stem Cemented Stem Pinnacle	310	1.44	0.88	[-0.42 , 2.19]	Non-inferiority not shown	0.185
[CoC] CPT Continuum	379	2.40	1.85	[0.77 , 2.92]	Inferior by ≥100%	0.001
[CoC] CPT Trilogy AB	468	1.70	1.15	[-0.02 , 2.31]	Non-inferiority not shown	0.053
[CoC] Corail DeltaMotion	633	1.64	1.08	[0.28 , 1.89]	Inferior by ≥20%	0.008
[CoC] Corail Duraloc Option	1,014	2.99	2.44	[1.35 , 3.52]	Inferior by ≥100%	<0.001
[CoC] Corail Pinnacle	17,510	2.40	1.85	[1.44 , 2.26]	Inferior by ≥100%	<0.001
[CoC] Excia Cementless Plasmacup SC	501	1.33	0.77	[0.00 , 1.54]	Non-inferiority not shown	0.049
[CoC] Exeter V40 ABG II Cementless Cup	1,138	1.20	0.64	[-0.04 , 1.32]	Non-inferiority not shown	0.063
[CoC] Exeter V40 Trident	8,141	1.50	0.94	[0.51 , 1.38]	Inferior by ≥20%	<0.001
[CoC] Furlong HAC Stem CSF	1,502	2.59	2.03	[1.18 , 2.89]	Inferior by ≥100%	<0.001
[CoC] Furlong HAC Stem Furlong HAC CSF Plus	5,470	1.84	1.28	[0.84 , 1.73]	Inferior by ≥100%	<0.001
[CoC] Furlong HAC Stem Furlong Threaded	318	3.01	2.46	[0.58 , 4.34]	Inferior by ≥100%	0.010
[CoC] M/L Taper Cementless Continuum	607	1.71	1.15	[0.46 , 1.85]	Inferior by ≥20%	0.001
[CoC] Metafix Stem Trinity	280	1.52	0.96	[0.14 , 1.79]	Inferior by ≥20%	0.022
[CoC] Omnifit Cementless Stem Trident	387	3.10	2.54	[0.95 , 4.13]	Inferior by ≥100%	0.002
[CoC] Polarstem Cementless R3 Cementless	300	0.78	0.22	[-0.40 , 0.85]	Non-inferiority not shown	0.487
[CoC] S-Rom Cementless Stem Pinnacle	407	4.35	3.79	[2.12 , 5.46]	Inferior by ≥100%	<0.001
[CoC] SL-Plus Cementless Stem EP-Fit Plus	1,115	4.96	4.40	[3.14 , 5.66]	Inferior by ≥100%	<0.001
[CoC] SPS Modular April - Ceramic	321	2.99	2.43	[0.95 , 3.92]	Inferior by ≥100%	0.001
[CoC] Summit Cementless Stem Pinnacle	385	1.33	0.78	[-0.18 , 1.73]	Non-inferiority not shown	0.110
[CoC] Taperloc Cementless Stem Exceed ABT	3,900	1.83	1.27	[0.80 , 1.74]	Inferior by ≥100%	<0.001
[CoP] Accolade Trident	1,372	1.95	1.39	[0.82 , 1.97]	Inferior by ≥100%	<0.001
[CoP] C-Stem Cemented Stem Elite Plus Ogee	474	0.75	0.20	[-0.56 , 0.96]	Non-inferiority not shown	0.609
[CoP] C-Stem Cemented Stem Marathon	542	1.34	0.79	[0.10 , 1.47]	Non-inferiority not shown	0.024
[CoP] C-Stem Cemented Stem Opera	656	1.33	0.77	[-0.12 , 1.67]	Non-inferiority not shown	0.091
[CoP] C-Stem Cemented Stem Wroblewski Golf Ball	738	1.05	0.49	[-0.25 , 1.24]	Non-inferiority not shown	0.196
[CoP] CPT ZCA	261	0.39	-0.17	[-0.82 , 0.48]	Non-inferiority not shown	0.611
[CoP] Corail Charnley and Elite Plus LPW	532	1.89	1.33	[0.25 , 2.42]	Inferior by ≥20%	0.016
[CoP] Corail Duraloc Cementless Cup	289	4.72	4.17	[1.81 , 6.53]	Inferior by ≥100%	0.001
[CoP] Corail Elite Plus Cemented Cup	328	1.50	0.95	[-0.24 , 2.13]	Non-inferiority not shown	0.117
[CoP] Corail Elite Plus Ogee	340	2.64	2.08	[0.61 , 3.55]	Inferior by ≥100%	0.006
[CoP] Corail Marathon	573	1.40	0.84	[0.19 , 1.49]	Inferior by ≥20%	0.012
[CoP] Corail Pinnacle	4,134	1.74	1.18	[0.74 , 1.63]	Inferior by ≥100%	<0.001

For peer review only

[CoP] Corail Trilogy	592	1.61	1.06	[0.04 , 2.07]	Non-inferiority not shown	0.041
[CoP] Exeter V40 Charnley and Elite Plus LPW	433	2.02	1.47	[0.38 , 2.55]	Inferior by ≥20%	0.008
[CoP] Exeter V40 Elite Plus Ogee	858	1.08	0.53	[-0.14 , 1.20]	Non-inferiority not shown	0.122
[CoP] Exeter V40 Exeter Contemporary Flanged	2,011	1.31	0.76	[0.23 , 1.28]	Inferior by ≥20%	0.005
[CoP] Exeter V40 Exeter Contemporary Hooded	593	2.58	2.03	[0.93 , 3.12]	Inferior by ≥100%	<0.001
[CoP] Exeter V40 Exeter Duration	664	0.95	0.40	[-0.36 , 1.16]	Non-inferiority not shown	0.304
[CoP] Exeter V40 Exeter X3 Rimfit	363	1.16	0.60	[0.09 , 1.12]	Non-inferiority not shown	0.022
[CoP] Exeter V40 Marathon	258	0.94	0.38	[-0.44 , 1.21]	Non-inferiority not shown	0.365
[CoP] Exeter V40 Pinnacle	297	1.53	0.98	[0.08 , 1.87]	Non-inferiority not shown	0.032
[CoP] Exeter V40 Trident	2,337	1.16	0.60	[0.15 , 1.06]	Inferior by ≥20%	0.009
[CoP] Exeter V40 Trilogy	1,627	1.31	0.76	[0.15 , 1.37]	Inferior by ≥20%	0.015
[CoP] Furlong HAC Stem CSF	5,425	1.65	1.09	[0.61 , 1.57]	Inferior by ≥100%	<0.001
[CoP] Furlong HAC Stem Furlong HAC CSF Plus	908	2.02	1.47	[0.74 , 2.19]	Inferior by ≥100%	<0.001
[CoP] Furlong HAC Stem Furlong Threaded	362	1.29	0.73	[-0.45 , 1.91]	Non-inferiority not shown	0.225
[CoP] Muller Straight Stem Original ME Muller Low Profile Cup	298	1.22	0.67	[-0.38 , 1.71]	Non-inferiority not shown	0.210
[CoP] SL-Plus Cementless Stem Bicon-Plus	558	4.05	3.50	[1.93 , 5.07]	Inferior by ≥100%	<0.001
[CoP] SL-Plus Cementless Stem EP-Fit Plus	810	3.41	2.86	[1.65 , 4.06]	Inferior by ≥100%	<0.001
[CoP] Stanmore Modular Stem Stanmore-Arcom Cup	257	1.19	0.64	[-0.46 , 1.74]	Non-inferiority not shown	0.257
[CoP] Taperloc Cementless Stem Exceed ABT	775	1.43	0.87	[0.26 , 1.49]	Inferior by ≥20%	0.006
[CoP] Versys Cementless Stem Trilogy	342	1.07	0.51	[-0.59 , 1.61]	Non-inferiority not shown	0.363
[MoP] ABG II Monolithic Cementless Stem ABG II Cementless Cup	419	1.92	1.37	[0.07 , 2.66]	Non-inferiority not shown	0.039
[MoP] ABG II Monolithic Cementless Stem Trident	402	4.11	3.56	[1.71 , 5.41]	Inferior by ≥100%	<0.001
[MoP] Accolade Trident	6,100	2.76	2.21	[1.72 , 2.70]	Inferior by ≥100%	<0.001
[MoP] Anthology R3 Cementless	353	2.14	1.59	[0.69 , 2.48]	Inferior by ≥100%	<0.001
[MoP] Bimetric Cementless Stem Mallory-Head Cementless Cup	271	2.05	1.50	[0.23 , 2.76]	Inferior by ≥20%	0.020
[MoP] C-Stem AMT Cemented Stem Charnley and Elite Plus LPW	1,397	1.46	0.90	[0.30 , 1.51]	Inferior by ≥20%	0.003
[MoP] C-Stem AMT Cemented Stem Elite Plus Cemented Cup	435	1.22	0.66	[-0.26 , 1.58]	Non-inferiority not shown	0.157
[MoP] C-Stem AMT Cemented Stem Elite Plus Ogee	918	1.01	0.46	[-0.13 , 1.05]	Non-inferiority not shown	0.130
[MoP] C-Stem AMT Cemented Stem Marathon	356	1.46	0.90	[0.10 , 1.71]	Non-inferiority not shown	0.028
[MoP] C-Stem AMT Cemented Stem Pinnacle	657	1.85	1.29	[0.56 , 2.02]	Inferior by ≥100%	0.001
[MoP] C-Stem Cemented Stem Charnley Ogee	680	1.86	1.30	[0.34 , 2.26]	Inferior by ≥20%	0.008
[MoP] C-Stem Cemented Stem Charnley and Elite Plus LPW	1,435	1.57	1.02	[0.31 , 1.72]	Inferior by ≥20%	0.005
[MoP] C-Stem Cemented Stem Duraloc Cementless Cup	496	2.67	2.12	[0.73 , 3.50]	Inferior by ≥100%	0.003
[MoP] C-Stem Cemented Stem Elite Plus Cemented Cup	586	0.90	0.34	[-0.46 , 1.15]	Non-inferiority not shown	0.405
[MoP] C-Stem Cemented Stem Elite Plus Ogee	2,459	1.13	0.57	[0.06 , 1.08]	Non-inferiority not shown	0.028
[MoP] C-Stem Cemented Stem Marathon	744	1.25	0.70	[0.08 , 1.32]	Non-inferiority not shown	0.028
[MoP] C-Stem Cemented Stem Opera	1,013	1.44	0.89	[0.16 , 1.62]	Inferior by ≥20%	0.017
[MoP] C-Stem Cemented Stem Pinnacle	331	1.56	1.01	[-0.13 , 2.14]	Non-inferiority not shown	0.083
[MoP] C-Stem Cemented Stem Polarcup Cementless	255	0.71	0.16	[-0.89 , 1.21]	Non-inferiority not shown	0.768
[MoP] C-Stem Cemented Stem Trilogy	338	0.82	0.26	[-0.73 , 1.25]	Non-inferiority not shown	0.606
[MoP] C-Stem Cemented Stem Wroblewski Golf Ball	756	1.50	0.94	[0.10 , 1.79]	Non-inferiority not shown	0.028
[MoP] CCA Cemented Stem CCB Cup	738	0.79	0.23	[-0.38 , 0.85]	Non-inferiority not shown	0.459
[MoP] CLS Cementless Stem Allofit	553	2.90	2.34	[1.01 , 3.68]	Inferior by ≥100%	0.001
[MoP] CLS Cementless Stem Trilogy	419	3.18	2.63	[1.17 , 4.09]	Inferior by ≥100%	<0.001
[MoP] CMK Modular Cemented Stem CMK Cemented Cup	418	1.08	0.52	[-0.49 , 1.53]	Non-inferiority not shown	0.310
[MoP] CPCS Opera	866	1.28	0.72	[-0.02 , 1.46]	Non-inferiority not shown	0.056

For peer review only

[MoP] CPCS Polarcup Cementless	368	0.99	0.43	[-0.60 , 1.46]	Non-inferiority not shown	0.410
[MoP] CPS Plus Cenator Cemented Cup	339	0.80	0.25	[-0.73 , 1.23]	Non-inferiority not shown	0.618
[MoP] CPS Plus EP-Fit Plus	282	3.23	2.68	[0.67 , 4.69]	Inferior by ≥100%	0.009
[MoP] CPS Plus Opera	464	2.16	1.61	[0.29 , 2.92]	Inferior by ≥20%	0.017
[MoP] CPT Allofit	430	0.94	0.39	[-0.41 , 1.18]	Non-inferiority not shown	0.339
[MoP] CPT Elite Plus Ogee	1,744	1.87	1.32	[0.67 , 1.97]	Inferior by ≥100%	<0.001
[MoP] CPT Exceed	263	4.10	3.55	[1.24 , 5.85]	Inferior by ≥100%	0.003
[MoP] CPT Opera	378	2.17	1.62	[0.16 , 3.07]	Inferior by ≥20%	0.029
[MoP] CPT Original ME Muller Low Profile Cup	422	2.20	1.64	[0.54 , 2.75]	Inferior by ≥20%	0.004
[MoP] CPT Pinnacle	639	2.42	1.86	[0.72 , 3.00]	Inferior by ≥100%	0.001
[MoP] CPT Trabecular Metal Modular Cementless Cup	523	2.41	1.85	[0.82 , 2.89]	Inferior by ≥100%	<0.001
[MoP] CPT Trilogy	5,765	2.20	1.65	[1.17 , 2.12]	Inferior by ≥100%	<0.001
[MoP] CPT ZCA	5,547	2.11	1.55	[1.07 , 2.03]	Inferior by ≥100%	<0.001
[MoP] Centrament Chirulen	310	1.12	0.57	[-0.59 , 1.72]	Non-inferiority not shown	0.337
[MoP] Charnley Cemented Stem Charnley Cemented Cup	3,601	1.72	1.17	[0.63 , 1.71]	Inferior by ≥100%	<0.001
[MoP] Charnley Cemented Stem Charnley Ogee	7,533	1.88	1.33	[0.86 , 1.79]	Inferior by ≥100%	<0.001
[MoP] Charnley Cemented Stem Charnley and Elite Plus LPW	5,041	1.12	0.56	[0.11 , 1.02]	Non-inferiority not shown	0.015
[MoP] Charnley Cemented Stem Opera	1,039	1.46	0.91	[0.14 , 1.67]	Inferior by ≥20%	0.020
[MoP] Charnley Cemented Stem Wroblewski Golf Ball	797	1.74	1.18	[0.30 , 2.06]	Inferior by ≥20%	0.008
[MoP] Charnley Modular Charnley and Elite Plus LPW	277	0.27	-0.28	[-0.93 , 0.37]	Non-inferiority not shown	0.393
[MoP] Corail Charnley and Elite Plus LPW	448	1.85	1.30	[0.30 , 2.29]	Inferior by ≥20%	0.010
[MoP] Corail Duraloc Cementless Cup	3,226	2.27	1.71	[1.10 , 2.33]	Inferior by ≥100%	<0.001
[MoP] Corail Elite Plus Cemented Cup	727	1.31	0.75	[-0.05 , 1.56]	Non-inferiority not shown	0.067
[MoP] Corail Elite Plus Ogee	1,029	1.70	1.14	[0.41 , 1.88]	Inferior by ≥20%	0.002
[MoP] Corail Exeter Contemporary Flanged	328	1.35	0.79	[-0.14 , 1.72]	Non-inferiority not shown	0.095
[MoP] Corail Marathon	1,385	1.23	0.67	[0.17 , 1.18]	Inferior by ≥20%	0.009
[MoP] Corail Pinnacle	16,408	1.70	1.15	[0.76 , 1.54]	Inferior by ≥100%	<0.001
[MoP] Corail Trilogy	1,383	1.72	1.17	[0.47 , 1.87]	Inferior by ≥20%	0.001
[MoP] Elite Plus Cemented Stem Charnley and Elite Plus LPW	308	1.66	1.11	[-0.26 , 2.47]	Non-inferiority not shown	0.113
[MoP] Elite Plus Cemented Stem Elite Plus Ogee	519	1.06	0.50	[-0.42 , 1.42]	Non-inferiority not shown	0.284
[MoP] Exeter Elite Plus Ogee	259	0.71	0.15	[-0.89 , 1.20]	Non-inferiority not shown	0.775
[MoP] Exeter V40 ABG II Cementless Cup	693	1.56	1.00	[0.05 , 1.95]	Non-inferiority not shown	0.038
[MoP] Exeter V40 Cenator Cemented Cup	1,725	1.99	1.44	[0.75 , 2.12]	Inferior by ≥100%	<0.001
[MoP] Exeter V40 Charnley Cemented Cup	286	0.87	0.32	[-0.73 , 1.37]	Non-inferiority not shown	0.553
[MoP] Exeter V40 Charnley Ogee	1,113	1.44	0.88	[0.15 , 1.61]	Inferior by ≥20%	0.018
[MoP] Exeter V40 Charnley and Elite Plus LPW	1,812	1.47	0.91	[0.34 , 1.48]	Inferior by ≥20%	0.002
[MoP] Exeter V40 Duraloc Cementless Cup	1,000	2.04	1.48	[0.56 , 2.40]	Inferior by ≥100%	0.002
[MoP] Exeter V40 EP-Fit Plus	496	2.56	2.01	[0.63 , 3.38]	Inferior by ≥100%	0.004
[MoP] Exeter V40 Elite Plus Cemented Cup	2,674	0.76	0.21	[-0.25 , 0.66]	Non-inferiority not shown	0.376
[MoP] Exeter V40 Elite Plus Ogee	12,458	1.11	0.56	[0.16 , 0.96]	Inferior by ≥20%	0.006
[MoP] Exeter V40 Exceed	375	0.94	0.39	[-0.51 , 1.29]	Non-inferiority not shown	0.399
[MoP] Exeter V40 Exceed ABT	305	1.17	0.62	[-0.43 , 1.67]	Non-inferiority not shown	0.251
[MoP] Exeter V40 Exeter Contemporary Flanged	26,691	1.21	0.65	[0.27 , 1.03]	Inferior by ≥20%	0.001
[MoP] Exeter V40 Exeter Contemporary Hooded	11,227	2.10	1.54	[1.12 , 1.96]	Inferior by ≥100%	<0.001
[MoP] Exeter V40 Exeter Duration	10,271	1.70	1.15	[0.72 , 1.57]	Inferior by ≥100%	<0.001
[MoP] Exeter V40 Exeter X3 Rimfit	859	1.16	0.61	[0.14 , 1.07]	Inferior by ≥20%	0.010

For peer review only

[MoP] Exeter V40 Marathon	555	1.54	0.99	[0.24 , 1.73]	Inferior by ≥20%	0.010
[MoP] Exeter V40 Opera	1,734	1.18	0.63	[0.06 , 1.19]	Non-inferiority not shown	0.030
[MoP] Exeter V40 Pinnacle	1,546	1.60	1.05	[0.48 , 1.62]	Inferior by ≥20%	<0.001
[MoP] Exeter V40 Reflection Cementless	1,812	1.58	1.02	[0.37 , 1.67]	Inferior by ≥20%	0.002
[MoP] Exeter V40 Trabecular Metal Modular Cementless Cup	276	2.23	1.68	[0.19 , 3.17]	Inferior by ≥20%	0.028
[MoP] Exeter V40 Trabecular Metal Natural Cup	284	4.06	3.51	[1.39 , 5.63]	Inferior by ≥100%	0.001
[MoP] Exeter V40 Trident	10,507	1.44	0.88	[0.48 , 1.29]	Inferior by ≥20%	<0.001
[MoP] Exeter V40 Trilogy	6,611	1.33	0.77	[0.34 , 1.21]	Inferior by ≥20%	0.001
[MoP] Exeter V40 Ultima Cemented Cup	1,040	2.19	1.64	[0.73 , 2.55]	Inferior by ≥100%	<0.001
[MoP] Furlong Cemented Stem JRI Cemented Cup	1,287	1.66	1.11	[0.37 , 1.84]	Inferior by ≥20%	0.003
[MoP] Furlong HAC Stem CSF	5,416	2.43	1.87	[1.36 , 2.38]	Inferior by ≥100%	<0.001
[MoP] Furlong HAC Stem Furlong HAC CSF Plus	1,808	2.91	2.35	[1.70 , 3.01]	Inferior by ≥100%	<0.001
[MoP] Furlong HAC Stem Furlong Threaded	416	3.01	2.46	[0.91 , 4.00]	Inferior by ≥100%	0.002
[MoP] Furlong HAC Stem Trilogy	264	1.90	1.35	[-0.10 , 2.80]	Non-inferiority not shown	0.067
[MoP] M/L Taper Cementless Allofit	295	2.04	1.48	[0.22 , 2.74]	Inferior by ≥20%	0.021
[MoP] M/L Taper Cementless Trilogy	328	2.54	1.98	[0.73 , 3.23]	Inferior by ≥100%	0.002
[MoP] MS-30 Allofit	269	0.57	0.01	[-0.85 , 0.87]	Non-inferiority not shown	0.981
[MoP] MS-30 Original ME Muller Low Profile Cup	531	1.23	0.67	[-0.19 , 1.54]	Non-inferiority not shown	0.128
[MoP] Mem Original ME Muller Low Profile Cup	313	0.86	0.30	[-0.73 , 1.34]	Non-inferiority not shown	0.567
[MoP] Muller Straight Stem Centerpulse Muller	442	1.79	1.24	[0.02 , 2.46]	Non-inferiority not shown	0.047
[MoP] Muller Straight Stem Original ME Muller Low Profile Cup	1,008	1.12	0.56	[-0.07 , 1.19]	Non-inferiority not shown	0.080
[MoP] Muller-Biomet Apollo	1,539	1.41	0.86	[0.23 , 1.49]	Inferior by ≥20%	0.008
[MoP] Muller-Biomet Original ME Muller Low Profile Cup	512	1.96	1.41	[0.41 , 2.40]	Inferior by ≥20%	0.006
[MoP] Omnifit Cemented Stem ODC	801	1.98	1.43	[0.45 , 2.41]	Inferior by ≥20%	0.004
[MoP] Omnifit Cemented Stem Trident	250	1.89	1.34	[-0.35 , 3.02]	Non-inferiority not shown	0.120
[MoP] Omnifit Cementless Stem Secure Fit Cementless Cup	275	0.69	0.14	[-0.89 , 1.16]	Non-inferiority not shown	0.791
[MoP] Omnifit Cementless Stem Trident	385	5.46	4.90	[2.78 , 7.02]	Inferior by ≥100%	<0.001
[MoP] P10 Muller Original ME Muller Low Profile Cup	293	0.96	0.41	[-0.52 , 1.34]	Non-inferiority not shown	0.388
[MoP] SL-Plus Cementless Stem EP-Fit Plus	1,651	3.51	2.96	[2.12 , 3.80]	Inferior by ≥100%	<0.001
[MoP] SP II Cemented Stem Interplanta	552	3.23	2.67	[1.29 , 4.05]	Inferior by ≥100%	<0.001
[MoP] Spectron Reflection Cementless	267	1.24	0.68	[-0.58 , 1.94]	Non-inferiority not shown	0.289
[MoP] Stanmore Modular Stem Elite Plus Cemented Cup	406	0.98	0.42	[-0.51 , 1.36]	Non-inferiority not shown	0.375
[MoP] Stanmore Modular Stem SHP Cup	677	0.95	0.40	[-0.30 , 1.10]	Non-inferiority not shown	0.265
[MoP] Stanmore Modular Stem Stanmore-Arcom Cup	2,920	1.62	1.06	[0.52 , 1.60]	Inferior by ≥20%	<0.001
[MoP] Synergy Cementless Stem Reflection Cementless	1,370	1.43	0.87	[0.16 , 1.59]	Inferior by ≥20%	0.016
[MoP] Taperfit Cemented Stem Atlas IIIp	250	2.18	1.63	[0.20 , 3.06]	Inferior by ≥20%	0.026
[MoP] Taperloc Cementless Stem Exceed	470	1.57	1.01	[-0.07 , 2.10]	Non-inferiority not shown	0.066
[MoP] Taperloc Cementless Stem Exceed ABT	1,793	2.07	1.52	[0.98 , 2.06]	Inferior by ≥100%	<0.001
[MoP] Versys Cementless Stem Trilogy	797	4.81	4.26	[2.82 , 5.69]	Inferior by ≥100%	<0.001

For peer review only

Supplementary table 1c: Difference in Kaplan-Meier failure estimate between a contemporary reference and implants with at least 250 at risk at 7 years since primary

Stem/cup brand	Number at risk	Cumulative failure (%)	Difference in failure (%)	95% CI	Equivalence status	p-value
[MoP] Exeter V40 Elite Plus Cemented Cup	1,773	0.91	[REFERENCE]			
[CoC] ABG II Monolithic Cementless Stem ABG II Cementless Cup	909	3.09	2.19	[1.10 , 3.27]	Inferior by ≥100%	<0.001
[CoC] ABG II Monolithic Cementless Stem Trident	705	3.68	2.77	[1.55 , 4.00]	Inferior by ≥100%	<0.001
[CoC] Accolade Trident	2,978	3.31	2.40	[1.86 , 2.95]	Inferior by ≥100%	<0.001
[CoC] C-Stem Cemented Stem Pinnacle	286	1.44	0.53	[-0.76 , 1.82]	Non-inferiority not shown	0.420
[CoC] CPT Trilogy AB	265	1.93	1.03	[-0.20 , 2.26]	Non-inferiority not shown	0.101
[CoC] Corail Duraloc Option	819	3.61	2.71	[1.54 , 3.88]	Inferior by ≥100%	<0.001
[CoC] Corail Pinnacle	7,589	2.93	2.03	[1.65 , 2.40]	Inferior by ≥100%	<0.001
[CoC] Exeter V40 ABG II Cementless Cup	918	1.69	0.79	[0.01 , 1.57]	Non-inferiority not shown	0.048
[CoC] Exeter V40 Trident	5,732	1.99	1.09	[0.67 , 1.51]	Inferior by ≥20%	<0.001
[CoC] Furlong HAC Stem CSF	1,405	3.19	2.29	[1.37 , 3.21]	Inferior by ≥100%	<0.001
[CoC] Furlong HAC Stem Furlong HAC CSF Plus	2,083	2.15	1.25	[0.81 , 1.69]	Inferior by ≥20%	<0.001
[CoC] Furlong HAC Stem Furlong Threaded	308	4.24	3.34	[1.14 , 5.53]	Inferior by ≥100%	0.003
[CoC] Omnifit Cementless Stem Trident	313	4.21	3.31	[1.41 , 5.21]	Inferior by ≥100%	0.001
[CoC] SL-Plus Cementless Stem EP-Fit Plus	968	5.32	4.41	[3.12 , 5.70]	Inferior by ≥100%	<0.001
[CoC] Summit Cementless Stem Pinnacle	301	1.60	0.69	[-0.37 , 1.76]	Non-inferiority not shown	0.203
[CoC] Taperloc Cementless Stem Exceed ABT	1,609	2.02	1.12	[0.65 , 1.58]	Inferior by ≥20%	<0.001
[CoP] Accolade Trident	544	2.20	1.29	[0.64 , 1.95]	Inferior by ≥20%	<0.001
[CoP] C-Stem Cemented Stem Elite Plus Ogee	342	0.75	-0.15	[-0.89 , 0.58]	Non-inferiority not shown	0.684
[CoP] C-Stem Cemented Stem Opera	478	1.69	0.79	[-0.22 , 1.80]	Non-inferiority not shown	0.127
[CoP] C-Stem Cemented Stem Wroblewski Golf Ball	598	1.67	0.76	[-0.18 , 1.70]	Non-inferiority not shown	0.112
[CoP] Corail Charnley and Elite Plus LPW	335	2.49	1.59	[0.32 , 2.85]	Inferior by ≥20%	0.014
[CoP] Corail Duraloc Cementless Cup	266	5.06	4.15	[1.72 , 6.59]	Inferior by ≥100%	0.001
[CoP] Corail Pinnacle	1,638	2.16	1.25	[0.78 , 1.73]	Inferior by ≥20%	<0.001
[CoP] Corail Trilogy	477	1.61	0.71	[-0.29 , 1.71]	Non-inferiority not shown	0.164
[CoP] Exeter V40 Charnley and Elite Plus LPW	250	2.02	1.12	[0.05 , 2.19]	Non-inferiority not shown	0.040
[CoP] Exeter V40 Elite Plus Ogee	535	1.35	0.44	[-0.29 , 1.18]	Non-inferiority not shown	0.240
[CoP] Exeter V40 Exeter Contemporary Flanged	1,110	1.42	0.52	[0.00 , 1.03]	Non-inferiority not shown	0.048
[CoP] Exeter V40 Exeter Contemporary Hooded	410	3.14	2.23	[0.98 , 3.48]	Inferior by ≥100%	<0.001
[CoP] Exeter V40 Exeter Duration	500	2.11	1.21	[0.08 , 2.33]	Non-inferiority not shown	0.035
[CoP] Exeter V40 Trident	1,113	1.46	0.56	[0.07 , 1.05]	Non-inferiority not shown	0.026
[CoP] Exeter V40 Trilogy	1,159	1.58	0.67	[0.04 , 1.30]	Non-inferiority not shown	0.037
[CoP] Furlong HAC Stem CSF	4,459	2.07	1.16	[0.69 , 1.64]	Inferior by ≥20%	<0.001
[CoP] Furlong HAC Stem Furlong HAC CSF Plus	345	2.46	1.55	[0.69 , 2.41]	Inferior by ≥20%	<0.001
[CoP] Furlong HAC Stem Furlong Threaded	327	1.84	0.94	[-0.45 , 2.33]	Non-inferiority not shown	0.187
[CoP] MS-30 Original ME Muller Low Profile Cup	675	0.70	-0.21	[-0.77 , 0.35]	Non-inferiority not shown	0.460
[CoP] SL-Plus Cementless Stem Bicon-Plus	406	4.66	3.75	[2.06 , 5.45]	Inferior by ≥100%	<0.001
[CoP] SL-Plus Cementless Stem EP-Fit Plus	579	4.10	3.20	[1.87 , 4.53]	Inferior by ≥100%	<0.001
[CoP] Versys Cementless Stem Trilogy	303	1.36	0.46	[-0.77 , 1.69]	Non-inferiority not shown	0.465
[MoP] ABG II Monolithic Cementless Stem ABG II Cementless Cup	376	2.40	1.50	[0.06 , 2.94]	Non-inferiority not shown	0.041

For peer review only

[MoP] ABG II Monolithic Cementless Stem Trident	301	5.72	4.81	[2.60 , 7.02]	Inferior by ≥100%	<0.001
[MoP] Accolade Trident	2,764	3.50	2.59	[2.07 , 3.11]	Inferior by ≥100%	<0.001
[MoP] C-Stem AMT Cemented Stem Charnley and Elite Plus LPW	782	1.81	0.91	[0.21 , 1.61]	Inferior by ≥20%	0.011
[MoP] C-Stem AMT Cemented Stem Elite Plus Ogee	523	1.71	0.81	[-0.02 , 1.64]	Non-inferiority not shown	0.057
[MoP] C-Stem AMT Cemented Stem Pinnacle	298	2.08	1.18	[0.34 , 2.02]	Inferior by ≥20%	0.006
[MoP] C-Stem Cemented Stem Charnley Ogee	539	2.03	1.12	[0.12 , 2.12]	Non-inferiority not shown	0.028
[MoP] C-Stem Cemented Stem Charnley and Elite Plus LPW	1,298	1.94	1.04	[0.29 , 1.79]	Inferior by ≥20%	0.007
[MoP] C-Stem Cemented Stem Duraloc Cementless Cup	391	3.73	2.83	[1.18 , 4.47]	Inferior by ≥100%	0.001
[MoP] C-Stem Cemented Stem Elite Plus Cemented Cup	472	1.62	0.71	[-0.34 , 1.76]	Non-inferiority not shown	0.184
[MoP] C-Stem Cemented Stem Elite Plus Ogee	1,851	1.55	0.65	[0.10 , 1.19]	Non-inferiority not shown	0.020
[MoP] C-Stem Cemented Stem Opera	744	2.01	1.10	[0.24 , 1.96]	Inferior by ≥20%	0.012
[MoP] C-Stem Cemented Stem Pinnacle	258	1.94	1.04	[-0.30 , 2.38]	Non-inferiority not shown	0.130
[MoP] C-Stem Cemented Stem Trilogy	278	1.11	0.21	[-0.92 , 1.34]	Non-inferiority not shown	0.719
[MoP] C-Stem Cemented Stem Wroblewski Golf Ball	502	1.88	0.97	[0.00 , 1.94]	Non-inferiority not shown	0.050
[MoP] CCA Cemented Stem CCB Cup	328	1.69	0.78	[-0.20 , 1.77]	Non-inferiority not shown	0.119
[MoP] CLS Cementless Stem Allofit	350	3.77	2.87	[1.30 , 4.44]	Inferior by ≥100%	<0.001
[MoP] CMK Modular Cemented Stem CMK Cemented Cup	329	1.08	0.17	[-0.82 , 1.16]	Non-inferiority not shown	0.734
[MoP] CPCS Opera	324	2.86	1.95	[0.78 , 3.12]	Inferior by ≥20%	0.001
[MoP] CPS Plus Cenator Cemented Cup	250	0.80	-0.10	[-1.06 , 0.86]	Non-inferiority not shown	0.836
[MoP] CPS Plus Opera	421	3.49	2.58	[0.92 , 4.25]	Inferior by ≥100%	0.002
[MoP] CPT Elite Plus Ogee	1,177	2.37	1.47	[0.75 , 2.19]	Inferior by ≥20%	<0.001
[MoP] CPT Opera	276	3.01	2.11	[0.39 , 3.82]	Inferior by ≥20%	0.016
[MoP] CPT Trabecular Metal Modular Cementless Cup	270	3.00	2.09	[0.78 , 3.40]	Inferior by ≥20%	0.002
[MoP] CPT Trilogy	3,606	2.69	1.78	[1.31 , 2.26]	Inferior by ≥100%	<0.001
[MoP] CPT ZCA	3,619	2.69	1.78	[1.30 , 2.27]	Inferior by ≥100%	<0.001
[MoP] Centrament Chirulen	257	1.12	0.22	[-0.93 , 1.36]	Non-inferiority not shown	0.710
[MoP] Charnley Cemented Stem Charnley Cemented Cup	3,004	2.31	1.41	[0.84 , 1.97]	Inferior by ≥20%	<0.001
[MoP] Charnley Cemented Stem Charnley Ogee	5,956	2.49	1.58	[1.13 , 2.04]	Inferior by ≥100%	<0.001
[MoP] Charnley Cemented Stem Charnley and Elite Plus LPW	4,036	1.51	0.60	[0.15 , 1.05]	Non-inferiority not shown	0.009
[MoP] Charnley Cemented Stem Opera	733	2.08	1.18	[0.29 , 2.07]	Inferior by ≥20%	0.009
[MoP] Charnley Cemented Stem Wroblewski Golf Ball	659	1.87	0.96	[0.07 , 1.86]	Non-inferiority not shown	0.035
[MoP] Corail Duraloc Cementless Cup	2,661	3.39	2.48	[1.79 , 3.18]	Inferior by ≥100%	<0.001
[MoP] Corail Elite Plus Cemented Cup	467	1.82	0.91	[-0.06 , 1.88]	Non-inferiority not shown	0.065
[MoP] Corail Elite Plus Ogee	696	1.83	0.92	[0.17 , 1.68]	Non-inferiority not shown	0.016
[MoP] Corail Marathon	354	1.49	0.59	[0.03 , 1.15]	Non-inferiority not shown	0.038
[MoP] Corail Pinnacle	7,798	2.17	1.26	[0.90 , 1.63]	Inferior by ≥20%	<0.001
[MoP] Corail Trilogy	855	2.65	1.75	[0.86 , 2.63]	Inferior by ≥20%	<0.001
[MoP] Elite Plus Cemented Stem Charnley and Elite Plus LPW	281	2.00	1.10	[-0.41 , 2.60]	Non-inferiority not shown	0.154
[MoP] Elite Plus Cemented Stem Elite Plus Ogee	465	1.26	0.35	[-0.63 , 1.33]	Non-inferiority not shown	0.480
[MoP] Exeter V40 ABG II Cementless Cup	626	2.02	1.11	[0.05 , 2.18]	Non-inferiority not shown	0.040
[MoP] Exeter V40 Cenator Cemented Cup	1,274	2.26	1.36	[0.65 , 2.07]	Inferior by ≥20%	<0.001
[MoP] Exeter V40 Charnley Ogee	901	1.62	0.71	[-0.03 , 1.46]	Non-inferiority not shown	0.061
[MoP] Exeter V40 Charnley and Elite Plus LPW	1,082	1.81	0.91	[0.29 , 1.53]	Inferior by ≥20%	0.004
[MoP] Exeter V40 Duraloc Cementless Cup	754	2.24	1.33	[0.39 , 2.27]	Inferior by ≥20%	0.005
[MoP] Exeter V40 EP-Fit Plus	424	3.64	2.74	[1.09 , 4.38]	Inferior by ≥100%	0.001
[MoP] Exeter V40 Elite Plus Ogee	8,638	1.50	0.59	[0.22 , 0.96]	Inferior by ≥20%	0.002

For peer review only

[MoP] Exeter V40 Exceed	265	0.94	0.04	[-0.84 , 0.92]	Non-inferiority not shown	0.934
[MoP] Exeter V40 Exeter Contemporary Flanged	16,415	1.58	0.67	[0.33 , 1.01]	Inferior by ≥20%	<0.001
[MoP] Exeter V40 Exeter Contemporary Hooded	7,073	2.79	1.89	[1.47 , 2.30]	Inferior by ≥100%	<0.001
[MoP] Exeter V40 Exeter Duration	7,252	2.45	1.55	[1.13 , 1.97]	Inferior by ≥100%	<0.001
[MoP] Exeter V40 Opera	1,144	1.59	0.68	[0.06 , 1.31]	Non-inferiority not shown	0.032
[MoP] Exeter V40 Pinnacle	718	1.89	0.98	[0.35 , 1.61]	Inferior by ≥20%	0.002
[MoP] Exeter V40 Reflection Cementless	1,359	2.31	1.41	[0.66 , 2.15]	Inferior by ≥20%	<0.001
[MoP] Exeter V40 Trident	5,685	1.85	0.95	[0.57 , 1.33]	Inferior by ≥20%	<0.001
[MoP] Exeter V40 Trilogy	4,380	1.67	0.77	[0.34 , 1.19]	Inferior by ≥20%	<0.001
[MoP] Exeter V40 Ultima Cemented Cup	916	2.29	1.38	[0.47 , 2.29]	Inferior by ≥20%	0.003
[MoP] Furlong Cemented Stem JRI Cemented Cup	1,041	1.99	1.09	[0.31 , 1.86]	Inferior by ≥20%	0.006
[MoP] Furlong HAC Stem CSF	4,138	3.06	2.15	[1.63 , 2.67]	Inferior by ≥100%	<0.001
[MoP] Furlong HAC Stem Furlong HAC CSF Plus	731	3.34	2.44	[1.72 , 3.16]	Inferior by ≥100%	<0.001
[MoP] Furlong HAC Stem Furlong Threaded	348	3.27	2.37	[0.75 , 3.98]	Inferior by ≥20%	0.004
[MoP] MS-30 Original ME Muller Low Profile Cup	334	1.74	0.83	[-0.27 , 1.93]	Non-inferiority not shown	0.139
[MoP] Mem Original ME Muller Low Profile Cup	286	0.86	-0.05	[-1.06 , 0.97]	Non-inferiority not shown	0.926
[MoP] Muller Straight Stem Centerpulse Muller	316	3.15	2.24	[0.56 , 3.92]	Inferior by ≥20%	0.009
[MoP] Muller Straight Stem Original ME Muller Low Profile Cup	646	2.06	1.16	[0.27 , 2.04]	Inferior by ≥20%	0.010
[MoP] Muller-Biomet Apollo	1,089	1.70	0.80	[0.13 , 1.46]	Non-inferiority not shown	0.019
[MoP] Muller-Biomet Original ME Muller Low Profile Cup	267	2.22	1.32	[0.21 , 2.42]	Inferior by ≥20%	0.019
[MoP] Omnifit Cemented Stem ODC	708	2.78	1.88	[0.73 , 3.03]	Inferior by ≥20%	0.001
[MoP] Omnifit Cementless Stem Secure Fit Cementless Cup	255	1.42	0.52	[-0.90 , 1.93]	Non-inferiority not shown	0.476
[MoP] Omnifit Cementless Stem Trident	321	6.51	5.61	[3.28 , 7.94]	Inferior by ≥100%	<0.001
[MoP] SL-Plus Cementless Stem EP-Fit Plus	1,230	4.42	3.52	[2.58 , 4.46]	Inferior by ≥100%	<0.001
[MoP] SP II Cemented Stem Interplanta	455	4.00	3.09	[1.54 , 4.65]	Inferior by ≥100%	<0.001
[MoP] Stanmore Modular Stem SHP Cup	480	1.46	0.55	[-0.33 , 1.44]	Non-inferiority not shown	0.222
[MoP] Stanmore Modular Stem Stanmore-Arcom Cup	1,908	1.97	1.06	[0.51 , 1.61]	Inferior by ≥20%	<0.001
[MoP] Synergy Cementless Stem Reflection Cementless	1,002	1.75	0.85	[0.10 , 1.60]	Non-inferiority not shown	0.027
[MoP] Taperloc Cementless Stem Exceed	370	2.27	1.37	[0.04 , 2.69]	Non-inferiority not shown	0.043
[MoP] Taperloc Cementless Stem Exceed ABT	513	2.52	1.61	[0.96 , 2.26]	Inferior by ≥100%	<0.001
[MoP] Versys Cementless Stem Trilogy	714	4.81	3.91	[2.48 , 5.33]	Inferior by ≥100%	<0.001

For peer review only

Supplementary table 1d: Difference in Kaplan-Meier failure estimate between a contemporary reference and implants with at least 250 at risk at 10 years since primary

Stem/cup brand	Number at risk	Cumulative failure (%)	Difference in failure (%)	95% CI	Equivalence status	p-value
[MoP] Exeter V40 Elite Plus Ogee	3,580	2.14	[REFERENCE]			
[CoC] ABG II Monolithic Cementless Stem ABG II Cementless Cup	695	4.42	2.28	[0.96 , 3.60]	Inferior by ≥20%	0.001
[CoC] ABG II Monolithic Cementless Stem Trident	412	4.15	2.01	[0.68 , 3.34]	Inferior by ≥20%	0.003
[CoC] Accolade Trident	454	4.38	2.24	[1.48 , 3.00]	Inferior by ≥20%	<0.001
[CoC] Corail Duraloc Option	383	4.45	2.31	[0.93 , 3.68]	Inferior by ≥20%	0.001
[CoC] Corail Pinnacle	886	3.90	1.76	[1.23 , 2.29]	Inferior by ≥20%	<0.001
[CoC] Exeter V40 ABG II Cementless Cup	440	2.66	0.52	[-0.54 , 1.58]	Non-inferiority not shown	0.334
[CoC] Exeter V40 Trident	1,846	2.59	0.44	[-0.04 , 0.93]	Non-inferiority not shown	0.072
[CoC] Furlong HAC Stem CSF	829	4.37	2.22	[1.13 , 3.32]	Inferior by ≥20%	<0.001
[CoC] SL-Plus Cementless Stem EP-Fit Plus	288	6.94	4.80	[3.25 , 6.35]	Inferior by ≥100%	<0.001
[CoP] C-Stem Cemented Stem Wroblewski Golf Ball	317	2.51	0.36	[-0.88 , 1.61]	Non-inferiority not shown	0.567
[CoP] Exeter V40 Exeter Contemporary Flanged	269	2.10	-0.05	[-0.90 , 0.80]	Non-inferiority not shown	0.913
[CoP] Exeter V40 Trilogy	494	1.91	-0.23	[-0.96 , 0.50]	Non-inferiority not shown	0.537
[CoP] Furlong HAC Stem CSF	2,520	2.65	0.51	[-0.02 , 1.03]	Non-inferiority not shown	0.058
[MoP] ABG II Monolithic Cementless Stem ABG II Cementless Cup	278	4.37	2.22	[0.21 , 4.24]	Non-inferiority not shown	0.031
[MoP] C-Stem Cemented Stem Charnley Ogee	293	2.25	0.11	[-0.97 , 1.19]	Non-inferiority not shown	0.846
[MoP] C-Stem Cemented Stem Charnley and Elite Plus LPW	725	2.45	0.30	[-0.54 , 1.14]	Non-inferiority not shown	0.481
[MoP] C-Stem Cemented Stem Elite Plus Cemented Cup	267	2.49	0.35	[-1.08 , 1.79]	Non-inferiority not shown	0.632
[MoP] C-Stem Cemented Stem Elite Plus Ogee	882	2.57	0.43	[-0.33 , 1.19]	Non-inferiority not shown	0.271
[MoP] C-Stem Cemented Stem Opera	356	3.79	1.64	[0.21 , 3.08]	Non-inferiority not shown	0.025
[MoP] C-Stem Cemented Stem Wroblewski Golf Ball	259	2.47	0.33	[-0.94 , 1.59]	Non-inferiority not shown	0.614
[MoP] CPT Elite Plus Ogee	408	3.16	1.01	[0.04 , 1.98]	Non-inferiority not shown	0.041
[MoP] CPT Trilogy	1,135	4.29	2.14	[1.42 , 2.86]	Inferior by ≥20%	<0.001
[MoP] CPT ZCA	1,233	3.71	1.57	[0.95 , 2.19]	Inferior by ≥20%	<0.001
[MoP] Charnley Cemented Stem Charnley Cemented Cup	1,751	3.52	1.38	[0.69 , 2.07]	Inferior by ≥20%	<0.001
[MoP] Charnley Cemented Stem Charnley Ogee	3,102	3.93	1.78	[1.23 , 2.34]	Inferior by ≥20%	<0.001
[MoP] Charnley Cemented Stem Charnley and Elite Plus LPW	2,284	2.43	0.29	[-0.25 , 0.83]	Non-inferiority not shown	0.297
[MoP] Charnley Cemented Stem Opera	254	3.99	1.85	[0.19 , 3.51]	Non-inferiority not shown	0.029
[MoP] Charnley Cemented Stem Wroblewski Golf Ball	419	2.42	0.28	[-0.81 , 1.36]	Non-inferiority not shown	0.619
[MoP] Corail Duraloc Cementless Cup	1,200	5.37	3.22	[2.31 , 4.14]	Inferior by ≥100%	<0.001
[MoP] Corail Pinnacle	1,681	3.05	0.91	[0.45 , 1.36]	Inferior by ≥20%	<0.001
[MoP] Elite Plus Cemented Stem Elite Plus Ogee	351	2.03	-0.11	[-1.41 , 1.19]	Non-inferiority not shown	0.865
[MoP] Exeter V40 ABG II Cementless Cup	429	2.62	0.48	[-0.77 , 1.73]	Non-inferiority not shown	0.456
[MoP] Exeter V40 Cenator Cemented Cup	694	2.64	0.49	[-0.29 , 1.28]	Non-inferiority not shown	0.219
[MoP] Exeter V40 Charnley Ogee	561	1.62	-0.52	[-1.26 , 0.21]	Non-inferior	0.163
[MoP] Exeter V40 Charnley and Elite Plus LPW	404	2.44	0.30	[-0.53 , 1.12]	Non-inferiority not shown	0.484
[MoP] Exeter V40 Duraloc Cementless Cup	451	4.19	2.05	[0.62 , 3.48]	Inferior by ≥20%	0.005
[MoP] Exeter V40 Elite Plus Cemented Cup	725	1.44	-0.70	[-1.31 , -0.09]	Non-inferior	0.024
[MoP] Exeter V40 Exeter Contemporary Flanged	4,653	2.28	0.13	[-0.23 , 0.49]	Non-inferiority not shown	0.469

For peer review only - http://bmjopen.bmj.com/site/about/guidelines.xhtml

[MoP] Exeter V40 Exeter Contemporary Hooded	2,355	4.12	1.97	[1.45 , 2.49]	Inferior by ≥20%	<0.001
[MoP] Exeter V40 Exeter Duration	2,967	3.71	1.56	[1.05 , 2.07]	Inferior by ≥20%	<0.001
[MoP] Exeter V40 Opera	443	3.23	1.09	[-0.03 , 2.21]	Non-inferiority not shown	0.057
[MoP] Exeter V40 Reflection Cementless	393	4.18	2.03	[0.76 , 3.30]	Inferior by ≥20%	0.002
[MoP] Exeter V40 Trident	1,116	2.71	0.56	[0.05 , 1.08]	Non-inferiority not shown	0.031
[MoP] Exeter V40 Trilogy	1,538	2.49	0.35	[-0.17 , 0.87]	Non-inferiority not shown	0.191
[MoP] Exeter V40 Ultima Cemented Cup	498	3.27	1.12	[-0.03 , 2.27]	Non-inferiority not shown	0.056
[MoP] Furlong Cemented Stem JRI Cemented Cup	584	3.23	1.09	[0.01 , 2.17]	Non-inferiority not shown	0.048
[MoP] Furlong HAC Stem CSF	1,697	4.41	2.27	[1.61 , 2.92]	Inferior by ≥20%	<0.001
[MoP] Muller-Biomet Apollo	371	2.67	0.53	[-0.56 , 1.62]	Non-inferiority not shown	0.345
[MoP] Omnifit Cemented Stem ODC	458	3.89	1.74	[0.35 , 3.14]	Non-inferiority not shown	0.014
[MoP] SP II Cemented Stem Interplanta	258	5.45	3.31	[1.32 , 5.30]	Inferior by ≥20%	0.001
[MoP] Stanmore Modular Stem Stanmore-Arcom Cup	669	2.52	0.38	[-0.29 , 1.04]	Non-inferiority not shown	0.264
[MoP] Synergy Cementless Stem Reflection Cementless	322	2.38	0.24	[-0.80 , 1.28]	Non-inferiority not shown	0.651
[MoP] Versys Cementless Stem Trilogy	464	5.47	3.32	[1.77 , 4.87]	Inferior by ≥20%	<0.001

For peer review only

Supplemental table 2a: Difference in Kaplan-Meier failure estimate between a contemporary reference and implants with at least 250 at risk at 3 years since primary in females

Stem/cup brand	Number at risk	Cumulative failure (%)	Difference in failure (%)	95% CI	Equivalence status	p-value
[CoP] MS-30 Original ME Muller Low Profile Cup	1,096	0.41	[REFERENCE]			
[CoC] ABG II Monolithic Cementless Stem ABG II Cementless Cup	502	1.36	0.96	[-0.11 , 2.02]	Non-inferiority not shown	0.079
[CoC] ABG II Monolithic Cementless Stem Trident	465	2.24	1.84	[0.48 , 3.19]	Inferior by ≥100%	0.008
[CoC] Accolade Trident	3,662	1.78	1.37	[0.83 , 1.92]	Inferior by ≥100%	<0.001
[CoC] Bimetric Cementless Stem Exceed ABT	511	1.18	0.77	[-0.17 , 1.70]	Non-inferiority not shown	0.108
[CoC] C-Stem AMT Cemented Stem Pinnacle	487	1.06	0.65	[-0.22 , 1.52]	Non-inferiority not shown	0.143
[CoC] CPT Continuum	481	1.17	0.76	[-0.13 , 1.64]	Non-inferiority not shown	0.093
[CoC] CPT Trilogy AB	305	0.97	0.56	[-0.59 , 1.71]	Non-inferiority not shown	0.339
[CoC] Corail Delta TT	453	1.90	1.49	[0.44 , 2.54]	Inferior by ≥100%	0.005
[CoC] Corail DeltaMotion	696	1.22	0.81	[-0.02 , 1.65]	Non-inferiority not shown	0.056
[CoC] Corail Duraloc Option	580	1.68	1.27	[0.18 , 2.36]	Inferior by ≥20%	0.023
[CoC] Corail Pinnacle	14,431	1.67	1.26	[0.85 , 1.66]	Inferior by ≥100%	<0.001
[CoC] Corail Pinnacle Gription	292	2.28	1.87	[0.57 , 3.17]	Inferior by ≥100%	0.005
[CoC] Corail Trinity	256	0.21	-0.20	[-0.74 , 0.35]	Non-inferiority not shown	0.474
[CoC] Excia Cementless Plasmacup SC	471	1.52	1.11	[0.06 , 2.17]	Non-inferiority not shown	0.038
[CoC] Exeter V40 ABG II Cementless Cup	887	0.40	-0.01	[-0.54 , 0.52]	Non-inferiority not shown	0.964
[CoC] Exeter V40 Trident	5,950	0.91	0.51	[0.08 , 0.93]	Non-inferiority not shown	0.020
[CoC] Furlong Evolution Cementless Furlong HAC CSF Plus	323	0.90	0.49	[-0.17 , 1.16]	Non-inferiority not shown	0.147
[CoC] Furlong HAC Stem CSF	881	1.54	1.13	[0.26 , 2.01]	Inferior by ≥20%	0.011
[CoC] Furlong HAC Stem Furlong HAC CSF Plus	4,954	1.49	1.08	[0.62 , 1.54]	Inferior by ≥100%	<0.001
[CoC] M/L Taper Cementless Continuum	689	1.52	1.11	[0.24 , 1.98]	Inferior by ≥20%	0.012
[CoC] Metafix Stem Trinity	469	1.43	1.03	[0.13 , 1.93]	Inferior by ≥20%	0.025
[CoC] Omnifit Cementless Stem Trident	267	2.20	1.79	[0.01 , 3.57]	Non-inferiority not shown	0.048
[CoC] Polarstem Cementless R3 Cementless	460	1.10	0.70	[-0.19 , 1.59]	Non-inferiority not shown	0.125
[CoC] S-Rom Cementless Stem Pinnacle	327	2.46	2.06	[0.50 , 3.61]	Inferior by ≥100%	0.010
[CoC] SL-Plus Cementless Stem EP-Fit Plus	602	1.75	1.34	[0.26 , 2.43]	Inferior by ≥20%	0.015
[CoC] SPS Modular April - Ceramic	302	2.39	1.98	[0.31 , 3.66]	Inferior by ≥20%	0.020
[CoC] Summit Cementless Stem Pinnacle	269	1.14	0.73	[-0.44 , 1.90]	Non-inferiority not shown	0.223
[CoC] Taperloc Cementless Stem Exceed ABT	3,728	1.43	1.02	[0.54 , 1.50]	Inferior by ≥100%	<0.001
[CoC] miniHip Trinity	441	1.11	0.70	[-0.19 , 1.60]	Non-inferiority not shown	0.125
[CoP] Accolade Trident	1,760	1.39	0.98	[0.40 , 1.56]	Inferior by ≥20%	0.001
[CoP] C-Stem Cemented Stem Elite Plus Ogee	342	0.28	-0.12	[-0.79 , 0.54]	Non-inferiority not shown	0.716
[CoP] C-Stem Cemented Stem Marathon	626	1.01	0.60	[-0.13 , 1.33]	Non-inferiority not shown	0.108
[CoP] C-Stem Cemented Stem Opera	445	0.22	-0.19	[-0.75 , 0.38]	Non-inferiority not shown	0.515
[CoP] C-Stem Cemented Stem Wroblewski Golf Ball	517	0.53	0.12	[-0.57 , 0.82]	Non-inferiority not shown	0.732
[CoP] CPT Trilogy	871	1.24	0.84	[0.27 , 1.40]	Inferior by ≥20%	0.004
[CoP] Corail Charnley and Elite Plus LPW	376	2.14	1.73	[0.30 , 3.16]	Inferior by ≥20%	0.018
[CoP] Corail Elite Plus Cemented Cup	267	0.91	0.50	[-0.58 , 1.58]	Non-inferiority not shown	0.367
[CoP] Corail Elite Plus Ogee	268	1.67	1.26	[-0.23 , 2.76]	Non-inferiority not shown	0.098
[CoP] Corail Marathon	787	0.72	0.31	[-0.26 , 0.89]	Non-inferiority not shown	0.285

For peer review only - http://bmjopen.bmj.com/site/about/guidelines.xhtml

[CoP] Corail Pinnacle	4,637	1.27	0.86	[0.43 , 1.29]	Inferior by ≥100%	<0.001
[CoP] Corail Trilogy	437	0.87	0.46	[-0.46 , 1.39]	Non-inferiority not shown	0.324
[CoP] Exeter V40 Charnley and Elite Plus LPW	374	1.99	1.58	[0.30 , 2.86]	Inferior by ≥20%	0.015
[CoP] Exeter V40 Elite Plus Ogee	655	0.57	0.16	[-0.45 , 0.77]	Non-inferiority not shown	0.611
[CoP] Exeter V40 Exeter Contemporary Flanged	1,849	1.14	0.73	[0.18 , 1.28]	Inferior by ≥20%	0.009
[CoP] Exeter V40 Exeter Contemporary Hooded	482	1.83	1.42	[0.29 , 2.56]	Inferior by ≥20%	0.014
[CoP] Exeter V40 Exeter Duration	476	0.93	0.52	[-0.37 , 1.40]	Non-inferiority not shown	0.251
[CoP] Exeter V40 Exeter X3 Rimfit	1,264	0.76	0.35	[-0.14 , 0.84]	Non-inferiority not shown	0.164
[CoP] Exeter V40 Marathon	284	0.92	0.51	[-0.48 , 1.49]	Non-inferiority not shown	0.312
[CoP] Exeter V40 Pinnacle	292	1.12	0.71	[-0.20 , 1.62]	Non-inferiority not shown	0.127
[CoP] Exeter V40 Trident	2,940	0.93	0.52	[0.08 , 0.96]	Inferior by ≥20%	0.020
[CoP] Exeter V40 Trilogy	1,182	0.60	0.19	[-0.36 , 0.74]	Non-inferiority not shown	0.491
[CoP] Exeter V40 Tritanium	250	1.74	1.33	[0.12 , 2.55]	Inferior by ≥20%	0.032
[CoP] Furlong HAC Stem CSF	3,748	1.32	0.91	[0.41 , 1.41]	Inferior by ≥100%	<0.001
[CoP] Furlong HAC Stem Furlong HAC CSF Plus	840	2.36	1.95	[0.99 , 2.91]	Inferior by ≥100%	<0.001
[CoP] SL-Plus Cementless Stem Bicon-Plus	342	1.66	1.25	[-0.11 , 2.61]	Non-inferiority not shown	0.073
[CoP] SL-Plus Cementless Stem EP-Fit Plus	593	1.93	1.52	[0.38 , 2.66]	Inferior by ≥20%	0.009
[CoP] Stanmore Modular Stem Stanmore-Arcom Cup	262	1.58	1.17	[-0.25 , 2.59]	Non-inferiority not shown	0.106
[CoP] Taperloc Cementless Stem Exceed ABT	1,062	0.99	0.59	[0.01 , 1.16]	Non-inferiority not shown	0.046
[MoP] Accolade Trident	5,487	1.75	1.34	[0.86 , 1.82]	Inferior by ≥100%	<0.001
[MoP] Anthology R3 Cementless	729	1.46	1.05	[0.29 , 1.81]	Inferior by ≥20%	0.007
[MoP] C-Stem AMT Cemented Stem Charnley and Elite Plus LPW	1,437	0.91	0.50	[-0.07 , 1.07]	Non-inferiority not shown	0.085
[MoP] C-Stem AMT Cemented Stem Elite Plus Cemented Cup	438	0.61	0.20	[-0.57 , 0.97]	Non-inferiority not shown	0.614
[MoP] C-Stem AMT Cemented Stem Elite Plus Ogee	1,135	0.84	0.43	[-0.15 , 1.01]	Non-inferiority not shown	0.149
[MoP] C-Stem AMT Cemented Stem Marathon	751	0.88	0.47	[-0.10 , 1.04]	Non-inferiority not shown	0.108
[MoP] C-Stem AMT Cemented Stem Pinnacle	985	1.24	0.84	[0.25 , 1.42]	Inferior by ≥20%	0.005
[MoP] C-Stem Cemented Stem Charnley Ogee	597	1.48	1.07	[0.09 , 2.05]	Inferior by ≥20%	0.033
[MoP] C-Stem Cemented Stem Charnley and Elite Plus LPW	1,119	1.17	0.76	[0.05 , 1.46]	Non-inferiority not shown	0.035
[MoP] C-Stem Cemented Stem Duraloc Cementless Cup	420	1.59	1.18	[-0.04 , 2.41]	Non-inferiority not shown	0.058
[MoP] C-Stem Cemented Stem Elite Plus Cemented Cup	390	0.47	0.06	[-0.68 , 0.80]	Non-inferiority not shown	0.870
[MoP] C-Stem Cemented Stem Elite Plus Ogee	1,985	0.69	0.28	[-0.21 , 0.78]	Non-inferiority not shown	0.257
[MoP] C-Stem Cemented Stem Marathon	959	0.63	0.22	[-0.30 , 0.73]	Non-inferiority not shown	0.407
[MoP] C-Stem Cemented Stem Opera	836	0.68	0.27	[-0.38 , 0.91]	Non-inferiority not shown	0.417
[MoP] C-Stem Cemented Stem Pinnacle	301	1.59	1.19	[-0.13 , 2.50]	Non-inferiority not shown	0.077
[MoP] C-Stem Cemented Stem Wroblewski Golf Ball	625	0.74	0.33	[-0.41 , 1.07]	Non-inferiority not shown	0.379
[MoP] CCA Cemented Stem CCB Cup	793	0.62	0.21	[-0.40 , 0.82]	Non-inferiority not shown	0.501
[MoP] CLS Cementless Stem Allofit	336	1.93	1.52	[0.06 , 2.99]	Non-inferiority not shown	0.041
[MoP] CLS Cementless Stem Trilogy	321	3.16	2.75	[0.88 , 4.62]	Inferior by ≥100%	0.004
[MoP] CMK Modular Cemented Stem CMK Cemented Cup	319	0.30	-0.11	[-0.79 , 0.58]	Non-inferiority not shown	0.755
[MoP] CPCS Opera	804	0.95	0.55	[-0.20 , 1.30]	Non-inferiority not shown	0.153
[MoP] CPCS Polarcup Cementless	259	0.76	0.35	[-0.76 , 1.46]	Non-inferiority not shown	0.535
[MoP] CPCS Reflection Cemented	264	1.10	0.69	[-0.26 , 1.64]	Non-inferiority not shown	0.156
[MoP] CPS Plus Cenator Cemented Cup	268	0.37	-0.04	[-0.85 , 0.77]	Non-inferiority not shown	0.931
[MoP] CPS Plus Opera	327	1.17	0.76	[-0.43 , 1.96]	Non-inferiority not shown	0.211
[MoP] CPT Allofit	485	0.32	-0.09	[-0.66 , 0.48]	Non-inferiority not shown	0.752
[MoP] CPT Continuum	315	2.66	2.25	[1.17 , 3.33]	Inferior by ≥100%	<0.001

For peer review only

[MoP] CPT Elite Plus Ogee	1,550	1.24	0.83	[0.21 , 1.46]	Inferior by ≥20%	0.009
[MoP] CPT Exeter Contemporary Flanged	283	2.17	1.76	[0.31 , 3.21]	Inferior by ≥20%	0.017
[MoP] CPT Opera	268	1.06	0.66	[-0.59 , 1.91]	Non-inferiority not shown	0.303
[MoP] CPT Original ME Muller Low Profile Cup	600	1.30	0.89	[0.00 , 1.78]	Non-inferiority not shown	0.050
[MoP] CPT Pinnacle	511	1.59	1.19	[0.09 , 2.28]	Inferior by ≥20%	0.033
[MoP] CPT Trabecular Metal Modular Cementless Cup	645	1.72	1.32	[0.40 , 2.23]	Inferior by ≥20%	0.005
[MoP] CPT Trilogy	5,622	1.20	0.79	[0.35 , 1.22]	Inferior by ≥20%	<0.001
[MoP] CPT Trilogy IT	304	2.93	2.52	[1.48 , 3.57]	Inferior by ≥100%	<0.001
[MoP] CPT ZCA	5,377	1.34	0.93	[0.49 , 1.38]	Inferior by ≥100%	<0.001
[MoP] Centrament Chirulen	294	0.00	- -	[-.-- , -.--]	No failures to date	
[MoP] Charnley Cemented Stem Charnley Cemented Cup	2,552	0.97	0.56	[0.04 , 1.07]	Non-inferiority not shown	0.033
[MoP] Charnley Cemented Stem Charnley Ogee	5,409	1.08	0.67	[0.22 , 1.11]	Inferior by ≥20%	0.003
[MoP] Charnley Cemented Stem Charnley and Elite Plus LPW	4,125	0.61	0.20	[-0.22 , 0.63]	Non-inferiority not shown	0.352
[MoP] Charnley Cemented Stem Opera	823	0.70	0.29	[-0.37 , 0.96]	Non-inferiority not shown	0.385
[MoP] Charnley Cemented Stem Wroblewski Golf Ball	609	1.53	1.12	[0.11 , 2.12]	Inferior by ≥20%	0.029
[MoP] Charnley Modular Charnley and Elite Plus LPW	275	0.36	-0.05	[-0.84 , 0.75]	Non-inferiority not shown	0.908
[MoP] Corail Charnley and Elite Plus LPW	543	0.83	0.42	[-0.33 , 1.17]	Non-inferiority not shown	0.274
[MoP] Corail Duraloc Cementless Cup	2,139	1.30	0.89	[0.30 , 1.48]	Inferior by ≥20%	0.003
[MoP] Corail Elite Plus Cemented Cup	629	0.42	0.02	[-0.58 , 0.62]	Non-inferiority not shown	0.955
[MoP] Corail Elite Plus Ogee	937	1.08	0.67	[-0.01 , 1.36]	Non-inferiority not shown	0.055
[MoP] Corail Exeter Contemporary Flanged	376	0.95	0.54	[-0.37 , 1.45]	Non-inferiority not shown	0.246
[MoP] Corail Marathon	1,873	0.80	0.39	[-0.08 , 0.87]	Non-inferiority not shown	0.107
[MoP] Corail Pinnacle	17,132	1.26	0.85	[0.47 , 1.23]	Inferior by ≥100%	<0.001
[MoP] Corail Trident	385	1.01	0.60	[-0.23 , 1.43]	Non-inferiority not shown	0.154
[MoP] Corail Trilogy	1,066	0.90	0.50	[-0.15 , 1.14]	Non-inferiority not shown	0.129
[MoP] Elite Plus Cemented Stem Elite Plus Ogee	402	0.49	0.08	[-0.68 , 0.84]	Non-inferiority not shown	0.842
[MoP] Exeter V40 ABG II Cementless Cup	477	0.80	0.39	[-0.47 , 1.25]	Non-inferiority not shown	0.373
[MoP] Exeter V40 Cenator Cemented Cup	1,425	1.45	1.04	[0.35 , 1.73]	Inferior by ≥20%	0.003
[MoP] Exeter V40 Charnley Ogee	1,068	1.00	0.59	[-0.08 , 1.26]	Non-inferiority not shown	0.083
[MoP] Exeter V40 Charnley and Elite Plus LPW	1,811	1.31	0.90	[0.31 , 1.50]	Inferior by ≥20%	0.003
[MoP] Exeter V40 Duraloc Cementless Cup	708	1.10	0.69	[-0.15 , 1.53]	Non-inferiority not shown	0.106
[MoP] Exeter V40 EP-Fit Plus	333	1.17	0.77	[-0.43 , 1.96]	Non-inferiority not shown	0.210
[MoP] Exeter V40 Elite Plus Cemented Cup	2,389	0.45	0.04	[-0.39 , 0.48]	Non-inferiority not shown	0.842
[MoP] Exeter V40 Elite Plus Ogee	10,898	0.70	0.29	[-0.10 , 0.67]	Non-inferiority not shown	0.142
[MoP] Exeter V40 Exceed	333	0.85	0.44	[-0.58 , 1.46]	Non-inferiority not shown	0.397
[MoP] Exeter V40 Exceed ABT	284	0.94	0.53	[-0.45 , 1.52]	Non-inferiority not shown	0.290
[MoP] Exeter V40 Exeter Contemporary Flanged	27,147	0.71	0.30	[-0.07 , 0.67]	Non-inferiority not shown	0.113
[MoP] Exeter V40 Exeter Contemporary Hooded	10,906	1.39	0.98	[0.58 , 1.39]	Inferior by ≥100%	<0.001
[MoP] Exeter V40 Exeter Duration	8,897	1.05	0.64	[0.23 , 1.05]	Inferior by ≥20%	0.002
[MoP] Exeter V40 Exeter X3 Rimfit	3,408	0.73	0.32	[-0.09 , 0.73]	Non-inferiority not shown	0.123
[MoP] Exeter V40 Furlong HAC CSF Plus	340	0.17	-0.24	[-0.73 , 0.25]	Non-inferiority not shown	0.333
[MoP] Exeter V40 Marathon	884	0.88	0.47	[-0.12 , 1.06]	Non-inferiority not shown	0.119
[MoP] Exeter V40 Opera	1,660	0.72	0.31	[-0.22 , 0.84]	Non-inferiority not shown	0.248
[MoP] Exeter V40 Pinnacle	1,807	0.97	0.56	[0.05 , 1.07]	Non-inferiority not shown	0.033
[MoP] Exeter V40 R3 Cementless	363	0.90	0.49	[-0.27 , 1.26]	Non-inferiority not shown	0.205
[MoP] Exeter V40 Reflection Cementless	1,308	0.73	0.32	[-0.25 , 0.90]	Non-inferiority not shown	0.273

For peer review only

[MoP] Exeter V40 Trabecular Metal Modular Cementless Cup	286	1.55	1.14	[-0.14 , 2.42]	Non-inferiority not shown	0.081
[MoP] Exeter V40 Trident	11,298	0.97	0.57	[0.18 , 0.95]	Inferior by ≥20%	0.004
[MoP] Exeter V40 Trilogy	5,145	0.83	0.43	[0.00 , 0.85]	Non-inferiority not shown	0.050
[MoP] Exeter V40 Tritanium	282	1.60	1.19	[0.12 , 2.25]	Inferior by ≥20%	0.029
[MoP] Exeter V40 Ultima Cemented Cup	777	1.10	0.69	[-0.11 , 1.49]	Non-inferiority not shown	0.090
[MoP] Furlong Cemented Stem JRI Cemented Cup	1,036	1.43	1.02	[0.24 , 1.81]	Inferior by ≥20%	0.010
[MoP] Furlong HAC Stem CSF	4,074	2.18	1.77	[1.22 , 2.33]	Inferior by ≥100%	<0.001
[MoP] Furlong HAC Stem Furlong HAC CSF Plus	1,829	2.12	1.71	[1.07 , 2.36]	Inferior by ≥100%	<0.001
[MoP] Furlong HAC Stem Furlong Threaded	311	1.83	1.42	[-0.07 , 2.92]	Non-inferiority not shown	0.062
[MoP] M/L Taper Cementless Continuum	381	1.72	1.31	[0.24 , 2.37]	Inferior by ≥20%	0.016
[MoP] M/L Taper Cementless Trilogy	281	2.08	1.68	[0.34 , 3.01]	Inferior by ≥20%	0.014
[MoP] MS-30 Original ME Muller Low Profile Cup	522	0.28	-0.13	[-0.66 , 0.40]	Non-inferiority not shown	0.637
[MoP] Muller Straight Stem Centerpulse Muller	328	0.88	0.47	[-0.58 , 1.52]	Non-inferiority not shown	0.382
[MoP] Muller Straight Stem Original ME Muller Low Profile Cup	999	0.92	0.52	[-0.12 , 1.15]	Non-inferiority not shown	0.112
[MoP] Muller-Biomet Apollo	1,201	1.32	0.91	[0.19 , 1.63]	Inferior by ≥20%	0.013
[MoP] Muller-Biomet Original ME Muller Low Profile Cup	707	1.32	0.91	[0.02 , 1.80]	Non-inferiority not shown	0.044
[MoP] Omnifit Cemented Stem ODC	614	1.56	1.15	[0.13 , 2.18]	Inferior by ≥20%	0.027
[MoP] Omnifit Cementless Stem Trident	251	2.95	2.55	[0.50 , 4.60]	Inferior by ≥100%	0.015
[MoP] P10 Muller Original ME Muller Low Profile Cup	268	1.03	0.62	[-0.59 , 1.83]	Non-inferiority not shown	0.317
[MoP] Polarstem Cementless R3 Cementless	592	0.73	0.33	[-0.19 , 0.85]	Non-inferiority not shown	0.217
[MoP] SL-Plus Cementless Stem EP-Fit Plus	1,198	2.40	2.00	[1.14 , 2.85]	Inferior by ≥100%	<0.001
[MoP] SP II Cemented Stem Interplanta	444	1.84	1.43	[0.19 , 2.68]	Inferior by ≥20%	0.024
[MoP] Stanmore Modular Stem Elite Plus Cemented Cup	367	0.26	-0.15	[-0.77 , 0.48]	Non-inferiority not shown	0.647
[MoP] Stanmore Modular Stem SHP Cup	664	0.75	0.34	[-0.36 , 1.04]	Non-inferiority not shown	0.339
[MoP] Stanmore Modular Stem Stanmore-Arcom Cup	2,729	1.12	0.71	[0.19 , 1.23]	Inferior by ≥20%	0.007
[MoP] Synergy Cementless Stem R3 Cementless	554	1.00	0.59	[-0.08 , 1.26]	Non-inferiority not shown	0.082
[MoP] Synergy Cementless Stem Reflection Cementless	859	0.68	0.27	[-0.38 , 0.92]	Non-inferiority not shown	0.416
[MoP] Taperfit Cemented Stem Atlas IIIp	327	1.14	0.73	[-0.33 , 1.79]	Non-inferiority not shown	0.177
[MoP] Taperloc Cementless Stem Exceed	318	1.20	0.79	[-0.43 , 2.01]	Non-inferiority not shown	0.204
[MoP] Taperloc Cementless Stem Exceed ABT	2,287	1.98	1.57	[0.98 , 2.16]	Inferior by ≥100%	<0.001
[MoP] Versys Cementless Stem Trilogy	492	3.79	3.38	[1.71 , 5.05]	Inferior by ≥100%	<0.001

For peer review only

Supplemental table 2b: Difference in Kaplan-Meier failure estimate between a contemporary reference and implants with at least 250 at risk at 5 years since primary in females

Stem/cup brand	Number at risk	Cumulative failure (%)	Difference in failure (%)	95% CI	Equivalence status	p-value
[MoP] Exeter V40 Elite Plus Cemented Cup	1,769	0.54	[REFERENCE]			
[CoC] ABG II Monolithic Cementless Stem ABG II Cementless Cup	485	1.76	1.22	[0.05 , 2.40]	Non-inferiority not shown	0.041
[CoC] ABG II Monolithic Cementless Stem Trident	372	2.95	2.41	[0.86 , 3.96]	Inferior by ≥100%	0.002
[CoC] Accolade Trident	2,906	2.56	2.02	[1.44 , 2.59]	Inferior by ≥100%	<0.001
[CoC] Bimetric Cementless Stem Exceed ABT	369	1.84	1.30	[0.13 , 2.48]	Inferior by ≥20%	0.030
[CoC] C-Stem AMT Cemented Stem Pinnacle	268	1.80	1.26	[0.08 , 2.44]	Non-inferiority not shown	0.037
[CoC] CPT Trilogy AB	278	1.64	1.10	[-0.35 , 2.55]	Non-inferiority not shown	0.138
[CoC] Corail DeltaMotion	373	1.47	0.93	[-0.01 , 1.86]	Non-inferiority not shown	0.051
[CoC] Corail Duraloc Option	566	2.70	2.16	[0.83 , 3.49]	Inferior by ≥100%	0.001
[CoC] Corail Pinnacle	9,650	2.16	1.62	[1.26 , 1.97]	Inferior by ≥100%	<0.001
[CoC] Excia Cementless Plasmacup SC	266	1.52	0.98	[-0.04 , 2.01]	Non-inferiority not shown	0.061
[CoC] Exeter V40 ABG II Cementless Cup	760	1.10	0.56	[-0.17 , 1.30]	Non-inferiority not shown	0.133
[CoC] Exeter V40 Trident	4,852	1.27	0.74	[0.35 , 1.13]	Inferior by ≥20%	<0.001
[CoC] Furlong HAC Stem CSF	839	2.11	1.57	[0.59 , 2.55]	Inferior by ≥100%	0.002
[CoC] Furlong HAC Stem Furlong HAC CSF Plus	3,077	1.78	1.24	[0.81 , 1.68]	Inferior by ≥100%	<0.001
[CoC] M/L Taper Cementless Continuum	304	1.68	1.14	[0.25 , 2.04]	Inferior by ≥20%	0.012
[CoC] S-Rom Cementless Stem Pinnacle	251	3.21	2.67	[0.83 , 4.52]	Inferior by ≥100%	0.004
[CoC] SL-Plus Cementless Stem EP-Fit Plus	565	3.43	2.89	[1.42 , 4.36]	Inferior by ≥100%	<0.001
[CoC] Taperloc Cementless Stem Exceed ABT	2,243	1.79	1.25	[0.78 , 1.72]	Inferior by ≥100%	<0.001
[CoP] Accolade Trident	828	1.84	1.31	[0.66 , 1.95]	Inferior by ≥100%	<0.001
[CoP] C-Stem Cemented Stem Elite Plus Ogee	262	0.62	0.08	[-0.82 , 0.98]	Non-inferiority not shown	0.860
[CoP] C-Stem Cemented Stem Marathon	297	1.21	0.67	[-0.13 , 1.48]	Non-inferiority not shown	0.099
[CoP] C-Stem Cemented Stem Opera	374	1.20	0.66	[-0.42 , 1.74]	Non-inferiority not shown	0.232
[CoP] C-Stem Cemented Stem Wroblewski Golf Ball	417	0.73	0.19	[-0.57 , 0.95]	Non-inferiority not shown	0.628
[CoP] Corail Charnley and Elite Plus LPW	321	2.42	1.88	[0.37 , 3.39]	Inferior by ≥20%	0.015
[CoP] Corail Marathon	333	1.04	0.50	[-0.19 , 1.19]	Non-inferiority not shown	0.153
[CoP] Corail Pinnacle	2,442	1.71	1.17	[0.75 , 1.59]	Inferior by ≥100%	<0.001
[CoP] Corail Trilogy	397	1.10	0.56	[-0.44 , 1.56]	Non-inferiority not shown	0.269
[CoP] Exeter V40 Elite Plus Ogee	478	1.06	0.52	[-0.27 , 1.31]	Non-inferiority not shown	0.198
[CoP] Exeter V40 Exeter Contemporary Flanged	1,225	1.60	1.06	[0.45 , 1.66]	Inferior by ≥20%	0.001
[CoP] Exeter V40 Exeter Contemporary Hooded	358	2.31	1.77	[0.48 , 3.06]	Inferior by ≥20%	0.007
[CoP] Exeter V40 Exeter Duration	405	0.93	0.39	[-0.47 , 1.24]	Non-inferiority not shown	0.374
[CoP] Exeter V40 Trident	1,446	1.28	0.75	[0.29 , 1.20]	Inferior by ≥20%	0.001
[CoP] Exeter V40 Trilogy	997	0.86	0.32	[-0.26 , 0.89]	Non-inferiority not shown	0.278
[CoP] Furlong HAC Stem CSF	3,217	1.83	1.29	[0.79 , 1.79]	Inferior by ≥100%	<0.001
[CoP] Furlong HAC Stem Furlong HAC CSF Plus	508	2.55	2.01	[1.01 , 3.01]	Inferior by ≥100%	<0.001
[CoP] MS-30 Original ME Muller Low Profile Cup	796	0.64	0.10	[-0.45 , 0.65]	Non-inferiority not shown	0.725
[CoP] SL-Plus Cementless Stem Bicon-Plus	306	3.49	2.95	[0.99 , 4.91]	Inferior by ≥100%	0.003
[CoP] SL-Plus Cementless Stem EP-Fit Plus	499	3.49	2.95	[1.46 , 4.45]	Inferior by ≥100%	<0.001
[CoP] Taperloc Cementless Stem Exceed ABT	466	1.23	0.69	[0.07 , 1.31]	Non-inferiority not shown	0.029

For peer review only

[MoP] Accolade Trident	3,698	2.36	1.82	[1.34 , 2.29]	Inferior by ≥100%	<0.001
[MoP] C-Stem AMT Cemented Stem Charnley and Elite Plus LPW	954	1.29	0.75	[0.13 , 1.37]	Inferior by ≥20%	0.017
[MoP] C-Stem AMT Cemented Stem Elite Plus Cemented Cup	313	0.85	0.31	[-0.57 , 1.18]	Non-inferiority not shown	0.488
[MoP] C-Stem AMT Cemented Stem Elite Plus Ogee	666	1.18	0.64	[-0.02 , 1.30]	Non-inferiority not shown	0.058
[MoP] C-Stem AMT Cemented Stem Pinnacle	426	1.66	1.12	[0.40 , 1.83]	Inferior by ≥20%	0.002
[MoP] C-Stem Cemented Stem Charnley Ogee	480	2.25	1.71	[0.50 , 2.92]	Inferior by ≥20%	0.006
[MoP] C-Stem Cemented Stem Charnley and Elite Plus LPW	1,007	1.81	1.28	[0.46 , 2.09]	Inferior by ≥20%	0.002
[MoP] C-Stem Cemented Stem Duraloc Cementless Cup	388	2.33	1.79	[0.34 , 3.24]	Inferior by ≥20%	0.016
[MoP] C-Stem Cemented Stem Elite Plus Cemented Cup	357	0.47	-0.07	[-0.77 , 0.64]	Non-inferiority not shown	0.848
[MoP] C-Stem Cemented Stem Elite Plus Ogee	1,554	1.02	0.49	[-0.02 , 0.99]	Non-inferiority not shown	0.062
[MoP] C-Stem Cemented Stem Marathon	473	0.91	0.37	[-0.24 , 0.98]	Non-inferiority not shown	0.235
[MoP] C-Stem Cemented Stem Opera	626	0.95	0.41	[-0.30 , 1.13]	Non-inferiority not shown	0.259
[MoP] C-Stem Cemented Stem Wroblewski Golf Ball	488	1.61	1.07	[0.04 , 2.11]	Non-inferiority not shown	0.042
[MoP] CCA Cemented Stem CCB Cup	535	0.92	0.38	[-0.32 , 1.08]	Non-inferiority not shown	0.289
[MoP] CLS Cementless Stem Allofit	316	2.53	1.99	[0.33 , 3.64]	Inferior by ≥20%	0.019
[MoP] CMK Modular Cemented Stem CMK Cemented Cup	291	1.25	0.72	[-0.54 , 1.97]	Non-inferiority not shown	0.263
[MoP] CPCS Opera	586	1.63	1.09	[0.17 , 2.02]	Inferior by ≥20%	0.020
[MoP] CPS Plus Opera	309	1.78	1.25	[-0.20 , 2.69]	Non-inferiority not shown	0.090
[MoP] CPT Allofit	308	0.58	0.04	[-0.69 , 0.77]	Non-inferiority not shown	0.921
[MoP] CPT Elite Plus Ogee	1,115	1.60	1.06	[0.40 , 1.73]	Inferior by ≥20%	0.002
[MoP] CPT Opera	252	1.84	1.30	[-0.32 , 2.92]	Non-inferiority not shown	0.116
[MoP] CPT Original ME Muller Low Profile Cup	322	1.98	1.44	[0.28 , 2.60]	Inferior by ≥20%	0.015
[MoP] CPT Pinnacle	433	2.03	1.49	[0.27 , 2.72]	Inferior by ≥20%	0.017
[MoP] CPT Trabecular Metal Modular Cementless Cup	399	2.48	1.95	[0.79 , 3.10]	Inferior by ≥100%	0.001
[MoP] CPT Trilogy	3,903	2.10	1.56	[1.11 , 2.01]	Inferior by ≥100%	<0.001
[MoP] CPT ZCA	3,998	1.98	1.44	[1.00 , 1.88]	Inferior by ≥100%	<0.001
[MoP] Charnley Cemented Stem Charnley Cemented Cup	2,265	1.42	0.88	[0.35 , 1.41]	Inferior by ≥20%	0.001
[MoP] Charnley Cemented Stem Charnley Ogee	4,710	1.63	1.10	[0.67 , 1.53]	Inferior by ≥100%	<0.001
[MoP] Charnley Cemented Stem Charnley and Elite Plus LPW	3,628	1.00	0.46	[0.05 , 0.86]	Non-inferiority not shown	0.027
[MoP] Charnley Cemented Stem Opera	697	1.35	0.81	[-0.03 , 1.65]	Non-inferiority not shown	0.058
[MoP] Charnley Cemented Stem Wroblewski Golf Ball	487	1.73	1.19	[0.14 , 2.24]	Inferior by ≥20%	0.027
[MoP] Corail Charnley and Elite Plus LPW	332	1.29	0.75	[-0.21 , 1.71]	Non-inferiority not shown	0.126
[MoP] Corail Duraloc Cementless Cup	2,023	2.10	1.56	[0.90 , 2.22]	Inferior by ≥100%	<0.001
[MoP] Corail Elite Plus Cemented Cup	481	0.99	0.45	[-0.39 , 1.29]	Non-inferiority not shown	0.295
[MoP] Corail Elite Plus Ogee	678	1.65	1.11	[0.30 , 1.93]	Inferior by ≥20%	0.007
[MoP] Corail Marathon	921	0.93	0.40	[-0.06 , 0.85]	Non-inferiority not shown	0.089
[MoP] Corail Pinnacle	10,090	1.56	1.03	[0.71 , 1.35]	Inferior by ≥100%	<0.001
[MoP] Corail Trilogy	859	1.22	0.69	[-0.01 , 1.38]	Non-inferiority not shown	0.054
[MoP] Elite Plus Cemented Stem Elite Plus Ogee	362	1.26	0.72	[-0.41 , 1.86]	Non-inferiority not shown	0.211
[MoP] Exeter V40 ABG II Cementless Cup	458	1.01	0.47	[-0.45 , 1.40]	Non-inferiority not shown	0.315
[MoP] Exeter V40 Cenator Cemented Cup	1,153	2.12	1.58	[0.80 , 2.36]	Inferior by ≥100%	<0.001
[MoP] Exeter V40 Charnley Ogee	900	1.39	0.85	[0.12 , 1.59]	Inferior by ≥20%	0.023
[MoP] Exeter V40 Charnley and Elite Plus LPW	1,309	1.61	1.07	[0.46 , 1.68]	Inferior by ≥20%	0.001
[MoP] Exeter V40 Duraloc Cementless Cup	671	1.82	1.28	[0.26 , 2.30]	Inferior by ≥20%	0.014
[MoP] Exeter V40 EP-Fit Plus	312	2.98	2.44	[0.60 , 4.29]	Inferior by ≥100%	0.009
[MoP] Exeter V40 Elite Plus Ogee	8,315	1.02	0.48	[0.15 , 0.81]	Inferior by ≥20%	0.004

For peer review only - http://bmjopen.bmj.com/site/about/guidelines.xhtml

[MoP] Exeter V40 Exceed	250	0.85	0.31	[-0.68 , 1.30]	Non-inferiority not shown	0.541
[MoP] Exeter V40 Exeter Contemporary Flanged	17,934	1.04	0.50	[0.20 , 0.80]	Inferior by ≥20%	0.001
[MoP] Exeter V40 Exeter Contemporary Hooded	7,664	2.02	1.48	[1.10 , 1.85]	Inferior by ≥100%	<0.001
[MoP] Exeter V40 Exeter Duration	7,168	1.43	0.89	[0.53 , 1.25]	Inferior by ≥20%	<0.001
[MoP] Exeter V40 Exeter X3 Rimfit	550	0.92	0.38	[-0.02 , 0.79]	Non-inferiority not shown	0.066
[MoP] Exeter V40 Marathon	364	1.25	0.71	[-0.04 , 1.46]	Non-inferiority not shown	0.064
[MoP] Exeter V40 Opera	1,221	0.98	0.44	[-0.10 , 0.98]	Non-inferiority not shown	0.110
[MoP] Exeter V40 Pinnacle	1,075	1.24	0.70	[0.17 , 1.24]	Inferior by ≥20%	0.010
[MoP] Exeter V40 Reflection Cementless	1,164	1.05	0.51	[-0.10 , 1.13]	Non-inferiority not shown	0.100
[MoP] Exeter V40 Trident	6,743	1.30	0.76	[0.43 , 1.09]	Inferior by ≥20%	<0.001
[MoP] Exeter V40 Trilogy	4,060	1.24	0.70	[0.30 , 1.10]	Inferior by ≥20%	0.001
[MoP] Exeter V40 Ultima Cemented Cup	720	1.90	1.36	[0.37 , 2.35]	Inferior by ≥20%	0.007
[MoP] Furlong Cemented Stem JRI Cemented Cup	892	1.74	1.20	[0.38 , 2.03]	Inferior by ≥20%	0.004
[MoP] Furlong HAC Stem CSF	3,340	2.50	1.96	[1.43 , 2.50]	Inferior by ≥100%	<0.001
[MoP] Furlong HAC Stem Furlong HAC CSF Plus	1,122	2.75	2.21	[1.48 , 2.94]	Inferior by ≥100%	<0.001
[MoP] Furlong HAC Stem Furlong Threaded	269	2.84	2.30	[0.45 , 4.15]	Inferior by ≥20%	0.015
[MoP] MS-30 Original ME Muller Low Profile Cup	366	0.98	0.44	[-0.48 , 1.36]	Non-inferiority not shown	0.351
[MoP] Muller Straight Stem Centerpulse Muller	291	1.85	1.31	[-0.18 , 2.81]	Non-inferiority not shown	0.085
[MoP] Muller Straight Stem Original ME Muller Low Profile Cup	718	1.26	0.72	[0.02 , 1.42]	Non-inferiority not shown	0.044
[MoP] Muller-Biomet Apollo	972	1.50	0.97	[0.24 , 1.69]	Inferior by ≥20%	0.009
[MoP] Muller-Biomet Original ME Muller Low Profile Cup	442	1.79	1.25	[0.25 , 2.26]	Inferior by ≥20%	0.015
[MoP] Omnifit Cemented Stem ODC	559	2.23	1.69	[0.50 , 2.87]	Inferior by ≥20%	0.005
[MoP] SL-Plus Cementless Stem EP-Fit Plus	980	3.12	2.58	[1.62 , 3.54]	Inferior by ≥100%	<0.001
[MoP] SP II Cemented Stem Interplanta	392	2.33	1.79	[0.40 , 3.18]	Inferior by ≥20%	0.012
[MoP] Stanmore Modular Stem Elite Plus Cemented Cup	273	1.19	0.65	[-0.55 , 1.85]	Non-inferiority not shown	0.287
[MoP] Stanmore Modular Stem SHP Cup	483	0.75	0.21	[-0.45 , 0.87]	Non-inferiority not shown	0.534
[MoP] Stanmore Modular Stem Stanmore-Arcom Cup	2,115	1.69	1.15	[0.60 , 1.70]	Inferior by ≥100%	<0.001
[MoP] Synergy Cementless Stem Reflection Cementless	825	0.91	0.38	[-0.31 , 1.06]	Non-inferiority not shown	0.284
[MoP] Taperloc Cementless Stem Exceed	277	1.84	1.30	[-0.18 , 2.79]	Non-inferiority not shown	0.086
[MoP] Taperloc Cementless Stem Exceed ABT	1,136	2.32	1.78	[1.17 , 2.38]	Inferior by ≥100%	<0.001
[MoP] Versys Cementless Stem Trilogy	462	4.99	4.45	[2.56 , 6.34]	Inferior by ≥100%	<0.001

For peer review only

Supplemental table 2c: Difference in Kaplan-Meier failure estimate between a contemporary reference and implants with at least 250 at risk at 7 years since primary in females

Stem/cup brand	Number at risk	Cumulative failure (%)	Difference in failure (%)	95% CI	Equivalence status	p-value
[MoP] Exeter V40 Elite Plus Cemented Cup	1,165	0.76	[REFERENCE]			
[CoC] ABG II Monolithic Cementless Stem ABG II Cementless Cup	441	2.60	1.84	[0.40 , 3.29]	Inferior by ≥20%	0.012
[CoC] ABG II Monolithic Cementless Stem Trident	341	3.21	2.45	[0.80 , 4.10]	Inferior by ≥100%	0.004
[CoC] Accolade Trident	1,744	3.15	2.39	[1.69 , 3.09]	Inferior by ≥100%	<0.001
[CoC] Corail Duraloc Option	466	3.09	2.33	[0.88 , 3.79]	Inferior by ≥100%	0.002
[CoC] Corail Pinnacle	4,397	2.64	1.88	[1.42 , 2.34]	Inferior by ≥100%	<0.001
[CoC] Exeter V40 ABG II Cementless Cup	615	1.55	0.79	[-0.13 , 1.71]	Non-inferiority not shown	0.094
[CoC] Exeter V40 Trident	3,449	1.85	1.09	[0.58 , 1.61]	Inferior by ≥20%	<0.001
[CoC] Furlong HAC Stem CSF	776	3.08	2.32	[1.12 , 3.53]	Inferior by ≥100%	<0.001
[CoC] Furlong HAC Stem Furlong HAC CSF Plus	1,170	2.19	1.43	[0.87 , 2.00]	Inferior by ≥100%	<0.001
[CoC] SL-Plus Cementless Stem EP-Fit Plus	486	3.61	2.85	[1.32 , 4.38]	Inferior by ≥100%	<0.001
[CoC] Taperloc Cementless Stem Exceed ABT	981	1.93	1.17	[0.60 , 1.74]	Inferior by ≥20%	<0.001
[CoP] Accolade Trident	345	1.98	1.22	[0.48 , 1.96]	Inferior by ≥20%	0.001
[CoP] C-Stem Cemented Stem Opera	274	1.84	1.08	[-0.34 , 2.49]	Non-inferiority not shown	0.135
[CoP] C-Stem Cemented Stem Wroblewski Golf Ball	347	1.80	1.05	[-0.27 , 2.37]	Non-inferiority not shown	0.120
[CoP] Corail Pinnacle	1,028	2.07	1.32	[0.74 , 1.89]	Inferior by ≥20%	<0.001
[CoP] Corail Trilogy	310	1.10	0.34	[-0.69 , 1.37]	Non-inferiority not shown	0.513
[CoP] Exeter V40 Elite Plus Ogee	304	1.29	0.54	[-0.41 , 1.49]	Non-inferiority not shown	0.268
[CoP] Exeter V40 Exeter Contemporary Flanged	694	1.78	1.02	[0.32 , 1.73]	Inferior by ≥20%	0.004
[CoP] Exeter V40 Exeter Duration	309	2.02	1.26	[-0.12 , 2.65]	Non-inferiority not shown	0.074
[CoP] Exeter V40 Trident	656	1.46	0.71	[0.13 , 1.28]	Non-inferiority not shown	0.017
[CoP] Exeter V40 Trilogy	723	1.19	0.43	[-0.30 , 1.16]	Non-inferiority not shown	0.249
[CoP] Furlong HAC Stem CSF	2,638	2.37	1.61	[0.99 , 2.22]	Inferior by ≥100%	<0.001
[CoP] MS-30 Original ME Muller Low Profile Cup	489	0.64	-0.12	[-0.72 , 0.48]	Non-inferiority not shown	0.696
[CoP] SL-Plus Cementless Stem EP-Fit Plus	364	4.38	3.63	[1.89 , 5.37]	Inferior by ≥100%	<0.001
[MoP] Accolade Trident	1,704	3.00	2.25	[1.63 , 2.86]	Inferior by ≥100%	<0.001
[MoP] C-Stem AMT Cemented Stem Charnley and Elite Plus LPW	534	1.65	0.89	[0.06 , 1.71]	Non-inferiority not shown	0.035
[MoP] C-Stem AMT Cemented Stem Elite Plus Ogee	378	1.98	1.22	[0.17 , 2.28]	Inferior by ≥20%	0.023
[MoP] C-Stem Cemented Stem Charnley Ogee	384	2.49	1.73	[0.41 , 3.05]	Inferior by ≥20%	0.010
[MoP] C-Stem Cemented Stem Charnley and Elite Plus LPW	915	2.34	1.58	[0.61 , 2.54]	Inferior by ≥20%	0.001
[MoP] C-Stem Cemented Stem Duraloc Cementless Cup	300	3.44	2.68	[0.86 , 4.49]	Inferior by ≥100%	0.004
[MoP] C-Stem Cemented Stem Elite Plus Cemented Cup	295	1.05	0.30	[-0.80 , 1.39]	Non-inferiority not shown	0.598
[MoP] C-Stem Cemented Stem Elite Plus Ogee	1,198	1.54	0.79	[0.10 , 1.47]	Non-inferiority not shown	0.024
[MoP] C-Stem Cemented Stem Opera	463	1.66	0.90	[-0.12 , 1.93]	Non-inferiority not shown	0.084
[MoP] C-Stem Cemented Stem Wroblewski Golf Ball	333	2.18	1.42	[0.10 , 2.73]	Non-inferiority not shown	0.034
[MoP] CPS Plus Opera	279	2.13	1.37	[-0.24 , 2.97]	Non-inferiority not shown	0.095
[MoP] CPT Elite Plus Ogee	741	2.31	1.55	[0.65 , 2.45]	Inferior by ≥20%	0.001
[MoP] CPT Trilogy	2,474	2.68	1.92	[1.34 , 2.50]	Inferior by ≥100%	<0.001
[MoP] CPT ZCA	2,612	2.46	1.70	[1.15 , 2.26]	Inferior by ≥100%	<0.001
[MoP] Charnley Cemented Stem Charnley Cemented Cup	1,879	1.93	1.18	[0.52 , 1.83]	Inferior by ≥20%	<0.001

For peer review only

[MoP] Charnley Cemented Stem Charnley Ogee	3,746	1.93	1.18	[0.65 , 1.70]	Inferior by ≥20%	<0.001
[MoP] Charnley Cemented Stem Charnley and Elite Plus LPW	2,920	1.35	0.59	[0.08 , 1.11]	Non-inferiority not shown	0.024
[MoP] Charnley Cemented Stem Opera	496	2.12	1.37	[0.26 , 2.47]	Inferior by ≥20%	0.015
[MoP] Charnley Cemented Stem Wroblewski Golf Ball	404	1.73	0.97	[-0.11 , 2.05]	Non-inferiority not shown	0.079
[MoP] Corail Duraloc Cementless Cup	1,695	3.25	2.49	[1.65 , 3.34]	Inferior by ≥100%	<0.001
[MoP] Corail Elite Plus Cemented Cup	322	1.46	0.70	[-0.39 , 1.79]	Non-inferiority not shown	0.208
[MoP] Corail Elite Plus Ogee	456	1.85	1.09	[0.16 , 2.03]	Inferior by ≥20%	0.022
[MoP] Corail Pinnacle	4,975	2.01	1.25	[0.82 , 1.68]	Inferior by ≥100%	<0.001
[MoP] Corail Trilogy	533	1.87	1.11	[0.14 , 2.08]	Non-inferiority not shown	0.025
[MoP] Elite Plus Cemented Stem Elite Plus Ogee	329	1.26	0.50	[-0.66 , 1.67]	Non-inferiority not shown	0.394
[MoP] Exeter V40 ABG II Cementless Cup	416	1.25	0.49	[-0.57 , 1.55]	Non-inferiority not shown	0.365
[MoP] Exeter V40 Cenator Cemented Cup	860	2.22	1.46	[0.62 , 2.30]	Inferior by ≥20%	0.001
[MoP] Exeter V40 Charnley Ogee	721	1.39	0.64	[-0.14 , 1.41]	Non-inferiority not shown	0.108
[MoP] Exeter V40 Charnley and Elite Plus LPW	788	1.99	1.23	[0.48 , 1.99]	Inferior by ≥20%	0.001
[MoP] Exeter V40 Duraloc Cementless Cup	507	2.12	1.37	[0.24 , 2.49]	Inferior by ≥20%	0.017
[MoP] Exeter V40 EP-Fit Plus	268	3.64	2.88	[0.82 , 4.94]	Inferior by ≥100%	0.006
[MoP] Exeter V40 Elite Plus Ogee	5,848	1.40	0.65	[0.21 , 1.08]	Inferior by ≥20%	0.004
[MoP] Exeter V40 Exeter Contemporary Flanged	11,143	1.36	0.60	[0.20 , 1.00]	Inferior by ≥20%	0.003
[MoP] Exeter V40 Exeter Contemporary Hooded	4,810	2.77	2.01	[1.52 , 2.51]	Inferior by ≥100%	<0.001
[MoP] Exeter V40 Exeter Duration	5,153	2.05	1.29	[0.81 , 1.77]	Inferior by ≥100%	<0.001
[MoP] Exeter V40 Opera	805	1.35	0.59	[-0.10 , 1.29]	Non-inferiority not shown	0.095
[MoP] Exeter V40 Pinnacle	507	1.45	0.69	[0.04 , 1.35]	Non-inferiority not shown	0.038
[MoP] Exeter V40 Reflection Cementless	877	1.73	0.97	[0.14 , 1.80]	Non-inferiority not shown	0.022
[MoP] Exeter V40 Trident	3,625	1.74	0.99	[0.53 , 1.44]	Inferior by ≥20%	<0.001
[MoP] Exeter V40 Trilogy	2,694	1.53	0.77	[0.27 , 1.28]	Inferior by ≥20%	0.003
[MoP] Exeter V40 Ultima Cemented Cup	635	2.04	1.28	[0.22 , 2.33]	Inferior by ≥20%	0.018
[MoP] Furlong Cemented Stem JRI Cemented Cup	730	2.09	1.33	[0.39 , 2.28]	Inferior by ≥20%	0.006
[MoP] Furlong HAC Stem CSF	2,586	3.04	2.28	[1.64 , 2.92]	Inferior by ≥100%	<0.001
[MoP] Furlong HAC Stem Furlong HAC CSF Plus	469	3.16	2.40	[1.53 , 3.27]	Inferior by ≥100%	<0.001
[MoP] Muller Straight Stem Original ME Muller Low Profile Cup	474	2.09	1.33	[0.29 , 2.37]	Inferior by ≥20%	0.012
[MoP] Muller-Biomet Apollo	714	1.85	1.09	[0.23 , 1.95]	Inferior by ≥20%	0.013
[MoP] Omnifit Cemented Stem ODC	495	3.36	2.60	[1.10 , 4.10]	Inferior by ≥100%	0.001
[MoP] SL-Plus Cementless Stem EP-Fit Plus	750	3.80	3.04	[1.92 , 4.16]	Inferior by ≥100%	<0.001
[MoP] SP II Cemented Stem Interplanta	326	2.88	2.12	[0.52 , 3.72]	Inferior by ≥20%	0.009
[MoP] Stanmore Modular Stem SHP Cup	352	1.24	0.48	[-0.50 , 1.46]	Non-inferiority not shown	0.338
[MoP] Stanmore Modular Stem Stanmore-Arcom Cup	1,379	2.01	1.25	[0.59 , 1.90]	Inferior by ≥20%	<0.001
[MoP] Synergy Cementless Stem Reflection Cementless	607	1.33	0.57	[-0.30 , 1.44]	Non-inferiority not shown	0.196
[MoP] Taperloc Cementless Stem Exceed ABT	346	2.99	2.24	[1.34 , 3.13]	Inferior by ≥100%	<0.001
[MoP] Versys Cementless Stem Trilogy	418	4.99	4.23	[2.32 , 6.14]	Inferior by ≥100%	<0.001

For peer review only

Supplemental table 2d: Difference in Kaplan-Meier failure estimate between a contemporary reference and implants with at least 250 at risk at 10 years since primary in females

Stem/cup brand	Number at risk	Cumulative failure (%)	Difference in failure (%)	95% CI	Equivalence status	p-value
[MoP] Exeter V40 Elite Plus Ogee	2,475	1.95	[REFERENCE]			
[CoC] ABG II Monolithic Cementless Stem ABG II Cementless Cup	347	3.34	1.38	[-0.27 , 3.03]	Non-inferiority not shown	0.100
[CoC] Accolade Trident	255	4.07	2.11	[1.17 , 3.05]	Inferior by ≥20%	<0.001
[CoC] Corail Pinnacle	523	3.61	1.65	[1.01 , 2.30]	Inferior by ≥20%	<0.001
[CoC] Exeter V40 ABG II Cementless Cup	302	2.63	0.67	[-0.64 , 1.98]	Non-inferiority not shown	0.317
[CoC] Exeter V40 Trident	1,079	2.45	0.50	[-0.11 , 1.11]	Non-inferiority not shown	0.106
[CoC] Furlong HAC Stem CSF	470	4.44	2.49	[1.01 , 3.97]	Inferior by ≥20%	0.001
[CoP] Exeter V40 Trilogy	300	1.58	-0.37	[-1.28 , 0.53]	Non-inferiority not shown	0.417
[CoP] Furlong HAC Stem CSF	1,467	3.08	1.12	[0.42 , 1.82]	Inferior by ≥20%	0.002
[MoP] C-Stem Cemented Stem Charnley and Elite Plus LPW	534	2.79	0.84	[-0.21 , 1.88]	Non-inferiority not shown	0.117
[MoP] C-Stem Cemented Stem Elite Plus Ogee	570	2.48	0.53	[-0.41 , 1.46]	Non-inferiority not shown	0.271
[MoP] CPT Elite Plus Ogee	256	2.80	0.85	[-0.31 , 2.01]	Non-inferiority not shown	0.151
[MoP] CPT Trilogy	754	4.11	2.16	[1.31 , 3.01]	Inferior by ≥20%	<0.001
[MoP] CPT ZCA	903	3.43	1.48	[0.76 , 2.19]	Inferior by ≥20%	<0.001
[MoP] Charnley Cemented Stem Charnley Cemented Cup	1,136	2.83	0.88	[0.10 , 1.66]	Non-inferiority not shown	0.027
[MoP] Charnley Cemented Stem Charnley Ogee	1,951	3.23	1.28	[0.63 , 1.92]	Inferior by ≥20%	<0.001
[MoP] Charnley Cemented Stem Charnley and Elite Plus LPW	1,623	2.23	0.28	[-0.34 , 0.90]	Non-inferiority not shown	0.376
[MoP] Charnley Cemented Stem Wroblewski Golf Ball	252	2.62	0.67	[-0.81 , 2.14]	Non-inferiority not shown	0.375
[MoP] Corail Duraloc Cementless Cup	783	4.61	2.66	[1.62 , 3.70]	Inferior by ≥20%	<0.001
[MoP] Corail Pinnacle	1,043	2.78	0.83	[0.28 , 1.38]	Non-inferiority not shown	0.003
[MoP] Exeter V40 ABG II Cementless Cup	279	1.55	-0.40	[-1.60 , 0.80]	Non-inferiority not shown	0.515
[MoP] Exeter V40 Cenator Cemented Cup	468	2.50	0.55	[-0.36 , 1.45]	Non-inferiority not shown	0.239
[MoP] Exeter V40 Charnley Ogee	434	1.39	-0.56	[-1.32 , 0.20]	Non-inferior	0.146
[MoP] Exeter V40 Charnley and Elite Plus LPW	292	2.60	0.65	[-0.30 , 1.60]	Non-inferiority not shown	0.180
[MoP] Exeter V40 Duraloc Cementless Cup	301	3.88	1.92	[0.23 , 3.62]	Non-inferiority not shown	0.026
[MoP] Exeter V40 Elite Plus Cemented Cup	480	1.04	-0.91	[-1.55 , -0.28]	Non-inferior	0.005
[MoP] Exeter V40 Exeter Contemporary Flanged	3,136	1.98	0.02	[-0.39 , 0.43]	Non-inferiority not shown	0.919
[MoP] Exeter V40 Exeter Contemporary Hooded	1,595	4.09	2.14	[1.52 , 2.75]	Inferior by ≥20%	<0.001
[MoP] Exeter V40 Exeter Duration	2,079	3.16	1.21	[0.64 , 1.78]	Inferior by ≥20%	<0.001
[MoP] Exeter V40 Opera	315	2.79	0.83	[-0.44 , 2.11]	Non-inferiority not shown	0.200
[MoP] Exeter V40 Trident	714	2.63	0.68	[0.06 , 1.29]	Non-inferiority not shown	0.031
[MoP] Exeter V40 Trilogy	955	2.15	0.20	[-0.40 , 0.80]	Non-inferiority not shown	0.518
[MoP] Exeter V40 Ultima Cemented Cup	334	2.65	0.70	[-0.55 , 1.94]	Non-inferiority not shown	0.275
[MoP] Furlong Cemented Stem JRI Cemented Cup	413	3.49	1.53	[0.20 , 2.87]	Non-inferiority not shown	0.024
[MoP] Furlong HAC Stem CSF	1,088	4.21	2.25	[1.46 , 3.04]	Inferior by ≥20%	<0.001
[MoP] Muller-Biomet Apollo	256	2.18	0.23	[-0.84 , 1.30]	Non-inferiority not shown	0.673
[MoP] Omnifit Cemented Stem ODC	321	4.49	2.54	[0.76 , 4.32]	Inferior by ≥20%	0.005
[MoP] Stanmore Modular Stem Stanmore-Arcom Cup	489	2.78	0.83	[-0.01 , 1.66]	Non-inferiority not shown	0.052
[MoP] Versys Cementless Stem Trilogy	263	5.52	3.57	[1.54 , 5.60]	Inferior by ≥20%	0.001

For peer review only

For peer review only

Supplemental table 3a: Difference in Kaplan-Meier failure estimate between a contemporary reference and implants with at least 250 at risk at 3 years since primary in females <55 years

Stem/cup brand	Number at risk	Cumulative failure (%)	Difference in failure (%)	95% CI	Equivalence status	p-value
[CoC] Exeter V40 Trident	1,785	0.79	[REFERENCE]			
[CoC] Accolade Trident	865	2.12	1.33	[0.33 , 2.33]	Inferior by ≥20%	0.009
[CoC] Corail DeltaMotion	342	1.53	0.74	[-0.54 , 2.02]	Non-inferiority not shown	0.256
[CoC] Corail Pinnacle	4,110	2.15	1.36	[0.81 , 1.92]	Inferior by ≥100%	<0.001
[CoC] Furlong HAC Stem Furlong HAC CSF Plus	969	1.57	0.78	[-0.01 , 1.57]	Non-inferiority not shown	0.052
[CoC] M/L Taper Cementless Continuum	324	1.22	0.43	[-0.70 , 1.57]	Non-inferiority not shown	0.456
[CoC] Taperloc Cementless Stem Exceed ABT	964	1.23	0.44	[-0.27 , 1.16]	Non-inferiority not shown	0.225
[CoP] Accolade Trident	313	1.01	0.22	[-0.67 , 1.12]	Non-inferiority not shown	0.629
[CoP] C-Stem Cemented Stem Marathon	251	1.23	0.44	[-0.71 , 1.58]	Non-inferiority not shown	0.457
[CoP] Corail Pinnacle	720	1.95	1.16	[0.31 , 2.00]	Inferior by ≥20%	0.007
[CoP] Exeter V40 Exeter X3 Rimfit	309	1.29	0.50	[-0.55 , 1.56]	Non-inferiority not shown	0.351
[CoP] Exeter V40 Trident	493	1.06	0.27	[-0.51 , 1.05]	Non-inferiority not shown	0.501
[CoP] Furlong HAC Stem CSF	259	1.81	1.02	[-0.60 , 2.64]	Non-inferiority not shown	0.217
[MoP] Accolade Trident	256	1.87	1.08	[-0.45 , 2.61]	Non-inferiority not shown	0.168
[MoP] Corail Pinnacle	594	1.73	0.94	[-0.05 , 1.93]	Non-inferiority not shown	0.062
[MoP] Exeter V40 Exeter Contemporary Flanged	366	0.56	-0.23	[-0.97 , 0.50]	Non-inferiority not shown	0.535
[MoP] Exeter V40 Trident	344	1.05	0.26	[-0.74 , 1.27]	Non-inferiority not shown	0.610

For peer review only

Supplemental table 3b: Difference in Kaplan-Meier failure estimate between a contemporary reference and implants with at least 250 at risk at 5 years since primary in females <55 years

Stem/cup brand	Number at risk	Cumulative failure (%)	Difference in failure (%)	95% CI	Equivalence status	p-value
[CoC] Exeter V40 Trident	1,426	1.17	[REFERENCE]			
[CoC] Accolade Trident	669	2.64	1.47	[0.31 , 2.62]	Inferior by ≥20%	0.013
[CoC] Corail Pinnacle	2,754	2.93	1.76	[1.07 , 2.45]	Inferior by ≥20%	<0.001
[CoC] Furlong HAC Stem Furlong HAC CSF Plus	610	1.82	0.65	[-0.26 , 1.57]	Non-inferiority not shown	0.161
[CoC] Taperloc Cementless Stem Exceed ABT	557	2.05	0.88	[-0.11 , 1.86]	Non-inferiority not shown	0.080
[CoP] Corail Pinnacle	369	2.59	1.42	[0.33 , 2.52]	Inferior by ≥20%	0.011
[MoP] Corail Pinnacle	365	2.65	1.48	[0.18 , 2.78]	Non-inferiority not shown	0.026
[MoP] Exeter V40 Exeter Contemporary Flanged	279	1.81	0.64	[-0.82 , 2.10]	Non-inferiority not shown	0.391

For peer review only

Supplemental table 3c: Difference in Kaplan-Meier failure estimate between a contemporary reference and implants with at least 250 at risk at 7 years since primary in females <55 years

Stem/cup brand	Number at risk	Cumulative failure (%)	Difference in failure (%)	95% CI	Equivalence status	p-value
[CoC] Corail Pinnacle	1,321	3.60	[REFERENCE]			
[CoC] Accolade Trident	370	3.66	0.06	[-1.38 , 1.51]	Non-inferiority not shown	0.930
[CoC] Exeter V40 Trident	982	2.07	-1.53	[-2.47 , -0.60]	Non-inferior	0.001

For peer review only

Supplemental table 4a: Difference in Kaplan-Meier failure estimate between a contemporary reference and implants with at least 250 at risk at 3 years since primary in females between 55 and 75 years

Stem/cup brand	Number at risk	Cumulative failure (%)	Difference in failure (%)	95% CI	Equivalence status	p-value
[MoP] Exeter V40 Elite Plus Cemented Cup	1,360	0.44	[REFERENCE]			
[CoC] ABG II Monolithic Cementless Stem ABG II Cementless Cup	375	1.57	1.13	[-0.16 , 2.42]	Non-inferiority not shown	0.087
[CoC] ABG II Monolithic Cementless Stem Trident	325	2.05	1.61	[0.07 , 3.14]	Non-inferiority not shown	0.041
[CoC] Accolade Trident	2,547	1.62	1.18	[0.60 , 1.75]	Inferior by ≥100%	<0.001
[CoC] Bimetric Cementless Stem Exceed ABT	407	1.48	1.04	[-0.10 , 2.17]	Non-inferiority not shown	0.074
[CoC] C-Stem AMT Cemented Stem Pinnacle	368	1.00	0.56	[-0.38 , 1.50]	Non-inferiority not shown	0.245
[CoC] CPT Continuum	275	0.90	0.45	[-0.61 , 1.52]	Non-inferiority not shown	0.405
[CoC] Corail Delta TT	293	2.02	1.58	[0.29 , 2.86]	Inferior by ≥20%	0.016
[CoC] Corail DeltaMotion	347	0.95	0.51	[-0.48 , 1.50]	Non-inferiority not shown	0.312
[CoC] Corail Duraloc Option	382	1.52	1.08	[-0.17 , 2.33]	Non-inferiority not shown	0.092
[CoC] Corail Pinnacle	9,567	1.41	0.97	[0.57 , 1.36]	Inferior by ≥100%	<0.001
[CoC] Excia Cementless Plasmacup SC	325	1.03	0.58	[-0.48 , 1.64]	Non-inferiority not shown	0.281
[CoC] Exeter V40 ABG II Cementless Cup	640	0.41	-0.03	[-0.60 , 0.54]	Non-inferiority not shown	0.909
[CoC] Exeter V40 Trident	3,977	0.95	0.50	[0.07 , 0.94]	Non-inferiority not shown	0.024
[CoC] Furlong HAC Stem CSF	620	1.73	1.28	[0.22 , 2.34]	Inferior by ≥20%	0.018
[CoC] Furlong HAC Stem Furlong HAC CSF Plus	3,449	1.59	1.15	[0.66 , 1.63]	Inferior by ≥100%	<0.001
[CoC] M/L Taper Cementless Continuum	363	1.61	1.16	[0.01 , 2.32]	Non-inferiority not shown	0.048
[CoC] Metafix Stem Trinity	290	1.39	0.94	[-0.16 , 2.04]	Non-inferiority not shown	0.093
[CoC] Polarstem Cementless R3 Cementless	370	0.84	0.40	[-0.49 , 1.29]	Non-inferiority not shown	0.377
[CoC] SL-Plus Cementless Stem EP-Fit Plus	376	1.53	1.09	[-0.17 , 2.34]	Non-inferiority not shown	0.091
[CoC] Taperloc Cementless Stem Exceed ABT	2,580	1.46	1.01	[0.50 , 1.53]	Inferior by ≥100%	<0.001
[CoP] Accolade Trident	1,324	1.44	0.99	[0.36 , 1.63]	Inferior by ≥20%	0.002
[CoP] C-Stem Cemented Stem Elite Plus Ogee	279	0.35	-0.09	[-0.85 , 0.66]	Non-inferiority not shown	0.807
[CoP] C-Stem Cemented Stem Marathon	364	0.59	0.15	[-0.62 , 0.91]	Non-inferiority not shown	0.706
[CoP] C-Stem Cemented Stem Opera	295	0.00	- -	[-.-- , -.--]	No failures to date	
[CoP] C-Stem Cemented Stem Wroblewski Golf Ball	346	0.53	0.08	[-0.72 , 0.89]	Non-inferiority not shown	0.836
[CoP] CPT Trilogy	573	1.20	0.75	[0.13 , 1.38]	Inferior by ≥20%	0.018
[CoP] Corail Charnley and Elite Plus LPW	296	2.41	1.97	[0.28 , 3.65]	Inferior by ≥20%	0.022
[CoP] Corail Marathon	559	0.73	0.29	[-0.35 , 0.93]	Non-inferiority not shown	0.378
[CoP] Corail Pinnacle	3,489	1.19	0.75	[0.33 , 1.17]	Inferior by ≥20%	<0.001
[CoP] Corail Trilogy	355	0.81	0.37	[-0.60 , 1.34]	Non-inferiority not shown	0.460
[CoP] Exeter V40 Charnley and Elite Plus LPW	355	1.69	1.24	[0.03 , 2.45]	Non-inferiority not shown	0.044
[CoP] Exeter V40 Elite Plus Ogee	529	0.70	0.26	[-0.44 , 0.95]	Non-inferiority not shown	0.468
[CoP] Exeter V40 Exeter Contemporary Flanged	1,464	1.06	0.62	[0.05 , 1.18]	Non-inferiority not shown	0.032
[CoP] Exeter V40 Exeter Contemporary Hooded	376	1.55	1.11	[-0.08 , 2.30]	Non-inferiority not shown	0.068
[CoP] Exeter V40 Exeter Duration	369	0.98	0.54	[-0.48 , 1.55]	Non-inferiority not shown	0.299
[CoP] Exeter V40 Exeter X3 Rimfit	896	0.66	0.22	[-0.27 , 0.70]	Non-inferiority not shown	0.385
[CoP] Exeter V40 Trident	2,023	1.02	0.57	[0.12 , 1.03]	Inferior by ≥20%	0.014
[CoP] Exeter V40 Trilogy	896	0.61	0.16	[-0.42 , 0.75]	Non-inferiority not shown	0.587
[CoP] Furlong HAC Stem CSF	2,797	1.29	0.85	[0.33 , 1.37]	Inferior by ≥20%	0.001

For peer review only

[CoP] Furlong HAC Stem Furlong HAC CSF Plus	660	2.00	1.56	[0.58 , 2.54]	Inferior by ≥100%	0.002
[CoP] MS-30 Original ME Muller Low Profile Cup	785	0.57	0.13	[-0.47 , 0.73]	Non-inferiority not shown	0.674
[CoP] SL-Plus Cementless Stem EP-Fit Plus	368	1.82	1.38	[-0.00 , 2.75]	Non-inferiority not shown	0.050
[CoP] Taperloc Cementless Stem Exceed ABT	789	1.04	0.60	[-0.03 , 1.22]	Non-inferiority not shown	0.062
[MoP] Accolade Trident	3,609	1.81	1.36	[0.84 , 1.89]	Inferior by ≥100%	<0.001
[MoP] Anthology R3 Cementless	506	1.00	0.56	[-0.18 , 1.29]	Non-inferiority not shown	0.138
[MoP] C-Stem AMT Cemented Stem Charnley and Elite Plus LPW	779	0.76	0.32	[-0.34 , 0.97]	Non-inferiority not shown	0.341
[MoP] C-Stem AMT Cemented Stem Elite Plus Ogee	482	0.79	0.35	[-0.43 , 1.12]	Non-inferiority not shown	0.380
[MoP] C-Stem AMT Cemented Stem Marathon	406	1.09	0.65	[-0.12 , 1.42]	Non-inferiority not shown	0.099
[MoP] C-Stem AMT Cemented Stem Pinnacle	609	1.05	0.60	[-0.07 , 1.27]	Non-inferiority not shown	0.077
[MoP] C-Stem Cemented Stem Charnley Ogee	400	1.80	1.35	[0.07 , 2.64]	Non-inferiority not shown	0.038
[MoP] C-Stem Cemented Stem Charnley and Elite Plus LPW	727	1.04	0.60	[-0.19 , 1.39]	Non-inferiority not shown	0.138
[MoP] C-Stem Cemented Stem Duraloc Cementless Cup	298	1.64	1.20	[-0.27 , 2.67]	Non-inferiority not shown	0.109
[MoP] C-Stem Cemented Stem Elite Plus Ogee	1,130	0.91	0.47	[-0.14 , 1.08]	Non-inferiority not shown	0.132
[MoP] C-Stem Cemented Stem Marathon	616	0.67	0.22	[-0.37 , 0.82]	Non-inferiority not shown	0.461
[MoP] C-Stem Cemented Stem Opera	541	0.71	0.26	[-0.50 , 1.03]	Non-inferiority not shown	0.500
[MoP] C-Stem Cemented Stem Wroblewski Golf Ball	397	0.48	0.04	[-0.71 , 0.78]	Non-inferiority not shown	0.925
[MoP] CCA Cemented Stem CCB Cup	457	0.34	-0.10	[-0.68 , 0.47]	Non-inferiority not shown	0.728
[MoP] CLS Cementless Stem Allofit	259	1.45	1.01	[-0.44 , 2.46]	Non-inferiority not shown	0.174
[MoP] CPCS Opera	488	1.19	0.75	[-0.26 , 1.75]	Non-inferiority not shown	0.145
[MoP] CPT Allofit	386	0.16	-0.28	[-0.74 , 0.17]	Non-inferiority not shown	0.220
[MoP] CPT Elite Plus Ogee	922	1.28	0.84	[0.07 , 1.61]	Non-inferiority not shown	0.032
[MoP] CPT Original ME Muller Low Profile Cup	324	0.92	0.48	[-0.48 , 1.44]	Non-inferiority not shown	0.329
[MoP] CPT Pinnacle	290	0.64	0.20	[-0.75 , 1.15]	Non-inferiority not shown	0.680
[MoP] CPT Trabecular Metal Modular Cementless Cup	317	2.73	2.29	[0.72 , 3.86]	Inferior by ≥100%	0.004
[MoP] CPT Trilogy	3,394	1.23	0.79	[0.32 , 1.26]	Inferior by ≥20%	0.001
[MoP] CPT ZCA	2,305	1.24	0.79	[0.28 , 1.31]	Inferior by ≥20%	0.003
[MoP] Charnley Cemented Stem Charnley Cemented Cup	1,559	0.98	0.54	[-0.04 , 1.12]	Non-inferiority not shown	0.069
[MoP] Charnley Cemented Stem Charnley Ogee	3,266	1.18	0.73	[0.25 , 1.22]	Inferior by ≥20%	0.003
[MoP] Charnley Cemented Stem Charnley and Elite Plus LPW	2,255	0.68	0.23	[-0.24 , 0.70]	Non-inferiority not shown	0.331
[MoP] Charnley Cemented Stem Opera	498	0.39	-0.05	[-0.69 , 0.58]	Non-inferiority not shown	0.868
[MoP] Charnley Cemented Stem Wroblewski Golf Ball	333	1.43	0.98	[-0.30 , 2.27]	Non-inferiority not shown	0.134
[MoP] Corail Charnley and Elite Plus LPW	334	0.23	-0.21	[-0.77 , 0.35]	Non-inferiority not shown	0.454
[MoP] Corail Duraloc Cementless Cup	1,523	1.59	1.14	[0.44 , 1.84]	Inferior by ≥20%	0.001
[MoP] Corail Elite Plus Cemented Cup	354	0.25	-0.20	[-0.78 , 0.39]	Non-inferiority not shown	0.510
[MoP] Corail Elite Plus Ogee	546	1.03	0.58	[-0.24 , 1.41]	Non-inferiority not shown	0.167
[MoP] Corail Marathon	1,156	0.74	0.30	[-0.21 , 0.80]	Non-inferiority not shown	0.246
[MoP] Corail Pinnacle	11,626	1.15	0.71	[0.34 , 1.08]	Inferior by ≥20%	<0.001
[MoP] Corail Trilogy	680	0.78	0.34	[-0.37 , 1.04]	Non-inferiority not shown	0.350
[MoP] Exeter V40 ABG II Cementless Cup	288	1.01	0.56	[-0.62 , 1.74]	Non-inferiority not shown	0.351
[MoP] Exeter V40 Cenator Cemented Cup	721	1.68	1.24	[0.27 , 2.20]	Inferior by ≥20%	0.012
[MoP] Exeter V40 Charnley Ogee	658	1.07	0.63	[-0.18 , 1.44]	Non-inferiority not shown	0.129
[MoP] Exeter V40 Charnley and Elite Plus LPW	943	1.27	0.82	[0.11 , 1.54]	Inferior by ≥20%	0.025
[MoP] Exeter V40 Duraloc Cementless Cup	509	0.77	0.33	[-0.49 , 1.15]	Non-inferiority not shown	0.437
[MoP] Exeter V40 EP-Fit Plus	265	1.11	0.67	[-0.63 , 1.96]	Non-inferiority not shown	0.312
[MoP] Exeter V40 Elite Plus Ogee	5,613	0.77	0.33	[-0.07 , 0.72]	Non-inferiority not shown	0.103

For peer review only

[MoP] Exeter V40 Exeter Contemporary Flanged	14,698	0.81	0.36	[0.01 , 0.72]	Non-inferiority not shown	0.043
[MoP] Exeter V40 Exeter Contemporary Hooded	5,787	1.59	1.14	[0.70 , 1.58]	Inferior by ≥100%	<0.001
[MoP] Exeter V40 Exeter Duration	4,914	1.14	0.70	[0.26 , 1.13]	Inferior by ≥20%	0.002
[MoP] Exeter V40 Exeter X3 Rimfit	1,966	0.71	0.27	[-0.15 , 0.68]	Non-inferiority not shown	0.210
[MoP] Exeter V40 Marathon	486	0.87	0.42	[-0.31 , 1.16]	Non-inferiority not shown	0.261
[MoP] Exeter V40 Opera	876	0.86	0.41	[-0.26 , 1.09]	Non-inferiority not shown	0.232
[MoP] Exeter V40 Pinnacle	1,011	1.06	0.61	[-0.02 , 1.25]	Non-inferiority not shown	0.058
[MoP] Exeter V40 Reflection Cementless	785	0.74	0.30	[-0.38 , 0.97]	Non-inferiority not shown	0.389
[MoP] Exeter V40 Trident	6,447	1.11	0.67	[0.28 , 1.06]	Inferior by ≥20%	0.001
[MoP] Exeter V40 Trilogy	3,453	0.79	0.34	[-0.09 , 0.77]	Non-inferiority not shown	0.119
[MoP] Exeter V40 Ultima Cemented Cup	425	1.38	0.93	[-0.21 , 2.08]	Non-inferiority not shown	0.109
[MoP] Furlong Cemented Stem JRI Cemented Cup	495	1.90	1.46	[0.24 , 2.67]	Inferior by ≥20%	0.019
[MoP] Furlong HAC Stem CSF	2,428	1.91	1.47	[0.85 , 2.08]	Inferior by ≥100%	<0.001
[MoP] Furlong HAC Stem Furlong HAC CSF Plus	1,020	1.79	1.35	[0.60 , 2.10]	Inferior by ≥100%	<0.001
[MoP] M/L Taper Cementless Continuum	253	1.51	1.07	[-0.14 , 2.28]	Non-inferiority not shown	0.084
[MoP] Muller Straight Stem Original ME Muller Low Profile Cup	530	1.06	0.62	[-0.24 , 1.47]	Non-inferiority not shown	0.157
[MoP] Muller-Biomet Apollo	634	1.93	1.49	[0.40 , 2.58]	Inferior by ≥20%	0.008
[MoP] Muller-Biomet Original ME Muller Low Profile Cup	362	1.57	1.12	[-0.16 , 2.41]	Non-inferiority not shown	0.087
[MoP] Omnifit Cemented Stem ODC	358	1.63	1.19	[-0.15 , 2.53]	Non-inferiority not shown	0.081
[MoP] Polarstem Cementless R3 Cementless	355	0.64	0.20	[-0.35 , 0.75]	Non-inferiority not shown	0.479
[MoP] SL-Plus Cementless Stem EP-Fit Plus	857	3.12	2.68	[1.56 , 3.79]	Inferior by ≥100%	<0.001
[MoP] SP II Cemented Stem Interplanta	258	2.11	1.67	[-0.04 , 3.37]	Non-inferiority not shown	0.055
[MoP] Stanmore Modular Stem SHP Cup	298	0.89	0.44	[-0.61 , 1.49]	Non-inferiority not shown	0.411
[MoP] Stanmore Modular Stem Stanmore-Arcom Cup	1,365	1.33	0.89	[0.22 , 1.56]	Inferior by ≥20%	0.009
[MoP] Synergy Cementless Stem R3 Cementless	363	0.63	0.19	[-0.46 , 0.83]	Non-inferiority not shown	0.571
[MoP] Synergy Cementless Stem Reflection Cementless	598	0.81	0.37	[-0.41 , 1.15]	Non-inferiority not shown	0.357
[MoP] Taperloc Cementless Stem Exceed ABT	1,475	2.08	1.63	[0.94 , 2.33]	Inferior by ≥100%	<0.001
[MoP] Versys Cementless Stem Trilogy	363	3.38	2.94	[1.10 , 4.78]	Inferior by ≥100%	0.002

Supplemental table 4b: Difference in Kaplan-Meier failure estimate between a contemporary reference and implants with at least 250 at risk at 5 years since primary in females between 55 and 75 years

Stem/cup brand	Number at risk	Cumulative failure (%)	Difference in failure (%)	95% CI	Equivalence status	p-value
[MoP] Exeter V40 Elite Plus Cemented Cup	1,039	0.52	[REFERENCE]			
[CoC] ABG II Monolithic Cementless Stem ABG II Cementless Cup	363	1.84	1.32	[-0.08 , 2.71]	Non-inferiority not shown	0.064
[CoC] ABG II Monolithic Cementless Stem Trident	250	3.09	2.57	[0.64 , 4.49]	Inferior by ≥100%	0.009
[CoC] Accolade Trident	2,044	2.56	2.04	[1.33 , 2.75]	Inferior by ≥100%	<0.001
[CoC] Bimetric Cementless Stem Exceed ABT	289	2.32	1.80	[0.32 , 3.28]	Inferior by ≥20%	0.017
[CoC] Corail Duraloc Option	370	2.82	2.30	[0.62 , 3.99]	Inferior by ≥100%	0.007
[CoC] Corail Pinnacle	6,445	1.82	1.30	[0.86 , 1.74]	Inferior by ≥100%	<0.001

For peer review only

[CoC] Exeter V40 ABG II Cementless Cup	563	1.05	0.53	[-0.33 , 1.38]	Non-inferiority not shown	0.225
[CoC] Exeter V40 Trident	3,263	1.29	0.77	[0.27 , 1.26]	Inferior by ≥20%	0.002
[CoC] Furlong HAC Stem CSF	597	2.05	1.53	[0.37 , 2.68]	Inferior by ≥20%	0.010
[CoC] Furlong HAC Stem Furlong HAC CSF Plus	2,167	1.86	1.34	[0.80 , 1.88]	Inferior by ≥100%	<0.001
[CoC] SL-Plus Cementless Stem EP-Fit Plus	352	3.15	2.63	[0.84 , 4.43]	Inferior by ≥100%	0.004
[CoC] Taperloc Cementless Stem Exceed ABT	1,583	1.68	1.16	[0.59 , 1.72]	Inferior by ≥100%	<0.001
[CoP] Accolade Trident	638	1.84	1.32	[0.56 , 2.08]	Inferior by ≥100%	0.001
[CoP] C-Stem Cemented Stem Wroblewski Golf Ball	283	0.82	0.30	[-0.69 , 1.30]	Non-inferiority not shown	0.549
[CoP] Corail Pinnacle	1,874	1.58	1.06	[0.56 , 1.57]	Inferior by ≥100%	<0.001
[CoP] Corail Trilogy	327	1.09	0.57	[-0.55 , 1.70]	Non-inferiority not shown	0.317
[CoP] Exeter V40 Elite Plus Ogee	397	1.31	0.79	[-0.20 , 1.77]	Non-inferiority not shown	0.117
[CoP] Exeter V40 Exeter Contemporary Flanged	964	1.38	0.86	[0.20 , 1.52]	Inferior by ≥20%	0.011
[CoP] Exeter V40 Exeter Contemporary Hooded	290	1.82	1.30	[-0.01 , 2.60]	Non-inferiority not shown	0.051
[CoP] Exeter V40 Exeter Duration	325	0.98	0.46	[-0.56 , 1.49]	Non-inferiority not shown	0.377
[CoP] Exeter V40 Trident	999	1.37	0.86	[0.28 , 1.43]	Inferior by ≥20%	0.003
[CoP] Exeter V40 Trilogy	748	0.72	0.20	[-0.44 , 0.84]	Non-inferiority not shown	0.540
[CoP] Furlong HAC Stem CSF	2,437	1.82	1.30	[0.69 , 1.90]	Inferior by ≥100%	<0.001
[CoP] Furlong HAC Stem Furlong HAC CSF Plus	397	2.25	1.73	[0.63 , 2.83]	Inferior by ≥100%	0.002
[CoP] MS-30 Original ME Muller Low Profile Cup	576	0.72	0.21	[-0.48 , 0.89]	Non-inferiority not shown	0.557
[CoP] SL-Plus Cementless Stem EP-Fit Plus	317	3.48	2.96	[1.07 , 4.86]	Inferior by ≥100%	0.002
[CoP] Taperloc Cementless Stem Exceed ABT	355	1.36	0.84	[0.06 , 1.61]	Non-inferiority not shown	0.035
[MoP] Accolade Trident	2,455	2.47	1.95	[1.34 , 2.56]	Inferior by ≥100%	<0.001
[MoP] C-Stem AMT Cemented Stem Charnley and Elite Plus LPW	556	1.17	0.65	[-0.16 , 1.46]	Non-inferiority not shown	0.117
[MoP] C-Stem AMT Cemented Stem Elite Plus Ogee	319	1.54	1.02	[-0.14 , 2.18]	Non-inferiority not shown	0.084
[MoP] C-Stem AMT Cemented Stem Pinnacle	308	1.68	1.16	[0.17 , 2.14]	Inferior by ≥20%	0.021
[MoP] C-Stem Cemented Stem Charnley Ogee	335	2.64	2.12	[0.53 , 3.71]	Inferior by ≥100%	0.009
[MoP] C-Stem Cemented Stem Charnley and Elite Plus LPW	671	1.88	1.36	[0.32 , 2.41]	Inferior by ≥20%	0.010
[MoP] C-Stem Cemented Stem Duraloc Cementless Cup	285	2.32	1.80	[0.06 , 3.54]	Non-inferiority not shown	0.042
[MoP] C-Stem Cemented Stem Elite Plus Ogee	906	1.40	0.88	[0.12 , 1.63]	Inferior by ≥20%	0.023
[MoP] C-Stem Cemented Stem Marathon	330	0.67	0.15	[-0.46 , 0.76]	Non-inferiority not shown	0.633
[MoP] C-Stem Cemented Stem Opera	407	0.90	0.38	[-0.48 , 1.24]	Non-inferiority not shown	0.390
[MoP] C-Stem Cemented Stem Wroblewski Golf Ball	324	1.57	1.05	[-0.25 , 2.36]	Non-inferiority not shown	0.113
[MoP] CCA Cemented Stem CCB Cup	327	0.61	0.09	[-0.70 , 0.88]	Non-inferiority not shown	0.827
[MoP] CPCS Opera	367	2.09	1.57	[0.24 , 2.91]	Inferior by ≥20%	0.021
[MoP] CPT Elite Plus Ogee	691	1.65	1.13	[0.25 , 2.01]	Inferior by ≥20%	0.012
[MoP] CPT Pinnacle	254	1.40	0.88	[-0.53 , 2.29]	Non-inferiority not shown	0.223
[MoP] CPT Trilogy	2,468	2.22	1.70	[1.10 , 2.30]	Inferior by ≥100%	<0.001
[MoP] CPT ZCA	1,787	2.02	1.50	[0.84 , 2.15]	Inferior by ≥100%	<0.001
[MoP] Charnley Cemented Stem Charnley Cemented Cup	1,420	1.52	1.00	[0.30 , 1.70]	Inferior by ≥20%	0.005
[MoP] Charnley Cemented Stem Charnley Ogee	2,959	1.84	1.32	[0.74 , 1.90]	Inferior by ≥100%	<0.001
[MoP] Charnley Cemented Stem Charnley and Elite Plus LPW	2,049	1.18	0.67	[0.09 , 1.24]	Non-inferiority not shown	0.023
[MoP] Charnley Cemented Stem Opera	454	1.22	0.70	[-0.34 , 1.73]	Non-inferiority not shown	0.185
[MoP] Charnley Cemented Stem Wroblewski Golf Ball	277	1.77	1.26	[-0.20 , 2.71]	Non-inferiority not shown	0.091
[MoP] Corail Duraloc Cementless Cup	1,465	2.31	1.79	[0.96 , 2.62]	Inferior by ≥100%	<0.001
[MoP] Corail Elite Plus Cemented Cup	277	0.55	0.03	[-0.81 , 0.87]	Non-inferiority not shown	0.947
[MoP] Corail Elite Plus Ogee	418	1.62	1.10	[0.03 , 2.17]	Non-inferiority not shown	0.044

For peer review only

[MoP] Corail Marathon	586	0.96	0.44	[-0.16 , 1.05]	Non-inferiority not shown	0.153
[MoP] Corail Pinnacle	7,089	1.41	0.89	[0.49 , 1.30]	Inferior by ≥20%	<0.001
[MoP] Corail Trilogy	561	0.94	0.42	[-0.36 , 1.20]	Non-inferiority not shown	0.293
[MoP] Exeter V40 ABG II Cementless Cup	283	1.01	0.49	[-0.70 , 1.67]	Non-inferiority not shown	0.422
[MoP] Exeter V40 Cenator Cemented Cup	611	2.67	2.15	[0.94 , 3.37]	Inferior by ≥100%	<0.001
[MoP] Exeter V40 Charnley Ogee	568	1.39	0.87	[-0.06 , 1.79]	Non-inferiority not shown	0.068
[MoP] Exeter V40 Charnley and Elite Plus LPW	718	1.72	1.20	[0.34 , 2.05]	Inferior by ≥20%	0.006
[MoP] Exeter V40 Duraloc Cementless Cup	488	1.37	0.85	[-0.22 , 1.93]	Non-inferiority not shown	0.119
[MoP] Exeter V40 EP-Fit Plus	250	3.01	2.49	[0.40 , 4.57]	Inferior by ≥20%	0.019
[MoP] Exeter V40 Elite Plus Ogee	4,543	1.18	0.66	[0.21 , 1.12]	Inferior by ≥20%	0.004
[MoP] Exeter V40 Exeter Contemporary Flanged	10,220	1.16	0.64	[0.25 , 1.04]	Inferior by ≥20%	0.001
[MoP] Exeter V40 Exeter Contemporary Hooded	4,277	2.45	1.93	[1.40 , 2.46]	Inferior by ≥100%	<0.001
[MoP] Exeter V40 Exeter Duration	4,048	1.48	0.96	[0.47 , 1.44]	Inferior by ≥20%	<0.001
[MoP] Exeter V40 Exeter X3 Rimfit	319	0.71	0.19	[-0.25 , 0.63]	Non-inferiority not shown	0.395
[MoP] Exeter V40 Opera	655	1.22	0.70	[-0.10 , 1.50]	Non-inferiority not shown	0.088
[MoP] Exeter V40 Pinnacle	658	1.40	0.88	[0.12 , 1.64]	Inferior by ≥20%	0.023
[MoP] Exeter V40 Reflection Cementless	732	1.14	0.62	[-0.20 , 1.44]	Non-inferiority not shown	0.141
[MoP] Exeter V40 Trident	4,016	1.44	0.92	[0.47 , 1.36]	Inferior by ≥20%	<0.001
[MoP] Exeter V40 Trilogy	2,821	1.20	0.68	[0.17 , 1.18]	Inferior by ≥20%	0.009
[MoP] Exeter V40 Ultima Cemented Cup	408	2.56	2.04	[0.51 , 3.58]	Inferior by ≥20%	0.009
[MoP] Furlong Cemented Stem JRI Cemented Cup	458	2.52	2.00	[0.60 , 3.41]	Inferior by ≥100%	0.005
[MoP] Furlong HAC Stem CSF	2,056	2.08	1.56	[0.91 , 2.22]	Inferior by ≥100%	<0.001
[MoP] Furlong HAC Stem Furlong HAC CSF Plus	633	2.82	2.30	[1.26 , 3.34]	Inferior by ≥100%	<0.001
[MoP] Muller Straight Stem Original ME Muller Low Profile Cup	395	1.47	0.95	[-0.08 , 1.98]	Non-inferiority not shown	0.071
[MoP] Muller-Biomet Apollo	535	2.11	1.59	[0.44 , 2.74]	Inferior by ≥20%	0.007
[MoP] Omnifit Cemented Stem ODC	340	2.48	1.96	[0.32 , 3.60]	Inferior by ≥20%	0.019
[MoP] SL-Plus Cementless Stem EP-Fit Plus	739	3.85	3.33	[2.07 , 4.59]	Inferior by ≥100%	<0.001
[MoP] Stanmore Modular Stem Stanmore-Arcom Cup	1,106	2.20	1.68	[0.83 , 2.53]	Inferior by ≥100%	<0.001
[MoP] Synergy Cementless Stem Reflection Cementless	577	1.15	0.63	[-0.29 , 1.55]	Non-inferiority not shown	0.179
[MoP] Taperloc Cementless Stem Exceed ABT	761	2.49	1.97	[1.18 , 2.77]	Inferior by ≥100%	<0.001
[MoP] Versys Cementless Stem Trilogy	342	4.74	4.22	[2.05 , 6.38]	Inferior by ≥100%	<0.001

For peer review only

Supplemental table 4c: Difference in Kaplan-Meier failure estimate between a contemporary reference and implants with at least 250 at risk at 7 years since primary in females between 55 and 75 years

Stem/cup brand	Number at risk	Cumulative failure (%)	Difference in failure (%)	95% CI	Equivalence status	p-value
[MoP] Exeter V40 Trilogy	1,962	1.45	[REFERENCE]			
[CoC] ABG II Monolithic Cementless Stem ABG II Cementless Cup	335	2.11	0.66	[-0.84 , 2.16]	Non-inferiority not shown	0.389
[CoC] Accolade Trident	1,256	3.07	1.62	[0.81 , 2.44]	Inferior by ≥20%	<0.001
[CoC] Corail Duraloc Option	301	3.15	1.70	[-0.11 , 3.50]	Non-inferiority not shown	0.065
[CoC] Corail Pinnacle	2,917	2.20	0.76	[0.25 , 1.26]	Non-inferiority not shown	0.004
[CoC] Exeter V40 ABG II Cementless Cup	454	1.65	0.20	[-0.90 , 1.31]	Non-inferiority not shown	0.721
[CoC] Exeter V40 Trident	2,340	1.76	0.31	[-0.28 , 0.90]	Non-inferiority not shown	0.300
[CoC] Furlong HAC Stem CSF	549	2.90	1.45	[0.07 , 2.83]	Non-inferiority not shown	0.040
[CoC] Furlong HAC Stem Furlong HAC CSF Plus	830	2.23	0.78	[0.12 , 1.44]	Non-inferiority not shown	0.021
[CoC] SL-Plus Cementless Stem EP-Fit Plus	295	3.46	2.01	[0.11 , 3.90]	Non-inferiority not shown	0.038
[CoC] Taperloc Cementless Stem Exceed ABT	712	1.81	0.36	[-0.29 , 1.01]	Non-inferiority not shown	0.279
[CoP] Accolade Trident	257	2.02	0.57	[-0.29 , 1.43]	Non-inferiority not shown	0.195
[CoP] Corail Pinnacle	771	1.86	0.41	[-0.20 , 1.02]	Non-inferiority not shown	0.183
[CoP] Corail Trilogy	258	1.09	-0.35	[-1.50 , 0.79]	Non-inferiority not shown	0.543
[CoP] Exeter V40 Elite Plus Ogee	251	1.59	0.15	[-1.00 , 1.29]	Non-inferiority not shown	0.804
[CoP] Exeter V40 Exeter Contemporary Flanged	563	1.38	-0.07	[-0.76 , 0.61]	Non-inferiority not shown	0.839
[CoP] Exeter V40 Exeter Duration	255	1.30	-0.14	[-1.36 , 1.07]	Non-inferiority not shown	0.818
[CoP] Exeter V40 Trident	463	1.63	0.19	[-0.52 , 0.89]	Non-inferiority not shown	0.606
[CoP] Exeter V40 Trilogy	561	1.02	-0.43	[-1.22 , 0.35]	Non-inferiority not shown	0.282
[CoP] Furlong HAC Stem CSF	2,033	2.34	0.90	[0.20 , 1.60]	Non-inferiority not shown	0.012
[CoP] MS-30 Original ME Muller Low Profile Cup	374	0.72	-0.72	[-1.43 , -0.01]	Non-inferior	0.046
[MoP] Accolade Trident	1,158	3.23	1.79	[1.03 , 2.54]	Inferior by ≥20%	<0.001
[MoP] C-Stem AMT Cemented Stem Charnley and Elite Plus LPW	344	1.72	0.28	[-0.85 , 1.41]	Non-inferiority not shown	0.632
[MoP] C-Stem Cemented Stem Charnley Ogee	284	2.97	1.52	[-0.20 , 3.24]	Non-inferiority not shown	0.084
[MoP] C-Stem Cemented Stem Charnley and Elite Plus LPW	617	2.66	1.21	[-0.04 , 2.46]	Non-inferiority not shown	0.058
[MoP] C-Stem Cemented Stem Elite Plus Ogee	733	2.13	0.68	[-0.29 , 1.65]	Non-inferiority not shown	0.170
[MoP] C-Stem Cemented Stem Opera	311	1.69	0.24	[-1.01 , 1.49]	Non-inferiority not shown	0.709
[MoP] CPT Elite Plus Ogee	470	2.58	1.13	[-0.08 , 2.34]	Non-inferiority not shown	0.067
[MoP] CPT Trilogy	1,661	2.95	1.50	[0.77 , 2.23]	Inferior by ≥20%	<0.001
[MoP] CPT ZCA	1,254	2.39	0.94	[0.20 , 1.68]	Non-inferiority not shown	0.013
[MoP] Charnley Cemented Stem Charnley Cemented Cup	1,220	2.10	0.65	[-0.18 , 1.48]	Non-inferiority not shown	0.125
[MoP] Charnley Cemented Stem Charnley Ogee	2,422	2.20	0.76	[0.11 , 1.40]	Non-inferiority not shown	0.022
[MoP] Charnley Cemented Stem Charnley and Elite Plus LPW	1,746	1.64	0.20	[-0.48 , 0.87]	Non-inferiority not shown	0.569
[MoP] Charnley Cemented Stem Opera	345	2.16	0.71	[-0.68 , 2.10]	Non-inferiority not shown	0.316
[MoP] Corail Duraloc Cementless Cup	1,258	3.66	2.21	[1.18 , 3.25]	Inferior by ≥20%	<0.001
[MoP] Corail Elite Plus Ogee	292	1.93	0.48	[-0.76 , 1.72]	Non-inferiority not shown	0.449
[MoP] Corail Pinnacle	3,659	1.82	0.38	[-0.10 , 0.86]	Non-inferiority not shown	0.123
[MoP] Corail Trilogy	369	1.65	0.20	[-0.93 , 1.34]	Non-inferiority not shown	0.725
[MoP] Exeter V40 ABG II Cementless Cup	272	1.01	-0.44	[-1.65 , 0.76]	Non-inferiority not shown	0.472

For peer review only

[MoP] Exeter V40 Cenator Cemented Cup	478	2.86	1.41	[0.13 , 2.69]	Non-inferiority not shown	0.030
[MoP] Exeter V40 Charnley Ogee	467	1.39	-0.06	[-1.01 , 0.89]	Non-inferiority not shown	0.897
[MoP] Exeter V40 Charnley and Elite Plus LPW	470	2.39	0.94	[-0.15 , 2.03]	Non-inferiority not shown	0.091
[MoP] Exeter V40 Duraloc Cementless Cup	392	1.79	0.34	[-0.89 , 1.56]	Non-inferiority not shown	0.589
[MoP] Exeter V40 Elite Plus Cemented Cup	701	0.63	-0.82	[-1.40 , -0.23]	Non-inferior	0.006
[MoP] Exeter V40 Elite Plus Ogee	3,436	1.59	0.14	[-0.38 , 0.67]	Non-inferiority not shown	0.593
[MoP] Exeter V40 Exeter Contemporary Flanged	6,755	1.51	0.06	[-0.39 , 0.52]	Non-inferiority not shown	0.788
[MoP] Exeter V40 Exeter Contemporary Hooded	2,891	3.20	1.75	[1.12 , 2.38]	Inferior by ≥20%	<0.001
[MoP] Exeter V40 Exeter Duration	3,049	2.17	0.72	[0.14 , 1.31]	Non-inferiority not shown	0.016
[MoP] Exeter V40 Opera	462	1.54	0.09	[-0.84 , 1.02]	Non-inferiority not shown	0.850
[MoP] Exeter V40 Pinnacle	346	1.56	0.11	[-0.73 , 0.95]	Non-inferiority not shown	0.797
[MoP] Exeter V40 Reflection Cementless	591	2.01	0.56	[-0.53 , 1.65]	Non-inferiority not shown	0.312
[MoP] Exeter V40 Trident	2,291	2.01	0.56	[0.01 , 1.11]	Non-inferiority not shown	0.046
[MoP] Exeter V40 Ultima Cemented Cup	379	2.80	1.36	[-0.26 , 2.97]	Non-inferiority not shown	0.100
[MoP] Furlong Cemented Stem JRI Cemented Cup	416	2.97	1.52	[-0.02 , 3.05]	Non-inferiority not shown	0.053
[MoP] Furlong HAC Stem CSF	1,634	2.65	1.20	[0.44 , 1.96]	Inferior by ≥20%	0.002
[MoP] Furlong HAC Stem Furlong HAC CSF Plus	270	3.32	1.87	[0.67 , 3.06]	Inferior by ≥20%	0.002
[MoP] Muller Straight Stem Original ME Muller Low Profile Cup	264	2.38	0.93	[-0.53 , 2.40]	Non-inferiority not shown	0.211
[MoP] Muller-Biomet Apollo	414	2.72	1.27	[-0.08 , 2.62]	Non-inferiority not shown	0.065
[MoP] Omnifit Cemented Stem ODC	309	3.99	2.54	[0.45 , 4.63]	Inferior by ≥20%	0.017
[MoP] SL-Plus Cementless Stem EP-Fit Plus	586	4.42	2.97	[1.59 , 4.36]	Inferior by ≥100%	<0.001
[MoP] Stanmore Modular Stem Stanmore-Arcom Cup	784	2.59	1.14	[0.19 , 2.09]	Non-inferiority not shown	0.018
[MoP] Synergy Cementless Stem Reflection Cementless	432	1.74	0.29	[-0.86 , 1.44]	Non-inferiority not shown	0.617
[MoP] Versys Cementless Stem Trilogy	318	4.74	3.29	[1.11 , 5.46]	Inferior by ≥20%	0.003

For peer review only

Supplemental table 4d: Difference in Kaplan-Meier failure estimate between a contemporary reference and implants with at least 250 at risk at 10 years since primary in females between 55 and 75 years

Stem/cup brand	Number at risk	Cumulative failure (%)	Difference in failure (%)	95% CI	Equivalence status	p-value
[MoP] Exeter V40 Elite Plus Ogee	1,609	2.16	[REFERENCE]			
[CoC] ABG II Monolithic Cementless Stem ABG II Cementless Cup	259	2.44	0.28	[-1.36 , 1.92]	Non-inferiority not shown	0.739
[CoC] Corail Pinnacle	342	2.97	0.81	[0.10 , 1.52]	Non-inferiority not shown	0.025
[CoC] Exeter V40 Trident	727	2.29	0.13	[-0.60 , 0.85]	Non-inferiority not shown	0.731
[CoC] Furlong HAC Stem CSF	322	3.61	1.45	[-0.16 , 3.06]	Non-inferiority not shown	0.077
[CoP] Exeter V40 Trilogy	250	1.52	-0.64	[-1.72 , 0.43]	Non-inferiority not shown	0.243
[CoP] Furlong HAC Stem CSF	1,151	3.18	1.02	[0.17 , 1.88]	Non-inferiority not shown	0.019
[MoP] C-Stem Cemented Stem Charnley and Elite Plus LPW	376	3.32	1.16	[-0.26 , 2.57]	Non-inferiority not shown	0.108
[MoP] C-Stem Cemented Stem Elite Plus Ogee	373	2.97	0.81	[-0.42 , 2.04]	Non-inferiority not shown	0.198
[MoP] CPT Trilogy	562	4.25	2.09	[1.06 , 3.12]	Inferior by ≥20%	<0.001
[MoP] CPT ZCA	520	3.72	1.56	[0.48 , 2.64]	Inferior by ≥20%	0.005
[MoP] Charnley Cemented Stem Charnley Cemented Cup	782	3.09	0.93	[-0.10 , 1.96]	Non-inferiority not shown	0.076
[MoP] Charnley Cemented Stem Charnley Ogee	1,368	3.80	1.64	[0.77 , 2.51]	Inferior by ≥20%	<0.001
[MoP] Charnley Cemented Stem Charnley and Elite Plus LPW	1,079	2.64	0.48	[-0.38 , 1.34]	Non-inferiority not shown	0.275
[MoP] Corail Duraloc Cementless Cup	598	5.19	3.03	[1.73 , 4.32]	Inferior by ≥20%	<0.001
[MoP] Corail Pinnacle	849	2.37	0.21	[-0.43 , 0.84]	Non-inferiority not shown	0.521
[MoP] Exeter V40 Cenator Cemented Cup	312	3.32	1.16	[-0.28 , 2.59]	Non-inferiority not shown	0.114
[MoP] Exeter V40 Charnley Ogee	312	1.39	-0.77	[-1.74 , 0.19]	Non-inferior	0.116
[MoP] Exeter V40 Duraloc Cementless Cup	257	3.96	1.80	[-0.21 , 3.82]	Non-inferiority not shown	0.079
[MoP] Exeter V40 Elite Plus Cemented Cup	305	0.89	-1.27	[-2.07 , -0.48]	Non-inferior	0.002
[MoP] Exeter V40 Exeter Contemporary Flanged	2,152	2.29	0.13	[-0.43 , 0.70]	Non-inferiority not shown	0.644
[MoP] Exeter V40 Exeter Contemporary Hooded	1,110	4.86	2.70	[1.83 , 3.56]	Inferior by ≥20%	<0.001
[MoP] Exeter V40 Exeter Duration	1,344	3.44	1.28	[0.51 , 2.05]	Inferior by ≥20%	0.001
[MoP] Exeter V40 Trident	523	3.05	0.89	[0.06 , 1.72]	Non-inferiority not shown	0.035
[MoP] Exeter V40 Trilogy	748	2.13	-0.03	[-0.78 , 0.72]	Non-inferiority not shown	0.931
[MoP] Furlong Cemented Stem JRI Cemented Cup	268	4.14	1.98	[0.07 , 3.89]	Non-inferiority not shown	0.043
[MoP] Furlong HAC Stem CSF	726	3.72	1.56	[0.60 , 2.51]	Inferior by ≥20%	0.001
[MoP] Stanmore Modular Stem Stanmore-Arcom Cup	329	3.31	1.15	[-0.00 , 2.30]	Non-inferiority not shown	0.050

For peer review only

Supplemental table 5a: Difference in Kaplan-Meier failure estimate between a contemporary reference and implants with at least 250 at risk at 3 years since primary in females >75 years

Stem/cup brand	Number at risk	Cumulative failure (%)	Difference in failure (%)	95% CI	Equivalence status	p-value
[MoP] Charnley Cemented Stem Charnley and Elite Plus LPW	1,794	0.55	[REFERENCE]			
[CoC] Accolade Trident	251	2.21	1.66	[-0.12 , 3.44]	Non-inferiority not shown	0.067
[CoC] Corail Pinnacle	757	2.22	1.67	[0.67 , 2.66]	Inferior by ≥100%	0.001
[CoC] Furlong HAC Stem Furlong HAC CSF Plus	540	0.68	0.13	[-0.55 , 0.81]	Non-inferiority not shown	0.700
[CoP] Corail Pinnacle	431	0.69	0.14	[-0.48 , 0.76]	Non-inferiority not shown	0.653
[CoP] Exeter V40 Trident	424	0.34	-0.21	[-0.72 , 0.30]	Non-inferiority not shown	0.417
[CoP] Furlong HAC Stem CSF	693	1.24	0.69	[-0.14 , 1.52]	Non-inferiority not shown	0.103
[CoP] MS-30 Original ME Muller Low Profile Cup	300	0.00	- -	[-.-- , -.--]	No failures to date	
[MoP] Accolade Trident	1,623	1.59	1.04	[0.40 , 1.69]	Inferior by ≥20%	0.002
[MoP] C-Stem AMT Cemented Stem Charnley and Elite Plus LPW	646	1.09	0.54	[-0.24 , 1.32]	Non-inferiority not shown	0.176
[MoP] C-Stem AMT Cemented Stem Elite Plus Ogee	646	0.88	0.33	[-0.37 , 1.03]	Non-inferiority not shown	0.354
[MoP] C-Stem AMT Cemented Stem Marathon	343	0.68	0.13	[-0.52 , 0.78]	Non-inferiority not shown	0.691
[MoP] C-Stem AMT Cemented Stem Pinnacle	369	1.48	0.93	[0.12 , 1.74]	Inferior by ≥20%	0.024
[MoP] C-Stem Cemented Stem Charnley and Elite Plus LPW	384	1.43	0.88	[-0.30 , 2.06]	Non-inferiority not shown	0.145
[MoP] C-Stem Cemented Stem Elite Plus Ogee	808	0.43	-0.12	[-0.65 , 0.42]	Non-inferiority not shown	0.672
[MoP] C-Stem Cemented Stem Marathon	333	0.58	0.03	[-0.63 , 0.69]	Non-inferiority not shown	0.928
[MoP] C-Stem Cemented Stem Opera	270	0.34	-0.21	[-0.95 , 0.52]	Non-inferiority not shown	0.568
[MoP] CCA Cemented Stem CCB Cup	329	1.00	0.45	[-0.59 , 1.49]	Non-inferiority not shown	0.394
[MoP] CPCS Opera	309	0.61	0.06	[-0.84 , 0.95]	Non-inferiority not shown	0.904
[MoP] CPT Elite Plus Ogee	585	1.11	0.56	[-0.27 , 1.40]	Non-inferiority not shown	0.186
[MoP] CPT Original ME Muller Low Profile Cup	272	1.78	1.23	[-0.25 , 2.70]	Non-inferiority not shown	0.104
[MoP] CPT Trabecular Metal Modular Cementless Cup	306	0.92	0.37	[-0.60 , 1.34]	Non-inferiority not shown	0.451
[MoP] CPT Trilogy	2,024	1.12	0.57	[0.07 , 1.08]	Non-inferiority not shown	0.027
[MoP] CPT ZCA	3,039	1.37	0.82	[0.34 , 1.30]	Inferior by ≥20%	0.001
[MoP] Charnley Cemented Stem Charnley Cemented Cup	926	0.92	0.37	[-0.31 , 1.05]	Non-inferiority not shown	0.289
[MoP] Charnley Cemented Stem Charnley Ogee	2,041	0.79	0.24	[-0.25 , 0.72]	Non-inferiority not shown	0.344
[MoP] Charnley Cemented Stem Opera	306	0.93	0.38	[-0.72 , 1.49]	Non-inferiority not shown	0.494
[MoP] Charnley Cemented Stem Wroblewski Golf Ball	275	1.66	1.11	[-0.37 , 2.59]	Non-inferiority not shown	0.142
[MoP] Corail Duraloc Cementless Cup	535	0.68	0.13	[-0.61 , 0.87]	Non-inferiority not shown	0.727
[MoP] Corail Elite Plus Cemented Cup	272	0.65	0.10	[-0.86 , 1.06]	Non-inferiority not shown	0.841
[MoP] Corail Elite Plus Ogee	376	1.19	0.64	[-0.36 , 1.64]	Non-inferiority not shown	0.211
[MoP] Corail Marathon	690	0.92	0.37	[-0.28 , 1.01]	Non-inferiority not shown	0.264
[MoP] Corail Pinnacle	4,912	1.43	0.88	[0.46 , 1.30]	Inferior by ≥20%	<0.001
[MoP] Corail Trilogy	336	0.77	0.22	[-0.71 , 1.15]	Non-inferiority not shown	0.642
[MoP] Exeter V40 Cenator Cemented Cup	694	0.99	0.44	[-0.32 , 1.20]	Non-inferiority not shown	0.256
[MoP] Exeter V40 Charnley Ogee	321	0.59	0.04	[-0.84 , 0.92]	Non-inferiority not shown	0.930
[MoP] Exeter V40 Charnley and Elite Plus LPW	847	1.31	0.76	[-0.02 , 1.54]	Non-inferiority not shown	0.057
[MoP] Exeter V40 Elite Plus Cemented Cup	906	0.42	-0.14	[-0.62 , 0.35]	Non-inferiority not shown	0.587
[MoP] Exeter V40 Elite Plus Ogee	5,144	0.61	0.06	[-0.32 , 0.43]	Non-inferiority not shown	0.772

For peer review only

[MoP] Exeter V40 Exeter Contemporary Flanged	12,084	0.59	0.04	[-0.31 , 0.39]	Non-inferiority not shown	0.821
[MoP] Exeter V40 Exeter Contemporary Hooded	5,022	1.11	0.56	[0.15 , 0.97]	Inferior by ≥20%	0.007
[MoP] Exeter V40 Exeter Duration	3,745	0.79	0.24	[-0.18 , 0.65]	Non-inferiority not shown	0.265
[MoP] Exeter V40 Exeter X3 Rimfit	1,286	0.68	0.13	[-0.31 , 0.57]	Non-inferiority not shown	0.567
[MoP] Exeter V40 Marathon	365	0.95	0.40	[-0.38 , 1.18]	Non-inferiority not shown	0.319
[MoP] Exeter V40 Opera	732	0.48	-0.07	[-0.64 , 0.50]	Non-inferiority not shown	0.815
[MoP] Exeter V40 Pinnacle	736	0.83	0.28	[-0.31 , 0.87]	Non-inferiority not shown	0.358
[MoP] Exeter V40 Reflection Cementless	494	0.75	0.20	[-0.60 , 1.00]	Non-inferiority not shown	0.623
[MoP] Exeter V40 Trident	4,508	0.77	0.22	[-0.16 , 0.60]	Non-inferiority not shown	0.261
[MoP] Exeter V40 Trilogy	1,511	0.87	0.32	[-0.21 , 0.86]	Non-inferiority not shown	0.240
[MoP] Exeter V40 Ultima Cemented Cup	337	0.82	0.27	[-0.71 , 1.25]	Non-inferiority not shown	0.591
[MoP] Furlong Cemented Stem JRI Cemented Cup	531	0.88	0.33	[-0.51 , 1.16]	Non-inferiority not shown	0.442
[MoP] Furlong HAC Stem CSF	1,553	2.48	1.93	[1.15 , 2.70]	Inferior by ≥100%	<0.001
[MoP] Furlong HAC Stem Furlong HAC CSF Plus	792	2.58	2.03	[1.08 , 2.97]	Inferior by ≥100%	<0.001
[MoP] MS-30 Original ME Muller Low Profile Cup	401	0.36	-0.20	[-0.78 , 0.39]	Non-inferiority not shown	0.516
[MoP] Muller Straight Stem Original ME Muller Low Profile Cup	461	0.78	0.23	[-0.53 , 0.99]	Non-inferiority not shown	0.550
[MoP] Muller-Biomet Apollo	551	0.66	0.11	[-0.61 , 0.83]	Non-inferiority not shown	0.763
[MoP] Muller-Biomet Original ME Muller Low Profile Cup	342	1.08	0.53	[-0.57 , 1.63]	Non-inferiority not shown	0.346
[MoP] Omnifit Cemented Stem ODC	253	1.47	0.92	[-0.55 , 2.39]	Non-inferiority not shown	0.218
[MoP] SL-Plus Cementless Stem EP-Fit Plus	290	0.71	0.16	[-0.70 , 1.02]	Non-inferiority not shown	0.720
[MoP] Stanmore Modular Stem SHP Cup	367	0.64	0.09	[-0.70 , 0.89]	Non-inferiority not shown	0.819
[MoP] Stanmore Modular Stem Stanmore-Arcom Cup	1,325	0.86	0.31	[-0.26 , 0.87]	Non-inferiority not shown	0.289
[MoP] Taperloc Cementless Stem Exceed ABT	733	1.73	1.18	[0.37 , 1.99]	Inferior by ≥20%	0.004

For peer review only

Supplemental table 5b: Difference in Kaplan-Meier failure estimate between a contemporary reference and implants with at least 250 at risk at 5 years since primary in females >75 years

Stem/cup brand	Number at risk	Cumulative failure (%)	Difference in failure (%)	95% CI	Equivalence status	p-value
[MoP] Charnley Cemented Stem Charnley and Elite Plus LPW	1,515	0.73	[REFERENCE]			
[CoC] Corail Pinnacle	454	2.22	1.49	[0.47 , 2.50]	Inferior by ≥20%	0.004
[CoC] Furlong HAC Stem Furlong HAC CSF Plus	302	1.22	0.49	[-0.55 , 1.53]	Non-inferiority not shown	0.352
[CoP] Furlong HAC Stem CSF	539	1.56	0.83	[-0.13 , 1.80]	Non-inferiority not shown	0.089
[MoP] Accolade Trident	1,066	2.01	1.28	[0.53 , 2.03]	Inferior by ≥20%	0.001
[MoP] C-Stem AMT Cemented Stem Charnley and Elite Plus LPW	388	1.25	0.52	[-0.34 , 1.39]	Non-inferiority not shown	0.236
[MoP] C-Stem AMT Cemented Stem Elite Plus Ogee	345	0.88	0.15	[-0.57 , 0.88]	Non-inferiority not shown	0.682
[MoP] C-Stem Cemented Stem Charnley and Elite Plus LPW	328	1.73	1.00	[-0.33 , 2.33]	Non-inferiority not shown	0.141
[MoP] C-Stem Cemented Stem Elite Plus Ogee	614	0.57	-0.16	[-0.79 , 0.47]	Non-inferiority not shown	0.615
[MoP] CPT Elite Plus Ogee	388	1.49	0.76	[-0.24 , 1.77]	Non-inferiority not shown	0.136
[MoP] CPT Trilogy	1,273	1.88	1.15	[0.47 , 1.83]	Inferior by ≥20%	0.001
[MoP] CPT ZCA	2,186	1.86	1.13	[0.55 , 1.71]	Inferior by ≥20%	<0.001
[MoP] Charnley Cemented Stem Charnley Cemented Cup	780	1.27	0.54	[-0.27 , 1.36]	Non-inferiority not shown	0.190
[MoP] Charnley Cemented Stem Charnley Ogee	1,658	1.15	0.42	[-0.17 , 1.01]	Non-inferiority not shown	0.162
[MoP] Corail Duraloc Cementless Cup	479	1.66	0.93	[-0.21 , 2.07]	Non-inferiority not shown	0.111
[MoP] Corail Marathon	325	0.92	0.19	[-0.49 , 0.86]	Non-inferiority not shown	0.585
[MoP] Corail Pinnacle	2,638	1.78	1.05	[0.55 , 1.55]	Inferior by ≥20%	<0.001
[MoP] Corail Trilogy	261	1.11	0.39	[-0.78 , 1.55]	Non-inferiority not shown	0.515
[MoP] Exeter V40 Cenator Cemented Cup	533	1.32	0.59	[-0.31 , 1.49]	Non-inferiority not shown	0.201
[MoP] Exeter V40 Charnley Ogee	264	1.27	0.54	[-0.76 , 1.85]	Non-inferiority not shown	0.414
[MoP] Exeter V40 Charnley and Elite Plus LPW	575	1.31	0.58	[-0.23 , 1.39]	Non-inferiority not shown	0.159
[MoP] Exeter V40 Elite Plus Cemented Cup	653	0.53	-0.20	[-0.77 , 0.38]	Non-inferiority not shown	0.502
[MoP] Exeter V40 Elite Plus Ogee	3,667	0.84	0.11	[-0.34 , 0.56]	Non-inferiority not shown	0.644
[MoP] Exeter V40 Exeter Contemporary Flanged	7,444	0.86	0.13	[-0.28 , 0.55]	Non-inferiority not shown	0.524
[MoP] Exeter V40 Exeter Contemporary Hooded	3,318	1.47	0.74	[0.24 , 1.23]	Inferior by ≥20%	0.003
[MoP] Exeter V40 Exeter Duration	2,928	1.18	0.46	[-0.05 , 0.96]	Non-inferiority not shown	0.078
[MoP] Exeter V40 Opera	530	0.63	-0.10	[-0.77 , 0.57]	Non-inferiority not shown	0.768
[MoP] Exeter V40 Pinnacle	386	1.03	0.30	[-0.44 , 1.04]	Non-inferiority not shown	0.422
[MoP] Exeter V40 Reflection Cementless	402	0.96	0.23	[-0.69 , 1.16]	Non-inferiority not shown	0.620
[MoP] Exeter V40 Trident	2,517	1.05	0.32	[-0.15 , 0.79]	Non-inferiority not shown	0.179
[MoP] Exeter V40 Trilogy	1,080	1.32	0.59	[-0.08 , 1.27]	Non-inferiority not shown	0.085
[MoP] Exeter V40 Ultima Cemented Cup	300	1.15	0.42	[-0.77 , 1.61]	Non-inferiority not shown	0.488
[MoP] Furlong Cemented Stem JRI Cemented Cup	426	0.88	0.15	[-0.71 , 1.00]	Non-inferiority not shown	0.734
[MoP] Furlong HAC Stem CSF	1,201	3.07	2.34	[1.44 , 3.24]	Inferior by ≥100%	<0.001
[MoP] Furlong HAC Stem Furlong HAC CSF Plus	481	2.71	1.98	[0.98 , 2.98]	Inferior by ≥100%	<0.001
[MoP] MS-30 Original ME Muller Low Profile Cup	259	1.30	0.57	[-0.67 , 1.81]	Non-inferiority not shown	0.366
[MoP] Muller Straight Stem Original ME Muller Low Profile Cup	319	1.04	0.31	[-0.62 , 1.25]	Non-inferiority not shown	0.514
[MoP] Muller-Biomet Apollo	422	0.86	0.13	[-0.71 , 0.98]	Non-inferiority not shown	0.759
[MoP] Stanmore Modular Stem Stanmore-Arcom Cup	979	1.12	0.39	[-0.28 , 1.06]	Non-inferiority not shown	0.253

For peer review only

[MoP] Taperloc Cementless Stem Exceed ABT	337	1.95	1.22	[0.29 , 2.15]	Inferior by ≥20%	0.010

For peer review only

Supplemental table 5c: Difference in Kaplan-Meier failure estimate between a contemporary reference and implants with at least 250 at risk at 7 years since primary in females >75 years

Stem/cup brand	Number at risk	Cumulative failure (%)	Difference in failure (%)	95% CI	Equivalence status	p-value
[MoP] Charnley Cemented Stem Charnley and Elite Plus LPW	1,119	0.88	[REFERENCE]			
[CoP] Furlong HAC Stem CSF	391	2.04	1.16	[-0.02 , 2.34]	Non-inferiority not shown	0.055
[MoP] Accolade Trident	460	2.40	1.52	[0.61 , 2.42]	Inferior by ≥20%	0.001
[MoP] C-Stem Cemented Stem Charnley and Elite Plus LPW	292	1.73	0.85	[-0.50 , 2.19]	Non-inferiority not shown	0.218
[MoP] C-Stem Cemented Stem Elite Plus Ogee	444	0.57	-0.31	[-0.98 , 0.35]	Non-inferiority not shown	0.354
[MoP] CPT Trilogy	689	2.22	1.34	[0.53 , 2.16]	Inferior by ≥20%	0.001
[MoP] CPT ZCA	1,341	2.44	1.56	[0.85 , 2.27]	Inferior by ≥20%	<0.001
[MoP] Charnley Cemented Stem Charnley Cemented Cup	605	1.72	0.84	[-0.14 , 1.81]	Non-inferiority not shown	0.093
[MoP] Charnley Cemented Stem Charnley Ogee	1,236	1.35	0.47	[-0.20 , 1.13]	Non-inferiority not shown	0.167
[MoP] Corail Duraloc Cementless Cup	363	2.36	1.48	[0.08 , 2.88]	Non-inferiority not shown	0.039
[MoP] Corail Pinnacle	1,150	2.17	1.28	[0.67 , 1.90]	Inferior by ≥20%	<0.001
[MoP] Exeter V40 Cenator Cemented Cup	373	1.32	0.44	[-0.49 , 1.37]	Non-inferiority not shown	0.355
[MoP] Exeter V40 Charnley and Elite Plus LPW	309	1.31	0.43	[-0.41 , 1.26]	Non-inferiority not shown	0.313
[MoP] Exeter V40 Elite Plus Cemented Cup	429	0.95	0.07	[-0.77 , 0.90]	Non-inferiority not shown	0.879
[MoP] Exeter V40 Elite Plus Ogee	2,329	1.18	0.30	[-0.24 , 0.83]	Non-inferiority not shown	0.283
[MoP] Exeter V40 Exeter Contemporary Flanged	4,187	1.13	0.24	[-0.24 , 0.73]	Non-inferiority not shown	0.320
[MoP] Exeter V40 Exeter Contemporary Hooded	1,866	2.16	1.28	[0.66 , 1.90]	Inferior by ≥20%	<0.001
[MoP] Exeter V40 Exeter Duration	1,961	1.66	0.78	[0.17 , 1.39]	Non-inferiority not shown	0.012
[MoP] Exeter V40 Opera	317	0.85	-0.03	[-0.86 , 0.80]	Non-inferiority not shown	0.950
[MoP] Exeter V40 Reflection Cementless	258	1.34	0.46	[-0.74 , 1.66]	Non-inferiority not shown	0.452
[MoP] Exeter V40 Trident	1,221	1.27	0.39	[-0.17 , 0.95]	Non-inferiority not shown	0.168
[MoP] Exeter V40 Trilogy	618	1.41	0.53	[-0.20 , 1.26]	Non-inferiority not shown	0.151
[MoP] Furlong Cemented Stem JRI Cemented Cup	307	1.11	0.23	[-0.76 , 1.22]	Non-inferiority not shown	0.649
[MoP] Furlong HAC Stem CSF	882	3.51	2.63	[1.63 , 3.63]	Inferior by ≥100%	<0.001
[MoP] Muller-Biomet Apollo	288	0.86	-0.02	[-0.89 , 0.85]	Non-inferiority not shown	0.965
[MoP] Stanmore Modular Stem Stanmore-Arcom Cup	575	1.36	0.48	[-0.29 , 1.26]	Non-inferiority not shown	0.223

For peer review only

Supplemental table 6a: Difference in Kaplan-Meier failure estimate between a contemporary reference and implants with at least 250 at risk at 3 years since primary in males

Stem/cup brand	Number at risk	Cumulative failure (%)	Difference in failure (%)	95% CI	Equivalence status	p-value
[CoP] Exeter V40 Exeter Contemporary Flanged	1,147	0.76	[REFERENCE]			
[CoC] ABG II Monolithic Cementless Stem ABG II Cementless Cup	560	2.22	1.46	[0.19 , 2.73]	Inferior by ≥20%	0.024
[CoC] ABG II Monolithic Cementless Stem Trident	544	1.76	1.00	[-0.16 , 2.17]	Non-inferiority not shown	0.091
[CoC] Accolade Trident	3,001	2.33	1.57	[0.90 , 2.25]	Inferior by ≥100%	<0.001
[CoC] Bimetric Cementless Stem Exceed ABT	377	0.89	0.14	[-0.84 , 1.11]	Non-inferiority not shown	0.784
[CoC] C-Stem AMT Cemented Stem Pinnacle	284	0.43	-0.33	[-1.06 , 0.40]	Non-inferiority not shown	0.380
[CoC] CPT Continuum	321	2.54	1.78	[0.30 , 3.27]	Inferior by ≥20%	0.019
[CoC] Corail Delta TT	274	2.45	1.69	[0.11 , 3.27]	Non-inferiority not shown	0.036
[CoC] Corail DeltaMotion	452	1.67	0.91	[-0.25 , 2.08]	Non-inferiority not shown	0.125
[CoC] Corail Duraloc Option	463	2.29	1.53	[0.13 , 2.94]	Non-inferiority not shown	0.033
[CoC] Corail Pinnacle	12,616	1.92	1.16	[0.68 , 1.64]	Inferior by ≥20%	<0.001
[CoC] Excia Cementless Plasmacup SC	419	0.85	0.09	[-0.77 , 0.95]	Non-inferiority not shown	0.832
[CoC] Exeter V40 ABG II Cementless Cup	457	1.13	0.38	[-0.62 , 1.38]	Non-inferiority not shown	0.460
[CoC] Exeter V40 Trident	4,326	1.13	0.37	[-0.15 , 0.89]	Non-inferiority not shown	0.158
[CoC] Furlong Evolution Cementless Furlong HAC CSF Plus	275	2.00	1.24	[0.12 , 2.36]	Non-inferiority not shown	0.030
[CoC] Furlong HAC Stem CSF	688	2.76	2.00	[0.73 , 3.27]	Inferior by ≥20%	0.002
[CoC] Furlong HAC Stem Furlong HAC CSF Plus	4,023	1.72	0.96	[0.41 , 1.51]	Inferior by ≥20%	0.001
[CoC] M/L Taper Cementless Continuum	704	1.73	0.97	[0.05 , 1.90]	Non-inferiority not shown	0.040
[CoC] Metafix Stem Trinity	344	1.09	0.33	[-0.59 , 1.26]	Non-inferiority not shown	0.479
[CoC] Polarstem Cementless R3 Cementless	341	0.37	-0.39	[-1.06 , 0.27]	Non-inferiority not shown	0.248
[CoC] SL-Plus Cementless Stem EP-Fit Plus	601	5.11	4.36	[2.60 , 6.11]	Inferior by ≥100%	<0.001
[CoC] SL-Plus Cementless Stem R3 Cementless	298	1.48	0.72	[-0.64 , 2.08]	Non-inferiority not shown	0.298
[CoC] SPS Modular April - Ceramic	291	0.59	-0.17	[-1.09 , 0.75]	Non-inferiority not shown	0.722
[CoC] Taperloc Cementless Stem Exceed ABT	2,976	1.64	0.88	[0.30 , 1.46]	Inferior by ≥20%	0.003
[CoC] miniHip Trinity	273	3.21	2.46	[0.68 , 4.24]	Inferior by ≥20%	0.007
[CoP] Accolade Trident	1,319	1.66	0.91	[0.21 , 1.60]	Inferior by ≥20%	0.011
[CoP] C-Stem Cemented Stem Elite Plus Ogee	265	0.93	0.17	[-0.96 , 1.30]	Non-inferiority not shown	0.771
[CoP] C-Stem Cemented Stem Marathon	526	1.07	0.31	[-0.49 , 1.11]	Non-inferiority not shown	0.447
[CoP] C-Stem Cemented Stem Opera	341	1.15	0.39	[-0.81 , 1.60]	Non-inferiority not shown	0.520
[CoP] C-Stem Cemented Stem Wroblewski Golf Ball	396	0.90	0.14	[-0.84 , 1.12]	Non-inferiority not shown	0.779
[CoP] CPT Trilogy	450	1.91	1.15	[0.24 , 2.05]	Inferior by ≥20%	0.013
[CoP] Corail Charnley and Elite Plus LPW	252	1.10	0.35	[-0.97 , 1.66]	Non-inferiority not shown	0.606
[CoP] Corail Marathon	525	1.69	0.93	[-0.02 , 1.89]	Non-inferiority not shown	0.055
[CoP] Corail Pinnacle	3,493	1.14	0.38	[-0.11 , 0.88]	Non-inferiority not shown	0.131
[CoP] Exeter V40 Charnley and Elite Plus LPW	300	1.07	0.31	[-0.82 , 1.44]	Non-inferiority not shown	0.593
[CoP] Exeter V40 Elite Plus Ogee	536	0.86	0.10	[-0.71 , 0.92]	Non-inferiority not shown	0.803
[CoP] Exeter V40 Exeter Contemporary Hooded	298	2.35	1.59	[-0.00 , 3.18]	Non-inferiority not shown	0.050
[CoP] Exeter V40 Exeter Duration	300	0.27	-0.49	[-1.17 , 0.18]	Non-inferiority not shown	0.152
[CoP] Exeter V40 Exeter X3 Rimfit	911	1.32	0.56	[-0.15 , 1.27]	Non-inferiority not shown	0.120
[CoP] Exeter V40 Pinnacle	251	1.17	0.41	[-0.58 , 1.40]	Non-inferiority not shown	0.418

For peer review only

[CoP] Exeter V40 Trident	1,917	0.79	0.04	[-0.47 , 0.55]	Non-inferiority not shown	0.887
[CoP] Exeter V40 Trilogy	764	1.73	0.97	[0.00 , 1.94]	Non-inferiority not shown	0.050
[CoP] Furlong HAC Stem CSF	2,536	1.18	0.42	[-0.17 , 1.01]	Non-inferiority not shown	0.162
[CoP] Furlong HAC Stem Furlong HAC CSF Plus	699	1.22	0.46	[-0.33 , 1.26]	Non-inferiority not shown	0.250
[CoP] MS-30 Original ME Muller Low Profile Cup	459	0.35	-0.41	[-1.06 , 0.24]	Non-inferiority not shown	0.217
[CoP] SL-Plus Cementless Stem Bicon-Plus	279	3.64	2.88	[0.73 , 5.04]	Inferior by ≥20%	0.009
[CoP] SL-Plus Cementless Stem EP-Fit Plus	366	1.82	1.06	[-0.34 , 2.47]	Non-inferiority not shown	0.138
[CoP] Taperloc Cementless Stem Exceed ABT	821	1.17	0.41	[-0.27 , 1.09]	Non-inferiority not shown	0.240
[MoP] Accolade Trident	3,597	2.40	1.65	[1.02 , 2.27]	Inferior by ≥100%	<0.001
[MoP] Anthology R3 Cementless	427	2.22	1.46	[0.36 , 2.56]	Inferior by ≥20%	0.009
[MoP] C-Stem AMT Cemented Stem Charnley and Elite Plus LPW	717	1.65	0.89	[-0.07 , 1.85]	Non-inferiority not shown	0.070
[MoP] C-Stem AMT Cemented Stem Elite Plus Ogee	473	0.61	-0.15	[-0.89 , 0.59]	Non-inferiority not shown	0.690
[MoP] C-Stem AMT Cemented Stem Marathon	406	1.04	0.28	[-0.47 , 1.03]	Non-inferiority not shown	0.461
[MoP] C-Stem AMT Cemented Stem Pinnacle	495	1.18	0.42	[-0.37 , 1.22]	Non-inferiority not shown	0.296
[MoP] C-Stem Cemented Stem Charnley Ogee	251	0.91	0.15	[-0.96 , 1.26]	Non-inferiority not shown	0.794
[MoP] C-Stem Cemented Stem Charnley and Elite Plus LPW	476	0.79	0.03	[-0.85 , 0.92]	Non-inferiority not shown	0.942
[MoP] C-Stem Cemented Stem Elite Plus Cemented Cup	261	0.70	-0.06	[-1.12 , 1.00]	Non-inferiority not shown	0.918
[MoP] C-Stem Cemented Stem Elite Plus Ogee	1,224	1.18	0.42	[-0.29 , 1.12]	Non-inferiority not shown	0.243
[MoP] C-Stem Cemented Stem Marathon	597	1.40	0.65	[-0.25 , 1.54]	Non-inferiority not shown	0.155
[MoP] C-Stem Cemented Stem Opera	528	1.62	0.86	[-0.27 , 2.00]	Non-inferiority not shown	0.137
[MoP] C-Stem Cemented Stem Wroblewski Golf Ball	354	1.29	0.53	[-0.67 , 1.73]	Non-inferiority not shown	0.388
[MoP] CCA Cemented Stem CCB Cup	337	0.47	-0.28	[-1.07 , 0.50]	Non-inferiority not shown	0.480
[MoP] CLS Cementless Stem Allofit	265	3.32	2.56	[0.49 , 4.63]	Inferior by ≥20%	0.015
[MoP] CPCS Opera	371	0.51	-0.24	[-1.07 , 0.59]	Non-inferiority not shown	0.567
[MoP] CPT Elite Plus Ogee	862	1.64	0.88	[-0.02 , 1.79]	Non-inferiority not shown	0.056
[MoP] CPT Trilogy	2,750	1.70	0.95	[0.35 , 1.54]	Inferior by ≥20%	0.002
[MoP] CPT ZCA	2,153	1.52	0.76	[0.15 , 1.37]	Non-inferiority not shown	0.015
[MoP] Charnley Cemented Stem Charnley Cemented Cup	1,527	1.34	0.58	[-0.12 , 1.28]	Non-inferiority not shown	0.107
[MoP] Charnley Cemented Stem Charnley Ogee	3,293	1.35	0.59	[0.02 , 1.16]	Non-inferiority not shown	0.043
[MoP] Charnley Cemented Stem Charnley and Elite Plus LPW	1,647	0.97	0.22	[-0.41 , 0.84]	Non-inferiority not shown	0.496
[MoP] Charnley Cemented Stem Opera	407	1.15	0.39	[-0.70 , 1.49]	Non-inferiority not shown	0.479
[MoP] Charnley Cemented Stem Wroblewski Golf Ball	382	1.45	0.69	[-0.54 , 1.92]	Non-inferiority not shown	0.270
[MoP] Corail Duraloc Cementless Cup	1,310	1.59	0.83	[0.05 , 1.62]	Non-inferiority not shown	0.038
[MoP] Corail Elite Plus Cemented Cup	337	1.52	0.76	[-0.52 , 2.04]	Non-inferiority not shown	0.245
[MoP] Corail Elite Plus Ogee	471	1.51	0.75	[-0.32 , 1.82]	Non-inferiority not shown	0.171
[MoP] Corail Marathon	978	1.50	0.74	[-0.00 , 1.48]	Non-inferiority not shown	0.050
[MoP] Corail Pinnacle	11,121	1.53	0.78	[0.31 , 1.25]	Inferior by ≥20%	0.001
[MoP] Corail Trilogy	726	1.48	0.72	[-0.21 , 1.66]	Non-inferiority not shown	0.131
[MoP] Exeter V40 ABG II Cementless Cup	258	2.15	1.39	[-0.36 , 3.15]	Non-inferiority not shown	0.120
[MoP] Exeter V40 Cenator Cemented Cup	705	1.30	0.55	[-0.37 , 1.46]	Non-inferiority not shown	0.240
[MoP] Exeter V40 Charnley and Elite Plus LPW	667	1.09	0.33	[-0.50 , 1.16]	Non-inferiority not shown	0.437
[MoP] Exeter V40 Duraloc Cementless Cup	355	2.15	1.40	[-0.14 , 2.93]	Non-inferiority not shown	0.075
[MoP] Exeter V40 Elite Plus Cemented Cup	1,212	0.84	0.08	[-0.56 , 0.72]	Non-inferiority not shown	0.809
[MoP] Exeter V40 Elite Plus Ogee	5,579	0.96	0.21	[-0.29 , 0.70]	Non-inferiority not shown	0.413
[MoP] Exeter V40 Exeter Contemporary Flanged	13,647	1.12	0.36	[-0.09 , 0.82]	Non-inferiority not shown	0.118
[MoP] Exeter V40 Exeter Contemporary Hooded	5,115	1.83	1.07	[0.53 , 1.60]	Inferior by ≥20%	<0.001

For peer review only

[MoP] Exeter V40 Exeter Duration	4,042	1.60	0.85	[0.29 , 1.41]	Inferior by ≥20%	0.003
[MoP] Exeter V40 Exeter X3 Rimfit	1,854	1.41	0.65	[0.06 , 1.24]	Non-inferiority not shown	0.031
[MoP] Exeter V40 Marathon	418	1.41	0.65	[-0.32 , 1.61]	Non-inferiority not shown	0.188
[MoP] Exeter V40 Opera	714	1.04	0.28	[-0.56 , 1.11]	Non-inferiority not shown	0.514
[MoP] Exeter V40 Pinnacle	784	1.67	0.91	[0.08 , 1.75]	Non-inferiority not shown	0.033
[MoP] Exeter V40 Reflection Cementless	729	1.79	1.04	[0.01 , 2.06]	Non-inferiority not shown	0.048
[MoP] Exeter V40 Trident	6,504	1.31	0.55	[0.07 , 1.04]	Non-inferiority not shown	0.025
[MoP] Exeter V40 Trilogy	3,337	1.06	0.31	[-0.23 , 0.85]	Non-inferiority not shown	0.265
[MoP] Exeter V40 Ultima Cemented Cup	363	2.49	1.73	[0.15 , 3.32]	Non-inferiority not shown	0.032
[MoP] Furlong Cemented Stem JRI Cemented Cup	443	1.25	0.50	[-0.59 , 1.58]	Non-inferiority not shown	0.370
[MoP] Furlong HAC Stem CSF	2,566	1.90	1.14	[0.49 , 1.79]	Inferior by ≥20%	0.001
[MoP] Furlong HAC Stem Furlong HAC CSF Plus	1,170	2.43	1.68	[0.85 , 2.50]	Inferior by ≥100%	<0.001
[MoP] M/L Taper Cementless Allofit	339	1.83	1.08	[-0.26 , 2.41]	Non-inferiority not shown	0.113
[MoP] Muller Straight Stem Original ME Muller Low Profile Cup	414	0.52	-0.24	[-0.97 , 0.49]	Non-inferiority not shown	0.522
[MoP] Muller-Biomet Apollo	748	1.13	0.37	[-0.48 , 1.22]	Non-inferiority not shown	0.395
[MoP] Omnifit Cemented Stem ODC	267	1.04	0.28	[-0.96 , 1.52]	Non-inferiority not shown	0.661
[MoP] Polarstem Cementless R3 Cementless	476	1.24	0.48	[-0.21 , 1.18]	Non-inferiority not shown	0.174
[MoP] SL-Plus Cementless Stem EP-Fit Plus	826	2.89	2.13	[1.01 , 3.26]	Inferior by ≥100%	<0.001
[MoP] Stanmore Modular Stem SHP Cup	267	0.99	0.23	[-0.97 , 1.44]	Non-inferiority not shown	0.702
[MoP] Stanmore Modular Stem Stanmore-Arcom Cup	1,070	1.02	0.26	[-0.44 , 0.97]	Non-inferiority not shown	0.461
[MoP] Synergy Cementless Stem R3 Cementless	503	1.52	0.76	[-0.10 , 1.63]	Non-inferiority not shown	0.083
[MoP] Synergy Cementless Stem Reflection Cementless	591	1.14	0.38	[-0.56 , 1.32]	Non-inferiority not shown	0.429
[MoP] Taperloc Cementless Stem Exceed ABT	1,447	1.40	0.64	[0.00 , 1.28]	Non-inferiority not shown	0.049
[MoP] Versys Cementless Stem Trilogy	369	4.30	3.54	[1.49 , 5.58]	Inferior by ≥100%	0.001

Supplemental table 6b: Difference in Kaplan-Meier failure estimate between a contemporary reference and implants with at least 250 at risk at 5 years since primary in males

Stem/cup brand	Number at risk	Cumulative failure (%)	Difference in failure (%)	95% CI	Equivalence status	p-value
[MoP] Exeter V40 Elite Plus Ogee	4,143	1.29	[REFERENCE]			
[CoC] ABG II Monolithic Cementless Stem ABG II Cementless Cup	536	2.75	1.47	[0.11 , 2.82]	Non-inferiority not shown	0.035
[CoC] ABG II Monolithic Cementless Stem Trident	463	3.15	1.86	[0.36 , 3.37]	Inferior by ≥20%	0.015
[CoC] Accolade Trident	2,264	3.15	1.87	[1.19 , 2.54]	Inferior by ≥20%	<0.001
[CoC] Bimetric Cementless Stem Exceed ABT	259	1.75	0.46	[-0.86 , 1.79]	Non-inferiority not shown	0.494
[CoC] Corail DeltaMotion	261	1.91	0.62	[-0.59 , 1.83]	Non-inferiority not shown	0.315
[CoC] Corail Duraloc Option	449	3.36	2.07	[0.43 , 3.72]	Inferior by ≥20%	0.013
[CoC] Corail Pinnacle	7,860	2.68	1.40	[1.01 , 1.79]	Inferior by ≥20%	<0.001
[CoC] Exeter V40 ABG II Cementless Cup	379	1.39	0.10	[-0.97 , 1.17]	Non-inferiority not shown	0.852
[CoC] Exeter V40 Trident	3,289	1.81	0.52	[0.04 , 1.00]	Non-inferiority not shown	0.034
[CoC] Furlong HAC Stem CSF	664	3.19	1.91	[0.59 , 3.22]	Inferior by ≥20%	0.005
[CoC] Furlong HAC Stem Furlong HAC CSF Plus	2,393	1.91	0.62	[0.15 , 1.10]	Non-inferiority not shown	0.010
[CoC] M/L Taper Cementless Continuum	304	1.73	0.44	[-0.42 , 1.31]	Non-inferiority not shown	0.316
[CoC] SL-Plus Cementless Stem EP-Fit Plus	551	6.43	5.14	[3.22 , 7.07]	Inferior by ≥100%	<0.001

For peer review only

For peer review only - http://bmjopen.bmj.com/site/about/guidelines.xhtml

[CoC] Taperloc Cementless Stem Exceed ABT	1,657	1.87	0.59	[0.07 , 1.11]	Non-inferiority not shown	0.027
[CoP] Accolade Trident	544	2.06	0.77	[0.05 , 1.50]	Non-inferiority not shown	0.037
[CoP] C-Stem Cemented Stem Opera	283	1.49	0.21	[-1.13 , 1.54]	Non-inferiority not shown	0.759
[CoP] C-Stem Cemented Stem Wroblewski Golf Ball	321	1.47	0.18	[-1.03 , 1.39]	Non-inferiority not shown	0.769
[CoP] Corail Pinnacle	1,695	1.78	0.49	[0.01 , 0.98]	Non-inferiority not shown	0.047
[CoP] Exeter V40 Elite Plus Ogee	381	1.11	-0.18	[-1.06 , 0.71]	Non-inferiority not shown	0.697
[CoP] Exeter V40 Exeter Contemporary Flanged	789	0.85	-0.44	[-0.98 , 0.11]	Non-inferior	0.116
[CoP] Exeter V40 Exeter Duration	261	1.01	-0.28	[-1.46 , 0.90]	Non-inferiority not shown	0.641
[CoP] Exeter V40 Trident	891	0.96	-0.33	[-0.79 , 0.13]	Non-inferior	0.162
[CoP] Exeter V40 Trilogy	631	2.00	0.72	[-0.27 , 1.71]	Non-inferiority not shown	0.154
[CoP] Furlong HAC Stem CSF	2,209	1.39	0.10	[-0.42 , 0.62]	Non-inferiority not shown	0.703
[CoP] Furlong HAC Stem Furlong HAC CSF Plus	400	1.38	0.10	[-0.69 , 0.88]	Non-inferiority not shown	0.809
[CoP] MS-30 Original ME Muller Low Profile Cup	330	0.35	-0.94	[-1.50 , -0.38]	Non-inferior	0.001
[CoP] SL-Plus Cementless Stem Bicon-Plus	253	4.74	3.46	[1.01 , 5.90]	Inferior by ≥20%	0.006
[CoP] SL-Plus Cementless Stem EP-Fit Plus	312	3.28	1.99	[0.14 , 3.85]	Non-inferiority not shown	0.035
[CoP] Taperloc Cementless Stem Exceed ABT	309	1.70	0.42	[-0.54 , 1.37]	Non-inferiority not shown	0.391
[MoP] Accolade Trident	2,404	3.37	2.08	[1.45 , 2.71]	Inferior by ≥100%	<0.001
[MoP] C-Stem AMT Cemented Stem Charnley and Elite Plus LPW	444	1.81	0.52	[-0.44 , 1.47]	Non-inferiority not shown	0.286
[MoP] C-Stem AMT Cemented Stem Elite Plus Ogee	253	0.61	-0.68	[-1.34 , -0.01]	Non-inferior	0.045
[MoP] C-Stem Cemented Stem Charnley and Elite Plus LPW	429	1.01	-0.27	[-1.20 , 0.66]	Non-inferiority not shown	0.564
[MoP] C-Stem Cemented Stem Elite Plus Ogee	906	1.28	0.00	[-0.66 , 0.66]	Non-inferiority not shown	0.997
[MoP] C-Stem Cemented Stem Marathon	274	1.82	0.54	[-0.48 , 1.55]	Non-inferiority not shown	0.300
[MoP] C-Stem Cemented Stem Opera	389	2.23	0.94	[-0.34 , 2.23]	Non-inferiority not shown	0.149
[MoP] C-Stem Cemented Stem Wroblewski Golf Ball	269	1.29	0.00	[-1.16 , 1.16]	Non-inferiority not shown	0.999
[MoP] CPCS Opera	281	0.51	-0.77	[-1.54 , -0.01]	Non-inferior	0.048
[MoP] CPT Elite Plus Ogee	630	2.35	1.07	[0.02 , 2.11]	Non-inferiority not shown	0.046
[MoP] CPT Trilogy	1,862	2.39	1.11	[0.50 , 1.71]	Inferior by ≥20%	<0.001
[MoP] CPT ZCA	1,549	2.42	1.13	[0.46 , 1.80]	Inferior by ≥20%	0.001
[MoP] Charnley Cemented Stem Charnley Cemented Cup	1,338	2.22	0.93	[0.15 , 1.71]	Non-inferiority not shown	0.019
[MoP] Charnley Cemented Stem Charnley Ogee	2,824	2.29	1.00	[0.42 , 1.58]	Inferior by ≥20%	0.001
[MoP] Charnley Cemented Stem Charnley and Elite Plus LPW	1,414	1.42	0.14	[-0.49 , 0.76]	Non-inferiority not shown	0.667
[MoP] Charnley Cemented Stem Opera	343	1.68	0.40	[-0.88 , 1.67]	Non-inferiority not shown	0.540
[MoP] Charnley Cemented Stem Wroblewski Golf Ball	311	1.75	0.47	[-0.86 , 1.79]	Non-inferiority not shown	0.489
[MoP] Corail Duraloc Cementless Cup	1,204	2.54	1.25	[0.36 , 2.14]	Inferior by ≥20%	0.006
[MoP] Corail Elite Plus Ogee	352	1.79	0.50	[-0.66 , 1.66]	Non-inferiority not shown	0.397
[MoP] Corail Marathon	465	1.77	0.48	[-0.28 , 1.24]	Non-inferiority not shown	0.216
[MoP] Corail Pinnacle	6,318	1.91	0.63	[0.26 , 0.99]	Inferior by ≥20%	0.001
[MoP] Corail Trilogy	526	2.48	1.20	[0.01 , 2.38]	Non-inferiority not shown	0.047
[MoP] Exeter V40 Cenator Cemented Cup	573	1.74	0.45	[-0.53 , 1.43]	Non-inferiority not shown	0.366
[MoP] Exeter V40 Charnley and Elite Plus LPW	504	1.09	-0.20	[-0.97 , 0.57]	Non-inferiority not shown	0.612
[MoP] Exeter V40 Duraloc Cementless Cup	330	2.45	1.16	[-0.44 , 2.77]	Non-inferiority not shown	0.156
[MoP] Exeter V40 Elite Plus Cemented Cup	906	1.20	-0.09	[-0.74 , 0.57]	Non-inferiority not shown	0.797
[MoP] Exeter V40 Exeter Contemporary Flanged	8,757	1.54	0.26	[-0.09 , 0.60]	Non-inferiority not shown	0.145
[MoP] Exeter V40 Exeter Contemporary Hooded	3,570	2.27	0.98	[0.51 , 1.45]	Inferior by ≥20%	<0.001
[MoP] Exeter V40 Exeter Duration	3,104	2.30	1.02	[0.49 , 1.54]	Inferior by ≥20%	<0.001
[MoP] Exeter V40 Exeter X3 Rimfit	309	1.61	0.33	[-0.31 , 0.96]	Non-inferiority not shown	0.314

For peer review only

[MoP] Exeter V40 Opera	515	1.66	0.37	[-0.61 , 1.35]	Non-inferiority not shown	0.457
[MoP] Exeter V40 Pinnacle	471	2.43	1.14	[0.13 , 2.15]	Non-inferiority not shown	0.027
[MoP] Exeter V40 Reflection Cementless	649	2.49	1.21	[0.06 , 2.35]	Non-inferiority not shown	0.039
[MoP] Exeter V40 Trident	3,769	1.68	0.40	[-0.00 , 0.80]	Non-inferiority not shown	0.051
[MoP] Exeter V40 Trilogy	2,555	1.46	0.18	[-0.31 , 0.66]	Non-inferiority not shown	0.473
[MoP] Exeter V40 Ultima Cemented Cup	321	2.77	1.48	[-0.16 , 3.12]	Non-inferiority not shown	0.077
[MoP] Furlong Cemented Stem JRI Cemented Cup	396	1.48	0.19	[-0.93 , 1.32]	Non-inferiority not shown	0.735
[MoP] Furlong HAC Stem CSF	2,076	2.31	1.02	[0.40 , 1.64]	Inferior by ≥20%	0.001
[MoP] Furlong HAC Stem Furlong HAC CSF Plus	686	3.15	1.86	[0.94 , 2.79]	Inferior by ≥20%	<0.001
[MoP] Muller Straight Stem Original ME Muller Low Profile Cup	291	0.78	-0.51	[-1.33 , 0.31]	Non-inferiority not shown	0.224
[MoP] Muller-Biomet Apollo	568	1.27	-0.02	[-0.85 , 0.81]	Non-inferiority not shown	0.967
[MoP] SL-Plus Cementless Stem EP-Fit Plus	672	4.08	2.79	[1.48 , 4.11]	Inferior by ≥100%	<0.001
[MoP] Stanmore Modular Stem Stanmore-Arcom Cup	805	1.43	0.15	[-0.59 , 0.89]	Non-inferiority not shown	0.697
[MoP] Synergy Cementless Stem Reflection Cementless	546	2.18	0.90	[-0.31 , 2.10]	Non-inferiority not shown	0.146
[MoP] Taperloc Cementless Stem Exceed ABT	657	1.71	0.42	[-0.23 , 1.08]	Non-inferiority not shown	0.208
[MoP] Versys Cementless Stem Trilogy	336	4.56	3.28	[1.20 , 5.36]	Inferior by ≥20%	0.002

For peer review only

Supplemental table 6c: Difference in Kaplan-Meier failure estimate between a contemporary reference and implants with at least 250 at risk at 7 years since primary in males

Stem/cup brand	Number at risk	Cumulative failure (%)	Difference in failure (%)	95% CI	Equivalence status	p-value
[CoP] Furlong HAC Stem CSF	1,823	1.64	[REFERENCE]			
[CoC] ABG II Monolithic Cementless Stem ABG II Cementless Cup	469	3.52	1.89	[0.29 , 3.48]	Non-inferiority not shown	0.020
[CoC] ABG II Monolithic Cementless Stem Trident	365	4.06	2.42	[0.64 , 4.20]	Inferior by ≥20%	0.008
[CoC] Accolade Trident	1,234	3.47	1.84	[1.01 , 2.66]	Inferior by ≥20%	<0.001
[CoC] Corail Duraloc Option	354	4.27	2.64	[0.74 , 4.54]	Inferior by ≥20%	0.006
[CoC] Corail Pinnacle	3,192	3.28	1.64	[1.05 , 2.23]	Inferior by ≥20%	<0.001
[CoC] Exeter V40 ABG II Cementless Cup	304	1.98	0.35	[-1.05 , 1.75]	Non-inferiority not shown	0.627
[CoC] Exeter V40 Trident	2,289	2.18	0.54	[-0.12 , 1.20]	Non-inferiority not shown	0.108
[CoC] Furlong HAC Stem CSF	630	3.34	1.71	[0.30 , 3.11]	Non-inferiority not shown	0.017
[CoC] Furlong HAC Stem Furlong HAC CSF Plus	913	2.10	0.47	[-0.18 , 1.11]	Non-inferiority not shown	0.158
[CoC] SL-Plus Cementless Stem EP-Fit Plus	483	6.96	5.33	[3.28 , 7.38]	Inferior by ≥100%	<0.001
[CoC] Taperloc Cementless Stem Exceed ABT	628	2.14	0.51	[-0.23 , 1.24]	Non-inferiority not shown	0.175
[CoP] C-Stem Cemented Stem Wroblewski Golf Ball	252	1.47	-0.17	[-1.44 , 1.11]	Non-inferiority not shown	0.796
[CoP] Corail Pinnacle	611	2.30	0.66	[-0.12 , 1.45]	Non-inferiority not shown	0.098
[CoP] Exeter V40 Exeter Contemporary Flanged	417	0.85	-0.79	[-1.46 , -0.11]	Non-inferior	0.022
[CoP] Exeter V40 Trident	459	1.46	-0.18	[-1.01 , 0.65]	Non-inferiority not shown	0.676
[CoP] Exeter V40 Trilogy	438	2.16	0.53	[-0.58 , 1.64]	Non-inferiority not shown	0.351
[MoP] Accolade Trident	1,060	4.24	2.60	[1.71 , 3.49]	Inferior by ≥100%	<0.001
[MoP] C-Stem Cemented Stem Charnley and Elite Plus LPW	384	1.01	-0.62	[-1.63 , 0.39]	Non-inferiority not shown	0.227
[MoP] C-Stem Cemented Stem Elite Plus Ogee	654	1.54	-0.09	[-0.94 , 0.76]	Non-inferiority not shown	0.830
[MoP] C-Stem Cemented Stem Opera	282	2.55	0.92	[-0.56 , 2.40]	Non-inferiority not shown	0.225
[MoP] CPT Elite Plus Ogee	436	2.51	0.87	[-0.29 , 2.03]	Non-inferiority not shown	0.140
[MoP] CPT Trilogy	1,134	2.68	1.04	[0.28 , 1.81]	Non-inferiority not shown	0.008
[MoP] CPT ZCA	1,009	3.27	1.63	[0.71 , 2.56]	Inferior by ≥20%	0.001
[MoP] Charnley Cemented Stem Charnley Cemented Cup	1,126	2.94	1.31	[0.31 , 2.30]	Non-inferiority not shown	0.010
[MoP] Charnley Cemented Stem Charnley Ogee	2,211	3.41	1.77	[0.96 , 2.58]	Inferior by ≥20%	<0.001
[MoP] Charnley Cemented Stem Charnley and Elite Plus LPW	1,116	1.90	0.26	[-0.57 , 1.09]	Non-inferiority not shown	0.539
[MoP] Charnley Cemented Stem Wroblewski Golf Ball	256	2.09	0.46	[-1.07 , 1.98]	Non-inferiority not shown	0.559
[MoP] Corail Duraloc Cementless Cup	967	3.62	1.98	[0.84 , 3.13]	Inferior by ≥20%	0.001
[MoP] Corail Pinnacle	2,826	2.42	0.79	[0.21 , 1.37]	Non-inferiority not shown	0.008
[MoP] Corail Trilogy	323	3.87	2.23	[0.58 , 3.89]	Inferior by ≥20%	0.008
[MoP] Exeter V40 Cenator Cemented Cup	415	2.35	0.71	[-0.55 , 1.97]	Non-inferiority not shown	0.267
[MoP] Exeter V40 Charnley and Elite Plus LPW	294	1.33	-0.30	[-1.29 , 0.68]	Non-inferiority not shown	0.547
[MoP] Exeter V40 Elite Plus Cemented Cup	608	1.20	-0.44	[-1.20 , 0.33]	Non-inferiority not shown	0.266
[MoP] Exeter V40 Elite Plus Ogee	2,790	1.68	0.04	[-0.56 , 0.64]	Non-inferiority not shown	0.888
[MoP] Exeter V40 Exeter Contemporary Flanged	5,272	2.01	0.37	[-0.18 , 0.92]	Non-inferiority not shown	0.183
[MoP] Exeter V40 Exeter Contemporary Hooded	2,263	2.84	1.20	[0.53 , 1.88]	Inferior by ≥20%	<0.001
[MoP] Exeter V40 Exeter Duration	2,100	3.36	1.72	[0.96 , 2.49]	Inferior by ≥20%	<0.001
[MoP] Exeter V40 Opera	340	2.14	0.50	[-0.75 , 1.75]	Non-inferiority not shown	0.432
[MoP] Exeter V40 Reflection Cementless	483	3.34	1.70	[0.29 , 3.12]	Non-inferiority not shown	0.018

For peer review only

[MoP] Exeter V40 Trident	2,065	2.04	0.41	[-0.20 , 1.01]	Non-inferiority not shown	0.191
[MoP] Exeter V40 Trilogy	1,688	1.88	0.25	[-0.44 , 0.93]	Non-inferiority not shown	0.477
[MoP] Exeter V40 Ultima Cemented Cup	282	2.77	1.13	[-0.55 , 2.82]	Non-inferiority not shown	0.188
[MoP] Furlong Cemented Stem JRI Cemented Cup	312	1.77	0.13	[-1.18 , 1.45]	Non-inferiority not shown	0.843
[MoP] Furlong HAC Stem CSF	1,556	3.09	1.46	[0.62 , 2.29]	Inferior by ≥20%	0.001
[MoP] Furlong HAC Stem Furlong HAC CSF Plus	265	3.66	2.02	[0.80 , 3.25]	Inferior by ≥20%	0.001
[MoP] Muller-Biomet Apollo	376	1.46	-0.18	[-1.17 , 0.81]	Non-inferiority not shown	0.722
[MoP] SL-Plus Cementless Stem EP-Fit Plus	483	5.32	3.69	[2.08 , 5.29]	Inferior by ≥100%	<0.001
[MoP] Stanmore Modular Stem Stanmore-Arcom Cup	529	1.86	0.23	[-0.74 , 1.20]	Non-inferiority not shown	0.644
[MoP] Synergy Cementless Stem Reflection Cementless	396	2.37	0.73	[-0.59 , 2.05]	Non-inferiority not shown	0.277
[MoP] Versys Cementless Stem Trilogy	297	4.56	2.93	[0.81 , 5.05]	Inferior by ≥20%	0.007

For peer review only

Supplemental table 6d: Difference in Kaplan-Meier failure estimate between a contemporary reference and implants with at least 250 at risk at 10 years since primary in males

Stem/cup brand	Number at risk	Cumulative failure (%)	Difference in failure (%)	95% CI	Equivalence status	p-value
[CoP] Furlong HAC Stem CSF	1,054	2.03	[REFERENCE]			
[CoC] ABG II Monolithic Cementless Stem ABG II Cementless Cup	348	5.43	3.40	[1.33 , 5.47]	Inferior by ≥20%	0.001
[CoC] Corail Pinnacle	363	4.24	2.21	[1.28 , 3.15]	Inferior by ≥20%	<0.001
[CoC] Exeter V40 Trident	767	2.76	0.74	[-0.10 , 1.57]	Non-inferiority not shown	0.083
[CoC] Furlong HAC Stem CSF	360	4.28	2.25	[0.60 , 3.90]	Inferior by ≥20%	0.008
[MoP] C-Stem Cemented Stem Elite Plus Ogee	313	2.71	0.68	[-0.63 , 1.98]	Non-inferiority not shown	0.308
[MoP] CPT Trilogy	382	4.61	2.58	[1.24 , 3.92]	Inferior by ≥20%	<0.001
[MoP] CPT ZCA	332	4.41	2.38	[1.15 , 3.61]	Inferior by ≥20%	<0.001
[MoP] Charnley Cemented Stem Charnley Cemented Cup	615	4.69	2.66	[1.32 , 4.00]	Inferior by ≥20%	<0.001
[MoP] Charnley Cemented Stem Charnley Ogee	1,151	5.08	3.05	[2.00 , 4.10]	Inferior by ≥20%	<0.001
[MoP] Charnley Cemented Stem Charnley and Elite Plus LPW	661	2.93	0.90	[-0.21 , 2.01]	Non-inferiority not shown	0.114
[MoP] Corail Duraloc Cementless Cup	418	6.69	4.66	[2.92 , 6.41]	Inferior by ≥100%	<0.001
[MoP] Corail Pinnacle	638	3.49	1.47	[0.63 , 2.30]	Inferior by ≥20%	0.001
[MoP] Exeter V40 Elite Plus Ogee	1,105	2.53	0.51	[-0.31 , 1.32]	Non-inferiority not shown	0.226
[MoP] Exeter V40 Exeter Contemporary Flanged	1,517	2.88	0.85	[0.13 , 1.57]	Non-inferiority not shown	0.020
[MoP] Exeter V40 Exeter Contemporary Hooded	760	4.17	2.15	[1.19 , 3.11]	Inferior by ≥20%	<0.001
[MoP] Exeter V40 Exeter Duration	888	4.95	2.92	[1.88 , 3.96]	Inferior by ≥20%	<0.001
[MoP] Exeter V40 Trident	402	2.84	0.81	[-0.12 , 1.74]	Non-inferiority not shown	0.089
[MoP] Exeter V40 Trilogy	583	3.02	0.99	[0.02 , 1.96]	Non-inferiority not shown	0.045
[MoP] Furlong HAC Stem CSF	609	4.76	2.73	[1.56 , 3.90]	Inferior by ≥20%	<0.001

For peer review only

Supplemental table 7a: Difference in Kaplan-Meier failure estimate between a contemporary reference and implants with at least 250 at risk at 3 years since primary in males <55 years

Stem/cup brand	Number at risk	Cumulative failure (%)	Difference in failure (%)	95% CI	Equivalence status	p-value
[CoC] Exeter V40 Trident	1,269	1.26	[REFERENCE]			
[CoC] Accolade Trident	730	2.96	1.70	[0.41 , 3.00]	Inferior by ≥20%	0.010
[CoC] Corail Pinnacle	3,796	2.29	1.03	[0.33 , 1.74]	Inferior by ≥20%	0.004
[CoC] Furlong HAC Stem Furlong HAC CSF Plus	837	1.71	0.46	[-0.49 , 1.41]	Non-inferiority not shown	0.345
[CoC] M/L Taper Cementless Continuum	296	2.21	0.95	[-0.59 , 2.49]	Non-inferiority not shown	0.226
[CoC] Taperloc Cementless Stem Exceed ABT	836	2.23	0.98	[-0.05 , 2.00]	Non-inferiority not shown	0.062
[CoP] Accolade Trident	251	2.12	0.87	[-0.64 , 2.37]	Non-inferiority not shown	0.259
[CoP] Corail Pinnacle	509	1.90	0.64	[-0.34 , 1.62]	Non-inferiority not shown	0.201
[CoP] Exeter V40 Trident	369	0.81	-0.44	[-1.25 , 0.37]	Non-inferiority not shown	0.286
[MoP] Corail Pinnacle	417	1.20	-0.06	[-1.12 , 1.00]	Non-inferiority not shown	0.915

For peer review only

Supplemental table 7b: Difference in Kaplan-Meier failure estimate between a contemporary reference and implants with at least 250 at risk at 5 years since primary in males <55 years

Stem/cup brand	Number at risk	Cumulative failure (%)	Difference in failure (%)	95% CI	Equivalence status	p-value
[CoC] Corail Pinnacle	2,313	3.33	[REFERENCE]			
[CoC] Accolade Trident	502	4.15	0.82	[-0.70 , 2.34]	Non-inferiority not shown	0.290
[CoC] Exeter V40 Trident	934	1.87	-1.46	[-2.36 , -0.56]	Non-inferior	0.001
[CoC] Furlong HAC Stem Furlong HAC CSF Plus	494	1.85	-1.48	[-2.45 , -0.50]	Non-inferior	0.003
[CoC] Taperloc Cementless Stem Exceed ABT	430	2.57	-0.76	[-1.88 , 0.35]	Non-inferior	0.179

For peer review only

Supplemental table 8a: Difference in Kaplan-Meier failure estimate between a contemporary reference and implants with at least 250 at risk at 3 years since primary in males between 55 and 75 years

Stem/cup brand	Number at risk	Cumulative failure (%)	Difference in failure (%)	95% CI	Equivalence status	p-value
[CoP] Exeter V40 Trident	1,339	0.83	[REFERENCE]			
[CoC] ABG II Monolithic Cementless Stem ABG II Cementless Cup	408	2.58	1.75	[0.21 , 3.30]	Inferior by ≥20%	0.026
[CoC] ABG II Monolithic Cementless Stem Trident	368	1.82	0.99	[-0.39 , 2.37]	Non-inferiority not shown	0.159
[CoC] Accolade Trident	2,101	2.16	1.33	[0.64 , 2.02]	Inferior by ≥20%	<0.001
[CoC] Bimetric Cementless Stem Exceed ABT	279	0.60	-0.22	[-1.13 , 0.68]	Non-inferiority not shown	0.628
[CoC] Corail Duraloc Option	311	1.86	1.04	[-0.48 , 2.55]	Non-inferiority not shown	0.181
[CoC] Corail Pinnacle	8,280	1.71	0.89	[0.46 , 1.31]	Inferior by ≥20%	<0.001
[CoC] Excia Cementless Plasmacup SC	284	1.25	0.42	[-0.72 , 1.57]	Non-inferiority not shown	0.468
[CoC] Exeter V40 ABG II Cementless Cup	324	0.80	-0.03	[-0.99 , 0.93]	Non-inferiority not shown	0.949
[CoC] Exeter V40 Trident	2,957	1.06	0.23	[-0.26 , 0.72]	Non-inferiority not shown	0.350
[CoC] Furlong HAC Stem CSF	452	3.36	2.53	[0.88 , 4.19]	Inferior by ≥100%	0.003
[CoC] Furlong HAC Stem Furlong HAC CSF Plus	2,839	1.63	0.80	[0.27 , 1.33]	Inferior by ≥20%	0.003
[CoC] M/L Taper Cementless Continuum	405	1.41	0.58	[-0.45 , 1.61]	Non-inferiority not shown	0.269
[CoC] Polarstem Cementless R3 Cementless	256	0.54	-0.29	[-1.11 , 0.54]	Non-inferiority not shown	0.496
[CoC] SL-Plus Cementless Stem EP-Fit Plus	398	4.90	4.07	[2.00 , 6.14]	Inferior by ≥100%	<0.001
[CoC] Taperloc Cementless Stem Exceed ABT	2,008	1.40	0.58	[0.02 , 1.13]	Non-inferiority not shown	0.041
[CoP] Accolade Trident	975	1.56	0.73	[0.03 , 1.44]	Non-inferiority not shown	0.042
[CoP] C-Stem Cemented Stem Marathon	344	0.69	-0.14	[-0.92 , 0.64]	Non-inferiority not shown	0.726
[CoP] C-Stem Cemented Stem Wroblewski Golf Ball	273	0.31	-0.52	[-1.21 , 0.17]	Non-inferiority not shown	0.142
[CoP] CPT Trilogy	274	1.73	0.90	[-0.14 , 1.94]	Non-inferiority not shown	0.089
[CoP] Corail Marathon	368	1.84	1.02	[-0.12 , 2.15]	Non-inferiority not shown	0.079
[CoP] Corail Pinnacle	2,678	0.94	0.11	[-0.33 , 0.54]	Non-inferiority not shown	0.630
[CoP] Exeter V40 Charnley and Elite Plus LPW	275	1.16	0.33	[-0.85 , 1.52]	Non-inferiority not shown	0.585
[CoP] Exeter V40 Elite Plus Ogee	420	0.56	-0.27	[-0.99 , 0.45]	Non-inferiority not shown	0.467
[CoP] Exeter V40 Exeter Contemporary Flanged	919	0.88	0.06	[-0.57 , 0.68]	Non-inferiority not shown	0.862
[CoP] Exeter V40 Exeter X3 Rimfit	649	1.27	0.44	[-0.27 , 1.15]	Non-inferiority not shown	0.225
[CoP] Exeter V40 Trilogy	599	1.36	0.53	[-0.42 , 1.48]	Non-inferiority not shown	0.274
[CoP] Furlong HAC Stem CSF	1,990	0.93	0.11	[-0.43 , 0.64]	Non-inferiority not shown	0.699
[CoP] Furlong HAC Stem Furlong HAC CSF Plus	563	1.40	0.58	[-0.29 , 1.44]	Non-inferiority not shown	0.193
[CoP] MS-30 Original ME Muller Low Profile Cup	375	0.21	-0.62	[-1.16 , -0.07]	Non-inferior	0.026
[CoP] SL-Plus Cementless Stem EP-Fit Plus	268	1.45	0.62	[-0.83 , 2.07]	Non-inferiority not shown	0.404
[CoP] Taperloc Cementless Stem Exceed ABT	601	1.04	0.21	[-0.48 , 0.89]	Non-inferiority not shown	0.552
[MoP] Accolade Trident	2,467	2.30	1.47	[0.83 , 2.12]	Inferior by ≥100%	<0.001
[MoP] Anthology R3 Cementless	292	2.08	1.25	[0.01 , 2.49]	Non-inferiority not shown	0.048
[MoP] C-Stem AMT Cemented Stem Charnley and Elite Plus LPW	419	1.68	0.85	[-0.36 , 2.06]	Non-inferiority not shown	0.167
[MoP] C-Stem AMT Cemented Stem Marathon	256	1.18	0.35	[-0.62 , 1.32]	Non-inferiority not shown	0.475
[MoP] C-Stem AMT Cemented Stem Pinnacle	322	1.15	0.32	[-0.55 , 1.19]	Non-inferiority not shown	0.474
[MoP] C-Stem Cemented Stem Charnley and Elite Plus LPW	322	1.16	0.34	[-0.85 , 1.52]	Non-inferiority not shown	0.579
[MoP] C-Stem Cemented Stem Elite Plus Ogee	830	1.26	0.43	[-0.36 , 1.23]	Non-inferiority not shown	0.283

For peer review only

[MoP] C-Stem Cemented Stem Marathon	418	1.27	0.44	[-0.53 , 1.41]	Non-inferiority not shown	0.372
[MoP] C-Stem Cemented Stem Opera	391	1.47	0.64	[-0.58 , 1.86]	Non-inferiority not shown	0.301
[MoP] CPT Elite Plus Ogee	550	1.47	0.64	[-0.37 , 1.65]	Non-inferiority not shown	0.217
[MoP] CPT Trilogy	1,745	1.63	0.80	[0.18 , 1.42]	Inferior by ≥20%	0.012
[MoP] CPT ZCA	1,031	1.26	0.43	[-0.25 , 1.12]	Non-inferiority not shown	0.213
[MoP] Charnley Cemented Stem Charnley Cemented Cup	1,058	1.60	0.77	[-0.04 , 1.58]	Non-inferiority not shown	0.063
[MoP] Charnley Cemented Stem Charnley Ogee	2,239	1.38	0.55	[-0.03 , 1.13]	Non-inferiority not shown	0.064
[MoP] Charnley Cemented Stem Charnley and Elite Plus LPW	1,040	0.88	0.05	[-0.59 , 0.69]	Non-inferiority not shown	0.886
[MoP] Charnley Cemented Stem Opera	280	1.36	0.53	[-0.84 , 1.90]	Non-inferiority not shown	0.446
[MoP] Corail Duraloc Cementless Cup	978	1.96	1.13	[0.21 , 2.05]	Inferior by ≥20%	0.016
[MoP] Corail Elite Plus Ogee	308	1.86	1.03	[-0.38 , 2.44]	Non-inferiority not shown	0.153
[MoP] Corail Marathon	645	1.37	0.54	[-0.27 , 1.35]	Non-inferiority not shown	0.188
[MoP] Corail Pinnacle	7,845	1.52	0.69	[0.28 , 1.11]	Inferior by ≥20%	0.001
[MoP] Corail Trilogy	517	1.76	0.93	[-0.20 , 2.07]	Non-inferiority not shown	0.108
[MoP] Exeter V40 Cenator Cemented Cup	395	1.46	0.63	[-0.58 , 1.84]	Non-inferiority not shown	0.307
[MoP] Exeter V40 Charnley and Elite Plus LPW	397	1.70	0.87	[-0.35 , 2.09]	Non-inferiority not shown	0.164
[MoP] Exeter V40 Duraloc Cementless Cup	265	2.54	1.71	[-0.18 , 3.60]	Non-inferiority not shown	0.076
[MoP] Exeter V40 Elite Plus Cemented Cup	749	0.95	0.12	[-0.62 , 0.86]	Non-inferiority not shown	0.748
[MoP] Exeter V40 Elite Plus Ogee	3,262	1.05	0.22	[-0.26 , 0.69]	Non-inferiority not shown	0.369
[MoP] Exeter V40 Exeter Contemporary Flanged	8,180	1.18	0.35	[-0.05 , 0.75]	Non-inferiority not shown	0.090
[MoP] Exeter V40 Exeter Contemporary Hooded	2,960	1.74	0.91	[0.36 , 1.45]	Inferior by ≥20%	0.001
[MoP] Exeter V40 Exeter Duration	2,482	1.53	0.70	[0.13 , 1.27]	Non-inferiority not shown	0.016
[MoP] Exeter V40 Exeter X3 Rimfit	1,213	1.48	0.65	[0.03 , 1.28]	Non-inferiority not shown	0.039
[MoP] Exeter V40 Marathon	261	0.86	0.03	[-0.80 , 0.85]	Non-inferiority not shown	0.946
[MoP] Exeter V40 Opera	414	0.71	-0.12	[-0.99 , 0.75]	Non-inferiority not shown	0.785
[MoP] Exeter V40 Pinnacle	462	1.75	0.92	[-0.12 , 1.97]	Non-inferiority not shown	0.084
[MoP] Exeter V40 Reflection Cementless	467	1.44	0.61	[-0.50 , 1.72]	Non-inferiority not shown	0.284
[MoP] Exeter V40 Trident	4,119	1.23	0.40	[-0.04 , 0.85]	Non-inferiority not shown	0.074
[MoP] Exeter V40 Trilogy	2,325	1.03	0.20	[-0.32 , 0.71]	Non-inferiority not shown	0.455
[MoP] Furlong Cemented Stem JRI Cemented Cup	250	1.52	0.69	[-0.83 , 2.21]	Non-inferiority not shown	0.371
[MoP] Furlong HAC Stem CSF	1,718	1.67	0.84	[0.17 , 1.51]	Inferior by ≥20%	0.014
[MoP] Furlong HAC Stem Furlong HAC CSF Plus	689	2.04	1.21	[0.27 , 2.14]	Inferior by ≥20%	0.011
[MoP] M/L Taper Cementless Allofit	280	1.41	0.58	[-0.69 , 1.85]	Non-inferiority not shown	0.373
[MoP] Muller-Biomet Apollo	460	1.24	0.41	[-0.63 , 1.46]	Non-inferiority not shown	0.438
[MoP] Polarstem Cementless R3 Cementless	330	1.22	0.39	[-0.35 , 1.13]	Non-inferiority not shown	0.302
[MoP] SL-Plus Cementless Stem EP-Fit Plus	642	3.41	2.58	[1.24 , 3.91]	Inferior by ≥100%	<0.001
[MoP] Stanmore Modular Stem Stanmore-Arcom Cup	546	0.90	0.07	[-0.72 , 0.87]	Non-inferiority not shown	0.856
[MoP] Synergy Cementless Stem R3 Cementless	318	1.21	0.38	[-0.54 , 1.29]	Non-inferiority not shown	0.416
[MoP] Synergy Cementless Stem Reflection Cementless	439	1.54	0.71	[-0.47 , 1.89]	Non-inferiority not shown	0.239
[MoP] Taperloc Cementless Stem Exceed ABT	971	1.32	0.49	[-0.18 , 1.17]	Non-inferiority not shown	0.154
[MoP] Versys Cementless Stem Trilogy	282	3.38	2.55	[0.46 , 4.64]	Inferior by ≥20%	0.017

For peer review only

Supplemental table 8b: Difference in Kaplan-Meier failure estimate between a contemporary reference and implants with at least 250 at risk at 5 years since primary in males between 55 and 75 years

Stem/cup brand	Number at risk	Cumulative failure (%)	Difference in failure (%)	95% CI	Equivalence status	p-value
[CoP] Furlong HAC Stem CSF	1,759	1.09	[REFERENCE]			
[CoC] ABG II Monolithic Cementless Stem ABG II Cementless Cup	392	2.82	1.73	[0.09 , 3.37]	Non-inferiority not shown	0.038
[CoC] ABG II Monolithic Cementless Stem Trident	306	3.89	2.80	[0.74 , 4.85]	Inferior by ≥20%	0.008
[CoC] Accolade Trident	1,624	2.87	1.77	[0.94 , 2.60]	Inferior by ≥20%	<0.001
[CoC] Corail Duraloc Option	303	2.50	1.41	[-0.36 , 3.18]	Non-inferiority not shown	0.119
[CoC] Corail Pinnacle	5,229	2.37	1.28	[0.74 , 1.82]	Inferior by ≥20%	<0.001
[CoC] Exeter V40 ABG II Cementless Cup	269	1.15	0.06	[-1.16 , 1.28]	Non-inferiority not shown	0.924
[CoC] Exeter V40 Trident	2,279	1.74	0.65	[0.00 , 1.29]	Non-inferiority not shown	0.049
[CoC] Furlong HAC Stem CSF	438	3.80	2.70	[0.93 , 4.48]	Inferior by ≥20%	0.003
[CoC] Furlong HAC Stem Furlong HAC CSF Plus	1,705	1.86	0.76	[0.13 , 1.40]	Non-inferiority not shown	0.018
[CoC] SL-Plus Cementless Stem EP-Fit Plus	367	5.89	4.79	[2.51 , 7.08]	Inferior by ≥100%	<0.001
[CoC] Taperloc Cementless Stem Exceed ABT	1,161	1.61	0.52	[-0.15 , 1.18]	Non-inferiority not shown	0.127
[CoP] Accolade Trident	422	1.94	0.85	[-0.03 , 1.72]	Non-inferiority not shown	0.057
[CoP] Corail Pinnacle	1,340	1.58	0.48	[-0.14 , 1.11]	Non-inferiority not shown	0.131
[CoP] Exeter V40 Elite Plus Ogee	294	0.88	-0.21	[-1.21 , 0.79]	Non-inferiority not shown	0.680
[CoP] Exeter V40 Exeter Contemporary Flanged	635	0.88	-0.21	[-0.90 , 0.48]	Non-inferiority not shown	0.548
[CoP] Exeter V40 Trident	670	0.97	-0.12	[-0.75 , 0.51]	Non-inferiority not shown	0.701
[CoP] Exeter V40 Trilogy	492	1.71	0.62	[-0.48 , 1.72]	Non-inferiority not shown	0.272
[CoP] Furlong HAC Stem Furlong HAC CSF Plus	327	1.60	0.51	[-0.48 , 1.50]	Non-inferiority not shown	0.313
[CoP] MS-30 Original ME Muller Low Profile Cup	267	0.21	-0.88	[-1.49 , -0.27]	Non-inferior	0.005
[MoP] Accolade Trident	1,703	3.33	2.24	[1.42 , 3.05]	Inferior by ≥100%	<0.001
[MoP] C-Stem AMT Cemented Stem Charnley and Elite Plus LPW	283	1.68	0.58	[-0.65 , 1.82]	Non-inferiority not shown	0.355
[MoP] C-Stem Cemented Stem Charnley and Elite Plus LPW	296	1.16	0.07	[-1.15 , 1.29]	Non-inferiority not shown	0.911
[MoP] C-Stem Cemented Stem Elite Plus Ogee	639	1.26	0.17	[-0.67 , 1.01]	Non-inferiority not shown	0.696
[MoP] C-Stem Cemented Stem Opera	288	2.29	1.20	[-0.35 , 2.75]	Non-inferiority not shown	0.129
[MoP] CPT Elite Plus Ogee	426	2.12	1.02	[-0.25 , 2.30]	Non-inferiority not shown	0.116
[MoP] CPT Trilogy	1,247	2.23	1.14	[0.35 , 1.92]	Inferior by ≥20%	0.005
[MoP] CPT ZCA	825	1.91	0.81	[-0.09 , 1.71]	Non-inferiority not shown	0.076
[MoP] Charnley Cemented Stem Charnley Cemented Cup	942	2.86	1.76	[0.67 , 2.85]	Inferior by ≥20%	0.002
[MoP] Charnley Cemented Stem Charnley Ogee	1,978	2.45	1.35	[0.58 , 2.13]	Inferior by ≥20%	0.001
[MoP] Charnley Cemented Stem Charnley and Elite Plus LPW	929	1.28	0.19	[-0.62 , 0.99]	Non-inferiority not shown	0.650
[MoP] Corail Duraloc Cementless Cup	923	2.79	1.69	[0.58 , 2.80]	Inferior by ≥20%	0.003
[MoP] Corail Marathon	326	1.77	0.67	[-0.34 , 1.69]	Non-inferiority not shown	0.193
[MoP] Corail Pinnacle	4,602	1.85	0.76	[0.24 , 1.28]	Inferior by ≥20%	0.004
[MoP] Corail Trilogy	393	2.43	1.34	[-0.05 , 2.72]	Non-inferiority not shown	0.059
[MoP] Exeter V40 Cenator Cemented Cup	337	1.97	0.87	[-0.55 , 2.30]	Non-inferiority not shown	0.229
[MoP] Exeter V40 Charnley and Elite Plus LPW	317	1.70	0.60	[-0.65 , 1.86]	Non-inferiority not shown	0.347
[MoP] Exeter V40 Duraloc Cementless Cup	252	2.92	1.83	[-0.22 , 3.87]	Non-inferiority not shown	0.080
[MoP] Exeter V40 Elite Plus Cemented Cup	583	1.39	0.30	[-0.64 , 1.23]	Non-inferiority not shown	0.533

For peer review only

[MoP] Exeter V40 Elite Plus Ogee	2,569	1.35	0.26	[-0.33 , 0.85]	Non-inferiority not shown	0.390
[MoP] Exeter V40 Exeter Contemporary Flanged	5,599	1.66	0.56	[0.04 , 1.08]	Non-inferiority not shown	0.033
[MoP] Exeter V40 Exeter Contemporary Hooded	2,246	2.26	1.17	[0.50 , 1.84]	Inferior by ≥20%	0.001
[MoP] Exeter V40 Exeter Duration	1,970	2.29	1.20	[0.47 , 1.92]	Inferior by ≥20%	0.001
[MoP] Exeter V40 Opera	298	1.53	0.43	[-0.86 , 1.73]	Non-inferiority not shown	0.512
[MoP] Exeter V40 Pinnacle	305	2.50	1.40	[0.04 , 2.77]	Non-inferiority not shown	0.044
[MoP] Exeter V40 Reflection Cementless	426	2.31	1.22	[-0.21 , 2.64]	Non-inferiority not shown	0.094
[MoP] Exeter V40 Trident	2,530	1.68	0.59	[0.02 , 1.16]	Non-inferiority not shown	0.044
[MoP] Exeter V40 Trilogy	1,851	1.21	0.12	[-0.50 , 0.74]	Non-inferiority not shown	0.703
[MoP] Furlong HAC Stem CSF	1,448	1.97	0.88	[0.11 , 1.65]	Non-inferiority not shown	0.026
[MoP] Furlong HAC Stem Furlong HAC CSF Plus	430	3.06	1.97	[0.70 , 3.23]	Inferior by ≥20%	0.002
[MoP] Muller-Biomet Apollo	375	1.47	0.37	[-0.80 , 1.54]	Non-inferiority not shown	0.531
[MoP] SL-Plus Cementless Stem EP-Fit Plus	554	4.71	3.62	[2.00 , 5.24]	Inferior by ≥100%	<0.001
[MoP] Stanmore Modular Stem Stanmore-Arcom Cup	434	1.51	0.41	[-0.67 , 1.50]	Non-inferiority not shown	0.455
[MoP] Synergy Cementless Stem Reflection Cementless	407	2.46	1.37	[-0.14 , 2.87]	Non-inferiority not shown	0.075
[MoP] Taperloc Cementless Stem Exceed ABT	448	1.64	0.55	[-0.32 , 1.41]	Non-inferiority not shown	0.214
[MoP] Versys Cementless Stem Trilogy	261	3.73	2.64	[0.43 , 4.85]	Inferior by ≥20%	0.019

For peer review only

Supplemental table 8c: Difference in Kaplan-Meier failure estimate between a contemporary reference and implants with at least 250 at risk at 7 years since primary in males between 55 and 75 years

Stem/cup brand	Number at risk	Cumulative failure (%)	Difference in failure (%)	95% CI	Equivalence status	p-value
[CoP] Furlong HAC Stem CSF	1,463	1.34	[REFERENCE]			
[CoC] ABG II Monolithic Cementless Stem ABG II Cementless Cup	345	3.88	2.53	[0.60 , 4.47]	Inferior by ≥20%	0.010
[CoC] Accolade Trident	907	3.15	1.81	[0.90 , 2.71]	Inferior by ≥20%	<0.001
[CoC] Corail Pinnacle	2,130	2.66	1.32	[0.70 , 1.94]	Inferior by ≥20%	<0.001
[CoC] Exeter V40 Trident	1,626	2.00	0.66	[-0.06 , 1.38]	Non-inferiority not shown	0.074
[CoC] Furlong HAC Stem CSF	415	4.02	2.68	[0.84 , 4.53]	Inferior by ≥20%	0.004
[CoC] Furlong HAC Stem Furlong HAC CSF Plus	674	2.13	0.79	[0.06 , 1.51]	Non-inferiority not shown	0.034
[CoC] SL-Plus Cementless Stem EP-Fit Plus	322	6.44	5.09	[2.69 , 7.50]	Inferior by ≥100%	<0.001
[CoC] Taperloc Cementless Stem Exceed ABT	469	1.99	0.65	[-0.20 , 1.49]	Non-inferiority not shown	0.132
[CoP] Corail Pinnacle	480	2.24	0.90	[-0.00 , 1.80]	Non-inferiority not shown	0.051
[CoP] Exeter V40 Exeter Contemporary Flanged	335	0.88	-0.46	[-1.19 , 0.27]	Non-inferiority not shown	0.218
[CoP] Exeter V40 Trident	362	1.17	-0.17	[-0.95 , 0.61]	Non-inferiority not shown	0.668
[CoP] Exeter V40 Trilogy	350	1.92	0.57	[-0.62 , 1.77]	Non-inferiority not shown	0.347
[MoP] Accolade Trident	787	3.94	2.60	[1.64 , 3.55]	Inferior by ≥100%	<0.001
[MoP] C-Stem Cemented Stem Charnley and Elite Plus LPW	272	1.16	-0.18	[-1.42 , 1.07]	Non-inferiority not shown	0.779
[MoP] C-Stem Cemented Stem Elite Plus Ogee	494	1.45	0.11	[-0.84 , 1.06]	Non-inferiority not shown	0.818
[MoP] CPT Elite Plus Ogee	325	2.35	1.01	[-0.37 , 2.38]	Non-inferiority not shown	0.151
[MoP] CPT Trilogy	815	2.41	1.06	[0.21 , 1.92]	Non-inferiority not shown	0.015
[MoP] CPT ZCA	599	2.69	1.35	[0.23 , 2.46]	Non-inferiority not shown	0.018
[MoP] Charnley Cemented Stem Charnley Cemented Cup	813	3.75	2.41	[1.14 , 3.68]	Inferior by ≥20%	<0.001
[MoP] Charnley Cemented Stem Charnley Ogee	1,632	3.68	2.34	[1.39 , 3.29]	Inferior by ≥100%	<0.001
[MoP] Charnley Cemented Stem Charnley and Elite Plus LPW	776	1.98	0.64	[-0.37 , 1.64]	Non-inferiority not shown	0.212
[MoP] Corail Duraloc Cementless Cup	750	3.84	2.50	[1.18 , 3.82]	Inferior by ≥20%	<0.001
[MoP] Corail Pinnacle	2,172	2.41	1.07	[0.44 , 1.70]	Inferior by ≥20%	0.001
[MoP] Corail Trilogy	254	3.58	2.24	[0.45 , 4.03]	Inferior by ≥20%	0.014
[MoP] Exeter V40 Cenator Cemented Cup	263	2.66	1.32	[-0.40 , 3.04]	Non-inferiority not shown	0.134
[MoP] Exeter V40 Elite Plus Cemented Cup	400	1.39	0.05	[-0.92 , 1.02]	Non-inferiority not shown	0.920
[MoP] Exeter V40 Elite Plus Ogee	1,861	1.79	0.45	[-0.24 , 1.14]	Non-inferiority not shown	0.203
[MoP] Exeter V40 Exeter Contemporary Flanged	3,658	2.15	0.81	[0.21 , 1.42]	Non-inferiority not shown	0.008
[MoP] Exeter V40 Exeter Contemporary Hooded	1,560	2.93	1.58	[0.78 , 2.38]	Inferior by ≥20%	<0.001
[MoP] Exeter V40 Exeter Duration	1,396	3.26	1.92	[1.02 , 2.82]	Inferior by ≥20%	<0.001
[MoP] Exeter V40 Reflection Cementless	339	2.55	1.21	[-0.31 , 2.73]	Non-inferiority not shown	0.117
[MoP] Exeter V40 Trident	1,455	1.83	0.48	[-0.16 , 1.12]	Non-inferiority not shown	0.140
[MoP] Exeter V40 Trilogy	1,299	1.71	0.37	[-0.38 , 1.12]	Non-inferiority not shown	0.332
[MoP] Furlong HAC Stem CSF	1,139	2.72	1.37	[0.45 , 2.30]	Inferior by ≥20%	0.004
[MoP] Muller-Biomet Apollo	277	1.47	0.13	[-1.07 , 1.32]	Non-inferiority not shown	0.835
[MoP] SL-Plus Cementless Stem EP-Fit Plus	411	6.01	4.67	[2.79 , 6.55]	Inferior by ≥100%	<0.001
[MoP] Stanmore Modular Stem Stanmore-Arcom Cup	312	2.00	0.66	[-0.64 , 1.96]	Non-inferiority not shown	0.320
[MoP] Synergy Cementless Stem Reflection Cementless	298	2.71	1.37	[-0.23 , 2.96]	Non-inferiority not shown	0.093

For peer review only - http://bmjopen.bmj.com/site/about/guidelines.xhtml

For peer review only

Supplemental table 8d: Difference in Kaplan-Meier failure estimate between a contemporary reference and implants with at least 250 at risk at 10 years since primary in males between 55 and 75 years

Stem/cup brand	Number at risk	Cumulative failure (%)	Difference in failure (%)	95% CI	Equivalence status	p-value
[MoP] Exeter V40 Exeter Contemporary Flanged	1,182	3.08	[REFERENCE]			
[CoC] ABG II Monolithic Cementless Stem ABG II Cementless Cup	257	5.45	2.38	[0.02 , 4.73]	Non-inferiority not shown	0.048
[CoC] Exeter V40 Trident	545	2.49	-0.59	[-1.46 , 0.27]	Non-inferior	0.179
[CoP] Furlong HAC Stem CSF	855	1.75	-1.32	[-2.14 , -0.51]	Non-inferior	0.001
[MoP] C-Stem Cemented Stem Elite Plus Ogee	254	2.48	-0.60	[-2.00 , 0.80]	Non-inferiority not shown	0.400
[MoP] CPT Trilogy	302	3.83	0.75	[-0.59 , 2.10]	Non-inferiority not shown	0.272
[MoP] Charnley Cemented Stem Charnley Cemented Cup	486	5.76	2.68	[1.04 , 4.31]	Inferior by ≥20%	0.001
[MoP] Charnley Cemented Stem Charnley Ogee	910	5.47	2.39	[1.21 , 3.58]	Inferior by ≥20%	<0.001
[MoP] Charnley Cemented Stem Charnley and Elite Plus LPW	500	3.21	0.13	[-1.19 , 1.45]	Non-inferiority not shown	0.846
[MoP] Corail Duraloc Cementless Cup	336	7.31	4.23	[2.19 , 6.27]	Inferior by ≥20%	<0.001
[MoP] Corail Pinnacle	545	3.39	0.31	[-0.53 , 1.15]	Non-inferiority not shown	0.468
[MoP] Exeter V40 Elite Plus Ogee	805	2.81	-0.27	[-1.18 , 0.65]	Non-inferiority not shown	0.568
[MoP] Exeter V40 Exeter Contemporary Hooded	600	4.02	0.94	[-0.09 , 1.98]	Non-inferiority not shown	0.074
[MoP] Exeter V40 Exeter Duration	642	5.21	2.14	[0.92 , 3.35]	Inferior by ≥20%	0.001
[MoP] Exeter V40 Trident	335	2.78	-0.30	[-1.32 , 0.72]	Non-inferiority not shown	0.566
[MoP] Exeter V40 Trilogy	482	3.05	-0.03	[-1.09 , 1.03]	Non-inferiority not shown	0.956
[MoP] Furlong HAC Stem CSF	483	4.63	1.55	[0.22 , 2.89]	Non-inferiority not shown	0.023

For peer review only

Supplemental table 9a: Difference in Kaplan-Meier failure estimate between a contemporary reference and implants with at least 250 at risk at 3 years since primary in males >75 years

Stem/cup brand	Number at risk	Cumulative failure (%)	Difference in failure (%)	95% CI	Equivalence status	p-value
[MoP] Exeter V40 Elite Plus Ogee	2,199	0.78	[REFERENCE]			
[CoC] Corail Pinnacle	540	2.35	1.57	[0.38 , 2.76]	Inferior by ≥20%	0.010
[CoC] Furlong HAC Stem Furlong HAC CSF Plus	350	2.46	1.68	[0.26 , 3.10]	Inferior by ≥20%	0.020
[CoP] Corail Pinnacle	306	1.43	0.65	[-0.25 , 1.56]	Non-inferiority not shown	0.159
[CoP] Furlong HAC Stem CSF	326	1.19	0.41	[-0.68 , 1.51]	Non-inferiority not shown	0.456
[MoP] Accolade Trident	911	2.78	2.00	[1.01 , 3.00]	Inferior by ≥100%	<0.001
[MoP] C-Stem AMT Cemented Stem Charnley and Elite Plus LPW	293	1.38	0.60	[-0.65 , 1.85]	Non-inferiority not shown	0.347
[MoP] C-Stem AMT Cemented Stem Elite Plus Ogee	265	0.56	-0.22	[-1.07 , 0.63]	Non-inferiority not shown	0.618
[MoP] C-Stem Cemented Stem Elite Plus Ogee	365	1.07	0.29	[-0.70 , 1.28]	Non-inferiority not shown	0.563
[MoP] CPT Elite Plus Ogee	287	2.08	1.30	[-0.26 , 2.87]	Non-inferiority not shown	0.103
[MoP] CPT Trilogy	894	1.93	1.15	[0.34 , 1.97]	Inferior by ≥20%	0.005
[MoP] CPT ZCA	1,080	1.70	0.93	[0.22 , 1.63]	Inferior by ≥20%	0.010
[MoP] Charnley Cemented Stem Charnley Cemented Cup	398	0.92	0.14	[-0.82 , 1.10]	Non-inferiority not shown	0.771
[MoP] Charnley Cemented Stem Charnley Ogee	959	1.32	0.54	[-0.20 , 1.28]	Non-inferiority not shown	0.154
[MoP] Charnley Cemented Stem Charnley and Elite Plus LPW	559	1.07	0.29	[-0.56 , 1.14]	Non-inferiority not shown	0.507
[MoP] Corail Duraloc Cementless Cup	280	0.64	-0.14	[-1.08 , 0.81]	Non-inferiority not shown	0.780
[MoP] Corail Marathon	317	1.78	1.00	[-0.14 , 2.14]	Non-inferiority not shown	0.084
[MoP] Corail Pinnacle	2,860	1.59	0.81	[0.33 , 1.30]	Inferior by ≥20%	0.001
[MoP] Exeter V40 Cenator Cemented Cup	301	1.13	0.35	[-0.80 , 1.51]	Non-inferiority not shown	0.547
[MoP] Exeter V40 Charnley and Elite Plus LPW	263	0.27	-0.51	[-1.13 , 0.12]	Non-inferior	0.113
[MoP] Exeter V40 Elite Plus Cemented Cup	394	0.78	0.00	[-0.83 , 0.84]	Non-inferiority not shown	0.994
[MoP] Exeter V40 Exeter Contemporary Flanged	5,295	1.03	0.25	[-0.15 , 0.65]	Non-inferiority not shown	0.221
[MoP] Exeter V40 Exeter Contemporary Hooded	2,098	1.84	1.06	[0.48 , 1.64]	Inferior by ≥20%	<0.001
[MoP] Exeter V40 Exeter Duration	1,426	1.51	0.73	[0.08 , 1.39]	Non-inferiority not shown	0.028
[MoP] Exeter V40 Exeter X3 Rimfit	537	1.21	0.43	[-0.28 , 1.13]	Non-inferiority not shown	0.237
[MoP] Exeter V40 Opera	290	1.47	0.69	[-0.63 , 2.01]	Non-inferiority not shown	0.306
[MoP] Exeter V40 Pinnacle	288	1.15	0.37	[-0.62 , 1.36]	Non-inferiority not shown	0.460
[MoP] Exeter V40 Reflection Cementless	256	2.46	1.68	[-0.15 , 3.51]	Non-inferiority not shown	0.072
[MoP] Exeter V40 Trident	2,148	1.41	0.63	[0.11 , 1.15]	Non-inferiority not shown	0.018
[MoP] Exeter V40 Trilogy	867	1.15	0.37	[-0.36 , 1.10]	Non-inferiority not shown	0.319
[MoP] Furlong HAC Stem CSF	770	1.99	1.21	[0.27 , 2.16]	Inferior by ≥20%	0.012
[MoP] Furlong HAC Stem Furlong HAC CSF Plus	449	3.06	2.28	[1.00 , 3.56]	Inferior by ≥100%	<0.001
[MoP] Muller-Biomet Apollo	276	0.96	0.18	[-0.95 , 1.31]	Non-inferiority not shown	0.755
[MoP] Stanmore Modular Stem Stanmore-Arcom Cup	510	1.01	0.23	[-0.64 , 1.09]	Non-inferiority not shown	0.610
[MoP] Taperloc Cementless Stem Exceed ABT	421	1.75	0.97	[0.01 , 1.94]	Non-inferiority not shown	0.049

For peer review only

Supplemental table 9b: Difference in Kaplan-Meier failure estimate between a contemporary reference and implants with at least 250 at risk at 5 years since primary in males >75 years

Stem/cup brand	Number at risk	Cumulative failure (%)	Difference in failure (%)	95% CI	Equivalence status	p-value
[MoP] Exeter V40 Elite Plus Ogee	1,490	1.10	[REFERENCE]			
[CoC] Corail Pinnacle	321	2.82	1.73	[0.35 , 3.11]	Inferior by ≥20%	0.014
[MoP] Accolade Trident	540	3.41	2.32	[1.12 , 3.51]	Inferior by ≥100%	<0.001
[MoP] C-Stem Cemented Stem Elite Plus Ogee	253	1.45	0.36	[-0.91 , 1.62]	Non-inferiority not shown	0.579
[MoP] CPT Trilogy	535	2.58	1.48	[0.46 , 2.50]	Inferior by ≥20%	0.004
[MoP] CPT ZCA	693	2.90	1.80	[0.78 , 2.83]	Inferior by ≥20%	0.001
[MoP] Charnley Cemented Stem Charnley Cemented Cup	325	0.92	-0.17	[-1.16 , 0.82]	Non-inferiority not shown	0.731
[MoP] Charnley Cemented Stem Charnley Ogee	760	1.91	0.81	[-0.12 , 1.75]	Non-inferiority not shown	0.088
[MoP] Charnley Cemented Stem Charnley and Elite Plus LPW	446	1.43	0.33	[-0.68 , 1.35]	Non-inferiority not shown	0.521
[MoP] Corail Pinnacle	1,479	1.98	0.88	[0.27 , 1.49]	Inferior by ≥20%	0.005
[MoP] Exeter V40 Elite Plus Cemented Cup	271	1.04	-0.06	[-1.06 , 0.95]	Non-inferiority not shown	0.910
[MoP] Exeter V40 Exeter Contemporary Flanged	3,050	1.33	0.24	[-0.26 , 0.74]	Non-inferiority not shown	0.350
[MoP] Exeter V40 Exeter Contemporary Hooded	1,283	2.09	1.00	[0.32 , 1.68]	Inferior by ≥20%	0.004
[MoP] Exeter V40 Exeter Duration	1,041	2.08	0.98	[0.17 , 1.80]	Non-inferiority not shown	0.018
[MoP] Exeter V40 Trident	1,100	1.57	0.48	[-0.13 , 1.09]	Non-inferiority not shown	0.125
[MoP] Exeter V40 Trilogy	592	2.07	0.97	[-0.05 , 1.99]	Non-inferiority not shown	0.063
[MoP] Furlong HAC Stem CSF	561	2.27	1.17	[0.12 , 2.22]	Non-inferiority not shown	0.029
[MoP] Stanmore Modular Stem Stanmore-Arcom Cup	358	1.20	0.11	[-0.88 , 1.09]	Non-inferiority not shown	0.834

For peer review only

Supplemental table 9c: Difference in Kaplan-Meier failure estimate between a contemporary reference and implants with at least 250 at risk at 7 years since primary in males >75 years

Stem/cup brand	Number at risk	Cumulative failure (%)	Difference in failure (%)	95% CI	Equivalence status	p-value
[MoP] Exeter V40 Exeter Contemporary Flanged	1,540	1.76	[REFERENCE]			
[MoP] CPT Trilogy	264	3.16	1.40	[0.20 , 2.60]	Non-inferiority not shown	0.022
[MoP] CPT ZCA	390	3.94	2.18	[0.82 , 3.55]	Inferior by ≥20%	0.002
[MoP] Charnley Cemented Stem Charnley Cemented Cup	252	1.25	-0.51	[-1.68 , 0.66]	Non-inferiority not shown	0.393
[MoP] Charnley Cemented Stem Charnley Ogee	502	2.56	0.80	[-0.32 , 1.92]	Non-inferiority not shown	0.162
[MoP] Charnley Cemented Stem Charnley and Elite Plus LPW	308	1.43	-0.33	[-1.34 , 0.68]	Non-inferiority not shown	0.520
[MoP] Corail Pinnacle	542	2.34	0.58	[-0.14 , 1.30]	Non-inferiority not shown	0.116
[MoP] Exeter V40 Elite Plus Ogee	868	1.43	-0.33	[-0.99 , 0.32]	Non-inferior	0.315
[MoP] Exeter V40 Exeter Contemporary Hooded	670	2.53	0.77	[-0.02 , 1.57]	Non-inferiority not shown	0.056
[MoP] Exeter V40 Exeter Duration	643	3.08	1.32	[0.29 , 2.36]	Non-inferiority not shown	0.012
[MoP] Exeter V40 Trident	538	2.35	0.59	[-0.28 , 1.46]	Non-inferiority not shown	0.185
[MoP] Exeter V40 Trilogy	321	2.07	0.31	[-0.71 , 1.32]	Non-inferiority not shown	0.553
[MoP] Furlong HAC Stem CSF	358	3.06	1.30	[0.01 , 2.60]	Non-inferiority not shown	0.049

For peer review only

Supplemental table 10a: Total number of implants at risk at each time-point by age-group and gender in implants with at least 500

	Time since primary procedure			
	3 years	5 years	7 years	10 years
Male, <55	7,977	3,749	No data	No data
Male, 55-75	79,299	49,540	27,512	6,584
Male, 75+	23,653	13,124	5,303	No data
Total Male	132,073	82,003	42,294	10,348
Female, <55	10,007	6,016	2,303	No data
Female, 55-75	136,561	91,874	52,940	15,848
Female, 75+	64,959	37,694	19,779	No data
Total Female	244,285	156,505	91,701	24,408
Total	415,608	268,809	156,138	41,908

For peer review only

Supplemental table 10b: Total number of implant failures at each time-point by age-group and gender in implants with at least 500 at risk

	Time since primary procedure			
	3 years	5 years	7 years	10 years
Male, <55	260	132	68	53
Male, 55-75	1,039	533	364	442
Male, 75+	293	133	86	57
Total Male	1,618	811	529	560
Female, <55	268	141	102	79
Female, 55-75	1,344	784	539	545
Female, 75+	458	255	161	120
Total Female	2,091	1,189	820	754
Total	3,733	2,009	1,359	1,325

For peer review only

The RECORD statement – checklist of items, extended from the STROBE statement, that should be reported in observational studies using routinely collected health data.

	Item No.	STROBE items	Location in manuscript where items are reported	RECORD items	Location in manuscript where items are reported
**Title and abstract**					
	1	(a) Indicate the study's design with a commonly used term in the title or the abstract (b) Provide in the abstract an informative and balanced summary of what was done and what was found	Title page 1, abstract page 2.	RECORD 1.1: The type of data used should be specified in the title or abstract. When possible, the name of the databases used should be included.<br><br>RECORD 1.2: If applicable, the geographic region and timeframe within which the study took place should be reported in the title or abstract.<br><br>RECORD 1.3: If linkage between databases was conducted for the study, this should be clearly stated in the title or abstract.	Title, abstract page 2.<br><br>Abstract page 2<br><br>N/A
**Introduction**					
Background rationale	2	Explain the scientific background and rationale for the investigation being reported	Introduction Page 4		
Objectives	3	State specific objectives, including any prespecified hypotheses	Introduction Page 5		
**Methods**					
Study Design	4	Present key elements of study design early in the paper	Methods pages 7 to 8		
Setting	5	Describe the setting, locations, and relevant dates, including periods of recruitment, exposure, follow-up, and data collection	Methods page 6 and 7		
Participants	6	*(a) Cohort study* - Give the eligibility criteria, and the sources and methods of selection of participants. Describe methods of follow-up	Methods page 6	RECORD 6.1: The methods of study population selection (such as codes or algorithms used to identify subjects) should be listed in detail. If this is not possible, an	Methods Page 6, supplemental figure 1

For peer review only

		Case-control study - Give the eligibility criteria, and the sources and methods of case ascertainment and control selection. Give the rationale for the choice of cases and controls<br>*Cross-sectional study* - Give the eligibility criteria, and the sources and methods of selection of participants<br><br>*(b) Cohort study* - For matched studies, give matching criteria and number of exposed and unexposed<br>*Case-control study* - For matched studies, give matching criteria and the number of controls per case		explanation should be provided.<br><br>RECORD 6.2: Any validation studies of the codes or algorithms used to select the population should be referenced. If validation was conducted for this study and not published elsewhere, detailed methods and results should be provided.<br><br>RECORD 6.3: If the study involved linkage of databases, consider use of a flow diagram or other graphical display to demonstrate the data linkage process, including the number of individuals with linked data at each stage.	 <br>N/A<br><br><br><br><br>N/A
Variables	7	Clearly define all outcomes, exposures, predictors, potential confounders, and effect modifiers. Give diagnostic criteria, if applicable.	Methods page 6 and 7	RECORD 7.1: A complete list of codes and algorithms used to classify exposures, outcomes, confounders, and effect modifiers should be provided. If these cannot be reported, an explanation should be provided.	Methods page 6 (description of data selection, no codes)
Data sources/ measurement	8	For each variable of interest, give sources of data and details of methods of assessment (measurement). Describe comparability of assessment methods if there is more than one group	Methods Page 6		
Bias	9	Describe any efforts to address potential sources of bias			
Study size	10	Explain how the study size was arrived at	Supplemental figure 1		
Quantitative variables	11	Explain how quantitative variables were handled in the analyses. If applicable, describe which groupings were chosen, and why	Methods Page 6 and 7		
Statistical methods	12	(a) Describe all statistical methods, including those used to control for confounding<br>(b) Describe any methods used to	Methods Page 6 and 7		

For peer review only

		examine subgroups and interactions<br>(c) Explain how missing data were addressed<br>(d) *Cohort study* - If applicable, explain how loss to follow-up was addressed<br>*Case-control study* - If applicable, explain how matching of cases and controls was addressed<br>*Cross-sectional study* - If applicable, describe analytical methods taking account of sampling strategy<br>(e) Describe any sensitivity analyses			
Data access and cleaning methods		..		RECORD 12.1: Authors should describe the extent to which the investigators had access to the database population used to create the study population.<br><br>RECORD 12.2: Authors should provide information on the data cleaning methods used in the study.	Acknowledgement section; contributions, page 15<br><br>Methods Page 6
Linkage		..		RECORD 12.3: State whether the study included person-level, institutional-level, or other data linkage across two or more databases. The methods of linkage and methods of linkage quality evaluation should be provided.	No linkage
**Results**					
Participants	13	(a) Report the numbers of individuals at each stage of the study (*e.g.*, numbers potentially eligible, examined for eligibility, confirmed eligible, included in the study, completing follow-up, and analysed)<br>(b) Give reasons for non-participation	Supplemental figure 1 and Results page 8	RECORD 13.1: Describe in detail the selection of the persons included in the study (*i.e.,* study population selection) including filtering based on data quality, data availability and linkage. The selection of included persons can be described in the text and/or by means of the study flow diagram.	Methods, page 6; Results page 8 and supplemental figure 1

For peer review only - http://bmjopen.bmj.com/site/about/guidelines.xhtml

		at each stage.<br>(c) Consider use of a flow diagram			
Descriptive data	14	(a) Give characteristics of study participants (*e.g.*, demographic, clinical, social) and information on exposures and potential confounders<br>(b) Indicate the number of participants with missing data for each variable of interest<br>(c) *Cohort study* - summarise follow-up time (*e.g.*, average and total amount)			
Outcome data	15	*Cohort study* - Report numbers of outcome events or summary measures over time<br>*Case-control study* - Report numbers in each exposure category, or summary measures of exposure<br>*Cross-sectional study* - Report numbers of outcome events or summary measures	All supplemental tables; supplemental tables 10a and b		
Main results	16	(a) Give unadjusted estimates and, if applicable, confounder-adjusted estimates and their precision (e.g., 95% confidence interval). Make clear which confounders were adjusted for and why they were included<br>(b) Report category boundaries when continuous variables were categorized<br>(c) If relevant, consider translating estimates of relative risk into absolute risk for a meaningful time period	Results Pages 8 to 11, Supplemental tables		
Other analyses	17	Report other analyses done—e.g., analyses of subgroups and interactions, and sensitivity analyses	Results Page 11		
**Discussion**					
Key results	18	Summarise key results with reference to study objectives	Discussion Page 12		

For peer review only

Limitations	19	Discuss limitations of the study, taking into account sources of potential bias or imprecision. Discuss both direction and magnitude of any potential bias	Discussion Pages 13	RECORD 19.1: Discuss the implications of using data that were not created or collected to answer the specific research question(s). Include discussion of misclassification bias, unmeasured confounding, missing data, and changing eligibility over time, as they pertain to the study being reported.	Discussion Pages 12 to 13
Interpretation	20	Give a cautious overall interpretation of results considering objectives, limitations, multiplicity of analyses, results from similar studies, and other relevant evidence	Discussion Pages 11-13		
Generalisability	21	Discuss the generalisability (external validity) of the study results	Discussion Page 14		
**Other Information**					
Funding	22	Give the source of funding and the role of the funders for the present study and, if applicable, for the original study on which the present article is based	Page 1 and page 15		
Accessibility of protocol, raw data, and programming code		..	N/A	RECORD 22.1: Authors should provide information on how to access any supplemental information such as the study protocol, raw data, or programming code.	N/A

For peer review only
