# Supplementary material for: Assessing the non-inferiority of prosthesis constructs used in hip replacement using data from the National Joint Registry of England, Wales, Northern Ireland and the Isle of Man: a benchmarking study
Source: BMJ Open. 2019 Apr 29;9(4):e026685. doi: 10.1136/bmjopen-2018-026685 (PMC6502009; doi:10.1136/bmjopen-2018-026685)
Supplement: Reviewer comments [file bmjopen-2018-026685.reviewer_comments.pdf]

PEER REVIEW HISTORY

BMJ Open publishes all reviews undertaken for accepted manuscripts. Reviewers are asked to complete a checklist review form (**http://bmjopen.bmj.com/site/about/resources/checklist.pdf**) and are provided with free text boxes to elaborate on their assessment. These free text comments are reproduced below.

ARTICLE DETAILS

**TITLE (PROVISIONAL)**	Assessing the non-inferiority of prosthesis constructs used in hip replacement using data from the National Joint Registry of England, Wales, Northern Ireland and the Isle of Man: A Benchmarking study
**AUTHORS**	Deere, Kevin; Whitehouse, Michael; Porter, Martyn; Blom, AW; Sayers, Adrian

Sorry, there is no peer review to display for this article.

BMJ Open operates using a fully open peer review. This means that accepted papers usually have their prepublication history posted alongside them.

This prepublication history comprises all previous versions of the manuscript, the reviewers' comments, and the authors' responses to all the comments from reviewers and editors. For clinical trials, this also includes the study protocol (submitting the protocol is mandatory for all clinical trials and encouraged for all other studies at BMJ Open).

In rare instances we determine after careful consideration that we should not make certain portions of the prepublication record publicly available. For example, in cases of stigmatised illnesses we seek to protect the confidentiality of reviewers who have these illnesses. In other instances there may be legal or regulatory considerations that make it inadvisable or impermissible to make available certain parts of the prepublication record.

In all instances in which we have determined that elements of the prepublication record should not be made publicly available, we expect that authors will respect these decisions and also will not share this information.
